# A Tale of Two Loads: Modulation of IL-1 Induced Inflammatory Responses of Meniscal Cells in Two Models of Dynamic Physiologic Loading

**DOI:** 10.3389/fbioe.2022.837619

**Published:** 2022-03-01

**Authors:** Benjamin D. Andress, Rebecca M. Irwin, Ishaan Puranam, Brenton D. Hoffman, Amy L. McNulty

**Affiliations:** ^1^ Department of Pathology, Duke University, Durham, NC, United States; ^2^ Department of Orthopaedic Surgery, Duke University School of Medicine, Durham, NC, United States; ^3^ Department of Biomedical Engineering, Duke University, Durham, NC, United States; ^4^ Department of Cell Biology, Duke University, Durham, NC, United States

**Keywords:** inflammation, gene expression, NFATc2, Nos2, CXCL10, STAT, IRF1, cartilage

## Abstract

Meniscus injuries are highly prevalent, and both meniscus injury and subsequent surgery are linked to the development of post-traumatic osteoarthritis (PTOA). Although the pathogenesis of PTOA remains poorly understood, the inflammatory cytokine IL-1 is elevated in synovial fluid following acute knee injuries and causes degradation of meniscus tissue and inhibits meniscus repair. Dynamic mechanical compression of meniscus tissue improves integrative meniscus repair in the presence of IL-1 and dynamic tensile strain modulates the response of meniscus cells to IL-1. Despite the promising observed effects of physiologic mechanical loading on suppressing inflammatory responses of meniscus cells, there is a lack of knowledge on the global effects of loading on meniscus transcriptomic profiles. In this study, we compared two established models of physiologic mechanical stimulation, dynamic compression of tissue explants and cyclic tensile stretch of isolated meniscus cells, to identify conserved responses to mechanical loading. RNA sequencing was performed on loaded and unloaded meniscus tissue or isolated cells from inner and outer zones, with and without IL-1. Overall, results from both models showed significant modulation of inflammation-related pathways with mechanical stimulation. Anti-inflammatory effects of loading were well-conserved between the tissue compression and cell stretch models for inner zone; however, the cell stretch model resulted in a larger number of differentially regulated genes. Our findings on the global transcriptomic profiles of two models of mechanical stimulation lay the groundwork for future mechanistic studies of meniscus mechanotransduction, which may lead to the discovery of novel therapeutic targets for the treatment of meniscus injuries.

## 1 Introduction

Meniscus injuries are highly prevalent, affecting people of all ages and stages of life (Nielsen and Yde, 1991). Meniscus injury and subsequent surgery are linked to the development of post-traumatic osteoarthritis (PTOA), with 50% of partial meniscectomy patients developing radiographic osteoarthritis (OA) within 10–20 years following surgery (Lohmander et al., 2007). Therefore, more recent clinical treatments have focused on repair surgeries to preserve meniscal function as much as possible. Notably, the avascular inner zone of the meniscus heals poorly due to the lack of blood supply (Rath and Richmond, 2000), therefore repair of inner zone meniscal tears is still only recommended in limited cases (Gallacher et al., 2010; Ghazi Zadeh et al., 2018). Although the pathogenesis of PTOA following meniscus injury remains poorly understood, the involvement of inflammation-mediated tissue degradation is increasingly appreciated as a major contributing factor in disease progression. Indeed, it has been found that inflammatory cytokines, including IL-1, IL-6, IL-8, TNF-α, and PGE2 are elevated following acute knee injuries (Bigoni et al., 2013; Liu et al., 2017; Clair et al., 2019), chronic knee injuries (Larsson et al., 2015; Bigoni et al., 2017), and in OA knees (van den Berg et al., 1999). Even very low concentrations of IL-1 have been shown to cause degradation of meniscus tissue (McNulty et al., 2013) and inhibit repair in an explant model of meniscus injury (McNulty et al., 2007; Wilusz et al., 2008; McNulty et al., 2009).

There are no approved disease modifying drugs for OA or PTOA, so novel therapeutic targets to stimulate meniscus healing and break the injury, inflammation, and tissue damage cycle are greatly needed. One promising area of investigation for novel therapeutic targets are mechanosensors and mechanotransduction pathways (McNulty and Guilak, 2015). Dynamic mechanical compression of meniscus tissue has been shown to improve integrative repair in the presence of IL-1 in an explant model of meniscus injury (McNulty et al., 2010). Dynamic tensile strain has also been shown to modulate the response of meniscus cells to an inflammatory stimulus (Deschner et al., 2006; Ferretti et al., 2006; Madhavan et al., 2007), and studies have linked mechanical stimulation of meniscal cells to increased synthesis of extracellular matrix (ECM) (Fermor et al., 2004; Puetzer et al., 2012). In contrast, joint immobilization leads to meniscus atrophy and OA (Videman et al., 1979). Therefore, mechanotransduction pathways could represent novel therapeutic targets to modulate inflammation to limit degradation of meniscus tissue and stimulate regenerative healing of the meniscus. Further understanding of meniscal mechanobiology in both non-inflammatory and inflammatory conditions may provide insight into mechanisms to improve tissue repair after injury.

Despite the promising effects of physiologic loading on meniscus cells, there remains a dearth of knowledge on global effects of loading on meniscus cell phenotype and transcriptomic profiles. This may be due in large part to the lack of well-characterized models to study the effects of mechanical stimulation on meniscus cells. Indeed, the complex geometry and anisotropic nature of the meniscus makes it difficult to model the mechanical environment of meniscus cells. Meniscus cells *in vivo* are subjected to a variety of dynamic mechanical forces, including compression and stretch due to deformation of the tissue, and hydrostatic pressure and fluid flow as the highly hydrated tissue is compressed and water is extruded. Since it is challenging to replicate all of these forces simultaneously in a single *in vitro* model system, previous studies of meniscus mechanotransduction have employed a wide variety of methods to model individual aspects of physiologic load. These model systems include dynamic compression of tissue explants (Shin et al., 2003; Upton et al., 2003; McHenry et al., 2006; McNulty et al., 2010; Han et al., 2014), dynamic compression of cells embedded in engineered constructs (Ballyns and Bonassar, 2011), cyclic tensile strain of isolated meniscus cells grown on a flexible substrate (Fermor et al., 2004; Deschner et al., 2006; Ferretti et al., 2006; Furumatsu et al., 2012; Kanazawa et al., 2012), fluid flow stimulation of isolated cells (Eifler et al., 2006), and hydrostatic pressure on isolated cells in monolayer (Zhang et al., 2019) or engineered constructs (Gunja and Athanasiou, 2010). Overall these studies have shown that dynamic loads at strain levels thought to correspond to physiologic loading promote anabolism (Eifler et al., 2006; Ballyns and Bonassar, 2011; Furumatsu et al., 2012; Kanazawa et al., 2012; Puetzer et al., 2012) and reduce expression of pro-inflammatory mediators (Deschner et al., 2006; Ferretti et al., 2006; Madhavan et al., 2007), while hyperphysiologic strain magnitudes (McHenry et al., 2006) or static loading (Upton et al., 2003) induce expression of inflammatory mediators and catabolic enzymes that lead to breakdown of the ECM. Notably, only a few studies have compared the responses of inner and outer zone cells to mechanical stimulation (Furumatsu et al., 2012; Kanazawa et al., 2012). The meniscus inner and outer zones vary in regard to cellular phenotype (Upton et al., 2006a; Son and Levenston, 2012; Grogan et al., 2017; Andress et al., 2021), ECM composition, and mechanical properties (Cheung, 1987; Scott et al., 1997; Sanchez-Adams et al., 2011), and experience different forces *in vivo* (Upton et al., 2006b; Freutel et al., 2014). Therefore, the inner and outer zone cells may exhibit differences in mechanoresponsiveness.

**FIGURE 1:**
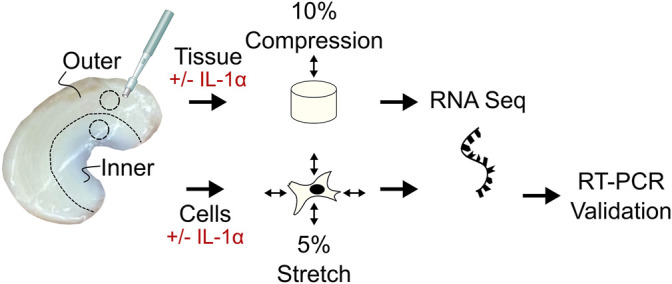
Study methods. Tissue explants and cells were harvested from the inner and outer zone of porcine menisci and subjected to dynamic cyclic loading (tissue: 10% axial compression, cells: 5% biaxial stretch) with or without IL-1α stimulation (0.1 ng/ml). RNA sequencing was performed on both tissue explants and cells to identify pathways and gene targets differentially expressed with load and IL-1α stimulation in inner and outer zones. For the cell stretch samples, RT-qPCR was performed to validate gene targets identified from RNA sequencing analysis.

In this study, we sought to compare two established models of dynamic physiologic loading of meniscus cells to identify conserved responses to mechanical loading. We utilized an unbiased approach by performing RNA sequencing (RNA-seq) on loaded and unloaded meniscus tissue explants or isolated cells from inner and outer zones, with and without IL-1 (Figure 1). The two *in vitro* models of dynamic loading employed in this study were cell stretch of isolated meniscus cells and a tissue explant compression model (McNulty et al., 2010). The *ex vivo* tissue compression model retains the native ECM microenvironment and ECM-cell interactions. This model may recapitulate more features of *in vivo* loading, as macro-scale tissue strain is transmitted to cells through the ECM and pericellular matrix, which may have either strain amplifying or attenuating properties (Upton et al., 2008; Han et al., 2013). Tissue explants from inner and outer zones of porcine medial menisci were stimulated with 10% dynamic compression, which is within the range of physiologic loading of the meniscus based on finite element modeling (Zielinska and Haut Donahue, 2005) and experimental data (Freutel et al., 2014). The cell stretch model may not recapitulate as many features of *in vivo* load, but allows for better control of experimental parameters, including intra-individual variability through pooling of cells from multiple subjects, and is not subject to variation due to irregularities in explants and individual menisci. Meniscus cells from inner and outer zones of porcine medial menisci were subjected to 5% dynamic equibiaxial stretch, which is within the range of strains predicted to be experienced at the cellular level for both inner and outer zone meniscus cells *in vivo* (Upton et al., 2006b). Both models were performed in the presence and absence of IL-1α at 0.1 ng/ml, which is a concentration observed in synovial fluid of moderate OA porcine knees (McNulty et al., 2013), has been shown to induce degradation of meniscus tissue *in vitro* (McNulty et al., 2013), and inhibits repair in an explant model of meniscus injury (McNulty et al., 2007; Wilusz et al., 2008; McNulty et al., 2010). The goals of this study were: 1) to identify IL-1 induced inflammatory responses modulated by load and 2) to determine conserved cellular responses to mechanical load in the presence and absence of IL-1 between two established models of mechanical stimulation in both the inner and outer zones of the meniscus.

## 2 Materials and Methods

### 2.1 Cell Stretch Experiments

#### 2.1.1 Meniscus Cell Isolation

Cells were isolated from the inner third and outer two-thirds of porcine medial menisci from skeletally mature female pigs obtained from a local abattoir, as previously described (Riera et al., 2011; McNulty et al., 2013; Andress et al., 2021). Briefly, menisci were separated into inner and outer regions and minced. Tissue was then digested by sequential 0.5% pronase (SKU 53702; EMD Millipore Corp., Temecula, CA) in Dulbecco’s Modified Eagle Medium, high glucose (DMEM-HG; Catalog # 11995073; Gibco, Carlsbad, CA) with 10X antibiotic/antimycotic (Catalog # 15240062; Gibco) for 1 h and then 0.2% collagenase type I (Catalog # LS004197; Worthington Biochemical Corp., Lakewood, NJ) in DMEM-HG with 10% fetal bovine serum (Catalog # SH30396.03; HyClone, Logan, UT) for approximately 16 h. Cells were isolated with a 70 μm cell strainer and frozen for later use.

#### 2.1.2 Cell Stretch

For each experiment, cells from at least three separate menisci were pooled and expanded for one passage in culture media containing DMEM-HG, 10% fetal bovine serum, 1× non-essential amino acids (Catalog # 11140050; Gibco), 1× antibiotic/antimycotic, 10 mM HEPES (Cat # 15630080; Gibco), and 40 μg/ml l-proline (SKU #H54409; Sigma-Aldrich, St. Louis, MO), and 50 μg/ml ascorbic acid (SKU # A8960; Sigma-Aldrich) added at time of use. Cells were then seeded onto Collagen type I coated Bioflex^®^ 6-well plates (Catalog # BF-3001C; Flexcell^®^ International Corporation, Burlington, NC) at a density of 250,000 cells/well (*n* = 3/group). Approximately 40 h after seeding, cells received fresh culture media, with or without recombinant porcine IL-1α (Catalog # 680-PI-010; R&D Systems, Minneapolis, MN) at a concentration of 0.1 ng/ml. Cells were then stimulated for a single bout of loading on a Flexcell^®^ FX-6000T™ with 5% equibiaxial dynamic strain in a sine wave pattern at 0.5 Hz for 4 h at 37°C (Agarwal et al., 2004; Furumatsu et al., 2012; Kanazawa et al., 2012). Unloaded cells (0%) with or without 0.1 ng/ml IL-1 were cultured in parallel on Bioflex plates in the same incubator.

#### 2.1.3 RNA Extraction

Immediately after loading, cells were lysed and total RNA was extracted and column purified according to the Norgen RNA extraction kit protocol (Catalog # 48300; Norgen Biotek Corp., Thorold, Canada).

### 2.2 Dynamic Compression Experiments

#### 2.2.1 Tissue Explant Harvest and Compression

Cylindrical explants from the inner and outer regions of six separate porcine medial menisci were obtained using a 5 mm biopsy punch (Catalog # 33-35; Integra, York, PA) and trimmed to a uniform 2 mm thickness retaining the femoral surface, using a custom cutting jig. Explants were distributed among treatment groups (loaded/unloaded, +/−IL-1), such that no group contained two explants from the same meniscus. Samples were washed for 1 h in DMEM-HG with 10X antibiotic/antimycotic, then transferred to culture media. The next day, fresh culture media with or without 0.1 ng/ml IL-1α was added and explants were subjected to 10% dynamic compression in a sine wave pattern at 1 Hz, using a custom bioreactor (McNulty et al., 2010), for 4 h at 37°C. Unloaded explants (0%) with or without 0.1 ng/ml IL-1 were cultured in parallel in the same incubator. Loading was repeated every 24 h for a total of three loading sessions. Immediately following the final loading session, explants were rinsed in phosphate buffered saline (PBS; catalog # 10010-023; Gibco), flash frozen in liquid nitrogen, and stored at −80°C until RNA extraction was performed.

#### 2.2.2 RNA Extraction

Explants were pulverized in TRIzol (Catalog # 15596026; Life Technologies, Carlsbad, CA) under liquid nitrogen using a freezer mill (Model 6875; SPEX SamplePrep, Metuchen, NJ) for three cycles of 2 min each at maximum frequency with 2 min precooling between cycles. RNA was extracted by TRIzol/chloroform separation, according to the manufacturer’s protocol. RNA was then resuspended in lysis buffer and column purified, according to the Norgen RNA extraction kit protocol (Catalog # 48300; Norgen Biotek Corp).

### 2.3 RNA-Sequencing (RNA-seq)

Stranded mRNA-sequencing was performed by the Duke Center for Genomic and Computational Biology (Durham, NC). All samples used for RNA-seq had an RNA integrity number (RIN) > 6.5. Tissue compression and cell stretch experiments were sequenced and analyzed separately. For each experiment, reads that were 20 nt or longer after trimming were mapped to the Sscrofa11.1v91 version of the pig genome and transcriptome (Kersey et al., 2012), using the STAR RNA-seq alignment tool (Dobin et al., 2013), and kept for subsequent analysis only if mapped to a single genomic location. Gene counts were compiled using the HTSeq tool, and genes that had at least 10 reads in any given library were used in subsequent analyses. Normalization and differential expression was performed using the DESeq2 (Love et al., 2014) Bioconductor (Huber et al., 2015) package within the R statistical programming environment. The false discovery rate (FDR) was calculated to control for multiple hypothesis testing. Genes with a base-2 log fold-change (LogFC) > 1 and FDR adjusted *p*-value <0.05 were considered significant. Gene set enrichment analysis (GSEA) (Mootha et al., 2003; Subramanian et al., 2005) was performed using the Hallmark pathways geneset database (Liberzon et al., 2015) comparing loaded/unloaded and IL-1 treated/untreated samples for each condition. Genesets with an FDR *q*-value < 0.25 were compared between the two loading models to identify genesets/pathways showing co-regulation (e.g., upregulated by loading in both models) or opposite regulation (e.g., upregulated by cell stretch, downregulated by tissue compression).

### 2.4 Reverse Transcriptase Quantitative PCR (RT-qPCR)

Genes selected from the RNA-sequencing results based on fold-change and potential biologic relevance were validated by RT-qPCR. Two hundred nanograms of total RNA per reaction was used to synthesize cDNA using the SuperScript VILO cDNA Synthesis Kit (Catalog # 11754050; Life Technologies). Then qPCR was performed using PowerUP SYBR Green master mix (Catalog # A25776; Thermo Fisher Scientific Baltics UAB, Vilnius, Lithuania) with gene specific primers (Supplementary Table 1) and the StepOne Plus real-time PCR system (Model 4376374; Applied Biosystems, Foster City, CA). Relative fold-change was determined by the 2^−∆∆Ct^ method (Livak and Schmittgen, 2001), using 18S as a reference gene. Results are presented as the base-2 log fold-change relative to the inner zone unloaded control group. Two-way ANOVAs were performed for each zone (inner and outer) separately where factors were load (0% and 5%) and IL-1 stimulation (+/−). A Tukey multiple comparison post hoc analysis was performed with targets that had a significant interaction term. For the targets that did not have a significant interaction term, factor level significance is depicted on the graph. ANOVA results for the *RRAD* target for outer zone samples are reported but failed the Shapiro-Wilk test for normality of residuals (*p* = 0.027, see Supplementary Figure 1 for QQ plot of residuals). ANOVA assumptions of homogeneity and normality were met for all other targets.

## 3 Results

**FIGURE 2:**
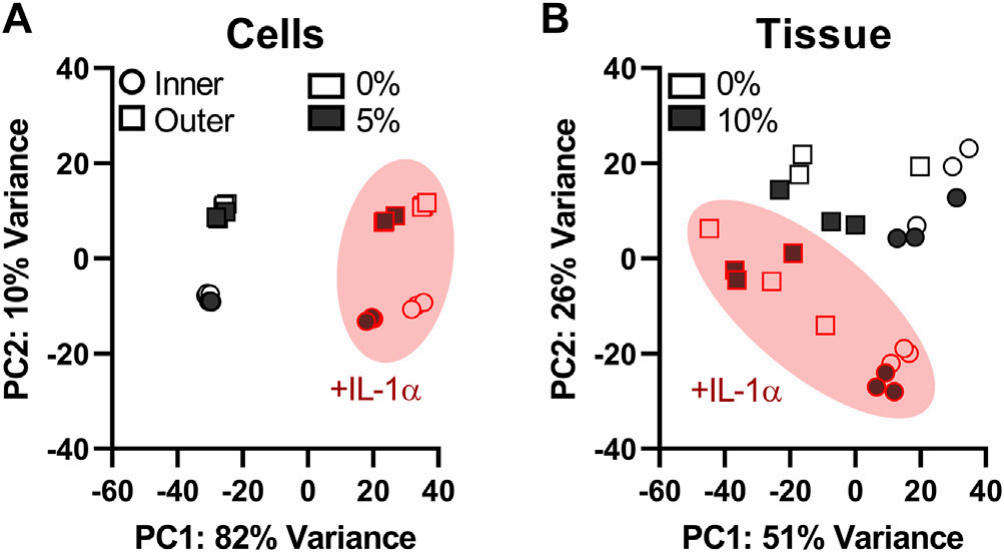
Effects of IL-1 treatment, zone, and dynamic load on global gene expression are more consistent in the cell stretch model than tissue compression. Principal component analysis (PCA) plots of the cell stretch experiment **(A)** show distinct clustering of samples by zone (inner and outer) and treatment (+/−IL-1, +/−dynamic loading). Samples from the tissue compression experiment **(B)** display considerable overlap between experimental groups. Shape corresponds to zone (circle: inner, square: outer), shape fill denotes load (unfilled: 0%, filled: loaded), and samples stimulated with IL-1α are outlined and circled in red. The *x*-axis (PC1) is the first principal direction along which samples from the RNA-seq experiment show the largest variation. The *y*-axis (PC2) is the second principal axis and the direction along which samples show the second largest variation. The total variability accounted for by each principal component is indicated in the axis labels.

In a principal component analysis (PCA) of the cell stretch model, experimental groups cluster distinctly by region (inner/outer) and treatment (+/−IL-1, +/−load) (Figure 2A). Principal component 1 (PC1), accounting for 82% of variance, appears to be dominated by the effect of IL-1 treatment, while PC2, accounting for 10% of variance, separates inner and outer zone cells. PCA of the tissue compression dataset shows much more overlap between samples in different experimental groups, indicating that there is considerable heterogeneity in gene expression within the experimental groups in this model (Figure 2B).

**FIGURE 3:**
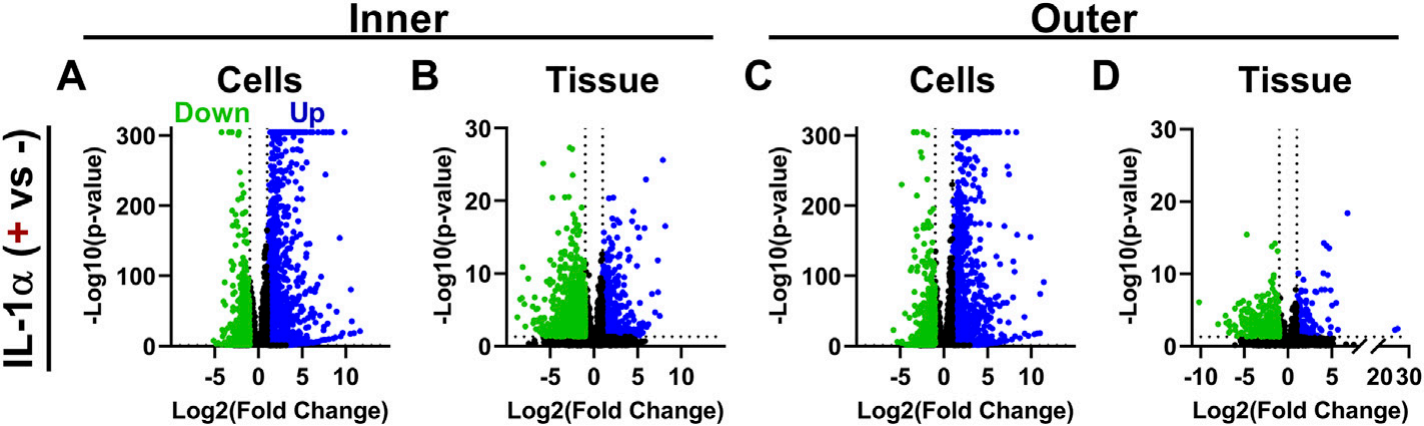
IL-1 treatment induces significant changes in gene expression. Volcano plots showing genes significantly up and downregulated by IL-1α (0.1 ng/ml) treatment in **(A)** inner zone cells in monolayer **(B)** inner zone tissue explants **(C)** outer zone cells in monolayer, and **(D)** outer zone tissue explants. Each data point is an individual gene; *y*-axis capped at 300 (adjusted *p*-value < 10^–300^). Color denotes if the gene was significantly upregulated (blue), downregulated (green), or not significant (black). Dashed lines indicate cutoffs for significant genes (FDR adjusted *p*-value < 0.05 and a base-2 log fold-change > |1|).

IL-1 treatment induced significant changes in expression for many genes in both monolayer cells and tissue explants. IL-1 treatment of inner zone cells resulted in significant downregulation (*p* < 0.05, LogFC < −1) of 976 genes and upregulation (*p* < 0.05, LogFC > 1) of 1,129 genes (Figure 3A). Treatment of inner zone tissue explants with IL-1 resulted in downregulation of 1,241 genes and upregulation of 526 genes (Figure 3B). Outer zone cells displayed similar numbers of differentially regulated genes upon treatment with IL-1, with 925 downregulated and 1,129 upregulated transcripts (Figure 3C). Outer zone tissue explants were slightly less responsive to IL-1 treatment, with 413 genes displaying downregulation and 169 showing upregulation (Figure 3D). IL-1 treatment induced upregulation of genes known to contribute to OA development, including inflammatory cytokines *IL6* and *LIF* (Kapoor et al., 2011), and degradative enzymes *MMP3* (Burrage et al., 2006) and *ADAMTS5* (Santamaria, 2020), in tissue and monolayer cells from inner and outer zones. Supplementary Data Sheets 1–4 provide lists of genes significantly differentially expressed with IL-1 treatment.

**FIGURE 4:**
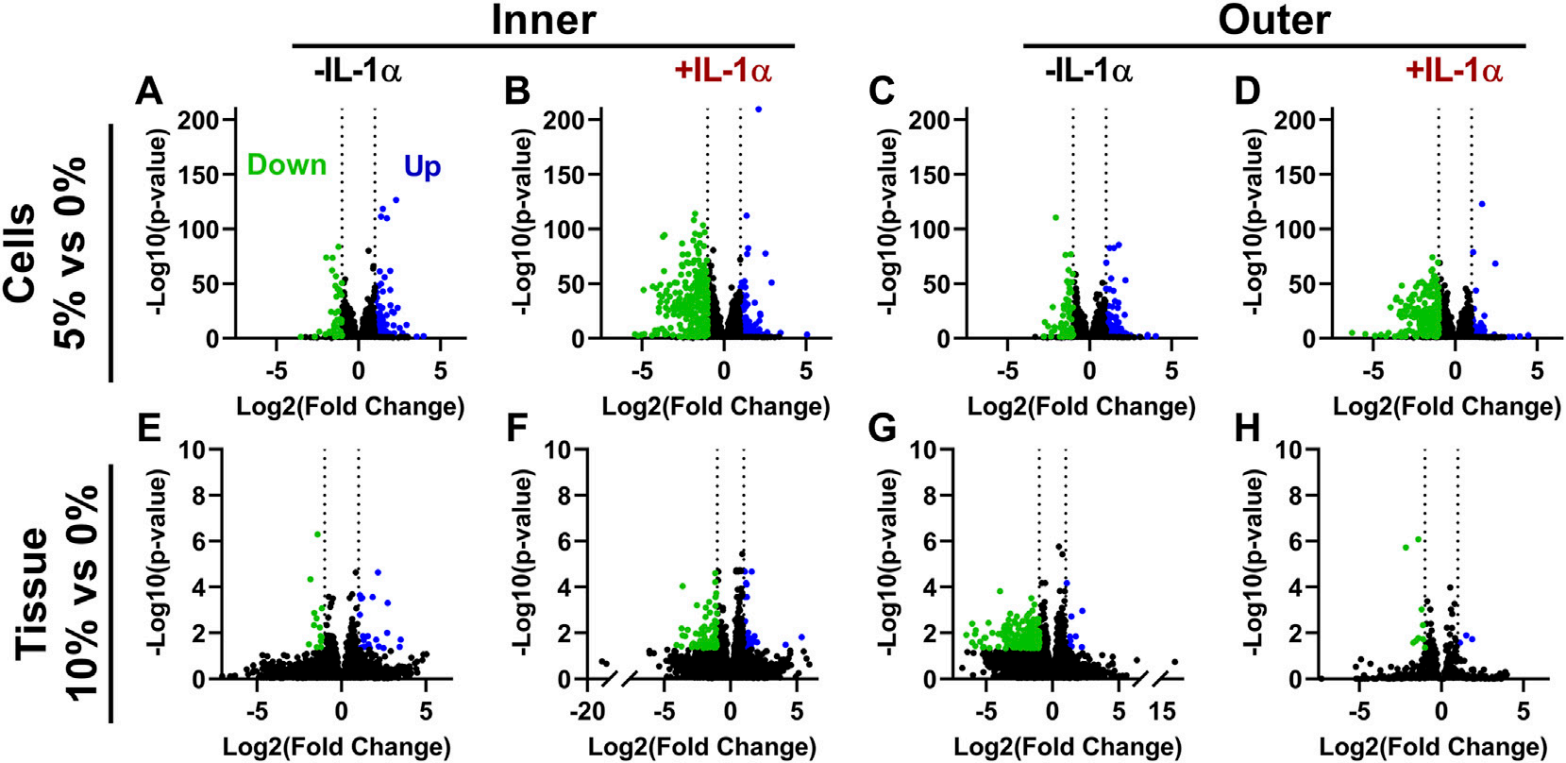
Dynamic loading downregulates gene expression in the presence of IL-1α. Volcano plots showing genes significantly up and downregulated by dynamic cell stretch of **(A)** inner zone cells **(B)** inner zone cells in the presence of IL-1α **(C)** outer zone cells, and **(D)** outer zone cells in the presence of IL-1α, or dynamic compression of **(E)** inner zone tissue **(F)** inner zone tissue in the presence IL-1α **(G)** outer zone tissue, and **(H)** outer zone tissue in the presence of IL-1α. Each data point is an individual gene. Color denotes if the gene was significantly up-regulated (blue), down-regulated (green), or not significant (black).

The effect of loading alone on global gene expression was modest in both models. There were only 158 transcripts showing significant differential expression with cell stretch and 35 with tissue compression (Figures 4A,F; LogFC>|1| and *p* < 0.05). Both models of dynamic loading (inner zone cell stretch and tissue compression) resulted in a larger number of downregulated transcripts in the presence of IL-1 (Figures 4B,F; LogFC < −1 and *p* < 0.05; 299 genes for cell stretch and 85 for tissue compression). Similarly, outer zone cells subjected to 5% stretch in the presence of IL-1 displayed a larger number of downregulated transcripts (Figure 4D; 242 genes with LogFC < −1 and *p* < 0.05) than outer zone cells subjected to loading in the absence of IL-1 (Figure 4C; 65 genes with LogFC < −1 and *p* < 0.05). However, compression of outer zone tissue induced a large number of downregulated genes in the absence of IL-1 (Figure 4G; 187 genes with LogFC < −1 and *p* < 0.05) but relatively little effect on overall gene expression in the presence of IL-1 (Figure 4H; 13 genes with LogFC > |1| and *p* < 0.05). Supplementary Data Sheets 5–12 provide lists of genes significantly differentially expressed with load.

**FIGURE 5:**
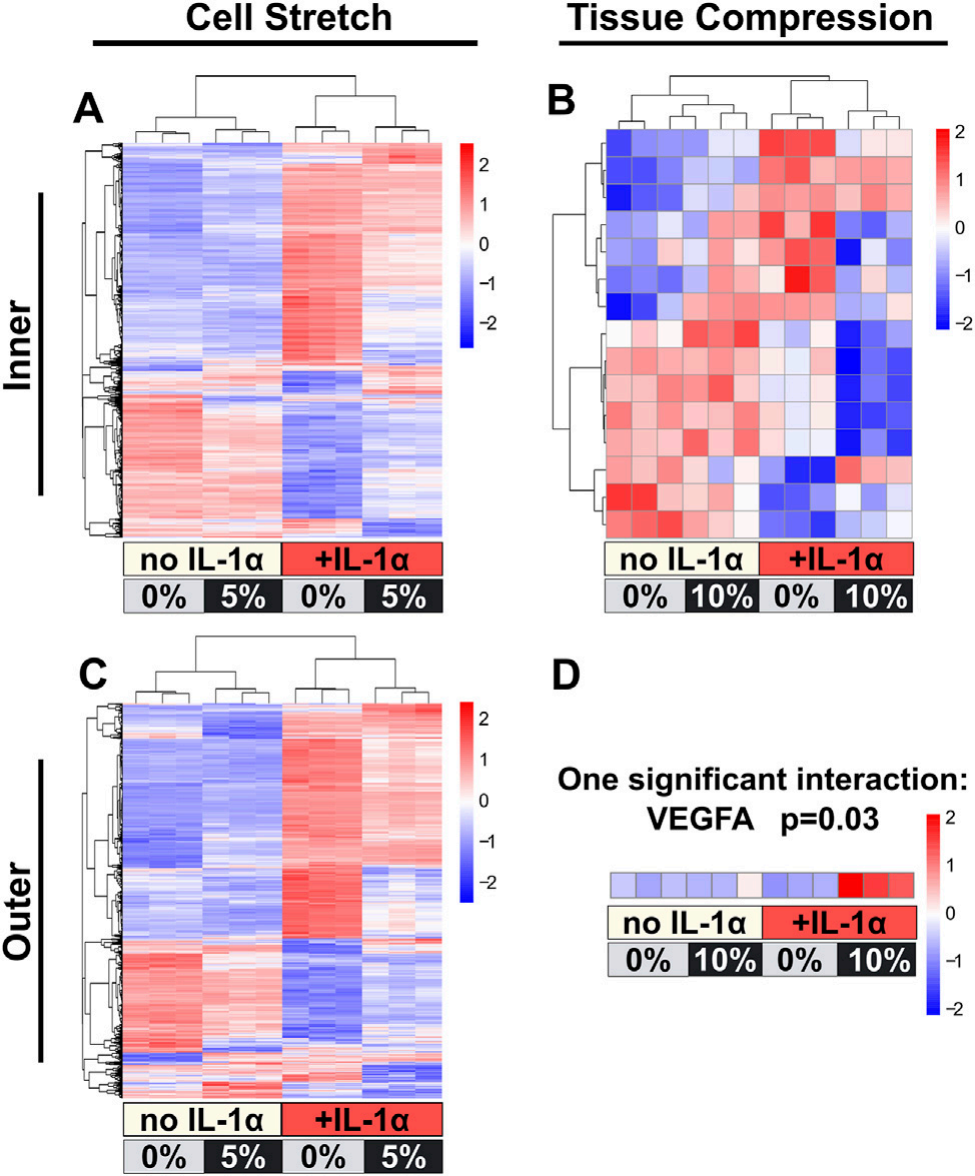
Dynamic cell stretch resulted in a larger number of differentially regulated genes with significant load and IL-1α interaction than dynamic tissue compression. Z-score heat maps for genes that had a significant interaction term between IL-1α and load are shown for cell stretch inner **(A)** and outer **(C)** zones, and tissue compression inner **(B)** and outer **(D)** zones. Cell stretch had a larger number of differentially regulated genes than tissue compression for both zones. Each column is an individual replicate. Hierarchical clustering grouped samples within their corresponding treatment (+/−IL-1α and +/−load).

In order to identify IL-1 regulated transcripts modulated by cell stretch or compression, genes with a significant interaction term between IL-1 treatment and dynamic load were identified from each dataset (*p* < 0.05, no LogFC cutoff). For cells subjected to 5% stretch, over 2,500 genes were identified with a significant interactive effect between load and IL-1 for inner zone (Figure 5A) and over 1,500 genes for outer zone (Figure 5C). For explants subjected to tissue compression, only 15 genes with a significant interactive effect were identified for inner zone (Figure 5B), and only one gene (VEGFA) displayed a significant interaction term for the outer zone (Figure 5D). Supplementary Data Sheets 13–15 provide lists of genes with a significant interaction between load and IL-1 treatment for each model and zone.

**FIGURE 6:**
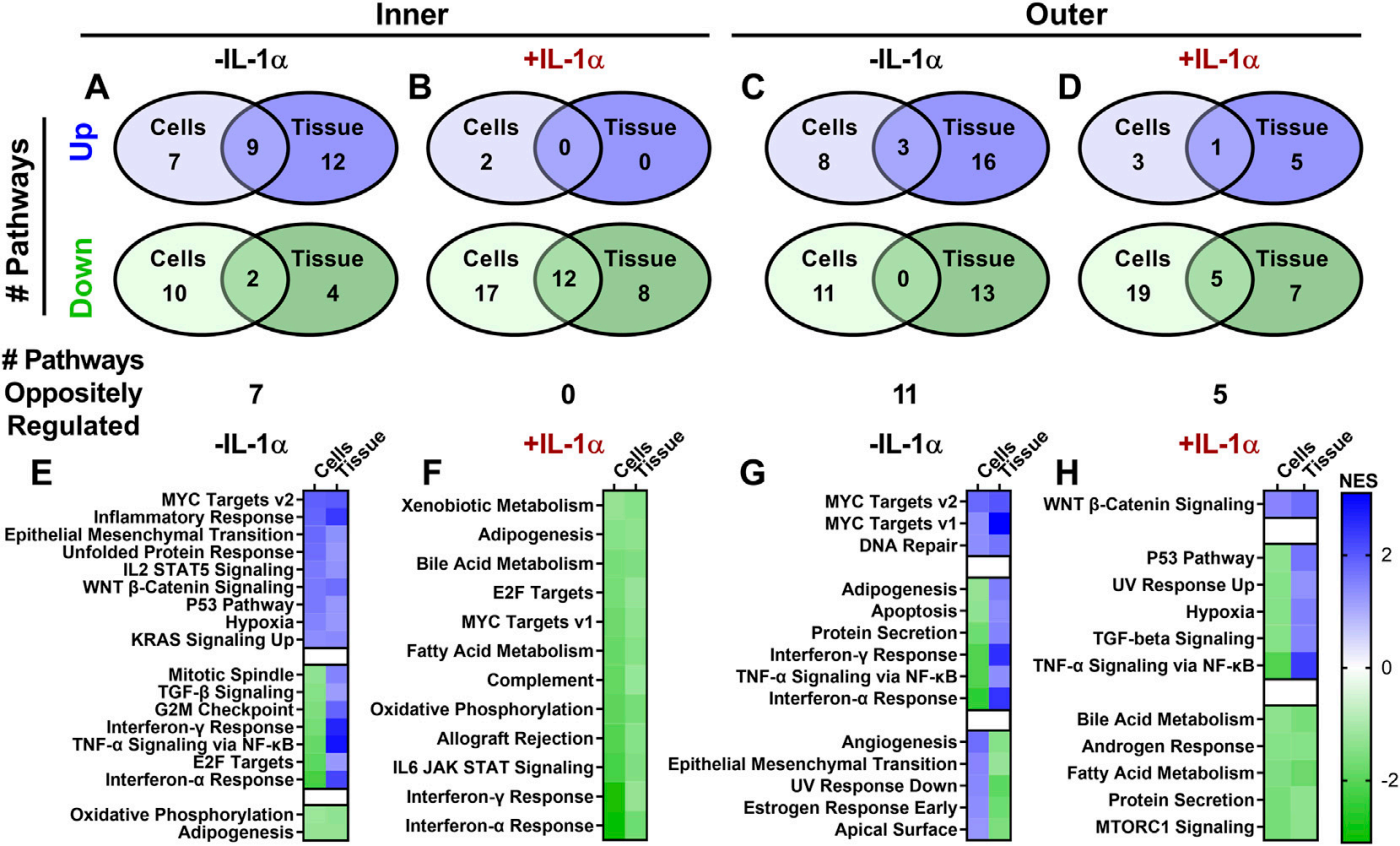
Pathways altered by dynamic loading are more similar in inner zone than outer zone for cell stretch and tissue compression. Gene Set Enrichment Analysis (GSEA) revealed pathways up- (blue) or downregulated (green) with dynamic loading in the presence or absence of IL-1α. The number of pathways regulated by loading were compared between cell stretch and tissue compression for inner (**A**: −IL-1α, **B**: +IL-1α) and outer (**C**: −IL-1α, **D**: +IL-1α) zones. Normalized enrichment scores (NES) of pathways significantly regulated by loading in both models were compared for inner (**E**: −IL-1α, **F**: + IL-1α) and outer (**G**: −IL-1α, **H**: +IL-1α) zones.

GSEA similarly revealed interesting differences in hallmark pathways differentially regulated by dynamic loading between zones and with or without IL-1. For the inner zone, 9 pathways were upregulated and 2 pathways were downregulated in common between the two loading modalities, while 7 pathways showed opposite regulation and were upregulated by tissue compression but downregulated by cell stretch (Figures 6A,E). However, in the presence of IL-1, there was strong agreement between the two models: 12 pathways were downregulated by loading in both models, and there were no pathways displaying opposite regulation (Figures 6B,F). Outer zone results were much less congruent between models, with only 3 pathways showing similar regulation and 11 pathways showing opposite regulation in response to loading alone (Figures 6C,G). Upon loading in the presence of IL-1, there were 6 pathways showing similar regulation and 5 pathways showing opposite regulation in the outer zone (Figures 6D,H). Notably, only a few pathways were significantly differentially regulated by both dynamic compression and cell stretch across both inner and outer zones. MYC Targets v2 was upregulated by both tissue compression and cell stretch across zones. Interferon-γ Response, TNF-α Signaling *via* NF-κB, and Interferon-α Response pathways were all downregulated by cell stretch but upregulated by tissue compression for both inner and outer zones (Figures 6E,G). In the presence of IL-1, Bile Acid Metabolism and Fatty Acid Metabolism pathways were downregulated by loading across models and zones (Figures 6F,H). However, no other GSEA pathway responses were conserved between inner and outer zones across both models of loading.

**FIGURE 7:**
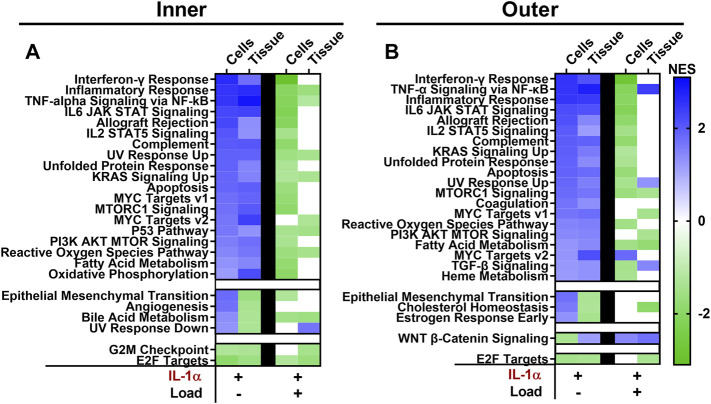
The majority of pathways upregulated by IL-1α are downregulated by dynamic cell stretch in the presence of IL-1α. Pathways significantly differentially regulated with IL-1α stimulation are compared to significantly differentially regulated pathways with dynamic loading in the presence of IL-1α for inner **(A)** and outer **(B)** zones. Cell stretch and tissue compression normalized enrichment scores (NES) are shown in each column. A NES > 0 identifies upregulated (blue) pathways and a NES < 0 identifies downregulated (green) pathways. White boxes denote that the geneset did not meet the cutoff for significance (FDR < 0.25).

Further evidence of the inflammation modulating effects of mechanical loading is seen by the effect of dynamic load on genesets significantly differentially regulated by IL-1 treatment. A total of 18 of the 19 genesets upregulated by IL-1 treatment of inner zone cells were downregulated by cell stretch in the presence of IL-1, and 7 of these pathways are downregulated by tissue compression (Figure 7A). Stretched cells from the outer zone display similar results, with 16 out of 20 pathways upregulated by IL-1 treatment showing downregulation by cell stretch in the presence of IL-1 (Figure 7B). However, only four of these pathways were downregulated by compression of outer zone tissue in the presence of IL-1, while three were further upregulated by tissue compression in the presence of IL-1 (Figure 7B).

**FIGURE 8:**
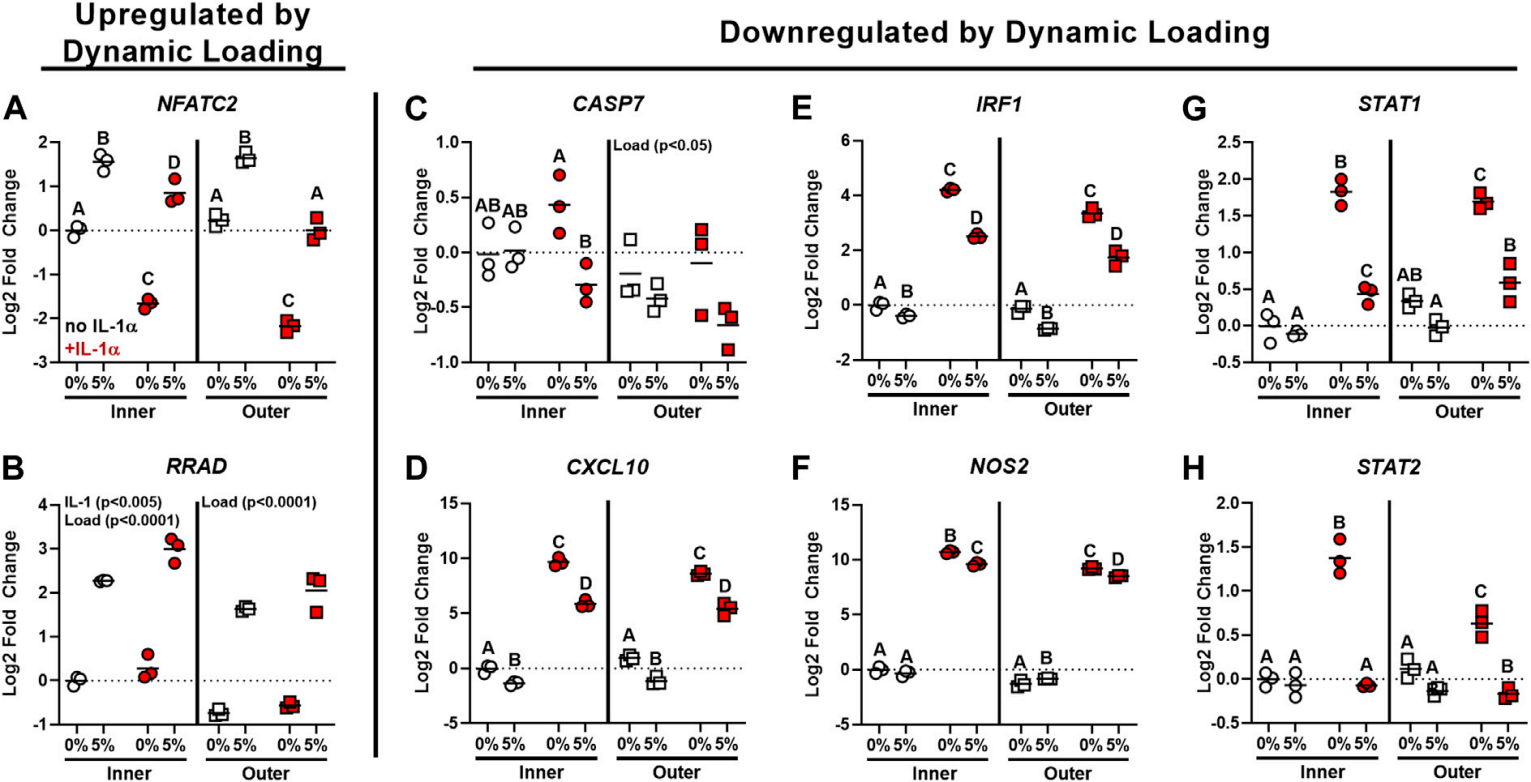
RT-qPCR validated gene targets identified by RNA-sequencing for dynamic cell stretch. Gene targets upregulated by dynamic loading (**A**: *NFATC2*, **B**: *RRAD*) and downregulated by dynamic loading (**C**: *CASP7*, **D**: *CXCL10*, **E**: *IRF1*, **F**: *NOS2*, **G**: *STAT1*, **H**: *STAT2*) identified by RNA-sequencing were validated using RT-qPCR. Shape denotes zone (circle: inner, square: outer) and color identifies IL-1α stimulation (white: no IL-1α, red: + IL-1α). Results are presented as fold-change relative to the inner zone unloaded control group (indicated by the dashed line). Groups not sharing a letter are significantly different (*p* < 0.05). A significant main effect by load or IL-1 with no significant interaction between load and IL-1 is indicated by the factor name on the individual graph.

Individual genes identified by RNA-seq as significantly differentially regulated by dynamic cell stretch in the presence of IL-1 and with known functions related to inflammation and PTOA development were chosen for further validation by RT-qPCR, which confirmed the patterns of gene expression seen in the RNA-seq data. *NFATC2* expression was downregulated by IL-1 treatment (Figure 8A, *p* < 0.0001), and was significantly upregulated by loading both in the presence and absence of IL-1 (*p* < 0.005). *RRAD* expression was also significantly upregulated by load for both inner and outer zone (Figure 8B, *p* < 0.0001) and expression was upregulated by IL-1 in inner zone cells (*p* < 0.005). There was a significant effect of load in both the inner and outer zone cells on *CASP7* expression (Figure 8C, *p* < 0.05) and expression was significantly downregulated by load in the presence of IL-1 for inner zone cells (*p* < 0.05). Expression of *CXCL10* was increased with IL-1 treatment (Figure 8D, *p* < 0.0001) and decreased both by loading in the absence of IL-1 (*p* < 0.01) and by loading of IL-1 treated inner and outer zone cells (*p* < 0.0001). There was a main effect of load (*p* < 0.0001) causing downregulation of *IRF1* (Figure 8E), *NOS2* (Figure 8F, inner zone cells only), *STAT1* (Figure 8G), and *STAT2* (Figure 8H). In both zones for each of these genes, IL-1 caused an upregulation of expression (*p* < 0.001) and there was an interactive effect of load and IL-1, which resulted in downregulation of gene expression compared to IL-1 alone (*p* < 0.05).

## 4 Discussion

Together the findings from this study support the idea that diverse types of dynamic load can modulate the IL-1 response of meniscus cells. Dynamic tissue compression of meniscus tissue explants and dynamic tensile stretch of monolayer meniscus cells showed considerable concordance in RNA-sequencing results, although the degree of overlap varied based on the anatomic region and presence of an inflammatory stimulus. The highest concordance between loading models was seen in the inner zone IL-1 treated condition, where all the hallmark pathways identified by GSEA as significantly different by loading in the presence of IL-1 for both models were downregulated and none showed opposite regulation. Overall, our findings reveal that dynamic mechanical loading mitigates the global inflammatory response of meniscus cells at the transcriptomic level.

In general, mechanical stimulation alone caused relatively few gene targets to be either up or downregulated for both loading models. The exception to this was outer zone tissue compression, which resulted in downregulation of numerous gene transcripts. We did not observe significant up-regulation of the anabolic genes *COL2A1*, *COL1A1*, or *ACAN* with dynamic loading in either model. Prior reports of the effect of dynamic loading on anabolic gene expression are mixed (Upton et al., 2003; Furumatsu et al., 2012), and the use of skeletally mature porcine tissue in this study may mean that these cells have limited anabolic capability due to age. Mechanical stimulation in the presence of IL-1 resulted in far more significantly differentially regulated genes than loading alone, and generally resulted in downregulation of many genes and upregulation of relatively few transcripts. Consistent with previous studies (Ferretti et al., 2006), *NOS2* expression was increased by IL-1 treatment and decreased by dynamic tensile stretch in the presence of IL-1 for both inner and outer zone cells, and the same effect was observed with dynamic compression of inner zone tissue. In addition to identifying individual genes of interest for meniscus research, this study reveals the global transcriptomic and pathway changes in both inner and outer zone meniscus cells in response to dynamic loading both in the presence and absence of the inflammatory stimulus IL-1.

Many of the hallmark pathways upregulated by IL-1 and downregulated by dynamic loading in the presence of IL-1 are inflammatory signaling pathways known to contribute to the development of OA, such as TNF-α signaling *via* NF-κB (Gao et al., 2020; Sueishi et al., 2020), interferon-γ (Otero et al., 2007), IL-6 JAK STAT signaling (Akeson and Malemud, 2017), and complement (Silawal et al., 2018). Genesets related to p53 and apoptosis, which are also known to play a role in the development of OA (Hwang and Kim, 2015; Xu et al., 2020), were also upregulated by IL-1 treatment and downregulated by dynamic loading in the presence of IL-1. Similarly, MTORC1 (Yang et al., 2020) and PI3K AKT MTOR (Sun et al., 2020) signaling pathways were also upregulated by IL-1 and downregulated by dynamic load. Interestingly, PI3K AKT MTOR signaling may play an important role in many processes known to be dysregulated in OA development, including apoptosis, proliferation, and metabolism (Zheng et al., 2021). Indeed, pathways related to proliferation and cell cycle regulation (KRAS signaling and MYC targets) were also identified by GSEA as being differentially regulated by dynamic loading in the presence of IL-1. Metabolic pathways, such as oxidative phosphorylation and fatty acid metabolism, were also upregulated by IL-1 and downregulated by dynamic load in the presence of IL-1. Due to the complex and often overlapping nature of these signaling pathways, determining the functional effects of dynamic mechanical stimulation will require considerable future work. However, these results demonstrate that dynamic loading has significant effects on diverse cellular functions related to tissue healing and OA development and dynamic loading seems to oppose the effects of IL-1. These findings underscore the potential utility of developing therapies targeted to mechanotransduction pathways, which may have widespread effects not limited to a single aspect of OA development, such as inflammation or metabolism, but may be able to combat this multifactorial disease by modulating multiple critical pathways.

The function of many individual genes identified by RNA-seq and validated by RT-qPCR point to the exciting potential effects of the interaction between dynamic load and inflammation in meniscus cells. Targets that were upregulated by dynamic loading may have protective roles in meniscus cells. Expression of *NFATC2*, which is a calcium-responsive transcription factor, was downregulated by IL-1 treatment and upregulated by dynamic load for both inner and outer zone cells. NFATC2 has been shown to be protective against OA development, and knockdown of NFATC2 causes OA in a mouse model (Wang et al., 2009; Rodova et al., 2011; Greenblatt et al., 2013). *RRAD*, which was upregulated by dynamic loading, is protective against cellular senescence (Wei et al., 2019), and is an inhibitor of the NF-κB pathway (Hsiao et al., 2014). In addition to these upregulated targets, there were many genes in the RNA-seq dataset that were downregulated by dynamic loading in the presence of IL-1. *CASP7*, which is called the “executioner protein” for its role in apoptosis and is an outcome measure in many studies of potential OA therapeutics (Murakami et al., 2019), was downregulated by dynamic load in the presence of IL-1 for inner zone cells. The other 5 targets validated by qPCR are significantly upregulated by IL-1 treatment and downregulated by dynamic loading in the presence of IL-1. CXCL10 is a chemokine that stimulates monocytes and T cells and has elevated expression in the synovium and cartilage following articular fracture (Furman et al., 2018) and is evaluated in studies treating OA and joint inflammation (Wang et al., 2015). IRF1 is a transcription factor with roles related to inflammatory response, proliferation, and apoptosis, and has been linked to increased expression of MMP-3 and MMP-13 (Lu et al., 2014), which are mediators of meniscus tissue degeneration and prevent meniscus tissue repair *in vitro* (McNulty et al., 2009). *NOS2* encodes inducible nitric oxide synthase (iNOS), which is a key indicator of inflammatory response in meniscus cells and chondrocytes and has been linked to ECM degradation (LeGrand et al., 2001; Shin et al., 2003; Yang et al., 2010). Transcription factors STAT1 and STAT2 are activated by interferons and are common targets in studies of potential therapeutics for OA and rheumatoid arthritis (Li et al., 2001; Legendre et al., 2003; Millward-Sadler et al., 2006; Dai et al., 2018). Future work is needed to explore the significance of these gene expression changes on functional outcomes, such as cell survival, anabolic/catabolic balance, and cellular senescence, but these data clearly show the power of dynamic mechanical stimulation to mitigate IL-1-induced changes in expression of genes relevant to meniscus homeostasis and OA development.

RT-qPCR validation was performed only on samples from the cell stretch model, as this model showed much greater consistency of expression changes at the single gene level. This is evidenced by the order of magnitude difference in *p*-values observed between models of loading in the volcano plots (Figure 4) and clustering of samples in the PCA plots (Figure 2). There are a number of possible sources for the heterogeneity observed in the tissue compression dataset, including heterogeneity of tissue samples both between menisci (inter-individual) and between samples obtained from the same meniscus (intra-individual), as the variability of cellular phenotypes and ECM composition throughout the meniscus varies not only based on inner and outer regions, but also between anterior, posterior, and midbody regions (Mauck et al., 2007; Di Giancamillo et al., 2014). The cell stretch model allowed for greater control of donor and site variability as isolated cells were pooled from the entire inner or outer region of multiple menisci, reducing both inter- and intra-individual variability. Another potential contributing factor to differences between the two datasets is the RNA extraction procedure. RNA extraction from meniscus tissue required pulverization in a freezer mill for homogenization, followed by phenol/chloroform extraction, whereas high quality RNA from monolayer cells was easily isolated by column purification. High quality RNA (RIN > 6.5) was obtained for all meniscus tissue and monolayer samples used in this study. Any differences in RNA preparation should not affect the differential expression results presented here, as each sample type was sequenced and analyzed separately. However, it could be a contributing factor to the differences in variability observed between the loading models. Overall, both models are useful for studies of mechanotransduction and modulation of inflammatory response by mechanical stimulation based on the conservation of global transcriptomic trends and GSEA pathways; however, the cell stretch model yielded superior consistency and repeatability for individual gene expression analyses and is more amenable to isolation of high-quality RNA for transcriptomic studies.

The tissue compression model was designed to mimic physiologic loading as closely as possible, by using a strain level thought to correspond to physiologic *in vivo* loads (Upton et al., 2006b; Freutel et al., 2014) and a frequency of 1Hz, which corresponds to a brisk walking pace (Tudor-Locke and Rowe, 2012). Explants were subjected to three loading bouts of 4 h each on three consecutive days, as this has been shown to be sufficient time to detect mechanical loading effects on IL-1 induced tissue degeneration (McNulty et al., 2010). However, It is not entirely clear how macro-scale tissue strains are translated to cellular deformation, so the cell stretch parameters were chosen based on empirically-derived parameters found in the literature (Ferretti et al., 2006; Kanazawa et al., 2012). A 5% equibiaxial strain at 0.5Hz has pro-anabolic and anti-inflammatory effects on meniscal cells (Ferretti et al., 2006; Kanazawa et al., 2012), and 4 h of loading causes sustained anti-inflammatory effects (Ferretti et al., 2006). Therefore, a single loading bout was used for monolayer cells due to ongoing cellular proliferation in monolayer culture, which would have resulted in different cell densities at the time of loading if performed on multiple consecutive days. Furthermore, prior work using dynamically compressed agarose-embedded chondrocytes revealed that there was an initial response in gene expression that decays over time, and when repeated loading was performed each day for 3 days, as was done in the tissue explant model, the results were the same as the single loading bout (Nims et al., 2021). Despite the differences in loading protocols, the amount of overlap in observed responses is remarkable, suggesting that there are conserved mechanisms of mechanotransduction activated by both cell stretch and tissue compression, and that both models of mechanical stimulation are useful for elucidating mechanisms by which meniscus cells sense and respond to their mechanical environment. However, the differences in loading protocols do limit the interpretation of our results and raise many additional interesting questions regarding the nature of mechanotransduction in meniscus cells.

Based on finite element modeling, 10% compression may represent physiologic loading for inner zone tissue but due to meniscus extrusion towards the periphery of the joint, outer zone tissue likely does not experience macroscale tissue compressive strains as high as 10% during normal physiologic loading (Upton et al., 2006b; Freutel et al., 2014). On the other hand, 5% cell stretch may be close to the magnitude of deformation experienced at the cellular level for both inner and outer zone cells (Upton et al., 2006b). Interestingly, it appears that the two loading regimes investigated in this study were comparable for the inner zone despite differences in loading parameters and cellular environment. It remains to be determined whether the differing response of outer zone cells to 10% tissue compression and 5% stretch is due to loading or environmental differences, such as differences in magnitude, frequency, type of load, duration of loading, or differences in the ECM and cell microenvironment. Further work is needed to elucidate the mechanisms of mechanosensation in meniscus cells, which could have implications for therapeutic targeting of mechanotransduction pathways to stimulate meniscus injury repair.

Little overlap was observed in GSEA pathways modulated by both models of loading between inner and outer zones. Only MYC Targets v2 was upregulated by both models across inner and outer zones. Interestingly, Interferon-γ Response, TNF-α Signaling *via* NF-κB, and Interferon-α Response pathways all showed the same pattern of expression, being upregulated by dynamic compression but downregulated by cell stretch in both inner and outer zone cells. In addition, very little agreement was observed between inner and outer zone response to loading in the presence of IL-1. Only the metabolic pathways Bile Acid Metabolism and Fatty Acid Metabolism are significantly regulated by both models of dynamic loading with IL-1 in both inner and outer zones. Inflammation related pathways IL6 JAK STAT Signaling and Interferon-γ Response were downregulated by both models of dynamic loading for inner zone samples in the presence of IL-1, and both of these pathways are downregulated by cell stretch of outer zone cells in the presence of IL-1, but not tissue compression. TNF-α Signaling via NF-κB, which is universally upregulated by IL-1 treatment alone, was also downregulated by both loading modalities for inner zone and by cell stretch of outer zone cells in the presence of IL-1, but upregulated by compression of outer zone tissue in the presence of IL-1. Generally, it appears that the anti-inflammatory effects of dynamic loading are well-conserved between inner and outer zone stretched monolayer cells and inner zone compressed tissue, but not outer zone compressed tissue. One explanation for this is that there may be ECM-dependent differences in mechanotransduction between zones, which could have important implications for targeting mechanotransduction pathways to stimulate injury healing. While it is beyond the scope of the analyses presented here to fully characterize the differences between inner and outer zone mechanoresponsiveness, interesting differences are apparent and warrant further investigation.

Overall, results from both models showed significant modulation of inflammation-related pathways with mechanical stimulation, supporting the potential of targeting mechanotransduction pathways as novel therapeutic targets to improve outcomes following meniscus injury. Anti-inflammatory effects of loading were well-conserved between the tissue compression and cell stretch models for inner zone, but the cell stretch model provided greater statistical power due to improved consistency between replicates and resulted in a larger number of significantly differentially regulated genes. Our findings on the global transcriptomic profiles of two models of mechanical stimulation lay the groundwork for future mechanistic studies of meniscus mechanotransduction, which may lead to the discovery of novel therapeutic targets for the treatment of meniscus injuries and the prevention of PTOA development.

## Data Availability

The datasets presented in this study can be found in the NCBI Gene Expression Omnibus (GEO) under accession numbers GSE191175 (tissue compression) and GSE191321 (cell stretch). https://www.ncbi.nlm.nih.gov/geo/, GSE191175; GSE191321.
