# Supplementary material for: A Tale of Two Loads: Modulation of IL-1 Induced Inflammatory Responses of Meniscal Cells in Two Models of Dynamic Physiologic Loading
Source: Front Bioeng Biotechnol. 2022 Mar 1;10:837619. doi: 10.3389/fbioe.2022.837619 (PMC8921261; doi:10.3389/fbioe.2022.837619)
Supplement: Supplementary file 1 [file DataSheet4.DOCX]

**Supplemental Table 5**: IL-1α stimulation compared to unstimulated samples for unloaded outer zone tissue.

| **Gene ID** | **Gene Name** | **Log2Fold Change** | **p-value** | **Up/Down Regulated** |
| --- | --- | --- | --- | --- |
| ENSSSCG00000038162 | CCL21 | 26.13642021 | 0.003892109 | UP |
| ENSSSCG00000009202 | MMRN1 | 25.21497727 | 0.005794324 | UP |
| ENSSSCG00000020970 | IL6 | 6.80265076 | 4.17098E-19 | UP |
| ENSSSCG00000015426 | RELN | 5.684205896 | 0.005158313 | UP |
| ENSSSCG00000008957 | AMCF-II | 5.499656198 | 1.05512E-06 | UP |
| ENSSSCG00000008953 | CXCL8 | 5.02823226 | 2.31034E-06 | UP |
| ENSSSCG00000039761 | MYCL | 4.909854521 | 0.002183386 | UP |
| ENSSSCG00000040725 | IL11 | 4.860538742 | 1.75836E-08 | UP |
| ENSSSCG00000008768 | ARAP2 | 4.779198591 | 0.018562782 | UP |
| ENSSSCG00000004195 | ARG1 | 4.706879785 | 2.81068E-14 | UP |
| ENSSSCG00000017488 | CSF3 | 4.588665236 | 0.000208116 | UP |
| ENSSSCG00000003669 | MFSD2A | 4.547581938 | 1.40806E-10 | UP |
| ENSSSCG00000040961 | LIF | 4.410323227 | 1.26881E-14 | UP |
| ENSSSCG00000008959 | CXCL2 | 4.20894494 | 1.49883E-08 | UP |
| ENSSSCG00000015579 | PTGS2 | 4.110506255 | 5.78771E-15 | UP |
| ENSSSCG00000014987 | MMP12 | 4.044212971 | 7.97739E-11 | UP |
| ENSSSCG00000008954 | NA | 3.810202779 | 1.50741E-08 | UP |
| ENSSSCG00000014897 | FAM181B | 3.810016405 | 0.006383659 | UP |
| ENSSSCG00000023627 | LYPD6 | 3.663781102 | 0.003441244 | UP |
| ENSSSCG00000024299 | SCN3B | 3.540979175 | 0.04645024 | UP |
| ENSSSCG00000005203 | IL33 | 3.380981734 | 0.040507745 | UP |
| ENSSSCG00000033952 | CITED4 | 3.322326948 | 0.030659523 | UP |
| ENSSSCG00000010054 | ADORA2A | 3.233549 | 3.48765E-08 | UP |
| ENSSSCG00000003744 | MOCOS | 3.120820406 | 0.001100732 | UP |
| ENSSSCG00000012027 | ADAMTS5 | 3.093660276 | 2.83433E-08 | UP |
| ENSSSCG00000023592 | TAC1 | 2.929051929 | 0.024920508 | UP |
| ENSSSCG00000000291 | GPR84 | 2.879875183 | 0.00287129 | UP |
| ENSSSCG00000012026 | ADAMTS1 | 2.844642327 | 0.009565643 | UP |
| ENSSSCG00000020953 | NA | 2.839197373 | 0.006099778 | UP |
| ENSSSCG00000026587 | BATF3 | 2.837380162 | 0.00190918 | UP |
| ENSSSCG00000006590 | S100A8 | 2.764694016 | 0.043134909 | UP |
| ENSSSCG00000038185 | EREG | 2.745137355 | 0.000323096 | UP |
| ENSSSCG00000016254 | CCL20 | 2.741767958 | 1.13248E-06 | UP |
| ENSSSCG00000032857 | S100A12 | 2.739548912 | 0.020177386 | UP |
| ENSSSCG00000031898 | CALHM5 | 2.623530299 | 0.045354288 | UP |
| ENSSSCG00000006391 | ATP1A2 | 2.565033135 | 0.012587103 | UP |
| ENSSSCG00000038727 | GDNF | 2.558271485 | 0.045096525 | UP |
| ENSSSCG00000008090 | IL1A | 2.509643048 | 0.004286274 | UP |
| ENSSSCG00000013551 | C3 | 2.499687127 | 0.000158417 | UP |
| ENSSSCG00000006588 | S100A9 | 2.438368785 | 0.038198539 | UP |
| ENSSSCG00000038929 | CEMIP | 2.42771333 | 0.000254921 | UP |
| ENSSSCG00000016497 | DENND2A | 2.423408396 | 4.49475E-05 | UP |
| ENSSSCG00000009060 | NA | 2.363141969 | 0.004444869 | UP |
| ENSSSCG00000009558 | F10 | 2.350462989 | 0.044215445 | UP |
| ENSSSCG00000024914 | NA | 2.285607359 | 0.000142756 | UP |
| ENSSSCG00000014985 | MMP3 | 2.218346851 | 1.11867E-05 | UP |
| ENSSSCG00000023837 | WFDC1 | 2.208411554 | 0.021378707 | UP |
| ENSSSCG00000016519 | AKR1D1 | 2.177330729 | 0.002801415 | UP |
| ENSSSCG00000022290 | UMODL1 | 2.172597348 | 0.011520027 | UP |
| ENSSSCG00000035456 | WWC1 | 2.167472606 | 0.007424637 | UP |
| ENSSSCG00000035037 | NA | 2.092158309 | 7.24832E-06 | UP |
| ENSSSCG00000028331 | IL1R2 | 2.092013652 | 0.011498221 | UP |
| ENSSSCG00000009002 | TLR2 | 2.074378327 | 4.9183E-07 | UP |
| ENSSSCG00000035392 | IGFBP2 | 2.067264796 | 0.029811571 | UP |
| ENSSSCG00000022446 | SEL1L3 | 2.064077496 | 5.27673E-05 | UP |
| ENSSSCG00000010219 | ARID5B | 2.034284417 | 1.05359E-06 | UP |
| ENSSSCG00000004670 | C15orf48 | 2.026227769 | 0.01625778 | UP |
| ENSSSCG00000021557 | SULT1A3 | 2.014446537 | 0.048057775 | UP |
| ENSSSCG00000036746 | RASL10B | 1.993859805 | 0.001988002 | UP |
| ENSSSCG00000014921 | PRSS23 | 1.992956505 | 0.006416902 | UP |
| ENSSSCG00000027030 | BDKRB2 | 1.991086311 | 0.028875436 | UP |
| ENSSSCG00000037642 | ARID3A | 1.969321974 | 2.35602E-08 | UP |
| ENSSSCG00000016230 | EPHA4 | 1.919388106 | 0.015341288 | UP |
| ENSSSCG00000015595 | ATF3 | 1.881148389 | 1.66556E-05 | UP |
| ENSSSCG00000007465 | B4GALT5 | 1.88103093 | 3.34263E-05 | UP |
| ENSSSCG00000013418 | CFD | 1.871116122 | 0.002269929 | UP |
| ENSSSCG00000012074 | NA | 1.866498244 | 0.000958345 | UP |
| ENSSSCG00000023520 | PCSK5 | 1.865189796 | 2.20839E-05 | UP |
| ENSSSCG00000015616 | HSD11B1 | 1.854550345 | 0.003604348 | UP |
| ENSSSCG00000013425 | MISP | 1.836752356 | 0.004145096 | UP |
| ENSSSCG00000033355 | LGI3 | 1.835078486 | 0.003975686 | UP |
| ENSSSCG00000017605 | MMD | 1.785222402 | 0.015063303 | UP |
| ENSSSCG00000038646 | NA | 1.785034958 | 2.80032E-05 | UP |
| ENSSSCG00000010850 | NA | 1.775682959 | 0.000349055 | UP |
| ENSSSCG00000023351 | PLA2G4A | 1.748949245 | 6.64555E-10 | UP |
| ENSSSCG00000015303 | CFAP69 | 1.74734655 | 0.007998179 | UP |
| ENSSSCG00000015302 | STEAP2 | 1.728855882 | 0.016984944 | UP |
| ENSSSCG00000016997 | FGF18 | 1.721914326 | 0.004485704 | UP |
| ENSSSCG00000022961 | CLMP | 1.709365586 | 0.004827372 | UP |
| ENSSSCG00000026454 | NA | 1.67953105 | 0.007582568 | UP |
| ENSSSCG00000008902 | PPAT | 1.673651863 | 0.012811655 | UP |
| ENSSSCG00000037803 | MARCKS | 1.67327006 | 0.049366192 | UP |
| ENSSSCG00000001782 | ABHD17C | 1.658576293 | 4.29548E-05 | UP |
| ENSSSCG00000038285 | KLHL36 | 1.653379636 | 1.18438E-05 | UP |
| ENSSSCG00000037815 | ZC3H12A | 1.646137951 | 2.06113E-05 | UP |
| ENSSSCG00000040207 | P2RY2 | 1.604728706 | 0.043946403 | UP |
| ENSSSCG00000026499 | NMT2 | 1.597749867 | 0.020059929 | UP |
| ENSSSCG00000032715 | CERS6 | 1.593224336 | 0.029232847 | UP |
| ENSSSCG00000032221 | FAM110C | 1.58554289 | 0.02869801 | UP |
| ENSSSCG00000037645 | COTL1 | 1.517063916 | 0.014668131 | UP |
| ENSSSCG00000022401 | AGTRAP | 1.515235178 | 1.75836E-08 | UP |
| ENSSSCG00000004125 | STX11 | 1.511648781 | 0.006944308 | UP |
| ENSSSCG00000003805 | PDE4B | 1.500687415 | 3.10784E-08 | UP |
| ENSSSCG00000000456 | SLC16A7 | 1.498373061 | 0.001505275 | UP |
| ENSSSCG00000012583 | ACSL4 | 1.496968155 | 6.28924E-07 | UP |
| ENSSSCG00000006187 | MSC | 1.494362747 | 0.017645321 | UP |
| ENSSSCG00000024793 | PORCN | 1.487260611 | 4.88542E-05 | UP |
| ENSSSCG00000013599 | ANGPTL4 | 1.431964163 | 0.010321168 | UP |
| ENSSSCG00000023716 | TNFAIP6 | 1.428984622 | 0.007582984 | UP |
| ENSSSCG00000040317 | SOD2 | 1.386261016 | 0.001313799 | UP |
| ENSSSCG00000012915 | CLCF1 | 1.376745591 | 4.86437E-06 | UP |
| ENSSSCG00000031970 | RASSF5 | 1.374423648 | 0.00031263 | UP |
| ENSSSCG00000038500 | TRIB1 | 1.373812625 | 6.12369E-06 | UP |
| ENSSSCG00000025270 | CHRD | 1.367568488 | 0.011453653 | UP |
| ENSSSCG00000022780 | UGCG | 1.36480158 | 3.50909E-05 | UP |
| ENSSSCG00000015872 | GPD2 | 1.352488819 | 4.63486E-05 | UP |
| ENSSSCG00000011643 | AMOTL2 | 1.338942663 | 2.18402E-06 | UP |
| ENSSSCG00000012880 | CPT1A | 1.337199195 | 0.001389542 | UP |
| ENSSSCG00000027529 | BIRC3 | 1.332021326 | 3.43085E-05 | UP |
| ENSSSCG00000038073 | NOD2 | 1.32759729 | 0.006848863 | UP |
| ENSSSCG00000012202 | NA | 1.31309137 | 0.01267331 | UP |
| ENSSSCG00000014840 | P4HA3 | 1.308183082 | 0.049366192 | UP |
| ENSSSCG00000008168 | RNF149 | 1.302542524 | 1.28717E-05 | UP |
| ENSSSCG00000026602 | PTGIR | 1.302185692 | 0.019517806 | UP |
| ENSSSCG00000005688 | PTGES | 1.292016804 | 0.002667467 | UP |
| ENSSSCG00000033786 | NA | 1.288197509 | 0.004286274 | UP |
| ENSSSCG00000024674 | ABL2 | 1.281507554 | 0.000649808 | UP |
| ENSSSCG00000009434 | RGCC | 1.279846757 | 0.00097947 | UP |
| ENSSSCG00000007874 | NA | 1.279423915 | 0.003709055 | UP |
| ENSSSCG00000040445 | RND3 | 1.265331898 | 0.021433055 | UP |
| ENSSSCG00000038879 | RELB | 1.257205495 | 3.00844E-07 | UP |
| ENSSSCG00000008443 | EPAS1 | 1.252910774 | 0.000340276 | UP |
| ENSSSCG00000015770 | VEGFC | 1.25009414 | 0.035993226 | UP |
| ENSSSCG00000001952 | NFKBIA | 1.241405982 | 6.12448E-05 | UP |
| ENSSSCG00000000951 | NA | 1.219940062 | 0.000673822 | UP |
| ENSSSCG00000029002 | PNKD | 1.213608366 | 9.14591E-06 | UP |
| ENSSSCG00000032367 | CEBPD | 1.207021134 | 1.95697E-05 | UP |
| ENSSSCG00000011218 | SLC4A7 | 1.206983644 | 0.000106082 | UP |
| ENSSSCG00000028536 | LHFPL2 | 1.203369275 | 2.88163E-05 | UP |
| ENSSSCG00000038842 | PCDH9 | 1.202261786 | 0.015471862 | UP |
| ENSSSCG00000010261 | PPA1 | 1.197297009 | 0.000108799 | UP |
| ENSSSCG00000007140 | SMOX | 1.194977016 | 0.000684833 | UP |
| ENSSSCG00000011828 | FAM43A | 1.188305228 | 0.004337836 | UP |
| ENSSSCG00000004241 | GJA1 | 1.173212955 | 0.016025802 | UP |
| ENSSSCG00000016101 | CFLAR | 1.171457994 | 8.82967E-11 | UP |
| ENSSSCG00000026962 | A4GALT | 1.166926515 | 0.013719319 | UP |
| ENSSSCG00000013735 | JUNB | 1.165695704 | 2.29748E-07 | UP |
| ENSSSCG00000015322 | TFPI2 | 1.159699008 | 0.015536069 | UP |
| ENSSSCG00000030300 | MT2A | 1.159278993 | 0.024157752 | UP |
| ENSSSCG00000006718 | ZNF697 | 1.153807415 | 0.010912502 | UP |
| ENSSSCG00000030283 | NA | 1.143469397 | 0.026383712 | UP |
| ENSSSCG00000004658 | FBN1 | 1.140140296 | 0.023334886 | UP |
| ENSSSCG00000000475 | IRAK3 | 1.136278512 | 0.003892109 | UP |
| ENSSSCG00000013360 | TMEM86A | 1.126041185 | 0.004524807 | UP |
| ENSSSCG00000010006 | NA | 1.123450189 | 0.01143593 | UP |
| ENSSSCG00000028322 | BTG2 | 1.119724633 | 0.002326651 | UP |
| ENSSSCG00000032434 | PLAUR | 1.1128007 | 0.006999595 | UP |
| ENSSSCG00000010448 | FAS | 1.109063799 | 0.040759337 | UP |
| ENSSSCG00000015784 | ACSL1 | 1.108327655 | 0.003441244 | UP |
| ENSSSCG00000032517 | DMXL2 | 1.095936581 | 0.038764143 | UP |
| ENSSSCG00000034207 | CEBPB | 1.083005958 | 0.001140609 | UP |
| ENSSSCG00000004154 | TNFAIP3 | 1.082840682 | 0.013677462 | UP |
| ENSSSCG00000012743 | MTMR1 | 1.082377299 | 1.04385E-07 | UP |
| ENSSSCG00000010540 | ENTPD7 | 1.074222147 | 2.82048E-05 | UP |
| ENSSSCG00000010036 | SLC5A1 | 1.065992344 | 0.015957516 | UP |
| ENSSSCG00000020705 | MAP3K8 | 1.064731013 | 0.003861296 | UP |
| ENSSSCG00000008383 | NA | 1.05254564 | 0.019058565 | UP |
| ENSSSCG00000038594 | SDC4 | 1.047360791 | 0.003087285 | UP |
| ENSSSCG00000008374 | B3GNT2 | 1.045657093 | 0.001395281 | UP |
| ENSSSCG00000039472 | SLC30A1 | 1.041008875 | 0.000452944 | UP |
| ENSSSCG00000025821 | C2CD2 | 1.037359355 | 0.002748585 | UP |
| ENSSSCG00000002311 | SUSD6 | 1.033226844 | 0.025272192 | UP |
| ENSSSCG00000004149 | NHSL1 | 1.02515992 | 0.020096847 | UP |
| ENSSSCG00000002385 | TGFB3 | 1.022626523 | 0.004733008 | UP |
| ENSSSCG00000032622 | PPP3CC | 1.015936173 | 0.000251177 | UP |
| ENSSSCG00000015014 | ZC3H12C | 1.015924904 | 0.001923999 | UP |
| ENSSSCG00000003169 | PIH1D1 | 1.015696833 | 0.030972126 | UP |
| ENSSSCG00000039867 | UCK2 | 1.007991722 | 6.36214E-06 | UP |
| ENSSSCG00000009620 | BMP1 | 1.007602701 | 0.00402107 | UP |
| ENSSSCG00000029420 | NA | -1.002603281 | 0.029129937 | DOWN |
| ENSSSCG00000022230 | CD9 | -1.009112387 | 0.008134451 | DOWN |
| ENSSSCG00000010325 | KCNMA1 | -1.011309068 | 0.005866367 | DOWN |
| ENSSSCG00000014139 | ARSK | -1.012087389 | 0.003768823 | DOWN |
| ENSSSCG00000025856 | TMEM106A | -1.019037413 | 0.049596344 | DOWN |
| ENSSSCG00000006237 | SDCBP | -1.021147453 | 0.014064807 | DOWN |
| ENSSSCG00000016338 | PER2 | -1.022823912 | 0.002756754 | DOWN |
| ENSSSCG00000011740 | SERPINI1 | -1.024615031 | 0.029426849 | DOWN |
| ENSSSCG00000011689 | PLOD2 | -1.03216457 | 0.041881071 | DOWN |
| ENSSSCG00000037450 | SSC5D | -1.033441722 | 0.045445094 | DOWN |
| ENSSSCG00000032632 | CMTM4 | -1.036948054 | 0.011453653 | DOWN |
| ENSSSCG00000006664 | MTMR11 | -1.042086112 | 0.006575709 | DOWN |
| ENSSSCG00000031940 | GAS2 | -1.046122768 | 0.045096525 | DOWN |
| ENSSSCG00000037016 | ID1 | -1.047178413 | 0.001560306 | DOWN |
| ENSSSCG00000031764 | NA | -1.053388853 | 0.030399086 | DOWN |
| ENSSSCG00000013282 | ACCS | -1.056875692 | 0.001890265 | DOWN |
| ENSSSCG00000040890 | ABHD14B | -1.062002403 | 1.81817E-05 | DOWN |
| ENSSSCG00000035863 | PLIN2 | -1.068710974 | 0.001133653 | DOWN |
| ENSSSCG00000036396 | NEAT1_2 | -1.072797964 | 0.019682548 | DOWN |
| ENSSSCG00000032942 | NA | -1.072848937 | 0.005914391 | DOWN |
| ENSSSCG00000015036 | DIXDC1 | -1.076501064 | 0.011107351 | DOWN |
| ENSSSCG00000026780 | NA | -1.077199507 | 0.030374807 | DOWN |
| ENSSSCG00000036383 | LGALS3BP | -1.078527293 | 0.004356841 | DOWN |
| ENSSSCG00000021208 | SELENOP | -1.08032858 | 0.015297928 | DOWN |
| ENSSSCG00000006861 | EXTL2 | -1.08134683 | 0.00422093 | DOWN |
| ENSSSCG00000006725 | TBX15 | -1.087859124 | 0.000714878 | DOWN |
| ENSSSCG00000010064 | NA | -1.089418418 | 0.022502719 | DOWN |
| ENSSSCG00000003715 | NA | -1.09301427 | 0.00408452 | DOWN |
| ENSSSCG00000017565 | SPATA20 | -1.093560694 | 0.011919348 | DOWN |
| ENSSSCG00000007288 | MYH7B | -1.097405122 | 0.018239853 | DOWN |
| ENSSSCG00000033001 | FZD8 | -1.099309993 | 0.000436521 | DOWN |
| ENSSSCG00000037376 | MXRA7 | -1.100170599 | 0.001560306 | DOWN |
| ENSSSCG00000037406 | TMEM136 | -1.106502067 | 0.020412588 | DOWN |
| ENSSSCG00000016857 | DAB2 | -1.108131779 | 0.000108739 | DOWN |
| ENSSSCG00000037483 | RFTN2 | -1.109342497 | 0.010761557 | DOWN |
| ENSSSCG00000005087 | SIX1 | -1.112298192 | 0.000786239 | DOWN |
| ENSSSCG00000029756 | ADGRG2 | -1.115871664 | 0.015196962 | DOWN |
| ENSSSCG00000027860 | ERAP2 | -1.120271998 | 0.041928258 | DOWN |
| ENSSSCG00000010446 | STAMBPL1 | -1.122011518 | 0.017058126 | DOWN |
| ENSSSCG00000036206 | C3orf58 | -1.123634916 | 6.6672E-07 | DOWN |
| ENSSSCG00000031144 | RAMP3 | -1.124868398 | 0.025247283 | DOWN |
| ENSSSCG00000017759 | ALDOC | -1.125101357 | 0.018276508 | DOWN |
| ENSSSCG00000037898 | CXXC5 | -1.130990058 | 0.00377138 | DOWN |
| ENSSSCG00000028052 | OBSL1 | -1.132775332 | 0.001465508 | DOWN |
| ENSSSCG00000039966 | NXPH4 | -1.133410198 | 0.014668131 | DOWN |
| ENSSSCG00000003491 | AKR7A2 | -1.134505943 | 0.000199379 | DOWN |
| ENSSSCG00000038902 | KCNK6 | -1.143230361 | 0.005917026 | DOWN |
| ENSSSCG00000039587 | NA | -1.148538056 | 0.023334886 | DOWN |
| ENSSSCG00000009268 | CRYL1 | -1.1486268 | 5.30403E-06 | DOWN |
| ENSSSCG00000040272 | TTC29 | -1.15134627 | 0.024907621 | DOWN |
| ENSSSCG00000025106 | NA | -1.153836434 | 0.00031263 | DOWN |
| ENSSSCG00000004902 | RNF152 | -1.154811419 | 0.005351986 | DOWN |
| ENSSSCG00000011704 | WWTR1 | -1.165446037 | 0.00096464 | DOWN |
| ENSSSCG00000028983 | TBC1D1 | -1.166771071 | 0.006878818 | DOWN |
| ENSSSCG00000002847 | GPT2 | -1.167939268 | 0.004216625 | DOWN |
| ENSSSCG00000002353 | FAM161B | -1.168404803 | 0.015673527 | DOWN |
| ENSSSCG00000014232 | LOX | -1.174423441 | 0.026095427 | DOWN |
| ENSSSCG00000001793 | ADAMTSL3 | -1.175716271 | 0.001161791 | DOWN |
| ENSSSCG00000039594 | SSBP3 | -1.176847135 | 0.002183386 | DOWN |
| ENSSSCG00000031334 | NA | -1.179342588 | 0.036212479 | DOWN |
| ENSSSCG00000003201 | ATF5 | -1.183867747 | 0.018322822 | DOWN |
| ENSSSCG00000004192 | CTGF | -1.1845028 | 0.023742594 | DOWN |
| ENSSSCG00000024669 | NA | -1.188831935 | 0.001140609 | DOWN |
| ENSSSCG00000011495 | PRICKLE2 | -1.19264967 | 0.022181834 | DOWN |
| ENSSSCG00000039780 | RTN4RL1 | -1.194626886 | 0.018276508 | DOWN |
| ENSSSCG00000029029 | ZNF713 | -1.197663275 | 0.001548155 | DOWN |
| ENSSSCG00000016618 | CPED1 | -1.198699939 | 0.00340701 | DOWN |
| ENSSSCG00000006729 | FAM46C | -1.200078516 | 0.04780413 | DOWN |
| ENSSSCG00000009123 | CAMK2D | -1.201177191 | 7.10387E-14 | DOWN |
| ENSSSCG00000009668 | CLU | -1.211349051 | 0.019847004 | DOWN |
| ENSSSCG00000003600 | TINAGL1 | -1.215792805 | 0.001613624 | DOWN |
| ENSSSCG00000012182 | PCYT1B | -1.219408527 | 0.048057775 | DOWN |
| ENSSSCG00000040793 | CTSD | -1.224916407 | 0.003387187 | DOWN |
| ENSSSCG00000039045 | SLC26A2 | -1.227463257 | 0.029620522 | DOWN |
| ENSSSCG00000013933 | PBX4 | -1.228634196 | 0.036286407 | DOWN |
| ENSSSCG00000016792 | RETREG1 | -1.232293975 | 0.027341338 | DOWN |
| ENSSSCG00000011765 | USP13 | -1.234132713 | 0.029285391 | DOWN |
| ENSSSCG00000037413 | NA | -1.235172108 | 0.005220118 | DOWN |
| ENSSSCG00000028282 | SLC1A4 | -1.239463753 | 7.98012E-08 | DOWN |
| ENSSSCG00000040985 | KCTD7 | -1.239593014 | 0.008462109 | DOWN |
| ENSSSCG00000033412 | B4GALNT3 | -1.241921644 | 0.044992712 | DOWN |
| ENSSSCG00000009281 | SGCG | -1.24507228 | 0.000139297 | DOWN |
| ENSSSCG00000014908 | CCDC89 | -1.247404824 | 0.023423568 | DOWN |
| ENSSSCG00000022296 | CDK15 | -1.250838199 | 0.012529845 | DOWN |
| ENSSSCG00000015607 | HHAT | -1.255988678 | 0.018946665 | DOWN |
| ENSSSCG00000003022 | TMEM145 | -1.26276651 | 0.002269929 | DOWN |
| ENSSSCG00000017082 | SPARC | -1.267764254 | 0.041015817 | DOWN |
| ENSSSCG00000000455 | LRIG3 | -1.268424165 | 0.013681587 | DOWN |
| ENSSSCG00000039793 | NA | -1.27062601 | 0.000714878 | DOWN |
| ENSSSCG00000015828 | ZNF703 | -1.274412235 | 0.000421096 | DOWN |
| ENSSSCG00000040735 | DDAH1 | -1.275576568 | 0.000108739 | DOWN |
| ENSSSCG00000004390 | SESN1 | -1.277216256 | 0.00030011 | DOWN |
| ENSSSCG00000016085 | NA | -1.278878699 | 0.01267331 | DOWN |
| ENSSSCG00000012519 | GPRASP1 | -1.279803054 | 0.000923562 | DOWN |
| ENSSSCG00000009122 | ARSJ | -1.28934685 | 0.000352816 | DOWN |
| ENSSSCG00000006475 | IQGAP3 | -1.299566965 | 0.031127543 | DOWN |
| ENSSSCG00000001727 | TNFRSF21 | -1.300730893 | 0.008134451 | DOWN |
| ENSSSCG00000004291 | NT5E | -1.302562882 | 0.03617491 | DOWN |
| ENSSSCG00000007477 | NFATC2 | -1.303831869 | 0.007507998 | DOWN |
| ENSSSCG00000032937 | NA | -1.311392182 | 2.00289E-05 | DOWN |
| ENSSSCG00000013403 | GALNT18 | -1.312059472 | 0.036087089 | DOWN |
| ENSSSCG00000009111 | SYNPO2 | -1.312808759 | 0.00149987 | DOWN |
| ENSSSCG00000005481 | NA | -1.313138832 | 7.63476E-06 | DOWN |
| ENSSSCG00000018044 | ALDH3A1 | -1.313256049 | 0.041039736 | DOWN |
| ENSSSCG00000023498 | HSPB6 | -1.313627895 | 2.5906E-07 | DOWN |
| ENSSSCG00000008545 | ZNF512 | -1.329830486 | 7.55303E-08 | DOWN |
| ENSSSCG00000002274 | HSPA2 | -1.330003123 | 3.70986E-05 | DOWN |
| ENSSSCG00000013049 | RCOR2 | -1.333097901 | 0.000358582 | DOWN |
| ENSSSCG00000024481 | NA | -1.334687481 | 0.000909197 | DOWN |
| ENSSSCG00000013909 | CRLF1 | -1.340542422 | 0.004542333 | DOWN |
| ENSSSCG00000038149 | KCNE4 | -1.347713731 | 0.000167906 | DOWN |
| ENSSSCG00000031849 | NA | -1.347737935 | 0.014064807 | DOWN |
| ENSSSCG00000004663 | SEMA6D | -1.350172773 | 0.015932187 | DOWN |
| ENSSSCG00000026516 | EPHB3 | -1.358861071 | 0.001780955 | DOWN |
| ENSSSCG00000026425 | ADAMTSL2 | -1.367969834 | 0.006848863 | DOWN |
| ENSSSCG00000027872 | MYBL1 | -1.370157703 | 0.030256445 | DOWN |
| ENSSSCG00000012504 | NAP1L3 | -1.376694496 | 0.000717082 | DOWN |
| ENSSSCG00000003108 | NPAS1 | -1.376931442 | 0.019325114 | DOWN |
| ENSSSCG00000023526 | RAPGEF3 | -1.376976135 | 0.00402107 | DOWN |
| ENSSSCG00000025308 | IL17D | -1.387443095 | 0.00019812 | DOWN |
| ENSSSCG00000039332 | SEC16B | -1.390730802 | 0.001456045 | DOWN |
| ENSSSCG00000017254 | MAP2K6 | -1.395334938 | 0.025129322 | DOWN |
| ENSSSCG00000032063 | THEM6 | -1.39579614 | 0.044671149 | DOWN |
| ENSSSCG00000015223 | DDX25 | -1.402822098 | 0.003441244 | DOWN |
| ENSSSCG00000029811 | PLCL2 | -1.413519364 | 2.46123E-06 | DOWN |
| ENSSSCG00000016794 | MYO10 | -1.419666979 | 4.49475E-05 | DOWN |
| ENSSSCG00000001910 | ISLR | -1.422542453 | 0.01356273 | DOWN |
| ENSSSCG00000006344 | NOS1AP | -1.42624843 | 0.029811571 | DOWN |
| ENSSSCG00000023273 | SH3YL1 | -1.428206426 | 0.015969773 | DOWN |
| ENSSSCG00000005657 | PKN3 | -1.43016478 | 0.041823167 | DOWN |
| ENSSSCG00000003439 | DHRS3 | -1.436942569 | 0.004178069 | DOWN |
| ENSSSCG00000040267 | CYS1 | -1.44171909 | 0.000443153 | DOWN |
| ENSSSCG00000014994 | PDGFD | -1.448447976 | 0.001541962 | DOWN |
| ENSSSCG00000006687 | ITGA10 | -1.449445644 | 0.000141585 | DOWN |
| ENSSSCG00000039703 | EEPD1 | -1.46972161 | 0.025115673 | DOWN |
| ENSSSCG00000014581 | TUB | -1.471800366 | 0.000106082 | DOWN |
| ENSSSCG00000037835 | TRIM7 | -1.484867976 | 0.009829189 | DOWN |
| ENSSSCG00000009720 | DDX60 | -1.48516488 | 0.036087089 | DOWN |
| ENSSSCG00000021207 | HESX1 | -1.485890278 | 0.001395281 | DOWN |
| ENSSSCG00000008838 | LNX1 | -1.489925964 | 6.51967E-15 | DOWN |
| ENSSSCG00000015368 | HDAC9 | -1.492105462 | 0.000996674 | DOWN |
| ENSSSCG00000012295 | MAGIX | -1.501279239 | 0.015560531 | DOWN |
| ENSSSCG00000037015 | SESN3 | -1.501737326 | 0.001890265 | DOWN |
| ENSSSCG00000006582 | S100A14 | -1.502454781 | 0.001055386 | DOWN |
| ENSSSCG00000023618 | FRMD7 | -1.502910823 | 0.046963774 | DOWN |
| ENSSSCG00000001203 | ZSCAN9 | -1.503041105 | 0.00095731 | DOWN |
| ENSSSCG00000035284 | BMF | -1.509948115 | 0.002499541 | DOWN |
| ENSSSCG00000017511 | PLXDC1 | -1.513177425 | 0.041616259 | DOWN |
| ENSSSCG00000036679 | SORBS2 | -1.514669344 | 0.000355276 | DOWN |
| ENSSSCG00000017255 | ABCA5 | -1.52209553 | 0.00473417 | DOWN |
| ENSSSCG00000012699 | NA | -1.530451791 | 3.48765E-08 | DOWN |
| ENSSSCG00000033648 | NA | -1.533192559 | 0.006605559 | DOWN |
| ENSSSCG00000040053 | LSMEM1 | -1.542157949 | 0.00959498 | DOWN |
| ENSSSCG00000006923 | GBP2 | -1.55104159 | 0.049099798 | DOWN |
| ENSSSCG00000022361 | NA | -1.56206926 | 0.002902068 | DOWN |
| ENSSSCG00000004281 | KCNQ5 | -1.56350775 | 0.00528125 | DOWN |
| ENSSSCG00000016863 | OXCT1 | -1.56531072 | 2.68795E-07 | DOWN |
| ENSSSCG00000040581 | CISH | -1.567330883 | 3.23215E-05 | DOWN |
| ENSSSCG00000038660 | HAAO | -1.574412937 | 0.022232361 | DOWN |
| ENSSSCG00000007073 | ISM1 | -1.575339166 | 0.018685066 | DOWN |
| ENSSSCG00000022247 | PROSER2 | -1.583280961 | 0.001619841 | DOWN |
| ENSSSCG00000030827 | FGFR3 | -1.584394443 | 0.009182771 | DOWN |
| ENSSSCG00000017868 | NA | -1.59111081 | 0.030256445 | DOWN |
| ENSSSCG00000038969 | DMPK | -1.601224799 | 0.000257796 | DOWN |
| ENSSSCG00000003148 | DBP | -1.605501051 | 6.08355E-05 | DOWN |
| ENSSSCG00000033338 | NA | -1.606694343 | 0.020383416 | DOWN |
| ENSSSCG00000016823 | NA | -1.610832621 | 0.015270446 | DOWN |
| ENSSSCG00000005627 | AK1 | -1.61486193 | 0.002292462 | DOWN |
| ENSSSCG00000009844 | HSPB8 | -1.61608566 | 1.67845E-05 | DOWN |
| ENSSSCG00000004602 | TEX9 | -1.618433735 | 0.012762406 | DOWN |
| ENSSSCG00000040603 | SGTB | -1.618492651 | 0.003056568 | DOWN |
| ENSSSCG00000005494 | TNC | -1.621623532 | 0.026429672 | DOWN |
| ENSSSCG00000040719 | KIAA0040 | -1.628336742 | 4.38403E-07 | DOWN |
| ENSSSCG00000001427 | C4A | -1.632099008 | 0.049099798 | DOWN |
| ENSSSCG00000000910 | CRADD | -1.636174104 | 1.36216E-10 | DOWN |
| ENSSSCG00000001834 | MFGE8 | -1.660397595 | 0.000898418 | DOWN |
| ENSSSCG00000021440 | GPSM2 | -1.66095467 | 2.73606E-05 | DOWN |
| ENSSSCG00000013382 | PLEKHA7 | -1.661527806 | 0.043107531 | DOWN |
| ENSSSCG00000001064 | GMPR | -1.664971008 | 0.048236218 | DOWN |
| ENSSSCG00000027466 | PCOLCE | -1.66511534 | 0.000206099 | DOWN |
| ENSSSCG00000022504 | CDON | -1.667299414 | 3.22775E-06 | DOWN |
| ENSSSCG00000004136 | AIG1 | -1.668337722 | 3.486E-10 | DOWN |
| ENSSSCG00000001873 | CSPG4 | -1.671901431 | 0.024732591 | DOWN |
| ENSSSCG00000006688 | ANKRD35 | -1.674789422 | 3.65312E-05 | DOWN |
| ENSSSCG00000029186 | SEZ6L2 | -1.677913069 | 0.001841683 | DOWN |
| ENSSSCG00000005094 | TMEM30B | -1.688071387 | 2.57437E-05 | DOWN |
| ENSSSCG00000017583 | SGCA | -1.688364983 | 0.000786239 | DOWN |
| ENSSSCG00000008230 | ATOH8 | -1.690462416 | 0.001960405 | DOWN |
| ENSSSCG00000036007 | MFAP4 | -1.695287692 | 0.00088835 | DOWN |
| ENSSSCG00000030217 | COLGALT2 | -1.711624416 | 0.000452944 | DOWN |
| ENSSSCG00000007463 | PTGIS | -1.717654333 | 1.92608E-10 | DOWN |
| ENSSSCG00000015913 | SCN9A | -1.719937001 | 0.013498184 | DOWN |
| ENSSSCG00000006748 | TSPAN2 | -1.720506128 | 0.005783223 | DOWN |
| ENSSSCG00000010698 | FGFR2 | -1.723412759 | 0.030154472 | DOWN |
| ENSSSCG00000011208 | ZNF385D | -1.724714122 | 0.00012707 | DOWN |
| ENSSSCG00000031346 | CMKLR1 | -1.73506943 | 0.00422557 | DOWN |
| ENSSSCG00000038838 | DLX5 | -1.738423008 | 0.002186605 | DOWN |
| ENSSSCG00000008468 | PKDCC | -1.74642625 | 0.001991362 | DOWN |
| ENSSSCG00000015271 | PRELP | -1.750179803 | 0.005202267 | DOWN |
| ENSSSCG00000035169 | HOTAIRM1_3 | -1.758153572 | 0.002756754 | DOWN |
| ENSSSCG00000016717 | MPP6 | -1.767170469 | 0.000228386 | DOWN |
| ENSSSCG00000030241 | TSC22D3 | -1.771889279 | 2.15069E-09 | DOWN |
| ENSSSCG00000014219 | CDO1 | -1.774170188 | 0.000766207 | DOWN |
| ENSSSCG00000012528 | BEX3 | -1.777421943 | 0.000154407 | DOWN |
| ENSSSCG00000035223 | SYNM | -1.782022917 | 1.08501E-08 | DOWN |
| ENSSSCG00000003633 | TEKT2 | -1.789352785 | 0.000391577 | DOWN |
| ENSSSCG00000004781 | NA | -1.806977874 | 0.009705766 | DOWN |
| ENSSSCG00000035243 | RAB27B | -1.80941767 | 0.000339329 | DOWN |
| ENSSSCG00000023522 | TGM2 | -1.812402603 | 0.01179495 | DOWN |
| ENSSSCG00000039651 | SLC2A5 | -1.813448049 | 0.007432134 | DOWN |
| ENSSSCG00000026142 | TNK1 | -1.814510114 | 0.018322822 | DOWN |
| ENSSSCG00000039802 | FBXL2 | -1.820399558 | 0.0002114 | DOWN |
| ENSSSCG00000015045 | NCAM1 | -1.826915515 | 0.025851868 | DOWN |
| ENSSSCG00000030113 | SHISA2 | -1.835107188 | 0.005507124 | DOWN |
| ENSSSCG00000033388 | NA | -1.841436551 | 0.014407742 | DOWN |
| ENSSSCG00000015399 | SEMA3E | -1.845045766 | 0.025272192 | DOWN |
| ENSSSCG00000000195 | PRPH | -1.851450091 | 0.040264298 | DOWN |
| ENSSSCG00000016784 | ANKH | -1.856601283 | 1.71051E-14 | DOWN |
| ENSSSCG00000039568 | SNAI2 | -1.860313393 | 0.000602814 | DOWN |
| ENSSSCG00000015270 | FMOD | -1.865896424 | 0.000158417 | DOWN |
| ENSSSCG00000012848 | EPS8L2 | -1.881778238 | 0.042997207 | DOWN |
| ENSSSCG00000016259 | FBXO36 | -1.893218569 | 0.048771456 | DOWN |
| ENSSSCG00000003147 | CA11 | -1.89375591 | 0.001358621 | DOWN |
| ENSSSCG00000016589 | LRRC4 | -1.908459118 | 0.00339482 | DOWN |
| ENSSSCG00000016093 | NA | -1.91263276 | 0.004927815 | DOWN |
| ENSSSCG00000031244 | GAP43 | -1.913800547 | 0.001837646 | DOWN |
| ENSSSCG00000017904 | ENO3 | -1.914534565 | 2.95003E-06 | DOWN |
| ENSSSCG00000010101 | P2RX6 | -1.924690365 | 0.007638848 | DOWN |
| ENSSSCG00000038290 | RNF182 | -1.933511073 | 0.013160514 | DOWN |
| ENSSSCG00000015556 | LAMC2 | -1.94080331 | 0.025486725 | DOWN |
| ENSSSCG00000015109 | CCDC153 | -1.950620292 | 0.002500704 | DOWN |
| ENSSSCG00000010627 | PDCD4 | -1.964427631 | 5.8393E-05 | DOWN |
| ENSSSCG00000011077 | NA | -1.96870101 | 0.004079841 | DOWN |
| ENSSSCG00000016866 | GHR | -1.971797169 | 1.05738E-09 | DOWN |
| ENSSSCG00000038384 | COX4I2 | -1.974129307 | 0.00190918 | DOWN |
| ENSSSCG00000031074 | FAM110D | -1.981345923 | 0.030661349 | DOWN |
| ENSSSCG00000005992 | SHAS2 | -2.003220696 | 0.005272726 | DOWN |
| ENSSSCG00000033509 | SAMD11 | -2.006089235 | 0.000193002 | DOWN |
| ENSSSCG00000016006 | NA | -2.013740198 | 0.044419819 | DOWN |
| ENSSSCG00000011831 | APOD | -2.026923108 | 0.002298062 | DOWN |
| ENSSSCG00000031487 | LSP1 | -2.03396735 | 0.000106082 | DOWN |
| ENSSSCG00000027157 | SLC40A1 | -2.042164225 | 0.000504544 | DOWN |
| ENSSSCG00000033314 | DLX6 | -2.049959099 | 0.006848863 | DOWN |
| ENSSSCG00000003410 | MASP2 | -2.059142383 | 0.003262439 | DOWN |
| ENSSSCG00000004891 | SERPINB7 | -2.064094221 | 0.00157127 | DOWN |
| ENSSSCG00000001620 | MDFI | -2.069709134 | 0.000119549 | DOWN |
| ENSSSCG00000003909 | NA | -2.070264947 | 0.000329311 | DOWN |
| ENSSSCG00000011582 | CAND2 | -2.073866694 | 4.38403E-07 | DOWN |
| ENSSSCG00000001832 | ACAN | -2.081920565 | 0.005459318 | DOWN |
| ENSSSCG00000027928 | TMEM9 | -2.082974534 | 0.001560306 | DOWN |
| ENSSSCG00000001807 | AP3B2 | -2.086491359 | 0.013619296 | DOWN |
| ENSSSCG00000004565 | CA12 | -2.09904219 | 0.045035204 | DOWN |
| ENSSSCG00000031866 | TIMP3 | -2.099195691 | 1.95697E-05 | DOWN |
| ENSSSCG00000035055 | NA | -2.117190721 | 0.024868334 | DOWN |
| ENSSSCG00000026404 | SERTAD4 | -2.124709172 | 0.000174051 | DOWN |
| ENSSSCG00000016983 | STC2 | -2.141137504 | 1.34224E-06 | DOWN |
| ENSSSCG00000013344 | ANO5 | -2.141180306 | 0.007507998 | DOWN |
| ENSSSCG00000022429 | KAZALD1 | -2.148567014 | 0.000724744 | DOWN |
| ENSSSCG00000021997 | ALS2CL | -2.158395373 | 0.00422093 | DOWN |
| ENSSSCG00000035419 | RARRES2 | -2.170166141 | 3.27573E-05 | DOWN |
| ENSSSCG00000011928 | CCDC80 | -2.1833089 | 0.000588347 | DOWN |
| ENSSSCG00000033993 | PLCXD3 | -2.195481191 | 0.003859735 | DOWN |
| ENSSSCG00000025777 | ESR1 | -2.20752187 | 0.000333588 | DOWN |
| ENSSSCG00000015396 | SEMA3D | -2.225759056 | 0.00041239 | DOWN |
| ENSSSCG00000010816 | TGFB2 | -2.261764751 | 5.59485E-08 | DOWN |
| ENSSSCG00000010529 | SFRP5 | -2.264778124 | 0.010392868 | DOWN |
| ENSSSCG00000024954 | FGF1 | -2.28140824 | 0.01116806 | DOWN |
| ENSSSCG00000004928 | CILP | -2.281946765 | 0.00105179 | DOWN |
| ENSSSCG00000014137 | HAPLN1 | -2.298508741 | 0.001613624 | DOWN |
| ENSSSCG00000004013 | SMOC2 | -2.300720823 | 0.000464187 | DOWN |
| ENSSSCG00000036438 | GPX3 | -2.307674835 | 0.002731834 | DOWN |
| ENSSSCG00000000602 | RERG | -2.312068103 | 0.000895224 | DOWN |
| ENSSSCG00000016841 | SLC1A3 | -2.328238913 | 0.024678429 | DOWN |
| ENSSSCG00000013294 | LDLRAD3 | -2.332881796 | 0.000145562 | DOWN |
| ENSSSCG00000021027 | PGBD5 | -2.337326935 | 0.025928571 | DOWN |
| ENSSSCG00000026689 | CCDC114 | -2.339431115 | 0.000998216 | DOWN |
| ENSSSCG00000022592 | FIBIN | -2.370855529 | 4.19648E-05 | DOWN |
| ENSSSCG00000030597 | HAPLN3 | -2.37201608 | 0.000688472 | DOWN |
| ENSSSCG00000000492 | LYZ | -2.37632205 | 0.003080201 | DOWN |
| ENSSSCG00000024223 | ARHGEF16 | -2.382247718 | 0.005144186 | DOWN |
| ENSSSCG00000011837 | MELTF | -2.390166682 | 0.021003801 | DOWN |
| ENSSSCG00000033235 | NA | -2.397654127 | 0.023157142 | DOWN |
| ENSSSCG00000004573 | NA | -2.404642511 | 0.000699801 | DOWN |
| ENSSSCG00000039364 | NA | -2.445745163 | 0.034707047 | DOWN |
| ENSSSCG00000034266 | NA | -2.449056519 | 0.019584581 | DOWN |
| ENSSSCG00000039182 | C11orf96 | -2.453415898 | 0.000106082 | DOWN |
| ENSSSCG00000017938 | YBX2 | -2.480643158 | 6.20317E-05 | DOWN |
| ENSSSCG00000000749 | SLC6A12 | -2.490893537 | 5.05554E-06 | DOWN |
| ENSSSCG00000001473 | COL11A2 | -2.494296568 | 0.023072256 | DOWN |
| ENSSSCG00000007949 | SRL | -2.50245759 | 0.019522447 | DOWN |
| ENSSSCG00000036748 | NA | -2.527731314 | 0.000433048 | DOWN |
| ENSSSCG00000017257 | ABCA9 | -2.540279621 | 0.000616382 | DOWN |
| ENSSSCG00000035798 | PRTG | -2.562194346 | 0.037555581 | DOWN |
| ENSSSCG00000006034 | RSPO2 | -2.569751998 | 0.004889212 | DOWN |
| ENSSSCG00000034178 | AIF1L | -2.575435246 | 0.025272192 | DOWN |
| ENSSSCG00000035729 | MYOZ3 | -2.57604497 | 0.010821409 | DOWN |
| ENSSSCG00000006933 | CLCA1 | -2.612060376 | 0.001983046 | DOWN |
| ENSSSCG00000026407 | NCCRP1 | -2.621487814 | 0.000240435 | DOWN |
| ENSSSCG00000006001 | ENPP2 | -2.656696793 | 0.011305294 | DOWN |
| ENSSSCG00000011455 | CACNA1D | -2.681138572 | 0.002042642 | DOWN |
| ENSSSCG00000013283 | ACCSL | -2.700377045 | 0.025120168 | DOWN |
| ENSSSCG00000035960 | NA | -2.704929054 | 0.010761557 | DOWN |
| ENSSSCG00000003333 | C1QTNF12 | -2.727365814 | 0.042997207 | DOWN |
| ENSSSCG00000011014 | BAMBI | -2.729012224 | 1.16485E-07 | DOWN |
| ENSSSCG00000026427 | RORC | -2.745869363 | 6.12369E-06 | DOWN |
| ENSSSCG00000011326 | PTH1R | -2.754297048 | 2.31034E-06 | DOWN |
| ENSSSCG00000039261 | WSCD2 | -2.796313388 | 0.0002748 | DOWN |
| ENSSSCG00000017569 | CHAD | -2.849221796 | 1.28717E-05 | DOWN |
| ENSSSCG00000006857 | COL11A1 | -2.855714708 | 3.26498E-06 | DOWN |
| ENSSSCG00000004225 | TPD52L1 | -2.868148322 | 0.01725702 | DOWN |
| ENSSSCG00000034323 | HOTAIRM1_4 | -2.903021536 | 0.030659523 | DOWN |
| ENSSSCG00000037335 | NA | -2.906600205 | 0.017815336 | DOWN |
| ENSSSCG00000036647 | SPIN4 | -2.917041875 | 0.015270446 | DOWN |
| ENSSSCG00000023537 | SYT8 | -2.920757497 | 0.016984944 | DOWN |
| ENSSSCG00000008397 | EFEMP1 | -2.923641208 | 0.00181905 | DOWN |
| ENSSSCG00000003592 | SDC3 | -2.949439779 | 1.0051E-07 | DOWN |
| ENSSSCG00000023261 | GDF5 | -2.9660296 | 0.002015043 | DOWN |
| ENSSSCG00000015353 | SCIN | -2.967424824 | 0.002345482 | DOWN |
| ENSSSCG00000008101 | FBLN7 | -2.977616465 | 2.31034E-06 | DOWN |
| ENSSSCG00000010142 | RYR2 | -2.984272184 | 0.008039791 | DOWN |
| ENSSSCG00000030076 | SLC6A13 | -2.991453072 | 0.01270835 | DOWN |
| ENSSSCG00000025423 | KCNK5 | -2.998884917 | 4.22366E-06 | DOWN |
| ENSSSCG00000021941 | NA | -2.999260439 | 0.023072256 | DOWN |
| ENSSSCG00000036157 | BARX2 | -3.041709809 | 0.047726892 | DOWN |
| ENSSSCG00000031053 | S100A1 | -3.044393432 | 2.31034E-06 | DOWN |
| ENSSSCG00000023915 | SLC2A4 | -3.053848397 | 0.010969036 | DOWN |
| ENSSSCG00000024492 | EPHB1 | -3.104862674 | 0.009944932 | DOWN |
| ENSSSCG00000009283 | TNFRSF19 | -3.105399309 | 5.67968E-07 | DOWN |
| ENSSSCG00000004939 | MEGF11 | -3.108579851 | 0.000329311 | DOWN |
| ENSSSCG00000025240 | DDIT4L | -3.119929848 | 0.026670498 | DOWN |
| ENSSSCG00000031738 | NA | -3.126895081 | 0.039544212 | DOWN |
| ENSSSCG00000003092 | FOXA3 | -3.169068417 | 0.000356369 | DOWN |
| ENSSSCG00000036907 | NMB | -3.190119551 | 5.00306E-05 | DOWN |
| ENSSSCG00000026868 | LRRC15 | -3.198265433 | 0.001333772 | DOWN |
| ENSSSCG00000036060 | RRAD | -3.229792843 | 6.98523E-07 | DOWN |
| ENSSSCG00000006808 | SLC16A4 | -3.265181517 | 0.001615919 | DOWN |
| ENSSSCG00000001490 | KHDRBS2 | -3.273066927 | 0.028743306 | DOWN |
| ENSSSCG00000014011 | RASGEF1C | -3.352179574 | 3.26308E-06 | DOWN |
| ENSSSCG00000008501 | VIT | -3.358563245 | 0.000687136 | DOWN |
| ENSSSCG00000040160 | MMP24 | -3.390800859 | 0.008462109 | DOWN |
| ENSSSCG00000030361 | PRKCZ | -3.433031566 | 0.001797683 | DOWN |
| ENSSSCG00000002954 | SPINT2 | -3.479160557 | 0.002642695 | DOWN |
| ENSSSCG00000015281 | PLEKHA6 | -3.502503491 | 0.001615919 | DOWN |
| ENSSSCG00000007391 | MATN4 | -3.520649516 | 0.000146554 | DOWN |
| ENSSSCG00000017747 | RAB11FIP4 | -3.557936342 | 1.26582E-07 | DOWN |
| ENSSSCG00000002297 | RDH12 | -3.584929352 | 0.009653566 | DOWN |
| ENSSSCG00000026517 | CALML4 | -3.588762511 | 8.93779E-05 | DOWN |
| ENSSSCG00000040554 | NA | -3.59846064 | 0.000987837 | DOWN |
| ENSSSCG00000016018 | FRZB | -3.611467946 | 0.000358582 | DOWN |
| ENSSSCG00000037067 | NA | -3.678896224 | 0.028212218 | DOWN |
| ENSSSCG00000008314 | NA | -3.690083577 | 0.001876223 | DOWN |
| ENSSSCG00000035928 | NA | -3.786026147 | 0.03859675 | DOWN |
| ENSSSCG00000026932 | RS1 | -3.787716635 | 0.000525069 | DOWN |
| ENSSSCG00000039358 | SUSD5 | -3.792108555 | 5.38508E-05 | DOWN |
| ENSSSCG00000040989 | GPRC5C | -3.802597624 | 3.43085E-05 | DOWN |
| ENSSSCG00000015618 | LAMB3 | -3.843698405 | 3.75803E-05 | DOWN |
| ENSSSCG00000011053 | CDNF | -3.853909854 | 0.04479105 | DOWN |
| ENSSSCG00000032203 | EPPK1 | -3.955798237 | 0.033941407 | DOWN |
| ENSSSCG00000040711 | NA | -3.989105742 | 0.006224343 | DOWN |
| ENSSSCG00000012584 | CAPN6 | -4.014385807 | 2.04233E-05 | DOWN |
| ENSSSCG00000016290 | EFHD1 | -4.088027701 | 0.000145562 | DOWN |
| ENSSSCG00000040513 | AQP3 | -4.104835002 | 6.43662E-05 | DOWN |
| ENSSSCG00000010428 | DKK1 | -4.168864651 | 0.04399401 | DOWN |
| ENSSSCG00000004422 | WISP3 | -4.192038895 | 1.21389E-05 | DOWN |
| ENSSSCG00000000591 | PIK3C2G | -4.196529532 | 0.008462109 | DOWN |
| ENSSSCG00000004191 | MOXD1 | -4.258602277 | 2.39341E-05 | DOWN |
| ENSSSCG00000011397 | SLC38A3 | -4.265469937 | 0.000634007 | DOWN |
| ENSSSCG00000013093 | VWCE | -4.271571751 | 0.0181716 | DOWN |
| ENSSSCG00000023403 | NA | -4.296323546 | 0.000160413 | DOWN |
| ENSSSCG00000037697 | MGP | -4.326148183 | 2.37859E-05 | DOWN |
| ENSSSCG00000038693 | RAB19 | -4.36795564 | 0.001692471 | DOWN |
| ENSSSCG00000014827 | PLEKHB1 | -4.462319025 | 0.002080326 | DOWN |
| ENSSSCG00000007385 | KCNS1 | -4.483351598 | 1.19942E-06 | DOWN |
| ENSSSCG00000025523 | COL2A1 | -4.592821558 | 6.09073E-06 | DOWN |
| ENSSSCG00000008725 | CYTL1 | -4.628802414 | 0.004814044 | DOWN |
| ENSSSCG00000016294 | C2orf82 | -4.641624123 | 0.000436521 | DOWN |
| ENSSSCG00000013385 | INSC | -4.680025755 | 2.0898E-05 | DOWN |
| ENSSSCG00000006171 | CRISPLD1 | -4.72961188 | 3.96742E-16 | DOWN |
| ENSSSCG00000023322 | NA | -4.732693275 | 0.000199379 | DOWN |
| ENSSSCG00000037881 | GDF5OS | -4.736589973 | 0.045096525 | DOWN |
| ENSSSCG00000037846 | ACOXL | -4.750843198 | 0.004083891 | DOWN |
| ENSSSCG00000000029 | SCUBE1 | -4.961037408 | 7.84943E-06 | DOWN |
| ENSSSCG00000004274 | NA | -4.963548471 | 0.001890265 | DOWN |
| ENSSSCG00000032400 | C1QTNF8 | -4.992426129 | 0.000151744 | DOWN |
| ENSSSCG00000033124 | NA | -5.121894838 | 0.017138012 | DOWN |
| ENSSSCG00000034604 | CLEC3A | -5.128423797 | 5.77801E-07 | DOWN |
| ENSSSCG00000032048 | SCRG1 | -5.172002189 | 5.77801E-07 | DOWN |
| ENSSSCG00000025483 | GREB1 | -5.192449271 | 0.000920419 | DOWN |
| ENSSSCG00000030998 | WIF1 | -5.216896547 | 0.000473959 | DOWN |
| ENSSSCG00000010545 | CPN1 | -5.250748385 | 0.020523604 | DOWN |
| ENSSSCG00000026943 | MRAP2 | -5.268378863 | 0.000181427 | DOWN |
| ENSSSCG00000021573 | KCNJ5 | -5.26865508 | 1.28717E-05 | DOWN |
| ENSSSCG00000033344 | H19_2 | -5.269875598 | 0.015034397 | DOWN |
| ENSSSCG00000017563 | MYCBPAP | -5.340883999 | 0.012572185 | DOWN |
| ENSSSCG00000034181 | NKX3-2 | -5.352380292 | 6.66593E-05 | DOWN |
| ENSSSCG00000003374 | ESPN | -5.409344504 | 9.5594E-07 | DOWN |
| ENSSSCG00000033234 | SSTR5 | -5.615788633 | 0.019211344 | DOWN |
| ENSSSCG00000000963 | SYCE3 | -5.691380201 | 0.014064807 | DOWN |
| ENSSSCG00000014066 | TMEM171 | -5.702548646 | 1.30935E-06 | DOWN |
| ENSSSCG00000004614 | UNC13C | -5.822836725 | 0.036008454 | DOWN |
| ENSSSCG00000006031 | TMEM74 | -6.009322239 | 0.00181905 | DOWN |
| ENSSSCG00000031903 | TNNT3 | -6.042999117 | 0.005599552 | DOWN |
| ENSSSCG00000002997 | NA | -6.073916191 | 6.21826E-06 | DOWN |
| ENSSSCG00000002476 | SERPINA1 | -6.165907586 | 0.001320177 | DOWN |
| ENSSSCG00000009500 | NA | -6.625595196 | 0.007432134 | DOWN |
| ENSSSCG00000021053 | GIPR | -6.689820617 | 0.001541962 | DOWN |
| ENSSSCG00000017498 | PPP1R1B | -6.73745676 | 0.000371983 | DOWN |
| ENSSSCG00000010800 | B3GALT2 | -6.754245695 | 0.013002377 | DOWN |
| ENSSSCG00000009219 | IBSP | -7.083092022 | 0.000588347 | DOWN |
| ENSSSCG00000029558 | EXTL1 | -7.164040605 | 6.08706E-05 | DOWN |
| ENSSSCG00000036474 | GJB6 | -7.998825822 | 0.000890849 | DOWN |
| ENSSSCG00000022554 | MATN1 | -10.18904535 | 8.02154E-07 | DOWN |

Gene Name “NA” indicates the gene ID was not matched to a HGNC gene name.
