# Supplementary material for: A Tale of Two Loads: Modulation of IL-1 Induced Inflammatory Responses of Meniscal Cells in Two Models of Dynamic Physiologic Loading
Source: Front Bioeng Biotechnol. 2022 Mar 1;10:837619. doi: 10.3389/fbioe.2022.837619 (PMC8921261; doi:10.3389/fbioe.2022.837619)
Supplement: Supplementary file 2 [file DataSheet13.DOCX]

**Supplemental Table 14**: Genes with a significant (p<0.05) interaction effect between load (5% cyclic tensile stretch) and IL-1α treatment, inner zone cells.

| **Gene ID** | **Gene Name** | **p-value** |
| --- | --- | --- |
| ENSSSCG00000031380 | NA | 3.94E-95 |
| ENSSSCG00000010454 | IFIT5 | 1.46E-81 |
| ENSSSCG00000025618 | TAP1 | 4.75E-67 |
| ENSSSCG00000039862 | TRIB3 | 2.49E-65 |
| ENSSSCG00000005724 | SETX | 7.42E-62 |
| ENSSSCG00000000411 | NAB2 | 2.5E-61 |
| ENSSSCG00000016512 | ZC3HAV1 | 3E-61 |
| ENSSSCG00000016438 | NUB1 | 2.57E-53 |
| ENSSSCG00000001233 | TRIM26 | 1.36E-52 |
| ENSSSCG00000004657 | CEP152 | 2.45E-50 |
| ENSSSCG00000030108 | ZNFX1 | 1.47E-48 |
| ENSSSCG00000032652 | NA | 1.66E-47 |
| ENSSSCG00000011465 | NA | 1.66E-47 |
| ENSSSCG00000020666 | EHD4 | 5.85E-46 |
| ENSSSCG00000005481 | NA | 3.59E-44 |
| ENSSSCG00000001912 | PML | 2.47E-43 |
| ENSSSCG00000004464 | FAM46A | 2.96E-43 |
| ENSSSCG00000003471 | EPHA2 | 5.87E-43 |
| ENSSSCG00000021383 | CGAS | 3.95E-42 |
| ENSSSCG00000026454 | NA | 3.95E-42 |
| ENSSSCG00000006209 | VCPIP1 | 5.77E-42 |
| ENSSSCG00000040887 | PAPD5 | 3.76E-41 |
| ENSSSCG00000035420 | HES4 | 1.79E-40 |
| ENSSSCG00000010261 | PPA1 | 1.12E-39 |
| ENSSSCG00000013599 | ANGPTL4 | 1.68E-39 |
| ENSSSCG00000028536 | LHFPL2 | 6.72E-39 |
| ENSSSCG00000035153 | TRIM38 | 1.13E-38 |
| ENSSSCG00000012890 | TCIRG1 | 2.73E-38 |
| ENSSSCG00000031201 | LMOD1 | 3.68E-38 |
| ENSSSCG00000027806 | SAMHD1 | 3.73E-38 |
| ENSSSCG00000017723 | CCL2 | 3.73E-38 |
| ENSSSCG00000015545 | GLUL | 1.74E-37 |
| ENSSSCG00000016057 | STAT1 | 2.52E-37 |
| ENSSSCG00000021597 | PHLDA2 | 3.71E-37 |
| ENSSSCG00000032613 | SNAI1 | 6.47E-37 |
| ENSSSCG00000016502 | PARP12 | 8.36E-37 |
| ENSSSCG00000037536 | SLC25A28 | 1.82E-36 |
| ENSSSCG00000003379 | KLHL21 | 1.91E-36 |
| ENSSSCG00000025593 | NA | 1.45E-34 |
| ENSSSCG00000035634 | NA | 2.4E-34 |
| ENSSSCG00000024219 | TIGAR | 2.41E-34 |
| ENSSSCG00000010540 | ENTPD7 | 3.4E-34 |
| ENSSSCG00000011859 | HEG1 | 2.99E-33 |
| ENSSSCG00000033321 | GAS1 | 6.08E-33 |
| ENSSSCG00000029438 | SESN2 | 6.56E-33 |
| ENSSSCG00000004897 | ZCCHC2 | 6.87E-33 |
| ENSSSCG00000008496 | EIF2AK2 | 7.08E-33 |
| ENSSSCG00000016857 | DAB2 | 7.71E-33 |
| ENSSSCG00000011495 | PRICKLE2 | 1.07E-32 |
| ENSSSCG00000016991 | DUSP1 | 1.4E-32 |
| ENSSSCG00000014985 | MMP3 | 1.49E-32 |
| ENSSSCG00000036322 | SPRY4 | 1.63E-32 |
| ENSSSCG00000035078 | CD40 | 3.35E-32 |
| ENSSSCG00000010996 | BAG1 | 5.92E-32 |
| ENSSSCG00000002841 | N4BP1 | 6.6E-32 |
| ENSSSCG00000012173 | SAT1 | 9.69E-32 |
| ENSSSCG00000016851 | OSMR | 4.81E-31 |
| ENSSSCG00000017614 | TRIM25 | 5.32E-31 |
| ENSSSCG00000015897 | IFIH1 | 8.7E-31 |
| ENSSSCG00000038185 | EREG | 1.44E-30 |
| ENSSSCG00000000396 | STAT2 | 3.08E-30 |
| ENSSSCG00000008957 | AMCF-II | 7.02E-30 |
| ENSSSCG00000015435 | NAMPT | 8.3E-30 |
| ENSSSCG00000017087 | GM2A | 1.31E-29 |
| ENSSSCG00000001081 | SOX4 | 1.64E-29 |
| ENSSSCG00000004971 | TLE3 | 2.62E-29 |
| ENSSSCG00000034191 | SOX6 | 4.32E-29 |
| ENSSSCG00000010312 | PLAU | 6.29E-29 |
| ENSSSCG00000023716 | TNFAIP6 | 6.95E-29 |
| ENSSSCG00000023956 | NA | 1.44E-28 |
| ENSSSCG00000015549 | RNASEL | 1.76E-28 |
| ENSSSCG00000040061 | NINJ1 | 2.64E-28 |
| ENSSSCG00000015525 | TOR3A | 2.68E-28 |
| ENSSSCG00000032861 | NUAK1 | 5.01E-28 |
| ENSSSCG00000012277 | TIMP1 | 7.22E-28 |
| ENSSSCG00000032622 | PPP3CC | 1.57E-27 |
| ENSSSCG00000006371 | USF1 | 1.57E-27 |
| ENSSSCG00000015014 | ZC3H12C | 4.57E-27 |
| ENSSSCG00000004053 | TAGAP | 5.85E-27 |
| ENSSSCG00000029260 | NDNF | 5.85E-27 |
| ENSSSCG00000014149 | MEF2C | 5.96E-27 |
| ENSSSCG00000020906 | TNFSF10 | 6.24E-27 |
| ENSSSCG00000031882 | PNPT1 | 1.39E-26 |
| ENSSSCG00000013114 | SLC15A3 | 1.67E-26 |
| ENSSSCG00000010452 | IFIT1 | 5.24E-26 |
| ENSSSCG00000007073 | ISM1 | 6.38E-26 |
| ENSSSCG00000001509 | DAXX | 2.16E-25 |
| ENSSSCG00000025788 | ENPP4 | 2.38E-25 |
| ENSSSCG00000037015 | SESN3 | 2.39E-25 |
| ENSSSCG00000014835 | C2CD3 | 3.51E-25 |
| ENSSSCG00000022961 | CLMP | 8.73E-25 |
| ENSSSCG00000013366 | LDHA | 8.73E-25 |
| ENSSSCG00000015782 | IRF2 | 9.28E-25 |
| ENSSSCG00000006127 | NBN | 1.21E-24 |
| ENSSSCG00000005308 | RUSC2 | 1.44E-24 |
| ENSSSCG00000035958 | EVA1A | 1.92E-24 |
| ENSSSCG00000007067 | JAG1 | 2.94E-24 |
| ENSSSCG00000027340 | NA | 3.45E-24 |
| ENSSSCG00000026951 | PSMB8 | 5.46E-24 |
| ENSSSCG00000001661 | SRF | 8.07E-24 |
| ENSSSCG00000009440 | ELF1 | 1E-23 |
| ENSSSCG00000009542 | TNFSF13B | 1.08E-23 |
| ENSSSCG00000001463 | PSMB9 | 1.31E-23 |
| ENSSSCG00000028606 | ZBTB7B | 1.45E-23 |
| ENSSSCG00000033786 | NA | 1.82E-23 |
| ENSSSCG00000004789 | THBS1 | 2.76E-23 |
| ENSSSCG00000014242 | ZNF608 | 3.11E-23 |
| ENSSSCG00000006324 | ALDH9A1 | 3.87E-23 |
| ENSSSCG00000023522 | TGM2 | 5.43E-23 |
| ENSSSCG00000034645 | C3orf38 | 5.44E-23 |
| ENSSSCG00000003670 | RLF | 6.35E-23 |
| ENSSSCG00000009111 | SYNPO2 | 6.52E-23 |
| ENSSSCG00000039080 | TRIB2 | 7.85E-23 |
| ENSSSCG00000027709 | PARP9 | 8.74E-23 |
| ENSSSCG00000003839 | NA | 1.4E-22 |
| ENSSSCG00000038521 | CHAC1 | 1.56E-22 |
| ENSSSCG00000004830 | ATP10A | 1.76E-22 |
| ENSSSCG00000017420 | CNP | 2.59E-22 |
| ENSSSCG00000040815 | DUSP5 | 3.01E-22 |
| ENSSSCG00000027777 | NA | 3.62E-22 |
| ENSSSCG00000023247 | OPTN | 4.42E-22 |
| ENSSSCG00000028063 | TACC2 | 4.76E-22 |
| ENSSSCG00000013655 | ICAM1 | 7.23E-22 |
| ENSSSCG00000034293 | ARL4C | 7.32E-22 |
| ENSSSCG00000036679 | SORBS2 | 1.03E-21 |
| ENSSSCG00000015880 | TANC1 | 1.46E-21 |
| ENSSSCG00000016684 | SCRN1 | 1.96E-21 |
| ENSSSCG00000024973 | NA | 1.99E-21 |
| ENSSSCG00000022011 | NMI | 2.07E-21 |
| ENSSSCG00000023379 | UBE2L6 | 2.19E-21 |
| ENSSSCG00000013297 | CD44 | 2.31E-21 |
| ENSSSCG00000001516 | BAK1 | 2.68E-21 |
| ENSSSCG00000000774 | USP18 | 3.17E-21 |
| ENSSSCG00000014924 | CTSC | 3.8E-21 |
| ENSSSCG00000040719 | KIAA0040 | 3.85E-21 |
| ENSSSCG00000005208 | RIC1 | 4.24E-21 |
| ENSSSCG00000017306 | ITGB3 | 5.65E-21 |
| ENSSSCG00000012853 | IRF7 | 5.71E-21 |
| ENSSSCG00000005423 | ABCA1 | 6.34E-21 |
| ENSSSCG00000037572 | EPSTI1 | 7.61E-21 |
| ENSSSCG00000011390 | IP6K1 | 9.1E-21 |
| ENSSSCG00000032408 | CASP7 | 9.44E-21 |
| ENSSSCG00000025206 | RNF19B | 1.31E-20 |
| ENSSSCG00000040162 | NUPR1 | 1.79E-20 |
| ENSSSCG00000004421 | FYN | 2.57E-20 |
| ENSSSCG00000026466 | SLC23A2 | 2.68E-20 |
| ENSSSCG00000011874 | PARP14 | 2.94E-20 |
| ENSSSCG00000030548 | HERC5 | 3E-20 |
| ENSSSCG00000038549 | ZFP36L2 | 3.93E-20 |
| ENSSSCG00000031118 | PREX1 | 3.93E-20 |
| ENSSSCG00000030408 | DDX58 | 4.33E-20 |
| ENSSSCG00000016758 | NA | 6.05E-20 |
| ENSSSCG00000014909 | NA | 9.4E-20 |
| ENSSSCG00000002501 | NA | 9.44E-20 |
| ENSSSCG00000001229 | NA | 1.02E-19 |
| ENSSSCG00000012258 | FUNDC1 | 1.07E-19 |
| ENSSSCG00000009042 | OTUD4 | 2.2E-19 |
| ENSSSCG00000034802 | NA | 2.38E-19 |
| ENSSSCG00000005965 | MYC | 3.08E-19 |
| ENSSSCG00000027372 | SAMD9 | 3.53E-19 |
| ENSSSCG00000004509 | LIPG | 4.44E-19 |
| ENSSSCG00000027646 | TIPARP | 4.67E-19 |
| ENSSSCG00000036488 | KLF3 | 5.07E-19 |
| ENSSSCG00000033613 | FOXS1 | 6.92E-19 |
| ENSSSCG00000007733 | NA | 7.89E-19 |
| ENSSSCG00000011198 | RFTN1 | 9.74E-19 |
| ENSSSCG00000016261 | SP110 | 1.31E-18 |
| ENSSSCG00000014277 | IRF1 | 1.36E-18 |
| ENSSSCG00000036274 | NA | 1.39E-18 |
| ENSSSCG00000038491 | MEX3B | 1.61E-18 |
| ENSSSCG00000006066 | RNF19A | 1.66E-18 |
| ENSSSCG00000005364 | TDRD7 | 1.95E-18 |
| ENSSSCG00000036326 | LATS2 | 1.95E-18 |
| ENSSSCG00000004371 | CRYBG1 | 2.23E-18 |
| ENSSSCG00000000717 | KCNA6 | 2.68E-18 |
| ENSSSCG00000009125 | ANK2 | 3.89E-18 |
| ENSSSCG00000033089 | NA | 5.03E-18 |
| ENSSSCG00000027157 | SLC40A1 | 1.23E-17 |
| ENSSSCG00000014822 | ARHGEF17 | 1.82E-17 |
| ENSSSCG00000006009 | EXT1 | 1.9E-17 |
| ENSSSCG00000011239 | NA | 2.6E-17 |
| ENSSSCG00000025060 | NA | 2.72E-17 |
| ENSSSCG00000009370 | FOXO1 | 2.79E-17 |
| ENSSSCG00000032360 | PANX1 | 5.75E-17 |
| ENSSSCG00000026108 | CDC42EP1 | 6.28E-17 |
| ENSSSCG00000008123 | ARID5A | 6.68E-17 |
| ENSSSCG00000011496 | ADAMTS9 | 7.99E-17 |
| ENSSSCG00000033222 | TRIM14 | 8.73E-17 |
| ENSSSCG00000013432 | MIDN | 9.45E-17 |
| ENSSSCG00000004201 | TMEM200A | 9.45E-17 |
| ENSSSCG00000007874 | NA | 1.02E-16 |
| ENSSSCG00000012055 | MORC3 | 1.05E-16 |
| ENSSSCG00000039751 | NLRC5 | 1.2E-16 |
| ENSSSCG00000006105 | GEM | 1.27E-16 |
| ENSSSCG00000009968 | TTC28 | 1.42E-16 |
| ENSSSCG00000039582 | NA | 1.5E-16 |
| ENSSSCG00000014670 | TRIM5 | 1.67E-16 |
| ENSSSCG00000010451 | IFIT2 | 1.7E-16 |
| ENSSSCG00000017091 | TNIP1 | 1.74E-16 |
| ENSSSCG00000030300 | MT2A | 1.76E-16 |
| ENSSSCG00000035479 | DISP1 | 1.77E-16 |
| ENSSSCG00000015770 | VEGFC | 1.84E-16 |
| ENSSSCG00000035791 | SIX5 | 2.22E-16 |
| ENSSSCG00000007508 | ZBP1 | 2.39E-16 |
| ENSSSCG00000003928 | PLK3 | 2.69E-16 |
| ENSSSCG00000016900 | ESM1 | 3.21E-16 |
| ENSSSCG00000035859 | WNT5A | 3.48E-16 |
| ENSSSCG00000031579 | NA | 3.48E-16 |
| ENSSSCG00000034989 | LRRTM2 | 3.87E-16 |
| ENSSSCG00000012583 | ACSL4 | 4.13E-16 |
| ENSSSCG00000015444 | LAMB1 | 6.64E-16 |
| ENSSSCG00000038410 | CPEB2 | 6.64E-16 |
| ENSSSCG00000016062 | NABP1 | 7.87E-16 |
| ENSSSCG00000038535 | ARSB | 8.48E-16 |
| ENSSSCG00000021815 | NA | 9.51E-16 |
| ENSSSCG00000013147 | FAM111B | 1.31E-15 |
| ENSSSCG00000005096 | HIF1A | 1.5E-15 |
| ENSSSCG00000032436 | NA | 1.76E-15 |
| ENSSSCG00000011251 | MYD88 | 1.98E-15 |
| ENSSSCG00000037815 | ZC3H12A | 2.04E-15 |
| ENSSSCG00000040334 | CBX6 | 2.19E-15 |
| ENSSSCG00000026169 | MFSD12 | 2.42E-15 |
| ENSSSCG00000001667 | ZNF318 | 2.45E-15 |
| ENSSSCG00000009320 | FLT1 | 2.72E-15 |
| ENSSSCG00000006923 | GBP2 | 3.59E-15 |
| ENSSSCG00000017202 | H3F3A | 3.59E-15 |
| ENSSSCG00000010146 | LGALS8 | 3.68E-15 |
| ENSSSCG00000022101 | BRCA1 | 3.68E-15 |
| ENSSSCG00000023298 | SRXN1 | 3.89E-15 |
| ENSSSCG00000037958 | TOB2 | 4.07E-15 |
| ENSSSCG00000025281 | NA | 4.53E-15 |
| ENSSSCG00000012915 | CLCF1 | 5.39E-15 |
| ENSSSCG00000016101 | CFLAR | 5.47E-15 |
| ENSSSCG00000003579 | AHDC1 | 5.55E-15 |
| ENSSSCG00000012077 | MX1 | 5.89E-15 |
| ENSSSCG00000022312 | RHPN2 | 6.04E-15 |
| ENSSSCG00000022447 | F3 | 7.58E-15 |
| ENSSSCG00000034484 | SPEN | 9.32E-15 |
| ENSSSCG00000012076 | MX2 | 9.42E-15 |
| ENSSSCG00000009216 | SPP1 | 9.61E-15 |
| ENSSSCG00000005943 | ST3GAL1 | 1.11E-14 |
| ENSSSCG00000023400 | ZNF598 | 1.14E-14 |
| ENSSSCG00000033703 | FAM111A | 1.16E-14 |
| ENSSSCG00000005706 | ABL1 | 1.21E-14 |
| ENSSSCG00000030655 | MAMDC2 | 1.22E-14 |
| ENSSSCG00000016186 | TMBIM1 | 1.28E-14 |
| ENSSSCG00000022208 | TNFRSF1B | 1.49E-14 |
| ENSSSCG00000008648 | RSAD2 | 1.49E-14 |
| ENSSSCG00000009396 | PHF11 | 1.49E-14 |
| ENSSSCG00000009053 | RNF150 | 1.59E-14 |
| ENSSSCG00000001347 | PPP1R10 | 1.88E-14 |
| ENSSSCG00000010816 | TGFB2 | 1.96E-14 |
| ENSSSCG00000008787 | KLHL5 | 2.25E-14 |
| ENSSSCG00000024096 | RIPK2 | 2.37E-14 |
| ENSSSCG00000013307 | LMO2 | 2.44E-14 |
| ENSSSCG00000036893 | PTHLH | 2.53E-14 |
| ENSSSCG00000040317 | SOD2 | 2.89E-14 |
| ENSSSCG00000014780 | TRIM21 | 3.06E-14 |
| ENSSSCG00000017924 | PELP1 | 3.07E-14 |
| ENSSSCG00000017962 | KDM6B | 3.11E-14 |
| ENSSSCG00000010698 | FGFR2 | 3.26E-14 |
| ENSSSCG00000015375 | ITGB8 | 3.48E-14 |
| ENSSSCG00000017298 | TANC2 | 3.73E-14 |
| ENSSSCG00000006940 | CYR61 | 3.81E-14 |
| ENSSSCG00000009833 | SH2B3 | 4.64E-14 |
| ENSSSCG00000004050 | WTAP | 4.87E-14 |
| ENSSSCG00000037267 | MAX | 5.88E-14 |
| ENSSSCG00000023525 | TMEM26 | 7.16E-14 |
| ENSSSCG00000002004 | PSME2 | 7.69E-14 |
| ENSSSCG00000001984 | KHNYN | 7.99E-14 |
| ENSSSCG00000015801 | TLR3 | 8.8E-14 |
| ENSSSCG00000004058 | EZR | 9.22E-14 |
| ENSSSCG00000009881 | OAS2 | 9.47E-14 |
| ENSSSCG00000005211 | CD274 | 9.66E-14 |
| ENSSSCG00000003137 | PLEKHA4 | 9.84E-14 |
| ENSSSCG00000014171 | ERAP1 | 1.18E-13 |
| ENSSSCG00000028097 | NA | 1.31E-13 |
| ENSSSCG00000023972 | DRAM1 | 1.43E-13 |
| ENSSSCG00000032154 | ERF | 1.43E-13 |
| ENSSSCG00000031329 | ST8SIA1 | 1.46E-13 |
| ENSSSCG00000009638 | RHOBTB2 | 1.58E-13 |
| ENSSSCG00000040013 | MTUS1 | 1.82E-13 |
| ENSSSCG00000014136 | VCAN | 1.86E-13 |
| ENSSSCG00000032469 | NA | 1.87E-13 |
| ENSSSCG00000011628 | DNAJC13 | 2.2E-13 |
| ENSSSCG00000001394 | NA | 2.44E-13 |
| ENSSSCG00000006235 | TOX | 2.66E-13 |
| ENSSSCG00000009806 | SETD1B | 2.89E-13 |
| ENSSSCG00000022649 | SLC7A11 | 2.99E-13 |
| ENSSSCG00000039780 | RTN4RL1 | 3.14E-13 |
| ENSSSCG00000010795 | NA | 3.18E-13 |
| ENSSSCG00000011411 | TMEM115 | 3.24E-13 |
| ENSSSCG00000007465 | B4GALT5 | 3.25E-13 |
| ENSSSCG00000006543 | ADAR | 3.58E-13 |
| ENSSSCG00000004576 | RORA | 3.81E-13 |
| ENSSSCG00000005277 | GCNT1 | 4.09E-13 |
| ENSSSCG00000006776 | MOV10 | 4.14E-13 |
| ENSSSCG00000001398 | NA | 4.16E-13 |
| ENSSSCG00000010836 | BROX | 4.71E-13 |
| ENSSSCG00000023351 | PLA2G4A | 4.89E-13 |
| ENSSSCG00000026041 | MAP3K5 | 5.41E-13 |
| ENSSSCG00000002516 | WARS | 5.42E-13 |
| ENSSSCG00000009395 | SETDB2 | 5.42E-13 |
| ENSSSCG00000038220 | RXRA | 5.6E-13 |
| ENSSSCG00000016453 | TCAF1 | 6.85E-13 |
| ENSSSCG00000007586 | FSCN1 | 7.06E-13 |
| ENSSSCG00000001849 | ANPEP | 7.34E-13 |
| ENSSSCG00000035223 | SYNM | 7.58E-13 |
| ENSSSCG00000014565 | NA | 8.4E-13 |
| ENSSSCG00000017475 | RARA | 8.45E-13 |
| ENSSSCG00000008953 | CXCL8 | 9.84E-13 |
| ENSSSCG00000016262 | NA | 1.07E-12 |
| ENSSSCG00000003715 | NA | 1.08E-12 |
| ENSSSCG00000021361 | ZNF710 | 1.08E-12 |
| ENSSSCG00000025729 | IRS1 | 1.12E-12 |
| ENSSSCG00000002669 | CRISPLD2 | 1.16E-12 |
| ENSSSCG00000031262 | TXNIP | 1.33E-12 |
| ENSSSCG00000004687 | B2M | 1.4E-12 |
| ENSSSCG00000000148 | NA | 1.67E-12 |
| ENSSSCG00000009048 | GAB1 | 1.94E-12 |
| ENSSSCG00000001341 | NA | 2E-12 |
| ENSSSCG00000001620 | MDFI | 2.05E-12 |
| ENSSSCG00000023907 | AFAP1 | 2.13E-12 |
| ENSSSCG00000034216 | OGFR | 2.69E-12 |
| ENSSSCG00000006237 | SDCBP | 3.5E-12 |
| ENSSSCG00000021586 | ZHX2 | 3.76E-12 |
| ENSSSCG00000017416 | DHX58 | 4.17E-12 |
| ENSSSCG00000015550 | RGS16 | 4.27E-12 |
| ENSSSCG00000002350 | ELMSAN1 | 4.81E-12 |
| ENSSSCG00000030996 | NA | 5.27E-12 |
| ENSSSCG00000009505 | MBNL2 | 5.29E-12 |
| ENSSSCG00000009633 | NA | 5.35E-12 |
| ENSSSCG00000031337 | SNX18 | 5.79E-12 |
| ENSSSCG00000035051 | ADORA2B | 6.3E-12 |
| ENSSSCG00000005601 | HSPA5 | 6.96E-12 |
| ENSSSCG00000006830 | NA | 7.19E-12 |
| ENSSSCG00000026931 | SERTAD1 | 8.09E-12 |
| ENSSSCG00000017146 | RNF213 | 9.3E-12 |
| ENSSSCG00000027467 | DZIP1L | 9.63E-12 |
| ENSSSCG00000015871 | NR4A2 | 1E-11 |
| ENSSSCG00000040575 | ISG15 | 1.03E-11 |
| ENSSSCG00000037159 | MNT | 1.18E-11 |
| ENSSSCG00000031871 | NA | 1.19E-11 |
| ENSSSCG00000000837 | CHST11 | 1.33E-11 |
| ENSSSCG00000003419 | MAD2L2 | 1.36E-11 |
| ENSSSCG00000010181 | C1orf198 | 1.43E-11 |
| ENSSSCG00000039314 | MCL1 | 1.51E-11 |
| ENSSSCG00000010311 | CAMK2G | 1.58E-11 |
| ENSSSCG00000027894 | FAM76A | 1.61E-11 |
| ENSSSCG00000036437 | NOG | 1.65E-11 |
| ENSSSCG00000037066 | GADD45A | 1.65E-11 |
| ENSSSCG00000025598 | COBLL1 | 1.8E-11 |
| ENSSSCG00000007140 | SMOX | 2.02E-11 |
| ENSSSCG00000029230 | ECM1 | 2.08E-11 |
| ENSSSCG00000006919 | NA | 2.34E-11 |
| ENSSSCG00000035400 | YPEL2 | 2.63E-11 |
| ENSSSCG00000011727 | PTX3 | 3.11E-11 |
| ENSSSCG00000006733 | TTF2 | 3.11E-11 |
| ENSSSCG00000011474 | PXK | 3.11E-11 |
| ENSSSCG00000009434 | RGCC | 3.2E-11 |
| ENSSSCG00000005983 | ATAD2 | 3.29E-11 |
| ENSSSCG00000000728 | PARP11 | 3.54E-11 |
| ENSSSCG00000008437 | SOCS5 | 3.57E-11 |
| ENSSSCG00000030484 | AHR | 3.67E-11 |
| ENSSSCG00000016067 | STK17B | 3.76E-11 |
| ENSSSCG00000023165 | SEMA7A | 3.78E-11 |
| ENSSSCG00000021646 | KLF9 | 4.07E-11 |
| ENSSSCG00000036340 | ZBTB5 | 4.33E-11 |
| ENSSSCG00000023408 | SAMD4A | 4.46E-11 |
| ENSSSCG00000013551 | C3 | 5.01E-11 |
| ENSSSCG00000003702 | GATA6 | 5.89E-11 |
| ENSSSCG00000008334 | MXD1 | 6.21E-11 |
| ENSSSCG00000006862 | VCAM1 | 7.47E-11 |
| ENSSSCG00000011074 | ARHGAP21 | 7.63E-11 |
| ENSSSCG00000015784 | ACSL1 | 7.88E-11 |
| ENSSSCG00000017274 | PITPNC1 | 8.75E-11 |
| ENSSSCG00000003805 | PDE4B | 8.92E-11 |
| ENSSSCG00000027869 | PHF13 | 9.04E-11 |
| ENSSSCG00000015820 | NSD3 | 9.52E-11 |
| ENSSSCG00000017877 | ANKFY1 | 9.67E-11 |
| ENSSSCG00000008275 | TTC31 | 1E-10 |
| ENSSSCG00000029430 | IPO13 | 1.01E-10 |
| ENSSSCG00000017301 | TLK2 | 1.05E-10 |
| ENSSSCG00000035895 | JDP2 | 1.05E-10 |
| ENSSSCG00000007477 | NFATC2 | 1.09E-10 |
| ENSSSCG00000017391 | PLEKHH3 | 1.12E-10 |
| ENSSSCG00000021359 | CDC42EP3 | 1.15E-10 |
| ENSSSCG00000003613 | NA | 1.23E-10 |
| ENSSSCG00000010438 | ATAD1 | 1.25E-10 |
| ENSSSCG00000026710 | CARHSP1 | 1.29E-10 |
| ENSSSCG00000012027 | ADAMTS5 | 1.33E-10 |
| ENSSSCG00000026552 | MFSD14B | 1.41E-10 |
| ENSSSCG00000002470 | DDX24 | 1.41E-10 |
| ENSSSCG00000034879 | MAML2 | 1.64E-10 |
| ENSSSCG00000006874 | PALMD | 1.65E-10 |
| ENSSSCG00000010118 | HIRA | 1.8E-10 |
| ENSSSCG00000010017 | SMTN | 1.87E-10 |
| ENSSSCG00000021750 | MEX3D | 2.02E-10 |
| ENSSSCG00000005215 | JAK2 | 2.15E-10 |
| ENSSSCG00000029594 | WBP1L | 2.15E-10 |
| ENSSSCG00000029763 | IFI35 | 2.16E-10 |
| ENSSSCG00000010235 | SIRT1 | 2.4E-10 |
| ENSSSCG00000033113 | CHMP7 | 2.49E-10 |
| ENSSSCG00000002003 | NA | 2.65E-10 |
| ENSSSCG00000040617 | TNFAIP8 | 2.65E-10 |
| ENSSSCG00000007554 | ZFAND2A | 2.82E-10 |
| ENSSSCG00000017886 | FBXO39 | 2.89E-10 |
| ENSSSCG00000032835 | TMEM268 | 3.4E-10 |
| ENSSSCG00000034697 | NA | 3.44E-10 |
| ENSSSCG00000011825 | ATP13A3 | 3.83E-10 |
| ENSSSCG00000016725 | NA | 3.88E-10 |
| ENSSSCG00000008553 | PREB | 3.98E-10 |
| ENSSSCG00000035059 | MCM10 | 4.12E-10 |
| ENSSSCG00000031970 | RASSF5 | 4.24E-10 |
| ENSSSCG00000007355 | NA | 4.38E-10 |
| ENSSSCG00000028509 | RBM8A | 4.68E-10 |
| ENSSSCG00000025286 | MCTP1 | 4.72E-10 |
| ENSSSCG00000035069 | CHSY3 | 4.79E-10 |
| ENSSSCG00000031023 | NA | 5.39E-10 |
| ENSSSCG00000001507 | TAPBP | 5.47E-10 |
| ENSSSCG00000040786 | SPTBN1 | 5.51E-10 |
| ENSSSCG00000000810 | AMIGO2 | 5.6E-10 |
| ENSSSCG00000005533 | PTGS1 | 5.6E-10 |
| ENSSSCG00000035240 | GPR63 | 5.74E-10 |
| ENSSSCG00000004547 | NA | 5.79E-10 |
| ENSSSCG00000025856 | TMEM106A | 5.79E-10 |
| ENSSSCG00000038492 | FAM109B | 6.16E-10 |
| ENSSSCG00000032015 | SH3BGRL2 | 6.33E-10 |
| ENSSSCG00000035774 | ERRFI1 | 6.56E-10 |
| ENSSSCG00000001951 | PSMA6 | 7.45E-10 |
| ENSSSCG00000017607 | TMEM100 | 7.74E-10 |
| ENSSSCG00000021761 | UBAP1 | 7.8E-10 |
| ENSSSCG00000015960 | MAP3K20 | 8.5E-10 |
| ENSSSCG00000025114 | FMNL3 | 8.5E-10 |
| ENSSSCG00000038854 | PSMF1 | 8.55E-10 |
| ENSSSCG00000040355 | TICRR | 9.04E-10 |
| ENSSSCG00000009101 | PRDM5 | 9.69E-10 |
| ENSSSCG00000011212 | RARB | 1.11E-09 |
| ENSSSCG00000011020 | JCAD | 1.12E-09 |
| ENSSSCG00000021712 | HERC6 | 1.35E-09 |
| ENSSSCG00000011317 | FYCO1 | 1.36E-09 |
| ENSSSCG00000013236 | MYBPC3 | 1.41E-09 |
| ENSSSCG00000034321 | TP53INP1 | 1.54E-09 |
| ENSSSCG00000011430 | DUSP7 | 1.85E-09 |
| ENSSSCG00000002135 | PNP | 1.85E-09 |
| ENSSSCG00000000600 | EPS8 | 1.89E-09 |
| ENSSSCG00000001561 | ETV7 | 1.96E-09 |
| ENSSSCG00000014997 | NA | 2.05E-09 |
| ENSSSCG00000004142 | CITED2 | 2.06E-09 |
| ENSSSCG00000028529 | REM1 | 2.18E-09 |
| ENSSSCG00000010219 | ARID5B | 2.23E-09 |
| ENSSSCG00000017403 | STAT3 | 2.42E-09 |
| ENSSSCG00000014204 | DCP2 | 2.42E-09 |
| ENSSSCG00000010600 | CALHM2 | 2.42E-09 |
| ENSSSCG00000009071 | JADE1 | 2.48E-09 |
| ENSSSCG00000034973 | CXCL12 | 2.55E-09 |
| ENSSSCG00000011463 | IL17RD | 2.56E-09 |
| ENSSSCG00000004974 | LARP6 | 2.57E-09 |
| ENSSSCG00000022478 | STK10 | 2.74E-09 |
| ENSSSCG00000004065 | TIAM2 | 2.77E-09 |
| ENSSSCG00000002375 | RPS6KL1 | 3.06E-09 |
| ENSSSCG00000015563 | RGL1 | 3.12E-09 |
| ENSSSCG00000030005 | LGALSL | 3.12E-09 |
| ENSSSCG00000040648 | CCL11 | 3.33E-09 |
| ENSSSCG00000035058 | PID1 | 3.62E-09 |
| ENSSSCG00000011876 | DTX3L | 3.71E-09 |
| ENSSSCG00000012828 | STARD8 | 3.81E-09 |
| ENSSSCG00000035952 | TGFB1I1 | 4.08E-09 |
| ENSSSCG00000029347 | NA | 4.14E-09 |
| ENSSSCG00000039616 | ZBTB25 | 5.16E-09 |
| ENSSSCG00000003644 | FHL3 | 5.17E-09 |
| ENSSSCG00000008641 | ADAM17 | 5.39E-09 |
| ENSSSCG00000010464 | PPP1R3C | 5.7E-09 |
| ENSSSCG00000032715 | CERS6 | 6.03E-09 |
| ENSSSCG00000036063 | LPAR6 | 6.17E-09 |
| ENSSSCG00000037670 | TMEM164 | 6.57E-09 |
| ENSSSCG00000009192 | PDLIM5 | 6.96E-09 |
| ENSSSCG00000005235 | KANK1 | 6.96E-09 |
| ENSSSCG00000016119 | NA | 6.96E-09 |
| ENSSSCG00000016656 | ELMO1 | 6.97E-09 |
| ENSSSCG00000017066 | GEMIN5 | 7.22E-09 |
| ENSSSCG00000016243 | RHBDD1 | 7.24E-09 |
| ENSSSCG00000000843 | TXNRD1 | 7.41E-09 |
| ENSSSCG00000003590 | PTPRU | 7.91E-09 |
| ENSSSCG00000003718 | TAF4B | 8.16E-09 |
| ENSSSCG00000008197 | SEMA4C | 8.27E-09 |
| ENSSSCG00000011437 | ALAS1 | 8.73E-09 |
| ENSSSCG00000015645 | NA | 9.15E-09 |
| ENSSSCG00000008980 | SCARB2 | 9.55E-09 |
| ENSSSCG00000012713 | ATP11C | 9.71E-09 |
| ENSSSCG00000006857 | COL11A1 | 9.92E-09 |
| ENSSSCG00000011950 | NXPE3 | 1.06E-08 |
| ENSSSCG00000008562 | SLC35F6 | 1.06E-08 |
| ENSSSCG00000027266 | PNPLA3 | 1.16E-08 |
| ENSSSCG00000005506 | MEGF9 | 1.25E-08 |
| ENSSSCG00000039756 | FOXC1 | 1.31E-08 |
| ENSSSCG00000011670 | PXYLP1 | 1.38E-08 |
| ENSSSCG00000011106 | CREM | 1.4E-08 |
| ENSSSCG00000025689 | DVL2 | 1.41E-08 |
| ENSSSCG00000026940 | CASP10 | 1.44E-08 |
| ENSSSCG00000004218 | RSPO3 | 1.44E-08 |
| ENSSSCG00000028117 | NA | 1.45E-08 |
| ENSSSCG00000029030 | SMCHD1 | 1.51E-08 |
| ENSSSCG00000000263 | TNS2 | 1.56E-08 |
| ENSSSCG00000015324 | GNG11 | 1.59E-08 |
| ENSSSCG00000005267 | ANXA1 | 1.8E-08 |
| ENSSSCG00000029002 | PNKD | 1.83E-08 |
| ENSSSCG00000004700 | PDIA3 | 1.89E-08 |
| ENSSSCG00000003000 | ITPKC | 1.99E-08 |
| ENSSSCG00000039555 | JOSD1 | 2.03E-08 |
| ENSSSCG00000014920 | FZD4 | 2.09E-08 |
| ENSSSCG00000017804 | ABR | 2.09E-08 |
| ENSSSCG00000016018 | FRZB | 2.15E-08 |
| ENSSSCG00000021814 | ZMYM2 | 2.19E-08 |
| ENSSSCG00000027855 | SOCS1 | 2.31E-08 |
| ENSSSCG00000024793 | PORCN | 2.43E-08 |
| ENSSSCG00000024166 | SLC2A6 | 2.65E-08 |
| ENSSSCG00000001070 | FAM8A1 | 2.65E-08 |
| ENSSSCG00000040037 | MTSS1L | 3.07E-08 |
| ENSSSCG00000009018 | SH3D19 | 3.28E-08 |
| ENSSSCG00000023784 | SEMA3C | 3.66E-08 |
| ENSSSCG00000013735 | JUNB | 3.74E-08 |
| ENSSSCG00000003253 | NA | 3.92E-08 |
| ENSSSCG00000005738 | RALGDS | 4.03E-08 |
| ENSSSCG00000004919 | NEDD4L | 4.15E-08 |
| ENSSSCG00000011538 | LMCD1 | 4.19E-08 |
| ENSSSCG00000004952 | SMAD3 | 4.22E-08 |
| ENSSSCG00000016002 | NA | 4.32E-08 |
| ENSSSCG00000038401 | TRAM2 | 4.32E-08 |
| ENSSSCG00000006651 | ADAMTSL4 | 4.64E-08 |
| ENSSSCG00000009172 | PPP3CA | 4.83E-08 |
| ENSSSCG00000015113 | NLRX1 | 4.85E-08 |
| ENSSSCG00000006625 | RFX5 | 4.89E-08 |
| ENSSSCG00000033509 | SAMD11 | 5.31E-08 |
| ENSSSCG00000001554 | SRPK1 | 5.39E-08 |
| ENSSSCG00000000769 | BID | 5.43E-08 |
| ENSSSCG00000011611 | HMCES | 5.46E-08 |
| ENSSSCG00000001518 | ITPR3 | 5.46E-08 |
| ENSSSCG00000006482 | MEF2D | 6.47E-08 |
| ENSSSCG00000021822 | RNF169 | 6.76E-08 |
| ENSSSCG00000027124 | NA | 7.01E-08 |
| ENSSSCG00000006173 | GDAP1 | 7.06E-08 |
| ENSSSCG00000006194 | NCOA2 | 7.16E-08 |
| ENSSSCG00000023419 | ARHGEF10 | 7.53E-08 |
| ENSSSCG00000038867 | PPM1K | 7.66E-08 |
| ENSSSCG00000010329 | ZMIZ1 | 7.9E-08 |
| ENSSSCG00000015595 | ATF3 | 8.01E-08 |
| ENSSSCG00000016233 | SERPINE2 | 8.16E-08 |
| ENSSSCG00000026636 | ZBTB4 | 8.59E-08 |
| ENSSSCG00000024914 | NA | 8.75E-08 |
| ENSSSCG00000011672 | RASA2 | 9.55E-08 |
| ENSSSCG00000021885 | MDFIC | 9.77E-08 |
| ENSSSCG00000008422 | NA | 9.89E-08 |
| ENSSSCG00000011775 | KLHL24 | 9.92E-08 |
| ENSSSCG00000022714 | OSER1 | 9.92E-08 |
| ENSSSCG00000012591 | AMOT | 1.03E-07 |
| ENSSSCG00000032632 | CMTM4 | 1.03E-07 |
| ENSSSCG00000034012 | CASP3 | 1.05E-07 |
| ENSSSCG00000036155 | FAT4 | 1.06E-07 |
| ENSSSCG00000011567 | NA | 1.12E-07 |
| ENSSSCG00000014303 | JADE2 | 1.14E-07 |
| ENSSSCG00000022895 | CRYBG3 | 1.18E-07 |
| ENSSSCG00000009338 | FRY | 1.19E-07 |
| ENSSSCG00000023229 | ETV5 | 1.24E-07 |
| ENSSSCG00000004963 | NA | 1.28E-07 |
| ENSSSCG00000034616 | LRRC58 | 1.29E-07 |
| ENSSSCG00000010995 | CHMP5 | 1.29E-07 |
| ENSSSCG00000025343 | ZNF628 | 1.31E-07 |
| ENSSSCG00000036403 | FAM180A | 1.34E-07 |
| ENSSSCG00000032164 | PEA15 | 1.37E-07 |
| ENSSSCG00000023362 | RHBDF2 | 1.42E-07 |
| ENSSSCG00000009676 | ZNF395 | 1.44E-07 |
| ENSSSCG00000032115 | OSGIN1 | 1.47E-07 |
| ENSSSCG00000007237 | PDRG1 | 1.5E-07 |
| ENSSSCG00000016916 | IL6ST | 1.53E-07 |
| ENSSSCG00000011936 | ZBED2 | 1.54E-07 |
| ENSSSCG00000000457 | USP15 | 1.54E-07 |
| ENSSSCG00000013766 | IL27RA | 1.56E-07 |
| ENSSSCG00000038460 | FOXF1 | 1.61E-07 |
| ENSSSCG00000008446 | SIX2 | 1.76E-07 |
| ENSSSCG00000017178 | SPHK1 | 1.78E-07 |
| ENSSSCG00000002852 | PLEKHF1 | 1.78E-07 |
| ENSSSCG00000008873 | FAM198B | 1.78E-07 |
| ENSSSCG00000022492 | AMPD3 | 1.87E-07 |
| ENSSSCG00000036383 | LGALS3BP | 1.98E-07 |
| ENSSSCG00000039053 | VGF | 2.06E-07 |
| ENSSSCG00000012112 | ARHGAP6 | 2.17E-07 |
| ENSSSCG00000031781 | PSMB10 | 2.18E-07 |
| ENSSSCG00000039194 | KANK2 | 2.26E-07 |
| ENSSSCG00000017201 | UNK | 2.3E-07 |
| ENSSSCG00000013382 | PLEKHA7 | 2.65E-07 |
| ENSSSCG00000010172 | EGLN1 | 2.7E-07 |
| ENSSSCG00000004082 | NA | 2.77E-07 |
| ENSSSCG00000023618 | FRMD7 | 2.81E-07 |
| ENSSSCG00000031789 | ACSL5 | 2.86E-07 |
| ENSSSCG00000035969 | THRA | 2.91E-07 |
| ENSSSCG00000027480 | KLF10 | 2.92E-07 |
| ENSSSCG00000007864 | GPRC5B | 2.93E-07 |
| ENSSSCG00000001471 | BRD2 | 2.94E-07 |
| ENSSSCG00000017705 | CCL5 | 2.97E-07 |
| ENSSSCG00000033001 | FZD8 | 3.05E-07 |
| ENSSSCG00000033453 | BST2 | 3.13E-07 |
| ENSSSCG00000015370 | TWIST1 | 3.34E-07 |
| ENSSSCG00000036206 | C3orf58 | 3.39E-07 |
| ENSSSCG00000028293 | RCOR3 | 3.68E-07 |
| ENSSSCG00000008647 | CMPK2 | 3.73E-07 |
| ENSSSCG00000039812 | RAP2A | 3.88E-07 |
| ENSSSCG00000029380 | ZFP2 | 3.89E-07 |
| ENSSSCG00000036639 | STOM | 3.92E-07 |
| ENSSSCG00000024592 | ANKRD17 | 4.16E-07 |
| ENSSSCG00000032475 | CEP44 | 4.2E-07 |
| ENSSSCG00000028345 | NA | 4.56E-07 |
| ENSSSCG00000032170 | DAPK3 | 4.71E-07 |
| ENSSSCG00000031627 | WBP4 | 4.72E-07 |
| ENSSSCG00000002716 | MLKL | 4.76E-07 |
| ENSSSCG00000030560 | NA | 4.86E-07 |
| ENSSSCG00000035790 | BTG1 | 4.99E-07 |
| ENSSSCG00000015944 | TLK1 | 5.21E-07 |
| ENSSSCG00000024373 | TRIP12 | 5.24E-07 |
| ENSSSCG00000002311 | SUSD6 | 5.59E-07 |
| ENSSSCG00000009943 | SSH1 | 5.65E-07 |
| ENSSSCG00000040773 | TOB1 | 5.65E-07 |
| ENSSSCG00000031924 | NKX3-1 | 5.86E-07 |
| ENSSSCG00000015541 | IER5 | 5.88E-07 |
| ENSSSCG00000013048 | C11orf84 | 6.28E-07 |
| ENSSSCG00000013298 | PDHX | 6.4E-07 |
| ENSSSCG00000040725 | IL11 | 6.52E-07 |
| ENSSSCG00000017046 | EBF1 | 7.05E-07 |
| ENSSSCG00000013901 | IFI30 | 7.12E-07 |
| ENSSSCG00000008265 | DOK1 | 7.34E-07 |
| ENSSSCG00000006331 | PBX1 | 7.69E-07 |
| ENSSSCG00000025836 | SULT1C4 | 7.81E-07 |
| ENSSSCG00000002376 | PGF | 8.15E-07 |
| ENSSSCG00000006319 | POGK | 8.53E-07 |
| ENSSSCG00000040743 | M6PR | 8.83E-07 |
| ENSSSCG00000017102 | PAPD7 | 9.15E-07 |
| ENSSSCG00000033830 | CCDC8 | 9.24E-07 |
| ENSSSCG00000001787 | IL16 | 9.33E-07 |
| ENSSSCG00000007576 | FOXK1 | 9.33E-07 |
| ENSSSCG00000005027 | FRMD6 | 9.38E-07 |
| ENSSSCG00000013722 | WDR83 | 9.38E-07 |
| ENSSSCG00000009652 | KCTD9 | 9.42E-07 |
| ENSSSCG00000001700 | SLC29A1 | 9.44E-07 |
| ENSSSCG00000029998 | KLF7 | 9.6E-07 |
| ENSSSCG00000035940 | SPSB1 | 9.6E-07 |
| ENSSSCG00000038912 | IFITM3 | 9.63E-07 |
| ENSSSCG00000011026 | ARHGAP12 | 9.8E-07 |
| ENSSSCG00000035037 | NA | 1.04E-06 |
| ENSSSCG00000036083 | NA | 1.07E-06 |
| ENSSSCG00000026196 | NA | 1.08E-06 |
| ENSSSCG00000040139 | B3GNT9 | 1.12E-06 |
| ENSSSCG00000013181 | SERPING1 | 1.18E-06 |
| ENSSSCG00000033787 | NA | 1.25E-06 |
| ENSSSCG00000016217 | DNAJB2 | 1.27E-06 |
| ENSSSCG00000001835 | ABHD2 | 1.32E-06 |
| ENSSSCG00000031147 | ACAP2 | 1.35E-06 |
| ENSSSCG00000006345 | OLFML2B | 1.36E-06 |
| ENSSSCG00000013397 | ARNTL | 1.42E-06 |
| ENSSSCG00000017957 | EIF4A1 | 1.43E-06 |
| ENSSSCG00000038141 | TICAM2 | 1.44E-06 |
| ENSSSCG00000001061 | JARID2 | 1.45E-06 |
| ENSSSCG00000034984 | PSMB4 | 1.48E-06 |
| ENSSSCG00000003167 | FLT3LG | 1.49E-06 |
| ENSSSCG00000009959 | ASPHD2 | 1.57E-06 |
| ENSSSCG00000010574 | LDB1 | 1.6E-06 |
| ENSSSCG00000005012 | ARF6 | 1.61E-06 |
| ENSSSCG00000011493 | ATXN7 | 1.62E-06 |
| ENSSSCG00000015011 | DDX10 | 1.62E-06 |
| ENSSSCG00000017251 | SOX9 | 1.63E-06 |
| ENSSSCG00000032749 | PCDH18 | 1.84E-06 |
| ENSSSCG00000001011 | SERPINB1 | 1.89E-06 |
| ENSSSCG00000009868 | RBM19 | 1.89E-06 |
| ENSSSCG00000036160 | ZNF250 | 2.06E-06 |
| ENSSSCG00000038958 | DNM3 | 2.07E-06 |
| ENSSSCG00000038469 | NA | 2.12E-06 |
| ENSSSCG00000014540 | NA | 2.13E-06 |
| ENSSSCG00000002429 | FOXN3 | 2.14E-06 |
| ENSSSCG00000014219 | CDO1 | 2.24E-06 |
| ENSSSCG00000013400 | MICAL2 | 2.3E-06 |
| ENSSSCG00000012654 | NA | 2.32E-06 |
| ENSSSCG00000022159 | FNDC3A | 2.41E-06 |
| ENSSSCG00000008981 | NA | 2.44E-06 |
| ENSSSCG00000001975 | PRKD1 | 2.48E-06 |
| ENSSSCG00000030015 | NA | 2.72E-06 |
| ENSSSCG00000007808 | NFATC2IP | 2.73E-06 |
| ENSSSCG00000036746 | RASL10B | 2.74E-06 |
| ENSSSCG00000024614 | ITSN1 | 2.74E-06 |
| ENSSSCG00000040989 | GPRC5C | 2.87E-06 |
| ENSSSCG00000013554 | TRIP10 | 2.87E-06 |
| ENSSSCG00000031610 | NA | 2.94E-06 |
| ENSSSCG00000007395 | NA | 2.94E-06 |
| ENSSSCG00000029805 | RHOBTB3 | 2.95E-06 |
| ENSSSCG00000017488 | CSF3 | 3.11E-06 |
| ENSSSCG00000009050 | INPP4B | 3.12E-06 |
| ENSSSCG00000017754 | NA | 3.16E-06 |
| ENSSSCG00000014339 | CTNNA1 | 3.3E-06 |
| ENSSSCG00000003197 | MED25 | 3.33E-06 |
| ENSSSCG00000036634 | MAP4K5 | 3.53E-06 |
| ENSSSCG00000015589 | VASH2 | 3.63E-06 |
| ENSSSCG00000003729 | RNF125 | 3.77E-06 |
| ENSSSCG00000021874 | UNC5C | 4E-06 |
| ENSSSCG00000029708 | SLC25A38 | 4.1E-06 |
| ENSSSCG00000008727 | MSX1 | 4.1E-06 |
| ENSSSCG00000029174 | CCDC126 | 4.16E-06 |
| ENSSSCG00000015826 | NA | 4.19E-06 |
| ENSSSCG00000004747 | RTF1 | 4.51E-06 |
| ENSSSCG00000017402 | STAT5A | 4.51E-06 |
| ENSSSCG00000009716 | SH3RF1 | 4.65E-06 |
| ENSSSCG00000017993 | NTN1 | 4.66E-06 |
| ENSSSCG00000005314 | ARHGEF39 | 4.7E-06 |
| ENSSSCG00000017584 | PPP1R9B | 4.72E-06 |
| ENSSSCG00000004387 | FOXO3 | 4.72E-06 |
| ENSSSCG00000037898 | CXXC5 | 4.81E-06 |
| ENSSSCG00000034259 | PMEPA1 | 4.86E-06 |
| ENSSSCG00000005358 | NA | 4.94E-06 |
| ENSSSCG00000005041 | FERMT2 | 5.02E-06 |
| ENSSSCG00000006917 | NA | 5.19E-06 |
| ENSSSCG00000006729 | FAM46C | 5.19E-06 |
| ENSSSCG00000000737 | TULP3 | 5.26E-06 |
| ENSSSCG00000006073 | OSR2 | 5.36E-06 |
| ENSSSCG00000013658 | S1PR2 | 5.36E-06 |
| ENSSSCG00000024385 | RNASEH2B | 5.38E-06 |
| ENSSSCG00000013391 | NA | 5.39E-06 |
| ENSSSCG00000007737 | TPST1 | 5.39E-06 |
| ENSSSCG00000030581 | VGLL4 | 5.46E-06 |
| ENSSSCG00000009630 | EGR3 | 5.52E-06 |
| ENSSSCG00000014338 | HSPA9 | 5.53E-06 |
| ENSSSCG00000002252 | ARRDC4 | 5.56E-06 |
| ENSSSCG00000011683 | PCOLCE2 | 5.78E-06 |
| ENSSSCG00000015238 | ARHGAP32 | 5.86E-06 |
| ENSSSCG00000027660 | IFI44L | 5.86E-06 |
| ENSSSCG00000023126 | NA | 5.91E-06 |
| ENSSSCG00000001532 | UHRF1BP1 | 5.91E-06 |
| ENSSSCG00000009720 | DDX60 | 5.91E-06 |
| ENSSSCG00000008043 | TRAF7 | 6.03E-06 |
| ENSSSCG00000040663 | HERPUD1 | 6.17E-06 |
| ENSSSCG00000007135 | NA | 6.19E-06 |
| ENSSSCG00000004825 | CHSY1 | 6.22E-06 |
| ENSSSCG00000011298 | CDCP1 | 6.24E-06 |
| ENSSSCG00000031706 | MICALCL | 6.28E-06 |
| ENSSSCG00000017920 | NA | 6.66E-06 |
| ENSSSCG00000007559 | MAFK | 6.79E-06 |
| ENSSSCG00000002795 | CDH11 | 6.79E-06 |
| ENSSSCG00000039094 | PLEC | 6.79E-06 |
| ENSSSCG00000014820 | FCHSD2 | 6.94E-06 |
| ENSSSCG00000039348 | H1F0 | 7.27E-06 |
| ENSSSCG00000009357 | SMAD9 | 7.35E-06 |
| ENSSSCG00000006169 | ZFHX4 | 7.55E-06 |
| ENSSSCG00000008601 | SDC1 | 7.66E-06 |
| ENSSSCG00000006453 | KIRREL1 | 7.7E-06 |
| ENSSSCG00000008170 | NA | 7.79E-06 |
| ENSSSCG00000026617 | EIF2S2 | 8.3E-06 |
| ENSSSCG00000033626 | SREBF1 | 8.36E-06 |
| ENSSSCG00000015965 | GPR155 | 8.58E-06 |
| ENSSSCG00000025156 | BRWD3 | 8.66E-06 |
| ENSSSCG00000015961 | CDCA7 | 8.7E-06 |
| ENSSSCG00000011022 | SVIL | 8.92E-06 |
| ENSSSCG00000005981 | FBXO32 | 8.98E-06 |
| ENSSSCG00000011933 | NECTIN3 | 9.08E-06 |
| ENSSSCG00000004109 | ZC3H12D | 9.08E-06 |
| ENSSSCG00000028878 | BCAR1 | 9.24E-06 |
| ENSSSCG00000004558 | CSNK1G1 | 9.37E-06 |
| ENSSSCG00000013664 | C19orf66 | 9.37E-06 |
| ENSSSCG00000011047 | FAM171A1 | 9.37E-06 |
| ENSSSCG00000003707 | NPC1 | 9.55E-06 |
| ENSSSCG00000017890 | KIAA0753 | 9.63E-06 |
| ENSSSCG00000003655 | NA | 9.68E-06 |
| ENSSSCG00000035648 | MANEA | 9.75E-06 |
| ENSSSCG00000013278 | TSPAN18 | 9.76E-06 |
| ENSSSCG00000016922 | GPBP1 | 1.02E-05 |
| ENSSSCG00000012074 | NA | 1.03E-05 |
| ENSSSCG00000022048 | REEP5 | 1.07E-05 |
| ENSSSCG00000008230 | ATOH8 | 1.09E-05 |
| ENSSSCG00000026904 | NFKBIB | 1.1E-05 |
| ENSSSCG00000002026 | EFS | 1.1E-05 |
| ENSSSCG00000011353 | PFKFB4 | 1.13E-05 |
| ENSSSCG00000007206 | RBCK1 | 1.13E-05 |
| ENSSSCG00000004507 | SMAD7 | 1.15E-05 |
| ENSSSCG00000034378 | IFNGR2 | 1.22E-05 |
| ENSSSCG00000028304 | ZFP36L1 | 1.22E-05 |
| ENSSSCG00000006742 | MAB21L3 | 1.22E-05 |
| ENSSSCG00000025344 | PHC2 | 1.22E-05 |
| ENSSSCG00000039419 | SLCO4A1 | 1.23E-05 |
| ENSSSCG00000010169 | SIPA1L2 | 1.32E-05 |
| ENSSSCG00000028454 | C16orf72 | 1.32E-05 |
| ENSSSCG00000010638 | TCF7L2 | 1.34E-05 |
| ENSSSCG00000034465 | LRP10 | 1.34E-05 |
| ENSSSCG00000035987 | EHD3 | 1.35E-05 |
| ENSSSCG00000011973 | COL8A1 | 1.35E-05 |
| ENSSSCG00000020856 | PPME1 | 1.39E-05 |
| ENSSSCG00000027426 | BCL3 | 1.42E-05 |
| ENSSSCG00000003580 | EYA3 | 1.42E-05 |
| ENSSSCG00000025806 | FAM118B | 1.49E-05 |
| ENSSSCG00000023653 | GLIS2 | 1.51E-05 |
| ENSSSCG00000011471 | FLNB | 1.53E-05 |
| ENSSSCG00000014672 | NA | 1.55E-05 |
| ENSSSCG00000025499 | NA | 1.6E-05 |
| ENSSSCG00000007454 | NA | 1.62E-05 |
| ENSSSCG00000005301 | FAM214B | 1.62E-05 |
| ENSSSCG00000003153 | FTL | 1.63E-05 |
| ENSSSCG00000000455 | LRIG3 | 1.69E-05 |
| ENSSSCG00000008468 | PKDCC | 1.77E-05 |
| ENSSSCG00000015227 | SRPRA | 1.78E-05 |
| ENSSSCG00000034347 | TRIM56 | 1.83E-05 |
| ENSSSCG00000012893 | UNC93B1 | 1.83E-05 |
| ENSSSCG00000003107 | ARHGAP35 | 1.86E-05 |
| ENSSSCG00000031657 | NA | 1.87E-05 |
| ENSSSCG00000012504 | NAP1L3 | 1.88E-05 |
| ENSSSCG00000017904 | ENO3 | 1.89E-05 |
| ENSSSCG00000028855 | GMPS | 1.9E-05 |
| ENSSSCG00000007486 | CYP24A1 | 1.92E-05 |
| ENSSSCG00000008772 | RELL1 | 2.04E-05 |
| ENSSSCG00000010053 | NA | 2.06E-05 |
| ENSSSCG00000014336 | EGR1 | 2.09E-05 |
| ENSSSCG00000037241 | RGS2 | 2.15E-05 |
| ENSSSCG00000007112 | PAX1 | 2.21E-05 |
| ENSSSCG00000033272 | DNAJB6 | 2.21E-05 |
| ENSSSCG00000013244 | PACSIN3 | 2.24E-05 |
| ENSSSCG00000010222 | ZNF365 | 2.25E-05 |
| ENSSSCG00000022401 | AGTRAP | 2.4E-05 |
| ENSSSCG00000021731 | WWC2 | 2.4E-05 |
| ENSSSCG00000009834 | ATXN2 | 2.45E-05 |
| ENSSSCG00000007659 | ZCWPW1 | 2.5E-05 |
| ENSSSCG00000012322 | KDM5C | 2.55E-05 |
| ENSSSCG00000020858 | KIF13A | 2.55E-05 |
| ENSSSCG00000011389 | AMIGO3 | 2.59E-05 |
| ENSSSCG00000012110 | MID1 | 2.71E-05 |
| ENSSSCG00000037583 | GNB4 | 2.71E-05 |
| ENSSSCG00000026082 | DNAJC3 | 2.71E-05 |
| ENSSSCG00000021440 | GPSM2 | 2.73E-05 |
| ENSSSCG00000039731 | NA | 2.75E-05 |
| ENSSSCG00000012479 | PCDH19 | 2.79E-05 |
| ENSSSCG00000002344 | RIOX1 | 2.79E-05 |
| ENSSSCG00000005232 | SMARCA2 | 2.87E-05 |
| ENSSSCG00000001009 | RIPK1 | 2.89E-05 |
| ENSSSCG00000017497 | ERBB2 | 2.98E-05 |
| ENSSSCG00000038940 | GNPDA1 | 3.03E-05 |
| ENSSSCG00000027378 | SH3PXD2B | 3.06E-05 |
| ENSSSCG00000029168 | MARF1 | 3.06E-05 |
| ENSSSCG00000025561 | VASN | 3.06E-05 |
| ENSSSCG00000037238 | NA | 3.06E-05 |
| ENSSSCG00000040731 | TMEM65 | 3.06E-05 |
| ENSSSCG00000008963 | AREG | 3.07E-05 |
| ENSSSCG00000037719 | PDPK1 | 3.14E-05 |
| ENSSSCG00000000633 | YBX3 | 3.15E-05 |
| ENSSSCG00000017258 | FAM20A | 3.19E-05 |
| ENSSSCG00000016824 | RAI14 | 3.19E-05 |
| ENSSSCG00000007675 | EPHB4 | 3.21E-05 |
| ENSSSCG00000016263 | NA | 3.22E-05 |
| ENSSSCG00000028327 | RHOBTB1 | 3.26E-05 |
| ENSSSCG00000007812 | XPO6 | 3.29E-05 |
| ENSSSCG00000030857 | LENG8 | 3.3E-05 |
| ENSSSCG00000007878 | PARN | 3.31E-05 |
| ENSSSCG00000001549 | FKBP5 | 3.39E-05 |
| ENSSSCG00000034379 | MAP2K3 | 3.43E-05 |
| ENSSSCG00000000773 | TUBA8 | 3.43E-05 |
| ENSSSCG00000011342 | DHX30 | 3.44E-05 |
| ENSSSCG00000008574 | KIF3C | 3.45E-05 |
| ENSSSCG00000002259 | NA | 3.46E-05 |
| ENSSSCG00000009122 | ARSJ | 3.49E-05 |
| ENSSSCG00000005657 | PKN3 | 3.5E-05 |
| ENSSSCG00000009664 | PTK2B | 3.53E-05 |
| ENSSSCG00000010016 | MORC2 | 3.77E-05 |
| ENSSSCG00000023973 | NOC2L | 3.85E-05 |
| ENSSSCG00000033641 | COL8A2 | 3.85E-05 |
| ENSSSCG00000035971 | DUSP2 | 4.02E-05 |
| ENSSSCG00000005229 | VLDLR | 4.03E-05 |
| ENSSSCG00000031492 | PPP1R18 | 4.04E-05 |
| ENSSSCG00000004989 | FBXO33 | 4.04E-05 |
| ENSSSCG00000006187 | MSC | 4.06E-05 |
| ENSSSCG00000037674 | NA | 4.11E-05 |
| ENSSSCG00000011193 | BTD | 4.28E-05 |
| ENSSSCG00000006740 | ATP1A1 | 4.33E-05 |
| ENSSSCG00000002039 | MMP14 | 4.35E-05 |
| ENSSSCG00000023264 | NA | 4.45E-05 |
| ENSSSCG00000021598 | EVA1C | 4.49E-05 |
| ENSSSCG00000023724 | ZBTB1 | 4.5E-05 |
| ENSSSCG00000004948 | SMAD6 | 4.5E-05 |
| ENSSSCG00000040673 | TMEM140 | 4.51E-05 |
| ENSSSCG00000010211 | CCDC6 | 4.54E-05 |
| ENSSSCG00000011000 | DNAJA1 | 4.6E-05 |
| ENSSSCG00000030801 | NA | 4.65E-05 |
| ENSSSCG00000024623 | USP25 | 4.67E-05 |
| ENSSSCG00000026516 | EPHB3 | 4.69E-05 |
| ENSSSCG00000017414 | KAT2A | 4.69E-05 |
| ENSSSCG00000000708 | TNFRSF1A | 4.78E-05 |
| ENSSSCG00000016983 | STC2 | 4.82E-05 |
| ENSSSCG00000014395 | PCDH12 | 4.96E-05 |
| ENSSSCG00000029652 | TMBIM6 | 4.96E-05 |
| ENSSSCG00000005437 | KLF4 | 4.98E-05 |
| ENSSSCG00000029666 | HOXA13 | 5E-05 |
| ENSSSCG00000001422 | C2 | 5.19E-05 |
| ENSSSCG00000004670 | C15orf48 | 5.21E-05 |
| ENSSSCG00000014012 | GFPT2 | 5.28E-05 |
| ENSSSCG00000003914 | NA | 5.41E-05 |
| ENSSSCG00000036693 | NA | 5.42E-05 |
| ENSSSCG00000000209 | NCKAP5L | 5.5E-05 |
| ENSSSCG00000002385 | TGFB3 | 5.56E-05 |
| ENSSSCG00000028679 | NA | 5.65E-05 |
| ENSSSCG00000009384 | INTS6 | 5.65E-05 |
| ENSSSCG00000036755 | FAM46B | 5.74E-05 |
| ENSSSCG00000034364 | NA | 5.77E-05 |
| ENSSSCG00000001595 | DAAM2 | 5.91E-05 |
| ENSSSCG00000037530 | NA | 5.92E-05 |
| ENSSSCG00000002847 | GPT2 | 5.98E-05 |
| ENSSSCG00000016986 | CREBRF | 6.08E-05 |
| ENSSSCG00000038366 | RAP2B | 6.11E-05 |
| ENSSSCG00000023177 | RRP9 | 6.23E-05 |
| ENSSSCG00000004163 | BCLAF1 | 6.24E-05 |
| ENSSSCG00000011133 | PFKFB3 | 6.28E-05 |
| ENSSSCG00000037950 | NIPAL1 | 6.3E-05 |
| ENSSSCG00000000704 | TAPBPL | 6.3E-05 |
| ENSSSCG00000026116 | FHOD1 | 6.35E-05 |
| ENSSSCG00000022073 | ZBTB38 | 6.38E-05 |
| ENSSSCG00000009037 | ZNF827 | 6.51E-05 |
| ENSSSCG00000016053 | NA | 6.51E-05 |
| ENSSSCG00000011218 | SLC4A7 | 6.6E-05 |
| ENSSSCG00000025560 | PGLYRP2 | 6.64E-05 |
| ENSSSCG00000039473 | NA | 6.67E-05 |
| ENSSSCG00000015981 | HOXD10 | 6.79E-05 |
| ENSSSCG00000022280 | DACT3 | 6.8E-05 |
| ENSSSCG00000027349 | NA | 6.89E-05 |
| ENSSSCG00000025021 | NA | 6.98E-05 |
| ENSSSCG00000039542 | NA | 7.03E-05 |
| ENSSSCG00000006497 | MEX3A | 7.15E-05 |
| ENSSSCG00000029547 | NAB1 | 7.16E-05 |
| ENSSSCG00000003079 | NA | 7.22E-05 |
| ENSSSCG00000000807 | SLC38A1 | 7.22E-05 |
| ENSSSCG00000010259 | TYSND1 | 7.26E-05 |
| ENSSSCG00000032433 | PTCHD1 | 7.34E-05 |
| ENSSSCG00000010987 | UBAP2 | 7.6E-05 |
| ENSSSCG00000031875 | ZNF469 | 7.62E-05 |
| ENSSSCG00000022609 | WWP2 | 7.73E-05 |
| ENSSSCG00000022839 | NA | 7.76E-05 |
| ENSSSCG00000032949 | NA | 7.84E-05 |
| ENSSSCG00000040855 | C5orf51 | 7.9E-05 |
| ENSSSCG00000006850 | FAM102B | 7.9E-05 |
| ENSSSCG00000039045 | SLC26A2 | 7.91E-05 |
| ENSSSCG00000017137 | METRNL | 7.97E-05 |
| ENSSSCG00000016703 | HOXA5 | 8.01E-05 |
| ENSSSCG00000010247 | DDX21 | 8.09E-05 |
| ENSSSCG00000023680 | MEPCE | 8.19E-05 |
| ENSSSCG00000011425 | RAD54L2 | 8.54E-05 |
| ENSSSCG00000010322 | ZNF503 | 8.59E-05 |
| ENSSSCG00000036920 | SELENOT | 8.65E-05 |
| ENSSSCG00000033993 | PLCXD3 | 8.68E-05 |
| ENSSSCG00000010525 | NA | 8.75E-05 |
| ENSSSCG00000012181 | PDK3 | 8.98E-05 |
| ENSSSCG00000030183 | NA | 9.08E-05 |
| ENSSSCG00000005683 | TOR1B | 9.08E-05 |
| ENSSSCG00000008510 | LTBP1 | 9.18E-05 |
| ENSSSCG00000020853 | BTBD3 | 9.38E-05 |
| ENSSSCG00000037832 | PMP22 | 9.38E-05 |
| ENSSSCG00000008147 | FHL2 | 9.4E-05 |
| ENSSSCG00000026001 | DNAJC1 | 9.54E-05 |
| ENSSSCG00000012050 | RCAN1 | 9.54E-05 |
| ENSSSCG00000036060 | RRAD | 9.58E-05 |
| ENSSSCG00000006556 | TPM3 | 0.0001 |
| ENSSSCG00000000716 | KCNA1 | 0.000102 |
| ENSSSCG00000006889 | ARHGAP29 | 0.000102 |
| ENSSSCG00000001689 | GTPBP2 | 0.000103 |
| ENSSSCG00000017578 | ITGA3 | 0.000103 |
| ENSSSCG00000039514 | ID3 | 0.000104 |
| ENSSSCG00000029855 | LHFPL6 | 0.000105 |
| ENSSSCG00000010605 | STN1 | 0.000105 |
| ENSSSCG00000002930 | ZNF260 | 0.000105 |
| ENSSSCG00000036129 | RNF114 | 0.000106 |
| ENSSSCG00000003486 | ARHGEF10L | 0.000106 |
| ENSSSCG00000009886 | TRAFD1 | 0.000107 |
| ENSSSCG00000014998 | AASDHPPT | 0.000107 |
| ENSSSCG00000002337 | ZFYVE1 | 0.000107 |
| ENSSSCG00000027860 | ERAP2 | 0.000108 |
| ENSSSCG00000035539 | ST8SIA4 | 0.00011 |
| ENSSSCG00000011357 | SHISA5 | 0.000111 |
| ENSSSCG00000021393 | TRMT1L | 0.000112 |
| ENSSSCG00000028033 | SECISBP2 | 0.000114 |
| ENSSSCG00000026602 | PTGIR | 0.000115 |
| ENSSSCG00000022636 | DENND5B | 0.000115 |
| ENSSSCG00000015390 | NA | 0.000116 |
| ENSSSCG00000040166 | FLRT2 | 0.000117 |
| ENSSSCG00000007667 | LRCH4 | 0.000117 |
| ENSSSCG00000013539 | GTF2F1 | 0.000117 |
| ENSSSCG00000006395 | NA | 0.000117 |
| ENSSSCG00000005300 | VCP | 0.000118 |
| ENSSSCG00000010025 | LIMK2 | 0.000118 |
| ENSSSCG00000009999 | NA | 0.000118 |
| ENSSSCG00000017785 | NUFIP2 | 0.000118 |
| ENSSSCG00000005385 | NR4A3 | 0.000124 |
| ENSSSCG00000014072 | ENC1 | 0.000124 |
| ENSSSCG00000032046 | NA | 0.000126 |
| ENSSSCG00000011521 | PDZRN3 | 0.000126 |
| ENSSSCG00000015522 | ANGPTL1 | 0.00013 |
| ENSSSCG00000013332 | KIF18A | 0.000134 |
| ENSSSCG00000015140 | HSPA8 | 0.000135 |
| ENSSSCG00000007585 | ACTB | 0.000136 |
| ENSSSCG00000016076 | COQ10B | 0.00014 |
| ENSSSCG00000009040 | SMAD1 | 0.00014 |
| ENSSSCG00000011071 | THNSL1 | 0.00014 |
| ENSSSCG00000031701 | GXYLT1 | 0.00014 |
| ENSSSCG00000017783 | NA | 0.000142 |
| ENSSSCG00000037263 | SIAH1 | 0.000142 |
| ENSSSCG00000002859 | ANKRD27 | 0.000144 |
| ENSSSCG00000026301 | RAP2C | 0.000145 |
| ENSSSCG00000025027 | METTL1 | 0.000147 |
| ENSSSCG00000015301 | STEAP1 | 0.000151 |
| ENSSSCG00000015282 | PPP1R15B | 0.000159 |
| ENSSSCG00000023357 | NA | 0.00016 |
| ENSSSCG00000011951 | NFKBIZ | 0.000162 |
| ENSSSCG00000030888 | FADD | 0.000162 |
| ENSSSCG00000020783 | SLC41A1 | 0.000162 |
| ENSSSCG00000015346 | ICA1 | 0.000162 |
| ENSSSCG00000009864 | MED13L | 0.000164 |
| ENSSSCG00000027916 | PEX10 | 0.000165 |
| ENSSSCG00000031744 | NA | 0.000167 |
| ENSSSCG00000033946 | DIDO1 | 0.000168 |
| ENSSSCG00000012656 | ELF4 | 0.00017 |
| ENSSSCG00000025298 | TWISTNB | 0.000173 |
| ENSSSCG00000039703 | EEPD1 | 0.000176 |
| ENSSSCG00000040954 | CSTF3 | 0.000178 |
| ENSSSCG00000033422 | NA | 0.000179 |
| ENSSSCG00000009229 | ARHGAP24 | 0.000181 |
| ENSSSCG00000009403 | ITM2B | 0.000181 |
| ENSSSCG00000004490 | SETBP1 | 0.000181 |
| ENSSSCG00000035181 | RNF24 | 0.000182 |
| ENSSSCG00000029592 | GPRC5A | 0.000187 |
| ENSSSCG00000035693 | POLRMT | 0.000187 |
| ENSSSCG00000032686 | RUNX3 | 0.000187 |
| ENSSSCG00000033591 | CHD9 | 0.000189 |
| ENSSSCG00000013367 | GTF2H1 | 0.000197 |
| ENSSSCG00000006703 | PRKAB2 | 0.000199 |
| ENSSSCG00000011514 | MITF | 0.000201 |
| ENSSSCG00000011369 | WDR6 | 0.000208 |
| ENSSSCG00000017159 | TBC1D16 | 0.000211 |
| ENSSSCG00000007337 | CTNNBL1 | 0.000213 |
| ENSSSCG00000040267 | CYS1 | 0.000215 |
| ENSSSCG00000016098 | ORC2 | 0.000218 |
| ENSSSCG00000036030 | FAM212B | 0.000218 |
| ENSSSCG00000036520 | EFNA5 | 0.000218 |
| ENSSSCG00000038902 | KCNK6 | 0.000218 |
| ENSSSCG00000009482 | SPRY2 | 0.00022 |
| ENSSSCG00000016519 | AKR1D1 | 0.000221 |
| ENSSSCG00000033660 | FIGNL2 | 0.000222 |
| ENSSSCG00000003472 | ARHGEF19 | 0.000222 |
| ENSSSCG00000026954 | TMEM87B | 0.000222 |
| ENSSSCG00000013992 | ARF1 | 0.000223 |
| ENSSSCG00000009283 | TNFRSF19 | 0.000225 |
| ENSSSCG00000017073 | FAM114A2 | 0.000225 |
| ENSSSCG00000006581 | NA | 0.000226 |
| ENSSSCG00000006802 | NA | 0.000226 |
| ENSSSCG00000040184 | LMO7 | 0.000227 |
| ENSSSCG00000024439 | PTGER4 | 0.000227 |
| ENSSSCG00000004205 | ARHGAP18 | 0.000227 |
| ENSSSCG00000015310 | AKAP9 | 0.00023 |
| ENSSSCG00000032437 | RBM4 | 0.000231 |
| ENSSSCG00000034302 | SLC12A7 | 0.000232 |
| ENSSSCG00000015410 | PHTF2 | 0.000233 |
| ENSSSCG00000037732 | SINHCAF | 0.000236 |
| ENSSSCG00000035867 | GFOD1 | 0.000239 |
| ENSSSCG00000026806 | DHX9 | 0.000239 |
| ENSSSCG00000006987 | SLC7A2 | 0.000239 |
| ENSSSCG00000008311 | CYP26B1 | 0.000242 |
| ENSSSCG00000012950 | RIN1 | 0.000242 |
| ENSSSCG00000024958 | GPR173 | 0.000247 |
| ENSSSCG00000012631 | LONRF3 | 0.000251 |
| ENSSSCG00000011643 | AMOTL2 | 0.000255 |
| ENSSSCG00000011367 | ARIH2 | 0.000258 |
| ENSSSCG00000032288 | SLC20A1 | 0.000258 |
| ENSSSCG00000007155 | C20orf194 | 0.000258 |
| ENSSSCG00000007572 | LFNG | 0.000258 |
| ENSSSCG00000017865 | CTNS | 0.000259 |
| ENSSSCG00000004856 | NFATC1 | 0.000259 |
| ENSSSCG00000006728 | GDAP2 | 0.000261 |
| ENSSSCG00000000908 | NA | 0.000263 |
| ENSSSCG00000029507 | RASGEF1B | 0.000265 |
| ENSSSCG00000038487 | TMPO | 0.000265 |
| ENSSSCG00000008799 | LIMCH1 | 0.000265 |
| ENSSSCG00000016061 | MYO1B | 0.000267 |
| ENSSSCG00000003189 | PRMT1 | 0.000267 |
| ENSSSCG00000000160 | PRDM4 | 0.000273 |
| ENSSSCG00000034858 | RAP1GAP2 | 0.000274 |
| ENSSSCG00000017832 | METTL16 | 0.000275 |
| ENSSSCG00000006530 | EFNA1 | 0.00028 |
| ENSSSCG00000030415 | DPP3 | 0.00028 |
| ENSSSCG00000001049 | HIVEP1 | 0.000287 |
| ENSSSCG00000015499 | RABGAP1L | 0.000293 |
| ENSSSCG00000003329 | ACAP3 | 0.000294 |
| ENSSSCG00000006000 | TAF2 | 0.000295 |
| ENSSSCG00000009853 | WSB2 | 0.000296 |
| ENSSSCG00000013448 | MKNK2 | 0.000298 |
| ENSSSCG00000037520 | ZNF275 | 0.0003 |
| ENSSSCG00000005688 | PTGES | 0.000305 |
| ENSSSCG00000006107 | PDP1 | 0.000305 |
| ENSSSCG00000004945 | NA | 0.000307 |
| ENSSSCG00000036423 | CCDC50 | 0.000312 |
| ENSSSCG00000011127 | TAF3 | 0.000314 |
| ENSSSCG00000015650 | MAPKAPK2 | 0.000319 |
| ENSSSCG00000009278 | FGF9 | 0.000319 |
| ENSSSCG00000035495 | KITLG | 0.000319 |
| ENSSSCG00000014538 | MGAT1 | 0.000319 |
| ENSSSCG00000011065 | MASTL | 0.000319 |
| ENSSSCG00000011877 | CD86 | 0.000319 |
| ENSSSCG00000012022 | APP | 0.000319 |
| ENSSSCG00000010467 | BTAF1 | 0.00032 |
| ENSSSCG00000035914 | PSMB2 | 0.00032 |
| ENSSSCG00000038948 | ETS2 | 0.00032 |
| ENSSSCG00000035598 | EDN1 | 0.000323 |
| ENSSSCG00000013399 | TEAD1 | 0.000338 |
| ENSSSCG00000011081 | NA | 0.000339 |
| ENSSSCG00000024108 | SLC43A2 | 0.00034 |
| ENSSSCG00000008467 | EML4 | 0.000342 |
| ENSSSCG00000010514 | RRP12 | 0.000343 |
| ENSSSCG00000032434 | PLAUR | 0.000346 |
| ENSSSCG00000005944 | NDRG1 | 0.000346 |
| ENSSSCG00000035612 | COX6B2 | 0.000347 |
| ENSSSCG00000017943 | ACAP1 | 0.00035 |
| ENSSSCG00000005217 | RCL1 | 0.000353 |
| ENSSSCG00000009713 | CLCN3 | 0.000355 |
| ENSSSCG00000009152 | SGMS2 | 0.000357 |
| ENSSSCG00000013074 | RAB3IL1 | 0.000361 |
| ENSSSCG00000007356 | PLCG1 | 0.000363 |
| ENSSSCG00000033120 | PALM2 | 0.00037 |
| ENSSSCG00000012252 | DDX3X | 0.000372 |
| ENSSSCG00000026729 | TMEM150C | 0.000374 |
| ENSSSCG00000016206 | CNPPD1 | 0.000377 |
| ENSSSCG00000006582 | S100A14 | 0.000383 |
| ENSSSCG00000011678 | NA | 0.000383 |
| ENSSSCG00000004347 | FBXL4 | 0.000383 |
| ENSSSCG00000006063 | NA | 0.000384 |
| ENSSSCG00000025858 | ELN | 0.000388 |
| ENSSSCG00000017693 | AATF | 0.00039 |
| ENSSSCG00000017787 | GIT1 | 0.00039 |
| ENSSSCG00000032710 | E2F2 | 0.000391 |
| ENSSSCG00000011085 | MLLT10 | 0.000396 |
| ENSSSCG00000008227 | ST3GAL5 | 0.000398 |
| ENSSSCG00000011120 | USP6NL | 0.0004 |
| ENSSSCG00000036428 | NA | 0.000402 |
| ENSSSCG00000004625 | LEO1 | 0.000404 |
| ENSSSCG00000021791 | SENP7 | 0.000406 |
| ENSSSCG00000030680 | TCF7 | 0.000408 |
| ENSSSCG00000030642 | PCNA | 0.000409 |
| ENSSSCG00000032340 | NA | 0.000418 |
| ENSSSCG00000007451 | SLC2A10 | 0.000422 |
| ENSSSCG00000020817 | RPS16 | 0.000425 |
| ENSSSCG00000028886 | IPO7 | 0.000427 |
| ENSSSCG00000005967 | FAM84B | 0.000428 |
| ENSSSCG00000009240 | PLAC8 | 0.000429 |
| ENSSSCG00000004334 | MAP3K7 | 0.000431 |
| ENSSSCG00000025423 | KCNK5 | 0.000434 |
| ENSSSCG00000040638 | DIO2 | 0.000438 |
| ENSSSCG00000009129 | TIFA | 0.000445 |
| ENSSSCG00000009858 | FBXO21 | 0.000446 |
| ENSSSCG00000027030 | BDKRB2 | 0.000447 |
| ENSSSCG00000031881 | CDC42SE1 | 0.00045 |
| ENSSSCG00000001021 | RREB1 | 0.000452 |
| ENSSSCG00000040383 | MFAP1 | 0.000457 |
| ENSSSCG00000006496 | LMNA | 0.00046 |
| ENSSSCG00000003684 | MTCL1 | 0.000465 |
| ENSSSCG00000031666 | C11orf95 | 0.000469 |
| ENSSSCG00000031631 | GNL2 | 0.000471 |
| ENSSSCG00000024161 | NA | 0.000476 |
| ENSSSCG00000025770 | ST6GAL1 | 0.00048 |
| ENSSSCG00000016866 | GHR | 0.000481 |
| ENSSSCG00000029331 | PALLD | 0.000482 |
| ENSSSCG00000033178 | NA | 0.000485 |
| ENSSSCG00000005078 | DAAM1 | 0.000487 |
| ENSSSCG00000001073 | TPMT | 0.000496 |
| ENSSSCG00000015368 | HDAC9 | 0.000496 |
| ENSSSCG00000035454 | B4GALT1 | 0.000499 |
| ENSSSCG00000003569 | SLC9A1 | 0.000502 |
| ENSSSCG00000004902 | RNF152 | 0.000519 |
| ENSSSCG00000003451 | NA | 0.000526 |
| ENSSSCG00000040713 | TXLNG | 0.000529 |
| ENSSSCG00000000403 | BAZ2A | 0.000535 |
| ENSSSCG00000031741 | NA | 0.000536 |
| ENSSSCG00000006089 | PTDSS1 | 0.000538 |
| ENSSSCG00000017749 | NA | 0.000545 |
| ENSSSCG00000006247 | PLAG1 | 0.000546 |
| ENSSSCG00000033879 | ZNF280B | 0.00055 |
| ENSSSCG00000034493 | ST3GAL6 | 0.000552 |
| ENSSSCG00000009047 | SMARCA5 | 0.000562 |
| ENSSSCG00000010054 | ADORA2A | 0.000564 |
| ENSSSCG00000016510 | UBN2 | 0.000569 |
| ENSSSCG00000014431 | AFAP1L1 | 0.000572 |
| ENSSSCG00000011978 | CPOX | 0.000572 |
| ENSSSCG00000040208 | PAG1 | 0.000574 |
| ENSSSCG00000006340 | UAP1 | 0.000577 |
| ENSSSCG00000034994 | AKAP1 | 0.000578 |
| ENSSSCG00000030575 | TMEM158 | 0.000578 |
| ENSSSCG00000033998 | SLC43A3 | 0.000578 |
| ENSSSCG00000014249 | MARCH3 | 0.000581 |
| ENSSSCG00000039380 | NANP | 0.000586 |
| ENSSSCG00000011194 | ANKRD28 | 0.000586 |
| ENSSSCG00000040607 | MAF | 0.000591 |
| ENSSSCG00000039061 | EIF2AK4 | 0.000596 |
| ENSSSCG00000000848 | GLT8D2 | 0.000596 |
| ENSSSCG00000015979 | HOXD13 | 0.0006 |
| ENSSSCG00000009655 | EBF2 | 0.000602 |
| ENSSSCG00000017573 | XYLT2 | 0.000609 |
| ENSSSCG00000000152 | RBFOX2 | 0.00061 |
| ENSSSCG00000012855 | SLC22A18 | 0.00061 |
| ENSSSCG00000006809 | RBM15 | 0.000615 |
| ENSSSCG00000008144 | NCK2 | 0.000638 |
| ENSSSCG00000038643 | KLF11 | 0.00064 |
| ENSSSCG00000016313 | HJURP | 0.000649 |
| ENSSSCG00000010580 | NFKB2 | 0.000649 |
| ENSSSCG00000013178 | ZDHHC5 | 0.000653 |
| ENSSSCG00000035284 | BMF | 0.000655 |
| ENSSSCG00000001231 | NA | 0.000655 |
| ENSSSCG00000001697 | TMEM63B | 0.000657 |
| ENSSSCG00000011765 | USP13 | 0.000666 |
| ENSSSCG00000033185 | PPP2R3A | 0.000667 |
| ENSSSCG00000008136 | RANBP2 | 0.000668 |
| ENSSSCG00000008747 | NCAPG | 0.000674 |
| ENSSSCG00000039609 | ANKRD33B | 0.000674 |
| ENSSSCG00000026733 | HIPK2 | 0.000675 |
| ENSSSCG00000032674 | TCEA3 | 0.000675 |
| ENSSSCG00000016521 | DGKI | 0.000681 |
| ENSSSCG00000029236 | ZBTB7A | 0.000681 |
| ENSSSCG00000028762 | VPS37C | 0.000692 |
| ENSSSCG00000032914 | MANF | 0.000707 |
| ENSSSCG00000039336 | GNA13 | 0.000714 |
| ENSSSCG00000005628 | ST6GALNAC4 | 0.000726 |
| ENSSSCG00000005072 | PSMA3 | 0.000734 |
| ENSSSCG00000006155 | ZBTB10 | 0.000736 |
| ENSSSCG00000003983 | SMAP2 | 0.000736 |
| ENSSSCG00000016535 | CALD1 | 0.000744 |
| ENSSSCG00000039950 | RMI1 | 0.000756 |
| ENSSSCG00000039952 | ZFAND3 | 0.000757 |
| ENSSSCG00000038362 | CMTM6 | 0.000759 |
| ENSSSCG00000011313 | LIMD1 | 0.000759 |
| ENSSSCG00000004854 | NA | 0.000764 |
| ENSSSCG00000007541 | PDGFA | 0.000768 |
| ENSSSCG00000038806 | AXL | 0.00078 |
| ENSSSCG00000008259 | LRRTM4 | 0.000781 |
| ENSSSCG00000014994 | PDGFD | 0.000783 |
| ENSSSCG00000015823 | NA | 0.000804 |
| ENSSSCG00000034151 | RNPEP | 0.000809 |
| ENSSSCG00000011332 | SETD2 | 0.000826 |
| ENSSSCG00000008090 | IL1A | 0.000831 |
| ENSSSCG00000037645 | COTL1 | 0.000831 |
| ENSSSCG00000017773 | SUPT6H | 0.000834 |
| ENSSSCG00000017165 | USP36 | 0.000838 |
| ENSSSCG00000023245 | NONO | 0.000839 |
| ENSSSCG00000005224 | GLIS3 | 0.000852 |
| ENSSSCG00000013079 | DAGLA | 0.000863 |
| ENSSSCG00000035956 | HABP4 | 0.000864 |
| ENSSSCG00000015071 | SIK3 | 0.000871 |
| ENSSSCG00000007668 | GIGYF1 | 0.000871 |
| ENSSSCG00000012293 | PRICKLE3 | 0.000881 |
| ENSSSCG00000014195 | MAN2A1 | 0.000881 |
| ENSSSCG00000009593 | ROR2 | 0.000896 |
| ENSSSCG00000029391 | PLSCR3 | 0.000898 |
| ENSSSCG00000000719 | NA | 0.000901 |
| ENSSSCG00000038222 | CACUL1 | 0.000905 |
| ENSSSCG00000006162 | ZC2HC1A | 0.000912 |
| ENSSSCG00000007493 | NA | 0.000914 |
| ENSSSCG00000009657 | PPP2R2A | 0.000917 |
| ENSSSCG00000038965 | ARC | 0.000918 |
| ENSSSCG00000010010 | PES1 | 0.000936 |
| ENSSSCG00000014362 | HBEGF | 0.000936 |
| ENSSSCG00000012968 | CCDC85B | 0.000937 |
| ENSSSCG00000011056 | FRMD4A | 0.000945 |
| ENSSSCG00000036751 | PPM1H | 0.00095 |
| ENSSSCG00000028004 | RIN2 | 0.000962 |
| ENSSSCG00000007432 | ZNF335 | 0.000971 |
| ENSSSCG00000031958 | KCTD2 | 0.000976 |
| ENSSSCG00000012981 | RELA | 0.000976 |
| ENSSSCG00000034265 | NA | 0.000976 |
| ENSSSCG00000033291 | PIP4K2B | 0.00098 |
| ENSSSCG00000008289 | MTHFD2 | 0.000989 |
| ENSSSCG00000015387 | TRA2A | 0.000997 |
| ENSSSCG00000007463 | PTGIS | 0.001001 |
| ENSSSCG00000006487 | CCT3 | 0.001006 |
| ENSSSCG00000003192 | IL4I1 | 0.001022 |
| ENSSSCG00000013742 | NFIX | 0.001026 |
| ENSSSCG00000011746 | SKIL | 0.001058 |
| ENSSSCG00000004505 | SMAD2 | 0.001075 |
| ENSSSCG00000003763 | IFI44 | 0.001102 |
| ENSSSCG00000033015 | B3GNT7 | 0.001106 |
| ENSSSCG00000016653 | DNAJB9 | 0.001106 |
| ENSSSCG00000016884 | PELO | 0.001106 |
| ENSSSCG00000002509 | CCNK | 0.001106 |
| ENSSSCG00000028007 | POLR1C | 0.001136 |
| ENSSSCG00000024411 | NA | 0.001157 |
| ENSSSCG00000007767 | ZNF668 | 0.001176 |
| ENSSSCG00000027105 | TUBGCP5 | 0.001178 |
| ENSSSCG00000005021 | SAV1 | 0.001214 |
| ENSSSCG00000009293 | NA | 0.001225 |
| ENSSSCG00000040550 | CASC1 | 0.001225 |
| ENSSSCG00000017748 | NF1 | 0.001231 |
| ENSSSCG00000023978 | RECQL5 | 0.001234 |
| ENSSSCG00000003114 | DHX34 | 0.001236 |
| ENSSSCG00000024384 | DCAF12 | 0.001245 |
| ENSSSCG00000006947 | SYDE2 | 0.001258 |
| ENSSSCG00000009132 | ENPEP | 0.001263 |
| ENSSSCG00000004489 | EEF1A1 | 0.001263 |
| ENSSSCG00000035715 | GCH1 | 0.001264 |
| ENSSSCG00000000549 | PPFIBP1 | 0.001265 |
| ENSSSCG00000035216 | SPRED3 | 0.001324 |
| ENSSSCG00000013410 | SWAP70 | 0.001324 |
| ENSSSCG00000024635 | SPART | 0.001372 |
| ENSSSCG00000006777 | RHOC | 0.001372 |
| ENSSSCG00000021273 | NA | 0.001376 |
| ENSSSCG00000040100 | ITPKB | 0.00138 |
| ENSSSCG00000029920 | ELF2 | 0.001401 |
| ENSSSCG00000015396 | SEMA3D | 0.001426 |
| ENSSSCG00000009356 | RFXAP | 0.001432 |
| ENSSSCG00000014794 | NUP98 | 0.00144 |
| ENSSSCG00000011760 | TBL1XR1 | 0.001444 |
| ENSSSCG00000022246 | NA | 0.001448 |
| ENSSSCG00000024152 | ETV6 | 0.001448 |
| ENSSSCG00000029838 | FZD2 | 0.001448 |
| ENSSSCG00000007484 | ZNF217 | 0.00145 |
| ENSSSCG00000029849 | S1PR1 | 0.001452 |
| ENSSSCG00000016809 | DROSHA | 0.001452 |
| ENSSSCG00000014168 | ELL2 | 0.001477 |
| ENSSSCG00000023409 | TIA1 | 0.00148 |
| ENSSSCG00000028879 | PLOD3 | 0.001492 |
| ENSSSCG00000000161 | PWP1 | 0.001506 |
| ENSSSCG00000002505 | NA | 0.001512 |
| ENSSSCG00000009300 | WASF3 | 0.001514 |
| ENSSSCG00000006688 | ANKRD35 | 0.001515 |
| ENSSSCG00000009627 | NA | 0.00152 |
| ENSSSCG00000017360 | UBTF | 0.001529 |
| ENSSSCG00000007950 | ADCY9 | 0.001529 |
| ENSSSCG00000001946 | MBIP | 0.001556 |
| ENSSSCG00000002664 | GSE1 | 0.001563 |
| ENSSSCG00000033323 | UBE2D1 | 0.001577 |
| ENSSSCG00000020921 | NA | 0.001581 |
| ENSSSCG00000015136 | UBASH3B | 0.001588 |
| ENSSSCG00000031593 | NUDT3 | 0.001607 |
| ENSSSCG00000016128 | EEF1B2 | 0.00163 |
| ENSSSCG00000000146 | NA | 0.001639 |
| ENSSSCG00000033971 | ZNF407 | 0.001675 |
| ENSSSCG00000005198 | KDM4C | 0.001675 |
| ENSSSCG00000034407 | AMN1 | 0.001675 |
| ENSSSCG00000004420 | TRAF3IP2 | 0.001701 |
| ENSSSCG00000007531 | FAM217B | 0.001705 |
| ENSSSCG00000037274 | NA | 0.001705 |
| ENSSSCG00000028549 | ECM2 | 0.001712 |
| ENSSSCG00000021920 | SOX12 | 0.001723 |
| ENSSSCG00000036614 | PGP | 0.001736 |
| ENSSSCG00000012361 | AMER1 | 0.001752 |
| ENSSSCG00000023630 | CPM | 0.001755 |
| ENSSSCG00000023243 | NFIA | 0.001758 |
| ENSSSCG00000004622 | GNB5 | 0.001765 |
| ENSSSCG00000012911 | CARNS1 | 0.001776 |
| ENSSSCG00000016911 | SKIV2L2 | 0.001781 |
| ENSSSCG00000013049 | RCOR2 | 0.001781 |
| ENSSSCG00000008479 | DHX57 | 0.001781 |
| ENSSSCG00000011250 | ACAA1 | 0.001799 |
| ENSSSCG00000010212 | NA | 0.001808 |
| ENSSSCG00000006653 | ENSA | 0.001809 |
| ENSSSCG00000025326 | TMEM243 | 0.001815 |
| ENSSSCG00000000171 | CKAP4 | 0.001825 |
| ENSSSCG00000016554 | MEST | 0.001834 |
| ENSSSCG00000005631 | FAM102A | 0.001841 |
| ENSSSCG00000011940 | DZIP3 | 0.001849 |
| ENSSSCG00000000095 | GTPBP1 | 0.001856 |
| ENSSSCG00000029985 | ARL10 | 0.00187 |
| ENSSSCG00000014869 | LRRC32 | 0.001874 |
| ENSSSCG00000001064 | GMPR | 0.001896 |
| ENSSSCG00000026587 | BATF3 | 0.001896 |
| ENSSSCG00000008348 | PLEK | 0.001896 |
| ENSSSCG00000028973 | PHACTR4 | 0.001896 |
| ENSSSCG00000005073 | ARID4A | 0.001897 |
| ENSSSCG00000040181 | ELL | 0.00195 |
| ENSSSCG00000016763 | GLI3 | 0.001952 |
| ENSSSCG00000039337 | ITPRIPL2 | 0.001955 |
| ENSSSCG00000010032 | PISD | 0.001957 |
| ENSSSCG00000004114 | RAB32 | 0.001959 |
| ENSSSCG00000032176 | SMURF2 | 0.001959 |
| ENSSSCG00000013446 | SCAMP4 | 0.001961 |
| ENSSSCG00000008484 | SRSF7 | 0.001963 |
| ENSSSCG00000009430 | NA | 0.001968 |
| ENSSSCG00000006273 | MCM4 | 0.00197 |
| ENSSSCG00000007721 | GTF2I | 0.001974 |
| ENSSSCG00000003165 | RPS11 | 0.002001 |
| ENSSSCG00000004826 | SELENOS | 0.002006 |
| ENSSSCG00000016170 | BARD1 | 0.002013 |
| ENSSSCG00000008820 | TEC | 0.00202 |
| ENSSSCG00000015883 | MARCH7 | 0.002024 |
| ENSSSCG00000016248 | AGFG1 | 0.002116 |
| ENSSSCG00000000696 | NOP2 | 0.002123 |
| ENSSSCG00000028228 | XPO1 | 0.002141 |
| ENSSSCG00000021292 | OSBPL9 | 0.00217 |
| ENSSSCG00000033180 | PSMD10 | 0.002179 |
| ENSSSCG00000008698 | RGS12 | 0.002191 |
| ENSSSCG00000028983 | TBC1D1 | 0.002194 |
| ENSSSCG00000008959 | CXCL2 | 0.002199 |
| ENSSSCG00000032936 | PIM3 | 0.002205 |
| ENSSSCG00000016207 | RETREG2 | 0.002209 |
| ENSSSCG00000002340 | PSEN1 | 0.002214 |
| ENSSSCG00000000840 | WASHC4 | 0.002214 |
| ENSSSCG00000024285 | DLG4 | 0.002217 |
| ENSSSCG00000028331 | IL1R2 | 0.002217 |
| ENSSSCG00000011698 | GYG1 | 0.002217 |
| ENSSSCG00000023710 | REEP1 | 0.002223 |
| ENSSSCG00000004138 | HIVEP2 | 0.002223 |
| ENSSSCG00000010943 | C9orf3 | 0.002285 |
| ENSSSCG00000006051 | CTHRC1 | 0.002288 |
| ENSSSCG00000032266 | SLC37A3 | 0.002291 |
| ENSSSCG00000005457 | NA | 0.002308 |
| ENSSSCG00000004782 | BUB1B | 0.002308 |
| ENSSSCG00000032078 | ZNF362 | 0.002308 |
| ENSSSCG00000031598 | CDADC1 | 0.002312 |
| ENSSSCG00000034615 | TNFAIP8L1 | 0.002315 |
| ENSSSCG00000023948 | UBTD2 | 0.00233 |
| ENSSSCG00000006926 | GTF2B | 0.002335 |
| ENSSSCG00000006840 | WDR47 | 0.002361 |
| ENSSSCG00000009359 | SUPT20H | 0.002383 |
| ENSSSCG00000009236 | HELQ | 0.002386 |
| ENSSSCG00000009084 | SPATA5 | 0.002396 |
| ENSSSCG00000019154 | ssc-mir-155 | 0.0024 |
| ENSSSCG00000009389 | TRIM13 | 0.0024 |
| ENSSSCG00000000697 | CHD4 | 0.0024 |
| ENSSSCG00000016817 | TARS | 0.002401 |
| ENSSSCG00000022387 | TBPL1 | 0.002409 |
| ENSSSCG00000003559 | ARID1A | 0.002418 |
| ENSSSCG00000038731 | STX18 | 0.002419 |
| ENSSSCG00000016755 | POLM | 0.002428 |
| ENSSSCG00000016342 | HES6 | 0.002439 |
| ENSSSCG00000032527 | FOSL2 | 0.002439 |
| ENSSSCG00000002404 | SPTLC2 | 0.002443 |
| ENSSSCG00000032488 | ZADH2 | 0.002449 |
| ENSSSCG00000030358 | SARM1 | 0.002466 |
| ENSSSCG00000001372 | GNL1 | 0.002469 |
| ENSSSCG00000011301 | ZDHHC3 | 0.002469 |
| ENSSSCG00000027457 | FBXO22 | 0.002469 |
| ENSSSCG00000021408 | TKT | 0.002469 |
| ENSSSCG00000035195 | HNMT | 0.002469 |
| ENSSSCG00000027700 | RPRD1A | 0.002481 |
| ENSSSCG00000027525 | DHCR24 | 0.002482 |
| ENSSSCG00000004600 | TCF12 | 0.002482 |
| ENSSSCG00000002245 | KATNBL1 | 0.002499 |
| ENSSSCG00000013592 | ELAVL1 | 0.002522 |
| ENSSSCG00000004332 | BACH2 | 0.002529 |
| ENSSSCG00000010440 | NA | 0.002529 |
| ENSSSCG00000015255 | IGSF9B | 0.002548 |
| ENSSSCG00000015357 | MEOX2 | 0.002551 |
| ENSSSCG00000014224 | SEMA6A | 0.002551 |
| ENSSSCG00000030268 | ZFAND5 | 0.002562 |
| ENSSSCG00000010850 | NA | 0.002581 |
| ENSSSCG00000026943 | MRAP2 | 0.002606 |
| ENSSSCG00000004772 | BAHD1 | 0.002617 |
| ENSSSCG00000006727 | WDR3 | 0.002651 |
| ENSSSCG00000013893 | ARRDC2 | 0.002656 |
| ENSSSCG00000015953 | DLX1 | 0.002685 |
| ENSSSCG00000008833 | SGCB | 0.002702 |
| ENSSSCG00000039890 | RASL11A | 0.002702 |
| ENSSSCG00000021738 | NA | 0.002702 |
| ENSSSCG00000004475 | NA | 0.002734 |
| ENSSSCG00000003757 | SSX2IP | 0.002743 |
| ENSSSCG00000021698 | RRP1B | 0.002745 |
| ENSSSCG00000004617 | FAM214A | 0.00276 |
| ENSSSCG00000010912 | KIF14 | 0.002764 |
| ENSSSCG00000037568 | RAB43 | 0.002776 |
| ENSSSCG00000008604 | WDR35 | 0.002791 |
| ENSSSCG00000001840 | KIF7 | 0.002821 |
| ENSSSCG00000008164 | MAP4K4 | 0.002845 |
| ENSSSCG00000007072 | SPTLC3 | 0.002854 |
| ENSSSCG00000036584 | HAUS8 | 0.002881 |
| ENSSSCG00000003134 | GRWD1 | 0.002918 |
| ENSSSCG00000032312 | RAB11A | 0.002922 |
| ENSSSCG00000008878 | PPID | 0.002924 |
| ENSSSCG00000040796 | IPP | 0.002926 |
| ENSSSCG00000034624 | TMEM50B | 0.002931 |
| ENSSSCG00000032648 | RBL2 | 0.002931 |
| ENSSSCG00000012527 | TCEAL9 | 0.00294 |
| ENSSSCG00000007485 | BCAS1 | 0.002952 |
| ENSSSCG00000027127 | MAP2K2 | 0.002954 |
| ENSSSCG00000000274 | PCBP2 | 0.002962 |
| ENSSSCG00000035939 | ZNF512B | 0.00299 |
| ENSSSCG00000037803 | MARCKS | 0.00302 |
| ENSSSCG00000025447 | MID1IP1 | 0.003053 |
| ENSSSCG00000023604 | LAP3 | 0.003068 |
| ENSSSCG00000039408 | ADCY7 | 0.003073 |
| ENSSSCG00000008682 | NSD2 | 0.003091 |
| ENSSSCG00000016043 | OSGEPL1 | 0.003116 |
| ENSSSCG00000002276 | PLEKHG3 | 0.003147 |
| ENSSSCG00000023307 | FBXW11 | 0.003147 |
| ENSSSCG00000024626 | TNFAIP1 | 0.003159 |
| ENSSSCG00000032398 | VGLL3 | 0.00316 |
| ENSSSCG00000006290 | SLC19A2 | 0.003177 |
| ENSSSCG00000032692 | SPIN3 | 0.003177 |
| ENSSSCG00000025826 | BOC | 0.003177 |
| ENSSSCG00000000736 | TEAD4 | 0.003195 |
| ENSSSCG00000035341 | C6orf89 | 0.003202 |
| ENSSSCG00000034184 | NA | 0.003202 |
| ENSSSCG00000034927 | NA | 0.003202 |
| ENSSSCG00000027455 | SLC39A6 | 0.003202 |
| ENSSSCG00000017508 | STAC2 | 0.003207 |
| ENSSSCG00000038044 | GYG2 | 0.003242 |
| ENSSSCG00000038760 | ANKRD40 | 0.00325 |
| ENSSSCG00000017047 | CLINT1 | 0.00325 |
| ENSSSCG00000003949 | CDC20 | 0.003264 |
| ENSSSCG00000032221 | FAM110C | 0.00327 |
| ENSSSCG00000010702 | PLEKHA1 | 0.003293 |
| ENSSSCG00000032474 | CXCL10 | 0.003302 |
| ENSSSCG00000003110 | ZC3H4 | 0.003308 |
| ENSSSCG00000029359 | PHLDA3 | 0.003312 |
| ENSSSCG00000040349 | HOXD8 | 0.003312 |
| ENSSSCG00000038967 | NADK | 0.003314 |
| ENSSSCG00000025698 | SERPINE1 | 0.003316 |
| ENSSSCG00000003876 | CDKN2C | 0.003322 |
| ENSSSCG00000001834 | MFGE8 | 0.003326 |
| ENSSSCG00000008261 | HK2 | 0.003327 |
| ENSSSCG00000033036 | ZC3H7B | 0.003356 |
| ENSSSCG00000031659 | NA | 0.003364 |
| ENSSSCG00000032357 | APPL1 | 0.003369 |
| ENSSSCG00000015120 | USP2 | 0.00345 |
| ENSSSCG00000034200 | SEC22C | 0.003452 |
| ENSSSCG00000031764 | NA | 0.003496 |
| ENSSSCG00000006039 | LRP12 | 0.003499 |
| ENSSSCG00000039222 | NA | 0.0035 |
| ENSSSCG00000000091 | APOBEC3B | 0.0035 |
| ENSSSCG00000005651 | ODF2 | 0.003508 |
| ENSSSCG00000031616 | FOSB | 0.003527 |
| ENSSSCG00000036537 | NFIC | 0.003561 |
| ENSSSCG00000005204 | RANBP6 | 0.00357 |
| ENSSSCG00000031170 | TMEM123 | 0.003597 |
| ENSSSCG00000035230 | PIEZO1 | 0.003602 |
| ENSSSCG00000015106 | HYOU1 | 0.003602 |
| ENSSSCG00000011147 | NA | 0.003622 |
| ENSSSCG00000029724 | RACK1 | 0.003623 |
| ENSSSCG00000014557 | RIC8A | 0.003694 |
| ENSSSCG00000015302 | STEAP2 | 0.003738 |
| ENSSSCG00000011942 | CD47 | 0.00374 |
| ENSSSCG00000010138 | ZDHHC8 | 0.003799 |
| ENSSSCG00000002959 | FAM98C | 0.003825 |
| ENSSSCG00000036812 | MPRIP | 0.003855 |
| ENSSSCG00000030502 | CEP170 | 0.003881 |
| ENSSSCG00000004996 | KLHL28 | 0.003906 |
| ENSSSCG00000014060 | NA | 0.00392 |
| ENSSSCG00000017882 | MYBBP1A | 0.003933 |
| ENSSSCG00000035595 | HMCN1 | 0.003996 |
| ENSSSCG00000022784 | PLEKHO1 | 0.00401 |
| ENSSSCG00000023807 | ACO1 | 0.004011 |
| ENSSSCG00000034040 | TMEM11 | 0.004042 |
| ENSSSCG00000007530 | PPP1R3D | 0.004063 |
| ENSSSCG00000001527 | C6orf106 | 0.004078 |
| ENSSSCG00000017330 | MAP3K14 | 0.004103 |
| ENSSSCG00000017428 | JUP | 0.004139 |
| ENSSSCG00000011972 | FILIP1L | 0.004157 |
| ENSSSCG00000003687 | EPB41L3 | 0.004181 |
| ENSSSCG00000000162 | BTBD11 | 0.004185 |
| ENSSSCG00000032366 | MOB3A | 0.004189 |
| ENSSSCG00000007993 | CAPN15 | 0.004195 |
| ENSSSCG00000016572 | TNPO3 | 0.004196 |
| ENSSSCG00000002829 | MMP2 | 0.004213 |
| ENSSSCG00000005376 | TBC1D2 | 0.004213 |
| ENSSSCG00000000118 | MICALL1 | 0.004218 |
| ENSSSCG00000010772 | ADAM8 | 0.004265 |
| ENSSSCG00000037510 | DYNLRB1 | 0.00436 |
| ENSSSCG00000006995 | ASAH1 | 0.004382 |
| ENSSSCG00000003730 | RNF138 | 0.004382 |
| ENSSSCG00000011914 | ZDHHC23 | 0.004436 |
| ENSSSCG00000031076 | MAPK7 | 0.004436 |
| ENSSSCG00000012548 | MUM1L1 | 0.004486 |
| ENSSSCG00000006571 | INTS3 | 0.004494 |
| ENSSSCG00000008368 | UGP2 | 0.004494 |
| ENSSSCG00000011853 | RUBCN | 0.004494 |
| ENSSSCG00000029627 | NA | 0.004496 |
| ENSSSCG00000033823 | NA | 0.004496 |
| ENSSSCG00000023556 | SUPT5H | 0.004507 |
| ENSSSCG00000025492 | NUDT13 | 0.004527 |
| ENSSSCG00000029203 | TOX4 | 0.004527 |
| ENSSSCG00000013659 | DNMT1 | 0.004575 |
| ENSSSCG00000015962 | SP3 | 0.004581 |
| ENSSSCG00000030089 | RBM18 | 0.004606 |
| ENSSSCG00000004570 | NA | 0.004621 |
| ENSSSCG00000013889 | NA | 0.00464 |
| ENSSSCG00000022901 | KLHDC3 | 0.004671 |
| ENSSSCG00000014207 | APC | 0.004683 |
| ENSSSCG00000005510 | PHF19 | 0.004713 |
| ENSSSCG00000004551 | ZNF609 | 0.004759 |
| ENSSSCG00000039480 | ERLIN2 | 0.004759 |
| ENSSSCG00000013335 | LGR4 | 0.004808 |
| ENSSSCG00000037549 | GCLM | 0.00481 |
| ENSSSCG00000032422 | NA | 0.004818 |
| ENSSSCG00000029154 | NOSIP | 0.004818 |
| ENSSSCG00000000767 | ATP6V1E1 | 0.004899 |
| ENSSSCG00000017732 | ZNF207 | 0.004922 |
| ENSSSCG00000010575 | PPRC1 | 0.004923 |
| ENSSSCG00000034821 | ARMCX4 | 0.004931 |
| ENSSSCG00000023539 | MBOAT2 | 0.005011 |
| ENSSSCG00000036855 | SLC25A36 | 0.005048 |
| ENSSSCG00000010027 | PATZ1 | 0.00505 |
| ENSSSCG00000012108 | WWC3 | 0.005054 |
| ENSSSCG00000015244 | APLP2 | 0.005071 |
| ENSSSCG00000033411 | ZBED5 | 0.005071 |
| ENSSSCG00000021576 | CD83 | 0.005071 |
| ENSSSCG00000013913 | KLHL26 | 0.005094 |
| ENSSSCG00000013517 | UHRF1 | 0.005106 |
| ENSSSCG00000023829 | CCP110 | 0.005108 |
| ENSSSCG00000005598 | SCAI | 0.005136 |
| ENSSSCG00000029662 | RASSF4 | 0.005147 |
| ENSSSCG00000031518 | STARD4 | 0.005165 |
| ENSSSCG00000025160 | DPF1 | 0.005171 |
| ENSSSCG00000013855 | FAM32A | 0.005191 |
| ENSSSCG00000003553 | MTFR1L | 0.005215 |
| ENSSSCG00000033115 | IFNAR1 | 0.005215 |
| ENSSSCG00000011217 | NEK10 | 0.005215 |
| ENSSSCG00000029408 | ZFP30 | 0.005234 |
| ENSSSCG00000015483 | METTL13 | 0.00524 |
| ENSSSCG00000004835 | MAGEL2 | 0.005325 |
| ENSSSCG00000035297 | ISG12(A) | 0.005435 |
| ENSSSCG00000012365 | MSN | 0.005473 |
| ENSSSCG00000032705 | USP42 | 0.00549 |
| ENSSSCG00000023998 | PSMD2 | 0.005523 |
| ENSSSCG00000009729 | ZNF84 | 0.005523 |
| ENSSSCG00000016701 | HOXA7 | 0.005541 |
| ENSSSCG00000017817 | PRPF8 | 0.00556 |
| ENSSSCG00000008503 | FEZ2 | 0.00557 |
| ENSSSCG00000003376 | PLEKHG5 | 0.00559 |
| ENSSSCG00000022464 | KBTBD2 | 0.00559 |
| ENSSSCG00000026389 | SYNJ2BP | 0.00563 |
| ENSSSCG00000002433 | PSMC1 | 0.005698 |
| ENSSSCG00000009967 | NA | 0.005698 |
| ENSSSCG00000012727 | FMR1 | 0.005745 |
| ENSSSCG00000037619 | EEF1AKMT3 | 0.005768 |
| ENSSSCG00000016111 | FZD7 | 0.005789 |
| ENSSSCG00000029201 | AJUBA | 0.005796 |
| ENSSSCG00000030225 | TRA2B | 0.005818 |
| ENSSSCG00000011593 | TMCC1 | 0.005892 |
| ENSSSCG00000024938 | SH3BP5 | 0.005908 |
| ENSSSCG00000007618 | ZNF789 | 0.005908 |
| ENSSSCG00000014960 | AMOTL1 | 0.005919 |
| ENSSSCG00000013409 | SBF2 | 0.005919 |
| ENSSSCG00000002651 | CDT1 | 0.005931 |
| ENSSSCG00000015277 | SOX13 | 0.006014 |
| ENSSSCG00000008812 | ATP10D | 0.006014 |
| ENSSSCG00000027206 | PARD6B | 0.006014 |
| ENSSSCG00000011549 | OGG1 | 0.006021 |
| ENSSSCG00000038879 | RELB | 0.006073 |
| ENSSSCG00000011358 | TREX1 | 0.006073 |
| ENSSSCG00000007000 | FAT1 | 0.006139 |
| ENSSSCG00000007672 | POP7 | 0.006164 |
| ENSSSCG00000011953 | ZBTB11 | 0.006165 |
| ENSSSCG00000016090 | SPATS2L | 0.006166 |
| ENSSSCG00000033298 | SAMD1 | 0.00618 |
| ENSSSCG00000034814 | MRTO4 | 0.006185 |
| ENSSSCG00000008624 | LPIN1 | 0.00622 |
| ENSSSCG00000036352 | RAB7B | 0.006229 |
| ENSSSCG00000009859 | NA | 0.00628 |
| ENSSSCG00000022005 | SFXN3 | 0.00628 |
| ENSSSCG00000015037 | IL18 | 0.006281 |
| ENSSSCG00000031509 | MTMR12 | 0.006294 |
| ENSSSCG00000028485 | YBX1 | 0.006302 |
| ENSSSCG00000001903 | EDC3 | 0.006441 |
| ENSSSCG00000034570 | IFI6 | 0.006461 |
| ENSSSCG00000009921 | OASL | 0.006469 |
| ENSSSCG00000027872 | MYBL1 | 0.006474 |
| ENSSSCG00000001878 | PTPN9 | 0.006481 |
| ENSSSCG00000006645 | SETDB1 | 0.006492 |
| ENSSSCG00000014146 | RASA1 | 0.006505 |
| ENSSSCG00000015336 | SLC25A13 | 0.006554 |
| ENSSSCG00000039652 | OTUD3 | 0.006594 |
| ENSSSCG00000016381 | SNED1 | 0.006595 |
| ENSSSCG00000001701 | NA | 0.006618 |
| ENSSSCG00000002799 | CNOT1 | 0.006637 |
| ENSSSCG00000001653 | PEX6 | 0.006642 |
| ENSSSCG00000004002 | RPS5 | 0.006692 |
| ENSSSCG00000033768 | LAX1 | 0.006747 |
| ENSSSCG00000024759 | CX3CL1 | 0.00677 |
| ENSSSCG00000010531 | R3HCC1L | 0.006792 |
| ENSSSCG00000039947 | KCNJ2 | 0.006883 |
| ENSSSCG00000022162 | RAB11FIP5 | 0.007002 |
| ENSSSCG00000005378 | ANKS6 | 0.007009 |
| ENSSSCG00000011215 | OXSM | 0.007021 |
| ENSSSCG00000039472 | SLC30A1 | 0.007026 |
| ENSSSCG00000011340 | SMARCC1 | 0.007026 |
| ENSSSCG00000003738 | MAPRE2 | 0.007097 |
| ENSSSCG00000003301 | PPP6R1 | 0.007163 |
| ENSSSCG00000017052 | ADAM19 | 0.007205 |
| ENSSSCG00000001750 | PAQR8 | 0.007205 |
| ENSSSCG00000009223 | NUDT9 | 0.007208 |
| ENSSSCG00000004497 | PSTPIP2 | 0.007221 |
| ENSSSCG00000031251 | DDA1 | 0.007221 |
| ENSSSCG00000025910 | ZNF277 | 0.007221 |
| ENSSSCG00000022322 | BCL2L11 | 0.007236 |
| ENSSSCG00000015872 | GPD2 | 0.007252 |
| ENSSSCG00000016550 | KLF14 | 0.007308 |
| ENSSSCG00000010128 | SEPT5 | 0.007308 |
| ENSSSCG00000036946 | NA | 0.00733 |
| ENSSSCG00000023760 | CLEC14A | 0.007346 |
| ENSSSCG00000015780 | STOX2 | 0.007373 |
| ENSSSCG00000008300 | STAMBP | 0.007373 |
| ENSSSCG00000006481 | GPATCH4 | 0.007403 |
| ENSSSCG00000010108 | KLHL22 | 0.007504 |
| ENSSSCG00000026404 | SERTAD4 | 0.007504 |
| ENSSSCG00000003042 | RPS19 | 0.007518 |
| ENSSSCG00000016781 | TRIO | 0.007518 |
| ENSSSCG00000009011 | FHDC1 | 0.007518 |
| ENSSSCG00000006542 | KCNN3 | 0.007524 |
| ENSSSCG00000000141 | EIF3D | 0.007524 |
| ENSSSCG00000021466 | BMT2 | 0.007524 |
| ENSSSCG00000014568 | ST5 | 0.007529 |
| ENSSSCG00000013755 | CCDC130 | 0.007545 |
| ENSSSCG00000017731 | PSMD11 | 0.007545 |
| ENSSSCG00000025417 | BBS2 | 0.007556 |
| ENSSSCG00000014041 | MXD3 | 0.0076 |
| ENSSSCG00000002820 | RSPRY1 | 0.00763 |
| ENSSSCG00000008816 | NFXL1 | 0.007742 |
| ENSSSCG00000032457 | NA | 0.007756 |
| ENSSSCG00000023174 | USP53 | 0.00776 |
| ENSSSCG00000036014 | RPLP0 | 0.007772 |
| ENSSSCG00000016322 | ACKR3 | 0.00779 |
| ENSSSCG00000002374 | DLST | 0.007907 |
| ENSSSCG00000016266 | CAB39 | 0.007907 |
| ENSSSCG00000029578 | NFATC3 | 0.007993 |
| ENSSSCG00000003473 | RSG1 | 0.008015 |
| ENSSSCG00000011230 | OSBPL10 | 0.008023 |
| ENSSSCG00000001734 | NA | 0.008023 |
| ENSSSCG00000035371 | NA | 0.008023 |
| ENSSSCG00000011004 | TOPORS | 0.008058 |
| ENSSSCG00000040608 | AKR1B1 | 0.008058 |
| ENSSSCG00000024064 | RNF6 | 0.008078 |
| ENSSSCG00000039998 | CLIC2 | 0.00812 |
| ENSSSCG00000016518 | TRIM24 | 0.008152 |
| ENSSSCG00000013460 | OAZ1 | 0.008152 |
| ENSSSCG00000010904 | NEK7 | 0.008258 |
| ENSSSCG00000011603 | NA | 0.008259 |
| ENSSSCG00000004751 | CHP1 | 0.008277 |
| ENSSSCG00000017104 | NSUN2 | 0.008294 |
| ENSSSCG00000000874 | GAS2L3 | 0.008317 |
| ENSSSCG00000022227 | BRD4 | 0.008327 |
| ENSSSCG00000031159 | BEND3 | 0.00843 |
| ENSSSCG00000009791 | RSRC2 | 0.008462 |
| ENSSSCG00000023091 | NA | 0.008469 |
| ENSSSCG00000018015 | DNAH9 | 0.008645 |
| ENSSSCG00000010209 | FAM13C | 0.008671 |
| ENSSSCG00000036317 | VASP | 0.008723 |
| ENSSSCG00000000910 | CRADD | 0.008735 |
| ENSSSCG00000017690 | DHRS11 | 0.008774 |
| ENSSSCG00000031912 | NA | 0.008783 |
| ENSSSCG00000005361 | ALDH1B1 | 0.008795 |
| ENSSSCG00000012377 | NA | 0.008846 |
| ENSSSCG00000039474 | FBXL14 | 0.008861 |
| ENSSSCG00000017525 | NFE2L1 | 0.00888 |
| ENSSSCG00000005917 | HSF1 | 0.008961 |
| ENSSSCG00000004705 | MAP1A | 0.008962 |
| ENSSSCG00000014163 | SLF1 | 0.008963 |
| ENSSSCG00000000084 | ATF4 | 0.008988 |
| ENSSSCG00000010826 | MARC2 | 0.008988 |
| ENSSSCG00000017197 | FBF1 | 0.00903 |
| ENSSSCG00000000104 | DDX17 | 0.009052 |
| ENSSSCG00000003468 | PLEKHM2 | 0.009055 |
| ENSSSCG00000012435 | MAGT1 | 0.009055 |
| ENSSSCG00000002406 | AHSA1 | 0.009217 |
| ENSSSCG00000011356 | UQCRC1 | 0.009231 |
| ENSSSCG00000030042 | SBNO2 | 0.009232 |
| ENSSSCG00000006274 | PRKDC | 0.009232 |
| ENSSSCG00000026181 | AKTIP | 0.009301 |
| ENSSSCG00000040017 | NKX2-2 | 0.009322 |
| ENSSSCG00000008201 | CNNM3 | 0.00936 |
| ENSSSCG00000003744 | MOCOS | 0.00936 |
| ENSSSCG00000009477 | EDNRB | 0.009385 |
| ENSSSCG00000030115 | HS1BP3 | 0.009407 |
| ENSSSCG00000016223 | ACSL3 | 0.009462 |
| ENSSSCG00000006899 | RPL5 | 0.009462 |
| ENSSSCG00000017983 | PER1 | 0.00951 |
| ENSSSCG00000031349 | ZNF580 | 0.009569 |
| ENSSSCG00000007801 | ZNF48 | 0.009611 |
| ENSSSCG00000028258 | AZI2 | 0.009729 |
| ENSSSCG00000035430 | YPEL3 | 0.009729 |
| ENSSSCG00000011104 | CUL2 | 0.00979 |
| ENSSSCG00000033363 | NA | 0.00979 |
| ENSSSCG00000004572 | NA | 0.009821 |
| ENSSSCG00000039255 | SNORD44 | 0.009885 |
| ENSSSCG00000038541 | TMEM60 | 0.009907 |
| ENSSSCG00000009113 | METTL14 | 0.009935 |
| ENSSSCG00000004561 | HERC1 | 0.010066 |
| ENSSSCG00000025568 | TEAD2 | 0.010071 |
| ENSSSCG00000036364 | EGR4 | 0.010123 |
| ENSSSCG00000028227 | RPN2 | 0.010204 |
| ENSSSCG00000024674 | ABL2 | 0.010251 |
| ENSSSCG00000003897 | MOB3C | 0.0103 |
| ENSSSCG00000002670 | USP10 | 0.0104 |
| ENSSSCG00000021343 | ZEB2 | 0.010455 |
| ENSSSCG00000012405 | RPS4X | 0.010455 |
| ENSSSCG00000000753 | WNK1 | 0.010531 |
| ENSSSCG00000004535 | TCF4 | 0.0106 |
| ENSSSCG00000012375 | DLG3 | 0.010625 |
| ENSSSCG00000010835 | AIDA | 0.010628 |
| ENSSSCG00000039847 | C1S | 0.010678 |
| ENSSSCG00000012287 | TFE3 | 0.010733 |
| ENSSSCG00000001892 | SCAMP2 | 0.010775 |
| ENSSSCG00000033465 | HIVEP3 | 0.010775 |
| ENSSSCG00000013028 | ESRRA | 0.010824 |
| ENSSSCG00000009055 | ELMOD2 | 0.010824 |
| ENSSSCG00000005590 | PSMB7 | 0.010846 |
| ENSSSCG00000017289 | DDX42 | 0.010876 |
| ENSSSCG00000003558 | RPS6KA1 | 0.01088 |
| ENSSSCG00000026893 | NA | 0.010886 |
| ENSSSCG00000009298 | CDK8 | 0.010896 |
| ENSSSCG00000024671 | WNT9A | 0.010913 |
| ENSSSCG00000011576 | HRH1 | 0.010915 |
| ENSSSCG00000035090 | RAC1 | 0.010937 |
| ENSSSCG00000040595 | BRAP | 0.011006 |
| ENSSSCG00000022300 | MAT2A | 0.011025 |
| ENSSSCG00000040215 | TFAP4 | 0.011064 |
| ENSSSCG00000016836 | NADK2 | 0.011178 |
| ENSSSCG00000000119 | EIF3L | 0.011289 |
| ENSSSCG00000000189 | LMBR1L | 0.011324 |
| ENSSSCG00000009818 | IFT81 | 0.011324 |
| ENSSSCG00000027278 | ARL6 | 0.011374 |
| ENSSSCG00000004177 | RPS12 | 0.011465 |
| ENSSSCG00000011621 | SEC61A1 | 0.011483 |
| ENSSSCG00000036700 | RPUSD2 | 0.011523 |
| ENSSSCG00000036741 | PITX1 | 0.011526 |
| ENSSSCG00000014411 | LARS | 0.011539 |
| ENSSSCG00000025293 | HLTF | 0.011579 |
| ENSSSCG00000039887 | MARCH5 | 0.011593 |
| ENSSSCG00000008218 | RNF103 | 0.011668 |
| ENSSSCG00000040433 | NA | 0.011668 |
| ENSSSCG00000004144 | HECA | 0.011675 |
| ENSSSCG00000012153 | RAI2 | 0.011685 |
| ENSSSCG00000025323 | SF3B3 | 0.0117 |
| ENSSSCG00000005659 | ZER1 | 0.011713 |
| ENSSSCG00000034741 | HOXD11 | 0.011747 |
| ENSSSCG00000022864 | NA | 0.011762 |
| ENSSSCG00000009185 | ADH5 | 0.011762 |
| ENSSSCG00000039024 | SPSB3 | 0.011792 |
| ENSSSCG00000034835 | SLC7A6 | 0.011942 |
| ENSSSCG00000034049 | NA | 0.011942 |
| ENSSSCG00000026746 | NA | 0.011957 |
| ENSSSCG00000002904 | HAUS5 | 0.01199 |
| ENSSSCG00000002957 | GGN | 0.012036 |
| ENSSSCG00000024780 | ZFAND1 | 0.012118 |
| ENSSSCG00000015085 | IL10RA | 0.012142 |
| ENSSSCG00000000406 | NA | 0.012251 |
| ENSSSCG00000030211 | NBR1 | 0.012251 |
| ENSSSCG00000030535 | SNX9 | 0.012257 |
| ENSSSCG00000028132 | NAA20 | 0.012257 |
| ENSSSCG00000015052 | USP28 | 0.012296 |
| ENSSSCG00000012265 | CHST7 | 0.01233 |
| ENSSSCG00000003489 | EMC1 | 0.012411 |
| ENSSSCG00000015861 | WDR33 | 0.012525 |
| ENSSSCG00000002831 | IRX3 | 0.01259 |
| ENSSSCG00000006510 | NA | 0.012778 |
| ENSSSCG00000012241 | NA | 0.012796 |
| ENSSSCG00000011582 | CAND2 | 0.012812 |
| ENSSSCG00000007252 | DNMT3B | 0.012815 |
| ENSSSCG00000032144 | ZNF140 | 0.012821 |
| ENSSSCG00000026382 | PPP2R5E | 0.012831 |
| ENSSSCG00000004859 | ZNF516 | 0.012831 |
| ENSSSCG00000027443 | MRAS | 0.01285 |
| ENSSSCG00000003577 | WASF2 | 0.012876 |
| ENSSSCG00000026748 | PLK1 | 0.012978 |
| ENSSSCG00000007249 | NOL4L | 0.012978 |
| ENSSSCG00000003169 | PIH1D1 | 0.013021 |
| ENSSSCG00000020934 | NA | 0.013027 |
| ENSSSCG00000005227 | PUM3 | 0.013081 |
| ENSSSCG00000007955 | CLUAP1 | 0.013081 |
| ENSSSCG00000002298 | ZFYVE26 | 0.01311 |
| ENSSSCG00000021129 | PFKM | 0.013295 |
| ENSSSCG00000026812 | EMG1 | 0.013312 |
| ENSSSCG00000035537 | RUNX1 | 0.013322 |
| ENSSSCG00000037232 | SNX29 | 0.013346 |
| ENSSSCG00000039182 | C11orf96 | 0.013368 |
| ENSSSCG00000031083 | NA | 0.013396 |
| ENSSSCG00000002681 | HSDL1 | 0.013429 |
| ENSSSCG00000022536 | SLC37A2 | 0.013467 |
| ENSSSCG00000024119 | GLB1 | 0.013499 |
| ENSSSCG00000024444 | DDX59 | 0.013499 |
| ENSSSCG00000020803 | BRPF3 | 0.013514 |
| ENSSSCG00000009974 | EWSR1 | 0.013514 |
| ENSSSCG00000017546 | ZNF652 | 0.013514 |
| ENSSSCG00000002804 | CSNK2A2 | 0.013514 |
| ENSSSCG00000014802 | NUMA1 | 0.013547 |
| ENSSSCG00000004753 | INO80 | 0.013547 |
| ENSSSCG00000029039 | BRCA2 | 0.013571 |
| ENSSSCG00000006358 | NDUFS2 | 0.013598 |
| ENSSSCG00000001987 | RIPK3 | 0.013617 |
| ENSSSCG00000037779 | CAPN10 | 0.01366 |
| ENSSSCG00000025374 | DVL3 | 0.013669 |
| ENSSSCG00000007108 | KIZ | 0.013741 |
| ENSSSCG00000009473 | MYCBP2 | 0.013743 |
| ENSSSCG00000039243 | CD81 | 0.013743 |
| ENSSSCG00000009622 | POLR3D | 0.013747 |
| ENSSSCG00000020710 | PRDM2 | 0.013792 |
| ENSSSCG00000013938 | GATAD2A | 0.013835 |
| ENSSSCG00000038947 | NA | 0.014001 |
| ENSSSCG00000004469 | LCA5 | 0.014045 |
| ENSSSCG00000008617 | FAM49A | 0.014062 |
| ENSSSCG00000014023 | RUFY1 | 0.014118 |
| ENSSSCG00000011570 | IRAK2 | 0.014127 |
| ENSSSCG00000027676 | NA | 0.014164 |
| ENSSSCG00000010915 | NAV1 | 0.014242 |
| ENSSSCG00000003460 | CASP9 | 0.014242 |
| ENSSSCG00000011612 | RPN1 | 0.014242 |
| ENSSSCG00000015982 | HOXD9 | 0.014308 |
| ENSSSCG00000025953 | OGFOD1 | 0.014308 |
| ENSSSCG00000004795 | MEIS2 | 0.014385 |
| ENSSSCG00000007720 | GTF2IRD1 | 0.014391 |
| ENSSSCG00000034427 | MEX3C | 0.014423 |
| ENSSSCG00000004146 | REPS1 | 0.014498 |
| ENSSSCG00000036201 | NPR3 | 0.0145 |
| ENSSSCG00000000180 | FKBP11 | 0.014502 |
| ENSSSCG00000031342 | NA | 0.014502 |
| ENSSSCG00000003402 | PGD | 0.014502 |
| ENSSSCG00000023848 | C8orf46 | 0.014557 |
| ENSSSCG00000001880 | SIN3A | 0.014665 |
| ENSSSCG00000008268 | AUP1 | 0.014675 |
| ENSSSCG00000030617 | INTS12 | 0.014684 |
| ENSSSCG00000012546 | NRK | 0.014705 |
| ENSSSCG00000039460 | NA | 0.014782 |
| ENSSSCG00000035746 | ECT2 | 0.014849 |
| ENSSSCG00000038543 | CENPA | 0.014859 |
| ENSSSCG00000028504 | RFC2 | 0.014912 |
| ENSSSCG00000007854 | DCUN1D3 | 0.015074 |
| ENSSSCG00000009885 | HECTD4 | 0.015124 |
| ENSSSCG00000039157 | CLASP1 | 0.015124 |
| ENSSSCG00000028725 | TMEM102 | 0.015145 |
| ENSSSCG00000005110 | SYNE2 | 0.015222 |
| ENSSSCG00000031547 | NA | 0.015222 |
| ENSSSCG00000006360 | B4GALT3 | 0.015265 |
| ENSSSCG00000031456 | ARL5B | 0.015335 |
| ENSSSCG00000010573 | NA | 0.015335 |
| ENSSSCG00000034758 | PDXK | 0.015335 |
| ENSSSCG00000023975 | C15orf52 | 0.015401 |
| ENSSSCG00000021997 | ALS2CL | 0.015487 |
| ENSSSCG00000037355 | RNF115 | 0.015571 |
| ENSSSCG00000010116 | SLC25A1 | 0.015702 |
| ENSSSCG00000032397 | LBR | 0.015723 |
| ENSSSCG00000006970 | DLC1 | 0.015745 |
| ENSSSCG00000006540 | PBXIP1 | 0.015783 |
| ENSSSCG00000004578 | ANXA2 | 0.015783 |
| ENSSSCG00000032517 | DMXL2 | 0.015783 |
| ENSSSCG00000033753 | UBXN2A | 0.015783 |
| ENSSSCG00000006870 | NA | 0.015783 |
| ENSSSCG00000014900 | RAB30 | 0.015838 |
| ENSSSCG00000017509 | NA | 0.015856 |
| ENSSSCG00000009620 | BMP1 | 0.015858 |
| ENSSSCG00000007895 | ZC3H7A | 0.015864 |
| ENSSSCG00000031707 | CTDSP2 | 0.015864 |
| ENSSSCG00000004033 | AGPAT4 | 0.015864 |
| ENSSSCG00000009422 | TSC22D1 | 0.015922 |
| ENSSSCG00000024754 | BBS9 | 0.015947 |
| ENSSSCG00000001639 | TRERF1 | 0.015948 |
| ENSSSCG00000000839 | ALDH1L2 | 0.01598 |
| ENSSSCG00000011196 | DPH3 | 0.016082 |
| ENSSSCG00000039861 | HDHD3 | 0.016084 |
| ENSSSCG00000015649 | DYRK3 | 0.016178 |
| ENSSSCG00000003326 | NA | 0.016222 |
| ENSSSCG00000036494 | ERG28 | 0.016223 |
| ENSSSCG00000039905 | C1orf109 | 0.016224 |
| ENSSSCG00000011272 | NA | 0.016234 |
| ENSSSCG00000026819 | NID1 | 0.016291 |
| ENSSSCG00000038776 | ASNA1 | 0.016387 |
| ENSSSCG00000010679 | EIF3A | 0.016434 |
| ENSSSCG00000004049 | ACAT2 | 0.016434 |
| ENSSSCG00000036592 | SRSF2 | 0.016439 |
| ENSSSCG00000000058 | SNU13 | 0.016439 |
| ENSSSCG00000029403 | EMC8 | 0.016448 |
| ENSSSCG00000017366 | HDAC5 | 0.016457 |
| ENSSSCG00000037209 | TLNRD1 | 0.016528 |
| ENSSSCG00000005455 | SVEP1 | 0.016647 |
| ENSSSCG00000021490 | PRDM16 | 0.016649 |
| ENSSSCG00000033010 | AHNAK | 0.016649 |
| ENSSSCG00000011329 | NA | 0.016718 |
| ENSSSCG00000000555 | ITPR2 | 0.016739 |
| ENSSSCG00000002464 | PRIMA1 | 0.016753 |
| ENSSSCG00000014598 | PPFIBP2 | 0.016879 |
| ENSSSCG00000032668 | PRR12 | 0.016955 |
| ENSSSCG00000038907 | NA | 0.01699 |
| ENSSSCG00000034581 | LRIG2 | 0.01699 |
| ENSSSCG00000037143 | NA | 0.01699 |
| ENSSSCG00000039265 | COX6A1 | 0.017049 |
| ENSSSCG00000034630 | ABI2 | 0.017134 |
| ENSSSCG00000022099 | TP53INP2 | 0.017158 |
| ENSSSCG00000028553 | ILF2 | 0.017216 |
| ENSSSCG00000023033 | PARP3 | 0.017216 |
| ENSSSCG00000013303 | ABTB2 | 0.017255 |
| ENSSSCG00000033792 | NA | 0.01726 |
| ENSSSCG00000013731 | DNASE2 | 0.017284 |
| ENSSSCG00000032620 | PLCXD2 | 0.017295 |
| ENSSSCG00000024517 | AKAP6 | 0.017295 |
| ENSSSCG00000006954 | EEF1D | 0.017333 |
| ENSSSCG00000006780 | WNT2B | 0.017348 |
| ENSSSCG00000032467 | RAB5C | 0.01757 |
| ENSSSCG00000002724 | PDPR | 0.01757 |
| ENSSSCG00000010854 | TMEM63A | 0.01757 |
| ENSSSCG00000001958 | BAZ1A | 0.017676 |
| ENSSSCG00000011848 | TFRC | 0.017679 |
| ENSSSCG00000031102 | C1orf174 | 0.017679 |
| ENSSSCG00000036601 | ZNF805 | 0.017729 |
| ENSSSCG00000015206 | CCDC15 | 0.017739 |
| ENSSSCG00000006074 | STK3 | 0.017771 |
| ENSSSCG00000013064 | EEF1G | 0.01781 |
| ENSSSCG00000023599 | TIMM8B | 0.01781 |
| ENSSSCG00000013437 | TCF3 | 0.01781 |
| ENSSSCG00000007719 | NA | 0.017828 |
| ENSSSCG00000036307 | OAF | 0.017917 |
| ENSSSCG00000010669 | NA | 0.017935 |
| ENSSSCG00000033074 | ZCCHC24 | 0.017973 |
| ENSSSCG00000005022 | NIN | 0.017973 |
| ENSSSCG00000014179 | FAM174A | 0.018104 |
| ENSSSCG00000021483 | NPBWR1 | 0.018104 |
| ENSSSCG00000021628 | TMEM70 | 0.01812 |
| ENSSSCG00000001805 | WHAMM | 0.018162 |
| ENSSSCG00000021704 | PTPDC1 | 0.018263 |
| ENSSSCG00000024463 | PJA1 | 0.018263 |
| ENSSSCG00000006280 | GORAB | 0.018263 |
| ENSSSCG00000035211 | GCC1 | 0.018286 |
| ENSSSCG00000000386 | ANKRD52 | 0.018318 |
| ENSSSCG00000017986 | NDEL1 | 0.018348 |
| ENSSSCG00000007592 | EIF2AK1 | 0.018355 |
| ENSSSCG00000029616 | FAAP100 | 0.018367 |
| ENSSSCG00000026959 | NPAT | 0.018393 |
| ENSSSCG00000020962 | DYNLL2 | 0.018473 |
| ENSSSCG00000040010 | BCL2A1 | 0.018487 |
| ENSSSCG00000022790 | SNX2 | 0.018626 |
| ENSSSCG00000015486 | PIGC | 0.018715 |
| ENSSSCG00000004537 | TXNL1 | 0.018738 |
| ENSSSCG00000015307 | CDK14 | 0.018752 |
| ENSSSCG00000034632 | PDXP | 0.018753 |
| ENSSSCG00000035937 | DPT | 0.018755 |
| ENSSSCG00000010599 | PDCD11 | 0.018963 |
| ENSSSCG00000000074 | MCHR1 | 0.019074 |
| ENSSSCG00000038459 | SURF2 | 0.019132 |
| ENSSSCG00000031576 | TMEM42 | 0.019299 |
| ENSSSCG00000000509 | ZFC3H1 | 0.019309 |
| ENSSSCG00000014114 | PAPD4 | 0.019332 |
| ENSSSCG00000030241 | TSC22D3 | 0.019336 |
| ENSSSCG00000004695 | WDR76 | 0.01937 |
| ENSSSCG00000027722 | BMP2K | 0.019421 |
| ENSSSCG00000021890 | GNB1 | 0.019456 |
| ENSSSCG00000022107 | CHTOP | 0.01949 |
| ENSSSCG00000009345 | PDS5B | 0.019501 |
| ENSSSCG00000010107 | MED15 | 0.01959 |
| ENSSSCG00000005008 | POLE2 | 0.01961 |
| ENSSSCG00000037594 | PNISR | 0.019654 |
| ENSSSCG00000003098 | PPP5C | 0.019703 |
| ENSSSCG00000035650 | NA | 0.019984 |
| ENSSSCG00000030260 | PFN2 | 0.020095 |
| ENSSSCG00000010023 | PLA2G3 | 0.020095 |
| ENSSSCG00000033295 | EIF2S1 | 0.02015 |
| ENSSSCG00000029785 | RPS9 | 0.020158 |
| ENSSSCG00000006664 | MTMR11 | 0.020164 |
| ENSSSCG00000017733 | C17orf75 | 0.020191 |
| ENSSSCG00000000078 | TNRC6B | 0.020379 |
| ENSSSCG00000003384 | DNAJC11 | 0.020434 |
| ENSSSCG00000036482 | ZSWIM7 | 0.020434 |
| ENSSSCG00000037661 | INAFM2 | 0.020482 |
| ENSSSCG00000000958 | DYRK2 | 0.020547 |
| ENSSSCG00000038370 | UQCC3 | 0.020618 |
| ENSSSCG00000004745 | RPAP1 | 0.020823 |
| ENSSSCG00000004927 | CLPX | 0.020833 |
| ENSSSCG00000035448 | SS18L1 | 0.020908 |
| ENSSSCG00000033175 | SASH1 | 0.020908 |
| ENSSSCG00000012847 | TALDO1 | 0.020911 |
| ENSSSCG00000030118 | SAP30 | 0.020958 |
| ENSSSCG00000008179 | REV1 | 0.020974 |
| ENSSSCG00000013065 | ASRGL1 | 0.02099 |
| ENSSSCG00000009692 | NA | 0.021019 |
| ENSSSCG00000023391 | LONP2 | 0.021054 |
| ENSSSCG00000004411 | ZBTB24 | 0.021058 |
| ENSSSCG00000008943 | SLC4A4 | 0.021062 |
| ENSSSCG00000026264 | HSPB11 | 0.021089 |
| ENSSSCG00000004589 | RNF111 | 0.021196 |
| ENSSSCG00000003362 | CEP104 | 0.021233 |
| ENSSSCG00000011929 | SLC35A5 | 0.021258 |
| ENSSSCG00000003549 | LDLRAP1 | 0.021258 |
| ENSSSCG00000026761 | CCT8 | 0.021304 |
| ENSSSCG00000013629 | SMARCA4 | 0.021304 |
| ENSSSCG00000039546 | KNTC1 | 0.021373 |
| ENSSSCG00000037964 | GNG10 | 0.021418 |
| ENSSSCG00000016531 | C7orf49 | 0.021421 |
| ENSSSCG00000009801 | BCL7A | 0.021421 |
| ENSSSCG00000023603 | NA | 0.021577 |
| ENSSSCG00000009680 | EXTL3 | 0.021577 |
| ENSSSCG00000013233 | CELF1 | 0.021577 |
| ENSSSCG00000035671 | USP22 | 0.02169 |
| ENSSSCG00000001637 | GUCA1B | 0.02169 |
| ENSSSCG00000009625 | NA | 0.021817 |
| ENSSSCG00000017476 | MSL1 | 0.021831 |
| ENSSSCG00000023366 | OARD1 | 0.021854 |
| ENSSSCG00000003791 | SRSF11 | 0.021854 |
| ENSSSCG00000003093 | SYMPK | 0.021879 |
| ENSSSCG00000029857 | NA | 0.021971 |
| ENSSSCG00000010628 | SHOC2 | 0.021992 |
| ENSSSCG00000008414 | ERLEC1 | 0.02201 |
| ENSSSCG00000039997 | ZNF41 | 0.022203 |
| ENSSSCG00000010672 | RAB11FIP2 | 0.022203 |
| ENSSSCG00000014112 | JMY | 0.022344 |
| ENSSSCG00000003085 | CLPTM1 | 0.022525 |
| ENSSSCG00000017279 | ERN1 | 0.022577 |
| ENSSSCG00000004587 | MYO1E | 0.022585 |
| ENSSSCG00000031786 | NCDN | 0.022585 |
| ENSSSCG00000010485 | TBC1D12 | 0.022644 |
| ENSSSCG00000015882 | BAZ2B | 0.022652 |
| ENSSSCG00000020988 | NA | 0.022751 |
| ENSSSCG00000015874 | ACVR1 | 0.022794 |
| ENSSSCG00000010309 | ZSWIM8 | 0.022857 |
| ENSSSCG00000024126 | CCT5 | 0.022921 |
| ENSSSCG00000014448 | ARSI | 0.022921 |
| ENSSSCG00000023818 | NA | 0.022921 |
| ENSSSCG00000005056 | DLGAP5 | 0.023025 |
| ENSSSCG00000027447 | TMTC1 | 0.023078 |
| ENSSSCG00000030480 | DYRK1A | 0.023106 |
| ENSSSCG00000006499 | LAMTOR2 | 0.02317 |
| ENSSSCG00000012695 | INTS6L | 0.023179 |
| ENSSSCG00000017835 | CLUH | 0.023197 |
| ENSSSCG00000034105 | TMEM127 | 0.023197 |
| ENSSSCG00000017769 | RAB34 | 0.023229 |
| ENSSSCG00000008713 | KIAA0232 | 0.02325 |
| ENSSSCG00000035673 | HBP1 | 0.023267 |
| ENSSSCG00000039838 | RGS10 | 0.023267 |
| ENSSSCG00000023378 | ZNF316 | 0.023375 |
| ENSSSCG00000040446 | NA | 0.023375 |
| ENSSSCG00000004457 | DOPEY1 | 0.02345 |
| ENSSSCG00000027421 | NA | 0.023507 |
| ENSSSCG00000025565 | NA | 0.023514 |
| ENSSSCG00000025176 | NOTCH3 | 0.023532 |
| ENSSSCG00000007840 | POLR3E | 0.023554 |
| ENSSSCG00000033335 | AKIP1 | 0.023657 |
| ENSSSCG00000008352 | NA | 0.023691 |
| ENSSSCG00000022689 | GADD45B | 0.023866 |
| ENSSSCG00000014280 | NA | 0.023905 |
| ENSSSCG00000001920 | HCN4 | 0.023936 |
| ENSSSCG00000022256 | C10orf10 | 0.023943 |
| ENSSSCG00000025928 | NA | 0.024024 |
| ENSSSCG00000009540 | LIG4 | 0.024043 |
| ENSSSCG00000031680 | GGPS1 | 0.024043 |
| ENSSSCG00000012026 | ADAMTS1 | 0.024077 |
| ENSSSCG00000036236 | ELOVL6 | 0.024077 |
| ENSSSCG00000025097 | TMEM61 | 0.024077 |
| ENSSSCG00000004544 | SPG21 | 0.024077 |
| ENSSSCG00000000739 | FOXM1 | 0.024097 |
| ENSSSCG00000008443 | EPAS1 | 0.024117 |
| ENSSSCG00000038181 | CCNT1 | 0.024185 |
| ENSSSCG00000010968 | IL11RA | 0.024204 |
| ENSSSCG00000036702 | ACTN1 | 0.024216 |
| ENSSSCG00000034278 | SLC31A1 | 0.024281 |
| ENSSSCG00000016520 | CREB3L2 | 0.024337 |
| ENSSSCG00000001696 | NA | 0.02435 |
| ENSSSCG00000026392 | BSDC1 | 0.024466 |
| ENSSSCG00000014080 | HMGCR | 0.024466 |
| ENSSSCG00000038026 | RAB12 | 0.02453 |
| ENSSSCG00000012029 | BACH1 | 0.02453 |
| ENSSSCG00000016050 | INPP1 | 0.02453 |
| ENSSSCG00000016848 | EGFLAM | 0.02456 |
| ENSSSCG00000039663 | TWSG1 | 0.024567 |
| ENSSSCG00000008072 | ASPN | 0.024764 |
| ENSSSCG00000039679 | ZNF623 | 0.024773 |
| ENSSSCG00000015850 | DUSP4 | 0.024785 |
| ENSSSCG00000002838 | ZNF423 | 0.025064 |
| ENSSSCG00000017337 | ACBD4 | 0.025101 |
| ENSSSCG00000039161 | MEIS1 | 0.025101 |
| ENSSSCG00000009230 | WDFY3 | 0.025106 |
| ENSSSCG00000011810 | BCL6 | 0.025153 |
| ENSSSCG00000030561 | LMTK3 | 0.025153 |
| ENSSSCG00000022993 | TBC1D8B | 0.025404 |
| ENSSSCG00000004973 | UACA | 0.025404 |
| ENSSSCG00000023912 | ORAI2 | 0.025406 |
| ENSSSCG00000009850 | TAOK3 | 0.02541 |
| ENSSSCG00000008048 | RNPS1 | 0.025419 |
| ENSSSCG00000029165 | DOK4 | 0.025438 |
| ENSSSCG00000009794 | MLXIP | 0.025455 |
| ENSSSCG00000004755 | DLL4 | 0.025471 |
| ENSSSCG00000023178 | BATF2 | 0.025482 |
| ENSSSCG00000000765 | IL17RA | 0.025482 |
| ENSSSCG00000007786 | RNF40 | 0.025618 |
| ENSSSCG00000009962 | TFIP11 | 0.025775 |
| ENSSSCG00000024001 | PLXNA1 | 0.025782 |
| ENSSSCG00000004370 | ATG5 | 0.025782 |
| ENSSSCG00000010513 | LCOR | 0.025811 |
| ENSSSCG00000039321 | FAM222B | 0.025834 |
| ENSSSCG00000013596 | NA | 0.025899 |
| ENSSSCG00000000891 | LTA4H | 0.026065 |
| ENSSSCG00000034473 | snoU82P | 0.026196 |
| ENSSSCG00000028572 | PER3 | 0.026284 |
| ENSSSCG00000010966 | CCL19 | 0.026339 |
| ENSSSCG00000029251 | NA | 0.026351 |
| ENSSSCG00000000376 | SUOX | 0.026478 |
| ENSSSCG00000011068 | NA | 0.026554 |
| ENSSSCG00000004569 | LACTB | 0.026554 |
| ENSSSCG00000040202 | CRLS1 | 0.026734 |
| ENSSSCG00000032171 | TCF20 | 0.026793 |
| ENSSSCG00000011867 | MYLK | 0.026793 |
| ENSSSCG00000003857 | ZYG11B | 0.026823 |
| ENSSSCG00000014416 | TCERG1 | 0.026828 |
| ENSSSCG00000029449 | NA | 0.026847 |
| ENSSSCG00000011704 | WWTR1 | 0.026847 |
| ENSSSCG00000000675 | C1R | 0.026885 |
| ENSSSCG00000035253 | MAFG | 0.026885 |
| ENSSSCG00000011326 | PTH1R | 0.027069 |
| ENSSSCG00000008181 | TXNDC9 | 0.027102 |
| ENSSSCG00000002863 | LRP3 | 0.027254 |
| ENSSSCG00000011407 | RASSF1 | 0.02739 |
| ENSSSCG00000022490 | GPR83 | 0.02739 |
| ENSSSCG00000010253 | HK1 | 0.027442 |
| ENSSSCG00000035212 | KLF6 | 0.027444 |
| ENSSSCG00000010719 | NA | 0.027455 |
| ENSSSCG00000028266 | TMUB1 | 0.027501 |
| ENSSSCG00000023974 | PHF21A | 0.027523 |
| ENSSSCG00000025252 | PEX5 | 0.027846 |
| ENSSSCG00000010559 | NA | 0.02795 |
| ENSSSCG00000037591 | AMMECR1 | 0.028025 |
| ENSSSCG00000017392 | CCR10 | 0.028061 |
| ENSSSCG00000002283 | FUT8 | 0.028131 |
| ENSSSCG00000011826 | TMEM44 | 0.028241 |
| ENSSSCG00000007019 | GPAT4 | 0.028273 |
| ENSSSCG00000026780 | NA | 0.028303 |
| ENSSSCG00000031791 | ARF4 | 0.02834 |
| ENSSSCG00000011864 | UMPS | 0.02837 |
| ENSSSCG00000029949 | CD248 | 0.028376 |
| ENSSSCG00000032024 | URB1 | 0.028624 |
| ENSSSCG00000026113 | ZBTB20 | 0.028639 |
| ENSSSCG00000028036 | MAP3K10 | 0.028704 |
| ENSSSCG00000003478 | NA | 0.028775 |
| ENSSSCG00000011732 | TRIM59 | 0.028931 |
| ENSSSCG00000033037 | NA | 0.029015 |
| ENSSSCG00000037185 | NA | 0.029057 |
| ENSSSCG00000003909 | NA | 0.029057 |
| ENSSSCG00000017638 | TSPOAP1 | 0.029057 |
| ENSSSCG00000037854 | L3HYPDH | 0.029128 |
| ENSSSCG00000033521 | NA | 0.029377 |
| ENSSSCG00000007423 | UBE2C | 0.029396 |
| ENSSSCG00000015986 | HOXD1 | 0.029633 |
| ENSSSCG00000016541 | SLC35B4 | 0.029651 |
| ENSSSCG00000038811 | NA | 0.029775 |
| ENSSSCG00000007940 | NA | 0.029784 |
| ENSSSCG00000005982 | NA | 0.030074 |
| ENSSSCG00000026622 | EAF1 | 0.030085 |
| ENSSSCG00000040538 | NA | 0.030113 |
| ENSSSCG00000026499 | NMT2 | 0.03017 |
| ENSSSCG00000009431 | DGKH | 0.030191 |
| ENSSSCG00000023662 | CHST3 | 0.030212 |
| ENSSSCG00000030067 | GINM1 | 0.030213 |
| ENSSSCG00000024818 | RCAN3 | 0.030213 |
| ENSSSCG00000040929 | TPT1 | 0.030219 |
| ENSSSCG00000013661 | EIF3G | 0.030219 |
| ENSSSCG00000010446 | STAMBPL1 | 0.030219 |
| ENSSSCG00000023803 | ELK3 | 0.030219 |
| ENSSSCG00000032094 | DKK2 | 0.030219 |
| ENSSSCG00000006096 | INTS8 | 0.030219 |
| ENSSSCG00000021353 | TMX4 | 0.030219 |
| ENSSSCG00000022538 | MARK4 | 0.030226 |
| ENSSSCG00000029803 | KDM2B | 0.030383 |
| ENSSSCG00000021319 | ALG3 | 0.03041 |
| ENSSSCG00000037767 | TSFM | 0.030429 |
| ENSSSCG00000022504 | CDON | 0.0305 |
| ENSSSCG00000025855 | RNMT | 0.0305 |
| ENSSSCG00000003105 | SLC1A5 | 0.0305 |
| ENSSSCG00000029771 | NA | 0.0305 |
| ENSSSCG00000015560 | ARPC5 | 0.030553 |
| ENSSSCG00000011798 | NA | 0.030622 |
| ENSSSCG00000015603 | LPGAT1 | 0.030647 |
| ENSSSCG00000013411 | NA | 0.030792 |
| ENSSSCG00000036436 | FZD1 | 0.030795 |
| ENSSSCG00000010928 | KDM5B | 0.030795 |
| ENSSSCG00000031360 | ADO | 0.031051 |
| ENSSSCG00000000849 | NA | 0.031051 |
| ENSSSCG00000031724 | LYNX1 | 0.03116 |
| ENSSSCG00000035332 | ZNF436 | 0.03116 |
| ENSSSCG00000035904 | RPL7A | 0.03116 |
| ENSSSCG00000032031 | QARS | 0.03116 |
| ENSSSCG00000011613 | H1FX | 0.031381 |
| ENSSSCG00000010450 | LIPA | 0.031511 |
| ENSSSCG00000005975 | MTSS1 | 0.031532 |
| ENSSSCG00000003809 | JAK1 | 0.031543 |
| ENSSSCG00000040732 | WDR81 | 0.031543 |
| ENSSSCG00000040690 | LRRC61 | 0.031782 |
| ENSSSCG00000036817 | PPP4C | 0.031838 |
| ENSSSCG00000011404 | HYAL1 | 0.031897 |
| ENSSSCG00000013351 | NAV2 | 0.031989 |
| ENSSSCG00000031462 | ZNRF1 | 0.03202 |
| ENSSSCG00000003433 | PLOD1 | 0.03207 |
| ENSSSCG00000037358 | NA | 0.032079 |
| ENSSSCG00000002307 | ERH | 0.032126 |
| ENSSSCG00000007547 | ADAP1 | 0.03224 |
| ENSSSCG00000009049 | USP38 | 0.032328 |
| ENSSSCG00000013421 | NA | 0.032328 |
| ENSSSCG00000035403 | RFX2 | 0.032328 |
| ENSSSCG00000024045 | NA | 0.032384 |
| ENSSSCG00000038452 | ADAMTS17 | 0.03243 |
| ENSSSCG00000012862 | OSBPL5 | 0.032486 |
| ENSSSCG00000032146 | NA | 0.032558 |
| ENSSSCG00000027401 | OPRD1 | 0.032676 |
| ENSSSCG00000012642 | STAG2 | 0.032676 |
| ENSSSCG00000015570 | IVNS1ABP | 0.032748 |
| ENSSSCG00000024481 | NA | 0.032966 |
| ENSSSCG00000025174 | WDFY1 | 0.033005 |
| ENSSSCG00000011529 | TRNT1 | 0.033005 |
| ENSSSCG00000032203 | EPPK1 | 0.033124 |
| ENSSSCG00000022145 | RPF1 | 0.033146 |
| ENSSSCG00000022173 | ANKRD50 | 0.033264 |
| ENSSSCG00000023727 | TRIM37 | 0.033264 |
| ENSSSCG00000017215 | SLC16A5 | 0.033317 |
| ENSSSCG00000029783 | MKX | 0.033406 |
| ENSSSCG00000000370 | DGKA | 0.033458 |
| ENSSSCG00000010283 | SPOCK2 | 0.03347 |
| ENSSSCG00000032327 | TMEM169 | 0.033779 |
| ENSSSCG00000011361 | SLC26A6 | 0.033793 |
| ENSSSCG00000016498 | MKRN1 | 0.034133 |
| ENSSSCG00000031991 | GLRX2 | 0.034162 |
| ENSSSCG00000011888 | GPR156 | 0.034236 |
| ENSSSCG00000038359 | NA | 0.034284 |
| ENSSSCG00000029413 | DNMT3A | 0.034307 |
| ENSSSCG00000030697 | CAPN7 | 0.034307 |
| ENSSSCG00000012510 | ARMCX2 | 0.034319 |
| ENSSSCG00000014235 | SNCAIP | 0.034319 |
| ENSSSCG00000006752 | CSDE1 | 0.034329 |
| ENSSSCG00000032633 | NA | 0.034345 |
| ENSSSCG00000026146 | GALNT5 | 0.034399 |
| ENSSSCG00000016441 | ABCF2 | 0.034441 |
| ENSSSCG00000040288 | ARNT2 | 0.034454 |
| ENSSSCG00000004929 | PARP16 | 0.03463 |
| ENSSSCG00000032585 | NA | 0.034813 |
| ENSSSCG00000030177 | EBNA1BP2 | 0.034972 |
| ENSSSCG00000032964 | STK11IP | 0.034974 |
| ENSSSCG00000032957 | PTPN23 | 0.035243 |
| ENSSSCG00000017615 | DGKE | 0.035324 |
| ENSSSCG00000003898 | MKNK1 | 0.035372 |
| ENSSSCG00000016175 | MREG | 0.035429 |
| ENSSSCG00000031774 | NA | 0.035601 |
| ENSSSCG00000027477 | TBC1D2B | 0.035611 |
| ENSSSCG00000006644 | CERS2 | 0.035611 |
| ENSSSCG00000032606 | NA | 0.035648 |
| ENSSSCG00000004708 | TUBGCP4 | 0.035668 |
| ENSSSCG00000039854 | NA | 0.035737 |
| ENSSSCG00000031600 | SYNGR2 | 0.035893 |
| ENSSSCG00000037376 | MXRA7 | 0.035909 |
| ENSSSCG00000040624 | REXO1 | 0.036006 |
| ENSSSCG00000033019 | NA | 0.036178 |
| ENSSSCG00000010187 | TAF5L | 0.036178 |
| ENSSSCG00000016045 | PMS1 | 0.036218 |
| ENSSSCG00000023294 | KRCC1 | 0.036245 |
| ENSSSCG00000011827 | LSG1 | 0.036485 |
| ENSSSCG00000001075 | NA | 0.03673 |
| ENSSSCG00000009532 | KDELC1 | 0.036797 |
| ENSSSCG00000012965 | DRAP1 | 0.03686 |
| ENSSSCG00000036321 | TRADD | 0.037013 |
| ENSSSCG00000000869 | UTP20 | 0.037143 |
| ENSSSCG00000038132 | IRF2BP2 | 0.037143 |
| ENSSSCG00000034070 | ALPK3 | 0.037158 |
| ENSSSCG00000015985 | HOXD3 | 0.037234 |
| ENSSSCG00000040944 | SKI | 0.037235 |
| ENSSSCG00000028771 | MEF2A | 0.037265 |
| ENSSSCG00000004962 | CORO2B | 0.037269 |
| ENSSSCG00000009217 | PKD2 | 0.037282 |
| ENSSSCG00000026981 | MRPS6 | 0.037472 |
| ENSSSCG00000016705 | HOXA3 | 0.037538 |
| ENSSSCG00000006550 | ATP8B2 | 0.037569 |
| ENSSSCG00000023667 | KEAP1 | 0.037651 |
| ENSSSCG00000010058 | SNRPD3 | 0.037664 |
| ENSSSCG00000025924 | IGFBP5 | 0.03771 |
| ENSSSCG00000004647 | DTWD1 | 0.03771 |
| ENSSSCG00000032575 | ZBTB26 | 0.037724 |
| ENSSSCG00000008041 | PKD1 | 0.037854 |
| ENSSSCG00000000759 | NA | 0.03804 |
| ENSSSCG00000021106 | GRIPAP1 | 0.038308 |
| ENSSSCG00000007727 | AUTS2 | 0.03834 |
| ENSSSCG00000032062 | WDR55 | 0.038351 |
| ENSSSCG00000025675 | EEF2 | 0.038429 |
| ENSSSCG00000010523 | HOGA1 | 0.038492 |
| ENSSSCG00000001092 | TDP2 | 0.038693 |
| ENSSSCG00000040912 | SNX6 | 0.038749 |
| ENSSSCG00000004452 | PRSS35 | 0.038761 |
| ENSSSCG00000006758 | SYT6 | 0.038847 |
| ENSSSCG00000017543 | CALCOCO2 | 0.038851 |
| ENSSSCG00000008175 | CHST10 | 0.038883 |
| ENSSSCG00000009429 | TNFSF11 | 0.038935 |
| ENSSSCG00000015866 | FMNL2 | 0.038935 |
| ENSSSCG00000005621 | CDK9 | 0.039006 |
| ENSSSCG00000015802 | FAM149A | 0.03905 |
| ENSSSCG00000016659 | KIAA0895 | 0.039291 |
| ENSSSCG00000017826 | SMG6 | 0.03963 |
| ENSSSCG00000000377 | NA | 0.03964 |
| ENSSSCG00000003881 | SPATA6 | 0.039841 |
| ENSSSCG00000024672 | KLF16 | 0.039999 |
| ENSSSCG00000031510 | POMK | 0.040094 |
| ENSSSCG00000003566 | NUDC | 0.040094 |
| ENSSSCG00000039332 | SEC16B | 0.040133 |
| ENSSSCG00000021893 | ROCK1 | 0.040149 |
| ENSSSCG00000021258 | NA | 0.040235 |
| ENSSSCG00000017310 | KANSL1 | 0.040335 |
| ENSSSCG00000012622 | CXorf56 | 0.040428 |
| ENSSSCG00000037598 | SNX10 | 0.040472 |
| ENSSSCG00000016189 | AAMP | 0.04051 |
| ENSSSCG00000034441 | MRGPRF | 0.04056 |
| ENSSSCG00000039758 | NA | 0.04056 |
| ENSSSCG00000000531 | BICD1 | 0.040582 |
| ENSSSCG00000029593 | FOXN2 | 0.040594 |
| ENSSSCG00000015987 | NFE2L2 | 0.040594 |
| ENSSSCG00000008386 | KIAA1841 | 0.040596 |
| ENSSSCG00000032153 | C19orf12 | 0.040596 |
| ENSSSCG00000015097 | DDX6 | 0.040693 |
| ENSSSCG00000039876 | NA | 0.040709 |
| ENSSSCG00000001528 | RPS10 | 0.040709 |
| ENSSSCG00000007806 | RABEP2 | 0.040762 |
| ENSSSCG00000017294 | DCAF7 | 0.040768 |
| ENSSSCG00000039167 | NA | 0.040987 |
| ENSSSCG00000009979 | NEFH | 0.040987 |
| ENSSSCG00000028602 | GTPBP4 | 0.041091 |
| ENSSSCG00000006725 | TBX15 | 0.041192 |
| ENSSSCG00000030451 | LRRC14 | 0.041192 |
| ENSSSCG00000024393 | B3GNT5 | 0.041222 |
| ENSSSCG00000021229 | MAT2B | 0.041256 |
| ENSSSCG00000012961 | NA | 0.041281 |
| ENSSSCG00000001402 | NFKBIL1 | 0.041364 |
| ENSSSCG00000000383 | RNF41 | 0.041528 |
| ENSSSCG00000039875 | NKD1 | 0.041544 |
| ENSSSCG00000000087 | TAB1 | 0.041561 |
| ENSSSCG00000013879 | SLC27A1 | 0.041659 |
| ENSSSCG00000007007 | IDO1 | 0.041662 |
| ENSSSCG00000029420 | NA | 0.04192 |
| ENSSSCG00000028239 | FBXL7 | 0.042051 |
| ENSSSCG00000015498 | RC3H1 | 0.042056 |
| ENSSSCG00000017991 | PIK3R5 | 0.04216 |
| ENSSSCG00000003877 | FAF1 | 0.042161 |
| ENSSSCG00000034691 | ZEB2_AS1_1 | 0.042367 |
| ENSSSCG00000024109 | BDH1 | 0.042383 |
| ENSSSCG00000002285 | GPHN | 0.042473 |
| ENSSSCG00000010029 | DRG1 | 0.042545 |
| ENSSSCG00000032381 | LITAF | 0.042585 |
| ENSSSCG00000014882 | RSF1 | 0.042596 |
| ENSSSCG00000029326 | CCNB1 | 0.042633 |
| ENSSSCG00000004684 | SPG11 | 0.042779 |
| ENSSSCG00000039793 | NA | 0.042838 |
| ENSSSCG00000035357 | BCL2L1 | 0.043158 |
| ENSSSCG00000007901 | CIITA | 0.04322 |
| ENSSSCG00000005593 | OLFML2A | 0.04322 |
| ENSSSCG00000012743 | MTMR1 | 0.04322 |
| ENSSSCG00000013613 | ELOF1 | 0.04322 |
| ENSSSCG00000011449 | GNL3 | 0.043334 |
| ENSSSCG00000011169 | AAED1 | 0.043401 |
| ENSSSCG00000008996 | PLRG1 | 0.043433 |
| ENSSSCG00000004369 | PRDM1 | 0.043504 |
| ENSSSCG00000014071 | NA | 0.043504 |
| ENSSSCG00000010688 | MCMBP | 0.043636 |
| ENSSSCG00000010320 | NA | 0.043798 |
| ENSSSCG00000001391 | CCHCR1 | 0.044006 |
| ENSSSCG00000036091 | MORC4 | 0.044319 |
| ENSSSCG00000000559 | RASSF8 | 0.044319 |
| ENSSSCG00000013336 | CCDC34 | 0.04442 |
| ENSSSCG00000015115 | CBL | 0.044546 |
| ENSSSCG00000008406 | CCDC88A | 0.044696 |
| ENSSSCG00000011470 | ABHD6 | 0.044715 |
| ENSSSCG00000003815 | ALG6 | 0.044715 |
| ENSSSCG00000001814 | IQGAP1 | 0.044761 |
| ENSSSCG00000017694 | ACACA | 0.044823 |
| ENSSSCG00000031652 | RPGRIP1L | 0.044919 |
| ENSSSCG00000009226 | KLHL8 | 0.044919 |
| ENSSSCG00000012275 | ARAF | 0.044999 |
| ENSSSCG00000007473 | ADNP | 0.045033 |
| ENSSSCG00000014579 | TMEM41B | 0.045033 |
| ENSSSCG00000022778 | SGF29 | 0.0451 |
| ENSSSCG00000008646 | RNF144A | 0.045102 |
| ENSSSCG00000011562 | EMC3 | 0.045102 |
| ENSSSCG00000011028 | EPC1 | 0.045102 |
| ENSSSCG00000003930 | NA | 0.045102 |
| ENSSSCG00000039109 | MTERF2 | 0.045102 |
| ENSSSCG00000024136 | AMPH | 0.045127 |
| ENSSSCG00000022161 | ZNF251 | 0.045284 |
| ENSSSCG00000002917 | NFKBID | 0.045322 |
| ENSSSCG00000023595 | NA | 0.045322 |
| ENSSSCG00000037439 | TRAPPC4 | 0.045506 |
| ENSSSCG00000033892 | EVA1B | 0.045721 |
| ENSSSCG00000016981 | CPEB4 | 0.04586 |
| ENSSSCG00000030610 | FNBP4 | 0.046133 |
| ENSSSCG00000009390 | SPRYD7 | 0.046133 |
| ENSSSCG00000014274 | PDLIM4 | 0.046133 |
| ENSSSCG00000007317 | DLGAP4 | 0.046268 |
| ENSSSCG00000038895 | DCTN6 | 0.046268 |
| ENSSSCG00000039701 | DCAF5 | 0.046268 |
| ENSSSCG00000006504 | KHDC4 | 0.046268 |
| ENSSSCG00000009509 | IPO5 | 0.046268 |
| ENSSSCG00000017589 | DLX3 | 0.046268 |
| ENSSSCG00000016027 | ITGAV | 0.046288 |
| ENSSSCG00000040857 | NA | 0.046357 |
| ENSSSCG00000017743 | CRLF3 | 0.046368 |
| ENSSSCG00000016323 | COPS8 | 0.04641 |
| ENSSSCG00000015453 | PDIA4 | 0.046485 |
| ENSSSCG00000007466 | SLC9A8 | 0.046664 |
| ENSSSCG00000012882 | NA | 0.046695 |
| ENSSSCG00000024399 | EVC2 | 0.046736 |
| ENSSSCG00000027166 | BICD2 | 0.046917 |
| ENSSSCG00000004844 | MTMR10 | 0.046917 |
| ENSSSCG00000017418 | NKIRAS2 | 0.047078 |
| ENSSSCG00000015730 | NIFK | 0.047176 |
| ENSSSCG00000002701 | MON1B | 0.047466 |
| ENSSSCG00000002294 | ARG2 | 0.04756 |
| ENSSSCG00000038757 | NA | 0.047596 |
| ENSSSCG00000006872 | AGL | 0.047781 |
| ENSSSCG00000030337 | NYAP1 | 0.047781 |
| ENSSSCG00000010706 | NA | 0.047781 |
| ENSSSCG00000031942 | YES1 | 0.047781 |
| ENSSSCG00000005504 | BRINP1 | 0.047836 |
| ENSSSCG00000035267 | NA | 0.048118 |
| ENSSSCG00000028157 | CASP8 | 0.048156 |
| ENSSSCG00000030113 | SHISA2 | 0.048163 |
| ENSSSCG00000034072 | NA | 0.048163 |
| ENSSSCG00000017900 | KIF1C | 0.048163 |
| ENSSSCG00000032574 | RTTN | 0.048221 |
| ENSSSCG00000001959 | CFL2 | 0.04837 |
| ENSSSCG00000016850 | LIFR | 0.04837 |
| ENSSSCG00000011330 | NBEAL2 | 0.048483 |
| ENSSSCG00000012366 | LAS1L | 0.04866 |
| ENSSSCG00000017134 | FN3KRP | 0.048846 |
| ENSSSCG00000016432 | PRKAG2 | 0.048897 |
| ENSSSCG00000036356 | ZSCAN2 | 0.048938 |
| ENSSSCG00000001488 | GCLC | 0.049077 |
| ENSSSCG00000008478 | SOS1 | 0.04916 |
| ENSSSCG00000002690 | GAN | 0.04916 |
| ENSSSCG00000024157 | ZNF800 | 0.04916 |
| ENSSSCG00000008729 | LYAR | 0.04916 |
| ENSSSCG00000028475 | KPNA1 | 0.049261 |
| ENSSSCG00000015537 | XPR1 | 0.049405 |
| ENSSSCG00000003830 | MYSM1 | 0.049633 |
| ENSSSCG00000016998 | NPM1 | 0.049633 |
| ENSSSCG00000036775 | SLC48A1 | 0.049633 |
| ENSSSCG00000004328 | MDN1 | 0.049668 |
| ENSSSCG00000021861 | PIGT | 0.049668 |
| ENSSSCG00000015892 | PSMD14 | 0.049668 |
| ENSSSCG00000008208 | EIF2AK3 | 0.049668 |
| ENSSSCG00000023593 | AFF1 | 0.049673 |
| ENSSSCG00000025488 | MCM3 | 0.049735 |
| ENSSSCG00000021042 | BRWD1 | 0.0498 |
| ENSSSCG00000032531 | SMG1 | 0.049855 |

Gene Name “NA” indicates the gene ID was not matched to a HGNC gene name.
