# Supplementary material for: A Tale of Two Loads: Modulation of IL-1 Induced Inflammatory Responses of Meniscal Cells in Two Models of Dynamic Physiologic Loading
Source: Front Bioeng Biotechnol. 2022 Mar 1;10:837619. doi: 10.3389/fbioe.2022.837619 (PMC8921261; doi:10.3389/fbioe.2022.837619)
Supplement: Supplementary file 3 [file DataSheet7.DOCX]

**Supplemental Table 8**: 5% stretch compared to 0% stretch for outer zone cells without exogenous IL-1α stimulation.

| **Gene ID** | **Gene Name** | **Log2Fold Change** | **p-value** | **Up/Down Regulated** |
| --- | --- | --- | --- | --- |
| ENSSSCG00000012026 | ADAMTS1 | 1.783347 | 2.94E-86 | UP |
| ENSSSCG00000007477 | NFATC2 | 1.216855 | 1.81E-83 | UP |
| ENSSSCG00000010329 | ZMIZ1 | 1.489326 | 2.28E-83 | UP |
| ENSSSCG00000034114 | GPR68 | 1.021242 | 7.09E-70 | UP |
| ENSSSCG00000006917 | NA | 1.307774 | 2.03E-55 | UP |
| ENSSSCG00000036060 | RRAD | 2.189105 | 9.46E-54 | UP |
| ENSSSCG00000029482 | CHST15 | 1.00848 | 3.67E-46 | UP |
| ENSSSCG00000007079 | FLRT3 | 1.214471 | 2.01E-45 | UP |
| ENSSSCG00000038521 | CHAC1 | 1.448741 | 3.20E-44 | UP |
| ENSSSCG00000008959 | CXCL2 | 1.552611 | 1.40E-34 | UP |
| ENSSSCG00000029592 | GPRC5A | 1.744833 | 2.32E-33 | UP |
| ENSSSCG00000008957 | AMCF-II | 1.159899 | 3.90E-30 | UP |
| ENSSSCG00000004572 | NA | 1.382499 | 1.38E-29 | UP |
| ENSSSCG00000021576 | CD83 | 1.714407 | 1.65E-28 | UP |
| ENSSSCG00000021569 | MMP25 | 1.117002 | 5.34E-23 | UP |
| ENSSSCG00000036893 | PTHLH | 1.140059 | 7.90E-23 | UP |
| ENSSSCG00000018047 | FAM83G | 2.131865 | 4.59E-22 | UP |
| ENSSSCG00000035403 | RFX2 | 1.22106 | 6.70E-22 | UP |
| ENSSSCG00000006073 | OSR2 | 1.29825 | 1.89E-21 | UP |
| ENSSSCG00000029656 | NDP | 1.603463 | 3.41E-20 | UP |
| ENSSSCG00000012741 | NA | 1.060325 | 1.16E-19 | UP |
| ENSSSCG00000016140 | FZD5 | 1.264236 | 2.38E-19 | UP |
| ENSSSCG00000005992 | SHAS2 | 1.574276 | 1.51E-18 | UP |
| ENSSSCG00000014437 | PPARGC1B | 1.076237 | 2.48E-16 | UP |
| ENSSSCG00000010169 | SIPA1L2 | 1.104567 | 5.54E-16 | UP |
| ENSSSCG00000012034 | TIAM1 | 1.674421 | 8.09E-16 | UP |
| ENSSSCG00000009320 | FLT1 | 1.76697 | 2.79E-15 | UP |
| ENSSSCG00000023522 | TGM2 | 1.099835 | 2.26E-14 | UP |
| ENSSSCG00000007682 | SH2B2 | 1.26156 | 1.29E-13 | UP |
| ENSSSCG00000008953 | CXCL8 | 1.666941 | 6.70E-13 | UP |
| ENSSSCG00000000766 | CECR2 | 1.218244 | 6.79E-13 | UP |
| ENSSSCG00000008963 | AREG | 1.260481 | 7.53E-13 | UP |
| ENSSSCG00000015255 | IGSF9B | 1.537983 | 1.75E-12 | UP |
| ENSSSCG00000033397 | KCNK9 | 1.617173 | 7.58E-12 | UP |
| ENSSSCG00000033465 | HIVEP3 | 1.10683 | 2.54E-10 | UP |
| ENSSSCG00000035847 | TAL1 | 1.337724 | 4.09E-10 | UP |
| ENSSSCG00000038965 | ARC | 1.066707 | 4.57E-10 | UP |
| ENSSSCG00000040388 | ZBTB46 | 1.635099 | 5.26E-10 | UP |
| ENSSSCG00000034858 | RAP1GAP2 | 1.236436 | 8.79E-10 | UP |
| ENSSSCG00000009060 | NA | 1.046655 | 1.30E-09 | UP |
| ENSSSCG00000022592 | FIBIN | 1.235111 | 1.35E-09 | UP |
| ENSSSCG00000009148 | LEF1 | 1.911144 | 1.92E-09 | UP |
| ENSSSCG00000008311 | CYP26B1 | 1.11281 | 1.29E-08 | UP |
| ENSSSCG00000024954 | FGF1 | 1.101041 | 1.30E-08 | UP |
| ENSSSCG00000005385 | NR4A3 | 1.014826 | 5.73E-08 | UP |
| ENSSSCG00000012104 | NA | 1.885375 | 2.48E-07 | UP |
| ENSSSCG00000038126 | MGAT3 | 1.615494 | 1.74E-06 | UP |
| ENSSSCG00000033049 | KLHL41 | 1.476882 | 2.85E-06 | UP |
| ENSSSCG00000001597 | LRFN2 | 1.691867 | 3.86E-06 | UP |
| ENSSSCG00000028531 | SH3BP1 | 1.080037 | 5.00E-06 | UP |
| ENSSSCG00000002835 | TOX3 | 2.148429 | 1.24E-05 | UP |
| ENSSSCG00000003702 | GATA6 | 1.080085 | 2.55E-05 | UP |
| ENSSSCG00000023710 | REEP1 | 1.062204 | 3.02E-05 | UP |
| ENSSSCG00000003069 | KCNN4 | 1.085888 | 6.14E-05 | UP |
| ENSSSCG00000015277 | SOX13 | 1.030651 | 7.26E-05 | UP |
| ENSSSCG00000023487 | MSLNL | 2.252107 | 8.51E-05 | UP |
| ENSSSCG00000006902 | GFI1 | 1.39992 | 9.00E-05 | UP |
| ENSSSCG00000013145 | DTX4 | 1.30309 | 0.000105 | UP |
| ENSSSCG00000004332 | BACH2 | 1.659913 | 0.0006 | UP |
| ENSSSCG00000016573 | IRF5 | 1.270515 | 0.000629 | UP |
| ENSSSCG00000014950 | VSTM5 | 1.114396 | 0.000684 | UP |
| ENSSSCG00000013236 | MYBPC3 | 1.121383 | 0.001046 | UP |
| ENSSSCG00000037856 | EDN2 | 3.543117 | 0.001325 | UP |
| ENSSSCG00000026618 | CAVIN2 | 1.00577 | 0.001401 | UP |
| ENSSSCG00000009977 | RASL10A | 2.510399 | 0.002533 | UP |
| ENSSSCG00000011721 | P2RY1 | 1.056893 | 0.002923 | UP |
| ENSSSCG00000003988 | NA | 1.31022 | 0.003926 | UP |
| ENSSSCG00000017470 | TNS4 | 1.102221 | 0.004239 | UP |
| ENSSSCG00000039761 | MYCL | 1.180587 | 0.004512 | UP |
| ENSSSCG00000029365 | MSI1 | 2.02777 | 0.004697 | UP |
| ENSSSCG00000035074 | FOXO6 | 1.802812 | 0.006967 | UP |
| ENSSSCG00000008768 | ARAP2 | 1.044443 | 0.008642 | UP |
| ENSSSCG00000027365 | WNT7B | 1.820967 | 0.008944 | UP |
| ENSSSCG00000034015 | NA | 4.03766 | 0.01035 | UP |
| ENSSSCG00000022833 | FGF16 | 2.144652 | 0.011198 | UP |
| ENSSSCG00000028015 | GAL3ST3 | 1.592598 | 0.011586 | UP |
| ENSSSCG00000003832 | TACSTD2 | 1.562013 | 0.012859 | UP |
| ENSSSCG00000016780 | CTNND2 | 1.018395 | 0.013985 | UP |
| ENSSSCG00000017411 | KCNH4 | 1.15245 | 0.01788 | UP |
| ENSSSCG00000023921 | VSTM2B | 1.670874 | 0.019487 | UP |
| ENSSSCG00000009648 | NEFM | 1.930462 | 0.020016 | UP |
| ENSSSCG00000029388 | PDE2A | 1.424303 | 0.020474 | UP |
| ENSSSCG00000015584 | PROX1 | 1.078563 | 0.02219 | UP |
| ENSSSCG00000020953 | NA | 2.357646 | 0.025209 | UP |
| ENSSSCG00000035598 | EDN1 | 1.354441 | 0.025307 | UP |
| ENSSSCG00000025992 | NA | 1.104205 | 0.026789 | UP |
| ENSSSCG00000015935 | CCDC173 | 1.350155 | 0.027142 | UP |
| ENSSSCG00000025416 | CAMKV | 1.627573 | 0.028804 | UP |
| ENSSSCG00000032098 | NA | 1.753406 | 0.029512 | UP |
| ENSSSCG00000016215 | NA | 1.099367 | 0.030286 | UP |
| ENSSSCG00000009477 | EDNRB | 1.015405 | 0.032077 | UP |
| ENSSSCG00000010584 | MFSD13A | 1.035989 | 0.033479 | UP |
| ENSSSCG00000038677 | GJB3 | 1.015827 | 0.035827 | UP |
| ENSSSCG00000009045 | HHIP | 1.103662 | 0.036875 | UP |
| ENSSSCG00000017995 | USP43 | 3.023664 | 0.038596 | UP |
| ENSSSCG00000016855 | FYB1 | 1.914204 | 0.039469 | UP |
| ENSSSCG00000036157 | BARX2 | 3.52327 | 0.039469 | UP |
| ENSSSCG00000005216 | NA | 1.100677 | 0.040393 | UP |
| ENSSSCG00000017734 | RHBDL3 | 1.404278 | 0.040819 | UP |
| ENSSSCG00000036865 | NA | 1.256809 | 0.040939 | UP |
| ENSSSCG00000009621 | PHYHIP | 1.175592 | 0.042792 | UP |
| ENSSSCG00000017552 | NXPH3 | 1.267102 | 0.042826 | UP |
| ENSSSCG00000039283 | SIX3 | 1.272492 | 0.043156 | UP |
| ENSSSCG00000010747 | C10orf90 | 1.217732 | 0.045994 | UP |
| ENSSSCG00000003736 | ASXL3 | 2.210452 | 0.048227 | UP |
| ENSSSCG00000027426 | BCL3 | -2.0702 | 3.47E-111 | DOWN |
| ENSSSCG00000013432 | MIDN | -1.14909 | 1.13E-77 | DOWN |
| ENSSSCG00000036136 | BHLHE40 | -1.46634 | 8.26E-77 | DOWN |
| ENSSSCG00000039473 | NA | -1.27996 | 4.39E-63 | DOWN |
| ENSSSCG00000032861 | NUAK1 | -1.03181 | 1.56E-59 | DOWN |
| ENSSSCG00000004948 | SMAD6 | -1.37252 | 6.41E-53 | DOWN |
| ENSSSCG00000021646 | KLF9 | -1.23696 | 1.71E-52 | DOWN |
| ENSSSCG00000017046 | EBF1 | -1.09689 | 3.61E-52 | DOWN |
| ENSSSCG00000027480 | KLF10 | -1.28626 | 4.41E-51 | DOWN |
| ENSSSCG00000002392 | IRF2BPL | -1.21849 | 1.17E-46 | DOWN |
| ENSSSCG00000025826 | BOC | -1.13585 | 7.08E-43 | DOWN |
| ENSSSCG00000032613 | SNAI1 | -1.2492 | 2.74E-42 | DOWN |
| ENSSSCG00000034191 | SOX6 | -1.4187 | 1.84E-36 | DOWN |
| ENSSSCG00000013735 | JUNB | -1.26612 | 7.36E-35 | DOWN |
| ENSSSCG00000035400 | YPEL2 | -1.30505 | 3.83E-33 | DOWN |
| ENSSSCG00000011672 | RASA2 | -1.32984 | 2.44E-31 | DOWN |
| ENSSSCG00000039568 | SNAI2 | -1.58155 | 7.99E-31 | DOWN |
| ENSSSCG00000031321 | NR4A1 | -2.25224 | 2.60E-29 | DOWN |
| ENSSSCG00000015882 | BAZ2B | -1.02057 | 4.11E-29 | DOWN |
| ENSSSCG00000035940 | SPSB1 | -1.57279 | 9.62E-28 | DOWN |
| ENSSSCG00000020705 | MAP3K8 | -1.40377 | 5.53E-27 | DOWN |
| ENSSSCG00000016841 | SLC1A3 | -1.21269 | 2.87E-26 | DOWN |
| ENSSSCG00000037241 | RGS2 | -1.28096 | 4.11E-25 | DOWN |
| ENSSSCG00000032936 | PIM3 | -1.27321 | 4.85E-24 | DOWN |
| ENSSSCG00000006729 | FAM46C | -1.62362 | 1.11E-21 | DOWN |
| ENSSSCG00000037468 | GNE | -1.24001 | 1.07E-19 | DOWN |
| ENSSSCG00000030511 | LGR5 | -1.11742 | 5.17E-18 | DOWN |
| ENSSSCG00000036742 | KLF15 | -2.73209 | 1.37E-15 | DOWN |
| ENSSSCG00000009630 | EGR3 | -1.73663 | 1.82E-14 | DOWN |
| ENSSSCG00000006530 | EFNA1 | -1.42004 | 3.97E-14 | DOWN |
| ENSSSCG00000015550 | RGS16 | -1.51017 | 1.07E-13 | DOWN |
| ENSSSCG00000008648 | RSAD2 | -2.52969 | 1.27E-11 | DOWN |
| ENSSSCG00000034184 | NA | -2.1443 | 4.03E-11 | DOWN |
| ENSSSCG00000008647 | CMPK2 | -1.80628 | 1.53E-10 | DOWN |
| ENSSSCG00000010451 | IFIT2 | -1.68878 | 3.75E-10 | DOWN |
| ENSSSCG00000008919 | EPHA5 | -1.14345 | 5.06E-10 | DOWN |
| ENSSSCG00000027628 | IL6R | -1.13894 | 7.47E-10 | DOWN |
| ENSSSCG00000036755 | FAM46B | -1.21379 | 7.68E-10 | DOWN |
| ENSSSCG00000010224 | EGR2 | -1.19214 | 4.88E-09 | DOWN |
| ENSSSCG00000011876 | DTX3L | -1.55145 | 9.91E-09 | DOWN |
| ENSSSCG00000011874 | PARP14 | -1.48607 | 1.60E-08 | DOWN |
| ENSSSCG00000035284 | BMF | -1.10073 | 2.68E-08 | DOWN |
| ENSSSCG00000023178 | BATF2 | -2.14946 | 5.56E-08 | DOWN |
| ENSSSCG00000010452 | IFIT1 | -1.32583 | 1.28E-07 | DOWN |
| ENSSSCG00000024973 | NA | -1.22815 | 1.07E-06 | DOWN |
| ENSSSCG00000007508 | ZBP1 | -1.27096 | 1.07E-06 | DOWN |
| ENSSSCG00000012076 | MX2 | -1.5867 | 1.09E-06 | DOWN |
| ENSSSCG00000030408 | DDX58 | -1.14075 | 1.69E-06 | DOWN |
| ENSSSCG00000032474 | CXCL10 | -2.30684 | 2.46E-06 | DOWN |
| ENSSSCG00000000148 | NA | -1.3948 | 3.63E-06 | DOWN |
| ENSSSCG00000020906 | TNFSF10 | -1.64948 | 4.04E-06 | DOWN |
| ENSSSCG00000011239 | NA | -1.17576 | 2.93E-05 | DOWN |
| ENSSSCG00000009720 | DDX60 | -1.01047 | 0.000486 | DOWN |
| ENSSSCG00000033089 | NA | -1.35286 | 0.001077 | DOWN |
| ENSSSCG00000039751 | NLRC5 | -1.04383 | 0.003351 | DOWN |
| ENSSSCG00000006038 | ZFPM2 | -1.46097 | 0.010158 | DOWN |
| ENSSSCG00000007146 | SIGLEC1 | -2.80131 | 0.013856 | DOWN |
| ENSSSCG00000010761 | STK32C | -1.36658 | 0.015302 | DOWN |
| ENSSSCG00000020300 | U6 | -2.35536 | 0.015944 | DOWN |
| ENSSSCG00000035388 | C16orf46 | -1.309 | 0.016018 | DOWN |
| ENSSSCG00000023627 | LYPD6 | -1.27882 | 0.018818 | DOWN |
| ENSSSCG00000013385 | INSC | -1.82173 | 0.029725 | DOWN |
| ENSSSCG00000040648 | CCL11 | -1.34308 | 0.038153 | DOWN |
| ENSSSCG00000003950 | TIE1 | -1.61146 | 0.041675 | DOWN |
| ENSSSCG00000032446 | C1orf116 | -1.1426 | 0.041788 | DOWN |

Gene Name “NA” indicates the gene ID was not matched to a HGNC gene name.
