# Supplementary material for: A Tale of Two Loads: Modulation of IL-1 Induced Inflammatory Responses of Meniscal Cells in Two Models of Dynamic Physiologic Loading
Source: Front Bioeng Biotechnol. 2022 Mar 1;10:837619. doi: 10.3389/fbioe.2022.837619 (PMC8921261; doi:10.3389/fbioe.2022.837619)
Supplement: Supplementary file 4 [file DataSheet6.DOCX]

**Supplemental Table 6**: 5% stretch compared to 0% stretch for inner zone cells with exogenous IL-1α stimulation.

| **Gene ID** | **Gene Name** | **Log2Fold Change** | **p-value** | **Up/Down Regulated** |
| --- | --- | --- | --- | --- |
| ENSSSCG00000007477 | NFATC2 | 2.104399262 | 1.91E-210 | Up |
| ENSSSCG00000034114 | GPR68 | 1.37345237 | 6.19E-113 | Up |
| ENSSSCG00000001081 | SOX4 | 1.469651161 | 4.04E-83 | Up |
| ENSSSCG00000036060 | RRAD | 2.522350434 | 3.10E-78 | Up |
| ENSSSCG00000004142 | CITED2 | 1.398949251 | 8.51E-78 | Up |
| ENSSSCG00000004856 | NFATC1 | 1.256014013 | 8.15E-53 | Up |
| ENSSSCG00000010329 | ZMIZ1 | 1.144667471 | 6.80E-52 | Up |
| ENSSSCG00000006105 | GEM | 1.079162017 | 8.83E-52 | Up |
| ENSSSCG00000038965 | ARC | 2.902728248 | 1.30E-51 | Up |
| ENSSSCG00000008437 | SOCS5 | 1.153981424 | 4.91E-51 | Up |
| ENSSSCG00000017475 | RARA | 1.116996854 | 1.14E-47 | Up |
| ENSSSCG00000015370 | TWIST1 | 1.013844482 | 1.77E-47 | Up |
| ENSSSCG00000004201 | TMEM200A | 1.300424793 | 2.12E-47 | Up |
| ENSSSCG00000009152 | SGMS2 | 1.091544776 | 1.37E-40 | Up |
| ENSSSCG00000010816 | TGFB2 | 1.045106102 | 2.93E-40 | Up |
| ENSSSCG00000031875 | ZNF469 | 1.365405063 | 7.75E-40 | Up |
| ENSSSCG00000038521 | CHAC1 | 1.130409342 | 2.21E-39 | Up |
| ENSSSCG00000016067 | STK17B | 1.239981175 | 3.77E-39 | Up |
| ENSSSCG00000012026 | ADAMTS1 | 1.07962677 | 4.10E-34 | Up |
| ENSSSCG00000014909 | NA | 1.249157815 | 5.12E-34 | Up |
| ENSSSCG00000014437 | PPARGC1B | 1.86493733 | 2.20E-33 | Up |
| ENSSSCG00000026108 | CDC42EP1 | 1.063826083 | 2.84E-33 | Up |
| ENSSSCG00000001061 | JARID2 | 1.442967697 | 6.46E-32 | Up |
| ENSSSCG00000021569 | MMP25 | 1.317921324 | 1.08E-31 | Up |
| ENSSSCG00000014235 | SNCAIP | 1.15681707 | 2.17E-30 | Up |
| ENSSSCG00000001620 | MDFI | 1.312674671 | 1.84E-29 | Up |
| ENSSSCG00000007249 | NOL4L | 1.288513985 | 7.77E-29 | Up |
| ENSSSCG00000009370 | FOXO1 | 1.012164526 | 2.29E-27 | Up |
| ENSSSCG00000010600 | CALHM2 | 1.199427272 | 1.01E-26 | Up |
| ENSSSCG00000039780 | RTN4RL1 | 1.046799402 | 7.43E-26 | Up |
| ENSSSCG00000012741 | NA | 1.06466724 | 1.02E-24 | Up |
| ENSSSCG00000040388 | ZBTB46 | 2.194471587 | 3.13E-23 | Up |
| ENSSSCG00000033327 | PDGFB | 2.211901925 | 9.40E-22 | Up |
| ENSSSCG00000020864 | VDR | 1.020751879 | 8.64E-21 | Up |
| ENSSSCG00000016983 | STC2 | 1.323798985 | 6.53E-20 | Up |
| ENSSSCG00000002847 | GPT2 | 1.343352603 | 1.38E-19 | Up |
| ENSSSCG00000028529 | REM1 | 1.035624482 | 2.10E-19 | Up |
| ENSSSCG00000000455 | LRIG3 | 1.019842817 | 1.48E-18 | Up |
| ENSSSCG00000005992 | SHAS2 | 1.42083417 | 6.38E-17 | Up |
| ENSSSCG00000018047 | FAM83G | 2.15936304 | 8.68E-17 | Up |
| ENSSSCG00000025260 | CARD10 | 1.089127969 | 1.08E-16 | Up |
| ENSSSCG00000009071 | JADE1 | 1.155520772 | 2.26E-16 | Up |
| ENSSSCG00000009580 | S1PR3 | 1.535762584 | 4.28E-16 | Up |
| ENSSSCG00000040013 | MTUS1 | 1.243284341 | 4.25E-15 | Up |
| ENSSSCG00000006290 | SLC19A2 | 1.025290525 | 2.56E-14 | Up |
| ENSSSCG00000000936 | SLC6A15 | 1.032621952 | 4.41E-14 | Up |
| ENSSSCG00000031616 | FOSB | 1.933974071 | 1.43E-13 | Up |
| ENSSSCG00000016573 | IRF5 | 1.171584337 | 6.64E-13 | Up |
| ENSSSCG00000014034 | N4BP3 | 1.226979973 | 7.67E-13 | Up |
| ENSSSCG00000029656 | NDP | 1.574287001 | 1.45E-12 | Up |
| ENSSSCG00000009676 | ZNF395 | 1.522951172 | 1.96E-11 | Up |
| ENSSSCG00000017749 | NA | 1.024479803 | 5.80E-11 | Up |
| ENSSSCG00000036555 | PRDM8 | 1.414964061 | 2.69E-10 | Up |
| ENSSSCG00000001621 | TFEB | 1.190575678 | 3.01E-10 | Up |
| ENSSSCG00000035448 | SS18L1 | 1.023040143 | 4.16E-10 | Up |
| ENSSSCG00000009060 | NA | 1.270823069 | 6.03E-10 | Up |
| ENSSSCG00000016589 | LRRC4 | 1.347019835 | 1.01E-09 | Up |
| ENSSSCG00000040607 | MAF | 1.034277308 | 1.30E-09 | Up |
| ENSSSCG00000036437 | NOG | 1.307097223 | 4.25E-09 | Up |
| ENSSSCG00000029849 | S1PR1 | 1.817407162 | 4.54E-09 | Up |
| ENSSSCG00000011538 | LMCD1 | 2.157266505 | 7.09E-09 | Up |
| ENSSSCG00000035077 | INHBA | 1.045020227 | 4.07E-08 | Up |
| ENSSSCG00000007572 | LFNG | 1.177043972 | 5.98E-08 | Up |
| ENSSSCG00000001710 | RUNX2 | 1.670981057 | 6.22E-08 | Up |
| ENSSSCG00000014347 | PROB1 | 1.339635198 | 7.29E-08 | Up |
| ENSSSCG00000021483 | NPBWR1 | 1.592123468 | 1.91E-07 | Up |
| ENSSSCG00000003256 | PRKCG | 1.290717306 | 3.38E-07 | Up |
| ENSSSCG00000012104 | NA | 1.474289647 | 4.09E-07 | Up |
| ENSSSCG00000039255 | SNORD44 | 1.988809373 | 5.88E-07 | Up |
| ENSSSCG00000012151 | NHS | 1.435039548 | 5.97E-07 | Up |
| ENSSSCG00000014598 | PPFIBP2 | 1.093397922 | 7.34E-07 | Up |
| ENSSSCG00000014395 | PCDH12 | 1.530128727 | 1.03E-06 | Up |
| ENSSSCG00000011294 | FAM198A | 1.232064707 | 1.10E-06 | Up |
| ENSSSCG00000031706 | MICALCL | 1.317511696 | 1.17E-06 | Up |
| ENSSSCG00000006286 | NA | 1.613275884 | 1.45E-06 | Up |
| ENSSSCG00000033397 | KCNK9 | 2.631068639 | 2.56E-06 | Up |
| ENSSSCG00000038643 | KLF11 | 2.080790869 | 4.25E-06 | Up |
| ENSSSCG00000022289 | PCDH1 | 1.091489292 | 4.39E-06 | Up |
| ENSSSCG00000004109 | ZC3H12D | 1.644489991 | 6.43E-06 | Up |
| ENSSSCG00000010222 | ZNF365 | 2.133787492 | 6.56E-06 | Up |
| ENSSSCG00000037856 | EDN2 | 3.43714492 | 8.08E-06 | Up |
| ENSSSCG00000006780 | WNT2B | 1.563018604 | 8.45E-06 | Up |
| ENSSSCG00000036364 | EGR4 | 1.764654519 | 1.10E-05 | Up |
| ENSSSCG00000032620 | PLCXD2 | 1.139874182 | 1.51E-05 | Up |
| ENSSSCG00000008230 | ATOH8 | 1.24301172 | 1.56E-05 | Up |
| ENSSSCG00000030337 | NYAP1 | 1.76331601 | 1.77E-05 | Up |
| ENSSSCG00000031764 | NA | 1.093514908 | 1.79E-05 | Up |
| ENSSSCG00000040875 | ZFPM1 | 1.138540248 | 1.82E-05 | Up |
| ENSSSCG00000002648 | CBFA2T3 | 1.126006719 | 1.93E-05 | Up |
| ENSSSCG00000006902 | GFI1 | 1.565311032 | 2.10E-05 | Up |
| ENSSSCG00000023848 | C8orf46 | 1.600427491 | 3.57E-05 | Up |
| ENSSSCG00000008689 | ZFYVE28 | 1.363307964 | 6.00E-05 | Up |
| ENSSSCG00000028725 | TMEM102 | 1.080055575 | 6.65E-05 | Up |
| ENSSSCG00000011071 | THNSL1 | 1.303509976 | 6.74E-05 | Up |
| ENSSSCG00000002440 | NA | 1.343568685 | 7.13E-05 | Up |
| ENSSSCG00000034632 | PDXP | 1.17129645 | 0.000140447 | Up |
| ENSSSCG00000038677 | GJB3 | 1.170227802 | 0.000152311 | Up |
| ENSSSCG00000009729 | ZNF84 | 1.035905387 | 0.000220429 | Up |
| ENSSSCG00000038126 | MGAT3 | 1.249116214 | 0.000243809 | Up |
| ENSSSCG00000009278 | FGF9 | 1.661769382 | 0.000270606 | Up |
| ENSSSCG00000012153 | RAI2 | 5.059725307 | 0.000394903 | Up |
| ENSSSCG00000035074 | FOXO6 | 2.358853363 | 0.00039974 | Up |
| ENSSSCG00000038290 | RNF182 | 1.449591822 | 0.000410311 | Up |
| ENSSSCG00000006383 | VANGL2 | 1.098113127 | 0.000567007 | Up |
| ENSSSCG00000014198 | NA | 1.229809165 | 0.00057509 | Up |
| ENSSSCG00000003069 | KCNN4 | 1.139330803 | 0.000608973 | Up |
| ENSSSCG00000006288 | SELP | 1.522967878 | 0.000623845 | Up |
| ENSSSCG00000030016 | PDE9A | 1.317287868 | 0.000638979 | Up |
| ENSSSCG00000034473 | snoU82P | 1.321213917 | 0.000675189 | Up |
| ENSSSCG00000001473 | COL11A2 | 1.021920638 | 0.000698157 | Up |
| ENSSSCG00000026837 | SNORD26 | 1.448220037 | 0.000731144 | Up |
| ENSSSCG00000031232 | SNORD60 | 2.357447292 | 0.000868 | Up |
| ENSSSCG00000028807 | SNORA16 | 1.292621789 | 0.000948897 | Up |
| ENSSSCG00000025160 | DPF1 | 1.082666218 | 0.001080098 | Up |
| ENSSSCG00000005975 | MTSS1 | 1.033652268 | 0.001190465 | Up |
| ENSSSCG00000002507 | BCL11B | 1.780893787 | 0.001292499 | Up |
| ENSSSCG00000001597 | LRFN2 | 1.596110022 | 0.001404523 | Up |
| ENSSSCG00000009953 | NA | 1.052567805 | 0.001528939 | Up |
| ENSSSCG00000022719 | RAB20 | 1.403460157 | 0.001570441 | Up |
| ENSSSCG00000017112 | IRX4 | 1.037078157 | 0.00166438 | Up |
| ENSSSCG00000005485 | AMBP | 2.363674023 | 0.001700553 | Up |
| ENSSSCG00000003135 | KCNJ14 | 1.19630868 | 0.001753142 | Up |
| ENSSSCG00000006758 | SYT6 | 2.651669643 | 0.001815697 | Up |
| ENSSSCG00000018229 | SNORD74 | 1.143337559 | 0.001894288 | Up |
| ENSSSCG00000035456 | WWC1 | 1.170986916 | 0.003130529 | Up |
| ENSSSCG00000024109 | BDH1 | 1.168706545 | 0.003172739 | Up |
| ENSSSCG00000025504 | SNORA55 | 2.675313344 | 0.003273174 | Up |
| ENSSSCG00000016705 | HOXA3 | 1.525585958 | 0.00330557 | Up |
| ENSSSCG00000017411 | KCNH4 | 1.092349532 | 0.003306396 | Up |
| ENSSSCG00000036261 | CROCC2 | 1.014384134 | 0.003443553 | Up |
| ENSSSCG00000019894 | SNORA62 | 1.313453144 | 0.003892668 | Up |
| ENSSSCG00000032327 | TMEM169 | 1.087306059 | 0.004188627 | Up |
| ENSSSCG00000019069 | SNORD78 | 1.082402257 | 0.004752149 | Up |
| ENSSSCG00000037195 | FOXF2 | 1.184769094 | 0.006044111 | Up |
| ENSSSCG00000019918 | SNORA2 | 2.764191496 | 0.006122232 | Up |
| ENSSSCG00000033718 | XIST_intron | 1.185126791 | 0.006184904 | Up |
| ENSSSCG00000009709 | MFAP3L | 2.298738953 | 0.006385303 | Up |
| ENSSSCG00000010475 | CYP26A1 | 1.358482932 | 0.006499794 | Up |
| ENSSSCG00000007115 | THBD | 1.246077765 | 0.006904835 | Up |
| ENSSSCG00000011579 | PPARG | 1.877176069 | 0.007546626 | Up |
| ENSSSCG00000038732 | AGAP2 | 1.410963509 | 0.007984749 | Up |
| ENSSSCG00000040446 | NA | 2.165938925 | 0.008696015 | Up |
| ENSSSCG00000015281 | PLEKHA6 | 1.262293256 | 0.008754165 | Up |
| ENSSSCG00000039946 | CD34 | 1.626169024 | 0.008925108 | Up |
| ENSSSCG00000000665 | RIMKLB | 1.251837769 | 0.009285432 | Up |
| ENSSSCG00000038600 | PRSS8 | 1.830737194 | 0.010452292 | Up |
| ENSSSCG00000000906 | TMCC3 | 1.114915918 | 0.011465581 | Up |
| ENSSSCG00000040720 | SLC26A9 | 1.737396255 | 0.011536425 | Up |
| ENSSSCG00000036787 | APOLD1 | 1.109352519 | 0.014036566 | Up |
| ENSSSCG00000027365 | WNT7B | 1.194387941 | 0.014226865 | Up |
| ENSSSCG00000016887 | ITGA2 | 1.441056799 | 0.015909935 | Up |
| ENSSSCG00000000584 | SLCO1A2 | 2.459802157 | 0.016365654 | Up |
| ENSSSCG00000019093 | SNORD16 | 2.231999409 | 0.018433404 | Up |
| ENSSSCG00000026098 | CMYA5 | 1.027181269 | 0.018844284 | Up |
| ENSSSCG00000016941 | RNF180 | 1.337395847 | 0.019177716 | Up |
| ENSSSCG00000015935 | CCDC173 | 1.554334261 | 0.022668466 | Up |
| ENSSSCG00000019428 | snoR38 | 1.441559622 | 0.023488438 | Up |
| ENSSSCG00000013931 | GMIP | 2.256970773 | 0.025163319 | Up |
| ENSSSCG00000020934 | NA | 1.055579775 | 0.025780696 | Up |
| ENSSSCG00000034283 | NA | 1.651860959 | 0.026052169 | Up |
| ENSSSCG00000029227 | LDB2 | 1.295038984 | 0.027739268 | Up |
| ENSSSCG00000009824 | RAD9B | 1.35652967 | 0.029758908 | Up |
| ENSSSCG00000040136 | SNORA11 | 1.262663278 | 0.030197456 | Up |
| ENSSSCG00000017789 | ABHD15 | 1.190086543 | 0.031711933 | Up |
| ENSSSCG00000007385 | KCNS1 | 1.453152979 | 0.032802445 | Up |
| ENSSSCG00000040031 | SMIM32 | 2.861407383 | 0.033838283 | Up |
| ENSSSCG00000019707 | SNORA75 | 1.671015187 | 0.034345742 | Up |
| ENSSSCG00000013760 | NA | 1.004342808 | 0.035128403 | Up |
| ENSSSCG00000008702 | DOK7 | 1.001593049 | 0.035717182 | Up |
| ENSSSCG00000033997 | NA | 1.709350134 | 0.035875563 | Up |
| ENSSSCG00000004469 | LCA5 | 1.451390122 | 0.03599942 | Up |
| ENSSSCG00000019323 | SNORA13 | 1.874039322 | 0.036620735 | Up |
| ENSSSCG00000027032 | SNORD31 | 1.007846342 | 0.036632735 | Up |
| ENSSSCG00000030368 | HSPA1L | 1.62214209 | 0.037289377 | Up |
| ENSSSCG00000006172 | PI15 | 3.268002817 | 0.037880915 | Up |
| ENSSSCG00000031537 | HAND2 | 2.116052073 | 0.038580658 | Up |
| ENSSSCG00000031345 | NA | 2.015669018 | 0.040972406 | Up |
| ENSSSCG00000022692 | RINL | 1.254417247 | 0.041621217 | Up |
| ENSSSCG00000019644 | SNORD47 | 1.430075858 | 0.041653015 | Up |
| ENSSSCG00000015200 | ESAM | 1.783600959 | 0.042242966 | Up |
| ENSSSCG00000013769 | C19orf67 | 1.865810749 | 0.043701743 | Up |
| ENSSSCG00000024403 | PRRT1 | 1.001252613 | 0.04429204 | Up |
| ENSSSCG00000012658 | RAB33A | 1.320790352 | 0.045099534 | Up |
| ENSSSCG00000029365 | MSI1 | 1.522613541 | 0.045287628 | Up |
| ENSSSCG00000032606 | NA | 1.300125025 | 0.04856325 | Up |
| ENSSSCG00000012890 | TCIRG1 | -1.773555251 | 1.02E-114 | Down |
| ENSSSCG00000025206 | RNF19B | -1.846325661 | 8.12E-109 | Down |
| ENSSSCG00000016438 | NUB1 | -1.27903491 | 2.99E-104 | Down |
| ENSSSCG00000025593 | NA | -1.172430911 | 1.13E-97 | Down |
| ENSSSCG00000024166 | SLC2A6 | -1.762287508 | 1.86E-96 | Down |
| ENSSSCG00000031380 | NA | -1.480219092 | 3.34E-95 | Down |
| ENSSSCG00000013551 | C3 | -3.627295621 | 5.21E-95 | Down |
| ENSSSCG00000040648 | CCL11 | -3.722032129 | 9.86E-94 | Down |
| ENSSSCG00000035400 | YPEL2 | -1.875043466 | 2.52E-90 | Down |
| ENSSSCG00000000396 | STAT2 | -1.48850336 | 5.80E-88 | Down |
| ENSSSCG00000001463 | PSMB9 | -2.561053427 | 1.66E-87 | Down |
| ENSSSCG00000004897 | ZCCHC2 | -1.676552907 | 3.49E-85 | Down |
| ENSSSCG00000010261 | PPA1 | -1.233337333 | 3.49E-85 | Down |
| ENSSSCG00000040887 | PAPD5 | -1.572410101 | 6.22E-81 | Down |
| ENSSSCG00000027806 | SAMHD1 | -1.557150882 | 2.44E-78 | Down |
| ENSSSCG00000001233 | TRIM26 | -1.537337133 | 2.65E-78 | Down |
| ENSSSCG00000025618 | TAP1 | -2.197217435 | 1.05E-77 | Down |
| ENSSSCG00000008123 | ARID5A | -2.536583977 | 2.76E-77 | Down |
| ENSSSCG00000026951 | PSMB8 | -1.55608836 | 4.62E-72 | Down |
| ENSSSCG00000032408 | CASP7 | -1.045286685 | 5.06E-72 | Down |
| ENSSSCG00000016502 | PARP12 | -1.815568364 | 5.58E-72 | Down |
| ENSSSCG00000021383 | CGAS | -2.263433652 | 2.07E-70 | Down |
| ENSSSCG00000005724 | SETX | -1.887657366 | 1.15E-69 | Down |
| ENSSSCG00000017420 | CNP | -2.156918721 | 2.10E-69 | Down |
| ENSSSCG00000004053 | TAGAP | -2.56951224 | 4.03E-68 | Down |
| ENSSSCG00000004657 | CEP152 | -1.457552174 | 1.06E-67 | Down |
| ENSSSCG00000035078 | CD40 | -1.300499324 | 1.12E-67 | Down |
| ENSSSCG00000016512 | ZC3HAV1 | -1.220438807 | 2.70E-67 | Down |
| ENSSSCG00000027426 | BCL3 | -1.321460161 | 2.54E-66 | Down |
| ENSSSCG00000015782 | IRF2 | -2.536267826 | 2.62E-66 | Down |
| ENSSSCG00000022011 | NMI | -1.242231261 | 1.48E-63 | Down |
| ENSSSCG00000001394 | NA | -1.10216985 | 1.35E-62 | Down |
| ENSSSCG00000024219 | TIGAR | -1.764253721 | 2.24E-62 | Down |
| ENSSSCG00000024973 | NA | -3.666610089 | 2.94E-62 | Down |
| ENSSSCG00000029438 | SESN2 | -1.273994476 | 1.36E-61 | Down |
| ENSSSCG00000035634 | NA | -1.460211269 | 1.52E-61 | Down |
| ENSSSCG00000002311 | SUSD6 | -1.094142941 | 8.06E-61 | Down |
| ENSSSCG00000037536 | SLC25A28 | -1.233327021 | 9.04E-61 | Down |
| ENSSSCG00000017723 | CCL2 | -1.188265105 | 2.05E-60 | Down |
| ENSSSCG00000007007 | IDO1 | -2.160887427 | 1.53E-59 | Down |
| ENSSSCG00000002841 | N4BP1 | -1.486141401 | 3.49E-59 | Down |
| ENSSSCG00000037572 | EPSTI1 | -2.622526117 | 7.79E-59 | Down |
| ENSSSCG00000017087 | GM2A | -1.507195483 | 1.56E-58 | Down |
| ENSSSCG00000013114 | SLC15A3 | -1.365977249 | 2.11E-57 | Down |
| ENSSSCG00000001509 | DAXX | -1.276348401 | 2.86E-57 | Down |
| ENSSSCG00000015550 | RGS16 | -1.707680002 | 3.72E-57 | Down |
| ENSSSCG00000011727 | PTX3 | -1.209229956 | 1.88E-56 | Down |
| ENSSSCG00000006209 | VCPIP1 | -1.093564473 | 1.09E-54 | Down |
| ENSSSCG00000002392 | IRF2BPL | -1.408754 | 3.08E-54 | Down |
| ENSSSCG00000011251 | MYD88 | -1.00409864 | 3.14E-54 | Down |
| ENSSSCG00000032360 | PANX1 | -1.608558116 | 4.75E-54 | Down |
| ENSSSCG00000011465 | NA | -1.045858722 | 1.14E-52 | Down |
| ENSSSCG00000006127 | NBN | -1.309206567 | 2.07E-52 | Down |
| ENSSSCG00000016261 | SP110 | -1.335988777 | 2.52E-52 | Down |
| ENSSSCG00000015549 | RNASEL | -2.363071256 | 4.34E-52 | Down |
| ENSSSCG00000009469 | ACOD1 | -2.99775422 | 7.99E-52 | Down |
| ENSSSCG00000006923 | GBP2 | -2.895067912 | 2.12E-51 | Down |
| ENSSSCG00000035153 | TRIM38 | -1.762619221 | 2.31E-51 | Down |
| ENSSSCG00000005364 | TDRD7 | -1.221928704 | 2.68E-51 | Down |
| ENSSSCG00000004670 | C15orf48 | -1.286849138 | 1.11E-50 | Down |
| ENSSSCG00000001667 | ZNF318 | -1.095340023 | 1.59E-50 | Down |
| ENSSSCG00000039751 | NLRC5 | -3.875781418 | 9.08E-50 | Down |
| ENSSSCG00000011874 | PARP14 | -3.320897958 | 1.31E-49 | Down |
| ENSSSCG00000014780 | TRIM21 | -1.256509821 | 1.62E-49 | Down |
| ENSSSCG00000015801 | TLR3 | -1.481515657 | 2.03E-49 | Down |
| ENSSSCG00000031356 | HES1 | -1.295083598 | 7.33E-49 | Down |
| ENSSSCG00000040061 | NINJ1 | -1.068174065 | 7.60E-49 | Down |
| ENSSSCG00000035940 | SPSB1 | -1.895688258 | 1.12E-48 | Down |
| ENSSSCG00000012853 | IRF7 | -2.364538523 | 4.02E-48 | Down |
| ENSSSCG00000016262 | NA | -1.117604042 | 5.22E-48 | Down |
| ENSSSCG00000031610 | NA | -4.346551299 | 5.85E-48 | Down |
| ENSSSCG00000014277 | IRF1 | -1.831254469 | 5.97E-48 | Down |
| ENSSSCG00000032715 | CERS6 | -1.3717314 | 1.27E-47 | Down |
| ENSSSCG00000015525 | TOR3A | -1.064713636 | 4.47E-47 | Down |
| ENSSSCG00000024914 | NA | -1.988717652 | 6.11E-47 | Down |
| ENSSSCG00000017488 | CSF3 | -2.307711516 | 8.08E-47 | Down |
| ENSSSCG00000033089 | NA | -4.134867252 | 1.47E-46 | Down |
| ENSSSCG00000003137 | PLEKHA4 | -2.009643218 | 6.46E-46 | Down |
| ENSSSCG00000017298 | TANC2 | -1.389606118 | 8.12E-46 | Down |
| ENSSSCG00000017614 | TRIM25 | -1.592529514 | 1.12E-45 | Down |
| ENSSSCG00000023379 | UBE2L6 | -2.030148162 | 1.17E-45 | Down |
| ENSSSCG00000036136 | BHLHE40 | -1.105884367 | 1.66E-45 | Down |
| ENSSSCG00000006625 | RFX5 | -1.401133525 | 1.81E-45 | Down |
| ENSSSCG00000026169 | MFSD12 | -1.167927914 | 2.08E-45 | Down |
| ENSSSCG00000015563 | RGL1 | -1.090928505 | 2.30E-45 | Down |
| ENSSSCG00000025856 | TMEM106A | -1.532966765 | 3.69E-45 | Down |
| ENSSSCG00000008648 | RSAD2 | -4.905152767 | 5.50E-45 | Down |
| ENSSSCG00000034980 | IRF8 | -3.495870956 | 8.09E-45 | Down |
| ENSSSCG00000033222 | TRIM14 | -1.649395674 | 2.88E-43 | Down |
| ENSSSCG00000012258 | FUNDC1 | -1.038744984 | 8.86E-43 | Down |
| ENSSSCG00000009125 | ANK2 | -1.011394418 | 1.46E-42 | Down |
| ENSSSCG00000011495 | PRICKLE2 | -2.678317043 | 1.89E-42 | Down |
| ENSSSCG00000030548 | HERC5 | -2.965650206 | 1.91E-42 | Down |
| ENSSSCG00000005224 | GLIS3 | -1.006749358 | 2.00E-42 | Down |
| ENSSSCG00000030801 | NA | -3.541529704 | 2.95E-42 | Down |
| ENSSSCG00000023716 | TNFAIP6 | -1.098329181 | 2.96E-42 | Down |
| ENSSSCG00000030108 | ZNFX1 | -1.280168394 | 3.38E-42 | Down |
| ENSSSCG00000027894 | FAM76A | -1.274044085 | 3.82E-42 | Down |
| ENSSSCG00000032436 | NA | -1.672844913 | 6.88E-42 | Down |
| ENSSSCG00000016057 | STAT1 | -1.443013555 | 9.65E-42 | Down |
| ENSSSCG00000017705 | CCL5 | -1.238594426 | 2.49E-41 | Down |
| ENSSSCG00000033703 | FAM111A | -1.003475871 | 5.64E-41 | Down |
| ENSSSCG00000014670 | TRIM5 | -1.186464092 | 9.39E-41 | Down |
| ENSSSCG00000037241 | RGS2 | -1.644517431 | 1.39E-40 | Down |
| ENSSSCG00000006919 | NA | -3.950152212 | 2.92E-40 | Down |
| ENSSSCG00000015784 | ACSL1 | -1.283939429 | 3.71E-40 | Down |
| ENSSSCG00000017146 | RNF213 | -2.012932449 | 7.67E-40 | Down |
| ENSSSCG00000035037 | NA | -1.771143396 | 1.45E-39 | Down |
| ENSSSCG00000025788 | ENPP4 | -2.229726743 | 1.62E-39 | Down |
| ENSSSCG00000011239 | NA | -3.314094642 | 5.76E-39 | Down |
| ENSSSCG00000000728 | PARP11 | -1.517311598 | 5.89E-39 | Down |
| ENSSSCG00000012076 | MX2 | -3.787010728 | 1.14E-38 | Down |
| ENSSSCG00000036956 | NA | -1.178719982 | 1.63E-37 | Down |
| ENSSSCG00000000148 | NA | -2.723677288 | 2.45E-37 | Down |
| ENSSSCG00000027646 | TIPARP | -1.020550161 | 4.27E-37 | Down |
| ENSSSCG00000040617 | TNFAIP8 | -1.739604337 | 1.14E-36 | Down |
| ENSSSCG00000004830 | ATP10A | -1.06373646 | 3.64E-36 | Down |
| ENSSSCG00000033613 | FOXS1 | -2.343926724 | 9.68E-36 | Down |
| ENSSSCG00000012077 | MX1 | -2.141866901 | 3.67E-35 | Down |
| ENSSSCG00000013147 | FAM111B | -1.075728569 | 3.83E-35 | Down |
| ENSSSCG00000027709 | PARP9 | -1.912207667 | 6.21E-35 | Down |
| ENSSSCG00000010452 | IFIT1 | -2.825628905 | 1.13E-34 | Down |
| ENSSSCG00000035420 | HES4 | -3.491621958 | 1.70E-34 | Down |
| ENSSSCG00000001347 | PPP1R10 | -1.020502204 | 1.89E-34 | Down |
| ENSSSCG00000008334 | MXD1 | -2.031560322 | 2.24E-34 | Down |
| ENSSSCG00000038132 | IRF2BP2 | -1.013289457 | 2.59E-34 | Down |
| ENSSSCG00000023178 | BATF2 | -2.976116644 | 7.48E-34 | Down |
| ENSSSCG00000032474 | CXCL10 | -3.87813204 | 9.05E-34 | Down |
| ENSSSCG00000010454 | IFIT5 | -1.601418803 | 1.26E-33 | Down |
| ENSSSCG00000017755 | NOS2 | -1.104890225 | 1.30E-33 | Down |
| ENSSSCG00000014565 | NA | -1.034401317 | 5.52E-33 | Down |
| ENSSSCG00000001912 | PML | -1.425948482 | 7.72E-33 | Down |
| ENSSSCG00000016986 | CREBRF | -1.453536348 | 7.75E-33 | Down |
| ENSSSCG00000009240 | PLAC8 | -2.806169092 | 1.37E-32 | Down |
| ENSSSCG00000013307 | LMO2 | -2.41053102 | 1.39E-32 | Down |
| ENSSSCG00000008787 | KLHL5 | -1.102446423 | 3.42E-32 | Down |
| ENSSSCG00000030408 | DDX58 | -2.562999471 | 3.47E-32 | Down |
| ENSSSCG00000007508 | ZBP1 | -2.768166746 | 4.30E-32 | Down |
| ENSSSCG00000032652 | NA | -2.076065864 | 2.65E-31 | Down |
| ENSSSCG00000008496 | EIF2AK2 | -1.42579077 | 3.45E-31 | Down |
| ENSSSCG00000012828 | STARD8 | -1.21281312 | 4.44E-31 | Down |
| ENSSSCG00000017416 | DHX58 | -2.031429725 | 2.41E-30 | Down |
| ENSSSCG00000010451 | IFIT2 | -2.818736527 | 3.54E-30 | Down |
| ENSSSCG00000027480 | KLF10 | -1.042446325 | 3.79E-30 | Down |
| ENSSSCG00000025286 | MCTP1 | -1.38259117 | 8.35E-30 | Down |
| ENSSSCG00000000774 | USP18 | -2.398785484 | 9.28E-30 | Down |
| ENSSSCG00000013382 | PLEKHA7 | -3.103087449 | 1.76E-29 | Down |
| ENSSSCG00000006987 | SLC7A2 | -1.869741568 | 1.82E-29 | Down |
| ENSSSCG00000011264 | CSRNP1 | -1.321604636 | 2.09E-29 | Down |
| ENSSSCG00000015897 | IFIH1 | -1.640137445 | 2.69E-29 | Down |
| ENSSSCG00000020906 | TNFSF10 | -3.548984907 | 6.99E-29 | Down |
| ENSSSCG00000034351 | B3GALT1 | -1.307522125 | 6.99E-29 | Down |
| ENSSSCG00000012055 | MORC3 | -1.018958692 | 7.93E-29 | Down |
| ENSSSCG00000009542 | TNFSF13B | -3.977310949 | 1.06E-28 | Down |
| ENSSSCG00000022312 | RHPN2 | -2.568413719 | 5.59E-28 | Down |
| ENSSSCG00000034973 | CXCL12 | -1.225214534 | 1.33E-27 | Down |
| ENSSSCG00000009881 | OAS2 | -2.212725312 | 1.36E-27 | Down |
| ENSSSCG00000015299 | STEAP4 | -3.072377817 | 2.12E-27 | Down |
| ENSSSCG00000015324 | GNG11 | -1.109734272 | 3.25E-27 | Down |
| ENSSSCG00000021712 | HERC6 | -2.066246727 | 3.34E-27 | Down |
| ENSSSCG00000001561 | ETV7 | -2.923840503 | 4.36E-27 | Down |
| ENSSSCG00000025836 | SULT1C4 | -2.634476453 | 8.13E-27 | Down |
| ENSSSCG00000005211 | CD274 | -3.377926492 | 1.72E-26 | Down |
| ENSSSCG00000034802 | NA | -1.426542594 | 3.61E-26 | Down |
| ENSSSCG00000010638 | TCF7L2 | -1.068169448 | 7.87E-26 | Down |
| ENSSSCG00000020705 | MAP3K8 | -1.052688747 | 1.10E-25 | Down |
| ENSSSCG00000027860 | ERAP2 | -1.131581391 | 1.12E-25 | Down |
| ENSSSCG00000009720 | DDX60 | -2.467610334 | 1.45E-25 | Down |
| ENSSSCG00000036340 | ZBTB5 | -1.991670934 | 1.32E-24 | Down |
| ENSSSCG00000014618 | NA | -3.978286341 | 1.32E-24 | Down |
| ENSSSCG00000040575 | ISG15 | -2.414553827 | 1.46E-24 | Down |
| ENSSSCG00000023630 | CPM | -1.223840752 | 4.03E-24 | Down |
| ENSSSCG00000036383 | LGALS3BP | -1.745588942 | 1.32E-23 | Down |
| ENSSSCG00000011876 | DTX3L | -2.432374853 | 1.80E-23 | Down |
| ENSSSCG00000027855 | SOCS1 | -2.262165487 | 2.11E-23 | Down |
| ENSSSCG00000011106 | CREM | -1.002218604 | 3.78E-23 | Down |
| ENSSSCG00000017754 | NA | -3.078459675 | 1.52E-22 | Down |
| ENSSSCG00000040673 | TMEM140 | -2.74754179 | 1.44E-21 | Down |
| ENSSSCG00000033453 | BST2 | -1.99497692 | 1.62E-21 | Down |
| ENSSSCG00000038867 | PPM1K | -1.224286277 | 1.62E-21 | Down |
| ENSSSCG00000035240 | GPR63 | -3.523455846 | 1.74E-21 | Down |
| ENSSSCG00000037735 | NA | -1.710816118 | 2.38E-20 | Down |
| ENSSSCG00000027660 | IFI44L | -1.366209889 | 3.35E-19 | Down |
| ENSSSCG00000008647 | CMPK2 | -2.312490509 | 2.86E-18 | Down |
| ENSSSCG00000039568 | SNAI2 | -1.265767671 | 3.41E-18 | Down |
| ENSSSCG00000016519 | AKR1D1 | -1.211657405 | 5.12E-18 | Down |
| ENSSSCG00000011353 | PFKFB4 | -1.021894055 | 4.23E-17 | Down |
| ENSSSCG00000025560 | PGLYRP2 | -1.55387062 | 4.30E-17 | Down |
| ENSSSCG00000038141 | TICAM2 | -1.677324989 | 4.91E-17 | Down |
| ENSSSCG00000012695 | INTS6L | -1.19105431 | 6.30E-17 | Down |
| ENSSSCG00000017886 | FBXO39 | -1.786527705 | 7.28E-17 | Down |
| ENSSSCG00000032475 | CEP44 | -1.055027748 | 1.18E-16 | Down |
| ENSSSCG00000003763 | IFI44 | -1.339027949 | 2.16E-16 | Down |
| ENSSSCG00000016018 | FRZB | -1.992138577 | 2.65E-16 | Down |
| ENSSSCG00000037016 | ID1 | -1.037307478 | 1.87E-15 | Down |
| ENSSSCG00000013664 | C19orf66 | -1.190553761 | 2.93E-15 | Down |
| ENSSSCG00000014672 | NA | -1.886685517 | 6.72E-15 | Down |
| ENSSSCG00000004902 | RNF152 | -1.206887106 | 1.26E-14 | Down |
| ENSSSCG00000035216 | SPRED3 | -1.032415895 | 1.81E-14 | Down |
| ENSSSCG00000005166 | MLLT3 | -1.501160616 | 2.60E-14 | Down |
| ENSSSCG00000032710 | E2F2 | -1.543187889 | 2.63E-14 | Down |
| ENSSSCG00000029507 | RASGEF1B | -1.624890545 | 6.40E-14 | Down |
| ENSSSCG00000037468 | GNE | -1.03832623 | 1.16E-13 | Down |
| ENSSSCG00000031924 | NKX3-1 | -2.026945429 | 3.38E-13 | Down |
| ENSSSCG00000033787 | NA | -2.696285329 | 3.75E-13 | Down |
| ENSSSCG00000014336 | EGR1 | -1.318690722 | 6.64E-13 | Down |
| ENSSSCG00000031321 | NR4A1 | -1.426429141 | 1.10E-12 | Down |
| ENSSSCG00000002252 | ARRDC4 | -1.008446393 | 1.32E-12 | Down |
| ENSSSCG00000004369 | PRDM1 | -1.175324843 | 3.09E-12 | Down |
| ENSSSCG00000006247 | PLAG1 | -1.349107226 | 1.07E-11 | Down |
| ENSSSCG00000007659 | ZCWPW1 | -1.410809076 | 1.91E-11 | Down |
| ENSSSCG00000016959 | MARVELD2 | -1.208372092 | 2.33E-11 | Down |
| ENSSSCG00000010224 | EGR2 | -1.38968589 | 2.80E-11 | Down |
| ENSSSCG00000001064 | GMPR | -1.533388468 | 1.27E-10 | Down |
| ENSSSCG00000028711 | CASP1 | -3.262705815 | 3.23E-10 | Down |
| ENSSSCG00000017607 | TMEM100 | -2.037043989 | 7.14E-10 | Down |
| ENSSSCG00000034570 | IFI6 | -1.264375935 | 9.36E-10 | Down |
| ENSSSCG00000034989 | LRRTM2 | -1.369657951 | 9.70E-10 | Down |
| ENSSSCG00000032422 | NA | -1.355478958 | 1.15E-09 | Down |
| ENSSSCG00000001787 | IL16 | -1.042451828 | 1.21E-09 | Down |
| ENSSSCG00000001252 | NA | -3.795603049 | 1.86E-09 | Down |
| ENSSSCG00000026729 | TMEM150C | -2.09718202 | 2.69E-09 | Down |
| ENSSSCG00000024161 | NA | -1.348821795 | 3.21E-09 | Down |
| ENSSSCG00000026943 | MRAP2 | -2.600947521 | 6.16E-09 | Down |
| ENSSSCG00000035297 | ISG12(A) | -1.601227101 | 7.63E-09 | Down |
| ENSSSCG00000012375 | DLG3 | -1.646652308 | 7.63E-09 | Down |
| ENSSSCG00000000623 | BCL2L14 | -4.02672771 | 2.54E-08 | Down |
| ENSSSCG00000036064 | CALHM6 | -1.764732506 | 3.14E-08 | Down |
| ENSSSCG00000018015 | DNAH9 | -2.140967823 | 3.24E-08 | Down |
| ENSSSCG00000009921 | OASL | -4.216613227 | 3.96E-08 | Down |
| ENSSSCG00000038149 | KCNE4 | -1.057328553 | 4.63E-08 | Down |
| ENSSSCG00000026592 | TLR6 | -1.727184729 | 4.94E-08 | Down |
| ENSSSCG00000000773 | TUBA8 | -1.483094859 | 7.31E-08 | Down |
| ENSSSCG00000036693 | NA | -1.307146308 | 9.66E-08 | Down |
| ENSSSCG00000032149 | PLET1 | -1.363082662 | 2.63E-07 | Down |
| ENSSSCG00000004497 | PSTPIP2 | -1.569300781 | 3.33E-07 | Down |
| ENSSSCG00000008978 | CXCL11 | -3.640263636 | 3.74E-07 | Down |
| ENSSSCG00000001231 | NA | -1.350020591 | 3.79E-07 | Down |
| ENSSSCG00000015522 | ANGPTL1 | -1.154948744 | 3.79E-07 | Down |
| ENSSSCG00000009410 | RUBCNL | -2.202575378 | 4.02E-07 | Down |
| ENSSSCG00000013311 | KIAA1549L | -1.121871996 | 4.95E-07 | Down |
| ENSSSCG00000004218 | RSPO3 | -1.22934132 | 5.80E-07 | Down |
| ENSSSCG00000009132 | ENPEP | -2.347261346 | 6.33E-07 | Down |
| ENSSSCG00000022849 | IL2RA | -1.086634287 | 2.51E-06 | Down |
| ENSSSCG00000034741 | HOXD11 | -1.234013687 | 4.78E-06 | Down |
| ENSSSCG00000035598 | EDN1 | -1.395983159 | 6.12E-06 | Down |
| ENSSSCG00000011391 | CDHR4 | -2.027200684 | 7.81E-06 | Down |
| ENSSSCG00000015979 | HOXD13 | -1.63423593 | 9.30E-06 | Down |
| ENSSSCG00000037598 | SNX10 | -1.169273517 | 1.18E-05 | Down |
| ENSSSCG00000022256 | C10orf10 | -1.201882495 | 1.19E-05 | Down |
| ENSSSCG00000012132 | ASB9 | -1.867032773 | 1.58E-05 | Down |
| ENSSSCG00000032221 | FAM110C | -1.020383505 | 1.77E-05 | Down |
| ENSSSCG00000022361 | NA | -1.161477248 | 2.43E-05 | Down |
| ENSSSCG00000005967 | FAM84B | -1.836941349 | 4.45E-05 | Down |
| ENSSSCG00000039300 | IL27 | -3.473381176 | 8.91E-05 | Down |
| ENSSSCG00000032686 | RUNX3 | -1.93586459 | 9.79E-05 | Down |
| ENSSSCG00000001042 | MAK | -1.17492528 | 0.000111946 | Down |
| ENSSSCG00000024867 | ISG20 | -4.862704815 | 0.000113984 | Down |
| ENSSSCG00000032561 | PDCD1LG2 | -4.083904267 | 0.000132032 | Down |
| ENSSSCG00000036742 | KLF15 | -2.323654037 | 0.000133391 | Down |
| ENSSSCG00000015476 | CHI3L1 | -1.140238984 | 0.000134652 | Down |
| ENSSSCG00000013890 | SLC5A5 | -1.898875862 | 0.000174734 | Down |
| ENSSSCG00000007901 | CIITA | -5.445298692 | 0.000193049 | Down |
| ENSSSCG00000005268 | RORB | -2.032995298 | 0.000215572 | Down |
| ENSSSCG00000016164 | IKZF2 | -1.463077616 | 0.000306397 | Down |
| ENSSSCG00000008124 | NEURL3 | -3.602530259 | 0.000402926 | Down |
| ENSSSCG00000006802 | NA | -1.006420298 | 0.000411957 | Down |
| ENSSSCG00000036932 | WNT6 | -1.31311033 | 0.000440745 | Down |
| ENSSSCG00000039758 | NA | -1.547669674 | 0.000564124 | Down |
| ENSSSCG00000005620 | SH2D3C | -1.286735623 | 0.000686056 | Down |
| ENSSSCG00000006418 | NA | -5.040851044 | 0.000862814 | Down |
| ENSSSCG00000023785 | TMEM156 | -4.986001388 | 0.001124634 | Down |
| ENSSSCG00000010504 | BLNK | -2.269008151 | 0.001190206 | Down |
| ENSSSCG00000009859 | NA | -3.124481317 | 0.001388346 | Down |
| ENSSSCG00000040017 | NKX2-2 | -1.103403136 | 0.001681872 | Down |
| ENSSSCG00000035732 | NA | -4.396014572 | 0.001711228 | Down |
| ENSSSCG00000034708 | PITX2 | -1.984636336 | 0.002131557 | Down |
| ENSSSCG00000034691 | ZEB2_AS1_1 | -1.085433875 | 0.002432077 | Down |
| ENSSSCG00000006542 | KCNN3 | -2.159446936 | 0.002454229 | Down |
| ENSSSCG00000026583 | TLR1 | -2.397498532 | 0.00275277 | Down |
| ENSSSCG00000006932 | NA | -5.251993886 | 0.003116444 | Down |
| ENSSSCG00000005994 | SNTB1 | -1.097503575 | 0.004045504 | Down |
| ENSSSCG00000015953 | DLX1 | -1.494041062 | 0.005341788 | Down |
| ENSSSCG00000011730 | IL12A | -1.881099648 | 0.00862554 | Down |
| ENSSSCG00000011630 | ACKR4 | -2.724480192 | 0.011814662 | Down |
| ENSSSCG00000022490 | GPR83 | -2.192466141 | 0.015257906 | Down |
| ENSSSCG00000006161 | IL7 | -1.195103857 | 0.01565801 | Down |
| ENSSSCG00000036865 | NA | -1.55605158 | 0.018077132 | Down |
| ENSSSCG00000033520 | IL23A | -1.092980502 | 0.021038418 | Down |
| ENSSSCG00000009051 | IL15 | -1.164077786 | 0.023422857 | Down |
| ENSSSCG00000000718 | GALNT8 | -2.249865464 | 0.024726144 | Down |
| ENSSSCG00000007499 | TFAP2C | -1.740553923 | 0.031961313 | Down |
| ENSSSCG00000006243 | PENK | -1.472359244 | 0.032174524 | Down |
| ENSSSCG00000039214 | NA | -1.167817175 | 0.032662693 | Down |
| ENSSSCG00000040412 | NA | -1.189321466 | 0.034254274 | Down |
| ENSSSCG00000029284 | NPHS1 | -1.590288286 | 0.03939 | Down |
| ENSSSCG00000016832 | IL7R | -2.630783765 | 0.045955746 | Down |
| ENSSSCG00000000573 | KCNJ8 | -2.158408052 | 0.046642867 | Down |
| ENSSSCG00000035907 | NA | -1.652440218 | 0.049094531 | Down |

Gene Name “NA” indicates the gene ID was not matched to a HGNC gene name.
