# Supplementary material for: A Tale of Two Loads: Modulation of IL-1 Induced Inflammatory Responses of Meniscal Cells in Two Models of Dynamic Physiologic Loading
Source: Front Bioeng Biotechnol. 2022 Mar 1;10:837619. doi: 10.3389/fbioe.2022.837619 (PMC8921261; doi:10.3389/fbioe.2022.837619)
Supplement: Supplementary file 5 [file Table1.DOCX]

**Supplemental Table 1**: Forward and reverse primer pairs for targets assessed using RT-qPCR on cell stretch samples.

| **Target Name** | **Forward** | **Reverse** |
| --- | --- | --- |
| *CASP7* | CGTTGCGGCTTTACTTTCG | GCTGGACGACACAGTCTTGA |
| *CXCL10* | TGCAGCACCATGAACCAAAG | TCTGGAGAGAGGTATTCCTTGAG |
| *IRF1* | AGCGACCTGTACAACTTCCA | CCTTCCCATCCACGTTTGTC |
| *NFATC2* | CAGAGAGAGGCTGCGTTCA | GGCGACCTTATGTGCATTCG |
| *NOS2* | AGCACAAGCTGAAGAAATCCG | TCACACTCGCCATCTCCATC |
| *RRAD* | GACGTGCCCATCATCCTAGT | CCCGGCCCTCATCCAA |
| *STAT1* | CCTCGATCAGCTGCAGAACT | GTCCCACAACGCTTGTTTGT |
| *STAT2* | GCATAGAGCGGGGTCTGA | GGTGCAGCTGATCCTGAAAT |
