# Supplementary material for: A Tale of Two Loads: Modulation of IL-1 Induced Inflammatory Responses of Meniscal Cells in Two Models of Dynamic Physiologic Loading
Source: Front Bioeng Biotechnol. 2022 Mar 1;10:837619. doi: 10.3389/fbioe.2022.837619 (PMC8921261; doi:10.3389/fbioe.2022.837619)
Supplement: Supplementary file 6 [file DataSheet3.DOCX]

**Supplemental Table 4**: IL-1α stimulation compared to unstimulated samples for unloaded outer zone cells.

| **Gene ID** | **Gene Name** | **Log2Fold Change** | **p-value** | **Up/Down Regulated** |
| --- | --- | --- | --- | --- |
| ENSSSCG00000008953 | CXCL8 | 8.274829784 | 0 | UP |
| ENSSSCG00000038562 | RND1 | 7.292939543 | 0 | UP |
| ENSSSCG00000008959 | CXCL2 | 6.345825596 | 0 | UP |
| ENSSSCG00000004572 | NA | 5.968234757 | 0 | UP |
| ENSSSCG00000010212 | NA | 5.623297919 | 0 | UP |
| ENSSSCG00000017705 | CCL5 | 5.448605481 | 0 | UP |
| ENSSSCG00000040725 | IL11 | 5.345510893 | 0 | UP |
| ENSSSCG00000040961 | LIF | 4.972142722 | 0 | UP |
| ENSSSCG00000011727 | PTX3 | 4.931633301 | 0 | UP |
| ENSSSCG00000006359 | ADAMTS4 | 4.86815473 | 0 | UP |
| ENSSSCG00000004125 | STX11 | 4.814005766 | 0 | UP |
| ENSSSCG00000023716 | TNFAIP6 | 4.752432922 | 0 | UP |
| ENSSSCG00000023522 | TGM2 | 4.715388721 | 0 | UP |
| ENSSSCG00000008957 | AMCF-II | 4.675163574 | 0 | UP |
| ENSSSCG00000004154 | TNFAIP3 | 4.656256851 | 0 | UP |
| ENSSSCG00000040317 | SOD2 | 4.638341266 | 0 | UP |
| ENSSSCG00000031970 | RASSF5 | 4.624521427 | 0 | UP |
| ENSSSCG00000015579 | PTGS2 | 4.400232318 | 0 | UP |
| ENSSSCG00000015784 | ACSL1 | 4.381498014 | 0 | UP |
| ENSSSCG00000005688 | PTGES | 3.941070871 | 0 | UP |
| ENSSSCG00000030300 | MT2A | 3.902740616 | 0 | UP |
| ENSSSCG00000031579 | NA | 3.872852521 | 0 | UP |
| ENSSSCG00000014924 | CTSC | 3.829495399 | 0 | UP |
| ENSSSCG00000014249 | MARCH3 | 3.705210857 | 0 | UP |
| ENSSSCG00000024166 | SLC2A6 | 3.704291467 | 0 | UP |
| ENSSSCG00000009125 | ANK2 | 3.644648789 | 0 | UP |
| ENSSSCG00000010330 | PPIF | 3.626327777 | 0 | UP |
| ENSSSCG00000031380 | NA | 3.565744245 | 0 | UP |
| ENSSSCG00000017723 | CCL2 | 3.522819767 | 0 | UP |
| ENSSSCG00000003669 | MFSD2A | 3.516488663 | 0 | UP |
| ENSSSCG00000002501 | NA | 3.472451571 | 0 | UP |
| ENSSSCG00000003451 | NA | 3.409206319 | 0 | UP |
| ENSSSCG00000009655 | EBF2 | 3.357000663 | 0 | UP |
| ENSSSCG00000013940 | NLRP3 | 3.352961044 | 0 | UP |
| ENSSSCG00000009434 | RGCC | 3.319518933 | 0 | UP |
| ENSSSCG00000030655 | MAMDC2 | 3.245047106 | 0 | UP |
| ENSSSCG00000007465 | B4GALT5 | 3.232234846 | 0 | UP |
| ENSSSCG00000032097 | DUSP16 | 3.17019883 | 0 | UP |
| ENSSSCG00000001952 | NFKBIA | 3.167209005 | 0 | UP |
| ENSSSCG00000039731 | NA | 3.154326825 | 0 | UP |
| ENSSSCG00000038594 | SDC4 | 3.139706308 | 0 | UP |
| ENSSSCG00000011218 | SLC4A7 | 3.034301319 | 0 | UP |
| ENSSSCG00000040608 | AKR1B1 | 3.002954567 | 0 | UP |
| ENSSSCG00000017920 | NA | 2.98491373 | 0 | UP |
| ENSSSCG00000031109 | HILPDA | 2.980664556 | 0 | UP |
| ENSSSCG00000037815 | ZC3H12A | 2.929500407 | 0 | UP |
| ENSSSCG00000027607 | IER3 | 2.924531847 | 0 | UP |
| ENSSSCG00000017091 | TNIP1 | 2.901320337 | 0 | UP |
| ENSSSCG00000032622 | PPP3CC | 2.863737981 | 0 | UP |
| ENSSSCG00000003805 | PDE4B | 2.805843383 | 0 | UP |
| ENSSSCG00000010580 | NFKB2 | 2.700623415 | 0 | UP |
| ENSSSCG00000004919 | NEDD4L | 2.653317486 | 0 | UP |
| ENSSSCG00000007874 | NA | 2.609903764 | 0 | UP |
| ENSSSCG00000016438 | NUB1 | 2.580516773 | 0 | UP |
| ENSSSCG00000017402 | STAT5A | 2.574503856 | 0 | UP |
| ENSSSCG00000030957 | NFKB1 | 2.542927534 | 0 | UP |
| ENSSSCG00000027477 | TBC1D2B | 2.533969686 | 0 | UP |
| ENSSSCG00000004050 | WTAP | 2.511559141 | 0 | UP |
| ENSSSCG00000001073 | TPMT | 2.367621758 | 0 | UP |
| ENSSSCG00000015650 | MAPKAPK2 | 2.339478983 | 0 | UP |
| ENSSSCG00000022402 | GRAMD2B | 2.336106528 | 0 | UP |
| ENSSSCG00000013655 | ICAM1 | 2.284938413 | 0 | UP |
| ENSSSCG00000023351 | PLA2G4A | 2.271696063 | 0 | UP |
| ENSSSCG00000016101 | CFLAR | 2.266985176 | 0 | UP |
| ENSSSCG00000025770 | ST6GAL1 | 2.242473077 | 0 | UP |
| ENSSSCG00000012583 | ACSL4 | 2.157441573 | 0 | UP |
| ENSSSCG00000013298 | PDHX | 2.106711384 | 0 | UP |
| ENSSSCG00000006502 | ARHGEF2 | 2.103836349 | 0 | UP |
| ENSSSCG00000002716 | MLKL | 2.082376995 | 0 | UP |
| ENSSSCG00000034378 | IFNGR2 | 2.07916226 | 0 | UP |
| ENSSSCG00000017835 | CLUH | 2.058759549 | 0 | UP |
| ENSSSCG00000033120 | PALM2 | 2.032044842 | 0 | UP |
| ENSSSCG00000008881 | RAPGEF2 | 1.979673937 | 0 | UP |
| ENSSSCG00000028536 | LHFPL2 | 1.944715529 | 0 | UP |
| ENSSSCG00000000843 | TXNRD1 | 1.863874007 | 0 | UP |
| ENSSSCG00000026466 | SLC23A2 | 1.829882669 | 0 | UP |
| ENSSSCG00000010540 | ENTPD7 | 1.80997547 | 0 | UP |
| ENSSSCG00000040181 | ELL | 1.796051827 | 0 | UP |
| ENSSSCG00000028679 | NA | 1.779631693 | 0 | UP |
| ENSSSCG00000037832 | PMP22 | 1.762926733 | 0 | UP |
| ENSSSCG00000013297 | CD44 | 1.735413113 | 0 | UP |
| ENSSSCG00000039314 | MCL1 | 1.72458547 | 0 | UP |
| ENSSSCG00000005096 | HIF1A | 1.493147651 | 0 | UP |
| ENSSSCG00000023408 | SAMD4A | 1.366851422 | 0 | UP |
| ENSSSCG00000010261 | PPA1 | 2.392549117 | 5.1E-305 | UP |
| ENSSSCG00000013366 | LDHA | 1.294849166 | 1.2E-304 | UP |
| ENSSSCG00000032417 | NGF | 3.971724139 | 3.3E-303 | UP |
| ENSSSCG00000028182 | CDK17 | 2.315542939 | 8.2E-301 | UP |
| ENSSSCG00000026041 | MAP3K5 | 2.861168483 | 9.6E-301 | UP |
| ENSSSCG00000004509 | LIPG | 2.49404951 | 4.1E-298 | UP |
| ENSSSCG00000010302 | USP54 | 2.200456968 | 5.2E-297 | UP |
| ENSSSCG00000015235 | ETS1 | 1.511992488 | 2.3E-288 | UP |
| ENSSSCG00000035650 | NA | 1.698664996 | 5.3E-286 | UP |
| ENSSSCG00000017672 | MED13 | 1.952864557 | 5.8E-284 | UP |
| ENSSSCG00000035078 | CD40 | 2.834266397 | 1.2E-280 | UP |
| ENSSSCG00000033786 | NA | 1.894646927 | 3.3E-273 | UP |
| ENSSSCG00000033015 | B3GNT7 | 3.559532705 | 1.4E-269 | UP |
| ENSSSCG00000007355 | NA | 1.536041711 | 4.4E-268 | UP |
| ENSSSCG00000007140 | SMOX | 2.012398525 | 8.9E-268 | UP |
| ENSSSCG00000011825 | ATP13A3 | 2.325704829 | 2E-267 | UP |
| ENSSSCG00000026454 | NA | 2.912195086 | 6.2E-267 | UP |
| ENSSSCG00000001516 | BAK1 | 2.045775334 | 1.6E-266 | UP |
| ENSSSCG00000035859 | WNT5A | 3.782639115 | 9.4E-265 | UP |
| ENSSSCG00000015435 | NAMPT | 2.579798441 | 3E-261 | UP |
| ENSSSCG00000028063 | TACC2 | 2.369634809 | 1.4E-258 | UP |
| ENSSSCG00000005097 | SNAPC1 | 3.177981067 | 1.7E-256 | UP |
| ENSSSCG00000020970 | IL6 | 7.286107709 | 2E-256 | UP |
| ENSSSCG00000024759 | CX3CL1 | 3.379719205 | 1.9E-254 | UP |
| ENSSSCG00000025593 | NA | 1.939381181 | 2E-252 | UP |
| ENSSSCG00000035037 | NA | 4.679126501 | 9.9E-252 | UP |
| ENSSSCG00000027340 | NA | 2.156553356 | 6E-249 | UP |
| ENSSSCG00000010241 | TET1 | 2.812497567 | 2.4E-245 | UP |
| ENSSSCG00000006987 | SLC7A2 | 7.437742752 | 5.5E-245 | UP |
| ENSSSCG00000011570 | IRAK2 | 4.61580024 | 5.9E-245 | UP |
| ENSSSCG00000039862 | TRIB3 | 3.496695901 | 3.1E-238 | UP |
| ENSSSCG00000002135 | PNP | 2.210009433 | 2E-236 | UP |
| ENSSSCG00000011437 | ALAS1 | 1.619531418 | 3.4E-234 | UP |
| ENSSSCG00000030165 | MAFF | 2.334094701 | 2.9E-233 | UP |
| ENSSSCG00000039953 | PNRC1 | 2.279751917 | 3.4E-233 | UP |
| ENSSSCG00000036746 | RASL10B | 2.304439972 | 2.1E-232 | UP |
| ENSSSCG00000025598 | COBLL1 | 2.44575882 | 3.9E-232 | UP |
| ENSSSCG00000022649 | SLC7A11 | 2.511715392 | 3.4E-231 | UP |
| ENSSSCG00000036893 | PTHLH | 3.31206408 | 1.9E-229 | UP |
| ENSSSCG00000004825 | CHSY1 | 1.663048848 | 5.5E-227 | UP |
| ENSSSCG00000033750 | NA | 3.927026576 | 4.5E-226 | UP |
| ENSSSCG00000038879 | RELB | 1.754469439 | 6.2E-221 | UP |
| ENSSSCG00000004421 | FYN | 1.607051479 | 1.8E-219 | UP |
| ENSSSCG00000038185 | EREG | 4.140562355 | 5.9E-219 | UP |
| ENSSSCG00000003707 | NPC1 | 1.888354476 | 3.1E-218 | UP |
| ENSSSCG00000015987 | NFE2L2 | 1.453790028 | 2.5E-216 | UP |
| ENSSSCG00000034207 | CEBPB | 1.871223585 | 4.6E-216 | UP |
| ENSSSCG00000009042 | OTUD4 | 1.931818819 | 3.7E-215 | UP |
| ENSSSCG00000013758 | ZSWIM4 | 2.080322136 | 1.7E-214 | UP |
| ENSSSCG00000006862 | VCAM1 | 4.056921159 | 9.8E-214 | UP |
| ENSSSCG00000014012 | GFPT2 | 1.719497697 | 3.8E-213 | UP |
| ENSSSCG00000015770 | VEGFC | 1.803092007 | 1.9E-212 | UP |
| ENSSSCG00000038500 | TRIB1 | 2.34299825 | 2.3E-212 | UP |
| ENSSSCG00000016922 | GPBP1 | 1.814957698 | 5.3E-211 | UP |
| ENSSSCG00000027030 | BDKRB2 | 3.108552278 | 4.2E-210 | UP |
| ENSSSCG00000011056 | FRMD4A | 1.304855224 | 3.2E-208 | UP |
| ENSSSCG00000012027 | ADAMTS5 | 3.563313358 | 3.3E-208 | UP |
| ENSSSCG00000035790 | BTG1 | 1.378238126 | 3.8E-208 | UP |
| ENSSSCG00000034386 | ATP2A2 | 1.019224699 | 7.6E-207 | UP |
| ENSSSCG00000009002 | TLR2 | 4.485918925 | 8.9E-207 | UP |
| ENSSSCG00000002311 | SUSD6 | 2.067396627 | 1.1E-206 | UP |
| ENSSSCG00000037670 | TMEM164 | 2.540237224 | 1.1E-205 | UP |
| ENSSSCG00000017306 | ITGB3 | 2.044524375 | 3.2E-205 | UP |
| ENSSSCG00000017403 | STAT3 | 1.379371595 | 3.3E-205 | UP |
| ENSSSCG00000036556 | IL10RB | 1.830735124 | 7.2E-204 | UP |
| ENSSSCG00000010987 | UBAP2 | 1.244541465 | 5.4E-203 | UP |
| ENSSSCG00000036274 | NA | 1.928032767 | 6.1E-197 | UP |
| ENSSSCG00000006354 | TOMM40L | 2.007881772 | 6.9E-197 | UP |
| ENSSSCG00000025114 | FMNL3 | 1.576648496 | 7.4E-197 | UP |
| ENSSSCG00000014224 | SEMA6A | 2.684080521 | 5.8E-196 | UP |
| ENSSSCG00000004156 | IFNGR1 | 1.668086499 | 1.3E-191 | UP |
| ENSSSCG00000029002 | PNKD | 1.879864158 | 1.4E-190 | UP |
| ENSSSCG00000037536 | SLC25A28 | 2.230378997 | 1.2E-187 | UP |
| ENSSSCG00000039947 | KCNJ2 | 3.036800993 | 1.2E-185 | UP |
| ENSSSCG00000040663 | HERPUD1 | 1.259296738 | 2.7E-185 | UP |
| ENSSSCG00000006776 | MOV10 | 1.713193174 | 4.7E-183 | UP |
| ENSSSCG00000032715 | CERS6 | 2.785279974 | 5.1E-182 | UP |
| ENSSSCG00000025588 | FJX1 | 2.193679501 | 9.3E-182 | UP |
| ENSSSCG00000024096 | RIPK2 | 2.086620972 | 3.7E-181 | UP |
| ENSSSCG00000011198 | RFTN1 | 1.980599564 | 3.7E-181 | UP |
| ENSSSCG00000002376 | PGF | 2.92379782 | 6.2E-181 | UP |
| ENSSSCG00000001931 | NA | 2.978044649 | 1.8E-179 | UP |
| ENSSSCG00000004114 | RAB32 | 1.196339416 | 5.9E-179 | UP |
| ENSSSCG00000009633 | NA | 1.642651783 | 3.2E-177 | UP |
| ENSSSCG00000026893 | NA | 1.936381022 | 3.2E-177 | UP |
| ENSSSCG00000024771 | TICAM1 | 2.023763247 | 1.4E-176 | UP |
| ENSSSCG00000014780 | TRIM21 | 2.480467477 | 2.5E-176 | UP |
| ENSSSCG00000014420 | JAKMIP2 | 2.929226545 | 1.1E-175 | UP |
| ENSSSCG00000006187 | MSC | 4.168637009 | 2.1E-175 | UP |
| ENSSSCG00000014277 | IRF1 | 3.554328803 | 7.9E-175 | UP |
| ENSSSCG00000012014 | NA | 1.347178715 | 3.9E-174 | UP |
| ENSSSCG00000010054 | ADORA2A | 5.170018937 | 1.9E-173 | UP |
| ENSSSCG00000013408 | ADM | 1.894588916 | 4.3E-173 | UP |
| ENSSSCG00000012743 | MTMR1 | 1.704446819 | 7.2E-173 | UP |
| ENSSSCG00000032434 | PLAUR | 1.549371463 | 2.4E-172 | UP |
| ENSSSCG00000008954 | NA | 6.92527583 | 2.6E-171 | UP |
| ENSSSCG00000023738 | COG3 | 1.162978537 | 7.7E-171 | UP |
| ENSSSCG00000030108 | ZNFX1 | 2.57302776 | 7E-170 | UP |
| ENSSSCG00000040162 | NUPR1 | 2.208754542 | 1.8E-169 | UP |
| ENSSSCG00000035059 | MCM10 | 1.871793315 | 2.1E-169 | UP |
| ENSSSCG00000006718 | ZNF697 | 1.657796417 | 2.6E-168 | UP |
| ENSSSCG00000040061 | NINJ1 | 2.017046848 | 4.1E-167 | UP |
| ENSSSCG00000007554 | ZFAND2A | 1.699472221 | 4.6E-167 | UP |
| ENSSSCG00000022780 | UGCG | 2.295601944 | 1.2E-166 | UP |
| ENSSSCG00000001076 | RNF144B | 2.249412792 | 2.2E-166 | UP |
| ENSSSCG00000010448 | FAS | 1.834658329 | 1.7E-165 | UP |
| ENSSSCG00000020666 | EHD4 | 1.505865533 | 1.8E-165 | UP |
| ENSSSCG00000013766 | IL27RA | 1.404468983 | 9.1E-165 | UP |
| ENSSSCG00000012967 | FOSL1 | 1.646497334 | 5.3E-164 | UP |
| ENSSSCG00000025206 | RNF19B | 2.341717149 | 3.8E-163 | UP |
| ENSSSCG00000005944 | NDRG1 | 1.590283491 | 2.3E-162 | UP |
| ENSSSCG00000035101 | KLF5 | 2.0595646 | 2.1E-161 | UP |
| ENSSSCG00000007586 | FSCN1 | 1.400080439 | 2.1E-161 | UP |
| ENSSSCG00000005533 | PTGS1 | 2.561726064 | 2.4E-161 | UP |
| ENSSSCG00000015595 | ATF3 | 2.347085239 | 1E-159 | UP |
| ENSSSCG00000002340 | PSEN1 | 1.215558652 | 2.6E-159 | UP |
| ENSSSCG00000014987 | MMP12 | 8.381712648 | 4.3E-159 | UP |
| ENSSSCG00000032408 | CASP7 | 1.649613119 | 4.4E-159 | UP |
| ENSSSCG00000033258 | TCF21 | 2.16486365 | 6.4E-158 | UP |
| ENSSSCG00000038535 | ARSB | 1.493712715 | 1E-157 | UP |
| ENSSSCG00000011670 | PXYLP1 | 1.746115625 | 1.5E-157 | UP |
| ENSSSCG00000007007 | IDO1 | 9.895470417 | 4.9E-156 | UP |
| ENSSSCG00000003768 | NA | 1.561421968 | 3.3E-155 | UP |
| ENSSSCG00000002004 | PSME2 | 1.6756664 | 1.1E-154 | UP |
| ENSSSCG00000006875 | PLPPR4 | 2.506280094 | 1.4E-154 | UP |
| ENSSSCG00000005706 | ABL1 | 1.392486345 | 1.5E-154 | UP |
| ENSSSCG00000000146 | NA | 2.973602162 | 1.6E-154 | UP |
| ENSSSCG00000005224 | GLIS3 | 1.926787584 | 2.9E-154 | UP |
| ENSSSCG00000016322 | ACKR3 | 1.485868232 | 3.6E-154 | UP |
| ENSSSCG00000004670 | C15orf48 | 4.148717217 | 1.4E-152 | UP |
| ENSSSCG00000009396 | PHF11 | 1.25721333 | 2.2E-152 | UP |
| ENSSSCG00000001554 | SRPK1 | 1.547891619 | 2.2E-152 | UP |
| ENSSSCG00000004952 | SMAD3 | 1.30361121 | 3.4E-152 | UP |
| ENSSSCG00000009047 | SMARCA5 | 1.092318457 | 3.5E-152 | UP |
| ENSSSCG00000007469 | PTPN1 | 1.595719374 | 7.3E-152 | UP |
| ENSSSCG00000012173 | SAT1 | 2.206754069 | 3.3E-151 | UP |
| ENSSSCG00000034347 | TRIM56 | 1.170431555 | 5.1E-151 | UP |
| ENSSSCG00000006340 | UAP1 | 1.231636352 | 5.6E-151 | UP |
| ENSSSCG00000031789 | ACSL5 | 1.126788205 | 1.4E-150 | UP |
| ENSSSCG00000020705 | MAP3K8 | 2.720668218 | 1.8E-150 | UP |
| ENSSSCG00000038727 | GDNF | 2.295455207 | 2.6E-150 | UP |
| ENSSSCG00000009216 | SPP1 | 1.710137254 | 3.7E-150 | UP |
| ENSSSCG00000001951 | PSMA6 | 1.094442725 | 5.5E-150 | UP |
| ENSSSCG00000031781 | PSMB10 | 1.6130288 | 8.5E-150 | UP |
| ENSSSCG00000012277 | TIMP1 | 1.636313495 | 1.2E-148 | UP |
| ENSSSCG00000022011 | NMI | 2.083152141 | 2.5E-148 | UP |
| ENSSSCG00000006066 | RNF19A | 1.986571386 | 2.6E-148 | UP |
| ENSSSCG00000008374 | B3GNT2 | 1.543207139 | 9.8E-148 | UP |
| ENSSSCG00000011951 | NFKBIZ | 2.908946193 | 1E-147 | UP |
| ENSSSCG00000015603 | LPGAT1 | 1.217481656 | 3E-147 | UP |
| ENSSSCG00000013599 | ANGPTL4 | 1.893769381 | 3.2E-147 | UP |
| ENSSSCG00000026082 | DNAJC3 | 1.239234312 | 6E-147 | UP |
| ENSSSCG00000030560 | NA | 1.284651098 | 1.5E-146 | UP |
| ENSSSCG00000003590 | PTPRU | 1.360899714 | 7.5E-145 | UP |
| ENSSSCG00000024674 | ABL2 | 1.615761022 | 1.9E-144 | UP |
| ENSSSCG00000039330 | ANO8 | 1.240538091 | 2.5E-144 | UP |
| ENSSSCG00000015411 | PTPN12 | 1.485471573 | 3.5E-144 | UP |
| ENSSSCG00000002294 | ARG2 | 2.585580842 | 1.8E-143 | UP |
| ENSSSCG00000005593 | OLFML2A | 2.385915846 | 4.4E-143 | UP |
| ENSSSCG00000016512 | ZC3HAV1 | 1.776712765 | 8E-143 | UP |
| ENSSSCG00000003729 | RNF125 | 2.236690581 | 1.7E-142 | UP |
| ENSSSCG00000003983 | SMAP2 | 1.29767559 | 2.3E-142 | UP |
| ENSSSCG00000008641 | ADAM17 | 1.082996503 | 2.3E-142 | UP |
| ENSSSCG00000021586 | ZHX2 | 2.227201404 | 5.6E-142 | UP |
| ENSSSCG00000008208 | EIF2AK3 | 1.203260891 | 1E-140 | UP |
| ENSSSCG00000007864 | GPRC5B | 1.275704338 | 1.4E-140 | UP |
| ENSSSCG00000029230 | ECM1 | 1.598715659 | 1.7E-140 | UP |
| ENSSSCG00000005385 | NR4A3 | 4.106727903 | 1.3E-139 | UP |
| ENSSSCG00000023803 | ELK3 | 1.134986531 | 1.2E-138 | UP |
| ENSSSCG00000005935 | AGO2 | 1.608809065 | 1.2E-138 | UP |
| ENSSSCG00000035715 | GCH1 | 2.119645973 | 7E-138 | UP |
| ENSSSCG00000018057 | SOCS7 | 1.336233378 | 1.3E-136 | UP |
| ENSSSCG00000023630 | CPM | 3.598578731 | 2.4E-136 | UP |
| ENSSSCG00000011000 | DNAJA1 | 1.006693844 | 8.6E-136 | UP |
| ENSSSCG00000020783 | SLC41A1 | 1.237323398 | 2.9E-135 | UP |
| ENSSSCG00000015014 | ZC3H12C | 1.803355255 | 4.9E-135 | UP |
| ENSSSCG00000009865 | TBX3 | 2.022723656 | 9.2E-135 | UP |
| ENSSSCG00000006009 | EXT1 | 1.236441037 | 5.6E-134 | UP |
| ENSSSCG00000038366 | RAP2B | 1.426251082 | 5.9E-134 | UP |
| ENSSSCG00000031023 | NA | 2.739404523 | 8.9E-133 | UP |
| ENSSSCG00000016027 | ITGAV | 1.420161213 | 4E-132 | UP |
| ENSSSCG00000003839 | NA | 1.54416302 | 1.1E-131 | UP |
| ENSSSCG00000011465 | NA | 1.674461864 | 1.3E-129 | UP |
| ENSSSCG00000016230 | EPHA4 | 1.760094231 | 1.5E-129 | UP |
| ENSSSCG00000014997 | NA | 1.61136397 | 3.7E-129 | UP |
| ENSSSCG00000015375 | ITGB8 | 1.691505096 | 4.2E-129 | UP |
| ENSSSCG00000008624 | LPIN1 | 1.355603084 | 1.5E-128 | UP |
| ENSSSCG00000013236 | MYBPC3 | 6.007580713 | 2.2E-128 | UP |
| ENSSSCG00000010948 | NA | 2.435431856 | 9.9E-128 | UP |
| ENSSSCG00000023784 | SEMA3C | 1.140985926 | 1.6E-126 | UP |
| ENSSSCG00000026904 | NFKBIB | 1.477675726 | 3.6E-126 | UP |
| ENSSSCG00000017986 | NDEL1 | 1.027080687 | 8.2E-126 | UP |
| ENSSSCG00000011499 | LRIG1 | 2.060902795 | 2.4E-125 | UP |
| ENSSSCG00000015550 | RGS16 | 3.080885449 | 9.3E-124 | UP |
| ENSSSCG00000033879 | ZNF280B | 1.720987503 | 1.5E-123 | UP |
| ENSSSCG00000032517 | DMXL2 | 2.227847678 | 4.1E-123 | UP |
| ENSSSCG00000005423 | ABCA1 | 1.429931347 | 1.7E-122 | UP |
| ENSSSCG00000001233 | TRIM26 | 1.937063865 | 2.6E-122 | UP |
| ENSSSCG00000012262 | KDM6A | 1.319265661 | 7.3E-122 | UP |
| ENSSSCG00000040773 | TOB1 | 1.049753035 | 1.2E-121 | UP |
| ENSSSCG00000014985 | MMP3 | 3.739533327 | 1.9E-121 | UP |
| ENSSSCG00000034379 | MAP2K3 | 1.085571297 | 2E-121 | UP |
| ENSSSCG00000032474 | CXCL10 | 7.931850233 | 4.4E-121 | UP |
| ENSSSCG00000016653 | DNAJB9 | 1.456421708 | 1.6E-119 | UP |
| ENSSSCG00000004420 | TRAF3IP2 | 1.120778779 | 4.5E-119 | UP |
| ENSSSCG00000031462 | ZNRF1 | 1.314747684 | 6.7E-119 | UP |
| ENSSSCG00000009850 | TAOK3 | 1.199140777 | 7.6E-119 | UP |
| ENSSSCG00000001463 | PSMB9 | 3.261435115 | 1.3E-118 | UP |
| ENSSSCG00000027060 | TBX2 | 1.693639102 | 1.9E-117 | UP |
| ENSSSCG00000027762 | TNFRSF11B | 6.427599231 | 1.9E-117 | UP |
| ENSSSCG00000013369 | NA | 4.573048406 | 5.5E-117 | UP |
| ENSSSCG00000015872 | GPD2 | 1.513743092 | 1.7E-116 | UP |
| ENSSSCG00000023972 | DRAM1 | 1.557559719 | 2.4E-116 | UP |
| ENSSSCG00000016991 | DUSP1 | 1.713466084 | 2.9E-116 | UP |
| ENSSSCG00000016186 | TMBIM1 | 1.165410359 | 4.4E-116 | UP |
| ENSSSCG00000012074 | NA | 1.041025407 | 9.2E-116 | UP |
| ENSSSCG00000015871 | NR4A2 | 2.638356363 | 1.5E-115 | UP |
| ENSSSCG00000006651 | ADAMTSL4 | 2.029758323 | 1.8E-115 | UP |
| ENSSSCG00000011699 | HPS3 | 1.390796613 | 3.7E-115 | UP |
| ENSSSCG00000012548 | MUM1L1 | 2.111092442 | 4.3E-115 | UP |
| ENSSSCG00000040466 | FMN1 | 1.465526948 | 8.2E-115 | UP |
| ENSSSCG00000005083 | DHRS7 | 1.555139935 | 1.5E-114 | UP |
| ENSSSCG00000006023 | SYBU | 3.225032199 | 1.9E-114 | UP |
| ENSSSCG00000024914 | NA | 3.874992559 | 3E-114 | UP |
| ENSSSCG00000024108 | SLC43A2 | 1.527590323 | 3.3E-113 | UP |
| ENSSSCG00000007067 | JAG1 | 1.499104956 | 4E-113 | UP |
| ENSSSCG00000034692 | SH3BP4 | 1.291757385 | 3.9E-110 | UP |
| ENSSSCG00000022961 | CLMP | 1.134267224 | 4.1E-110 | UP |
| ENSSSCG00000003705 | CABLES1 | 1.366667157 | 8.9E-109 | UP |
| ENSSSCG00000012026 | ADAMTS1 | 1.979031426 | 1.4E-108 | UP |
| ENSSSCG00000031356 | HES1 | 1.926190261 | 4.8E-108 | UP |
| ENSSSCG00000011752 | FNDC3B | 1.029794312 | 6.6E-108 | UP |
| ENSSSCG00000031856 | DACT1 | 3.248503822 | 6.9E-108 | UP |
| ENSSSCG00000007484 | ZNF217 | 1.298717121 | 1.1E-107 | UP |
| ENSSSCG00000009446 | PCDH17 | 1.279042921 | 3.7E-107 | UP |
| ENSSSCG00000040445 | RND3 | 1.300457702 | 1.2E-106 | UP |
| ENSSSCG00000017488 | CSF3 | 8.075242592 | 1.2E-106 | UP |
| ENSSSCG00000010341 | TSPAN14 | 1.40429188 | 1.4E-106 | UP |
| ENSSSCG00000006209 | VCPIP1 | 1.529044861 | 2.5E-106 | UP |
| ENSSSCG00000026951 | PSMB8 | 1.989740791 | 2.7E-106 | UP |
| ENSSSCG00000016758 | NA | 1.268925982 | 8.6E-106 | UP |
| ENSSSCG00000011521 | PDZRN3 | 1.169566555 | 1.7E-105 | UP |
| ENSSSCG00000007079 | FLRT3 | 1.818993407 | 3.2E-105 | UP |
| ENSSSCG00000011298 | CDCP1 | 3.586672803 | 4.1E-105 | UP |
| ENSSSCG00000015823 | NA | 3.087478197 | 1E-104 | UP |
| ENSSSCG00000030642 | PCNA | 1.446170792 | 2.4E-104 | UP |
| ENSSSCG00000002841 | N4BP1 | 1.977864049 | 5.3E-104 | UP |
| ENSSSCG00000016981 | CPEB4 | 1.329177906 | 5.3E-104 | UP |
| ENSSSCG00000004657 | CEP152 | 1.887520386 | 5.3E-103 | UP |
| ENSSSCG00000036446 | PALD1 | 2.070822545 | 5.9E-103 | UP |
| ENSSSCG00000037216 | PPP4R2 | 1.224750796 | 2.1E-102 | UP |
| ENSSSCG00000034993 | NA | 1.685335479 | 3.5E-102 | UP |
| ENSSSCG00000023592 | TAC1 | 2.590891319 | 1.4E-101 | UP |
| ENSSSCG00000034012 | CASP3 | 1.336636861 | 1.5E-101 | UP |
| ENSSSCG00000016519 | AKR1D1 | 4.209394595 | 4.3E-101 | UP |
| ENSSSCG00000011859 | HEG1 | 1.381435732 | 1.1E-100 | UP |
| ENSSSCG00000015960 | MAP3K20 | 1.156031153 | 1.6E-100 | UP |
| ENSSSCG00000033321 | GAS1 | 1.138439552 | 2.5E-100 | UP |
| ENSSSCG00000017865 | CTNS | 1.163906008 | 3.4E-100 | UP |
| ENSSSCG00000004971 | TLE3 | 1.983385799 | 5.9E-100 | UP |
| ENSSSCG00000015667 | MBD5 | 1.687449256 | 1.9E-99 | UP |
| ENSSSCG00000001229 | NA | 1.571906166 | 7.2E-99 | UP |
| ENSSSCG00000003730 | RNF138 | 1.626326849 | 5.43E-98 | UP |
| ENSSSCG00000016502 | PARP12 | 2.130924311 | 1.47E-97 | UP |
| ENSSSCG00000006105 | GEM | 1.604106509 | 1.88E-97 | UP |
| ENSSSCG00000040887 | PAPD5 | 1.723737416 | 5.56E-97 | UP |
| ENSSSCG00000032320 | TCIM | 4.396198236 | 6.15E-97 | UP |
| ENSSSCG00000008388 | REL | 1.771938006 | 1.15E-96 | UP |
| ENSSSCG00000015302 | STEAP2 | 1.319553668 | 1.73E-96 | UP |
| ENSSSCG00000017991 | PIK3R5 | 3.796516188 | 2.09E-96 | UP |
| ENSSSCG00000002669 | CRISPLD2 | 1.216776823 | 3.28E-95 | UP |
| ENSSSCG00000011518 | SHQ1 | 1.878324345 | 5.08E-95 | UP |
| ENSSSCG00000001695 | VEGFA | 2.204203457 | 2.9E-94 | UP |
| ENSSSCG00000009172 | PPP3CA | 1.117410647 | 4.19E-94 | UP |
| ENSSSCG00000015545 | GLUL | 1.267471372 | 5.38E-94 | UP |
| ENSSSCG00000028304 | ZFP36L1 | 1.602845273 | 2.81E-93 | UP |
| ENSSSCG00000021383 | CGAS | 2.840041237 | 7.8E-93 | UP |
| ENSSSCG00000010329 | ZMIZ1 | 1.559947665 | 8.51E-93 | UP |
| ENSSSCG00000003155 | PPP1R15A | 1.232150874 | 9.34E-93 | UP |
| ENSSSCG00000000559 | RASSF8 | 1.296911635 | 2.32E-92 | UP |
| ENSSSCG00000035400 | YPEL2 | 1.846101342 | 3.01E-92 | UP |
| ENSSSCG00000014171 | ERAP1 | 1.348973293 | 3.45E-92 | UP |
| ENSSSCG00000011848 | TFRC | 1.355416998 | 3.6E-92 | UP |
| ENSSSCG00000011828 | FAM43A | 1.357392892 | 7.54E-92 | UP |
| ENSSSCG00000016254 | CCL20 | 11.44097035 | 1.08E-91 | UP |
| ENSSSCG00000016900 | ESM1 | 3.081969209 | 1.14E-91 | UP |
| ENSSSCG00000009320 | FLT1 | 4.013673719 | 1.34E-91 | UP |
| ENSSSCG00000030153 | SMURF1 | 1.37064126 | 1.78E-91 | UP |
| ENSSSCG00000015549 | RNASEL | 3.233694336 | 2.09E-91 | UP |
| ENSSSCG00000034364 | NA | 1.41420243 | 3.08E-91 | UP |
| ENSSSCG00000006127 | NBN | 1.710804682 | 1.96E-90 | UP |
| ENSSSCG00000006543 | ADAR | 1.368113054 | 4.39E-90 | UP |
| ENSSSCG00000009049 | USP38 | 1.09782293 | 8.71E-90 | UP |
| ENSSSCG00000001841 | RHCG | 3.329063395 | 8.87E-90 | UP |
| ENSSSCG00000021657 | SATB2 | 1.82008921 | 1.39E-89 | UP |
| ENSSSCG00000033830 | CCDC8 | 1.325016541 | 2.81E-89 | UP |
| ENSSSCG00000004830 | ATP10A | 1.771776171 | 4.02E-89 | UP |
| ENSSSCG00000012029 | BACH1 | 1.556537967 | 4.36E-89 | UP |
| ENSSSCG00000010219 | ARID5B | 1.095505845 | 1.01E-88 | UP |
| ENSSSCG00000010184 | AGT | 2.784967712 | 1.11E-88 | UP |
| ENSSSCG00000035634 | NA | 1.75252615 | 1.28E-88 | UP |
| ENSSSCG00000038410 | CPEB2 | 1.462385252 | 2.92E-88 | UP |
| ENSSSCG00000011874 | PARP14 | 4.474372103 | 1.11E-87 | UP |
| ENSSSCG00000010502 | CCNJ | 1.608754613 | 1.59E-87 | UP |
| ENSSSCG00000005288 | TLE4 | 1.370053845 | 1.98E-87 | UP |
| ENSSSCG00000015085 | IL10RA | 2.179585277 | 3.73E-87 | UP |
| ENSSSCG00000016031 | CALCRL | 4.065836789 | 6.05E-87 | UP |
| ENSSSCG00000009440 | ELF1 | 1.012908447 | 8.6E-87 | UP |
| ENSSSCG00000009129 | TIFA | 2.494301199 | 1.06E-86 | UP |
| ENSSSCG00000025618 | TAP1 | 2.310228303 | 1.17E-86 | UP |
| ENSSSCG00000032367 | CEBPD | 1.370581439 | 1.46E-86 | UP |
| ENSSSCG00000026940 | CASP10 | 1.252959313 | 3.05E-86 | UP |
| ENSSSCG00000027503 | OSBPL8 | 1.29529338 | 3.95E-86 | UP |
| ENSSSCG00000001394 | NA | 1.31213245 | 1.03E-85 | UP |
| ENSSSCG00000037358 | NA | 4.828778532 | 1.22E-85 | UP |
| ENSSSCG00000030484 | AHR | 1.097528661 | 1.46E-85 | UP |
| ENSSSCG00000003744 | MOCOS | 2.560746131 | 3.51E-85 | UP |
| ENSSSCG00000035940 | SPSB1 | 2.506190108 | 6.12E-85 | UP |
| ENSSSCG00000017756 | NLK | 1.500208444 | 9.81E-84 | UP |
| ENSSSCG00000017199 | TRIM47 | 1.506452175 | 2.53E-83 | UP |
| ENSSSCG00000016851 | OSMR | 1.225214943 | 3.08E-83 | UP |
| ENSSSCG00000009592 | NFIL3 | 1.25623637 | 4.31E-83 | UP |
| ENSSSCG00000024596 | NOCT | 2.040343564 | 7.52E-83 | UP |
| ENSSSCG00000001509 | DAXX | 1.535262137 | 8.45E-83 | UP |
| ENSSSCG00000035403 | RFX2 | 2.268794585 | 1.29E-82 | UP |
| ENSSSCG00000001507 | TAPBP | 1.135474805 | 2.17E-82 | UP |
| ENSSSCG00000026602 | PTGIR | 2.381877397 | 2.91E-82 | UP |
| ENSSSCG00000005208 | RIC1 | 1.103071736 | 3.12E-82 | UP |
| ENSSSCG00000024136 | AMPH | 2.051286173 | 4.64E-82 | UP |
| ENSSSCG00000000951 | NA | 1.062266977 | 5.6E-82 | UP |
| ENSSSCG00000016690 | CREB5 | 1.910881427 | 1.75E-81 | UP |
| ENSSSCG00000003513 | ECE1 | 1.756484103 | 5.24E-81 | UP |
| ENSSSCG00000012258 | FUNDC1 | 1.449932698 | 9.17E-81 | UP |
| ENSSSCG00000000475 | IRAK3 | 2.146114437 | 2.2E-80 | UP |
| ENSSSCG00000026382 | PPP2R5E | 1.162873525 | 2.47E-80 | UP |
| ENSSSCG00000009616 | HR | 1.492149073 | 6.48E-80 | UP |
| ENSSSCG00000008261 | HK2 | 1.390577788 | 6.77E-80 | UP |
| ENSSSCG00000004989 | FBXO33 | 1.131340393 | 8.07E-80 | UP |
| ENSSSCG00000009348 | STARD13 | 1.054287967 | 1.34E-79 | UP |
| ENSSSCG00000016233 | SERPINE2 | 1.049228745 | 1.51E-79 | UP |
| ENSSSCG00000034973 | CXCL12 | 2.489646939 | 1.51E-79 | UP |
| ENSSSCG00000017993 | NTN1 | 1.592397537 | 4.78E-79 | UP |
| ENSSSCG00000011493 | ATXN7 | 1.110381872 | 7.4E-79 | UP |
| ENSSSCG00000032433 | PTCHD1 | 2.784948465 | 1.01E-78 | UP |
| ENSSSCG00000016062 | NABP1 | 1.550796297 | 1.69E-78 | UP |
| ENSSSCG00000014136 | VCAN | 1.450681409 | 1.86E-78 | UP |
| ENSSSCG00000036911 | NA | 2.258686859 | 3.63E-78 | UP |
| ENSSSCG00000015897 | IFIH1 | 2.715554132 | 3.79E-78 | UP |
| ENSSSCG00000000148 | NA | 4.187158237 | 3.81E-78 | UP |
| ENSSSCG00000016518 | TRIM24 | 1.193858005 | 7.88E-78 | UP |
| ENSSSCG00000005472 | SLC46A2 | 4.291808347 | 1.38E-77 | UP |
| ENSSSCG00000034753 | FAM177A1 | 1.218452579 | 2.02E-77 | UP |
| ENSSSCG00000008123 | ARID5A | 2.722881081 | 2.09E-77 | UP |
| ENSSSCG00000010033 | PRR14L | 1.004777294 | 2.3E-77 | UP |
| ENSSSCG00000030507 | SMNDC1 | 1.338846851 | 9.63E-77 | UP |
| ENSSSCG00000029438 | SESN2 | 1.433518562 | 6.87E-76 | UP |
| ENSSSCG00000001849 | ANPEP | 1.059079413 | 1.25E-75 | UP |
| ENSSSCG00000002375 | RPS6KL1 | 1.638247102 | 2.86E-75 | UP |
| ENSSSCG00000010454 | IFIT5 | 2.397345675 | 2.91E-75 | UP |
| ENSSSCG00000035057 | RUNDC1 | 1.425830164 | 4.16E-75 | UP |
| ENSSSCG00000031147 | ACAP2 | 1.271518702 | 4.2E-75 | UP |
| ENSSSCG00000017755 | NOS2 | 11.01685968 | 5.26E-75 | UP |
| ENSSSCG00000036339 | FAM126B | 1.355320949 | 2.02E-74 | UP |
| ENSSSCG00000011047 | FAM171A1 | 1.154406431 | 2.6E-74 | UP |
| ENSSSCG00000016261 | SP110 | 1.610376243 | 3.1E-74 | UP |
| ENSSSCG00000034484 | SPEN | 1.165707536 | 6.96E-74 | UP |
| ENSSSCG00000006059 | NCALD | 3.556440715 | 7.87E-74 | UP |
| ENSSSCG00000038487 | TMPO | 1.465468766 | 2.52E-73 | UP |
| ENSSSCG00000025194 | ZSWIM6 | 1.443917237 | 3.25E-73 | UP |
| ENSSSCG00000035051 | ADORA2B | 1.095780074 | 5.08E-73 | UP |
| ENSSSCG00000034835 | SLC7A6 | 1.033716934 | 1.24E-72 | UP |
| ENSSSCG00000003079 | NA | 1.176552426 | 3.16E-72 | UP |
| ENSSSCG00000005211 | CD274 | 6.195664321 | 3.69E-72 | UP |
| ENSSSCG00000024973 | NA | 3.92041936 | 7.82E-72 | UP |
| ENSSSCG00000021738 | NA | 1.146533869 | 1.03E-71 | UP |
| ENSSSCG00000040904 | CLDN1 | 3.506866772 | 1.08E-71 | UP |
| ENSSSCG00000006917 | NA | 1.472075452 | 1.13E-71 | UP |
| ENSSSCG00000012112 | ARHGAP6 | 1.683724116 | 1.28E-71 | UP |
| ENSSSCG00000005457 | NA | 1.055890878 | 1.31E-71 | UP |
| ENSSSCG00000014016 | SQSTM1 | 1.026281274 | 8.35E-71 | UP |
| ENSSSCG00000010451 | IFIT2 | 4.33411257 | 9.83E-71 | UP |
| ENSSSCG00000013114 | SLC15A3 | 1.499737892 | 1.78E-70 | UP |
| ENSSSCG00000008090 | IL1A | 7.91813172 | 3.53E-70 | UP |
| ENSSSCG00000009429 | TNFSF11 | 5.896480207 | 1.08E-69 | UP |
| ENSSSCG00000003670 | RLF | 1.343267626 | 6.16E-69 | UP |
| ENSSSCG00000032015 | SH3BGRL2 | 1.667689224 | 7.29E-69 | UP |
| ENSSSCG00000011212 | RARB | 1.38768365 | 7.63E-69 | UP |
| ENSSSCG00000027646 | TIPARP | 1.399722965 | 9.96E-69 | UP |
| ENSSSCG00000005186 | TTC39B | 1.30643191 | 1.2E-68 | UP |
| ENSSSCG00000038180 | ADAMTS3 | 2.193718469 | 1.63E-68 | UP |
| ENSSSCG00000023710 | REEP1 | 3.509719304 | 2.47E-68 | UP |
| ENSSSCG00000004464 | FAM46A | 1.384022385 | 6.84E-68 | UP |
| ENSSSCG00000024174 | TGIF1 | 1.180056049 | 7.87E-68 | UP |
| ENSSSCG00000009786 | HIP1R | 3.545058868 | 1.59E-67 | UP |
| ENSSSCG00000010148 | ERO1B | 2.397935163 | 1.68E-67 | UP |
| ENSSSCG00000012996 | CDC42EP2 | 3.377493858 | 1.78E-67 | UP |
| ENSSSCG00000023907 | AFAP1 | 1.007354201 | 2.18E-67 | UP |
| ENSSSCG00000038521 | CHAC1 | 1.762090769 | 4.29E-67 | UP |
| ENSSSCG00000016243 | RHBDD1 | 1.037887597 | 5.04E-67 | UP |
| ENSSSCG00000031456 | ARL5B | 2.307809701 | 6.92E-67 | UP |
| ENSSSCG00000037267 | MAX | 1.063331825 | 7.12E-67 | UP |
| ENSSSCG00000003167 | FLT3LG | 1.373456352 | 7.53E-67 | UP |
| ENSSSCG00000035774 | ERRFI1 | 2.238090965 | 1.31E-66 | UP |
| ENSSSCG00000015299 | STEAP4 | 4.701944556 | 1.32E-66 | UP |
| ENSSSCG00000013551 | C3 | 5.223140331 | 2.36E-66 | UP |
| ENSSSCG00000027700 | RPRD1A | 1.152515594 | 8.55E-66 | UP |
| ENSSSCG00000021571 | KIF27 | 1.566151366 | 9.96E-66 | UP |
| ENSSSCG00000033089 | NA | 5.077055113 | 4.63E-65 | UP |
| ENSSSCG00000027529 | BIRC3 | 1.191644484 | 8.17E-65 | UP |
| ENSSSCG00000001912 | PML | 2.001834689 | 1.36E-64 | UP |
| ENSSSCG00000004687 | B2M | 1.39992672 | 1.4E-64 | UP |
| ENSSSCG00000016554 | MEST | 2.501955127 | 1.71E-64 | UP |
| ENSSSCG00000027806 | SAMHD1 | 1.389703741 | 1.98E-64 | UP |
| ENSSSCG00000015301 | STEAP1 | 1.073494695 | 3.18E-64 | UP |
| ENSSSCG00000006725 | TBX15 | 1.081687869 | 3.54E-64 | UP |
| ENSSSCG00000014869 | LRRC32 | 1.082670241 | 4.28E-64 | UP |
| ENSSSCG00000017551 | FAM117A | 1.874492378 | 4.4E-64 | UP |
| ENSSSCG00000028076 | ZBTB7C | 1.181600218 | 4.53E-64 | UP |
| ENSSSCG00000009157 | TET2 | 1.144169073 | 5.69E-64 | UP |
| ENSSSCG00000036033 | THRB | 1.03950488 | 6.2E-64 | UP |
| ENSSSCG00000023165 | SEMA7A | 2.60385688 | 6.23E-64 | UP |
| ENSSSCG00000000704 | TAPBPL | 1.168438043 | 6.79E-64 | UP |
| ENSSSCG00000015136 | UBASH3B | 1.301299905 | 8.63E-64 | UP |
| ENSSSCG00000013885 | FCHO1 | 7.202367933 | 1E-62 | UP |
| ENSSSCG00000030278 | MLLT11 | 1.58615709 | 1.17E-62 | UP |
| ENSSSCG00000010169 | SIPA1L2 | 2.089266372 | 1.31E-62 | UP |
| ENSSSCG00000010006 | NA | 1.085026377 | 1.58E-62 | UP |
| ENSSSCG00000039134 | NA | 1.169131033 | 2.75E-62 | UP |
| ENSSSCG00000000291 | GPR84 | 6.784684576 | 3.31E-62 | UP |
| ENSSSCG00000008617 | FAM49A | 2.55773481 | 3.61E-62 | UP |
| ENSSSCG00000003718 | TAF4B | 1.366735529 | 5.51E-62 | UP |
| ENSSSCG00000004897 | ZCCHC2 | 1.395095884 | 1.31E-61 | UP |
| ENSSSCG00000033909 | NA | 6.776190221 | 1.34E-61 | UP |
| ENSSSCG00000009535 | EFNB2 | 1.033156028 | 3.11E-61 | UP |
| ENSSSCG00000010452 | IFIT1 | 3.718481618 | 1.78E-60 | UP |
| ENSSSCG00000011972 | FILIP1L | 1.393121248 | 1.9E-60 | UP |
| ENSSSCG00000004195 | ARG1 | 5.982334657 | 2.51E-60 | UP |
| ENSSSCG00000021815 | NA | 2.147738255 | 2.63E-60 | UP |
| ENSSSCG00000040648 | CCL11 | 5.057490847 | 2.76E-60 | UP |
| ENSSSCG00000006919 | NA | 5.590759291 | 3.01E-60 | UP |
| ENSSSCG00000021598 | EVA1C | 2.387660587 | 3.21E-60 | UP |
| ENSSSCG00000037572 | EPSTI1 | 2.649877136 | 3.25E-60 | UP |
| ENSSSCG00000007000 | FAT1 | 1.213001548 | 4.79E-60 | UP |
| ENSSSCG00000027855 | SOCS1 | 3.850211795 | 5.39E-60 | UP |
| ENSSSCG00000024793 | PORCN | 1.093134231 | 8.81E-60 | UP |
| ENSSSCG00000008496 | EIF2AK2 | 1.964133529 | 1.86E-59 | UP |
| ENSSSCG00000000504 | PTPRB | 3.133699627 | 5.46E-59 | UP |
| ENSSSCG00000005683 | TOR1B | 1.109932592 | 1.04E-58 | UP |
| ENSSSCG00000032652 | NA | 2.856516573 | 2.27E-58 | UP |
| ENSSSCG00000013457 | DOT1L | 1.147565713 | 2.34E-58 | UP |
| ENSSSCG00000036113 | NA | 2.397365879 | 6.41E-58 | UP |
| ENSSSCG00000033952 | CITED4 | 6.355945003 | 8.82E-58 | UP |
| ENSSSCG00000001805 | WHAMM | 1.178353277 | 9.29E-58 | UP |
| ENSSSCG00000033037 | NA | 1.732374766 | 1.1E-57 | UP |
| ENSSSCG00000008820 | TEC | 2.501191399 | 1.63E-57 | UP |
| ENSSSCG00000012853 | IRF7 | 2.568682199 | 4.29E-57 | UP |
| ENSSSCG00000016262 | NA | 1.206700973 | 5.46E-57 | UP |
| ENSSSCG00000004053 | TAGAP | 2.27339311 | 1.06E-56 | UP |
| ENSSSCG00000008727 | MSX1 | 2.259684323 | 1.15E-56 | UP |
| ENSSSCG00000007034 | NA | 1.017300194 | 1.19E-56 | UP |
| ENSSSCG00000011251 | MYD88 | 1.005255858 | 1.19E-56 | UP |
| ENSSSCG00000009029 | ARHGAP10 | 1.088710162 | 2.02E-56 | UP |
| ENSSSCG00000004121 | FBXO30 | 1.190268502 | 2.93E-56 | UP |
| ENSSSCG00000037087 | PRRT4 | 4.305954583 | 5.16E-56 | UP |
| ENSSSCG00000033367 | PLEKHA2 | 1.153803362 | 6.33E-56 | UP |
| ENSSSCG00000039998 | CLIC2 | 1.639507057 | 6.69E-56 | UP |
| ENSSSCG00000030408 | DDX58 | 3.36413406 | 6.91E-56 | UP |
| ENSSSCG00000015644 | IKBKE | 1.086423223 | 7.92E-56 | UP |
| ENSSSCG00000023379 | UBE2L6 | 2.228354705 | 1.2E-55 | UP |
| ENSSSCG00000021646 | KLF9 | 1.128904402 | 1.58E-55 | UP |
| ENSSSCG00000035791 | SIX5 | 1.341107569 | 1.76E-55 | UP |
| ENSSSCG00000006864 | CDC14A | 1.618813894 | 1.96E-55 | UP |
| ENSSSCG00000012631 | LONRF3 | 1.057734708 | 2.69E-55 | UP |
| ENSSSCG00000001561 | ETV7 | 4.554494096 | 2.88E-55 | UP |
| ENSSSCG00000005724 | SETX | 1.651376577 | 4.38E-55 | UP |
| ENSSSCG00000017258 | FAM20A | 1.587289018 | 1.24E-54 | UP |
| ENSSSCG00000022159 | FNDC3A | 1.013323453 | 1.51E-54 | UP |
| ENSSSCG00000012055 | MORC3 | 1.414602575 | 3.59E-54 | UP |
| ENSSSCG00000008648 | RSAD2 | 5.322106154 | 7.47E-54 | UP |
| ENSSSCG00000027652 | KLHL15 | 1.140517017 | 2.15E-53 | UP |
| ENSSSCG00000004149 | NHSL1 | 1.634877094 | 3.29E-53 | UP |
| ENSSSCG00000025560 | PGLYRP2 | 3.469298655 | 6.48E-53 | UP |
| ENSSSCG00000015255 | IGSF9B | 2.983369715 | 9.37E-53 | UP |
| ENSSSCG00000004383 | OSTM1 | 1.044512249 | 1.08E-52 | UP |
| ENSSSCG00000036520 | EFNA5 | 1.46129271 | 1.13E-52 | UP |
| ENSSSCG00000015846 | RBPMS | 1.057400363 | 1.19E-52 | UP |
| ENSSSCG00000006742 | MAB21L3 | 2.270451522 | 2.58E-52 | UP |
| ENSSSCG00000038505 | MSI2 | 1.774096586 | 4.58E-52 | UP |
| ENSSSCG00000011133 | PFKFB3 | 1.326852809 | 4.68E-52 | UP |
| ENSSSCG00000016057 | STAT1 | 1.592327074 | 9.18E-52 | UP |
| ENSSSCG00000027372 | SAMD9 | 1.720630863 | 1E-51 | UP |
| ENSSSCG00000016656 | ELMO1 | 1.178337889 | 3.55E-51 | UP |
| ENSSSCG00000012202 | NA | 1.321079595 | 6.44E-51 | UP |
| ENSSSCG00000009998 | CASTOR1 | 1.40066033 | 1.17E-50 | UP |
| ENSSSCG00000034610 | NA | 1.853041751 | 1.26E-50 | UP |
| ENSSSCG00000028964 | PIM2 | 2.052888836 | 1.26E-50 | UP |
| ENSSSCG00000015390 | NA | 1.001984978 | 1.43E-50 | UP |
| ENSSSCG00000015801 | TLR3 | 1.453992341 | 1.66E-50 | UP |
| ENSSSCG00000032532 | CHRM2 | 4.26267313 | 1.78E-50 | UP |
| ENSSSCG00000010235 | SIRT1 | 1.099757512 | 1.88E-50 | UP |
| ENSSSCG00000017645 | TEX14 | 5.507270193 | 2.17E-50 | UP |
| ENSSSCG00000013382 | PLEKHA7 | 4.553753973 | 2.33E-50 | UP |
| ENSSSCG00000012076 | MX2 | 4.294906195 | 2.72E-50 | UP |
| ENSSSCG00000015436 | CCDC71L | 1.204363997 | 3.1E-50 | UP |
| ENSSSCG00000015037 | IL18 | 1.441150786 | 9.15E-50 | UP |
| ENSSSCG00000010438 | ATAD1 | 1.129273414 | 1.01E-49 | UP |
| ENSSSCG00000029507 | RASGEF1B | 3.779741445 | 1.78E-49 | UP |
| ENSSSCG00000015525 | TOR3A | 1.07661653 | 2.08E-49 | UP |
| ENSSSCG00000034167 | SLC5A3 | 1.265512216 | 2.18E-49 | UP |
| ENSSSCG00000014976 | ARHGAP42 | 1.06708368 | 4.04E-49 | UP |
| ENSSSCG00000017274 | PITPNC1 | 1.559641461 | 3.93E-48 | UP |
| ENSSSCG00000027860 | ERAP2 | 1.690656575 | 1.41E-47 | UP |
| ENSSSCG00000025286 | MCTP1 | 1.685002847 | 3.23E-47 | UP |
| ENSSSCG00000006923 | GBP2 | 2.742614836 | 3.35E-47 | UP |
| ENSSSCG00000012077 | MX1 | 2.458602831 | 4.65E-47 | UP |
| ENSSSCG00000014362 | HBEGF | 2.402266622 | 7.66E-47 | UP |
| ENSSSCG00000000567 | SOX5 | 1.256378136 | 8.45E-47 | UP |
| ENSSSCG00000013303 | ABTB2 | 3.44067256 | 1.91E-46 | UP |
| ENSSSCG00000021612 | ARHGAP28 | 1.086650872 | 3.2E-46 | UP |
| ENSSSCG00000015782 | IRF2 | 2.054613875 | 5.49E-46 | UP |
| ENSSSCG00000025053 | RYBP | 1.305984539 | 7.43E-46 | UP |
| ENSSSCG00000017700 | CCL3L1 | 4.196573512 | 6.23E-45 | UP |
| ENSSSCG00000008334 | MXD1 | 2.290043113 | 6.61E-45 | UP |
| ENSSSCG00000012134 | PIGA | 1.256659253 | 1.23E-44 | UP |
| ENSSSCG00000031610 | NA | 4.975320299 | 1.39E-44 | UP |
| ENSSSCG00000023178 | BATF2 | 3.671024646 | 1.65E-44 | UP |
| ENSSSCG00000040815 | DUSP5 | 1.796164483 | 1.74E-44 | UP |
| ENSSSCG00000001061 | JARID2 | 1.696652344 | 1.76E-44 | UP |
| ENSSSCG00000030801 | NA | 4.215314744 | 3.42E-44 | UP |
| ENSSSCG00000002252 | ARRDC4 | 2.05365524 | 3.52E-44 | UP |
| ENSSSCG00000017614 | TRIM25 | 1.549213133 | 3.58E-44 | UP |
| ENSSSCG00000001347 | PPP1R10 | 1.137810482 | 3.66E-44 | UP |
| ENSSSCG00000006625 | RFX5 | 1.337607894 | 4.77E-44 | UP |
| ENSSSCG00000032644 | HLX | 1.296989145 | 4.95E-44 | UP |
| ENSSSCG00000010772 | ADAM8 | 2.163838003 | 5.32E-44 | UP |
| ENSSSCG00000040575 | ISG15 | 3.220440922 | 5.86E-44 | UP |
| ENSSSCG00000009705 | GALNT7 | 1.07546481 | 8.55E-44 | UP |
| ENSSSCG00000015649 | DYRK3 | 1.546650218 | 1.19E-43 | UP |
| ENSSSCG00000032705 | USP42 | 1.067022039 | 1.39E-43 | UP |
| ENSSSCG00000034260 | GDF15 | 1.085365055 | 1.69E-43 | UP |
| ENSSSCG00000024219 | TIGAR | 1.44155253 | 1.89E-43 | UP |
| ENSSSCG00000029296 | FOXC2 | 2.168142449 | 1.94E-43 | UP |
| ENSSSCG00000035181 | RNF24 | 1.057444784 | 2.17E-43 | UP |
| ENSSSCG00000008348 | PLEK | 2.176081254 | 2.92E-43 | UP |
| ENSSSCG00000009827 | HVCN1 | 1.34870068 | 4.09E-43 | UP |
| ENSSSCG00000035077 | INHBA | 2.495820641 | 4.42E-43 | UP |
| ENSSSCG00000015569 | SWT1 | 1.471203403 | 8.84E-43 | UP |
| ENSSSCG00000027709 | PARP9 | 2.109388911 | 1.15E-42 | UP |
| ENSSSCG00000033613 | FOXS1 | 3.048141257 | 1.51E-42 | UP |
| ENSSSCG00000022446 | SEL1L3 | 1.28594567 | 1.58E-42 | UP |
| ENSSSCG00000009477 | EDNRB | 4.467469576 | 1.73E-42 | UP |
| ENSSSCG00000004075 | RGS17 | 1.25135541 | 2.22E-42 | UP |
| ENSSSCG00000009469 | ACOD1 | 9.204790283 | 2.33E-42 | UP |
| ENSSSCG00000039442 | BMP2 | 2.419158115 | 2.64E-42 | UP |
| ENSSSCG00000037483 | RFTN2 | 1.044762987 | 9.04E-42 | UP |
| ENSSSCG00000004332 | BACH2 | 5.085361458 | 9.64E-42 | UP |
| ENSSSCG00000016140 | FZD5 | 1.811557962 | 1.53E-41 | UP |
| ENSSSCG00000005364 | TDRD7 | 1.059477102 | 1.68E-41 | UP |
| ENSSSCG00000017416 | DHX58 | 2.366195729 | 2.29E-41 | UP |
| ENSSSCG00000024022 | TRPC6 | 5.347436154 | 3.02E-41 | UP |
| ENSSSCG00000017420 | CNP | 1.630865947 | 3.07E-41 | UP |
| ENSSSCG00000009979 | NEFH | 3.918162249 | 3.44E-41 | UP |
| ENSSSCG00000004859 | ZNF516 | 1.132115076 | 3.83E-41 | UP |
| ENSSSCG00000032436 | NA | 1.615194035 | 4.72E-41 | UP |
| ENSSSCG00000036136 | BHLHE40 | 1.000348409 | 9.36E-41 | UP |
| ENSSSCG00000030548 | HERC5 | 2.85691734 | 1.7E-40 | UP |
| ENSSSCG00000017087 | GM2A | 1.225768335 | 2.56E-40 | UP |
| ENSSSCG00000021712 | HERC6 | 2.503779703 | 2.96E-40 | UP |
| ENSSSCG00000009431 | DGKH | 1.116694753 | 4.72E-40 | UP |
| ENSSSCG00000010071 | MMP11 | 1.258731298 | 8.18E-40 | UP |
| ENSSSCG00000008647 | CMPK2 | 3.409341287 | 1.26E-39 | UP |
| ENSSSCG00000004902 | RNF152 | 1.924492866 | 1.3E-39 | UP |
| ENSSSCG00000009240 | PLAC8 | 2.74150781 | 1.36E-39 | UP |
| ENSSSCG00000028552 | BHLHE41 | 1.062258107 | 1.99E-39 | UP |
| ENSSSCG00000031262 | TXNIP | 1.677561395 | 2.47E-39 | UP |
| ENSSSCG00000017797 | SLC6A4 | 2.608683164 | 2.59E-39 | UP |
| ENSSSCG00000023957 | CFAP126 | 3.285277612 | 6.62E-39 | UP |
| ENSSSCG00000039751 | NLRC5 | 3.379219051 | 1.28E-38 | UP |
| ENSSSCG00000039761 | MYCL | 4.004556842 | 2.37E-38 | UP |
| ENSSSCG00000002267 | RGMA | 1.020900631 | 2.9E-38 | UP |
| ENSSSCG00000016568 | AHCYL2 | 1.350210704 | 3.03E-38 | UP |
| ENSSSCG00000031794 | TNFAIP8L3 | 2.267993743 | 3.48E-38 | UP |
| ENSSSCG00000017886 | FBXO39 | 2.814981083 | 4.07E-38 | UP |
| ENSSSCG00000036340 | ZBTB5 | 2.487880489 | 7.77E-38 | UP |
| ENSSSCG00000000774 | USP18 | 2.679188531 | 9.05E-38 | UP |
| ENSSSCG00000014565 | NA | 1.083755744 | 1.02E-37 | UP |
| ENSSSCG00000038471 | NUAK2 | 1.843805144 | 1.09E-37 | UP |
| ENSSSCG00000019154 | ssc-mir-155 | 2.588276727 | 1.2E-37 | UP |
| ENSSSCG00000031882 | PNPT1 | 1.020637597 | 1.21E-37 | UP |
| ENSSSCG00000007485 | BCAS1 | 2.955903689 | 1.61E-37 | UP |
| ENSSSCG00000009542 | TNFSF13B | 5.005812434 | 1.64E-37 | UP |
| ENSSSCG00000000717 | KCNA6 | 1.691016984 | 7.08E-37 | UP |
| ENSSSCG00000029592 | GPRC5A | 1.80057802 | 8.72E-37 | UP |
| ENSSSCG00000034980 | IRF8 | 6.405934768 | 1.81E-36 | UP |
| ENSSSCG00000034802 | NA | 1.661992794 | 2.96E-36 | UP |
| ENSSSCG00000011239 | NA | 2.989991223 | 4.48E-36 | UP |
| ENSSSCG00000004755 | DLL4 | 4.853887475 | 6.43E-36 | UP |
| ENSSSCG00000035153 | TRIM38 | 1.439876839 | 6.46E-36 | UP |
| ENSSSCG00000016687 | CHN2 | 1.027270172 | 7.76E-36 | UP |
| ENSSSCG00000008383 | NA | 1.129580263 | 1.07E-35 | UP |
| ENSSSCG00000029160 | NA | 1.421453324 | 1.21E-35 | UP |
| ENSSSCG00000017962 | KDM6B | 1.713475095 | 1.7E-35 | UP |
| ENSSSCG00000035420 | HES4 | 3.487500732 | 2.05E-35 | UP |
| ENSSSCG00000007508 | ZBP1 | 2.85475257 | 4.45E-35 | UP |
| ENSSSCG00000005269 | TRPM6 | 2.499466869 | 1.93E-34 | UP |
| ENSSSCG00000040036 | NA | 7.386542765 | 2.63E-34 | UP |
| ENSSSCG00000009293 | NA | 2.022311163 | 2.97E-34 | UP |
| ENSSSCG00000014156 | ARRDC3 | 1.287627002 | 3.85E-34 | UP |
| ENSSSCG00000006360 | B4GALT3 | 1.057471698 | 1.34E-33 | UP |
| ENSSSCG00000034858 | RAP1GAP2 | 2.180874191 | 1.34E-33 | UP |
| ENSSSCG00000022246 | NA | 1.207213731 | 3.37E-33 | UP |
| ENSSSCG00000025788 | ENPP4 | 1.932494122 | 6.94E-33 | UP |
| ENSSSCG00000013296 | SLC1A2 | 2.632899784 | 7.66E-33 | UP |
| ENSSSCG00000011876 | DTX3L | 2.859901434 | 1.01E-32 | UP |
| ENSSSCG00000036063 | LPAR6 | 1.512933317 | 1.25E-32 | UP |
| ENSSSCG00000035867 | GFOD1 | 1.168350749 | 1.37E-32 | UP |
| ENSSSCG00000032778 | PLEKHG1 | 1.682962668 | 1.45E-32 | UP |
| ENSSSCG00000016853 | RICTOR | 1.12100792 | 1.71E-32 | UP |
| ENSSSCG00000037530 | NA | 2.112215519 | 2.09E-32 | UP |
| ENSSSCG00000008664 | FAM84A | 3.923356409 | 2.7E-32 | UP |
| ENSSSCG00000024071 | SCARF1 | 1.303131839 | 2.78E-32 | UP |
| ENSSSCG00000009653 | CDCA2 | 1.096804741 | 3.65E-32 | UP |
| ENSSSCG00000037452 | HTR2A | 2.117762066 | 4.69E-32 | UP |
| ENSSSCG00000009881 | OAS2 | 2.341420698 | 1.27E-31 | UP |
| ENSSSCG00000023085 | STAC | 1.895517234 | 1.99E-31 | UP |
| ENSSSCG00000011678 | NA | 1.042674545 | 2.3E-31 | UP |
| ENSSSCG00000006648 | CTSS | 1.050772422 | 2.89E-31 | UP |
| ENSSSCG00000039042 | NA | 2.720484325 | 5.87E-31 | UP |
| ENSSSCG00000034989 | LRRTM2 | 2.59757072 | 9.73E-31 | UP |
| ENSSSCG00000017548 | NGFR | 1.989157469 | 1.59E-30 | UP |
| ENSSSCG00000040010 | BCL2A1 | 1.512964776 | 2.22E-30 | UP |
| ENSSSCG00000037116 | ZNRF2 | 2.395600447 | 3.1E-30 | UP |
| ENSSSCG00000016792 | RETREG1 | 1.167080765 | 4.53E-30 | UP |
| ENSSSCG00000015715 | EN1 | 3.495401776 | 5.8E-30 | UP |
| ENSSSCG00000032360 | PANX1 | 1.138362613 | 7.19E-30 | UP |
| ENSSSCG00000020906 | TNFSF10 | 3.447603277 | 3.24E-29 | UP |
| ENSSSCG00000010444 | LIPM | 1.166076587 | 4.33E-29 | UP |
| ENSSSCG00000009720 | DDX60 | 2.626903555 | 4.62E-29 | UP |
| ENSSSCG00000006288 | SELP | 4.615565744 | 6.58E-29 | UP |
| ENSSSCG00000009395 | SETDB2 | 1.09103423 | 8.96E-29 | UP |
| ENSSSCG00000017146 | RNF213 | 1.67944528 | 1E-28 | UP |
| ENSSSCG00000004369 | PRDM1 | 5.21395269 | 1.29E-28 | UP |
| ENSSSCG00000033222 | TRIM14 | 1.359428773 | 1.45E-28 | UP |
| ENSSSCG00000002032 | SLC7A8 | 1.618303738 | 1.68E-28 | UP |
| ENSSSCG00000013360 | TMEM86A | 1.910046666 | 1.69E-28 | UP |
| ENSSSCG00000022312 | RHPN2 | 2.768504725 | 2.28E-28 | UP |
| ENSSSCG00000008835 | RASL11B | 1.037107045 | 2.54E-28 | UP |
| ENSSSCG00000000728 | PARP11 | 1.207715493 | 1.36E-27 | UP |
| ENSSSCG00000025134 | FAM171B | 1.411147574 | 2.04E-27 | UP |
| ENSSSCG00000015324 | GNG11 | 1.114538805 | 3.4E-27 | UP |
| ENSSSCG00000009482 | SPRY2 | 1.356129356 | 3.68E-27 | UP |
| ENSSSCG00000012265 | CHST7 | 1.91576852 | 4.23E-27 | UP |
| ENSSSCG00000005992 | SHAS2 | 1.857267305 | 4.72E-27 | UP |
| ENSSSCG00000039182 | C11orf96 | 1.323451617 | 6.31E-27 | UP |
| ENSSSCG00000032709 | ARL4A | 2.602610105 | 6.4E-27 | UP |
| ENSSSCG00000017754 | NA | 2.722257911 | 7.12E-27 | UP |
| ENSSSCG00000020872 | NA | 2.259656329 | 9.21E-27 | UP |
| ENSSSCG00000027935 | FHOD3 | 1.144686879 | 1.32E-26 | UP |
| ENSSSCG00000016986 | CREBRF | 1.11063787 | 5.73E-26 | UP |
| ENSSSCG00000014618 | NA | 5.524000084 | 1.23E-25 | UP |
| ENSSSCG00000026701 | NA | 3.766981059 | 2.89E-25 | UP |
| ENSSSCG00000010449 | CH25H | 3.883498794 | 5.13E-25 | UP |
| ENSSSCG00000010338 | DYDC1 | 2.30684031 | 7.08E-25 | UP |
| ENSSSCG00000003137 | PLEKHA4 | 1.433224906 | 8.82E-25 | UP |
| ENSSSCG00000025836 | SULT1C4 | 2.422578858 | 2.05E-24 | UP |
| ENSSSCG00000038783 | IGFBP3 | 3.058245428 | 2.23E-24 | UP |
| ENSSSCG00000005504 | BRINP1 | 4.515960991 | 2.68E-24 | UP |
| ENSSSCG00000029449 | NA | 2.381813662 | 3.16E-24 | UP |
| ENSSSCG00000006173 | GDAP1 | 1.35204047 | 3.31E-24 | UP |
| ENSSSCG00000040617 | TNFAIP8 | 1.269828526 | 5.17E-24 | UP |
| ENSSSCG00000025856 | TMEM106A | 1.103677503 | 8.11E-24 | UP |
| ENSSSCG00000016521 | DGKI | 3.210165551 | 6E-23 | UP |
| ENSSSCG00000024671 | WNT9A | 1.418122894 | 7.37E-23 | UP |
| ENSSSCG00000017605 | MMD | 1.079663875 | 2.25E-22 | UP |
| ENSSSCG00000034087 | TNFSF15 | 1.982919365 | 2.91E-22 | UP |
| ENSSSCG00000038929 | CEMIP | 1.222011968 | 8.46E-22 | UP |
| ENSSSCG00000034369 | SPSB4 | 2.125911694 | 1.07E-21 | UP |
| ENSSSCG00000009664 | PTK2B | 1.03772749 | 1.94E-21 | UP |
| ENSSSCG00000008963 | AREG | 1.581932122 | 2.39E-21 | UP |
| ENSSSCG00000015403 | HGF | 1.769972268 | 2.43E-21 | UP |
| ENSSSCG00000007559 | MAFK | 1.248339413 | 3.06E-21 | UP |
| ENSSSCG00000006737 | IGSF3 | 2.765608481 | 3.25E-21 | UP |
| ENSSSCG00000023796 | NA | 3.91534027 | 4.72E-21 | UP |
| ENSSSCG00000040673 | TMEM140 | 2.826549788 | 5.49E-21 | UP |
| ENSSSCG00000036383 | LGALS3BP | 1.611609937 | 5.97E-21 | UP |
| ENSSSCG00000033453 | BST2 | 1.906177938 | 2.01E-20 | UP |
| ENSSSCG00000010509 | PIK3AP1 | 3.095368386 | 2.04E-20 | UP |
| ENSSSCG00000032475 | CEP44 | 1.191855857 | 2.11E-20 | UP |
| ENSSSCG00000008796 | RBM47 | 4.735082996 | 2.37E-20 | UP |
| ENSSSCG00000010340 | FAM213A | 1.134720524 | 2.9E-20 | UP |
| ENSSSCG00000004165 | PDE7B | 1.157551642 | 6.4E-20 | UP |
| ENSSSCG00000004629 | LYSMD2 | 1.237235665 | 7.73E-20 | UP |
| ENSSSCG00000015476 | CHI3L1 | 4.760731386 | 9.51E-20 | UP |
| ENSSSCG00000023374 | SRGN | 3.149512772 | 1.12E-19 | UP |
| ENSSSCG00000005589 | NR6A1 | 1.488388157 | 1.15E-19 | UP |
| ENSSSCG00000023004 | FZD9 | 2.640709188 | 1.19E-19 | UP |
| ENSSSCG00000035612 | COX6B2 | 1.974695556 | 1.33E-19 | UP |
| ENSSSCG00000004218 | RSPO3 | 2.392152648 | 1.39E-19 | UP |
| ENSSSCG00000033871 | ANAPC10 | 1.062054019 | 1.55E-19 | UP |
| ENSSSCG00000023737 | CSF2 | 10.9890004 | 2.72E-19 | UP |
| ENSSSCG00000005994 | SNTB1 | 2.907308617 | 3.73E-19 | UP |
| ENSSSCG00000016959 | MARVELD2 | 3.578099997 | 4.14E-19 | UP |
| ENSSSCG00000017921 | ZMYND15 | 3.623171651 | 5.42E-19 | UP |
| ENSSSCG00000016053 | NA | 1.382691404 | 5.81E-19 | UP |
| ENSSSCG00000040550 | CASC1 | 1.084224188 | 7.2E-19 | UP |
| ENSSSCG00000026499 | NMT2 | 1.026036834 | 7.2E-19 | UP |
| ENSSSCG00000031924 | NKX3-1 | 3.254803223 | 9.68E-19 | UP |
| ENSSSCG00000034015 | NA | 10.68595248 | 1.17E-18 | UP |
| ENSSSCG00000001404 | TNF | 9.229411273 | 1.4E-18 | UP |
| ENSSSCG00000037735 | NA | 10.34991491 | 1.5E-17 | UP |
| ENSSSCG00000009408 | LRCH1 | 1.00066626 | 2.22E-17 | UP |
| ENSSSCG00000012960 | CST6 | 6.572675849 | 2.39E-17 | UP |
| ENSSSCG00000012066 | KCNJ15 | 2.725811071 | 2.83E-17 | UP |
| ENSSSCG00000025097 | TMEM61 | 1.196509511 | 8E-17 | UP |
| ENSSSCG00000013418 | CFD | 3.412224303 | 8.15E-17 | UP |
| ENSSSCG00000021557 | SULT1A3 | 1.245625932 | 8.27E-17 | UP |
| ENSSSCG00000027660 | IFI44L | 1.229258244 | 1.16E-16 | UP |
| ENSSSCG00000012375 | DLG3 | 2.05805886 | 1.63E-16 | UP |
| ENSSSCG00000000433 | B4GALNT1 | 1.354210768 | 1.94E-16 | UP |
| ENSSSCG00000022447 | F3 | 1.335847071 | 2.6E-16 | UP |
| ENSSSCG00000015598 | TMEM206 | 1.003277312 | 6.07E-16 | UP |
| ENSSSCG00000008742 | CD38 | 3.392968331 | 7.29E-16 | UP |
| ENSSSCG00000015959 | RAPGEF4 | 3.204268871 | 7.72E-16 | UP |
| ENSSSCG00000004493 | SIGLEC15 | 4.194385353 | 7.87E-16 | UP |
| ENSSSCG00000032911 | DUSP8 | 1.397484108 | 8.08E-16 | UP |
| ENSSSCG00000013307 | LMO2 | 1.328507791 | 1.08E-15 | UP |
| ENSSSCG00000008311 | CYP26B1 | 1.457213693 | 1.36E-15 | UP |
| ENSSSCG00000033327 | PDGFB | 4.260691598 | 1.39E-15 | UP |
| ENSSSCG00000005375 | CORO2A | 1.231822706 | 1.55E-15 | UP |
| ENSSSCG00000022029 | RAP1GAP | 1.785183038 | 2.02E-15 | UP |
| ENSSSCG00000021576 | CD83 | 1.270922427 | 2.05E-15 | UP |
| ENSSSCG00000032149 | PLET1 | 9.670175857 | 2.23E-15 | UP |
| ENSSSCG00000014993 | NA | 1.111032826 | 2.38E-15 | UP |
| ENSSSCG00000014221 | LVRN | 2.43772736 | 6.08E-15 | UP |
| ENSSSCG00000033520 | IL23A | 6.883360145 | 8.4E-15 | UP |
| ENSSSCG00000025667 | FBXO2 | 1.667067169 | 1.05E-14 | UP |
| ENSSSCG00000022405 | P2RX1 | 8.129747497 | 1.22E-14 | UP |
| ENSSSCG00000037132 | POU2F2 | 5.46847335 | 1.45E-14 | UP |
| ENSSSCG00000036064 | CALHM6 | 4.668623526 | 2.32E-14 | UP |
| ENSSSCG00000037642 | ARID3A | 1.083567804 | 3.23E-14 | UP |
| ENSSSCG00000022833 | FGF16 | 4.946757502 | 3.41E-14 | UP |
| ENSSSCG00000008768 | ARAP2 | 2.362663325 | 3.88E-14 | UP |
| ENSSSCG00000010339 | DYDC2 | 2.90840116 | 4.09E-14 | UP |
| ENSSSCG00000016642 | GPR85 | 3.095208062 | 4.51E-14 | UP |
| ENSSSCG00000033787 | NA | 2.475375624 | 5.79E-14 | UP |
| ENSSSCG00000017590 | NA | 2.957690109 | 6.23E-14 | UP |
| ENSSSCG00000013654 | NA | 2.361340661 | 7.58E-14 | UP |
| ENSSSCG00000034570 | IFI6 | 1.442319479 | 4.75E-13 | UP |
| ENSSSCG00000024161 | NA | 1.798083351 | 4.99E-13 | UP |
| ENSSSCG00000000766 | CECR2 | 1.195365956 | 5.37E-13 | UP |
| ENSSSCG00000040847 | AP1S3 | 1.552371636 | 5.63E-13 | UP |
| ENSSSCG00000001764 | SH2D7 | 2.840712175 | 6.04E-13 | UP |
| ENSSSCG00000031321 | NR4A1 | 1.366710068 | 1.29E-12 | UP |
| ENSSSCG00000039950 | RMI1 | 1.024954993 | 1.57E-12 | UP |
| ENSSSCG00000015584 | PROX1 | 2.545527373 | 2.05E-12 | UP |
| ENSSSCG00000003763 | IFI44 | 1.118611986 | 2.22E-12 | UP |
| ENSSSCG00000005163 | IFNB1 | 8.679871773 | 2.34E-12 | UP |
| ENSSSCG00000009169 | SLC39A8 | 1.761624227 | 2.47E-12 | UP |
| ENSSSCG00000016573 | IRF5 | 2.174927817 | 3.19E-12 | UP |
| ENSSSCG00000038460 | FOXF1 | 1.128908649 | 3.8E-12 | UP |
| ENSSSCG00000003192 | IL4I1 | 1.085332858 | 4.09E-12 | UP |
| ENSSSCG00000010581 | PSD | 2.112835567 | 4.26E-12 | UP |
| ENSSSCG00000006309 | CD247 | 1.178680296 | 4.29E-12 | UP |
| ENSSSCG00000022064 | MLNR | 1.673514082 | 4.32E-12 | UP |
| ENSSSCG00000005268 | RORB | 1.416815877 | 5E-12 | UP |
| ENSSSCG00000013664 | C19orf66 | 1.022339071 | 5.12E-12 | UP |
| ENSSSCG00000036125 | NA | 3.272394592 | 5.66E-12 | UP |
| ENSSSCG00000016918 | MAP3K1 | 1.075363189 | 5.67E-12 | UP |
| ENSSSCG00000029675 | MMP8 | 1.775221931 | 5.78E-12 | UP |
| ENSSSCG00000006199 | PREX2 | 2.684660575 | 7.59E-12 | UP |
| ENSSSCG00000016255 | DAW1 | 3.007654797 | 8.7E-12 | UP |
| ENSSSCG00000015556 | LAMC2 | 1.185392438 | 8.7E-12 | UP |
| ENSSSCG00000035598 | EDN1 | 3.105283626 | 8.74E-12 | UP |
| ENSSSCG00000011286 | KLHL40 | 3.957426818 | 9.54E-12 | UP |
| ENSSSCG00000038677 | GJB3 | 2.44579907 | 1.1E-11 | UP |
| ENSSSCG00000016497 | DENND2A | 1.78426814 | 1.3E-11 | UP |
| ENSSSCG00000014672 | NA | 1.918078561 | 1.56E-11 | UP |
| ENSSSCG00000017466 | CCR7 | 4.026050268 | 2.16E-11 | UP |
| ENSSSCG00000007486 | CYP24A1 | 1.460545413 | 3.88E-11 | UP |
| ENSSSCG00000008159 | IL1RL1 | 1.583607005 | 4.22E-11 | UP |
| ENSSSCG00000012132 | ASB9 | 4.25148519 | 4.5E-11 | UP |
| ENSSSCG00000006186 | TRPA1 | 2.052290517 | 4.62E-11 | UP |
| ENSSSCG00000021906 | SYT7 | 3.092074981 | 1.07E-10 | UP |
| ENSSSCG00000028711 | CASP1 | 4.146107605 | 1.23E-10 | UP |
| ENSSSCG00000016018 | FRZB | 1.39945718 | 1.25E-10 | UP |
| ENSSSCG00000002821 | CCL22 | 6.910426454 | 1.58E-10 | UP |
| ENSSSCG00000034702 | NA | 1.388586279 | 1.87E-10 | UP |
| ENSSSCG00000014034 | N4BP3 | 1.370895852 | 2.08E-10 | UP |
| ENSSSCG00000012276 | SYN1 | 1.608534831 | 2.96E-10 | UP |
| ENSSSCG00000022089 | ADGRF4 | 7.777552274 | 4.54E-10 | UP |
| ENSSSCG00000016022 | ZNF804A | 2.929929031 | 6.58E-10 | UP |
| ENSSSCG00000015303 | CFAP69 | 1.036985553 | 6.94E-10 | UP |
| ENSSSCG00000035297 | ISG12(A) | 1.64259572 | 7.29E-10 | UP |
| ENSSSCG00000004479 | FILIP1 | 3.055954923 | 1.13E-09 | UP |
| ENSSSCG00000029037 | DRD1 | 5.518440716 | 1.36E-09 | UP |
| ENSSSCG00000009621 | PHYHIP | 2.555687377 | 3.7E-09 | UP |
| ENSSSCG00000013425 | MISP | 6.382907006 | 3.73E-09 | UP |
| ENSSSCG00000011899 | CD80 | 1.91288812 | 4.5E-09 | UP |
| ENSSSCG00000012104 | NA | 2.017728651 | 4.8E-09 | UP |
| ENSSSCG00000009921 | OASL | 3.788281429 | 5.62E-09 | UP |
| ENSSSCG00000000749 | SLC6A12 | 4.691927735 | 5.65E-09 | UP |
| ENSSSCG00000011391 | CDHR4 | 2.313183701 | 1.13E-08 | UP |
| ENSSSCG00000030767 | NA | 1.363203941 | 1.45E-08 | UP |
| ENSSSCG00000007499 | TFAP2C | 4.512950056 | 1.55E-08 | UP |
| ENSSSCG00000030228 | PIWIL2 | 4.248945355 | 1.58E-08 | UP |
| ENSSSCG00000001064 | GMPR | 1.1691111 | 1.69E-08 | UP |
| ENSSSCG00000007659 | ZCWPW1 | 1.184704314 | 1.69E-08 | UP |
| ENSSSCG00000009100 | TNIP3 | 1.415370195 | 1.75E-08 | UP |
| ENSSSCG00000001252 | NA | 7.177507979 | 1.93E-08 | UP |
| ENSSSCG00000025969 | PTPRR | 2.717620406 | 2.17E-08 | UP |
| ENSSSCG00000012839 | NA | 6.951981501 | 2.46E-08 | UP |
| ENSSSCG00000022083 | NA | 3.037048211 | 2.56E-08 | UP |
| ENSSSCG00000038055 | CORO1A | 1.531797751 | 2.79E-08 | UP |
| ENSSSCG00000015839 | NA | 1.217635272 | 2.79E-08 | UP |
| ENSSSCG00000026729 | TMEM150C | 1.60695033 | 2.99E-08 | UP |
| ENSSSCG00000017262 | SLC16A6 | 4.174228851 | 3.2E-08 | UP |
| ENSSSCG00000000623 | BCL2L14 | 5.116978993 | 3.51E-08 | UP |
| ENSSSCG00000001231 | NA | 1.495459836 | 3.87E-08 | UP |
| ENSSSCG00000008978 | CXCL11 | 7.054423352 | 4.15E-08 | UP |
| ENSSSCG00000028637 | CYP27B1 | 1.445305922 | 4.51E-08 | UP |
| ENSSSCG00000007899 | NA | 7.013944049 | 5.26E-08 | UP |
| ENSSSCG00000030681 | MYBPH | 3.258433777 | 7.02E-08 | UP |
| ENSSSCG00000014198 | NA | 6.739554072 | 7.57E-08 | UP |
| ENSSSCG00000014399 | ARHGAP26 | 1.227986094 | 9.5E-08 | UP |
| ENSSSCG00000018056 | SRCIN1 | 1.701084147 | 1.05E-07 | UP |
| ENSSSCG00000016030 | ZSWIM2 | 6.734448304 | 1.14E-07 | UP |
| ENSSSCG00000009997 | OSM | 6.865965541 | 1.2E-07 | UP |
| ENSSSCG00000040839 | NA | 1.546828176 | 1.23E-07 | UP |
| ENSSSCG00000003102 | PNMA8A | 1.895631963 | 1.44E-07 | UP |
| ENSSSCG00000028816 | NA | 7.043965848 | 1.57E-07 | UP |
| ENSSSCG00000005967 | FAM84B | 2.102195638 | 2.68E-07 | UP |
| ENSSSCG00000036865 | NA | 2.411754373 | 3.35E-07 | UP |
| ENSSSCG00000026318 | NETO2 | 1.018232182 | 4.12E-07 | UP |
| ENSSSCG00000012408 | NHSL2 | 1.341397584 | 4.84E-07 | UP |
| ENSSSCG00000037579 | PPBP | 1.279765245 | 5.61E-07 | UP |
| ENSSSCG00000015810 | HTRA4 | 1.148675636 | 5.66E-07 | UP |
| ENSSSCG00000004890 | SERPINB2 | 6.370519843 | 5.85E-07 | UP |
| ENSSSCG00000039300 | IL27 | 5.759559373 | 6.96E-07 | UP |
| ENSSSCG00000031255 | NA | 1.080505447 | 7.09E-07 | UP |
| ENSSSCG00000007964 | NA | 6.469679377 | 7.49E-07 | UP |
| ENSSSCG00000007490 | CBLN4 | 1.019653059 | 1.09E-06 | UP |
| ENSSSCG00000001036 | TFAP2A | 1.352437244 | 1.15E-06 | UP |
| ENSSSCG00000006172 | PI15 | 2.898122265 | 1.26E-06 | UP |
| ENSSSCG00000008124 | NEURL3 | 6.528219635 | 1.3E-06 | UP |
| ENSSSCG00000035240 | GPR63 | 1.370770979 | 1.56E-06 | UP |
| ENSSSCG00000005511 | TRAF1 | 1.067089774 | 1.6E-06 | UP |
| ENSSSCG00000032558 | EMX2 | 1.443967271 | 1.65E-06 | UP |
| ENSSSCG00000009132 | ENPEP | 2.004156017 | 1.86E-06 | UP |
| ENSSSCG00000033843 | ETFRF1 | 1.098541529 | 1.93E-06 | UP |
| ENSSSCG00000015379 | NA | 2.594221546 | 2.28E-06 | UP |
| ENSSSCG00000031646 | APOC4 | 6.292120152 | 2.69E-06 | UP |
| ENSSSCG00000009219 | IBSP | 6.499859717 | 2.84E-06 | UP |
| ENSSSCG00000038080 | EMCN | 1.710552834 | 2.86E-06 | UP |
| ENSSSCG00000014897 | FAM181B | 4.54988369 | 2.87E-06 | UP |
| ENSSSCG00000040830 | NA | 2.3457684 | 2.91E-06 | UP |
| ENSSSCG00000014988 | MMP13 | 2.479719309 | 3.14E-06 | UP |
| ENSSSCG00000036742 | KLF15 | 1.187783875 | 3.24E-06 | UP |
| ENSSSCG00000002002 | IRF9 | 2.160517263 | 3.48E-06 | UP |
| ENSSSCG00000024344 | CCR5 | 5.335186725 | 3.64E-06 | UP |
| ENSSSCG00000031868 | XK | 3.858470078 | 4.05E-06 | UP |
| ENSSSCG00000035016 | NA | 3.836870905 | 4.77E-06 | UP |
| ENSSSCG00000010755 | PTPRE | 1.545382387 | 5.27E-06 | UP |
| ENSSSCG00000035500 | ZEB2_AS1_3 | 2.356243577 | 5.67E-06 | UP |
| ENSSSCG00000039828 | NA | 1.235485319 | 6.73E-06 | UP |
| ENSSSCG00000026943 | MRAP2 | 1.866386941 | 9.79E-06 | UP |
| ENSSSCG00000037949 | NA | 5.927460124 | 1.03E-05 | UP |
| ENSSSCG00000002504 | AK7 | 2.824949905 | 1.04E-05 | UP |
| ENSSSCG00000039214 | NA | 5.810402069 | 1.14E-05 | UP |
| ENSSSCG00000040557 | SERTM1 | 2.965425721 | 1.18E-05 | UP |
| ENSSSCG00000016832 | IL7R | 6.157460766 | 1.38E-05 | UP |
| ENSSSCG00000003737 | NOL4 | 2.202756188 | 1.56E-05 | UP |
| ENSSSCG00000016903 | GZMA | 5.737614539 | 1.6E-05 | UP |
| ENSSSCG00000022361 | NA | 1.245959442 | 1.72E-05 | UP |
| ENSSSCG00000039341 | NA | 6.031030913 | 2.07E-05 | UP |
| ENSSSCG00000032221 | FAM110C | 1.110251453 | 2.07E-05 | UP |
| ENSSSCG00000032826 | KCTD16 | 1.32859324 | 2.25E-05 | UP |
| ENSSSCG00000017474 | GJD3 | 4.249944688 | 2.35E-05 | UP |
| ENSSSCG00000022849 | IL2RA | 3.430905023 | 2.53E-05 | UP |
| ENSSSCG00000027967 | IGSF6 | 4.307423188 | 2.64E-05 | UP |
| ENSSSCG00000037278 | NA | 1.043183396 | 2.87E-05 | UP |
| ENSSSCG00000021997 | ALS2CL | 1.397372021 | 2.94E-05 | UP |
| ENSSSCG00000006398 | SLAMF8 | 2.062159889 | 3.2E-05 | UP |
| ENSSSCG00000005098 | SYT16 | 1.623281359 | 3.75E-05 | UP |
| ENSSSCG00000010445 | ANKRD22 | 5.475034082 | 3.97E-05 | UP |
| ENSSSCG00000034691 | ZEB2_AS1_1 | 1.526313385 | 4.26E-05 | UP |
| ENSSSCG00000001042 | MAK | 1.157090792 | 4.53E-05 | UP |
| ENSSSCG00000023192 | NA | 2.340792243 | 4.66E-05 | UP |
| ENSSSCG00000004791 | RASGRP1 | 5.407068272 | 4.77E-05 | UP |
| ENSSSCG00000006477 | BCAN | 4.083275205 | 5.02E-05 | UP |
| ENSSSCG00000022512 | TRDC | 5.771166658 | 5.23E-05 | UP |
| ENSSSCG00000036364 | EGR4 | 1.578018592 | 5.27E-05 | UP |
| ENSSSCG00000005439 | ACTL7A | 5.468731203 | 5.34E-05 | UP |
| ENSSSCG00000024867 | ISG20 | 4.127078547 | 5.73E-05 | UP |
| ENSSSCG00000001405 | LTB | 1.223060699 | 5.84E-05 | UP |
| ENSSSCG00000006286 | NA | 2.837635778 | 5.85E-05 | UP |
| ENSSSCG00000008347 | FBXO48 | 1.022493909 | 5.89E-05 | UP |
| ENSSSCG00000036157 | BARX2 | 5.252963539 | 6.19E-05 | UP |
| ENSSSCG00000022618 | CMTM8 | 1.725905702 | 6.41E-05 | UP |
| ENSSSCG00000040720 | SLC26A9 | 4.659665925 | 7.11E-05 | UP |
| ENSSSCG00000001916 | C15orf59 | 1.214841585 | 7.45E-05 | UP |
| ENSSSCG00000007607 | KPNA7 | 2.852576686 | 7.74E-05 | UP |
| ENSSSCG00000009748 | RIMBP2 | 3.466334774 | 8.08E-05 | UP |
| ENSSSCG00000004540 | ONECUT2 | 1.374095474 | 8.62E-05 | UP |
| ENSSSCG00000004236 | PKIB | 1.484459209 | 0.000102 | UP |
| ENSSSCG00000014099 | OTP | 5.586108696 | 0.000103 | UP |
| ENSSSCG00000025410 | PRSS16 | 3.845730694 | 0.000105 | UP |
| ENSSSCG00000015362 | TSPAN13 | 1.089495859 | 0.000106 | UP |
| ENSSSCG00000001483 | FAM83B | 5.444157188 | 0.000119 | UP |
| ENSSSCG00000003801 | IL23R | 5.191622734 | 0.000125 | UP |
| ENSSSCG00000033286 | NA | 3.635598978 | 0.000149 | UP |
| ENSSSCG00000032561 | PDCD1LG2 | 3.234292422 | 0.000161 | UP |
| ENSSSCG00000006161 | IL7 | 1.897262371 | 0.00017 | UP |
| ENSSSCG00000003006 | CYP2B6 | 2.959959743 | 0.00018 | UP |
| ENSSSCG00000011736 | SLITRK3 | 3.377067515 | 0.000248 | UP |
| ENSSSCG00000006802 | NA | 1.289978706 | 0.000254 | UP |
| ENSSSCG00000015433 | NA | 2.664862365 | 0.000257 | UP |
| ENSSSCG00000023591 | ADGRF2 | 2.701609508 | 0.000282 | UP |
| ENSSSCG00000026592 | TLR6 | 1.007379232 | 0.000297 | UP |
| ENSSSCG00000029284 | NPHS1 | 3.303207956 | 0.000328 | UP |
| ENSSSCG00000000649 | CLEC1A | 2.88151739 | 0.000337 | UP |
| ENSSSCG00000006196 | NA | 1.722621557 | 0.000337 | UP |
| ENSSSCG00000039779 | CHGB | 1.056188294 | 0.000342 | UP |
| ENSSSCG00000029754 | SLC39A2 | 1.966143723 | 0.000343 | UP |
| ENSSSCG00000000647 | OLR1 | 5.224551236 | 0.000353 | UP |
| ENSSSCG00000006383 | VANGL2 | 1.240635547 | 0.000358 | UP |
| ENSSSCG00000022584 | PPP1R3F | 1.066157337 | 0.000362 | UP |
| ENSSSCG00000034633 | CFAP99 | 2.00501454 | 0.00037 | UP |
| ENSSSCG00000032082 | NA | 5.097390154 | 0.000375 | UP |
| ENSSSCG00000030675 | MIXL1 | 1.940689069 | 0.000438 | UP |
| ENSSSCG00000034708 | PITX2 | 3.177345297 | 0.00047 | UP |
| ENSSSCG00000011596 | TRH | 1.266828704 | 0.000525 | UP |
| ENSSSCG00000023684 | MT1A | 2.839924822 | 0.000563 | UP |
| ENSSSCG00000005446 | EPB41L4B | 1.01773971 | 0.000604 | UP |
| ENSSSCG00000036785 | NA | 1.330398217 | 0.000634 | UP |
| ENSSSCG00000033878 | RAB38 | 3.128099226 | 0.000668 | UP |
| ENSSSCG00000033385 | NA | 1.120037827 | 0.00075 | UP |
| ENSSSCG00000016256 | SPHKAP | 1.843988744 | 0.000754 | UP |
| ENSSSCG00000037247 | NA | 3.976276436 | 0.000851 | UP |
| ENSSSCG00000037541 | NA | 4.78524335 | 0.000863 | UP |
| ENSSSCG00000036768 | PRAG1 | 1.033021848 | 0.000877 | UP |
| ENSSSCG00000021573 | KCNJ5 | 1.582439138 | 0.000895 | UP |
| ENSSSCG00000032686 | RUNX3 | 1.552484773 | 0.000941 | UP |
| ENSSSCG00000016284 | NA | 4.874470822 | 0.000982 | UP |
| ENSSSCG00000036932 | WNT6 | 1.238738477 | 0.000983 | UP |
| ENSSSCG00000029096 | HRH2 | 2.116551619 | 0.001049 | UP |
| ENSSSCG00000038420 | PERP | 1.131420724 | 0.001073 | UP |
| ENSSSCG00000017158 | CCDC40 | 1.000895608 | 0.001088 | UP |
| ENSSSCG00000035732 | NA | 4.736658368 | 0.001147 | UP |
| ENSSSCG00000009410 | RUBCNL | 1.26949454 | 0.001354 | UP |
| ENSSSCG00000004151 | NA | 1.134196992 | 0.00141 | UP |
| ENSSSCG00000006140 | CA2 | 2.952680706 | 0.001449 | UP |
| ENSSSCG00000021515 | HS3ST1 | 4.435249994 | 0.001451 | UP |
| ENSSSCG00000033637 | NA | 2.966998044 | 0.001506 | UP |
| ENSSSCG00000032405 | TMEM190 | 1.544848157 | 0.001588 | UP |
| ENSSSCG00000021596 | KCNA5 | 1.880625357 | 0.001598 | UP |
| ENSSSCG00000010504 | BLNK | 1.75400848 | 0.001739 | UP |
| ENSSSCG00000017720 | CCL1 | 4.5367355 | 0.001772 | UP |
| ENSSSCG00000016059 | STAT4 | 1.76209049 | 0.001778 | UP |
| ENSSSCG00000004831 | NA | 1.579337011 | 0.001949 | UP |
| ENSSSCG00000032980 | NA | 1.405718169 | 0.002021 | UP |
| ENSSSCG00000016286 | PRSS56 | 1.392789699 | 0.002031 | UP |
| ENSSSCG00000033736 | NA | 1.354012636 | 0.002059 | UP |
| ENSSSCG00000036011 | ISLR2 | 1.477302967 | 0.002064 | UP |
| ENSSSCG00000011730 | IL12A | 2.873751305 | 0.002079 | UP |
| ENSSSCG00000025500 | HTR1D | 3.745226932 | 0.002088 | UP |
| ENSSSCG00000009045 | HHIP | 1.35201167 | 0.002102 | UP |
| ENSSSCG00000006542 | KCNN3 | 2.609351039 | 0.002341 | UP |
| ENSSSCG00000015979 | HOXD13 | 1.335147903 | 0.002394 | UP |
| ENSSSCG00000033721 | NA | 1.379670875 | 0.002412 | UP |
| ENSSSCG00000035521 | KLHL38 | 4.131510322 | 0.002445 | UP |
| ENSSSCG00000017995 | USP43 | 3.608852732 | 0.002522 | UP |
| ENSSSCG00000002474 | PPP4R4 | 1.736200063 | 0.002566 | UP |
| ENSSSCG00000040412 | NA | 1.52504961 | 0.002669 | UP |
| ENSSSCG00000006981 | MICU3 | 1.433183515 | 0.003156 | UP |
| ENSSSCG00000006289 | F5 | 1.483242135 | 0.003247 | UP |
| ENSSSCG00000014996 | NA | 3.529407834 | 0.004049 | UP |
| ENSSSCG00000035955 | NA | 1.303946017 | 0.004166 | UP |
| ENSSSCG00000016567 | STRIP2 | 1.431047528 | 0.004196 | UP |
| ENSSSCG00000023785 | TMEM156 | 3.759661489 | 0.00423 | UP |
| ENSSSCG00000033507 | ASCL2 | 4.348809423 | 0.004262 | UP |
| ENSSSCG00000006013 | AARD | 3.21195067 | 0.004552 | UP |
| ENSSSCG00000026345 | NA | 1.494540841 | 0.004735 | UP |
| ENSSSCG00000037202 | CACNG4 | 1.498785405 | 0.005051 | UP |
| ENSSSCG00000027405 | U2 | 1.770904317 | 0.005928 | UP |
| ENSSSCG00000011393 | UBA7 | 3.196861519 | 0.006062 | UP |
| ENSSSCG00000005222 | SLC1A1 | 1.803711932 | 0.006137 | UP |
| ENSSSCG00000033768 | LAX1 | 2.743880149 | 0.007265 | UP |
| ENSSSCG00000031649 | RNASE1 | 2.161069252 | 0.007285 | UP |
| ENSSSCG00000006418 | NA | 3.450767838 | 0.008462 | UP |
| ENSSSCG00000006693 | PDZK1 | 1.638645244 | 0.008637 | UP |
| ENSSSCG00000015953 | DLX1 | 1.712568558 | 0.008702 | UP |
| ENSSSCG00000026958 | WNT10A | 3.689913717 | 0.008889 | UP |
| ENSSSCG00000040728 | EYA1 | 1.5067827 | 0.008922 | UP |
| ENSSSCG00000006455 | NA | 4.079115727 | 0.008968 | UP |
| ENSSSCG00000018173 | ssc-mir-425 | 2.947438704 | 0.009287 | UP |
| ENSSSCG00000015559 | NCF2 | 1.61984317 | 0.009701 | UP |
| ENSSSCG00000038903 | NA | 2.068945144 | 0.009709 | UP |
| ENSSSCG00000016247 | TM4SF20 | 2.477142378 | 0.010414 | UP |
| ENSSSCG00000025535 | TFAP2B | 1.867266342 | 0.010981 | UP |
| ENSSSCG00000009859 | NA | 1.59311666 | 0.012001 | UP |
| ENSSSCG00000031788 | PTAFR | 3.70392757 | 0.012363 | UP |
| ENSSSCG00000009445 | PCDH8 | 1.688051012 | 0.012499 | UP |
| ENSSSCG00000000657 | NA | 2.225074484 | 0.01292 | UP |
| ENSSSCG00000010337 | MAT1A | 1.631296815 | 0.012966 | UP |
| ENSSSCG00000009347 | NA | 3.269611409 | 0.013182 | UP |
| ENSSSCG00000037063 | PROKR2 | 1.122448661 | 0.013636 | UP |
| ENSSSCG00000001786 | CFAP161 | 3.309570987 | 0.013721 | UP |
| ENSSSCG00000001500 | NA | 1.342770389 | 0.013919 | UP |
| ENSSSCG00000038622 | NA | 2.620001926 | 0.01463 | UP |
| ENSSSCG00000017411 | KCNH4 | 1.051082124 | 0.015363 | UP |
| ENSSSCG00000017861 | ASPA | 1.89409665 | 0.015797 | UP |
| ENSSSCG00000023791 | TMEM229B | 1.956203849 | 0.016809 | UP |
| ENSSSCG00000022968 | ARFGEF3 | 2.713879787 | 0.016883 | UP |
| ENSSSCG00000000718 | GALNT8 | 3.119215796 | 0.01697 | UP |
| ENSSSCG00000026349 | ALDH3B2 | 2.051536623 | 0.016993 | UP |
| ENSSSCG00000006256 | SOX17 | 2.751880726 | 0.017891 | UP |
| ENSSSCG00000004039 | SLC22A3 | 1.712025207 | 0.018126 | UP |
| ENSSSCG00000029419 | SCN11A | 2.297557002 | 0.018633 | UP |
| ENSSSCG00000020953 | NA | 2.132556252 | 0.020435 | UP |
| ENSSSCG00000038351 | NA | 1.36186857 | 0.022212 | UP |
| ENSSSCG00000040973 | HYDIN | 1.364289384 | 0.022275 | UP |
| ENSSSCG00000040903 | NA | 3.026142181 | 0.022445 | UP |
| ENSSSCG00000009520 | ZIC2 | 2.214849008 | 0.023152 | UP |
| ENSSSCG00000003386 | TNFRSF9 | 1.942014096 | 0.023901 | UP |
| ENSSSCG00000019224 | SNORD93 | 1.53341488 | 0.024669 | UP |
| ENSSSCG00000006932 | NA | 3.907388294 | 0.02514 | UP |
| ENSSSCG00000003161 | PRRG2 | 1.072734612 | 0.02645 | UP |
| ENSSSCG00000031874 | REM2 | 1.74061962 | 0.029448 | UP |
| ENSSSCG00000000500 | RAB3IP | 1.150412058 | 0.032608 | UP |
| ENSSSCG00000007482 | NA | 1.910670919 | 0.032734 | UP |
| ENSSSCG00000034788 | NA | 1.508997234 | 0.033189 | UP |
| ENSSSCG00000008802 | BEND4 | 1.895417611 | 0.034076 | UP |
| ENSSSCG00000012456 | RPS6KA6 | 1.722191326 | 0.034585 | UP |
| ENSSSCG00000039959 | NA | 1.869716649 | 0.034816 | UP |
| ENSSSCG00000010650 | AFAP1L2 | 1.142956599 | 0.035755 | UP |
| ENSSSCG00000008213 | CD8B | 2.516460037 | 0.035842 | UP |
| ENSSSCG00000004781 | NA | 1.145428516 | 0.037757 | UP |
| ENSSSCG00000009648 | NEFM | 1.552929577 | 0.038752 | UP |
| ENSSSCG00000037470 | SNORA70 | 1.407619003 | 0.040315 | UP |
| ENSSSCG00000026886 | NA | 1.528777731 | 0.046109 | UP |
| ENSSSCG00000022060 | RASSF10 | 1.330616145 | 0.047917 | UP |
| ENSSSCG00000009051 | IL15 | 1.914744663 | 0.048014 | UP |
| ENSSSCG00000018605 | SNORA20 | 1.395893619 | 0.048868 | UP |
| ENSSSCG00000037177 | C2CD4C | 1.887551466 | 0.049198 | UP |
| ENSSSCG00000016857 | DAB2 | -2.360283108 | 0 | DOWN |
| ENSSSCG00000027157 | SLC40A1 | -3.098618626 | 0 | DOWN |
| ENSSSCG00000037066 | GADD45A | -3.470544142 | 0 | DOWN |
| ENSSSCG00000032527 | FOSL2 | -1.905739241 | 1.6E-301 | DOWN |
| ENSSSCG00000032749 | PCDH18 | -2.65562527 | 1.2E-276 | DOWN |
| ENSSSCG00000009111 | SYNPO2 | -2.509037802 | 4.3E-269 | DOWN |
| ENSSSCG00000007073 | ISM1 | -1.909807632 | 2.7E-238 | DOWN |
| ENSSSCG00000033001 | FZD8 | -4.830395168 | 9E-231 | DOWN |
| ENSSSCG00000036679 | SORBS2 | -3.085186663 | 6.3E-215 | DOWN |
| ENSSSCG00000034491 | PRICKLE1 | -1.211505518 | 2.8E-196 | DOWN |
| ENSSSCG00000037900 | CCND1 | -1.537155795 | 9.1E-192 | DOWN |
| ENSSSCG00000003715 | NA | -1.842757705 | 1.1E-178 | DOWN |
| ENSSSCG00000007356 | PLCG1 | -1.802993094 | 3.7E-176 | DOWN |
| ENSSSCG00000031201 | LMOD1 | -2.326169545 | 8.1E-173 | DOWN |
| ENSSSCG00000003644 | FHL3 | -1.49483716 | 2.1E-171 | DOWN |
| ENSSSCG00000004192 | CTGF | -1.752464685 | 2.8E-170 | DOWN |
| ENSSSCG00000014242 | ZNF608 | -1.811404963 | 2.9E-170 | DOWN |
| ENSSSCG00000009638 | RHOBTB2 | -1.70580406 | 7.8E-170 | DOWN |
| ENSSSCG00000029260 | NDNF | -2.145682093 | 2.7E-169 | DOWN |
| ENSSSCG00000039780 | RTN4RL1 | -2.353876111 | 9.1E-169 | DOWN |
| ENSSSCG00000001620 | MDFI | -3.16960126 | 8.6E-167 | DOWN |
| ENSSSCG00000011463 | IL17RD | -3.056661679 | 2.6E-157 | DOWN |
| ENSSSCG00000026710 | CARHSP1 | -1.340090925 | 1.9E-148 | DOWN |
| ENSSSCG00000016111 | FZD7 | -2.271138914 | 3.5E-146 | DOWN |
| ENSSSCG00000038838 | DLX5 | -3.430327007 | 8E-137 | DOWN |
| ENSSSCG00000029998 | KLF7 | -1.482525747 | 1.3E-136 | DOWN |
| ENSSSCG00000014822 | ARHGEF17 | -1.766320563 | 6.8E-134 | DOWN |
| ENSSSCG00000009567 | RASA3 | -1.874643523 | 1.9E-128 | DOWN |
| ENSSSCG00000038149 | KCNE4 | -3.312492395 | 1.6E-127 | DOWN |
| ENSSSCG00000000910 | CRADD | -1.837722746 | 5.9E-127 | DOWN |
| ENSSSCG00000007727 | AUTS2 | -1.510988223 | 4.7E-122 | DOWN |
| ENSSSCG00000007476 | KCNG1 | -2.284088488 | 3.3E-121 | DOWN |
| ENSSSCG00000014909 | NA | -2.485807009 | 6.1E-121 | DOWN |
| ENSSSCG00000005308 | RUSC2 | -1.246680775 | 8.1E-120 | DOWN |
| ENSSSCG00000017046 | EBF1 | -1.788953379 | 1.3E-119 | DOWN |
| ENSSSCG00000038220 | RXRA | -1.732853626 | 1.3E-117 | DOWN |
| ENSSSCG00000006889 | ARHGAP29 | -1.858379031 | 1.6E-116 | DOWN |
| ENSSSCG00000010017 | SMTN | -1.112893113 | 1.7E-116 | DOWN |
| ENSSSCG00000035987 | EHD3 | -2.027387105 | 1.8E-115 | DOWN |
| ENSSSCG00000028529 | REM1 | -2.167151788 | 2.4E-115 | DOWN |
| ENSSSCG00000026516 | EPHB3 | -2.265335549 | 1.3E-114 | DOWN |
| ENSSSCG00000014149 | MEF2C | -1.977110748 | 6.2E-114 | DOWN |
| ENSSSCG00000033509 | SAMD11 | -3.022763267 | 6.5E-114 | DOWN |
| ENSSSCG00000022289 | PCDH1 | -3.687122871 | 4.9E-112 | DOWN |
| ENSSSCG00000030680 | TCF7 | -1.256258345 | 5.2E-112 | DOWN |
| ENSSSCG00000006273 | MCM4 | -1.300897399 | 5.6E-112 | DOWN |
| ENSSSCG00000023653 | GLIS2 | -1.229921027 | 8.4E-111 | DOWN |
| ENSSSCG00000008230 | ATOH8 | -3.804282634 | 1.6E-108 | DOWN |
| ENSSSCG00000013400 | MICAL2 | -1.1557683 | 7.8E-108 | DOWN |
| ENSSSCG00000008468 | PKDCC | -3.056005966 | 3.4E-107 | DOWN |
| ENSSSCG00000035952 | TGFB1I1 | -1.330548283 | 1.2E-104 | DOWN |
| ENSSSCG00000000455 | LRIG3 | -2.204168221 | 6.4E-104 | DOWN |
| ENSSSCG00000007477 | NFATC2 | -1.464598519 | 5.7E-101 | DOWN |
| ENSSSCG00000027467 | DZIP1L | -1.429429221 | 2.6E-99 | DOWN |
| ENSSSCG00000004948 | SMAD6 | -1.957001731 | 8.2E-97 | DOWN |
| ENSSSCG00000015880 | TANC1 | -1.623125464 | 1.09E-95 | DOWN |
| ENSSSCG00000038492 | FAM109B | -1.435940913 | 6.47E-95 | DOWN |
| ENSSSCG00000026116 | FHOD1 | -1.57635843 | 1.86E-94 | DOWN |
| ENSSSCG00000003376 | PLEKHG5 | -1.396210483 | 3.66E-92 | DOWN |
| ENSSSCG00000008422 | NA | -1.278404139 | 3.15E-89 | DOWN |
| ENSSSCG00000016453 | TCAF1 | -1.044033937 | 1.45E-86 | DOWN |
| ENSSSCG00000027669 | NA | -1.05043051 | 2.66E-85 | DOWN |
| ENSSSCG00000011074 | ARHGAP21 | -1.228741643 | 1.19E-84 | DOWN |
| ENSSSCG00000010600 | CALHM2 | -1.765420659 | 1.61E-82 | DOWN |
| ENSSSCG00000032170 | DAPK3 | -1.075097106 | 2.04E-81 | DOWN |
| ENSSSCG00000009357 | SMAD9 | -1.420467482 | 3E-81 | DOWN |
| ENSSSCG00000003486 | ARHGEF10L | -1.457436068 | 3.95E-81 | DOWN |
| ENSSSCG00000006874 | PALMD | -1.771157587 | 5.48E-79 | DOWN |
| ENSSSCG00000029662 | RASSF4 | -1.035358455 | 8.33E-79 | DOWN |
| ENSSSCG00000040037 | MTSS1L | -1.534794346 | 1.81E-78 | DOWN |
| ENSSSCG00000011430 | DUSP7 | -1.15247005 | 1.08E-77 | DOWN |
| ENSSSCG00000010209 | FAM13C | -3.36254516 | 1.1E-77 | DOWN |
| ENSSSCG00000010698 | FGFR2 | -1.461647234 | 1.36E-77 | DOWN |
| ENSSSCG00000016703 | HOXA5 | -1.497664068 | 1.48E-76 | DOWN |
| ENSSSCG00000030827 | FGFR3 | -1.221486035 | 3.8E-76 | DOWN |
| ENSSSCG00000021440 | GPSM2 | -1.738870378 | 6.57E-76 | DOWN |
| ENSSSCG00000028606 | ZBTB7B | -1.098516213 | 6.87E-76 | DOWN |
| ENSSSCG00000040334 | CBX6 | -1.192115803 | 8.79E-76 | DOWN |
| ENSSSCG00000039194 | KANK2 | -1.037592889 | 2.96E-74 | DOWN |
| ENSSSCG00000016841 | SLC1A3 | -2.398314819 | 5.22E-74 | DOWN |
| ENSSSCG00000005773 | RBFA | -1.904114556 | 1.15E-73 | DOWN |
| ENSSSCG00000000160 | PRDM4 | -1.429915972 | 5.05E-73 | DOWN |
| ENSSSCG00000012591 | AMOT | -1.109268754 | 5.44E-73 | DOWN |
| ENSSSCG00000009122 | ARSJ | -1.274214064 | 6.04E-73 | DOWN |
| ENSSSCG00000035895 | JDP2 | -1.586699917 | 8.43E-73 | DOWN |
| ENSSSCG00000009468 | KCTD12 | -1.70195599 | 1.15E-72 | DOWN |
| ENSSSCG00000023525 | TMEM26 | -1.840626312 | 1.61E-71 | DOWN |
| ENSSSCG00000017144 | NPTX1 | -1.501590498 | 9.73E-71 | DOWN |
| ENSSSCG00000013351 | NAV2 | -1.455123943 | 2.19E-69 | DOWN |
| ENSSSCG00000030005 | LGALSL | -1.299249001 | 3.65E-69 | DOWN |
| ENSSSCG00000034551 | SCX | -1.422517543 | 5.86E-69 | DOWN |
| ENSSSCG00000032473 | NYNRIN | -1.478190371 | 6.1E-69 | DOWN |
| ENSSSCG00000007675 | EPHB4 | -1.553922034 | 6.56E-69 | DOWN |
| ENSSSCG00000004974 | LARP6 | -1.428081075 | 4.39E-68 | DOWN |
| ENSSSCG00000036436 | FZD1 | -1.143324697 | 1.09E-67 | DOWN |
| ENSSSCG00000003549 | LDLRAP1 | -1.048497949 | 3.04E-67 | DOWN |
| ENSSSCG00000032632 | CMTM4 | -1.678918452 | 2.25E-66 | DOWN |
| ENSSSCG00000031329 | ST8SIA1 | -1.52185555 | 2.07E-65 | DOWN |
| ENSSSCG00000025610 | AGAP3 | -1.391662309 | 3.92E-65 | DOWN |
| ENSSSCG00000040731 | TMEM65 | -1.494411455 | 2.2E-64 | DOWN |
| ENSSSCG00000039514 | ID3 | -1.603571277 | 3.14E-64 | DOWN |
| ENSSSCG00000005361 | ALDH1B1 | -1.755845967 | 3E-63 | DOWN |
| ENSSSCG00000040732 | WDR81 | -1.004224546 | 4.72E-63 | DOWN |
| ENSSSCG00000014207 | APC | -1.144365419 | 7.3E-63 | DOWN |
| ENSSSCG00000017890 | KIAA0753 | -1.19928126 | 1.12E-62 | DOWN |
| ENSSSCG00000015336 | SLC25A13 | -1.314780355 | 1.18E-62 | DOWN |
| ENSSSCG00000040638 | DIO2 | -2.294097402 | 3.4E-62 | DOWN |
| ENSSSCG00000005378 | ANKS6 | -1.649202199 | 9.71E-62 | DOWN |
| ENSSSCG00000012950 | RIN1 | -1.468184727 | 1.28E-61 | DOWN |
| ENSSSCG00000004082 | NA | -2.002056819 | 2.78E-61 | DOWN |
| ENSSSCG00000006850 | FAM102B | -1.134248798 | 3.32E-61 | DOWN |
| ENSSSCG00000011278 | TRAK1 | -1.064518794 | 1.43E-60 | DOWN |
| ENSSSCG00000011602 | XPC | -1.034964703 | 1.58E-60 | DOWN |
| ENSSSCG00000004201 | TMEM200A | -1.53994079 | 4.83E-60 | DOWN |
| ENSSSCG00000040267 | CYS1 | -1.140607801 | 5.94E-60 | DOWN |
| ENSSSCG00000001639 | TRERF1 | -2.539459897 | 1.85E-59 | DOWN |
| ENSSSCG00000006331 | PBX1 | -1.262930156 | 8.35E-59 | DOWN |
| ENSSSCG00000006729 | FAM46C | -3.352346568 | 2.23E-58 | DOWN |
| ENSSSCG00000026989 | RAD51D | -1.067861615 | 3.52E-58 | DOWN |
| ENSSSCG00000036634 | MAP4K5 | -1.067827947 | 9.43E-58 | DOWN |
| ENSSSCG00000013049 | RCOR2 | -1.422783644 | 1.04E-57 | DOWN |
| ENSSSCG00000009071 | JADE1 | -2.396812351 | 1.64E-57 | DOWN |
| ENSSSCG00000038164 | RAI1 | -1.351418109 | 1.73E-57 | DOWN |
| ENSSSCG00000031875 | ZNF469 | -1.623262526 | 1.85E-57 | DOWN |
| ENSSSCG00000032154 | ERF | -1.01491251 | 2.08E-57 | DOWN |
| ENSSSCG00000031510 | POMK | -1.158118712 | 2.75E-57 | DOWN |
| ENSSSCG00000029201 | AJUBA | -2.715662002 | 4.46E-57 | DOWN |
| ENSSSCG00000025488 | MCM3 | -1.075558235 | 1.27E-56 | DOWN |
| ENSSSCG00000011936 | ZBED2 | -1.925068504 | 6.79E-56 | DOWN |
| ENSSSCG00000028239 | FBXL7 | -1.083256737 | 3.8E-55 | DOWN |
| ENSSSCG00000010311 | CAMK2G | -1.052516613 | 6.2E-55 | DOWN |
| ENSSSCG00000025260 | CARD10 | -1.739623446 | 3.61E-54 | DOWN |
| ENSSSCG00000007572 | LFNG | -2.692445559 | 3.63E-54 | DOWN |
| ENSSSCG00000005506 | MEGF9 | -1.477820665 | 9.36E-54 | DOWN |
| ENSSSCG00000006235 | TOX | -3.000191307 | 1.37E-53 | DOWN |
| ENSSSCG00000001573 | PIM1 | -1.591707417 | 1.55E-53 | DOWN |
| ENSSSCG00000002350 | ELMSAN1 | -1.098959116 | 1.19E-52 | DOWN |
| ENSSSCG00000006250 | LYN | -1.046012118 | 3.64E-52 | DOWN |
| ENSSSCG00000031741 | NA | -1.021258382 | 4.36E-52 | DOWN |
| ENSSSCG00000001081 | SOX4 | -1.077932271 | 4.7E-52 | DOWN |
| ENSSSCG00000017497 | ERBB2 | -1.171880307 | 6.31E-52 | DOWN |
| ENSSSCG00000007451 | SLC2A10 | -1.137993648 | 2.79E-51 | DOWN |
| ENSSSCG00000029687 | TMEM246 | -1.375247735 | 3.36E-51 | DOWN |
| ENSSSCG00000007603 | NPTX2 | -1.482906292 | 3.67E-51 | DOWN |
| ENSSSCG00000015961 | CDCA7 | -1.388463633 | 3.94E-51 | DOWN |
| ENSSSCG00000017573 | XYLT2 | -1.015030721 | 7.65E-51 | DOWN |
| ENSSSCG00000038491 | MEX3B | -1.655488209 | 9.7E-51 | DOWN |
| ENSSSCG00000039171 | MKL2 | -1.109838084 | 2.14E-50 | DOWN |
| ENSSSCG00000007664 | AGFG2 | -1.250730827 | 6.07E-50 | DOWN |
| ENSSSCG00000008446 | SIX2 | -2.015257059 | 6.48E-50 | DOWN |
| ENSSSCG00000030511 | LGR5 | -2.06724562 | 7.26E-50 | DOWN |
| ENSSSCG00000023618 | FRMD7 | -2.507512647 | 1.82E-49 | DOWN |
| ENSSSCG00000000087 | TAB1 | -1.092631239 | 3.36E-49 | DOWN |
| ENSSSCG00000008593 | KLHL29 | -1.451135503 | 4.61E-49 | DOWN |
| ENSSSCG00000016725 | NA | -1.740998753 | 2.05E-48 | DOWN |
| ENSSSCG00000029949 | CD248 | -1.009075249 | 6E-48 | DOWN |
| ENSSSCG00000017943 | ACAP1 | -1.058798944 | 1.09E-47 | DOWN |
| ENSSSCG00000000162 | BTBD11 | -3.087293988 | 4.65E-47 | DOWN |
| ENSSSCG00000001078 | MBOAT1 | -1.12416033 | 5.82E-47 | DOWN |
| ENSSSCG00000036360 | LURAP1L | -4.441129592 | 6.01E-47 | DOWN |
| ENSSSCG00000023724 | ZBTB1 | -1.006629583 | 6.81E-47 | DOWN |
| ENSSSCG00000014448 | ARSI | -2.916483107 | 7.56E-47 | DOWN |
| ENSSSCG00000039425 | BPGM | -1.182967141 | 1.22E-46 | DOWN |
| ENSSSCG00000013244 | PACSIN3 | -1.19467465 | 3.48E-46 | DOWN |
| ENSSSCG00000003949 | CDC20 | -1.029275945 | 6.11E-46 | DOWN |
| ENSSSCG00000034948 | RASSF9 | -2.212393258 | 6.46E-46 | DOWN |
| ENSSSCG00000000530 | FGD4 | -1.613714581 | 7.71E-46 | DOWN |
| ENSSSCG00000032078 | ZNF362 | -1.251339399 | 8.41E-46 | DOWN |
| ENSSSCG00000009968 | TTC28 | -1.089119297 | 9.37E-46 | DOWN |
| ENSSSCG00000031819 | TP53I11 | -1.825922319 | 9.87E-46 | DOWN |
| ENSSSCG00000036030 | FAM212B | -1.73156338 | 1.14E-45 | DOWN |
| ENSSSCG00000007463 | PTGIS | -1.20936367 | 2.34E-45 | DOWN |
| ENSSSCG00000029849 | S1PR1 | -3.327541099 | 4.07E-45 | DOWN |
| ENSSSCG00000017793 | ANKRD13B | -1.053401728 | 4.11E-45 | DOWN |
| ENSSSCG00000015396 | SEMA3D | -1.047969841 | 1.03E-44 | DOWN |
| ENSSSCG00000027869 | PHF13 | -1.216525102 | 2.72E-44 | DOWN |
| ENSSSCG00000037647 | TFDP2 | -1.350967253 | 3.12E-44 | DOWN |
| ENSSSCG00000015780 | STOX2 | -2.068893971 | 5.54E-44 | DOWN |
| ENSSSCG00000015052 | USP28 | -1.1587911 | 8.9E-44 | DOWN |
| ENSSSCG00000009101 | PRDM5 | -1.455450173 | 1.54E-43 | DOWN |
| ENSSSCG00000016217 | DNAJB2 | -1.618659278 | 1.86E-43 | DOWN |
| ENSSSCG00000016342 | HES6 | -2.181806507 | 2.96E-43 | DOWN |
| ENSSSCG00000036751 | PPM1H | -1.507674174 | 3E-43 | DOWN |
| ENSSSCG00000002681 | HSDL1 | -1.255640851 | 4.69E-43 | DOWN |
| ENSSSCG00000016002 | NA | -2.238489675 | 6.3E-43 | DOWN |
| ENSSSCG00000037015 | SESN3 | -1.271152589 | 1.62E-42 | DOWN |
| ENSSSCG00000033993 | PLCXD3 | -1.65226917 | 1.89E-42 | DOWN |
| ENSSSCG00000010589 | SFXN2 | -1.360014403 | 5.86E-42 | DOWN |
| ENSSSCG00000039056 | GAS7 | -1.450647056 | 7.67E-42 | DOWN |
| ENSSSCG00000020931 | MSH6 | -1.031253009 | 1.65E-41 | DOWN |
| ENSSSCG00000011593 | TMCC1 | -1.11466277 | 1.74E-41 | DOWN |
| ENSSSCG00000037499 | NEDD4 | -1.168776176 | 2.31E-41 | DOWN |
| ENSSSCG00000032684 | BOK | -1.553421416 | 2.49E-41 | DOWN |
| ENSSSCG00000011538 | LMCD1 | -3.967377501 | 2.5E-41 | DOWN |
| ENSSSCG00000034191 | SOX6 | -1.589491547 | 2.79E-41 | DOWN |
| ENSSSCG00000038071 | RASL12 | -1.681046129 | 2.95E-41 | DOWN |
| ENSSSCG00000011514 | MITF | -1.848213716 | 4.23E-41 | DOWN |
| ENSSSCG00000021138 | CEP250 | -1.364759247 | 4.51E-41 | DOWN |
| ENSSSCG00000005204 | RANBP6 | -1.104236779 | 5.85E-41 | DOWN |
| ENSSSCG00000011765 | USP13 | -1.713484424 | 8.49E-41 | DOWN |
| ENSSSCG00000011404 | HYAL1 | -1.712778834 | 8.81E-41 | DOWN |
| ENSSSCG00000022797 | PPP1R3B | -1.529151517 | 1.08E-40 | DOWN |
| ENSSSCG00000003114 | DHX34 | -1.059197321 | 1.17E-40 | DOWN |
| ENSSSCG00000009605 | GFRA2 | -1.564571356 | 1.75E-40 | DOWN |
| ENSSSCG00000010259 | TYSND1 | -1.556033523 | 2.02E-40 | DOWN |
| ENSSSCG00000016206 | CNPPD1 | -1.095034698 | 2.16E-40 | DOWN |
| ENSSSCG00000004632 | GLDN | -1.635836183 | 2.36E-40 | DOWN |
| ENSSSCG00000016067 | STK17B | -1.264603317 | 3.52E-40 | DOWN |
| ENSSSCG00000036206 | C3orf58 | -1.231574638 | 5.98E-40 | DOWN |
| ENSSSCG00000039348 | H1F0 | -1.293437673 | 6.8E-40 | DOWN |
| ENSSSCG00000038852 | NA | -1.244238298 | 8.28E-40 | DOWN |
| ENSSSCG00000008259 | LRRTM4 | -1.611414256 | 2.06E-39 | DOWN |
| ENSSSCG00000032831 | BRI3BP | -1.650918458 | 2.26E-39 | DOWN |
| ENSSSCG00000001392 | TCF19 | -1.400318776 | 3.55E-39 | DOWN |
| ENSSSCG00000003471 | EPHA2 | -1.135832391 | 4.7E-39 | DOWN |
| ENSSSCG00000031346 | CMKLR1 | -1.381808666 | 8.65E-39 | DOWN |
| ENSSSCG00000021204 | HOXA10 | -1.511763788 | 1.34E-38 | DOWN |
| ENSSSCG00000015368 | HDAC9 | -1.90181859 | 6.16E-38 | DOWN |
| ENSSSCG00000040208 | PAG1 | -1.763035404 | 8.62E-38 | DOWN |
| ENSSSCG00000011326 | PTH1R | -2.119344277 | 9.2E-38 | DOWN |
| ENSSSCG00000023155 | NA | -1.342098754 | 1.5E-37 | DOWN |
| ENSSSCG00000001646 | BICRAL | -1.01769008 | 1.77E-37 | DOWN |
| ENSSSCG00000031830 | NA | -1.119484393 | 1.88E-37 | DOWN |
| ENSSSCG00000039261 | WSCD2 | -1.627184473 | 1.97E-37 | DOWN |
| ENSSSCG00000008646 | RNF144A | -1.145283952 | 2.07E-37 | DOWN |
| ENSSSCG00000016368 | FARP2 | -1.284097597 | 3.01E-37 | DOWN |
| ENSSSCG00000026326 | CCNF | -1.001812072 | 4.66E-37 | DOWN |
| ENSSSCG00000014998 | AASDHPPT | -1.098503419 | 1.53E-36 | DOWN |
| ENSSSCG00000029165 | DOK4 | -1.359645386 | 2.43E-36 | DOWN |
| ENSSSCG00000030485 | ELFN1 | -2.150976432 | 3.97E-36 | DOWN |
| ENSSSCG00000002651 | CDT1 | -1.341890525 | 7.2E-36 | DOWN |
| ENSSSCG00000016531 | C7orf49 | -1.417176529 | 9.89E-36 | DOWN |
| ENSSSCG00000031701 | GXYLT1 | -1.012016335 | 1.55E-35 | DOWN |
| ENSSSCG00000000739 | FOXM1 | -1.228963148 | 1.68E-35 | DOWN |
| ENSSSCG00000015982 | HOXD9 | -1.391257045 | 5.34E-35 | DOWN |
| ENSSSCG00000030294 | ZNF398 | -1.418270904 | 8.04E-35 | DOWN |
| ENSSSCG00000002831 | IRX3 | -2.339930976 | 9.42E-35 | DOWN |
| ENSSSCG00000026248 | PAQR4 | -1.137813129 | 1.27E-34 | DOWN |
| ENSSSCG00000014598 | PPFIBP2 | -2.25613078 | 1.96E-34 | DOWN |
| ENSSSCG00000022280 | DACT3 | -1.146230244 | 1.99E-34 | DOWN |
| ENSSSCG00000036755 | FAM46B | -3.169736467 | 2.36E-34 | DOWN |
| ENSSSCG00000005400 | GRIN3A | -2.3523066 | 2.11E-33 | DOWN |
| ENSSSCG00000013079 | DAGLA | -1.371471901 | 4.16E-33 | DOWN |
| ENSSSCG00000039875 | NKD1 | -1.774638308 | 4.69E-33 | DOWN |
| ENSSSCG00000007530 | PPP1R3D | -1.745986134 | 5.98E-33 | DOWN |
| ENSSSCG00000017311 | MAPT | -1.150317246 | 6.39E-33 | DOWN |
| ENSSSCG00000006530 | EFNA1 | -2.939717761 | 7.92E-33 | DOWN |
| ENSSSCG00000016784 | ANKH | -1.050585828 | 1.25E-32 | DOWN |
| ENSSSCG00000026404 | SERTAD4 | -1.087592951 | 1.78E-32 | DOWN |
| ENSSSCG00000009389 | TRIM13 | -1.532435833 | 1.83E-32 | DOWN |
| ENSSSCG00000024261 | CBX2 | -2.484623383 | 2.5E-32 | DOWN |
| ENSSSCG00000003719 | NA | -1.21483902 | 2.62E-32 | DOWN |
| ENSSSCG00000036160 | ZNF250 | -1.129649698 | 3.3E-32 | DOWN |
| ENSSSCG00000009229 | ARHGAP24 | -2.177146271 | 3.41E-32 | DOWN |
| ENSSSCG00000002838 | ZNF423 | -1.495600716 | 3.43E-32 | DOWN |
| ENSSSCG00000030018 | ZNF396 | -1.895160009 | 3.8E-32 | DOWN |
| ENSSSCG00000015113 | NLRX1 | -1.012300762 | 5.06E-32 | DOWN |
| ENSSSCG00000008606 | OSR1 | -1.302645692 | 5.08E-32 | DOWN |
| ENSSSCG00000004018 | AFDN | -1.115766674 | 6.18E-32 | DOWN |
| ENSSSCG00000005045 | BMP4 | -1.21811074 | 7.25E-32 | DOWN |
| ENSSSCG00000021232 | SYNC | -1.16324768 | 1.33E-31 | DOWN |
| ENSSSCG00000038701 | EFNA2 | -2.128264073 | 1.47E-31 | DOWN |
| ENSSSCG00000015986 | HOXD1 | -2.187638361 | 1.56E-31 | DOWN |
| ENSSSCG00000008703 | ADRA2C | -1.526703251 | 1.77E-31 | DOWN |
| ENSSSCG00000014892 | USP35 | -1.777579311 | 2.82E-31 | DOWN |
| ENSSSCG00000022705 | SALL2 | -2.152231177 | 3.43E-31 | DOWN |
| ENSSSCG00000033178 | NA | -2.052195437 | 3.53E-31 | DOWN |
| ENSSSCG00000011732 | TRIM59 | -1.070560258 | 4.52E-31 | DOWN |
| ENSSSCG00000003017 | TGFB1 | -1.01945625 | 9.08E-31 | DOWN |
| ENSSSCG00000012952 | TMEM151A | -2.280938101 | 9.47E-31 | DOWN |
| ENSSSCG00000010108 | KLHL22 | -1.049143811 | 1.1E-30 | DOWN |
| ENSSSCG00000036437 | NOG | -2.377964092 | 1.43E-30 | DOWN |
| ENSSSCG00000005052 | WDHD1 | -1.395726922 | 1.51E-30 | DOWN |
| ENSSSCG00000000738 | RHNO1 | -1.134461667 | 1.71E-30 | DOWN |
| ENSSSCG00000023975 | C15orf52 | -1.638507533 | 2.29E-30 | DOWN |
| ENSSSCG00000008545 | ZNF512 | -1.998584285 | 4.12E-30 | DOWN |
| ENSSSCG00000040013 | MTUS1 | -1.436137717 | 5.4E-30 | DOWN |
| ENSSSCG00000026113 | ZBTB20 | -1.169431295 | 2.15E-29 | DOWN |
| ENSSSCG00000002930 | ZNF260 | -1.288296511 | 6.36E-29 | DOWN |
| ENSSSCG00000011125 | GATA3 | -1.543053044 | 8.22E-29 | DOWN |
| ENSSSCG00000010026 | PIK3IP1 | -1.744265238 | 1.5E-28 | DOWN |
| ENSSSCG00000037016 | ID1 | -1.371894617 | 2.24E-28 | DOWN |
| ENSSSCG00000023681 | PWWP2B | -1.242948984 | 2.38E-28 | DOWN |
| ENSSSCG00000009676 | ZNF395 | -2.564441706 | 2.54E-28 | DOWN |
| ENSSSCG00000035908 | NA | -1.091987394 | 2.79E-28 | DOWN |
| ENSSSCG00000003628 | CLSPN | -1.183425302 | 7.03E-28 | DOWN |
| ENSSSCG00000009854 | RFC5 | -1.052616224 | 7.17E-28 | DOWN |
| ENSSSCG00000002348 | NA | -1.468701408 | 1.23E-27 | DOWN |
| ENSSSCG00000031744 | NA | -1.813197849 | 3.33E-27 | DOWN |
| ENSSSCG00000036975 | PSRC1 | -1.440315543 | 3.76E-27 | DOWN |
| ENSSSCG00000026991 | LURAP1 | -2.087047126 | 4.19E-27 | DOWN |
| ENSSSCG00000004490 | SETBP1 | -1.223444902 | 4.28E-27 | DOWN |
| ENSSSCG00000016170 | BARD1 | -1.596021591 | 6.47E-27 | DOWN |
| ENSSSCG00000008281 | RTKN | -1.066896999 | 8.48E-27 | DOWN |
| ENSSSCG00000036428 | NA | -1.053722208 | 1.01E-26 | DOWN |
| ENSSSCG00000008919 | EPHA5 | -2.208676613 | 1.26E-26 | DOWN |
| ENSSSCG00000035448 | SS18L1 | -1.574233996 | 1.77E-26 | DOWN |
| ENSSSCG00000037580 | ITPRIPL1 | -1.122079807 | 2.14E-26 | DOWN |
| ENSSSCG00000034049 | NA | -1.175014977 | 4.27E-26 | DOWN |
| ENSSSCG00000011888 | GPR156 | -1.465901178 | 5.13E-26 | DOWN |
| ENSSSCG00000001977 | STXBP6 | -1.091846116 | 7.28E-26 | DOWN |
| ENSSSCG00000009053 | RNF150 | -1.231248654 | 1.15E-25 | DOWN |
| ENSSSCG00000004782 | BUB1B | -1.001227071 | 1.62E-25 | DOWN |
| ENSSSCG00000003551 | PAQR7 | -1.015780195 | 1.63E-24 | DOWN |
| ENSSSCG00000012944 | PELI3 | -1.613637754 | 1.69E-24 | DOWN |
| ENSSSCG00000011914 | ZDHHC23 | -1.466867496 | 1.81E-24 | DOWN |
| ENSSSCG00000035355 | F2R | -1.51618736 | 2.32E-24 | DOWN |
| ENSSSCG00000012504 | NAP1L3 | -1.60927586 | 3.42E-24 | DOWN |
| ENSSSCG00000039838 | RGS10 | -1.673399408 | 3.65E-24 | DOWN |
| ENSSSCG00000012151 | NHS | -1.699726594 | 4.7E-24 | DOWN |
| ENSSSCG00000037391 | NA | -2.095012818 | 6.48E-24 | DOWN |
| ENSSSCG00000024780 | ZFAND1 | -1.029049168 | 6.96E-24 | DOWN |
| ENSSSCG00000004390 | SESN1 | -1.067835612 | 7.83E-24 | DOWN |
| ENSSSCG00000000399 | TIMELESS | -1.262348854 | 8.09E-24 | DOWN |
| ENSSSCG00000034328 | SP7 | -5.782813951 | 8.84E-24 | DOWN |
| ENSSSCG00000029838 | FZD2 | -1.717557745 | 1.09E-23 | DOWN |
| ENSSSCG00000015036 | DIXDC1 | -1.082655225 | 1.78E-23 | DOWN |
| ENSSSCG00000001750 | PAQR8 | -1.079166893 | 2.75E-23 | DOWN |
| ENSSSCG00000011787 | MAGEF1 | -1.0056494 | 4.91E-23 | DOWN |
| ENSSSCG00000037100 | OPA3 | -1.006165822 | 7.63E-23 | DOWN |
| ENSSSCG00000039373 | SNX24 | -1.201634445 | 7.89E-23 | DOWN |
| ENSSSCG00000000695 | IFFO1 | -1.000411558 | 8.69E-23 | DOWN |
| ENSSSCG00000017515 | TBKBP1 | -1.07608889 | 1.64E-22 | DOWN |
| ENSSSCG00000038902 | KCNK6 | -1.389599202 | 2.12E-22 | DOWN |
| ENSSSCG00000014581 | TUB | -1.371570666 | 2.4E-22 | DOWN |
| ENSSSCG00000001885 | C15orf39 | -1.461681589 | 3.04E-22 | DOWN |
| ENSSSCG00000024158 | ANO1 | -1.570272347 | 4.1E-22 | DOWN |
| ENSSSCG00000010027 | PATZ1 | -1.049421873 | 5.97E-22 | DOWN |
| ENSSSCG00000004109 | ZC3H12D | -2.798570096 | 6.5E-22 | DOWN |
| ENSSSCG00000024134 | MGLL | -1.334075023 | 6.75E-22 | DOWN |
| ENSSSCG00000031589 | NA | -1.473778513 | 8.72E-22 | DOWN |
| ENSSSCG00000033521 | NA | -1.003123082 | 1.49E-21 | DOWN |
| ENSSSCG00000022987 | TMEM132E | -1.450633336 | 1.54E-21 | DOWN |
| ENSSSCG00000002847 | GPT2 | -1.464986859 | 1.57E-21 | DOWN |
| ENSSSCG00000029408 | ZFP30 | -1.285961041 | 2.06E-21 | DOWN |
| ENSSSCG00000011743 | MECOM | -1.178419687 | 3.99E-21 | DOWN |
| ENSSSCG00000035539 | ST8SIA4 | -1.726886503 | 5.97E-21 | DOWN |
| ENSSSCG00000007954 | SLX4 | -1.019863818 | 6.16E-21 | DOWN |
| ENSSSCG00000005376 | TBC1D2 | -1.231382048 | 6.35E-21 | DOWN |
| ENSSSCG00000039861 | HDHD3 | -1.105313896 | 7.14E-21 | DOWN |
| ENSSSCG00000016625 | CTTNBP2 | -1.204996785 | 7.41E-21 | DOWN |
| ENSSSCG00000008241 | TCF7L1 | -1.184618686 | 7.79E-21 | DOWN |
| ENSSSCG00000012284 | ZNF182 | -1.906252642 | 8.36E-21 | DOWN |
| ENSSSCG00000029039 | BRCA2 | -1.185159753 | 9.56E-21 | DOWN |
| ENSSSCG00000031652 | RPGRIP1L | -1.259100394 | 9.59E-21 | DOWN |
| ENSSSCG00000008197 | SEMA4C | -1.009602063 | 1.13E-20 | DOWN |
| ENSSSCG00000022611 | CAPRIN2 | -2.067386457 | 1.17E-20 | DOWN |
| ENSSSCG00000016678 | NOD1 | -1.102104912 | 1.27E-20 | DOWN |
| ENSSSCG00000022636 | DENND5B | -1.150564803 | 1.41E-20 | DOWN |
| ENSSSCG00000031072 | ZNF514 | -1.761568862 | 1.43E-20 | DOWN |
| ENSSSCG00000017313 | NA | -2.266052934 | 1.47E-20 | DOWN |
| ENSSSCG00000009243 | THAP9 | -1.003916358 | 2.05E-20 | DOWN |
| ENSSSCG00000021638 | NEU3 | -1.369118236 | 2.13E-20 | DOWN |
| ENSSSCG00000017518 | OSBPL7 | -1.268362565 | 2.7E-20 | DOWN |
| ENSSSCG00000014878 | PAK1 | -1.482944072 | 2.96E-20 | DOWN |
| ENSSSCG00000034262 | FIGN | -2.117017078 | 3.14E-20 | DOWN |
| ENSSSCG00000017589 | DLX3 | -1.143697321 | 3.7E-20 | DOWN |
| ENSSSCG00000021920 | SOX12 | -2.326846388 | 5.09E-20 | DOWN |
| ENSSSCG00000021704 | PTPDC1 | -1.803010325 | 7.4E-20 | DOWN |
| ENSSSCG00000000936 | SLC6A15 | -1.148470843 | 7.95E-20 | DOWN |
| ENSSSCG00000005314 | ARHGEF39 | -1.839805339 | 9.1E-20 | DOWN |
| ENSSSCG00000003341 | TAS1R3 | -1.454904513 | 1.07E-19 | DOWN |
| ENSSSCG00000037433 | C3orf18 | -2.270433469 | 1.09E-19 | DOWN |
| ENSSSCG00000015593 | NSL1 | -1.469564637 | 1.55E-19 | DOWN |
| ENSSSCG00000039412 | DOC2B | -1.723901055 | 1.99E-19 | DOWN |
| ENSSSCG00000039568 | SNAI2 | -1.207961219 | 2.61E-19 | DOWN |
| ENSSSCG00000006162 | ZC2HC1A | -1.723510389 | 6.86E-19 | DOWN |
| ENSSSCG00000023776 | CCSAP | -1.148580583 | 7.54E-19 | DOWN |
| ENSSSCG00000000791 | PDZRN4 | -3.028083394 | 1.22E-18 | DOWN |
| ENSSSCG00000032372 | ZFP90 | -1.028645374 | 1.3E-18 | DOWN |
| ENSSSCG00000005020 | ATL1 | -1.035270645 | 1.34E-18 | DOWN |
| ENSSSCG00000002383 | FOS | -1.751062256 | 1.4E-18 | DOWN |
| ENSSSCG00000040985 | KCTD7 | -2.487888501 | 1.76E-18 | DOWN |
| ENSSSCG00000002648 | CBFA2T3 | -2.011882756 | 1.78E-18 | DOWN |
| ENSSSCG00000016836 | NADK2 | -1.080349887 | 2.18E-18 | DOWN |
| ENSSSCG00000009351 | MAB21L1 | -1.229094648 | 2.28E-18 | DOWN |
| ENSSSCG00000003783 | FPGT | -1.728304683 | 2.28E-18 | DOWN |
| ENSSSCG00000005025 | TRIM9 | -1.148148447 | 2.83E-18 | DOWN |
| ENSSSCG00000008464 | MTA3 | -1.107021712 | 6.83E-18 | DOWN |
| ENSSSCG00000000531 | BICD1 | -2.981860977 | 1.13E-17 | DOWN |
| ENSSSCG00000011215 | OXSM | -1.032461424 | 1.15E-17 | DOWN |
| ENSSSCG00000023303 | FIGNL1 | -2.097069415 | 1.35E-17 | DOWN |
| ENSSSCG00000006947 | SYDE2 | -1.068334146 | 1.72E-17 | DOWN |
| ENSSSCG00000038569 | ARMCX1 | -1.027344381 | 2.2E-17 | DOWN |
| ENSSSCG00000008892 | NA | -1.085444403 | 2.55E-17 | DOWN |
| ENSSSCG00000010222 | ZNF365 | -3.082131359 | 2.82E-17 | DOWN |
| ENSSSCG00000004813 | TTC23 | -1.040921195 | 3.38E-17 | DOWN |
| ENSSSCG00000036452 | LRRC17 | -1.228301456 | 3.45E-17 | DOWN |
| ENSSSCG00000014908 | CCDC89 | -3.022163529 | 4.05E-17 | DOWN |
| ENSSSCG00000031102 | C1orf174 | -1.007744067 | 4.49E-17 | DOWN |
| ENSSSCG00000012283 | ZNF81 | -1.261700276 | 5.71E-17 | DOWN |
| ENSSSCG00000035664 | ZBTB45 | -1.074861361 | 5.99E-17 | DOWN |
| ENSSSCG00000026055 | PRIM1 | -1.059827139 | 6.08E-17 | DOWN |
| ENSSSCG00000033032 | ZBTB14 | -1.134993496 | 8.14E-17 | DOWN |
| ENSSSCG00000038095 | NA | -1.254270456 | 8.99E-17 | DOWN |
| ENSSSCG00000027478 | PPCDC | -1.080381001 | 1.1E-16 | DOWN |
| ENSSSCG00000014406 | PRELID2 | -1.266284834 | 1.45E-16 | DOWN |
| ENSSSCG00000003112 | NA | -1.961302894 | 1.65E-16 | DOWN |
| ENSSSCG00000015507 | TNN | -1.354314778 | 1.9E-16 | DOWN |
| ENSSSCG00000002464 | PRIMA1 | -2.73782507 | 2.38E-16 | DOWN |
| ENSSSCG00000005657 | PKN3 | -1.308562184 | 2.81E-16 | DOWN |
| ENSSSCG00000006240 | FAM110B | -1.412099434 | 4.31E-16 | DOWN |
| ENSSSCG00000035055 | NA | -1.299964159 | 7.61E-16 | DOWN |
| ENSSSCG00000027105 | TUBGCP5 | -1.034685745 | 1.52E-15 | DOWN |
| ENSSSCG00000035284 | BMF | -1.602800509 | 1.77E-15 | DOWN |
| ENSSSCG00000017391 | PLEKHH3 | -1.048807753 | 1.8E-15 | DOWN |
| ENSSSCG00000021273 | NA | -1.996238617 | 1.94E-15 | DOWN |
| ENSSSCG00000032383 | NA | -1.80142458 | 5.33E-15 | DOWN |
| ENSSSCG00000015281 | PLEKHA6 | -2.277831563 | 8.68E-15 | DOWN |
| ENSSSCG00000003269 | LENG9 | -1.153981414 | 1.05E-14 | DOWN |
| ENSSSCG00000017879 | SPNS2 | -3.654215885 | 1.15E-14 | DOWN |
| ENSSSCG00000034821 | ARMCX4 | -1.914897884 | 1.84E-14 | DOWN |
| ENSSSCG00000031706 | MICALCL | -1.876030543 | 1.88E-14 | DOWN |
| ENSSSCG00000016782 | FAM105A | -1.083409258 | 1.94E-14 | DOWN |
| ENSSSCG00000006982 | ZDHHC2 | -1.021512995 | 2.21E-14 | DOWN |
| ENSSSCG00000027016 | C19orf47 | -1.02131096 | 2.3E-14 | DOWN |
| ENSSSCG00000010376 | GDF10 | -2.165572849 | 2.4E-14 | DOWN |
| ENSSSCG00000023760 | CLEC14A | -1.41783553 | 3.25E-14 | DOWN |
| ENSSSCG00000011618 | GATA2 | -1.391340886 | 3.7E-14 | DOWN |
| ENSSSCG00000031925 | SHANK3 | -1.303352161 | 4.22E-14 | DOWN |
| ENSSSCG00000014385 | PCDHGC5 | -1.508598562 | 5.18E-14 | DOWN |
| ENSSSCG00000040215 | TFAP4 | -1.223736031 | 5.32E-14 | DOWN |
| ENSSSCG00000006780 | WNT2B | -2.350175043 | 5.36E-14 | DOWN |
| ENSSSCG00000021749 | MCF2L2 | -1.475301518 | 5.79E-14 | DOWN |
| ENSSSCG00000010012 | SLC35E4 | -1.22931606 | 6.34E-14 | DOWN |
| ENSSSCG00000038644 | HOXC8 | -2.450166167 | 8.63E-14 | DOWN |
| ENSSSCG00000021683 | NA | -1.911656894 | 1.01E-13 | DOWN |
| ENSSSCG00000010497 | NA | -1.263557877 | 1.02E-13 | DOWN |
| ENSSSCG00000004300 | SMIM8 | -1.483710066 | 1.09E-13 | DOWN |
| ENSSSCG00000039677 | CHRNA4 | -1.846745906 | 1.29E-13 | DOWN |
| ENSSSCG00000008722 | NA | -1.204527865 | 1.63E-13 | DOWN |
| ENSSSCG00000015954 | DLX2 | -2.417187885 | 1.73E-13 | DOWN |
| ENSSSCG00000022347 | WNT11 | -1.338444234 | 2.09E-13 | DOWN |
| ENSSSCG00000040937 | ALKBH3 | -1.178270741 | 2.12E-13 | DOWN |
| ENSSSCG00000032153 | C19orf12 | -1.02453583 | 2.18E-13 | DOWN |
| ENSSSCG00000029866 | CCDC112 | -2.014041754 | 3.58E-13 | DOWN |
| ENSSSCG00000006940 | CYR61 | -1.001900808 | 3.9E-13 | DOWN |
| ENSSSCG00000002353 | FAM161B | -1.162597212 | 4.29E-13 | DOWN |
| ENSSSCG00000009630 | EGR3 | -1.592996781 | 5.12E-13 | DOWN |
| ENSSSCG00000017892 | PITPNM3 | -2.288059904 | 5.48E-13 | DOWN |
| ENSSSCG00000031936 | GJB2 | -2.717907276 | 6.38E-13 | DOWN |
| ENSSSCG00000006121 | PIP4P2 | -1.901301375 | 6.89E-13 | DOWN |
| ENSSSCG00000009729 | ZNF84 | -1.564785862 | 7.28E-13 | DOWN |
| ENSSSCG00000014437 | PPARGC1B | -1.160540305 | 9.06E-13 | DOWN |
| ENSSSCG00000004170 | NA | -2.076423085 | 1.34E-12 | DOWN |
| ENSSSCG00000039161 | MEIS1 | -1.560899127 | 1.37E-12 | DOWN |
| ENSSSCG00000028327 | RHOBTB1 | -1.111839476 | 2.06E-12 | DOWN |
| ENSSSCG00000032834 | ZNF169 | -1.087144751 | 2.22E-12 | DOWN |
| ENSSSCG00000016992 | NEURL1B | -3.786953029 | 2.31E-12 | DOWN |
| ENSSSCG00000007252 | DNMT3B | -1.336198497 | 2.32E-12 | DOWN |
| ENSSSCG00000026346 | UBE3D | -1.609572533 | 2.67E-12 | DOWN |
| ENSSSCG00000014830 | COA4 | -1.163242365 | 2.7E-12 | DOWN |
| ENSSSCG00000035222 | CHST8 | -1.773926048 | 2.89E-12 | DOWN |
| ENSSSCG00000010416 | ZNF32 | -1.35041257 | 2.96E-12 | DOWN |
| ENSSSCG00000035891 | ADGRB1 | -2.581516261 | 3.42E-12 | DOWN |
| ENSSSCG00000022247 | PROSER2 | -1.3185085 | 3.49E-12 | DOWN |
| ENSSSCG00000031764 | NA | -1.771344024 | 3.91E-12 | DOWN |
| ENSSSCG00000025349 | CCDC14 | -1.141927117 | 5.48E-12 | DOWN |
| ENSSSCG00000040905 | FAAP24 | -1.197437278 | 7.14E-12 | DOWN |
| ENSSSCG00000031849 | NA | -1.10751413 | 8.45E-12 | DOWN |
| ENSSSCG00000012371 | AR | -1.201073238 | 8.84E-12 | DOWN |
| ENSSSCG00000015985 | HOXD3 | -1.593188325 | 1.11E-11 | DOWN |
| ENSSSCG00000038290 | RNF182 | -2.352812507 | 1.26E-11 | DOWN |
| ENSSSCG00000017376 | MEOX1 | -2.068661982 | 1.37E-11 | DOWN |
| ENSSSCG00000016674 | MINDY4 | -1.103163393 | 1.38E-11 | DOWN |
| ENSSSCG00000033766 | ETFBKMT | -1.962929465 | 2.39E-11 | DOWN |
| ENSSSCG00000004518 | NA | -1.041627353 | 2.8E-11 | DOWN |
| ENSSSCG00000009283 | TNFRSF19 | -1.626320827 | 2.94E-11 | DOWN |
| ENSSSCG00000007531 | FAM217B | -1.154278586 | 3.13E-11 | DOWN |
| ENSSSCG00000027052 | PEX11A | -1.128939772 | 4E-11 | DOWN |
| ENSSSCG00000004548 | PIF1 | -1.727142709 | 4.02E-11 | DOWN |
| ENSSSCG00000001637 | GUCA1B | -1.77352214 | 4.06E-11 | DOWN |
| ENSSSCG00000018758 | ssc-mir-214 | -1.540212182 | 4.67E-11 | DOWN |
| ENSSSCG00000034184 | NA | -2.115624268 | 5.25E-11 | DOWN |
| ENSSSCG00000004750 | OIP5 | -1.156569505 | 6.25E-11 | DOWN |
| ENSSSCG00000009774 | C12orf65 | -1.191200292 | 6.36E-11 | DOWN |
| ENSSSCG00000032157 | NA | -3.620198378 | 6.41E-11 | DOWN |
| ENSSSCG00000009901 | SIRT4 | -1.891637538 | 6.42E-11 | DOWN |
| ENSSSCG00000027952 | ADCY5 | -1.63094011 | 6.76E-11 | DOWN |
| ENSSSCG00000015742 | TMEM177 | -1.641343325 | 7.34E-11 | DOWN |
| ENSSSCG00000017789 | ABHD15 | -3.109805918 | 9.51E-11 | DOWN |
| ENSSSCG00000030016 | PDE9A | -2.435121384 | 1.14E-10 | DOWN |
| ENSSSCG00000034474 | HOXC9 | -2.37513946 | 1.4E-10 | DOWN |
| ENSSSCG00000038384 | COX4I2 | -1.111909435 | 1.44E-10 | DOWN |
| ENSSSCG00000033788 | OXLD1 | -1.07611077 | 1.72E-10 | DOWN |
| ENSSSCG00000028790 | ANKRD16 | -1.183603457 | 1.91E-10 | DOWN |
| ENSSSCG00000031565 | NA | -1.89241254 | 2.06E-10 | DOWN |
| ENSSSCG00000002793 | NA | -1.113466323 | 2.09E-10 | DOWN |
| ENSSSCG00000000111 | BAIAP2L2 | -1.000952145 | 2.44E-10 | DOWN |
| ENSSSCG00000016679 | NA | -1.097229325 | 2.49E-10 | DOWN |
| ENSSSCG00000010076 | ZNF70 | -1.824913274 | 2.82E-10 | DOWN |
| ENSSSCG00000029753 | CYTIP | -1.592291711 | 3.07E-10 | DOWN |
| ENSSSCG00000017041 | ADRA1B | -2.13047868 | 3.34E-10 | DOWN |
| ENSSSCG00000037195 | FOXF2 | -2.046294582 | 3.58E-10 | DOWN |
| ENSSSCG00000002900 | NA | -1.064885751 | 5.25E-10 | DOWN |
| ENSSSCG00000038048 | NECAB2 | -2.116311647 | 5.68E-10 | DOWN |
| ENSSSCG00000010031 | NA | -1.862513044 | 6.15E-10 | DOWN |
| ENSSSCG00000016157 | NA | -1.177220848 | 6.56E-10 | DOWN |
| ENSSSCG00000001808 | CPEB1 | -1.575586965 | 6.92E-10 | DOWN |
| ENSSSCG00000014187 | NUDT12 | -1.020115467 | 8.14E-10 | DOWN |
| ENSSSCG00000017640 | RNF43 | -1.98937539 | 8.91E-10 | DOWN |
| ENSSSCG00000015947 | DCAF17 | -1.006804151 | 1.47E-09 | DOWN |
| ENSSSCG00000011075 | KIAA1217 | -1.286821645 | 1.47E-09 | DOWN |
| ENSSSCG00000032250 | SRRM3 | -2.697697302 | 1.53E-09 | DOWN |
| ENSSSCG00000029843 | NPHP4 | -1.140752558 | 1.66E-09 | DOWN |
| ENSSSCG00000001621 | TFEB | -1.375950004 | 1.68E-09 | DOWN |
| ENSSSCG00000033759 | TBXA2R | -1.825345454 | 1.71E-09 | DOWN |
| ENSSSCG00000033456 | GPR157 | -1.851907894 | 1.91E-09 | DOWN |
| ENSSSCG00000027970 | NA | -1.553830839 | 2.28E-09 | DOWN |
| ENSSSCG00000023848 | C8orf46 | -1.79171392 | 3.42E-09 | DOWN |
| ENSSSCG00000040875 | ZFPM1 | -1.407890065 | 3.56E-09 | DOWN |
| ENSSSCG00000002988 | NA | -1.386913279 | 3.93E-09 | DOWN |
| ENSSSCG00000010133 | ARVCF | -1.137323683 | 4.9E-09 | DOWN |
| ENSSSCG00000017510 | CACNB1 | -1.169588211 | 4.92E-09 | DOWN |
| ENSSSCG00000034008 | NA | -2.220268589 | 5.32E-09 | DOWN |
| ENSSSCG00000032969 | NA | -1.29604264 | 6.25E-09 | DOWN |
| ENSSSCG00000007949 | SRL | -2.330317523 | 6.35E-09 | DOWN |
| ENSSSCG00000023376 | PARD6G | -1.476266758 | 6.46E-09 | DOWN |
| ENSSSCG00000011412 | CACNA2D2 | -1.406473212 | 6.83E-09 | DOWN |
| ENSSSCG00000037354 | MMACHC | -1.796838626 | 7.08E-09 | DOWN |
| ENSSSCG00000038296 | NA | -2.387061811 | 7.23E-09 | DOWN |
| ENSSSCG00000006321 | FAM78B | -1.420631133 | 8.58E-09 | DOWN |
| ENSSSCG00000015816 | LETM2 | -1.5184839 | 8.73E-09 | DOWN |
| ENSSSCG00000033800 | PELI2 | -1.90222882 | 1.18E-08 | DOWN |
| ENSSSCG00000011217 | NEK10 | -1.70093538 | 1.21E-08 | DOWN |
| ENSSSCG00000025335 | ZSCAN31 | -1.034737638 | 1.24E-08 | DOWN |
| ENSSSCG00000034632 | PDXP | -1.53852555 | 1.31E-08 | DOWN |
| ENSSSCG00000017392 | CCR10 | -1.168735276 | 1.37E-08 | DOWN |
| ENSSSCG00000038662 | ZBED8 | -1.204491492 | 1.52E-08 | DOWN |
| ENSSSCG00000035182 | SDR42E1 | -1.012718408 | 1.74E-08 | DOWN |
| ENSSSCG00000027790 | NA | -2.168474111 | 1.86E-08 | DOWN |
| ENSSSCG00000006495 | SEMA4A | -3.081567589 | 2.21E-08 | DOWN |
| ENSSSCG00000015862 | LIMS2 | -1.02182139 | 2.51E-08 | DOWN |
| ENSSSCG00000039890 | RASL11A | -1.550486842 | 2.53E-08 | DOWN |
| ENSSSCG00000007072 | SPTLC3 | -1.035665038 | 2.56E-08 | DOWN |
| ENSSSCG00000008720 | HMX1 | -2.966399787 | 3.08E-08 | DOWN |
| ENSSSCG00000002857 | NA | -1.067839392 | 3.34E-08 | DOWN |
| ENSSSCG00000033616 | ZNF10 | -1.004813566 | 4.03E-08 | DOWN |
| ENSSSCG00000025160 | DPF1 | -1.672533789 | 4.56E-08 | DOWN |
| ENSSSCG00000016175 | MREG | -1.14993688 | 4.67E-08 | DOWN |
| ENSSSCG00000032620 | PLCXD2 | -1.305076418 | 6.25E-08 | DOWN |
| ENSSSCG00000004663 | SEMA6D | -1.399717587 | 6.43E-08 | DOWN |
| ENSSSCG00000007528 | PHACTR3 | -1.342614165 | 6.45E-08 | DOWN |
| ENSSSCG00000014157 | NR2F1 | -1.115155819 | 7.16E-08 | DOWN |
| ENSSSCG00000003995 | ZNF606 | -1.115416726 | 7.5E-08 | DOWN |
| ENSSSCG00000024663 | SPICE1 | -1.310098972 | 7.69E-08 | DOWN |
| ENSSSCG00000020987 | WDR31 | -1.447732417 | 8.94E-08 | DOWN |
| ENSSSCG00000013276 | PRDM11 | -1.566796504 | 9.05E-08 | DOWN |
| ENSSSCG00000006034 | RSPO2 | -2.023339331 | 9.93E-08 | DOWN |
| ENSSSCG00000003473 | RSG1 | -1.300560088 | 1.11E-07 | DOWN |
| ENSSSCG00000030395 | ASB5 | -1.984144988 | 1.12E-07 | DOWN |
| ENSSSCG00000013859 | C19orf44 | -1.196364229 | 1.31E-07 | DOWN |
| ENSSSCG00000013897 | RAB3A | -1.138259701 | 1.32E-07 | DOWN |
| ENSSSCG00000002258 | KLHL25 | -1.281196612 | 1.35E-07 | DOWN |
| ENSSSCG00000033314 | DLX6 | -4.812260667 | 1.36E-07 | DOWN |
| ENSSSCG00000031361 | CELSR1 | -1.079945606 | 1.45E-07 | DOWN |
| ENSSSCG00000014047 | FGFR4 | -1.150446323 | 1.47E-07 | DOWN |
| ENSSSCG00000032145 | IRX5 | -3.41112723 | 1.52E-07 | DOWN |
| ENSSSCG00000005249 | FAM189A2 | -1.557660446 | 1.61E-07 | DOWN |
| ENSSSCG00000011271 | ZNF619 | -1.047400931 | 1.65E-07 | DOWN |
| ENSSSCG00000021490 | PRDM16 | -1.322686523 | 1.66E-07 | DOWN |
| ENSSSCG00000015346 | ICA1 | -1.125823442 | 1.66E-07 | DOWN |
| ENSSSCG00000036261 | CROCC2 | -1.645965558 | 1.69E-07 | DOWN |
| ENSSSCG00000015474 | PPFIA4 | -1.365480348 | 1.73E-07 | DOWN |
| ENSSSCG00000005008 | POLE2 | -1.326865059 | 1.89E-07 | DOWN |
| ENSSSCG00000035243 | RAB27B | -1.147992249 | 2.83E-07 | DOWN |
| ENSSSCG00000016873 | NIM1K | -1.67964445 | 3.08E-07 | DOWN |
| ENSSSCG00000027505 | ESYT3 | -1.807130529 | 3.39E-07 | DOWN |
| ENSSSCG00000035968 | LMX1B | -1.492626278 | 3.86E-07 | DOWN |
| ENSSSCG00000026961 | NA | -2.064439002 | 3.9E-07 | DOWN |
| ENSSSCG00000013320 | PAX6 | -1.691832338 | 4.25E-07 | DOWN |
| ENSSSCG00000029186 | SEZ6L2 | -1.222843353 | 4.56E-07 | DOWN |
| ENSSSCG00000021741 | ZNF527 | -1.40366952 | 4.56E-07 | DOWN |
| ENSSSCG00000001486 | LRRC1 | -1.571717348 | 4.7E-07 | DOWN |
| ENSSSCG00000037404 | DRGX | -1.900437044 | 4.75E-07 | DOWN |
| ENSSSCG00000034516 | CDKL1 | -1.069002919 | 5.02E-07 | DOWN |
| ENSSSCG00000036356 | ZSCAN2 | -1.130055292 | 5.39E-07 | DOWN |
| ENSSSCG00000033979 | KLHL34 | -1.498911381 | 5.4E-07 | DOWN |
| ENSSSCG00000006336 | CCDC190 | -2.495297143 | 5.46E-07 | DOWN |
| ENSSSCG00000032327 | TMEM169 | -1.511721649 | 5.59E-07 | DOWN |
| ENSSSCG00000035788 | NA | -1.752491762 | 5.61E-07 | DOWN |
| ENSSSCG00000014395 | PCDH12 | -1.363377188 | 6.01E-07 | DOWN |
| ENSSSCG00000029796 | KBTBD11 | -2.679197062 | 7.06E-07 | DOWN |
| ENSSSCG00000003243 | NA | -1.732344924 | 7.08E-07 | DOWN |
| ENSSSCG00000006810 | KCNC4 | -1.642620011 | 7.78E-07 | DOWN |
| ENSSSCG00000000523 | BBS10 | -1.323766066 | 8.45E-07 | DOWN |
| ENSSSCG00000010475 | CYP26A1 | -5.333800955 | 8.87E-07 | DOWN |
| ENSSSCG00000033363 | NA | -1.956454124 | 1.02E-06 | DOWN |
| ENSSSCG00000033809 | IFITM10 | -2.825329725 | 1.08E-06 | DOWN |
| ENSSSCG00000012164 | CNKSR2 | -1.511458044 | 1.09E-06 | DOWN |
| ENSSSCG00000028699 | NEIL2 | -1.11400145 | 1.29E-06 | DOWN |
| ENSSSCG00000027684 | TRIM63 | -2.329835014 | 1.45E-06 | DOWN |
| ENSSSCG00000038643 | KLF11 | -2.025021656 | 1.53E-06 | DOWN |
| ENSSSCG00000014015 | MRNIP | -2.171685916 | 1.71E-06 | DOWN |
| ENSSSCG00000034883 | AMIGO1 | -1.392763302 | 1.88E-06 | DOWN |
| ENSSSCG00000006793 | NA | -1.261407744 | 1.92E-06 | DOWN |
| ENSSSCG00000016451 | NA | -1.114671365 | 2.07E-06 | DOWN |
| ENSSSCG00000006635 | SEMA6C | -1.007563496 | 2.14E-06 | DOWN |
| ENSSSCG00000011439 | PHF7 | -1.866274594 | 2.35E-06 | DOWN |
| ENSSSCG00000036201 | NPR3 | -1.153327148 | 2.45E-06 | DOWN |
| ENSSSCG00000023596 | NA | -1.462342126 | 2.49E-06 | DOWN |
| ENSSSCG00000004928 | CILP | -1.070931529 | 2.63E-06 | DOWN |
| ENSSSCG00000006168 | PEX2 | -1.012293385 | 2.66E-06 | DOWN |
| ENSSSCG00000028531 | SH3BP1 | -1.25206113 | 3.09E-06 | DOWN |
| ENSSSCG00000012508 | NA | -2.001288972 | 3.19E-06 | DOWN |
| ENSSSCG00000017112 | IRX4 | -1.709350449 | 3.42E-06 | DOWN |
| ENSSSCG00000039074 | TNFRSF13C | -1.057949461 | 3.43E-06 | DOWN |
| ENSSSCG00000027423 | TMEM53 | -1.312506342 | 3.55E-06 | DOWN |
| ENSSSCG00000006497 | MEX3A | -1.159215351 | 3.8E-06 | DOWN |
| ENSSSCG00000040554 | NA | -1.078441311 | 4.26E-06 | DOWN |
| ENSSSCG00000022550 | DGKG | -2.559984481 | 4.51E-06 | DOWN |
| ENSSSCG00000010653 | TRUB1 | -1.648710542 | 4.64E-06 | DOWN |
| ENSSSCG00000008187 | KIAA1211L | -3.234379651 | 4.94E-06 | DOWN |
| ENSSSCG00000011071 | THNSL1 | -1.363848478 | 5.68E-06 | DOWN |
| ENSSSCG00000035267 | NA | -1.268800824 | 5.88E-06 | DOWN |
| ENSSSCG00000039109 | MTERF2 | -1.848120225 | 6.03E-06 | DOWN |
| ENSSSCG00000024205 | ARHGEF26 | -1.173556285 | 6.59E-06 | DOWN |
| ENSSSCG00000017129 | NA | -1.298545949 | 6.67E-06 | DOWN |
| ENSSSCG00000009679 | FZD3 | -1.484362965 | 6.89E-06 | DOWN |
| ENSSSCG00000003054 | ZNF575 | -1.027233253 | 7.12E-06 | DOWN |
| ENSSSCG00000031272 | FGF5 | -2.089027016 | 7.21E-06 | DOWN |
| ENSSSCG00000015144 | GRAMD1B | -1.241357436 | 7.58E-06 | DOWN |
| ENSSSCG00000039627 | TMEM200C | -1.192108624 | 8.25E-06 | DOWN |
| ENSSSCG00000029323 | KCNE3 | -1.766351337 | 9.04E-06 | DOWN |
| ENSSSCG00000032606 | NA | -1.929482405 | 9.7E-06 | DOWN |
| ENSSSCG00000013333 | BDNF | -1.602109262 | 1.01E-05 | DOWN |
| ENSSSCG00000023976 | DCP1B | -1.455508545 | 1.3E-05 | DOWN |
| ENSSSCG00000012785 | PDZD4 | -2.204868753 | 1.35E-05 | DOWN |
| ENSSSCG00000031905 | KCNS3 | -2.121146085 | 1.39E-05 | DOWN |
| ENSSSCG00000013327 | MPPED2 | -1.210995764 | 1.44E-05 | DOWN |
| ENSSSCG00000029875 | ZNF461 | -1.443321485 | 1.46E-05 | DOWN |
| ENSSSCG00000039821 | GPRIN3 | -4.906948064 | 1.49E-05 | DOWN |
| ENSSSCG00000040730 | NA | -2.719134739 | 1.69E-05 | DOWN |
| ENSSSCG00000008319 | CD207 | -1.201026747 | 1.73E-05 | DOWN |
| ENSSSCG00000033213 | LYRM7 | -1.044632861 | 1.81E-05 | DOWN |
| ENSSSCG00000004215 | KIAA0408 | -3.129782219 | 1.91E-05 | DOWN |
| ENSSSCG00000040603 | SGTB | -1.011100569 | 1.97E-05 | DOWN |
| ENSSSCG00000032094 | DKK2 | -1.187524718 | 2.07E-05 | DOWN |
| ENSSSCG00000031799 | TMEM139 | -2.949782251 | 2.36E-05 | DOWN |
| ENSSSCG00000000179 | CCDC65 | -1.378884753 | 2.54E-05 | DOWN |
| ENSSSCG00000032536 | B3GNT8 | -1.11065993 | 2.54E-05 | DOWN |
| ENSSSCG00000001872 | LINGO1 | -1.126187551 | 2.6E-05 | DOWN |
| ENSSSCG00000001203 | ZSCAN9 | -2.409851844 | 2.94E-05 | DOWN |
| ENSSSCG00000037099 | ANKLE1 | -1.796716663 | 3.02E-05 | DOWN |
| ENSSSCG00000021460 | NYX | -1.089038492 | 3.04E-05 | DOWN |
| ENSSSCG00000038598 | ADRB2 | -2.144602163 | 3.07E-05 | DOWN |
| ENSSSCG00000015283 | PIK3C2B | -1.042333494 | 3.07E-05 | DOWN |
| ENSSSCG00000031979 | NA | -1.906383063 | 3.15E-05 | DOWN |
| ENSSSCG00000003740 | ZSCAN30 | -2.296362227 | 3.15E-05 | DOWN |
| ENSSSCG00000039089 | NA | -1.82071511 | 3.23E-05 | DOWN |
| ENSSSCG00000006526 | TRIM46 | -1.632952605 | 3.25E-05 | DOWN |
| ENSSSCG00000011837 | MELTF | -1.015724448 | 3.36E-05 | DOWN |
| ENSSSCG00000038578 | HRH3 | -4.731565525 | 3.41E-05 | DOWN |
| ENSSSCG00000010721 | PSTK | -1.136275907 | 3.52E-05 | DOWN |
| ENSSSCG00000004990 | LRFN5 | -2.220847847 | 3.81E-05 | DOWN |
| ENSSSCG00000036052 | NA | -2.435999041 | 4.08E-05 | DOWN |
| ENSSSCG00000016963 | CCDC125 | -1.05031962 | 4.41E-05 | DOWN |
| ENSSSCG00000035682 | ANKRD63 | -3.73060384 | 4.45E-05 | DOWN |
| ENSSSCG00000004027 | PDE10A | -1.784259804 | 4.6E-05 | DOWN |
| ENSSSCG00000011881 | IQCB1 | -1.083942551 | 4.81E-05 | DOWN |
| ENSSSCG00000003921 | ZSWIM5 | -2.945716022 | 4.96E-05 | DOWN |
| ENSSSCG00000030585 | HOXC6 | -1.797416771 | 5.04E-05 | DOWN |
| ENSSSCG00000035692 | NA | -1.571732253 | 5.08E-05 | DOWN |
| ENSSSCG00000015206 | CCDC15 | -1.053262009 | 5.15E-05 | DOWN |
| ENSSSCG00000032446 | C1orf116 | -3.048209214 | 5.35E-05 | DOWN |
| ENSSSCG00000030229 | ABHD14A | -1.172334414 | 5.39E-05 | DOWN |
| ENSSSCG00000012824 | GAB3 | -1.361849815 | 5.48E-05 | DOWN |
| ENSSSCG00000036104 | NA | -1.941319217 | 5.54E-05 | DOWN |
| ENSSSCG00000034120 | CDKN2B | -1.303636377 | 5.69E-05 | DOWN |
| ENSSSCG00000022195 | NA | -1.296327842 | 6.28E-05 | DOWN |
| ENSSSCG00000015984 | HOXD4 | -1.842441433 | 6.64E-05 | DOWN |
| ENSSSCG00000027565 | ARHGAP45 | -1.079047379 | 6.89E-05 | DOWN |
| ENSSSCG00000028192 | DNAJC28 | -1.085422259 | 7.14E-05 | DOWN |
| ENSSSCG00000028927 | LGI2 | -1.676091509 | 7.48E-05 | DOWN |
| ENSSSCG00000033397 | KCNK9 | -1.28484555 | 7.58E-05 | DOWN |
| ENSSSCG00000029960 | LRRC4B | -1.002165702 | 8.23E-05 | DOWN |
| ENSSSCG00000033338 | NA | -1.111891427 | 8.72E-05 | DOWN |
| ENSSSCG00000037512 | ZSCAN23 | -2.779914419 | 9.16E-05 | DOWN |
| ENSSSCG00000007649 | GPC2 | -1.045411324 | 9.58E-05 | DOWN |
| ENSSSCG00000016672 | ADCYAP1R1 | -2.773940388 | 0.000101 | DOWN |
| ENSSSCG00000033351 | NA | -1.167844021 | 0.000105 | DOWN |
| ENSSSCG00000015753 | ANGPT2 | -1.887960733 | 0.000112 | DOWN |
| ENSSSCG00000001216 | NA | -2.012875056 | 0.000115 | DOWN |
| ENSSSCG00000017349 | ADAM11 | -1.513634982 | 0.000131 | DOWN |
| ENSSSCG00000009067 | PABPC4L | -1.175733281 | 0.000135 | DOWN |
| ENSSSCG00000007033 | AP3M2 | -1.302573951 | 0.000141 | DOWN |
| ENSSSCG00000032156 | GPR146 | -2.203942208 | 0.000156 | DOWN |
| ENSSSCG00000005315 | CA9 | -1.02099413 | 0.00016 | DOWN |
| ENSSSCG00000001624 | FRS3 | -1.124279903 | 0.000163 | DOWN |
| ENSSSCG00000015873 | ACVR1C | -3.583264506 | 0.000182 | DOWN |
| ENSSSCG00000015756 | XKR5 | -1.06105273 | 0.00019 | DOWN |
| ENSSSCG00000002935 | ZNF568 | -1.243874861 | 0.000197 | DOWN |
| ENSSSCG00000008874 | TMEM144 | -1.015269361 | 0.000227 | DOWN |
| ENSSSCG00000016617 | WNT16 | -1.45504501 | 0.000258 | DOWN |
| ENSSSCG00000024403 | PRRT1 | -1.271774997 | 0.000268 | DOWN |
| ENSSSCG00000040228 | NA | -1.559589692 | 0.000276 | DOWN |
| ENSSSCG00000038886 | CAGE1 | -1.566522565 | 0.000305 | DOWN |
| ENSSSCG00000002777 | HSD11B2 | -1.51792927 | 0.000329 | DOWN |
| ENSSSCG00000023225 | SYN2 | -1.514836075 | 0.000393 | DOWN |
| ENSSSCG00000026422 | GPER1 | -1.06871793 | 0.000404 | DOWN |
| ENSSSCG00000007022 | ANK1 | -1.871223725 | 0.000412 | DOWN |
| ENSSSCG00000035798 | PRTG | -1.10481392 | 0.00043 | DOWN |
| ENSSSCG00000011167 | ZNF782 | -1.09711604 | 0.000468 | DOWN |
| ENSSSCG00000033103 | NA | -3.995481132 | 0.00049 | DOWN |
| ENSSSCG00000040698 | PRR7 | -1.782195345 | 0.000493 | DOWN |
| ENSSSCG00000015923 | NOSTRIN | -2.082005156 | 0.000502 | DOWN |
| ENSSSCG00000012352 | KLF8 | -1.103611718 | 0.000524 | DOWN |
| ENSSSCG00000037823 | ANKRD34A | -3.397814206 | 0.000528 | DOWN |
| ENSSSCG00000008702 | DOK7 | -2.632468848 | 0.000529 | DOWN |
| ENSSSCG00000014095 | ZBED3 | -1.44114238 | 0.000568 | DOWN |
| ENSSSCG00000017750 | EVI2B | -1.26657806 | 0.000568 | DOWN |
| ENSSSCG00000017036 | CCNJL | -4.065927778 | 0.000579 | DOWN |
| ENSSSCG00000000203 | KCNH3 | -1.854447241 | 0.0006 | DOWN |
| ENSSSCG00000007350 | PPP1R16B | -1.067331802 | 0.000622 | DOWN |
| ENSSSCG00000031713 | H2AFY2 | -1.034874941 | 0.000639 | DOWN |
| ENSSSCG00000018029 | NA | -1.522848273 | 0.000652 | DOWN |
| ENSSSCG00000036363 | PDE4C | -1.3630131 | 0.000655 | DOWN |
| ENSSSCG00000030209 | MFNG | -4.026493903 | 0.000664 | DOWN |
| ENSSSCG00000013718 | NA | -1.857328258 | 0.000684 | DOWN |
| ENSSSCG00000015035 | C11orf52 | -2.985870545 | 0.000692 | DOWN |
| ENSSSCG00000009457 | DACH1 | -2.938457645 | 0.000766 | DOWN |
| ENSSSCG00000013023 | RASGRP2 | -1.788563299 | 0.000769 | DOWN |
| ENSSSCG00000035739 | SLC26A1 | -2.013023809 | 0.000789 | DOWN |
| ENSSSCG00000033541 | NAT8L | -2.42121559 | 0.000872 | DOWN |
| ENSSSCG00000033816 | NA | -1.0195173 | 0.000884 | DOWN |
| ENSSSCG00000040798 | NA | -1.033452304 | 0.000898 | DOWN |
| ENSSSCG00000035388 | C16orf46 | -1.715839584 | 0.000908 | DOWN |
| ENSSSCG00000031915 | ZFP82 | -1.253795166 | 0.000966 | DOWN |
| ENSSSCG00000012652 | SASH3 | -2.134812955 | 0.000972 | DOWN |
| ENSSSCG00000031977 | PTCH2 | -1.895966678 | 0.001034 | DOWN |
| ENSSSCG00000038013 | DIRAS1 | -1.529197617 | 0.001094 | DOWN |
| ENSSSCG00000039374 | NA | -1.572324262 | 0.001159 | DOWN |
| ENSSSCG00000028341 | NA | -3.232146138 | 0.001161 | DOWN |
| ENSSSCG00000039062 | NA | -1.544885299 | 0.001221 | DOWN |
| ENSSSCG00000010561 | TLX1 | -1.463452876 | 0.001241 | DOWN |
| ENSSSCG00000012768 | ZFP92 | -1.710199432 | 0.001291 | DOWN |
| ENSSSCG00000006531 | NA | -2.906591959 | 0.001311 | DOWN |
| ENSSSCG00000040339 | ZFHX2 | -1.234219096 | 0.001431 | DOWN |
| ENSSSCG00000010303 | SYNPO2L | -1.008218828 | 0.001441 | DOWN |
| ENSSSCG00000021941 | NA | -2.942127485 | 0.001511 | DOWN |
| ENSSSCG00000029257 | NA | -1.419220589 | 0.001543 | DOWN |
| ENSSSCG00000034181 | NKX3-2 | -1.619141973 | 0.001661 | DOWN |
| ENSSSCG00000039259 | NA | -1.036666242 | 0.001668 | DOWN |
| ENSSSCG00000009000 | NA | -1.362031649 | 0.001733 | DOWN |
| ENSSSCG00000003069 | KCNN4 | -1.010202991 | 0.001801 | DOWN |
| ENSSSCG00000003989 | NA | -1.022121444 | 0.001814 | DOWN |
| ENSSSCG00000006038 | ZFPM2 | -1.646354966 | 0.001821 | DOWN |
| ENSSSCG00000021218 | TDRD6 | -2.259799388 | 0.001833 | DOWN |
| ENSSSCG00000011721 | P2RY1 | -1.388600009 | 0.001855 | DOWN |
| ENSSSCG00000016608 | IQUB | -1.807953487 | 0.00189 | DOWN |
| ENSSSCG00000026130 | EPHA3 | -1.005126853 | 0.001943 | DOWN |
| ENSSSCG00000037987 | MS4A10 | -1.433357399 | 0.002 | DOWN |
| ENSSSCG00000015010 | EXPH5 | -1.050730573 | 0.002071 | DOWN |
| ENSSSCG00000032698 | TPPP | -4.13124854 | 0.00236 | DOWN |
| ENSSSCG00000004898 | TNFRSF11A | -2.138381346 | 0.002361 | DOWN |
| ENSSSCG00000036098 | NKX6-2 | -2.065901774 | 0.00237 | DOWN |
| ENSSSCG00000001888 | RPP25 | -1.052945283 | 0.002375 | DOWN |
| ENSSSCG00000040419 | NA | -1.341721578 | 0.002395 | DOWN |
| ENSSSCG00000002268 | AKAP5 | -1.271009148 | 0.002456 | DOWN |
| ENSSSCG00000016861 | C6 | -4.390420931 | 0.002553 | DOWN |
| ENSSSCG00000008120 | NA | -1.328663923 | 0.002583 | DOWN |
| ENSSSCG00000033997 | NA | -1.773164754 | 0.00263 | DOWN |
| ENSSSCG00000038580 | NA | -1.074034929 | 0.002749 | DOWN |
| ENSSSCG00000036831 | EFNA4 | -3.578258988 | 0.002836 | DOWN |
| ENSSSCG00000026618 | CAVIN2 | -1.060180398 | 0.00284 | DOWN |
| ENSSSCG00000031963 | SAXO2 | -1.160495877 | 0.002843 | DOWN |
| ENSSSCG00000006396 | IGSF9 | -1.813932646 | 0.00291 | DOWN |
| ENSSSCG00000032522 | NA | -1.114187359 | 0.002984 | DOWN |
| ENSSSCG00000002507 | BCL11B | -1.762807328 | 0.002994 | DOWN |
| ENSSSCG00000011744 | NA | -1.841850278 | 0.003079 | DOWN |
| ENSSSCG00000012658 | RAB33A | -1.538334611 | 0.003082 | DOWN |
| ENSSSCG00000037567 | NA | -1.154895974 | 0.003106 | DOWN |
| ENSSSCG00000007139 | ADRA1D | -1.77419228 | 0.003165 | DOWN |
| ENSSSCG00000001523 | GRM4 | -3.584390462 | 0.003235 | DOWN |
| ENSSSCG00000033305 | PGBD2 | -1.004349609 | 0.003247 | DOWN |
| ENSSSCG00000000906 | TMCC3 | -1.340891631 | 0.003298 | DOWN |
| ENSSSCG00000035810 | NA | -1.175567082 | 0.003311 | DOWN |
| ENSSSCG00000006876 | PLPPR5 | -2.373394965 | 0.003374 | DOWN |
| ENSSSCG00000027688 | RNF183 | -4.194684112 | 0.00351 | DOWN |
| ENSSSCG00000000279 | HOXC13 | -3.00920431 | 0.00364 | DOWN |
| ENSSSCG00000001434 | EGFL8 | -1.142763019 | 0.00366 | DOWN |
| ENSSSCG00000011768 | CCDC39 | -1.751773023 | 0.003689 | DOWN |
| ENSSSCG00000032063 | THEM6 | -1.099459733 | 0.003766 | DOWN |
| ENSSSCG00000032249 | GBX1 | -1.49549366 | 0.003902 | DOWN |
| ENSSSCG00000033009 | NA | -1.550130982 | 0.003985 | DOWN |
| ENSSSCG00000006890 | ABCA4 | -2.355241693 | 0.004128 | DOWN |
| ENSSSCG00000035720 | HRCT1 | -3.459633136 | 0.004315 | DOWN |
| ENSSSCG00000013145 | DTX4 | -1.329273462 | 0.004458 | DOWN |
| ENSSSCG00000034987 | IQANK1 | -1.345977901 | 0.004714 | DOWN |
| ENSSSCG00000011911 | DRD3 | -1.351906447 | 0.004974 | DOWN |
| ENSSSCG00000040446 | NA | -1.433618844 | 0.005145 | DOWN |
| ENSSSCG00000037995 | NA | -1.737135523 | 0.005296 | DOWN |
| ENSSSCG00000016705 | HOXA3 | -1.45615241 | 0.005417 | DOWN |
| ENSSSCG00000007305 | SPAG4 | -1.974169037 | 0.005529 | DOWN |
| ENSSSCG00000022175 | MYH15 | -1.337009046 | 0.005679 | DOWN |
| ENSSSCG00000010761 | STK32C | -1.402985327 | 0.006122 | DOWN |
| ENSSSCG00000023084 | ATP2B2 | -2.275474269 | 0.006365 | DOWN |
| ENSSSCG00000003240 | NA | -1.048169118 | 0.006371 | DOWN |
| ENSSSCG00000023156 | NA | -1.225643788 | 0.006535 | DOWN |
| ENSSSCG00000011333 | NA | -2.583786435 | 0.006656 | DOWN |
| ENSSSCG00000039392 | SNPH | -1.538350072 | 0.007001 | DOWN |
| ENSSSCG00000000277 | NPFF | -2.269356554 | 0.007237 | DOWN |
| ENSSSCG00000039413 | SLC9A3 | -1.436916704 | 0.007239 | DOWN |
| ENSSSCG00000008396 | CCDC85A | -1.489610372 | 0.007646 | DOWN |
| ENSSSCG00000017738 | ADAP2 | -1.636349623 | 0.007921 | DOWN |
| ENSSSCG00000007146 | SIGLEC1 | -2.72863608 | 0.008137 | DOWN |
| ENSSSCG00000034921 | CLDN19 | -1.420199875 | 0.00832 | DOWN |
| ENSSSCG00000011695 | AGTR1 | -1.411328878 | 0.008502 | DOWN |
| ENSSSCG00000031537 | HAND2 | -1.267373735 | 0.008586 | DOWN |
| ENSSSCG00000005369 | FOXE1 | -1.389723313 | 0.00863 | DOWN |
| ENSSSCG00000040031 | SMIM32 | -1.145441397 | 0.00866 | DOWN |
| ENSSSCG00000035711 | NA | -2.215878538 | 0.008671 | DOWN |
| ENSSSCG00000020934 | NA | -1.123711244 | 0.008719 | DOWN |
| ENSSSCG00000024290 | NA | -1.82665619 | 0.00884 | DOWN |
| ENSSSCG00000010543 | ABCC2 | -2.269117324 | 0.009364 | DOWN |
| ENSSSCG00000037494 | SPTBN4 | -1.524029255 | 0.009479 | DOWN |
| ENSSSCG00000009953 | NA | -2.042773568 | 0.009554 | DOWN |
| ENSSSCG00000013273 | CHST1 | -2.774953117 | 0.009731 | DOWN |
| ENSSSCG00000016777 | NA | -1.074207522 | 0.009881 | DOWN |
| ENSSSCG00000000443 | GLI1 | -1.39297649 | 0.009951 | DOWN |
| ENSSSCG00000016838 | RANBP3L | -1.473199792 | 0.010138 | DOWN |
| ENSSSCG00000026417 | EPS8L1 | -1.852106364 | 0.010189 | DOWN |
| ENSSSCG00000021479 | RGS9BP | -2.683668181 | 0.01021 | DOWN |
| ENSSSCG00000032943 | uc_338 | -1.231159466 | 0.010318 | DOWN |
| ENSSSCG00000022728 | HOXC10 | -1.016726469 | 0.010441 | DOWN |
| ENSSSCG00000005452 | C9orf152 | -4.079798541 | 0.010777 | DOWN |
| ENSSSCG00000020300 | U6 | -2.250790043 | 0.01082 | DOWN |
| ENSSSCG00000021601 | ZNF711 | -2.441917333 | 0.010931 | DOWN |
| ENSSSCG00000040018 | NA | -1.161185875 | 0.011022 | DOWN |
| ENSSSCG00000012492 | DRP2 | -1.520054543 | 0.011322 | DOWN |
| ENSSSCG00000019456 | ssc-mir-23b | -2.002944049 | 0.011392 | DOWN |
| ENSSSCG00000004746 | ITPKA | -1.476712745 | 0.011431 | DOWN |
| ENSSSCG00000022462 | ZNF852 | -1.022026717 | 0.01149 | DOWN |
| ENSSSCG00000038278 | NTSR1 | -1.356450576 | 0.011592 | DOWN |
| ENSSSCG00000034997 | NA | -2.212827272 | 0.011621 | DOWN |
| ENSSSCG00000010086 | NA | -1.955522079 | 0.011648 | DOWN |
| ENSSSCG00000015707 | GPR39 | -3.837755377 | 0.012258 | DOWN |
| ENSSSCG00000001629 | USP49 | -1.154158269 | 0.012309 | DOWN |
| ENSSSCG00000034990 | NA | -1.005099646 | 0.012319 | DOWN |
| ENSSSCG00000031976 | ANKRD53 | -1.25209602 | 0.012371 | DOWN |
| ENSSSCG00000038970 | BCL2L15 | -1.106077076 | 0.012695 | DOWN |
| ENSSSCG00000009896 | BICDL1 | -1.059893641 | 0.012826 | DOWN |
| ENSSSCG00000004167 | MYB | -1.405420988 | 0.012959 | DOWN |
| ENSSSCG00000040837 | NA | -1.163281216 | 0.013318 | DOWN |
| ENSSSCG00000015700 | TMEM163 | -2.101245292 | 0.013395 | DOWN |
| ENSSSCG00000013461 | PEAK3 | -2.69597652 | 0.013421 | DOWN |
| ENSSSCG00000010603 | NEURL1 | -1.826833853 | 0.013536 | DOWN |
| ENSSSCG00000007191 | RAD21L1 | -2.380841538 | 0.013672 | DOWN |
| ENSSSCG00000037405 | SNORD36 | -1.719787492 | 0.014082 | DOWN |
| ENSSSCG00000023258 | CLEC4F | -1.561809256 | 0.014085 | DOWN |
| ENSSSCG00000031634 | NA | -1.459398864 | 0.014794 | DOWN |
| ENSSSCG00000034776 | FAM19A5 | -1.874324897 | 0.014885 | DOWN |
| ENSSSCG00000038932 | PPM1E | -1.249939287 | 0.014966 | DOWN |
| ENSSSCG00000000634 | STYK1 | -2.053186587 | 0.015048 | DOWN |
| ENSSSCG00000022988 | TSPEAR | -1.890588518 | 0.015077 | DOWN |
| ENSSSCG00000035897 | NA | -3.034197208 | 0.015286 | DOWN |
| ENSSSCG00000013106 | PTGDR2 | -1.283181462 | 0.01545 | DOWN |
| ENSSSCG00000040134 | TNFRSF13B | -2.055908403 | 0.015467 | DOWN |
| ENSSSCG00000012415 | NAP1L2 | -1.734111361 | 0.015651 | DOWN |
| ENSSSCG00000033313 | C7orf31 | -1.932223376 | 0.016095 | DOWN |
| ENSSSCG00000040393 | DCAF16 | -2.512648152 | 0.016362 | DOWN |
| ENSSSCG00000033908 | SMIM2 | -2.506282715 | 0.017832 | DOWN |
| ENSSSCG00000035751 | NA | -1.887971196 | 0.018138 | DOWN |
| ENSSSCG00000029752 | C16orf54 | -1.917690089 | 0.019496 | DOWN |
| ENSSSCG00000037835 | TRIM7 | -1.356942977 | 0.019572 | DOWN |
| ENSSSCG00000006764 | PTPN22 | -1.323855988 | 0.019685 | DOWN |
| ENSSSCG00000003049 | NA | -1.141430413 | 0.020172 | DOWN |
| ENSSSCG00000019211 | NA | -1.540582398 | 0.021376 | DOWN |
| ENSSSCG00000005475 | ZFP37 | -1.219594796 | 0.021962 | DOWN |
| ENSSSCG00000034696 | NA | -1.360883179 | 0.02197 | DOWN |
| ENSSSCG00000040219 | NA | -1.292857954 | 0.02202 | DOWN |
| ENSSSCG00000009220 | DMP1 | -1.732238724 | 0.022427 | DOWN |
| ENSSSCG00000031550 | NA | -1.818239212 | 0.022516 | DOWN |
| ENSSSCG00000013231 | C1QTNF4 | -1.513514161 | 0.022768 | DOWN |
| ENSSSCG00000006812 | ALX3 | -2.359004521 | 0.023124 | DOWN |
| ENSSSCG00000003539 | GRHL3 | -1.112103662 | 0.023139 | DOWN |
| ENSSSCG00000036209 | HOXA11-AS1_6 | -1.453976359 | 0.023234 | DOWN |
| ENSSSCG00000028562 | NA | -1.331740111 | 0.023316 | DOWN |
| ENSSSCG00000021651 | SCN2B | -3.371386115 | 0.02432 | DOWN |
| ENSSSCG00000032048 | SCRG1 | -3.132509291 | 0.024744 | DOWN |
| ENSSSCG00000038126 | MGAT3 | -1.051734813 | 0.024941 | DOWN |
| ENSSSCG00000005480 | NA | -2.114425182 | 0.02531 | DOWN |
| ENSSSCG00000033628 | NA | -1.265942906 | 0.026281 | DOWN |
| ENSSSCG00000002866 | CEBPA | -1.007338438 | 0.026347 | DOWN |
| ENSSSCG00000028784 | NA | -1.656475795 | 0.026777 | DOWN |
| ENSSSCG00000008392 | BCL11A | -1.854271727 | 0.027042 | DOWN |
| ENSSSCG00000021453 | RIMS4 | -1.154995702 | 0.027583 | DOWN |
| ENSSSCG00000000730 | PRMT8 | -1.218772628 | 0.027643 | DOWN |
| ENSSSCG00000016941 | RNF180 | -1.526129144 | 0.028079 | DOWN |
| ENSSSCG00000000991 | FOXQ1 | -1.252385693 | 0.029089 | DOWN |
| ENSSSCG00000032827 | NA | -1.448852949 | 0.029363 | DOWN |
| ENSSSCG00000016391 | AMER3 | -1.960482762 | 0.0299 | DOWN |
| ENSSSCG00000019141 | ssc-mir-365-1 | -2.465649018 | 0.029921 | DOWN |
| ENSSSCG00000039283 | SIX3 | -1.85304412 | 0.030006 | DOWN |
| ENSSSCG00000030843 | APLN | -1.865709434 | 0.030026 | DOWN |
| ENSSSCG00000033189 | FAM107A | -1.910336432 | 0.030473 | DOWN |
| ENSSSCG00000007424 | TNNC2 | -2.465816027 | 0.032877 | DOWN |
| ENSSSCG00000001247 | ZFP57 | -1.148882004 | 0.032911 | DOWN |
| ENSSSCG00000018699 | SNORA38 | -1.71178882 | 0.033351 | DOWN |
| ENSSSCG00000002778 | ZDHHC1 | -1.20475543 | 0.033426 | DOWN |
| ENSSSCG00000034609 | RNF112 | -1.865597762 | 0.033816 | DOWN |
| ENSSSCG00000032735 | INAVA | -1.27695181 | 0.033984 | DOWN |
| ENSSSCG00000021068 | TRAF5 | -1.800648543 | 0.036313 | DOWN |
| ENSSSCG00000012332 | WNK3 | -1.209637001 | 0.036471 | DOWN |
| ENSSSCG00000005216 | NA | -1.496265554 | 0.036475 | DOWN |
| ENSSSCG00000035924 | SHISA7 | -1.734331053 | 0.036513 | DOWN |
| ENSSSCG00000017223 | USH1G | -3.215296829 | 0.036775 | DOWN |
| ENSSSCG00000029392 | HUNK | -1.175878917 | 0.036873 | DOWN |
| ENSSSCG00000004776 | DISP2 | -1.084859092 | 0.03797 | DOWN |
| ENSSSCG00000026718 | PLCH1 | -2.418046743 | 0.039113 | DOWN |
| ENSSSCG00000005182 | CCDC171 | -1.141391595 | 0.039327 | DOWN |
| ENSSSCG00000016166 | NA | -2.021916588 | 0.039422 | DOWN |
| ENSSSCG00000039990 | NA | -1.635784988 | 0.040328 | DOWN |
| ENSSSCG00000027179 | SIM2 | -1.273006109 | 0.041143 | DOWN |
| ENSSSCG00000012349 | USP51 | -1.284494669 | 0.041201 | DOWN |
| ENSSSCG00000034881 | NA | -1.609149328 | 0.041202 | DOWN |
| ENSSSCG00000002512 | DEGS2 | -1.667665675 | 0.042035 | DOWN |
| ENSSSCG00000029886 | LYVE1 | -1.618444212 | 0.043061 | DOWN |
| ENSSSCG00000000113 | SLC16A8 | -1.034903468 | 0.044272 | DOWN |
| ENSSSCG00000003071 | ZNF404 | -1.084870843 | 0.04439 | DOWN |
| ENSSSCG00000016600 | TMEM229A | -2.601812577 | 0.044825 | DOWN |
| ENSSSCG00000036367 | PABPN1L | -2.992529352 | 0.045857 | DOWN |
| ENSSSCG00000035629 | CA5A | -1.481453091 | 0.046981 | DOWN |
| ENSSSCG00000030368 | HSPA1L | -1.061166597 | 0.046994 | DOWN |
| ENSSSCG00000026579 | NA | -1.628841989 | 0.04906 | DOWN |

Gene Name “NA” indicates the gene ID was not matched to a HGNC gene name.
