# Supplementary material for: A Tale of Two Loads: Modulation of IL-1 Induced Inflammatory Responses of Meniscal Cells in Two Models of Dynamic Physiologic Loading
Source: Front Bioeng Biotechnol. 2022 Mar 1;10:837619. doi: 10.3389/fbioe.2022.837619 (PMC8921261; doi:10.3389/fbioe.2022.837619)
Supplement: Supplementary file 7 [file DataSheet14.DOCX]

**Supplemental Table 15**: Genes with a significant (p<0.05) interaction effect between load (5% cyclic tensile stretch) and IL-1α treatment, outer zone cells.

| **Gene ID** | **Gene Name** | **p-value** |
| --- | --- | --- |
| ENSSSCG00000031380 | NA | 3.44E-62 |
| ENSSSCG00000027426 | BCL3 | 2.21E-54 |
| ENSSSCG00000017298 | TANC2 | 2.89E-42 |
| ENSSSCG00000004657 | CEP152 | 4.47E-39 |
| ENSSSCG00000000411 | NAB2 | 7.04E-36 |
| ENSSSCG00000008957 | AMCF-II | 1.52E-33 |
| ENSSSCG00000040887 | PAPD5 | 1.71E-32 |
| ENSSSCG00000016857 | DAB2 | 3.43E-31 |
| ENSSSCG00000010329 | ZMIZ1 | 6.9E-30 |
| ENSSSCG00000004830 | ATP10A | 1.75E-29 |
| ENSSSCG00000011020 | JCAD | 1.84E-29 |
| ENSSSCG00000034191 | SOX6 | 1.91E-29 |
| ENSSSCG00000017420 | CNP | 6.33E-29 |
| ENSSSCG00000017046 | EBF1 | 1.47E-28 |
| ENSSSCG00000016438 | NUB1 | 1.95E-26 |
| ENSSSCG00000032360 | PANX1 | 5.76E-26 |
| ENSSSCG00000002516 | WARS | 1.38E-25 |
| ENSSSCG00000012890 | TCIRG1 | 3.06E-25 |
| ENSSSCG00000012026 | ADAMTS1 | 2.71E-24 |
| ENSSSCG00000023522 | TGM2 | 8.36E-24 |
| ENSSSCG00000021646 | KLF9 | 1.4E-23 |
| ENSSSCG00000015014 | ZC3H12C | 1.86E-23 |
| ENSSSCG00000001667 | ZNF318 | 1.06E-22 |
| ENSSSCG00000025593 | NA | 1.65E-22 |
| ENSSSCG00000009111 | SYNPO2 | 7.93E-22 |
| ENSSSCG00000040719 | KIAA0040 | 1.55E-21 |
| ENSSSCG00000027806 | SAMHD1 | 2.32E-21 |
| ENSSSCG00000034879 | MAML2 | 9.24E-21 |
| ENSSSCG00000031201 | LMOD1 | 9.24E-21 |
| ENSSSCG00000015525 | TOR3A | 9.88E-20 |
| ENSSSCG00000000396 | STAT2 | 1E-19 |
| ENSSSCG00000011212 | RARB | 1.97E-19 |
| ENSSSCG00000013382 | PLEKHA7 | 2.35E-19 |
| ENSSSCG00000037536 | SLC25A28 | 5.75E-19 |
| ENSSSCG00000001233 | TRIM26 | 7.06E-19 |
| ENSSSCG00000004464 | FAM46A | 9.48E-19 |
| ENSSSCG00000023298 | SRXN1 | 1.21E-18 |
| ENSSSCG00000015784 | ACSL1 | 2.55E-18 |
| ENSSSCG00000036679 | SORBS2 | 3.69E-18 |
| ENSSSCG00000004421 | FYN | 1.49E-17 |
| ENSSSCG00000037815 | ZC3H12A | 1.88E-17 |
| ENSSSCG00000007874 | NA | 4.3E-17 |
| ENSSSCG00000009968 | TTC28 | 7.28E-17 |
| ENSSSCG00000024219 | TIGAR | 7.79E-17 |
| ENSSSCG00000007079 | FLRT3 | 7.86E-17 |
| ENSSSCG00000026466 | SLC23A2 | 7.94E-17 |
| ENSSSCG00000008959 | CXCL2 | 1.28E-16 |
| ENSSSCG00000016512 | ZC3HAV1 | 1.28E-16 |
| ENSSSCG00000030042 | SBNO2 | 1.62E-16 |
| ENSSSCG00000004053 | TAGAP | 1.67E-16 |
| ENSSSCG00000034207 | CEBPB | 1.76E-16 |
| ENSSSCG00000007554 | ZFAND2A | 2.37E-16 |
| ENSSSCG00000010261 | PPA1 | 2.62E-16 |
| ENSSSCG00000010540 | ENTPD7 | 2.76E-16 |
| ENSSSCG00000028536 | LHFPL2 | 3.93E-16 |
| ENSSSCG00000035153 | TRIM38 | 4.03E-16 |
| ENSSSCG00000007073 | ISM1 | 4.3E-16 |
| ENSSSCG00000021383 | CGAS | 4.37E-16 |
| ENSSSCG00000010464 | PPP1R3C | 7.88E-16 |
| ENSSSCG00000020666 | EHD4 | 8.26E-16 |
| ENSSSCG00000040786 | SPTBN1 | 8.26E-16 |
| ENSSSCG00000036274 | NA | 9.3E-16 |
| ENSSSCG00000016758 | NA | 9.54E-16 |
| ENSSSCG00000032154 | ERF | 1.53E-15 |
| ENSSSCG00000007067 | JAG1 | 1.54E-15 |
| ENSSSCG00000008727 | MSX1 | 2.6E-15 |
| ENSSSCG00000014924 | CTSC | 2.81E-15 |
| ENSSSCG00000035479 | DISP1 | 3.03E-15 |
| ENSSSCG00000011495 | PRICKLE2 | 5.21E-15 |
| ENSSSCG00000010996 | BAG1 | 8.22E-15 |
| ENSSSCG00000005208 | RIC1 | 8.22E-15 |
| ENSSSCG00000033613 | FOXS1 | 8.22E-15 |
| ENSSSCG00000026454 | NA | 1.19E-14 |
| ENSSSCG00000005027 | FRMD6 | 1.6E-14 |
| ENSSSCG00000017087 | GM2A | 1.63E-14 |
| ENSSSCG00000006127 | NBN | 1.77E-14 |
| ENSSSCG00000001661 | SRF | 1.91E-14 |
| ENSSSCG00000008437 | SOCS5 | 2.49E-14 |
| ENSSSCG00000013432 | MIDN | 2.49E-14 |
| ENSSSCG00000008123 | ARID5A | 2.53E-14 |
| ENSSSCG00000006923 | GBP2 | 2.73E-14 |
| ENSSSCG00000032367 | CEBPD | 3.04E-14 |
| ENSSSCG00000003379 | KLHL21 | 3.19E-14 |
| ENSSSCG00000014909 | NA | 3.91E-14 |
| ENSSSCG00000009370 | FOXO1 | 6.02E-14 |
| ENSSSCG00000032408 | CASP7 | 6.02E-14 |
| ENSSSCG00000005308 | RUSC2 | 6.04E-14 |
| ENSSSCG00000003471 | EPHA2 | 8.22E-14 |
| ENSSSCG00000013366 | LDHA | 1.05E-13 |
| ENSSSCG00000003590 | PTPRU | 1.22E-13 |
| ENSSSCG00000022101 | BRCA1 | 1.22E-13 |
| ENSSSCG00000005364 | TDRD7 | 1.72E-13 |
| ENSSSCG00000006776 | MOV10 | 3.03E-13 |
| ENSSSCG00000005983 | ATAD2 | 3.9E-13 |
| ENSSSCG00000014149 | MEF2C | 4.53E-13 |
| ENSSSCG00000039780 | RTN4RL1 | 4.53E-13 |
| ENSSSCG00000036893 | PTHLH | 4.54E-13 |
| ENSSSCG00000039947 | KCNJ2 | 8.22E-13 |
| ENSSSCG00000016502 | PARP12 | 1.01E-12 |
| ENSSSCG00000001229 | NA | 1.27E-12 |
| ENSSSCG00000010219 | ARID5B | 1.98E-12 |
| ENSSSCG00000023716 | TNFAIP6 | 2.6E-12 |
| ENSSSCG00000038491 | MEX3B | 4.22E-12 |
| ENSSSCG00000001394 | NA | 4.52E-12 |
| ENSSSCG00000039419 | SLCO4A1 | 4.86E-12 |
| ENSSSCG00000006917 | NA | 6.41E-12 |
| ENSSSCG00000011859 | HEG1 | 1.05E-11 |
| ENSSSCG00000032936 | PIM3 | 1.16E-11 |
| ENSSSCG00000007458 | NCOA3 | 1.16E-11 |
| ENSSSCG00000015375 | ITGB8 | 1.2E-11 |
| ENSSSCG00000038854 | PSMF1 | 1.21E-11 |
| ENSSSCG00000030996 | NA | 1.3E-11 |
| ENSSSCG00000034989 | LRRTM2 | 1.55E-11 |
| ENSSSCG00000031023 | NA | 1.64E-11 |
| ENSSSCG00000015770 | VEGFC | 1.64E-11 |
| ENSSSCG00000006235 | TOX | 2.05E-11 |
| ENSSSCG00000013147 | FAM111B | 2.08E-11 |
| ENSSSCG00000025826 | BOC | 2.18E-11 |
| ENSSSCG00000033222 | TRIM14 | 2.22E-11 |
| ENSSSCG00000000810 | AMIGO2 | 2.28E-11 |
| ENSSSCG00000004971 | TLE3 | 2.58E-11 |
| ENSSSCG00000039473 | NA | 3.06E-11 |
| ENSSSCG00000040184 | LMO7 | 3.06E-11 |
| ENSSSCG00000023907 | AFAP1 | 3.27E-11 |
| ENSSSCG00000004192 | CTGF | 3.68E-11 |
| ENSSSCG00000013599 | ANGPTL4 | 3.69E-11 |
| ENSSSCG00000005385 | NR4A3 | 4.78E-11 |
| ENSSSCG00000011465 | NA | 4.89E-11 |
| ENSSSCG00000023972 | DRAM1 | 5.44E-11 |
| ENSSSCG00000003579 | AHDC1 | 5.49E-11 |
| ENSSSCG00000017705 | CCL5 | 6.89E-11 |
| ENSSSCG00000003000 | ITPKC | 7.35E-11 |
| ENSSSCG00000023400 | ZNF598 | 7.76E-11 |
| ENSSSCG00000032251 | MAFB | 9.24E-11 |
| ENSSSCG00000026951 | PSMB8 | 9.24E-11 |
| ENSSSCG00000022447 | F3 | 9.6E-11 |
| ENSSSCG00000009192 | PDLIM5 | 1.01E-10 |
| ENSSSCG00000035078 | CD40 | 1.1E-10 |
| ENSSSCG00000021597 | PHLDA2 | 1.27E-10 |
| ENSSSCG00000039194 | KANK2 | 1.61E-10 |
| ENSSSCG00000037159 | MNT | 1.85E-10 |
| ENSSSCG00000001835 | ABHD2 | 1.96E-10 |
| ENSSSCG00000038185 | EREG | 1.96E-10 |
| ENSSSCG00000014565 | NA | 2.31E-10 |
| ENSSSCG00000001516 | BAK1 | 2.87E-10 |
| ENSSSCG00000035181 | RNF24 | 3.28E-10 |
| ENSSSCG00000006209 | VCPIP1 | 3.33E-10 |
| ENSSSCG00000022011 | NMI | 4.13E-10 |
| ENSSSCG00000032835 | TMEM268 | 5.04E-10 |
| ENSSSCG00000014171 | ERAP1 | 5.1E-10 |
| ENSSSCG00000005437 | KLF4 | 5.66E-10 |
| ENSSSCG00000026041 | MAP3K5 | 5.66E-10 |
| ENSSSCG00000009320 | FLT1 | 5.96E-10 |
| ENSSSCG00000031492 | PPP1R18 | 7.08E-10 |
| ENSSSCG00000011463 | IL17RD | 7.19E-10 |
| ENSSSCG00000001509 | DAXX | 9.14E-10 |
| ENSSSCG00000011672 | RASA2 | 9.66E-10 |
| ENSSSCG00000029030 | SMCHD1 | 9.81E-10 |
| ENSSSCG00000009053 | RNF150 | 1.03E-09 |
| ENSSSCG00000033321 | GAS1 | 1.28E-09 |
| ENSSSCG00000006066 | RNF19A | 1.35E-09 |
| ENSSSCG00000014820 | FCHSD2 | 1.35E-09 |
| ENSSSCG00000027340 | NA | 1.45E-09 |
| ENSSSCG00000038521 | CHAC1 | 1.53E-09 |
| ENSSSCG00000011474 | PXK | 1.6E-09 |
| ENSSSCG00000006371 | USF1 | 1.96E-09 |
| ENSSSCG00000025618 | TAP1 | 2.13E-09 |
| ENSSSCG00000031118 | PREX1 | 2.17E-09 |
| ENSSSCG00000028606 | ZBTB7B | 2.19E-09 |
| ENSSSCG00000016262 | NA | 2.5E-09 |
| ENSSSCG00000022312 | RHPN2 | 2.56E-09 |
| ENSSSCG00000005012 | ARF6 | 2.56E-09 |
| ENSSSCG00000010698 | FGFR2 | 2.9E-09 |
| ENSSSCG00000004371 | CRYBG1 | 3.07E-09 |
| ENSSSCG00000033509 | SAMD11 | 3.11E-09 |
| ENSSSCG00000017754 | NA | 3.31E-09 |
| ENSSSCG00000030560 | NA | 3.36E-09 |
| ENSSSCG00000012050 | RCAN1 | 3.81E-09 |
| ENSSSCG00000001398 | NA | 4.45E-09 |
| ENSSSCG00000035634 | NA | 4.76E-09 |
| ENSSSCG00000032622 | PPP3CC | 4.84E-09 |
| ENSSSCG00000015299 | STEAP4 | 4.96E-09 |
| ENSSSCG00000005423 | ABCA1 | 5.28E-09 |
| ENSSSCG00000017301 | TLK2 | 5.73E-09 |
| ENSSSCG00000012258 | FUNDC1 | 6.01E-09 |
| ENSSSCG00000025206 | RNF19B | 6.18E-09 |
| ENSSSCG00000035790 | BTG1 | 6.63E-09 |
| ENSSSCG00000009048 | GAB1 | 7.69E-09 |
| ENSSSCG00000011390 | IP6K1 | 7.73E-09 |
| ENSSSCG00000022714 | OSER1 | 9.09E-09 |
| ENSSSCG00000004789 | THBS1 | 9.88E-09 |
| ENSSSCG00000015782 | IRF2 | 9.89E-09 |
| ENSSSCG00000015545 | GLUL | 1.07E-08 |
| ENSSSCG00000009716 | SH3RF1 | 1.14E-08 |
| ENSSSCG00000040061 | NINJ1 | 1.17E-08 |
| ENSSSCG00000014835 | C2CD3 | 1.22E-08 |
| ENSSSCG00000006331 | PBX1 | 1.32E-08 |
| ENSSSCG00000021822 | RNF169 | 1.38E-08 |
| ENSSSCG00000015871 | NR4A2 | 1.56E-08 |
| ENSSSCG00000011106 | CREM | 1.56E-08 |
| ENSSSCG00000039582 | NA | 2.12E-08 |
| ENSSSCG00000015324 | GNG11 | 2.2E-08 |
| ENSSSCG00000009125 | ANK2 | 2.2E-08 |
| ENSSSCG00000000146 | NA | 2.22E-08 |
| ENSSSCG00000017274 | PITPNC1 | 2.38E-08 |
| ENSSSCG00000008787 | KLHL5 | 3E-08 |
| ENSSSCG00000001507 | TAPBP | 3.2E-08 |
| ENSSSCG00000006073 | OSR2 | 3.24E-08 |
| ENSSSCG00000036634 | MAP4K5 | 3.46E-08 |
| ENSSSCG00000004201 | TMEM200A | 3.75E-08 |
| ENSSSCG00000035791 | SIX5 | 3.75E-08 |
| ENSSSCG00000009018 | SH3D19 | 3.8E-08 |
| ENSSSCG00000004919 | NEDD4L | 3.94E-08 |
| ENSSSCG00000014869 | LRRC32 | 4.19E-08 |
| ENSSSCG00000015820 | NSD3 | 4.83E-08 |
| ENSSSCG00000011950 | NXPE3 | 4.97E-08 |
| ENSSSCG00000001463 | PSMB9 | 5.37E-08 |
| ENSSSCG00000016261 | SP110 | 5.39E-08 |
| ENSSSCG00000013448 | MKNK2 | 5.39E-08 |
| ENSSSCG00000037572 | EPSTI1 | 6.09E-08 |
| ENSSSCG00000022649 | SLC7A11 | 6.09E-08 |
| ENSSSCG00000040162 | NUPR1 | 6.09E-08 |
| ENSSSCG00000035059 | MCM10 | 6.14E-08 |
| ENSSSCG00000005965 | MYC | 6.5E-08 |
| ENSSSCG00000040334 | CBX6 | 8.97E-08 |
| ENSSSCG00000012893 | UNC93B1 | 9.02E-08 |
| ENSSSCG00000009943 | SSH1 | 9.23E-08 |
| ENSSSCG00000004974 | LARP6 | 9.66E-08 |
| ENSSSCG00000010836 | BROX | 9.93E-08 |
| ENSSSCG00000006173 | GDAP1 | 1.08E-07 |
| ENSSSCG00000000455 | LRIG3 | 1.09E-07 |
| ENSSSCG00000032475 | CEP44 | 1.12E-07 |
| ENSSSCG00000029347 | NA | 1.12E-07 |
| ENSSSCG00000014822 | ARHGEF17 | 1.22E-07 |
| ENSSSCG00000011198 | RFTN1 | 1.33E-07 |
| ENSSSCG00000008562 | SLC35F6 | 1.39E-07 |
| ENSSSCG00000012583 | ACSL4 | 1.6E-07 |
| ENSSSCG00000013297 | CD44 | 1.65E-07 |
| ENSSSCG00000027869 | PHF13 | 1.68E-07 |
| ENSSSCG00000015801 | TLR3 | 1.93E-07 |
| ENSSSCG00000035420 | HES4 | 2.02E-07 |
| ENSSSCG00000005683 | TOR1B | 2.1E-07 |
| ENSSSCG00000033786 | NA | 2.32E-07 |
| ENSSSCG00000012828 | STARD8 | 2.43E-07 |
| ENSSSCG00000021586 | ZHX2 | 2.43E-07 |
| ENSSSCG00000006864 | CDC14A | 2.58E-07 |
| ENSSSCG00000027124 | NA | 2.74E-07 |
| ENSSSCG00000027894 | FAM76A | 2.74E-07 |
| ENSSSCG00000027157 | SLC40A1 | 2.74E-07 |
| ENSSSCG00000034645 | C3orf38 | 2.92E-07 |
| ENSSSCG00000002841 | N4BP1 | 2.96E-07 |
| ENSSSCG00000003702 | GATA6 | 3.18E-07 |
| ENSSSCG00000001984 | KHNYN | 3.31E-07 |
| ENSSSCG00000038731 | STX18 | 3.47E-07 |
| ENSSSCG00000037670 | TMEM164 | 3.62E-07 |
| ENSSSCG00000032170 | DAPK3 | 4.13E-07 |
| ENSSSCG00000004572 | NA | 4.13E-07 |
| ENSSSCG00000001620 | MDFI | 4.49E-07 |
| ENSSSCG00000005724 | SETX | 4.8E-07 |
| ENSSSCG00000032709 | ARL4A | 4.86E-07 |
| ENSSSCG00000022492 | AMPD3 | 5.06E-07 |
| ENSSSCG00000008953 | CXCL8 | 5.59E-07 |
| ENSSSCG00000025281 | NA | 5.59E-07 |
| ENSSSCG00000005096 | HIF1A | 5.85E-07 |
| ENSSSCG00000030655 | MAMDC2 | 6.11E-07 |
| ENSSSCG00000010311 | CAMK2G | 6.12E-07 |
| ENSSSCG00000010017 | SMTN | 6.16E-07 |
| ENSSSCG00000032469 | NA | 6.46E-07 |
| ENSSSCG00000012375 | DLG3 | 6.83E-07 |
| ENSSSCG00000013408 | ADM | 7.3E-07 |
| ENSSSCG00000035595 | HMCN1 | 7.3E-07 |
| ENSSSCG00000016053 | NA | 7.73E-07 |
| ENSSSCG00000013114 | SLC15A3 | 7.91E-07 |
| ENSSSCG00000035537 | RUNX1 | 8.06E-07 |
| ENSSSCG00000031882 | PNPT1 | 8.13E-07 |
| ENSSSCG00000012027 | ADAMTS5 | 8.35E-07 |
| ENSSSCG00000031871 | NA | 8.86E-07 |
| ENSSSCG00000015880 | TANC1 | 9.36E-07 |
| ENSSSCG00000011972 | FILIP1L | 9.48E-07 |
| ENSSSCG00000003670 | RLF | 1.02E-06 |
| ENSSSCG00000035240 | GPR63 | 1.11E-06 |
| ENSSSCG00000004205 | ARHGAP18 | 1.21E-06 |
| ENSSSCG00000002259 | NA | 1.27E-06 |
| ENSSSCG00000006733 | TTF2 | 1.28E-06 |
| ENSSSCG00000017146 | RNF213 | 1.28E-06 |
| ENSSSCG00000009216 | SPP1 | 1.29E-06 |
| ENSSSCG00000040208 | PAG1 | 1.31E-06 |
| ENSSSCG00000008467 | EML4 | 1.35E-06 |
| ENSSSCG00000006453 | KIRREL1 | 1.37E-06 |
| ENSSSCG00000032115 | OSGIN1 | 1.41E-06 |
| ENSSSCG00000005041 | FERMT2 | 1.46E-06 |
| ENSSSCG00000012479 | PCDH19 | 1.58E-06 |
| ENSSSCG00000005601 | HSPA5 | 1.62E-06 |
| ENSSSCG00000004165 | PDE7B | 1.62E-06 |
| ENSSSCG00000010146 | LGALS8 | 1.69E-06 |
| ENSSSCG00000021815 | NA | 1.72E-06 |
| ENSSSCG00000040663 | HERPUD1 | 1.8E-06 |
| ENSSSCG00000025343 | ZNF628 | 1.81E-06 |
| ENSSSCG00000030511 | LGR5 | 1.81E-06 |
| ENSSSCG00000004082 | NA | 1.86E-06 |
| ENSSSCG00000006625 | RFX5 | 1.88E-06 |
| ENSSSCG00000013332 | KIF18A | 1.89E-06 |
| ENSSSCG00000008963 | AREG | 1.93E-06 |
| ENSSSCG00000021393 | TRMT1L | 1.93E-06 |
| ENSSSCG00000012853 | IRF7 | 1.93E-06 |
| ENSSSCG00000026710 | CARHSP1 | 1.93E-06 |
| ENSSSCG00000036322 | SPRY4 | 1.94E-06 |
| ENSSSCG00000002004 | PSME2 | 2E-06 |
| ENSSSCG00000024973 | NA | 2.08E-06 |
| ENSSSCG00000029260 | NDNF | 2.11E-06 |
| ENSSSCG00000004509 | LIPG | 2.12E-06 |
| ENSSSCG00000025856 | TMEM106A | 2.17E-06 |
| ENSSSCG00000038471 | NUAK2 | 2.17E-06 |
| ENSSSCG00000009396 | PHF11 | 2.23E-06 |
| ENSSSCG00000030642 | PCNA | 2.32E-06 |
| ENSSSCG00000011538 | LMCD1 | 2.34E-06 |
| ENSSSCG00000010915 | NAV1 | 2.35E-06 |
| ENSSSCG00000005533 | PTGS1 | 2.39E-06 |
| ENSSSCG00000006009 | EXT1 | 2.46E-06 |
| ENSSSCG00000038401 | TRAM2 | 2.49E-06 |
| ENSSSCG00000006530 | EFNA1 | 2.62E-06 |
| ENSSSCG00000035952 | TGFB1I1 | 3.17E-06 |
| ENSSSCG00000000263 | TNS2 | 3.24E-06 |
| ENSSSCG00000011676 | NA | 3.64E-06 |
| ENSSSCG00000027467 | DZIP1L | 3.84E-06 |
| ENSSSCG00000016884 | PELO | 3.97E-06 |
| ENSSSCG00000007465 | B4GALT5 | 3.98E-06 |
| ENSSSCG00000017091 | TNIP1 | 4.07E-06 |
| ENSSSCG00000034758 | PDXK | 4.31E-06 |
| ENSSSCG00000030300 | MT2A | 4.42E-06 |
| ENSSSCG00000007817 | IL4R | 4.72E-06 |
| ENSSSCG00000009505 | MBNL2 | 4.75E-06 |
| ENSSSCG00000004290 | TBX18 | 4.85E-06 |
| ENSSSCG00000016554 | MEST | 4.85E-06 |
| ENSSSCG00000006543 | ADAR | 5.09E-06 |
| ENSSSCG00000039542 | NA | 5.11E-06 |
| ENSSSCG00000007572 | LFNG | 5.33E-06 |
| ENSSSCG00000024166 | SLC2A6 | 5.34E-06 |
| ENSSSCG00000016313 | HJURP | 5.41E-06 |
| ENSSSCG00000002375 | RPS6KL1 | 5.45E-06 |
| ENSSSCG00000031337 | SNX18 | 5.45E-06 |
| ENSSSCG00000034216 | OGFR | 5.73E-06 |
| ENSSSCG00000009440 | ELF1 | 5.9E-06 |
| ENSSSCG00000011628 | DNAJC13 | 6.01E-06 |
| ENSSSCG00000021761 | UBAP1 | 6.01E-06 |
| ENSSSCG00000032613 | SNAI1 | 6.08E-06 |
| ENSSSCG00000032176 | SMURF2 | 6.13E-06 |
| ENSSSCG00000000849 | NA | 6.49E-06 |
| ENSSSCG00000003465 | FBLIM1 | 6.7E-06 |
| ENSSSCG00000040181 | ELL | 7.06E-06 |
| ENSSSCG00000001554 | SRPK1 | 7.2E-06 |
| ENSSSCG00000028097 | NA | 7.31E-06 |
| ENSSSCG00000016841 | SLC1A3 | 7.43E-06 |
| ENSSSCG00000000084 | ATF4 | 7.89E-06 |
| ENSSSCG00000008553 | PREB | 7.89E-06 |
| ENSSSCG00000010054 | ADORA2A | 8.21E-06 |
| ENSSSCG00000016062 | NABP1 | 8.44E-06 |
| ENSSSCG00000012055 | MORC3 | 8.44E-06 |
| ENSSSCG00000022300 | MAT2A | 8.44E-06 |
| ENSSSCG00000011389 | AMIGO3 | 8.58E-06 |
| ENSSSCG00000040773 | TOB1 | 8.75E-06 |
| ENSSSCG00000015390 | NA | 8.9E-06 |
| ENSSSCG00000032433 | PTCHD1 | 8.94E-06 |
| ENSSSCG00000004065 | TIAM2 | 9.76E-06 |
| ENSSSCG00000013048 | C11orf84 | 9.96E-06 |
| ENSSSCG00000036340 | ZBTB5 | 1E-05 |
| ENSSSCG00000015435 | NAMPT | 1E-05 |
| ENSSSCG00000002376 | PGF | 1.01E-05 |
| ENSSSCG00000014670 | TRIM5 | 1.01E-05 |
| ENSSSCG00000022246 | NA | 1.04E-05 |
| ENSSSCG00000012112 | ARHGAP6 | 1.07E-05 |
| ENSSSCG00000016991 | DUSP1 | 1.09E-05 |
| ENSSSCG00000013244 | PACSIN3 | 1.09E-05 |
| ENSSSCG00000014780 | TRIM21 | 1.15E-05 |
| ENSSSCG00000011056 | FRMD4A | 1.23E-05 |
| ENSSSCG00000026931 | SERTAD1 | 1.26E-05 |
| ENSSSCG00000009806 | SETD1B | 1.27E-05 |
| ENSSSCG00000002003 | NA | 1.29E-05 |
| ENSSSCG00000003715 | NA | 1.36E-05 |
| ENSSSCG00000009395 | SETDB2 | 1.4E-05 |
| ENSSSCG00000036741 | PITX1 | 1.4E-05 |
| ENSSSCG00000028004 | RIN2 | 1.43E-05 |
| ENSSSCG00000011411 | TMEM115 | 1.43E-05 |
| ENSSSCG00000005358 | NA | 1.49E-05 |
| ENSSSCG00000012277 | TIMP1 | 1.52E-05 |
| ENSSSCG00000012915 | CLCF1 | 1.57E-05 |
| ENSSSCG00000009567 | RASA3 | 1.61E-05 |
| ENSSSCG00000014882 | RSF1 | 1.61E-05 |
| ENSSSCG00000039080 | TRIB2 | 1.63E-05 |
| ENSSSCG00000031789 | ACSL5 | 1.73E-05 |
| ENSSSCG00000034105 | TMEM127 | 1.88E-05 |
| ENSSSCG00000040673 | TMEM140 | 1.9E-05 |
| ENSSSCG00000017475 | RARA | 1.95E-05 |
| ENSSSCG00000025689 | DVL2 | 1.96E-05 |
| ENSSSCG00000024096 | RIPK2 | 2E-05 |
| ENSSSCG00000034012 | CASP3 | 2.07E-05 |
| ENSSSCG00000032436 | NA | 2.07E-05 |
| ENSSSCG00000010312 | PLAU | 2.18E-05 |
| ENSSSCG00000038220 | RXRA | 2.18E-05 |
| ENSSSCG00000029594 | WBP1L | 2.21E-05 |
| ENSSSCG00000039950 | RMI1 | 2.23E-05 |
| ENSSSCG00000014303 | JADE2 | 2.24E-05 |
| ENSSSCG00000014401 | NR3C1 | 2.24E-05 |
| ENSSSCG00000002026 | EFS | 2.27E-05 |
| ENSSSCG00000032843 | AHRR | 2.33E-05 |
| ENSSSCG00000031912 | NA | 2.34E-05 |
| ENSSSCG00000021359 | CDC42EP3 | 2.36E-05 |
| ENSSSCG00000039472 | SLC30A1 | 2.52E-05 |
| ENSSSCG00000032517 | DMXL2 | 2.53E-05 |
| ENSSSCG00000008275 | TTC31 | 2.62E-05 |
| ENSSSCG00000006874 | PALMD | 2.67E-05 |
| ENSSSCG00000007586 | FSCN1 | 2.76E-05 |
| ENSSSCG00000005688 | PTGES | 2.77E-05 |
| ENSSSCG00000017943 | ACAP1 | 2.78E-05 |
| ENSSSCG00000029621 | BMPR1B | 2.79E-05 |
| ENSSSCG00000028063 | TACC2 | 2.82E-05 |
| ENSSSCG00000002470 | DDX24 | 2.83E-05 |
| ENSSSCG00000010235 | SIRT1 | 2.88E-05 |
| ENSSSCG00000027628 | IL6R | 2.89E-05 |
| ENSSSCG00000012074 | NA | 3.07E-05 |
| ENSSSCG00000000549 | PPFIBP1 | 3.09E-05 |
| ENSSSCG00000033657 | GREM1 | 3.11E-05 |
| ENSSSCG00000024623 | USP25 | 3.16E-05 |
| ENSSSCG00000039952 | ZFAND3 | 3.17E-05 |
| ENSSSCG00000003253 | NA | 3.18E-05 |
| ENSSSCG00000010454 | IFIT5 | 3.22E-05 |
| ENSSSCG00000004050 | WTAP | 3.22E-05 |
| ENSSSCG00000025788 | ENPP4 | 3.27E-05 |
| ENSSSCG00000004331 | CASP8AP2 | 3.27E-05 |
| ENSSSCG00000036824 | AVPI1 | 3.41E-05 |
| ENSSSCG00000014204 | DCP2 | 3.43E-05 |
| ENSSSCG00000012173 | SAT1 | 3.45E-05 |
| ENSSSCG00000035284 | BMF | 3.55E-05 |
| ENSSSCG00000006729 | FAM46C | 3.55E-05 |
| ENSSSCG00000009357 | SMAD9 | 3.55E-05 |
| ENSSSCG00000007727 | AUTS2 | 3.62E-05 |
| ENSSSCG00000009638 | RHOBTB2 | 3.65E-05 |
| ENSSSCG00000035212 | KLF6 | 3.78E-05 |
| ENSSSCG00000009384 | INTS6 | 3.78E-05 |
| ENSSSCG00000006324 | ALDH9A1 | 3.99E-05 |
| ENSSSCG00000004948 | SMAD6 | 4.03E-05 |
| ENSSSCG00000027675 | FOXP1 | 4.09E-05 |
| ENSSSCG00000013664 | C19orf66 | 4.09E-05 |
| ENSSSCG00000025598 | COBLL1 | 4.12E-05 |
| ENSSSCG00000011493 | ATXN7 | 4.16E-05 |
| ENSSSCG00000000769 | BID | 4.26E-05 |
| ENSSSCG00000006247 | PLAG1 | 4.34E-05 |
| ENSSSCG00000036437 | NOG | 4.34E-05 |
| ENSSSCG00000017178 | SPHK1 | 4.51E-05 |
| ENSSSCG00000010211 | CCDC6 | 4.61E-05 |
| ENSSSCG00000033830 | CCDC8 | 4.69E-05 |
| ENSSSCG00000023165 | SEMA7A | 4.72E-05 |
| ENSSSCG00000015549 | RNASEL | 4.74E-05 |
| ENSSSCG00000029507 | RASGEF1B | 4.79E-05 |
| ENSSSCG00000001817 | FURIN | 4.82E-05 |
| ENSSSCG00000037732 | SINHCAF | 4.84E-05 |
| ENSSSCG00000025060 | NA | 4.9E-05 |
| ENSSSCG00000008617 | FAM49A | 4.9E-05 |
| ENSSSCG00000009542 | TNFSF13B | 4.99E-05 |
| ENSSSCG00000004700 | PDIA3 | 4.99E-05 |
| ENSSSCG00000010600 | CALHM2 | 5.14E-05 |
| ENSSSCG00000005301 | FAM214B | 5.14E-05 |
| ENSSSCG00000032861 | NUAK1 | 5.29E-05 |
| ENSSSCG00000006502 | ARHGEF2 | 5.29E-05 |
| ENSSSCG00000015071 | SIK3 | 5.33E-05 |
| ENSSSCG00000009959 | ASPHD2 | 5.33E-05 |
| ENSSSCG00000035598 | EDN1 | 5.49E-05 |
| ENSSSCG00000016653 | DNAJB9 | 5.6E-05 |
| ENSSSCG00000007477 | NFATC2 | 5.67E-05 |
| ENSSSCG00000003419 | MAD2L2 | 5.84E-05 |
| ENSSSCG00000028902 | RB1CC1 | 5.95E-05 |
| ENSSSCG00000001951 | PSMA6 | 6.04E-05 |
| ENSSSCG00000004952 | SMAD3 | 6.06E-05 |
| ENSSSCG00000010209 | FAM13C | 6.28E-05 |
| ENSSSCG00000007733 | NA | 6.34E-05 |
| ENSSSCG00000039541 | ANKRD11 | 6.52E-05 |
| ENSSSCG00000026729 | TMEM150C | 6.57E-05 |
| ENSSSCG00000017551 | FAM117A | 6.81E-05 |
| ENSSSCG00000026108 | CDC42EP1 | 6.95E-05 |
| ENSSSCG00000000791 | PDZRN4 | 7.42E-05 |
| ENSSSCG00000016698 | HOXA11 | 7.72E-05 |
| ENSSSCG00000039756 | FOXC1 | 7.73E-05 |
| ENSSSCG00000000645 | GABARAPL1 | 7.82E-05 |
| ENSSSCG00000040366 | ADAMTSL1 | 7.91E-05 |
| ENSSSCG00000007737 | TPST1 | 8.13E-05 |
| ENSSSCG00000013766 | IL27RA | 8.15E-05 |
| ENSSSCG00000004223 | HEY2 | 8.31E-05 |
| ENSSSCG00000007140 | SMOX | 8.43E-05 |
| ENSSSCG00000009676 | ZNF395 | 8.72E-05 |
| ENSSSCG00000008388 | REL | 8.94E-05 |
| ENSSSCG00000016057 | STAT1 | 8.94E-05 |
| ENSSSCG00000006497 | MEX3A | 9.01E-05 |
| ENSSSCG00000001064 | GMPR | 9.17E-05 |
| ENSSSCG00000004897 | ZCCHC2 | 9.27E-05 |
| ENSSSCG00000015136 | UBASH3B | 9.36E-05 |
| ENSSSCG00000040215 | TFAP4 | 9.42E-05 |
| ENSSSCG00000033113 | CHMP7 | 9.42E-05 |
| ENSSSCG00000007463 | PTGIS | 9.44E-05 |
| ENSSSCG00000003137 | PLEKHA4 | 9.47E-05 |
| ENSSSCG00000005943 | ST3GAL1 | 9.47E-05 |
| ENSSSCG00000008919 | EPHA5 | 9.51E-05 |
| ENSSSCG00000023351 | PLA2G4A | 9.67E-05 |
| ENSSSCG00000015396 | SEMA3D | 9.82E-05 |
| ENSSSCG00000039408 | ADCY7 | 0.0001 |
| ENSSSCG00000039751 | NLRC5 | 0.0001 |
| ENSSSCG00000027443 | MRAS | 0.0001 |
| ENSSSCG00000007585 | ACTB | 0.000101 |
| ENSSSCG00000005654 | SPTAN1 | 0.000101 |
| ENSSSCG00000031462 | ZNRF1 | 0.000101 |
| ENSSSCG00000031781 | PSMB10 | 0.000101 |
| ENSSSCG00000040207 | P2RY2 | 0.000103 |
| ENSSSCG00000022159 | FNDC3A | 0.000107 |
| ENSSSCG00000014327 | BRD8 | 0.00011 |
| ENSSSCG00000031610 | NA | 0.000111 |
| ENSSSCG00000016033 | GULP1 | 0.000111 |
| ENSSSCG00000029449 | NA | 0.000116 |
| ENSSSCG00000031866 | TIMP3 | 0.000116 |
| ENSSSCG00000026116 | FHOD1 | 0.000117 |
| ENSSSCG00000016243 | RHBDD1 | 0.000119 |
| ENSSSCG00000003577 | WASF2 | 0.000119 |
| ENSSSCG00000002669 | CRISPLD2 | 0.000121 |
| ENSSSCG00000009240 | PLAC8 | 0.000125 |
| ENSSSCG00000016346 | MAD2L1BP | 0.000127 |
| ENSSSCG00000014361 | NA | 0.000128 |
| ENSSSCG00000006237 | SDCBP | 0.00013 |
| ENSSSCG00000003928 | PLK3 | 0.000133 |
| ENSSSCG00000000737 | TULP3 | 0.000134 |
| ENSSSCG00000023419 | ARHGEF10 | 0.000135 |
| ENSSSCG00000038410 | CPEB2 | 0.000137 |
| ENSSSCG00000011407 | RASSF1 | 0.000139 |
| ENSSSCG00000022839 | NA | 0.000139 |
| ENSSSCG00000040617 | TNFAIP8 | 0.00014 |
| ENSSSCG00000011611 | HMCES | 0.000141 |
| ENSSSCG00000011251 | MYD88 | 0.000141 |
| ENSSSCG00000008197 | SEMA4C | 0.000141 |
| ENSSSCG00000015584 | PROX1 | 0.000141 |
| ENSSSCG00000022478 | STK10 | 0.000142 |
| ENSSSCG00000015277 | SOX13 | 0.000142 |
| ENSSSCG00000000625 | LRP6 | 0.000142 |
| ENSSSCG00000016501 | KDM7A | 0.00015 |
| ENSSSCG00000013554 | TRIP10 | 0.000154 |
| ENSSSCG00000013400 | MICAL2 | 0.000156 |
| ENSSSCG00000035223 | SYNM | 0.000157 |
| ENSSSCG00000000014 | FAM118A | 0.000158 |
| ENSSSCG00000036160 | ZNF250 | 0.00016 |
| ENSSSCG00000017258 | FAM20A | 0.000161 |
| ENSSSCG00000022895 | CRYBG3 | 0.000164 |
| ENSSSCG00000007864 | GPRC5B | 0.000164 |
| ENSSSCG00000001518 | ITPR3 | 0.000166 |
| ENSSSCG00000006857 | COL11A1 | 0.000167 |
| ENSSSCG00000015563 | RGL1 | 0.000173 |
| ENSSSCG00000008334 | MXD1 | 0.000175 |
| ENSSSCG00000001070 | FAM8A1 | 0.000182 |
| ENSSSCG00000040608 | AKR1B1 | 0.000182 |
| ENSSSCG00000039862 | TRIB3 | 0.000183 |
| ENSSSCG00000040355 | TICRR | 0.000183 |
| ENSSSCG00000011810 | BCL6 | 0.000184 |
| ENSSSCG00000007451 | SLC2A10 | 0.000184 |
| ENSSSCG00000029482 | CHST15 | 0.000185 |
| ENSSSCG00000038958 | DNM3 | 0.000185 |
| ENSSSCG00000033879 | ZNF280B | 0.000188 |
| ENSSSCG00000016866 | GHR | 0.000192 |
| ENSSSCG00000009022 | MAB21L2 | 0.000192 |
| ENSSSCG00000033089 | NA | 0.000193 |
| ENSSSCG00000001347 | PPP1R10 | 0.000196 |
| ENSSSCG00000037015 | SESN3 | 0.000211 |
| ENSSSCG00000017877 | ANKFY1 | 0.000213 |
| ENSSSCG00000040731 | TMEM65 | 0.000214 |
| ENSSSCG00000014339 | CTNNA1 | 0.000219 |
| ENSSSCG00000002753 | NOB1 | 0.000227 |
| ENSSSCG00000035069 | CHSY3 | 0.000229 |
| ENSSSCG00000006919 | NA | 0.000233 |
| ENSSSCG00000011317 | FYCO1 | 0.000235 |
| ENSSSCG00000000078 | TNRC6B | 0.000235 |
| ENSSSCG00000029708 | SLC25A38 | 0.000238 |
| ENSSSCG00000039474 | FBXL14 | 0.000238 |
| ENSSSCG00000039616 | ZBTB25 | 0.000243 |
| ENSSSCG00000000837 | CHST11 | 0.000249 |
| ENSSSCG00000028529 | REM1 | 0.00025 |
| ENSSSCG00000003513 | ECE1 | 0.00025 |
| ENSSSCG00000035715 | GCH1 | 0.000263 |
| ENSSSCG00000036002 | TGIF2 | 0.000263 |
| ENSSSCG00000013037 | VEGFB | 0.000267 |
| ENSSSCG00000036751 | PPM1H | 0.000272 |
| ENSSSCG00000009040 | SMAD1 | 0.000278 |
| ENSSSCG00000011425 | RAD54L2 | 0.000286 |
| ENSSSCG00000032658 | TRAF4 | 0.000287 |
| ENSSSCG00000037144 | CDKN1B | 0.00029 |
| ENSSSCG00000005267 | ANXA1 | 0.00029 |
| ENSSSCG00000005078 | DAAM1 | 0.000294 |
| ENSSSCG00000017066 | GEMIN5 | 0.000295 |
| ENSSSCG00000021750 | MEX3D | 0.000296 |
| ENSSSCG00000029236 | ZBTB7A | 0.000297 |
| ENSSSCG00000024793 | PORCN | 0.000297 |
| ENSSSCG00000010212 | NA | 0.000299 |
| ENSSSCG00000011074 | ARHGAP21 | 0.000303 |
| ENSSSCG00000037066 | GADD45A | 0.000308 |
| ENSSSCG00000016535 | CALD1 | 0.000308 |
| ENSSSCG00000004917 | MALT1 | 0.000311 |
| ENSSSCG00000008504 | CRIM1 | 0.000314 |
| ENSSSCG00000014540 | NA | 0.000314 |
| ENSSSCG00000003909 | NA | 0.000329 |
| ENSSSCG00000005022 | NIN | 0.000334 |
| ENSSSCG00000010186 | URB2 | 0.000337 |
| ENSSSCG00000008265 | DOK1 | 0.000338 |
| ENSSSCG00000039094 | PLEC | 0.000338 |
| ENSSSCG00000006340 | UAP1 | 0.000341 |
| ENSSSCG00000000475 | IRAK3 | 0.00035 |
| ENSSSCG00000009834 | ATXN2 | 0.000351 |
| ENSSSCG00000034379 | MAP2K3 | 0.000358 |
| ENSSSCG00000013236 | MYBPC3 | 0.000358 |
| ENSSSCG00000036081 | TBC1D20 | 0.000364 |
| ENSSSCG00000017804 | ABR | 0.000378 |
| ENSSSCG00000000728 | PARP11 | 0.00038 |
| ENSSSCG00000036488 | KLF3 | 0.000387 |
| ENSSSCG00000040166 | FLRT2 | 0.00039 |
| ENSSSCG00000001611 | NFYA | 0.000394 |
| ENSSSCG00000014012 | GFPT2 | 0.000394 |
| ENSSSCG00000004058 | EZR | 0.000394 |
| ENSSSCG00000022689 | GADD45B | 0.000394 |
| ENSSSCG00000023229 | ETV5 | 0.000395 |
| ENSSSCG00000016634 | CAV1 | 0.000396 |
| ENSSSCG00000028033 | SECISBP2 | 0.000397 |
| ENSSSCG00000031262 | TXNIP | 0.000412 |
| ENSSSCG00000009886 | TRAFD1 | 0.000413 |
| ENSSSCG00000033001 | FZD8 | 0.000413 |
| ENSSSCG00000030548 | HERC5 | 0.000416 |
| ENSSSCG00000017890 | KIAA0753 | 0.000416 |
| ENSSSCG00000020962 | DYNLL2 | 0.000429 |
| ENSSSCG00000034087 | TNFSF15 | 0.000431 |
| ENSSSCG00000031970 | RASSF5 | 0.000434 |
| ENSSSCG00000001081 | SOX4 | 0.000439 |
| ENSSSCG00000039905 | C1orf109 | 0.000441 |
| ENSSSCG00000036063 | LPAR6 | 0.000459 |
| ENSSSCG00000001912 | PML | 0.000462 |
| ENSSSCG00000009293 | NA | 0.000464 |
| ENSSSCG00000017589 | DLX3 | 0.000468 |
| ENSSSCG00000017607 | TMEM100 | 0.000477 |
| ENSSSCG00000030108 | ZNFX1 | 0.000477 |
| ENSSSCG00000007675 | EPHB4 | 0.00049 |
| ENSSSCG00000022208 | TNFRSF1B | 0.000493 |
| ENSSSCG00000011430 | DUSP7 | 0.000494 |
| ENSSSCG00000036746 | RASL10B | 0.000504 |
| ENSSSCG00000011194 | ANKRD28 | 0.000507 |
| ENSSSCG00000016922 | GPBP1 | 0.000518 |
| ENSSSCG00000016432 | PRKAG2 | 0.000518 |
| ENSSSCG00000006290 | SLC19A2 | 0.000528 |
| ENSSSCG00000000403 | BAZ2A | 0.000538 |
| ENSSSCG00000010340 | FAM213A | 0.000547 |
| ENSSSCG00000009652 | KCTD9 | 0.000551 |
| ENSSSCG00000002298 | ZFYVE26 | 0.000559 |
| ENSSSCG00000040267 | CYS1 | 0.000573 |
| ENSSSCG00000013307 | LMO2 | 0.000589 |
| ENSSSCG00000023379 | UBE2L6 | 0.00059 |
| ENSSSCG00000008468 | PKDCC | 0.000594 |
| ENSSSCG00000014794 | NUP98 | 0.00061 |
| ENSSSCG00000005211 | CD274 | 0.000612 |
| ENSSSCG00000018058 | ARHGAP23 | 0.000623 |
| ENSSSCG00000011136 | NA | 0.000626 |
| ENSSSCG00000017605 | MMD | 0.000626 |
| ENSSSCG00000032046 | NA | 0.000632 |
| ENSSSCG00000013017 | EHD1 | 0.000649 |
| ENSSSCG00000007664 | AGFG2 | 0.00068 |
| ENSSSCG00000024635 | SPART | 0.000685 |
| ENSSSCG00000039867 | UCK2 | 0.000709 |
| ENSSSCG00000011496 | ADAMTS9 | 0.000711 |
| ENSSSCG00000030451 | LRRC14 | 0.000732 |
| ENSSSCG00000015519 | RASAL2 | 0.000748 |
| ENSSSCG00000007808 | NFATC2IP | 0.000757 |
| ENSSSCG00000007530 | PPP1R3D | 0.00077 |
| ENSSSCG00000003197 | MED25 | 0.000776 |
| ENSSSCG00000007135 | NA | 0.000778 |
| ENSSSCG00000013303 | ABTB2 | 0.000788 |
| ENSSSCG00000016217 | DNAJB2 | 0.00081 |
| ENSSSCG00000009172 | PPP3CA | 0.000818 |
| ENSSSCG00000017202 | H3F3A | 0.000818 |
| ENSSSCG00000030241 | TSC22D3 | 0.000818 |
| ENSSSCG00000032164 | PEA15 | 0.000818 |
| ENSSSCG00000015106 | HYOU1 | 0.000818 |
| ENSSSCG00000039314 | MCL1 | 0.000818 |
| ENSSSCG00000008446 | SIX2 | 0.000825 |
| ENSSSCG00000009390 | SPRYD7 | 0.000841 |
| ENSSSCG00000013655 | ICAM1 | 0.000848 |
| ENSSSCG00000005268 | RORB | 0.000848 |
| ENSSSCG00000013278 | TSPAN18 | 0.000858 |
| ENSSSCG00000017924 | PELP1 | 0.00086 |
| ENSSSCG00000007309 | RBM39 | 0.00086 |
| ENSSSCG00000040815 | DUSP5 | 0.000877 |
| ENSSSCG00000005518 | NA | 0.000877 |
| ENSSSCG00000008227 | ST3GAL5 | 0.000877 |
| ENSSSCG00000023724 | ZBTB1 | 0.000877 |
| ENSSSCG00000010313 | VCL | 0.000877 |
| ENSSSCG00000033626 | SREBF1 | 0.000877 |
| ENSSSCG00000035859 | WNT5A | 0.0009 |
| ENSSSCG00000006169 | ZFHX4 | 0.000907 |
| ENSSSCG00000039555 | JOSD1 | 0.000915 |
| ENSSSCG00000014950 | VSTM5 | 0.000967 |
| ENSSSCG00000018015 | DNAH9 | 0.000985 |
| ENSSSCG00000010575 | PPRC1 | 0.000992 |
| ENSSSCG00000013391 | NA | 0.001006 |
| ENSSSCG00000011239 | NA | 0.001006 |
| ENSSSCG00000025156 | BRWD3 | 0.001007 |
| ENSSSCG00000036556 | IL10RB | 0.001014 |
| ENSSSCG00000013551 | C3 | 0.001014 |
| ENSSSCG00000002464 | PRIMA1 | 0.001014 |
| ENSSSCG00000023247 | OPTN | 0.001018 |
| ENSSSCG00000024933 | SLC36A4 | 0.001024 |
| ENSSSCG00000029805 | RHOBTB3 | 0.001029 |
| ENSSSCG00000009326 | KATNAL1 | 0.001042 |
| ENSSSCG00000026516 | EPHB3 | 0.001072 |
| ENSSSCG00000004154 | TNFAIP3 | 0.001072 |
| ENSSSCG00000023362 | RHBDF2 | 0.001086 |
| ENSSSCG00000017614 | TRIM25 | 0.001091 |
| ENSSSCG00000005935 | AGO2 | 0.001096 |
| ENSSSCG00000029438 | SESN2 | 0.001102 |
| ENSSSCG00000021440 | GPSM2 | 0.001127 |
| ENSSSCG00000029760 | CIPC | 0.001164 |
| ENSSSCG00000014431 | AFAP1L1 | 0.001202 |
| ENSSSCG00000014242 | ZNF608 | 0.001205 |
| ENSSSCG00000038487 | TMPO | 0.001217 |
| ENSSSCG00000029380 | ZFP2 | 0.001224 |
| ENSSSCG00000024674 | ABL2 | 0.00126 |
| ENSSSCG00000017391 | PLEKHH3 | 0.001284 |
| ENSSSCG00000014168 | ELL2 | 0.001292 |
| ENSSSCG00000026552 | MFSD14B | 0.001292 |
| ENSSSCG00000001341 | NA | 0.001297 |
| ENSSSCG00000015444 | LAMB1 | 0.001358 |
| ENSSSCG00000015498 | RC3H1 | 0.001388 |
| ENSSSCG00000015378 | SP4 | 0.001458 |
| ENSSSCG00000015645 | NA | 0.001459 |
| ENSSSCG00000027860 | ERAP2 | 0.00146 |
| ENSSSCG00000017962 | KDM6B | 0.001463 |
| ENSSSCG00000029866 | CCDC112 | 0.001473 |
| ENSSSCG00000017497 | ERBB2 | 0.00149 |
| ENSSSCG00000017428 | JUP | 0.00149 |
| ENSSSCG00000034858 | RAP1GAP2 | 0.001501 |
| ENSSSCG00000001042 | MAK | 0.001508 |
| ENSSSCG00000007493 | NA | 0.001522 |
| ENSSSCG00000036756 | ZNF496 | 0.001523 |
| ENSSSCG00000003192 | IL4I1 | 0.001523 |
| ENSSSCG00000000704 | TAPBPL | 0.001526 |
| ENSSSCG00000004963 | NA | 0.001526 |
| ENSSSCG00000035774 | ERRFI1 | 0.001526 |
| ENSSSCG00000033823 | NA | 0.001567 |
| ENSSSCG00000016101 | CFLAR | 0.001572 |
| ENSSSCG00000009633 | NA | 0.001578 |
| ENSSSCG00000017753 | KSR1 | 0.001613 |
| ENSSSCG00000040383 | MFAP1 | 0.001629 |
| ENSSSCG00000010073 | CHCHD10 | 0.001649 |
| ENSSSCG00000029815 | SRGAP1 | 0.00165 |
| ENSSSCG00000000457 | USP15 | 0.001668 |
| ENSSSCG00000001471 | BRD2 | 0.001689 |
| ENSSSCG00000010605 | STN1 | 0.001737 |
| ENSSSCG00000036383 | LGALS3BP | 0.001737 |
| ENSSSCG00000010968 | IL11RA | 0.00176 |
| ENSSSCG00000023710 | REEP1 | 0.001768 |
| ENSSSCG00000009468 | KCTD12 | 0.001836 |
| ENSSSCG00000023525 | TMEM26 | 0.001846 |
| ENSSSCG00000032357 | APPL1 | 0.00186 |
| ENSSSCG00000030610 | FNBP4 | 0.001894 |
| ENSSSCG00000005457 | NA | 0.001894 |
| ENSSSCG00000008147 | FHL2 | 0.001903 |
| ENSSSCG00000025114 | FMNL3 | 0.001904 |
| ENSSSCG00000035867 | GFOD1 | 0.001915 |
| ENSSSCG00000016067 | STK17B | 0.001932 |
| ENSSSCG00000016656 | ELMO1 | 0.001937 |
| ENSSSCG00000027700 | RPRD1A | 0.001951 |
| ENSSSCG00000027342 | BAZ1B | 0.002026 |
| ENSSSCG00000005269 | TRPM6 | 0.002039 |
| ENSSSCG00000032561 | PDCD1LG2 | 0.002039 |
| ENSSSCG00000000559 | RASSF8 | 0.002054 |
| ENSSSCG00000012504 | NAP1L3 | 0.002136 |
| ENSSSCG00000032674 | TCEA3 | 0.002142 |
| ENSSSCG00000017826 | SMG6 | 0.002174 |
| ENSSSCG00000001488 | GCLC | 0.002175 |
| ENSSSCG00000021731 | WWC2 | 0.002185 |
| ENSSSCG00000010169 | SIPA1L2 | 0.002196 |
| ENSSSCG00000000766 | CECR2 | 0.002202 |
| ENSSSCG00000004332 | BACH2 | 0.002207 |
| ENSSSCG00000030260 | PFN2 | 0.00222 |
| ENSSSCG00000008162 | IL1R1 | 0.002224 |
| ENSSSCG00000036326 | LATS2 | 0.002271 |
| ENSSSCG00000026301 | RAP2C | 0.002289 |
| ENSSSCG00000010148 | ERO1B | 0.00231 |
| ENSSSCG00000013735 | JUNB | 0.002313 |
| ENSSSCG00000009042 | OTUD4 | 0.002352 |
| ENSSSCG00000002795 | CDH11 | 0.002371 |
| ENSSSCG00000016263 | NA | 0.002395 |
| ENSSSCG00000028466 | UTP3 | 0.002418 |
| ENSSSCG00000034973 | CXCL12 | 0.002428 |
| ENSSSCG00000039348 | H1F0 | 0.002429 |
| ENSSSCG00000015336 | SLC25A13 | 0.00247 |
| ENSSSCG00000031329 | ST8SIA1 | 0.00247 |
| ENSSSCG00000029331 | PALLD | 0.002495 |
| ENSSSCG00000035058 | PID1 | 0.002495 |
| ENSSSCG00000026004 | MSRB3 | 0.002593 |
| ENSSSCG00000028509 | RBM8A | 0.002616 |
| ENSSSCG00000007719 | NA | 0.002651 |
| ENSSSCG00000036317 | VASP | 0.002654 |
| ENSSSCG00000040013 | MTUS1 | 0.002699 |
| ENSSSCG00000039053 | VGF | 0.002727 |
| ENSSSCG00000003613 | NA | 0.002732 |
| ENSSSCG00000016330 | NA | 0.002741 |
| ENSSSCG00000014277 | IRF1 | 0.002745 |
| ENSSSCG00000004163 | BCLAF1 | 0.002772 |
| ENSSSCG00000010894 | TP53BP2 | 0.002783 |
| ENSSSCG00000017041 | ADRA1B | 0.002817 |
| ENSSSCG00000039393 | SPRED1 | 0.00282 |
| ENSSSCG00000002350 | ELMSAN1 | 0.002855 |
| ENSSSCG00000015850 | DUSP4 | 0.002891 |
| ENSSSCG00000027266 | PNPLA3 | 0.002893 |
| ENSSSCG00000016002 | NA | 0.002902 |
| ENSSSCG00000006889 | ARHGAP29 | 0.002917 |
| ENSSSCG00000006051 | CTHRC1 | 0.002954 |
| ENSSSCG00000032266 | SLC37A3 | 0.002999 |
| ENSSSCG00000003472 | ARHGEF19 | 0.003004 |
| ENSSSCG00000022031 | CHST2 | 0.003028 |
| ENSSSCG00000013742 | NFIX | 0.00304 |
| ENSSSCG00000034484 | SPEN | 0.003094 |
| ENSSSCG00000035969 | THRA | 0.003132 |
| ENSSSCG00000016453 | TCAF1 | 0.003163 |
| ENSSSCG00000026006 | KLF13 | 0.00319 |
| ENSSSCG00000029592 | GPRC5A | 0.003285 |
| ENSSSCG00000029430 | IPO13 | 0.00329 |
| ENSSSCG00000036236 | ELOVL6 | 0.003353 |
| ENSSSCG00000024351 | MET | 0.003404 |
| ENSSSCG00000037238 | NA | 0.003498 |
| ENSSSCG00000039887 | MARCH5 | 0.003509 |
| ENSSSCG00000000839 | ALDH1L2 | 0.003535 |
| ENSSSCG00000000164 | CRY1 | 0.003535 |
| ENSSSCG00000009140 | CASP6 | 0.003567 |
| ENSSSCG00000013722 | WDR83 | 0.003617 |
| ENSSSCG00000034493 | ST3GAL6 | 0.003641 |
| ENSSSCG00000007351 | FAM83D | 0.003653 |
| ENSSSCG00000016018 | FRZB | 0.003654 |
| ENSSSCG00000013522 | SAFB | 0.003655 |
| ENSSSCG00000011727 | PTX3 | 0.003683 |
| ENSSSCG00000036067 | PDP2 | 0.003685 |
| ENSSSCG00000024970 | ETV3 | 0.003777 |
| ENSSSCG00000001078 | MBOAT1 | 0.003794 |
| ENSSSCG00000035949 | FTO | 0.003794 |
| ENSSSCG00000032015 | SH3BGRL2 | 0.003812 |
| ENSSSCG00000010795 | NA | 0.003822 |
| ENSSSCG00000037958 | TOB2 | 0.003846 |
| ENSSSCG00000013352 | E2F8 | 0.003925 |
| ENSSSCG00000011889 | GSK3B | 0.003953 |
| ENSSSCG00000007541 | PDGFA | 0.003955 |
| ENSSSCG00000036129 | RNF114 | 0.003964 |
| ENSSSCG00000025260 | CARD10 | 0.003988 |
| ENSSSCG00000034752 | SUPT4H1 | 0.003988 |
| ENSSSCG00000034427 | MEX3C | 0.003988 |
| ENSSSCG00000002859 | ANKRD27 | 0.004115 |
| ENSSSCG00000011000 | DNAJA1 | 0.004115 |
| ENSSSCG00000016781 | TRIO | 0.004116 |
| ENSSSCG00000010071 | MMP11 | 0.004117 |
| ENSSSCG00000021712 | HERC6 | 0.004124 |
| ENSSSCG00000029763 | IFI35 | 0.004208 |
| ENSSSCG00000017785 | NUFIP2 | 0.004238 |
| ENSSSCG00000000521 | PHLDA1 | 0.004238 |
| ENSSSCG00000038838 | DLX5 | 0.004261 |
| ENSSSCG00000009151 | CYP2U1 | 0.004348 |
| ENSSSCG00000026113 | ZBTB20 | 0.004429 |
| ENSSSCG00000008041 | PKD1 | 0.004494 |
| ENSSSCG00000015589 | VASH2 | 0.004661 |
| ENSSSCG00000024592 | ANKRD17 | 0.004701 |
| ENSSSCG00000023391 | LONP2 | 0.004729 |
| ENSSSCG00000000843 | TXNRD1 | 0.004754 |
| ENSSSCG00000015581 | CENPF | 0.004772 |
| ENSSSCG00000016090 | SPATS2L | 0.004786 |
| ENSSSCG00000008130 | CIAO1 | 0.004786 |
| ENSSSCG00000005657 | PKN3 | 0.004855 |
| ENSSSCG00000009122 | ARSJ | 0.004875 |
| ENSSSCG00000037267 | MAX | 0.004904 |
| ENSSSCG00000000716 | KCNA1 | 0.004922 |
| ENSSSCG00000004859 | ZNF516 | 0.004935 |
| ENSSSCG00000023176 | TROAP | 0.004958 |
| ENSSSCG00000036790 | AKAP7 | 0.004958 |
| ENSSSCG00000006830 | NA | 0.005082 |
| ENSSSCG00000024488 | NUBP1 | 0.005082 |
| ENSSSCG00000021862 | PTGER2 | 0.005164 |
| ENSSSCG00000038423 | GLA | 0.005168 |
| ENSSSCG00000000567 | SOX5 | 0.005299 |
| ENSSSCG00000012361 | AMER1 | 0.005344 |
| ENSSSCG00000011825 | ATP13A3 | 0.005491 |
| ENSSSCG00000000162 | BTBD11 | 0.005491 |
| ENSSSCG00000012262 | KDM6A | 0.005496 |
| ENSSSCG00000008484 | SRSF7 | 0.005577 |
| ENSSSCG00000024958 | GPR173 | 0.005577 |
| ENSSSCG00000005498 | PAPPA | 0.005579 |
| ENSSSCG00000003730 | RNF138 | 0.005612 |
| ENSSSCG00000007237 | PDRG1 | 0.00563 |
| ENSSSCG00000016836 | NADK2 | 0.005716 |
| ENSSSCG00000006107 | PDP1 | 0.005739 |
| ENSSSCG00000036724 | CRYAB | 0.005751 |
| ENSSSCG00000004146 | REPS1 | 0.005764 |
| ENSSSCG00000009864 | MED13L | 0.005778 |
| ENSSSCG00000026602 | PTGIR | 0.005831 |
| ENSSSCG00000016254 | CCL20 | 0.005865 |
| ENSSSCG00000013311 | KIAA1549L | 0.005865 |
| ENSSSCG00000038141 | TICAM2 | 0.005922 |
| ENSSSCG00000017991 | PIK3R5 | 0.005922 |
| ENSSSCG00000035939 | ZNF512B | 0.006066 |
| ENSSSCG00000006651 | ADAMTSL4 | 0.006071 |
| ENSSSCG00000001805 | WHAMM | 0.006099 |
| ENSSSCG00000026596 | SYNRG | 0.006111 |
| ENSSSCG00000028552 | BHLHE41 | 0.006129 |
| ENSSSCG00000021911 | NDRG4 | 0.006146 |
| ENSSSCG00000021232 | SYNC | 0.006175 |
| ENSSSCG00000034378 | IFNGR2 | 0.006186 |
| ENSSSCG00000001009 | RIPK1 | 0.006287 |
| ENSSSCG00000000600 | EPS8 | 0.006363 |
| ENSSSCG00000005524 | DAB2IP | 0.006367 |
| ENSSSCG00000023662 | CHST3 | 0.006444 |
| ENSSSCG00000003839 | NA | 0.006444 |
| ENSSSCG00000029652 | TMBIM6 | 0.006465 |
| ENSSSCG00000016851 | OSMR | 0.006542 |
| ENSSSCG00000008136 | RANBP2 | 0.006555 |
| ENSSSCG00000015453 | PDIA4 | 0.006558 |
| ENSSSCG00000022773 | NA | 0.006558 |
| ENSSSCG00000013410 | SWAP70 | 0.006563 |
| ENSSSCG00000016981 | CPEB4 | 0.006575 |
| ENSSSCG00000016230 | EPHA4 | 0.006621 |
| ENSSSCG00000040017 | NKX2-2 | 0.006621 |
| ENSSSCG00000024384 | DCAF12 | 0.006845 |
| ENSSSCG00000002656 | ZCCHC14 | 0.006845 |
| ENSSSCG00000026001 | DNAJC1 | 0.006872 |
| ENSSSCG00000012880 | CPT1A | 0.006887 |
| ENSSSCG00000011278 | TRAK1 | 0.006984 |
| ENSSSCG00000024913 | HYLS1 | 0.006984 |
| ENSSSCG00000040010 | BCL2A1 | 0.007002 |
| ENSSSCG00000013988 | IBA57 | 0.007054 |
| ENSSSCG00000005073 | ARID4A | 0.007089 |
| ENSSSCG00000011193 | BTD | 0.007114 |
| ENSSSCG00000022464 | KBTBD2 | 0.007164 |
| ENSSSCG00000034293 | ARL4C | 0.007169 |
| ENSSSCG00000023045 | BAHCC1 | 0.007169 |
| ENSSSCG00000007356 | PLCG1 | 0.007273 |
| ENSSSCG00000011867 | MYLK | 0.00728 |
| ENSSSCG00000014538 | MGAT1 | 0.007392 |
| ENSSSCG00000022005 | SFXN3 | 0.007392 |
| ENSSSCG00000009630 | EGR3 | 0.007486 |
| ENSSSCG00000002959 | FAM98C | 0.007486 |
| ENSSSCG00000014927 | NOX4 | 0.007495 |
| ENSSSCG00000026989 | RAD51D | 0.007505 |
| ENSSSCG00000034570 | IFI6 | 0.007587 |
| ENSSSCG00000014998 | AASDHPPT | 0.007657 |
| ENSSSCG00000028239 | FBXL7 | 0.007686 |
| ENSSSCG00000015310 | AKAP9 | 0.007707 |
| ENSSSCG00000004962 | CORO2B | 0.007707 |
| ENSSSCG00000009998 | CASTOR1 | 0.007732 |
| ENSSSCG00000022504 | CDON | 0.00795 |
| ENSSSCG00000009243 | THAP9 | 0.00795 |
| ENSSSCG00000010438 | ATAD1 | 0.007983 |
| ENSSSCG00000030115 | HS1BP3 | 0.008038 |
| ENSSSCG00000010589 | SFXN2 | 0.00806 |
| ENSSSCG00000040638 | DIO2 | 0.008139 |
| ENSSSCG00000028190 | HOMEZ | 0.008139 |
| ENSSSCG00000001431 | NA | 0.008175 |
| ENSSSCG00000038569 | ARMCX1 | 0.008214 |
| ENSSSCG00000007767 | ZNF668 | 0.008243 |
| ENSSSCG00000009230 | WDFY3 | 0.008253 |
| ENSSSCG00000009101 | PRDM5 | 0.0084 |
| ENSSSCG00000017835 | CLUH | 0.008453 |
| ENSSSCG00000025218 | TXNL4B | 0.008497 |
| ENSSSCG00000020744 | DUSP3 | 0.008502 |
| ENSSSCG00000013539 | GTF2F1 | 0.008508 |
| ENSSSCG00000032728 | EFNB1 | 0.008599 |
| ENSSSCG00000027857 | DMXL1 | 0.00866 |
| ENSSSCG00000026959 | NPAT | 0.008742 |
| ENSSSCG00000002820 | RSPRY1 | 0.008757 |
| ENSSSCG00000000846 | HCFC2 | 0.008853 |
| ENSSSCG00000025286 | MCTP1 | 0.008877 |
| ENSSSCG00000006947 | SYDE2 | 0.008889 |
| ENSSSCG00000002457 | ITPK1 | 0.00894 |
| ENSSSCG00000000531 | BICD1 | 0.009075 |
| ENSSSCG00000030680 | TCF7 | 0.009166 |
| ENSSSCG00000034616 | LRRC58 | 0.009178 |
| ENSSSCG00000032527 | FOSL2 | 0.009327 |
| ENSSSCG00000033971 | ZNF407 | 0.009327 |
| ENSSSCG00000006552 | HAX1 | 0.009327 |
| ENSSSCG00000002898 | NA | 0.009385 |
| ENSSSCG00000008747 | NCAPG | 0.009398 |
| ENSSSCG00000009148 | LEF1 | 0.009654 |
| ENSSSCG00000001986 | NFATC4 | 0.009665 |
| ENSSSCG00000012315 | SYNJ2 | 0.009685 |
| ENSSSCG00000005229 | VLDLR | 0.009697 |
| ENSSSCG00000016958 | PIK3R1 | 0.009706 |
| ENSSSCG00000004671 | SPATA5L1 | 0.009706 |
| ENSSSCG00000011874 | PARP14 | 0.009706 |
| ENSSSCG00000012001 | ROBO1 | 0.009723 |
| ENSSSCG00000037583 | GNB4 | 0.009729 |
| ENSSSCG00000023307 | FBXW11 | 0.009956 |
| ENSSSCG00000004144 | HECA | 0.00997 |
| ENSSSCG00000008835 | RASL11B | 0.010003 |
| ENSSSCG00000033120 | PALM2 | 0.010069 |
| ENSSSCG00000029230 | ECM1 | 0.010074 |
| ENSSSCG00000023975 | C15orf52 | 0.010084 |
| ENSSSCG00000001977 | STXBP6 | 0.010108 |
| ENSSSCG00000011332 | SETD2 | 0.010135 |
| ENSSSCG00000001689 | GTPBP2 | 0.010135 |
| ENSSSCG00000004825 | CHSY1 | 0.010207 |
| ENSSSCG00000031552 | NA | 0.010235 |
| ENSSSCG00000003107 | ARHGAP35 | 0.010235 |
| ENSSSCG00000034364 | NA | 0.010319 |
| ENSSSCG00000036700 | RPUSD2 | 0.010381 |
| ENSSSCG00000013731 | DNASE2 | 0.01039 |
| ENSSSCG00000027709 | PARP9 | 0.010398 |
| ENSSSCG00000014448 | ARSI | 0.010398 |
| ENSSSCG00000033074 | ZCCHC24 | 0.010519 |
| ENSSSCG00000017466 | CCR7 | 0.01064 |
| ENSSSCG00000007812 | XPO6 | 0.010711 |
| ENSSSCG00000027777 | NA | 0.010736 |
| ENSSSCG00000014249 | MARCH3 | 0.010746 |
| ENSSSCG00000026382 | PPP2R5E | 0.010746 |
| ENSSSCG00000035612 | COX6B2 | 0.010751 |
| ENSSSCG00000006132 | MMP16 | 0.010778 |
| ENSSSCG00000032705 | USP42 | 0.010778 |
| ENSSSCG00000024614 | ITSN1 | 0.010843 |
| ENSSSCG00000012967 | FOSL1 | 0.010881 |
| ENSSSCG00000025130 | KLF12 | 0.010909 |
| ENSSSCG00000003888 | STIL | 0.010917 |
| ENSSSCG00000035971 | DUSP2 | 0.010985 |
| ENSSSCG00000012981 | RELA | 0.010985 |
| ENSSSCG00000023788 | TBC1D30 | 0.010992 |
| ENSSSCG00000013753 | IER2 | 0.011028 |
| ENSSSCG00000025214 | TSHZ3 | 0.011055 |
| ENSSSCG00000000209 | NCKAP5L | 0.011117 |
| ENSSSCG00000032652 | NA | 0.011174 |
| ENSSSCG00000009052 | NA | 0.011185 |
| ENSSSCG00000033521 | NA | 0.011186 |
| ENSSSCG00000009616 | HR | 0.011186 |
| ENSSSCG00000004473 | PHIP | 0.011186 |
| ENSSSCG00000000104 | DDX17 | 0.011237 |
| ENSSSCG00000024373 | TRIP12 | 0.011284 |
| ENSSSCG00000006781 | CTTNBP2NL | 0.011334 |
| ENSSSCG00000031744 | NA | 0.011379 |
| ENSSSCG00000005215 | JAK2 | 0.011472 |
| ENSSSCG00000035341 | C6orf89 | 0.011506 |
| ENSSSCG00000004505 | SMAD2 | 0.01157 |
| ENSSSCG00000035230 | PIEZO1 | 0.011582 |
| ENSSSCG00000002252 | ARRDC4 | 0.011624 |
| ENSSSCG00000006392 | IGSF8 | 0.011627 |
| ENSSSCG00000006496 | LMNA | 0.01171 |
| ENSSSCG00000027294 | CDV3 | 0.011799 |
| ENSSSCG00000024136 | AMPH | 0.011875 |
| ENSSSCG00000016824 | RAI14 | 0.011988 |
| ENSSSCG00000007985 | TMEM8A | 0.012158 |
| ENSSSCG00000016510 | UBN2 | 0.012159 |
| ENSSSCG00000006940 | CYR61 | 0.012159 |
| ENSSSCG00000002404 | SPTLC2 | 0.012239 |
| ENSSSCG00000004695 | WDR76 | 0.012239 |
| ENSSSCG00000032715 | CERS6 | 0.012395 |
| ENSSSCG00000016061 | MYO1B | 0.012399 |
| ENSSSCG00000010181 | C1orf198 | 0.012449 |
| ENSSSCG00000003301 | PPP6R1 | 0.012511 |
| ENSSSCG00000031652 | RPGRIP1L | 0.012533 |
| ENSSSCG00000008633 | PDIA6 | 0.012551 |
| ENSSSCG00000017416 | DHX58 | 0.012844 |
| ENSSSCG00000038954 | PLIN3 | 0.012864 |
| ENSSSCG00000030005 | LGALSL | 0.013022 |
| ENSSSCG00000003439 | DHRS3 | 0.013022 |
| ENSSSCG00000008980 | SCARB2 | 0.013151 |
| ENSSSCG00000030565 | GRIA4 | 0.013229 |
| ENSSSCG00000010551 | BLOC1S2 | 0.01337 |
| ENSSSCG00000006875 | PLPPR4 | 0.01339 |
| ENSSSCG00000003532 | LUZP1 | 0.013405 |
| ENSSSCG00000036364 | EGR4 | 0.013429 |
| ENSSSCG00000004570 | NA | 0.013476 |
| ENSSSCG00000001994 | TINF2 | 0.013476 |
| ENSSSCG00000026893 | NA | 0.013527 |
| ENSSSCG00000005235 | KANK1 | 0.013537 |
| ENSSSCG00000033591 | CHD9 | 0.013537 |
| ENSSSCG00000005706 | ABL1 | 0.013543 |
| ENSSSCG00000016173 | ATIC | 0.013569 |
| ENSSSCG00000037719 | PDPK1 | 0.013603 |
| ENSSSCG00000001578 | RNF8 | 0.013644 |
| ENSSSCG00000027646 | TIPARP | 0.013696 |
| ENSSSCG00000015255 | IGSF9B | 0.013779 |
| ENSSSCG00000010016 | MORC2 | 0.013899 |
| ENSSSCG00000024108 | SLC43A2 | 0.013959 |
| ENSSSCG00000039821 | GPRIN3 | 0.013959 |
| ENSSSCG00000002135 | PNP | 0.013997 |
| ENSSSCG00000012134 | PIGA | 0.014007 |
| ENSSSCG00000031287 | NA | 0.014048 |
| ENSSSCG00000017865 | CTNS | 0.014081 |
| ENSSSCG00000038051 | ZNFX1-AS1_2 | 0.014105 |
| ENSSSCG00000009999 | NA | 0.014128 |
| ENSSSCG00000015882 | BAZ2B | 0.014177 |
| ENSSSCG00000003155 | PPP1R15A | 0.014225 |
| ENSSSCG00000001549 | FKBP5 | 0.014239 |
| ENSSSCG00000027898 | ATP2B1 | 0.014275 |
| ENSSSCG00000004996 | KLHL28 | 0.014291 |
| ENSSSCG00000036064 | CALHM6 | 0.014468 |
| ENSSSCG00000032632 | CMTM4 | 0.014468 |
| ENSSSCG00000021814 | ZMYM2 | 0.014596 |
| ENSSSCG00000025440 | NA | 0.014605 |
| ENSSSCG00000015872 | GPD2 | 0.014605 |
| ENSSSCG00000011104 | CUL2 | 0.014613 |
| ENSSSCG00000003914 | NA | 0.014613 |
| ENSSSCG00000012252 | DDX3X | 0.014613 |
| ENSSSCG00000004687 | B2M | 0.014651 |
| ENSSSCG00000027525 | DHCR24 | 0.014995 |
| ENSSSCG00000031600 | SYNGR2 | 0.015021 |
| ENSSSCG00000016566 | NRF1 | 0.015122 |
| ENSSSCG00000001849 | ANPEP | 0.015255 |
| ENSSSCG00000011567 | NA | 0.015277 |
| ENSSSCG00000003153 | FTL | 0.015407 |
| ENSSSCG00000009047 | SMARCA5 | 0.015509 |
| ENSSSCG00000003091 | IRF2BP1 | 0.015669 |
| ENSSSCG00000008348 | PLEK | 0.015693 |
| ENSSSCG00000022833 | FGF16 | 0.015727 |
| ENSSSCG00000010798 | GGA2 | 0.015818 |
| ENSSSCG00000020783 | SLC41A1 | 0.016192 |
| ENSSSCG00000036446 | PALD1 | 0.016212 |
| ENSSSCG00000034757 | FGF11 | 0.01627 |
| ENSSSCG00000004109 | ZC3H12D | 0.016277 |
| ENSSSCG00000028878 | BCAR1 | 0.016297 |
| ENSSSCG00000034090 | CRTC3 | 0.01638 |
| ENSSSCG00000036868 | CTNNBIP1 | 0.016382 |
| ENSSSCG00000031925 | SHANK3 | 0.016614 |
| ENSSSCG00000037910 | DUSP6 | 0.016614 |
| ENSSSCG00000036584 | HAUS8 | 0.016625 |
| ENSSSCG00000017543 | CALCOCO2 | 0.017009 |
| ENSSSCG00000006319 | POGK | 0.017076 |
| ENSSSCG00000021361 | ZNF710 | 0.017098 |
| ENSSSCG00000015874 | ACVR1 | 0.017098 |
| ENSSSCG00000021885 | MDFIC | 0.01719 |
| ENSSSCG00000017573 | XYLT2 | 0.01719 |
| ENSSSCG00000011326 | PTH1R | 0.017274 |
| ENSSSCG00000006187 | MSC | 0.017274 |
| ENSSSCG00000013754 | CACNA1A | 0.017274 |
| ENSSSCG00000017137 | METRNL | 0.017317 |
| ENSSSCG00000023004 | FZD9 | 0.017339 |
| ENSSSCG00000008641 | ADAM17 | 0.017354 |
| ENSSSCG00000016690 | CREB5 | 0.017354 |
| ENSSSCG00000009000 | NA | 0.017442 |
| ENSSSCG00000008574 | KIF3C | 0.017458 |
| ENSSSCG00000009653 | CDCA2 | 0.017589 |
| ENSSSCG00000005361 | ALDH1B1 | 0.017589 |
| ENSSSCG00000013074 | RAB3IL1 | 0.01764 |
| ENSSSCG00000011678 | NA | 0.01764 |
| ENSSSCG00000011504 | EOGT | 0.01764 |
| ENSSSCG00000000171 | CKAP4 | 0.017657 |
| ENSSSCG00000013298 | PDHX | 0.017941 |
| ENSSSCG00000033324 | SPATA2 | 0.018113 |
| ENSSSCG00000007206 | RBCK1 | 0.018312 |
| ENSSSCG00000033402 | SARAF | 0.018393 |
| ENSSSCG00000015598 | TMEM206 | 0.018531 |
| ENSSSCG00000028855 | GMPS | 0.018612 |
| ENSSSCG00000024598 | PRKAA1 | 0.018612 |
| ENSSSCG00000024344 | CCR5 | 0.018795 |
| ENSSSCG00000025588 | FJX1 | 0.01888 |
| ENSSSCG00000021443 | SGK1 | 0.01888 |
| ENSSSCG00000031342 | NA | 0.01888 |
| ENSSSCG00000021803 | RANBP10 | 0.01906 |
| ENSSSCG00000034015 | NA | 0.01907 |
| ENSSSCG00000009720 | DDX60 | 0.01907 |
| ENSSSCG00000038549 | ZFP36L2 | 0.01907 |
| ENSSSCG00000035954 | ATAD5 | 0.019073 |
| ENSSSCG00000007618 | ZNF789 | 0.019103 |
| ENSSSCG00000016549 | MKLN1 | 0.019145 |
| ENSSSCG00000009152 | SGMS2 | 0.019251 |
| ENSSSCG00000034031 | ZNF16 | 0.019257 |
| ENSSSCG00000016983 | STC2 | 0.019279 |
| ENSSSCG00000008055 | TEDC2 | 0.01941 |
| ENSSSCG00000015499 | RABGAP1L | 0.01941 |
| ENSSSCG00000031898 | CALHM5 | 0.01957 |
| ENSSSCG00000012686 | MOSPD1 | 0.019609 |
| ENSSSCG00000014146 | RASA1 | 0.019609 |
| ENSSSCG00000000033 | TSPO | 0.019641 |
| ENSSSCG00000007993 | CAPN15 | 0.019726 |
| ENSSSCG00000002434 | NRDE2 | 0.019737 |
| ENSSSCG00000023618 | FRMD7 | 0.019871 |
| ENSSSCG00000032962 | ZNF664 | 0.019874 |
| ENSSSCG00000011978 | CPOX | 0.019874 |
| ENSSSCG00000025770 | ST6GAL1 | 0.019874 |
| ENSSSCG00000029662 | RASSF4 | 0.019894 |
| ENSSSCG00000025021 | NA | 0.019936 |
| ENSSSCG00000033049 | KLHL41 | 0.020057 |
| ENSSSCG00000009833 | SH2B3 | 0.020067 |
| ENSSSCG00000038492 | FAM109B | 0.020215 |
| ENSSSCG00000038594 | SDC4 | 0.020229 |
| ENSSSCG00000026326 | CCNF | 0.020418 |
| ENSSSCG00000008772 | RELL1 | 0.020639 |
| ENSSSCG00000021791 | SENP7 | 0.020695 |
| ENSSSCG00000006873 | FRRS1 | 0.020695 |
| ENSSSCG00000003687 | EPB41L3 | 0.020868 |
| ENSSSCG00000035051 | ADORA2B | 0.020868 |
| ENSSSCG00000007155 | C20orf194 | 0.021027 |
| ENSSSCG00000000806 | SCAF11 | 0.021081 |
| ENSSSCG00000016900 | ESM1 | 0.021114 |
| ENSSSCG00000025836 | SULT1C4 | 0.021144 |
| ENSSSCG00000001561 | ETV7 | 0.021144 |
| ENSSSCG00000025326 | TMEM243 | 0.021282 |
| ENSSSCG00000037452 | HTR2A | 0.021313 |
| ENSSSCG00000004387 | FOXO3 | 0.021354 |
| ENSSSCG00000038965 | ARC | 0.021361 |
| ENSSSCG00000001075 | NA | 0.021455 |
| ENSSSCG00000000383 | RNF41 | 0.021486 |
| ENSSSCG00000006358 | NDUFS2 | 0.02161 |
| ENSSSCG00000015301 | STEAP1 | 0.021687 |
| ENSSSCG00000005981 | FBXO32 | 0.02173 |
| ENSSSCG00000001073 | TPMT | 0.021808 |
| ENSSSCG00000030123 | CDS2 | 0.021956 |
| ENSSSCG00000036083 | NA | 0.022022 |
| ENSSSCG00000001579 | CMTR1 | 0.022056 |
| ENSSSCG00000035693 | POLRMT | 0.022123 |
| ENSSSCG00000000661 | PHC1 | 0.022213 |
| ENSSSCG00000010816 | TGFB2 | 0.022261 |
| ENSSSCG00000021598 | EVA1C | 0.0223 |
| ENSSSCG00000020963 | EPDR1 | 0.022369 |
| ENSSSCG00000006088 | SDC2 | 0.022384 |
| ENSSSCG00000004576 | RORA | 0.022384 |
| ENSSSCG00000016420 | INSIG1 | 0.022399 |
| ENSSSCG00000024174 | TGIF1 | 0.022468 |
| ENSSSCG00000036775 | SLC48A1 | 0.022675 |
| ENSSSCG00000007286 | ACSS2 | 0.022675 |
| ENSSSCG00000040595 | BRAP | 0.022675 |
| ENSSSCG00000038384 | COX4I2 | 0.023052 |
| ENSSSCG00000038535 | ARSB | 0.02309 |
| ENSSSCG00000001695 | VEGFA | 0.02318 |
| ENSSSCG00000000108 | TMEM184B | 0.023242 |
| ENSSSCG00000015537 | XPR1 | 0.023335 |
| ENSSSCG00000035958 | EVA1A | 0.023375 |
| ENSSSCG00000029771 | NA | 0.023434 |
| ENSSSCG00000038460 | FOXF1 | 0.023508 |
| ENSSSCG00000001201 | ZKSCAN8 | 0.023531 |
| ENSSSCG00000032984 | BEND7 | 0.023588 |
| ENSSSCG00000017504 | CDK12 | 0.023648 |
| ENSSSCG00000009060 | NA | 0.023648 |
| ENSSSCG00000033453 | BST2 | 0.023687 |
| ENSSSCG00000009278 | FGF9 | 0.023691 |
| ENSSSCG00000003451 | NA | 0.023752 |
| ENSSSCG00000014997 | NA | 0.024052 |
| ENSSSCG00000024570 | KDM4B | 0.024201 |
| ENSSSCG00000030888 | FADD | 0.02432 |
| ENSSSCG00000037556 | COG5 | 0.024325 |
| ENSSSCG00000004218 | RSPO3 | 0.024383 |
| ENSSSCG00000007878 | PARN | 0.024406 |
| ENSSSCG00000005506 | MEGF9 | 0.024444 |
| ENSSSCG00000014558 | SIRT3 | 0.024444 |
| ENSSSCG00000004845 | FAN1 | 0.024541 |
| ENSSSCG00000039684 | PGPEP1 | 0.024593 |
| ENSSSCG00000009664 | PTK2B | 0.024593 |
| ENSSSCG00000025729 | IRS1 | 0.02491 |
| ENSSSCG00000029196 | DIP2B | 0.025131 |
| ENSSSCG00000029715 | OLFM1 | 0.025192 |
| ENSSSCG00000024813 | MAP3K3 | 0.025214 |
| ENSSSCG00000023619 | SLC12A9 | 0.025665 |
| ENSSSCG00000015649 | DYRK3 | 0.025732 |
| ENSSSCG00000024578 | PHLDB1 | 0.02584 |
| ENSSSCG00000028879 | PLOD3 | 0.025896 |
| ENSSSCG00000029359 | PHLDA3 | 0.025926 |
| ENSSSCG00000015742 | TMEM177 | 0.025926 |
| ENSSSCG00000023680 | MEPCE | 0.026024 |
| ENSSSCG00000024015 | FADS1 | 0.026416 |
| ENSSSCG00000012911 | CARNS1 | 0.02653 |
| ENSSSCG00000015222 | PUS3 | 0.02653 |
| ENSSSCG00000025194 | ZSWIM6 | 0.02653 |
| ENSSSCG00000016782 | FAM105A | 0.026718 |
| ENSSSCG00000010531 | R3HCC1L | 0.026787 |
| ENSSSCG00000023126 | NA | 0.026827 |
| ENSSSCG00000026169 | MFSD12 | 0.026995 |
| ENSSSCG00000020858 | KIF13A | 0.027158 |
| ENSSSCG00000003680 | RALBP1 | 0.0273 |
| ENSSSCG00000009298 | CDK8 | 0.02736 |
| ENSSSCG00000008478 | SOS1 | 0.02736 |
| ENSSSCG00000039317 | SLC25A21 | 0.027616 |
| ENSSSCG00000036693 | NA | 0.027663 |
| ENSSSCG00000016206 | CNPPD1 | 0.027674 |
| ENSSSCG00000011961 | TFG | 0.027674 |
| ENSSSCG00000012076 | MX2 | 0.027799 |
| ENSSSCG00000000436 | PIP4K2C | 0.02785 |
| ENSSSCG00000011933 | NECTIN3 | 0.02785 |
| ENSSSCG00000002507 | BCL11B | 0.027908 |
| ENSSSCG00000006663 | OTUD7B | 0.027908 |
| ENSSSCG00000016929 | PDE4D | 0.028022 |
| ENSSSCG00000023593 | AFF1 | 0.028029 |
| ENSSSCG00000011133 | PFKFB3 | 0.028134 |
| ENSSSCG00000008218 | RNF103 | 0.028217 |
| ENSSSCG00000038967 | NADK | 0.02845 |
| ENSSSCG00000000087 | TAB1 | 0.028671 |
| ENSSSCG00000039997 | ZNF41 | 0.028671 |
| ENSSSCG00000004921 | NA | 0.028671 |
| ENSSSCG00000014338 | HSPA9 | 0.028671 |
| ENSSSCG00000002451 | RIN3 | 0.028671 |
| ENSSSCG00000028679 | NA | 0.028671 |
| ENSSSCG00000029165 | DOK4 | 0.028863 |
| ENSSSCG00000000773 | TUBA8 | 0.028961 |
| ENSSSCG00000007314 | NA | 0.029031 |
| ENSSSCG00000007143 | MAVS | 0.029134 |
| ENSSSCG00000024385 | RNASEH2B | 0.029158 |
| ENSSSCG00000011120 | USP6NL | 0.029226 |
| ENSSSCG00000017745 | UTP6 | 0.029309 |
| ENSSSCG00000028983 | TBC1D1 | 0.029658 |
| ENSSSCG00000003757 | SSX2IP | 0.029849 |
| ENSSSCG00000027480 | KLF10 | 0.029941 |
| ENSSSCG00000010719 | NA | 0.030255 |
| ENSSSCG00000026590 | HGH1 | 0.030315 |
| ENSSSCG00000009971 | ZNRF3 | 0.030347 |
| ENSSSCG00000037549 | GCLM | 0.030364 |
| ENSSSCG00000003079 | NA | 0.030397 |
| ENSSSCG00000009145 | OSTC | 0.030468 |
| ENSSSCG00000014214 | TRIM36 | 0.030651 |
| ENSSSCG00000009129 | TIFA | 0.030787 |
| ENSSSCG00000015979 | HOXD13 | 0.030838 |
| ENSSSCG00000012474 | DIAPH2 | 0.030838 |
| ENSSSCG00000016520 | CREB3L2 | 0.030848 |
| ENSSSCG00000012713 | ATP11C | 0.031124 |
| ENSSSCG00000009946 | SART3 | 0.031124 |
| ENSSSCG00000034551 | SCX | 0.031129 |
| ENSSSCG00000015569 | SWT1 | 0.031194 |
| ENSSSCG00000038969 | DMPK | 0.031332 |
| ENSSSCG00000021874 | UNC5C | 0.031534 |
| ENSSSCG00000033641 | COL8A2 | 0.031534 |
| ENSSSCG00000005944 | NDRG1 | 0.031571 |
| ENSSSCG00000016725 | NA | 0.031652 |
| ENSSSCG00000028036 | MAP3K10 | 0.031684 |
| ENSSSCG00000008729 | LYAR | 0.031693 |
| ENSSSCG00000033272 | DNAJB6 | 0.031693 |
| ENSSSCG00000025298 | TWISTNB | 0.031695 |
| ENSSSCG00000010928 | KDM5B | 0.031795 |
| ENSSSCG00000016701 | HOXA7 | 0.031926 |
| ENSSSCG00000014878 | PAK1 | 0.031926 |
| ENSSSCG00000007789 | NA | 0.031979 |
| ENSSSCG00000017525 | NFE2L1 | 0.031979 |
| ENSSSCG00000006194 | NCOA2 | 0.03228 |
| ENSSSCG00000033410 | FAM212A | 0.032393 |
| ENSSSCG00000040732 | WDR81 | 0.032399 |
| ENSSSCG00000026943 | MRAP2 | 0.032399 |
| ENSSSCG00000038505 | MSI2 | 0.032559 |
| ENSSSCG00000012366 | LAS1L | 0.032657 |
| ENSSSCG00000023140 | EIF2B4 | 0.032733 |
| ENSSSCG00000036742 | KLF15 | 0.032756 |
| ENSSSCG00000003402 | PGD | 0.032756 |
| ENSSSCG00000022446 | SEL1L3 | 0.032756 |
| ENSSSCG00000000774 | USP18 | 0.032796 |
| ENSSSCG00000003580 | EYA3 | 0.033163 |
| ENSSSCG00000015052 | USP28 | 0.033329 |
| ENSSSCG00000000160 | PRDM4 | 0.033336 |
| ENSSSCG00000011698 | GYG1 | 0.033472 |
| ENSSSCG00000030801 | NA | 0.033756 |
| ENSSSCG00000013256 | ARHGAP1 | 0.033776 |
| ENSSSCG00000017548 | NGFR | 0.03387 |
| ENSSSCG00000005376 | TBC1D2 | 0.034199 |
| ENSSSCG00000012968 | CCDC85B | 0.034205 |
| ENSSSCG00000030211 | NBR1 | 0.034255 |
| ENSSSCG00000028973 | PHACTR4 | 0.034345 |
| ENSSSCG00000011437 | ALAS1 | 0.034447 |
| ENSSSCG00000008624 | LPIN1 | 0.034608 |
| ENSSSCG00000039761 | MYCL | 0.034623 |
| ENSSSCG00000027529 | BIRC3 | 0.034801 |
| ENSSSCG00000002799 | CNOT1 | 0.034902 |
| ENSSSCG00000033998 | SLC43A3 | 0.034984 |
| ENSSSCG00000024604 | ATAT1 | 0.034984 |
| ENSSSCG00000008812 | ATP10D | 0.034984 |
| ENSSSCG00000004551 | ZNF609 | 0.034984 |
| ENSSSCG00000016755 | POLM | 0.034984 |
| ENSSSCG00000010138 | ZDHHC8 | 0.035148 |
| ENSSSCG00000034072 | NA | 0.035505 |
| ENSSSCG00000037274 | NA | 0.035505 |
| ENSSSCG00000015570 | IVNS1ABP | 0.035526 |
| ENSSSCG00000021161 | CKS2 | 0.035692 |
| ENSSSCG00000006728 | GDAP2 | 0.035723 |
| ENSSSCG00000028974 | UST | 0.035891 |
| ENSSSCG00000039045 | SLC26A2 | 0.035998 |
| ENSSSCG00000015576 | TPR | 0.036071 |
| ENSSSCG00000006482 | MEF2D | 0.036071 |
| ENSSSCG00000015650 | MAPKAPK2 | 0.036071 |
| ENSSSCG00000003815 | ALG6 | 0.036191 |
| ENSSSCG00000013248 | LRP4 | 0.036249 |
| ENSSSCG00000026302 | NA | 0.036396 |
| ENSSSCG00000032203 | EPPK1 | 0.036618 |
| ENSSSCG00000031565 | NA | 0.036618 |
| ENSSSCG00000011877 | CD86 | 0.036618 |
| ENSSSCG00000017927 | BCL6B | 0.036714 |
| ENSSSCG00000003428 | MTHFR | 0.036714 |
| ENSSSCG00000028696 | FEN1 | 0.037175 |
| ENSSSCG00000009620 | BMP1 | 0.037316 |
| ENSSSCG00000003512 | EIF4G3 | 0.037361 |
| ENSSSCG00000008125 | NCAPH | 0.037734 |
| ENSSSCG00000016233 | SERPINE2 | 0.03774 |
| ENSSSCG00000040352 | DUS1L | 0.037767 |
| ENSSSCG00000009178 | H2AFZ | 0.037767 |
| ENSSSCG00000031875 | ZNF469 | 0.037776 |
| ENSSSCG00000012056 | CHAF1B | 0.037804 |
| ENSSSCG00000040288 | ARNT2 | 0.037804 |
| ENSSSCG00000037950 | NIPAL1 | 0.038162 |
| ENSSSCG00000030408 | DDX58 | 0.038162 |
| ENSSSCG00000021784 | TBCK | 0.038162 |
| ENSSSCG00000010128 | SEPT5 | 0.038162 |
| ENSSSCG00000031594 | NA | 0.03872 |
| ENSSSCG00000011065 | MASTL | 0.038725 |
| ENSSSCG00000009045 | HHIP | 0.038725 |
| ENSSSCG00000039703 | EEPD1 | 0.038725 |
| ENSSSCG00000032529 | NA | 0.038732 |
| ENSSSCG00000033993 | PLCXD3 | 0.038756 |
| ENSSSCG00000020856 | PPME1 | 0.038764 |
| ENSSSCG00000036047 | THG1L | 0.038776 |
| ENSSSCG00000002009 | PCK2 | 0.039043 |
| ENSSSCG00000009331 | MEDAG | 0.039055 |
| ENSSSCG00000028345 | NA | 0.039055 |
| ENSSSCG00000004379 | SOBP | 0.039228 |
| ENSSSCG00000008423 | PPP1R21 | 0.039581 |
| ENSSSCG00000002023 | PABPN1 | 0.039581 |
| ENSSSCG00000010794 | PALB2 | 0.039581 |
| ENSSSCG00000002425 | PTPN21 | 0.039581 |
| ENSSSCG00000002344 | RIOX1 | 0.039581 |
| ENSSSCG00000007709 | TBL2 | 0.039581 |
| ENSSSCG00000032062 | WDR55 | 0.039691 |
| ENSSSCG00000008292 | TET3 | 0.039691 |
| ENSSSCG00000032645 | CHIC2 | 0.039884 |
| ENSSSCG00000025174 | WDFY1 | 0.03989 |
| ENSSSCG00000010468 | CPEB3 | 0.039921 |
| ENSSSCG00000037847 | MOB2 | 0.039921 |
| ENSSSCG00000024672 | KLF16 | 0.039935 |
| ENSSSCG00000010357 | WAPL | 0.03996 |
| ENSSSCG00000008799 | LIMCH1 | 0.03996 |
| ENSSSCG00000029547 | NAB1 | 0.040129 |
| ENSSSCG00000024555 | ING2 | 0.040232 |
| ENSSSCG00000012656 | ELF4 | 0.040232 |
| ENSSSCG00000010006 | NA | 0.04036 |
| ENSSSCG00000008981 | NA | 0.040741 |
| ENSSSCG00000001011 | SERPINB1 | 0.040883 |
| ENSSSCG00000023231 | RBM22 | 0.040956 |
| ENSSSCG00000026863 | FARP1 | 0.040956 |
| ENSSSCG00000003488 | UBR4 | 0.040956 |
| ENSSSCG00000008176 | LONRF2 | 0.041093 |
| ENSSSCG00000023761 | HNRNPR | 0.041093 |
| ENSSSCG00000006845 | STXBP3 | 0.041698 |
| ENSSSCG00000015496 | CENPL | 0.041925 |
| ENSSSCG00000006571 | INTS3 | 0.042018 |
| ENSSSCG00000022611 | CAPRIN2 | 0.042173 |
| ENSSSCG00000003167 | FLT3LG | 0.042203 |
| ENSSSCG00000004854 | NA | 0.042203 |
| ENSSSCG00000016119 | NA | 0.042556 |
| ENSSSCG00000011549 | OGG1 | 0.042556 |
| ENSSSCG00000023803 | ELK3 | 0.042704 |
| ENSSSCG00000005711 | NUP214 | 0.042752 |
| ENSSSCG00000039545 | NA | 0.042752 |
| ENSSSCG00000021698 | RRP1B | 0.042981 |
| ENSSSCG00000039311 | NCS1 | 0.042985 |
| ENSSSCG00000035403 | RFX2 | 0.04323 |
| ENSSSCG00000032531 | SMG1 | 0.04323 |
| ENSSSCG00000011081 | NA | 0.04323 |
| ENSSSCG00000004569 | LACTB | 0.04323 |
| ENSSSCG00000004826 | SELENOS | 0.04324 |
| ENSSSCG00000028504 | RFC2 | 0.043428 |
| ENSSSCG00000012077 | MX1 | 0.043482 |
| ENSSSCG00000002703 | NA | 0.043734 |
| ENSSSCG00000008144 | NCK2 | 0.044031 |
| ENSSSCG00000012307 | CCNB3 | 0.04426 |
| ENSSSCG00000028322 | BTG2 | 0.044528 |
| ENSSSCG00000015407 | GNAI1 | 0.044528 |
| ENSSSCG00000029593 | FOXN2 | 0.04457 |
| ENSSSCG00000007839 | EEF2K | 0.044686 |
| ENSSSCG00000040943 | DCTN5 | 0.044756 |
| ENSSSCG00000012889 | CHKA | 0.044791 |
| ENSSSCG00000009739 | NOC4L | 0.044805 |
| ENSSSCG00000025981 | SLC38A5 | 0.04485 |
| ENSSSCG00000011471 | FLNB | 0.044983 |
| ENSSSCG00000008858 | KLHL2 | 0.044983 |
| ENSSSCG00000035863 | PLIN2 | 0.045015 |
| ENSSSCG00000008479 | DHX57 | 0.045015 |
| ENSSSCG00000009051 | IL15 | 0.045074 |
| ENSSSCG00000002276 | PLEKHG3 | 0.045144 |
| ENSSSCG00000008422 | NA | 0.045441 |
| ENSSSCG00000037779 | CAPN10 | 0.045506 |
| ENSSSCG00000036155 | FAT4 | 0.045872 |
| ENSSSCG00000009473 | MYCBP2 | 0.04588 |
| ENSSSCG00000010005 | RNF215 | 0.04591 |
| ENSSSCG00000016679 | NA | 0.04591 |
| ENSSSCG00000032914 | MANF | 0.046311 |
| ENSSSCG00000040796 | IPP | 0.046462 |
| ENSSSCG00000014224 | SEMA6A | 0.046465 |
| ENSSSCG00000038296 | NA | 0.046608 |
| ENSSSCG00000017349 | ADAM11 | 0.04705 |
| ENSSSCG00000005643 | NA | 0.047063 |
| ENSSSCG00000007337 | CTNNBL1 | 0.047063 |
| ENSSSCG00000026733 | HIPK2 | 0.047088 |
| ENSSSCG00000021576 | CD83 | 0.047264 |
| ENSSSCG00000005967 | FAM84B | 0.047374 |
| ENSSSCG00000015859 | SAP130 | 0.047433 |
| ENSSSCG00000033178 | NA | 0.047496 |
| ENSSSCG00000010248 | KIF1BP | 0.04759 |
| ENSSSCG00000004435 | NT5DC1 | 0.04762 |
| ENSSSCG00000038940 | GNPDA1 | 0.047679 |
| ENSSSCG00000037832 | PMP22 | 0.047725 |
| ENSSSCG00000036975 | PSRC1 | 0.047773 |
| ENSSSCG00000006236 | NSMAF | 0.047773 |
| ENSSSCG00000027127 | MAP2K2 | 0.048249 |
| ENSSSCG00000010085 | SDF2L1 | 0.048249 |
| ENSSSCG00000039594 | SSBP3 | 0.048381 |
| ENSSSCG00000027007 | NA | 0.048388 |
| ENSSSCG00000017733 | C17orf75 | 0.048419 |
| ENSSSCG00000028979 | UVRAG | 0.048956 |
| ENSSSCG00000006809 | RBM15 | 0.049119 |
| ENSSSCG00000032269 | LIMA1 | 0.049191 |
| ENSSSCG00000006742 | MAB21L3 | 0.04935 |
| ENSSSCG00000012746 | NA | 0.049828 |
| ENSSSCG00000040828 | NA | 0.049839 |
| ENSSSCG00000026617 | EIF2S2 | 0.049955 |
| ENSSSCG00000039663 | TWSG1 | 0.049964 |
| ENSSSCG00000017473 | TOP2A | 0.049964 |

Gene Name “NA” indicates the gene ID was not matched to a HGNC gene name.
