# Supplementary material for: A Tale of Two Loads: Modulation of IL-1 Induced Inflammatory Responses of Meniscal Cells in Two Models of Dynamic Physiologic Loading
Source: Front Bioeng Biotechnol. 2022 Mar 1;10:837619. doi: 10.3389/fbioe.2022.837619 (PMC8921261; doi:10.3389/fbioe.2022.837619)
Supplement: Supplementary file 8 [file DataSheet9.DOCX]

**Supplemental Table 10**: 10% compression compared to 0% compression for inner zone tissue without exogenous IL-1α stimulation.

| **Gene ID** | **Gene Name** | **Log2Fold Change** | **p-value** | **Up/Down Regulated** |
| --- | --- | --- | --- | --- |
| ENSSSCG00000034114 | GPR68 | 2.151931 | 2.32E-05 | UP |
| ENSSSCG00000009334 | HSPH1 | 1.080116 | 0.000226 | UP |
| ENSSSCG00000034167 | SLC5A3 | 1.845208 | 0.000273 | UP |
| ENSSSCG00000011643 | AMOTL2 | 1.212232 | 0.000298 | UP |
| ENSSSCG00000027607 | IER3 | 1.149959 | 0.000314 | UP |
| ENSSSCG00000011973 | COL8A1 | 2.732043 | 0.000497 | UP |
| ENSSSCG00000032367 | CEBPD | 1.086106 | 0.001607 | UP |
| ENSSSCG00000008959 | CXCL2 | 2.699562 | 0.009761 | UP |
| ENSSSCG00000034802 | NA | 1.263669 | 0.013886 | UP |
| ENSSSCG00000034449 | RSRP1 | 1.553788 | 0.013886 | UP |
| ENSSSCG00000011195 | GALNT15 | 1.368847 | 0.01531 | UP |
| ENSSSCG00000040961 | LIF | 2.051259 | 0.019702 | UP |
| ENSSSCG00000008953 | CXCL8 | 3.50378 | 0.019775 | UP |
| ENSSSCG00000002135 | PNP | 1.311111 | 0.020527 | UP |
| ENSSSCG00000012084 | PRDM15 | 1.590504 | 0.031084 | UP |
| ENSSSCG00000008954 | NA | 2.20468 | 0.038225 | UP |
| ENSSSCG00000008957 | AMCF-II | 3.437726 | 0.040622 | UP |
| ENSSSCG00000037572 | EPSTI1 | 1.374897 | 0.040622 | UP |
| ENSSSCG00000004241 | GJA1 | 1.266081 | 0.041503 | UP |
| ENSSSCG00000005503 | TLR4 | 1.15292 | 0.043825 | UP |
| ENSSSCG00000016254 | CCL20 | 1.678721 | 0.044318 | UP |
| ENSSSCG00000020970 | IL6 | 2.487124 | 0.045624 | UP |
| ENSSSCG00000013427 | CIRBP | -1.42413 | 5.12E-07 | DOWN |
| ENSSSCG00000023871 | NA | -1.84317 | 4.60E-05 | DOWN |
| ENSSSCG00000009973 | RHBDD3 | -1.16763 | 0.000845 | DOWN |
| ENSSSCG00000004209 | PTPRK | -1.61867 | 0.001342 | DOWN |
| ENSSSCG00000027415 | WWOX | -1.49188 | 0.002307 | DOWN |
| ENSSSCG00000016720 | PGAM2 | -1.63604 | 0.004587 | DOWN |
| ENSSSCG00000010464 | PPP1R3C | -1.29518 | 0.005712 | DOWN |
| ENSSSCG00000027144 | LMNTD1 | -1.24145 | 0.013886 | DOWN |
| ENSSSCG00000033268 | CABCOCO1 | -1.17806 | 0.01531 | DOWN |
| ENSSSCG00000021749 | MCF2L2 | -1.23575 | 0.023238 | DOWN |
| ENSSSCG00000013933 | PBX4 | -1.9426 | 0.031084 | DOWN |
| ENSSSCG00000022322 | BCL2L11 | -1.22892 | 0.039849 | DOWN |
| ENSSSCG00000040603 | SGTB | -1.47255 | 0.041503 | DOWN |

Gene Name “NA” indicates the gene ID was not matched to a HGNC gene name.
