# Supplementary material for: A Tale of Two Loads: Modulation of IL-1 Induced Inflammatory Responses of Meniscal Cells in Two Models of Dynamic Physiologic Loading
Source: Front Bioeng Biotechnol. 2022 Mar 1;10:837619. doi: 10.3389/fbioe.2022.837619 (PMC8921261; doi:10.3389/fbioe.2022.837619)
Supplement: Supplementary file 9 [file DataSheet5.DOCX]

**Supplemental Table 6**: 5% stretch compared to 0% stretch for inner zone cells without exogenous IL-1α stimulation.

| **Gene ID** | **Gene Name** | **Log2Fold Change** | **p-value** | **Up/Down Regulated** |
| --- | --- | --- | --- | --- |
| ENSSSCG00000038521 | CHAC1 | 2.291348 | 3.28E-127 | UP |
| ENSSSCG00000007477 | NFATC2 | 1.479733 | 4.44E-119 | UP |
| ENSSSCG00000034114 | GPR68 | 1.371853 | 4.70E-112 | UP |
| ENSSSCG00000010329 | ZMIZ1 | 1.739065 | 1.27E-110 | UP |
| ENSSSCG00000039862 | TRIB3 | 1.941874 | 1.88E-62 | UP |
| ENSSSCG00000013599 | ANGPTL4 | 1.292785 | 4.64E-62 | UP |
| ENSSSCG00000008957 | AMCF-II | 1.598791 | 1.22E-56 | UP |
| ENSSSCG00000012026 | ADAMTS1 | 1.371741 | 1.52E-50 | UP |
| ENSSSCG00000006917 | NA | 1.223802 | 1.33E-48 | UP |
| ENSSSCG00000036060 | RRAD | 1.934305 | 7.00E-45 | UP |
| ENSSSCG00000036893 | PTHLH | 1.488278 | 1.85E-43 | UP |
| ENSSSCG00000000837 | CHST11 | 1.096534 | 4.71E-43 | UP |
| ENSSSCG00000026006 | KLF13 | 1.051709 | 6.25E-42 | UP |
| ENSSSCG00000007079 | FLRT3 | 1.128265 | 7.62E-38 | UP |
| ENSSSCG00000017142 | BAIAP2 | 1.047557 | 4.45E-35 | UP |
| ENSSSCG00000038965 | ARC | 1.95323 | 5.96E-31 | UP |
| ENSSSCG00000021569 | MMP25 | 1.186148 | 4.31E-30 | UP |
| ENSSSCG00000021576 | CD83 | 1.33446 | 1.08E-29 | UP |
| ENSSSCG00000018047 | FAM83G | 2.380397 | 1.27E-28 | UP |
| ENSSSCG00000014437 | PPARGC1B | 1.444519 | 1.37E-28 | UP |
| ENSSSCG00000009320 | FLT1 | 2.036205 | 3.44E-25 | UP |
| ENSSSCG00000008953 | CXCL8 | 2.158647 | 3.19E-24 | UP |
| ENSSSCG00000008959 | CXCL2 | 1.292854 | 4.33E-24 | UP |
| ENSSSCG00000035403 | RFX2 | 1.361735 | 2.20E-23 | UP |
| ENSSSCG00000012741 | NA | 1.02926 | 3.48E-23 | UP |
| ENSSSCG00000005385 | NR4A3 | 1.718584 | 6.16E-22 | UP |
| ENSSSCG00000029592 | GPRC5A | 1.37573 | 3.23E-21 | UP |
| ENSSSCG00000038185 | EREG | 1.308985 | 8.54E-21 | UP |
| ENSSSCG00000012034 | TIAM1 | 1.183981 | 2.08E-19 | UP |
| ENSSSCG00000033465 | HIVEP3 | 1.410939 | 8.70E-18 | UP |
| ENSSSCG00000005992 | SHAS2 | 1.481584 | 2.49E-17 | UP |
| ENSSSCG00000034858 | RAP1GAP2 | 1.213088 | 4.04E-17 | UP |
| ENSSSCG00000029656 | NDP | 1.472164 | 5.87E-17 | UP |
| ENSSSCG00000015085 | IL10RA | 1.19254 | 5.72E-16 | UP |
| ENSSSCG00000012241 | NA | 1.026417 | 3.18E-15 | UP |
| ENSSSCG00000016900 | ESM1 | 1.243573 | 3.39E-15 | UP |
| ENSSSCG00000040638 | DIO2 | 1.109612 | 1.42E-14 | UP |
| ENSSSCG00000008311 | CYP26B1 | 1.392792 | 1.89E-13 | UP |
| ENSSSCG00000040388 | ZBTB46 | 1.72114 | 2.24E-13 | UP |
| ENSSSCG00000007486 | CYP24A1 | 2.935038 | 7.30E-13 | UP |
| ENSSSCG00000004572 | NA | 1.043386 | 7.47E-13 | UP |
| ENSSSCG00000021598 | EVA1C | 1.243677 | 1.15E-12 | UP |
| ENSSSCG00000007682 | SH2B2 | 1.221426 | 1.24E-12 | UP |
| ENSSSCG00000033397 | KCNK9 | 2.135438 | 1.61E-12 | UP |
| ENSSSCG00000006023 | SYBU | 1.14606 | 3.84E-12 | UP |
| ENSSSCG00000031023 | NA | 1.202189 | 6.99E-12 | UP |
| ENSSSCG00000024954 | FGF1 | 1.058042 | 9.04E-11 | UP |
| ENSSSCG00000000766 | CECR2 | 1.157592 | 1.22E-10 | UP |
| ENSSSCG00000015255 | IGSF9B | 1.393409 | 1.45E-10 | UP |
| ENSSSCG00000035612 | COX6B2 | 2.502497 | 3.13E-10 | UP |
| ENSSSCG00000009580 | S1PR3 | 1.007093 | 3.20E-10 | UP |
| ENSSSCG00000001710 | RUNX2 | 1.313181 | 6.15E-10 | UP |
| ENSSSCG00000040636 | KRT80 | 1.179973 | 6.49E-10 | UP |
| ENSSSCG00000009060 | NA | 1.130784 | 7.29E-10 | UP |
| ENSSSCG00000008963 | AREG | 1.052223 | 1.34E-09 | UP |
| ENSSSCG00000002648 | CBFA2T3 | 1.075399 | 1.81E-09 | UP |
| ENSSSCG00000003702 | GATA6 | 1.171533 | 6.80E-09 | UP |
| ENSSSCG00000003256 | PRKCG | 1.271289 | 1.71E-08 | UP |
| ENSSSCG00000013236 | MYBPC3 | 1.552155 | 3.12E-08 | UP |
| ENSSSCG00000006902 | GFI1 | 1.631208 | 1.29E-07 | UP |
| ENSSSCG00000035847 | TAL1 | 1.137633 | 2.21E-07 | UP |
| ENSSSCG00000038460 | FOXF1 | 1.008382 | 3.85E-07 | UP |
| ENSSSCG00000014347 | PROB1 | 1.012232 | 7.25E-07 | UP |
| ENSSSCG00000036555 | PRDM8 | 1.166123 | 1.84E-06 | UP |
| ENSSSCG00000006742 | MAB21L3 | 1.047658 | 2.21E-06 | UP |
| ENSSSCG00000000906 | TMCC3 | 1.721052 | 2.40E-06 | UP |
| ENSSSCG00000003069 | KCNN4 | 1.198499 | 6.26E-06 | UP |
| ENSSSCG00000038126 | MGAT3 | 1.213361 | 7.83E-06 | UP |
| ENSSSCG00000012104 | NA | 1.584276 | 8.02E-06 | UP |
| ENSSSCG00000004332 | BACH2 | 2.163828 | 1.43E-05 | UP |
| ENSSSCG00000037856 | EDN2 | 2.681828 | 1.57E-05 | UP |
| ENSSSCG00000017112 | IRX4 | 1.06551 | 2.23E-05 | UP |
| ENSSSCG00000008768 | ARAP2 | 1.873628 | 3.51E-05 | UP |
| ENSSSCG00000016573 | IRF5 | 1.161714 | 6.89E-05 | UP |
| ENSSSCG00000027401 | OPRD1 | 1.01288 | 0.000107 | UP |
| ENSSSCG00000009148 | LEF1 | 1.117354 | 0.000232 | UP |
| ENSSSCG00000001597 | LRFN2 | 1.593852 | 0.000315 | UP |
| ENSSSCG00000005216 | NA | 1.149751 | 0.000344 | UP |
| ENSSSCG00000035074 | FOXO6 | 1.753875 | 0.00057 | UP |
| ENSSSCG00000014823 | P2RY6 | 2.099812 | 0.000885 | UP |
| ENSSSCG00000015839 | NA | 1.006871 | 0.00099 | UP |
| ENSSSCG00000009977 | RASL10A | 2.666556 | 0.001183 | UP |
| ENSSSCG00000028531 | SH3BP1 | 1.044198 | 0.001616 | UP |
| ENSSSCG00000008689 | ZFYVE28 | 1.124995 | 0.002143 | UP |
| ENSSSCG00000004218 | RSPO3 | 1.161224 | 0.002293 | UP |
| ENSSSCG00000022202 | TOX2 | 1.064342 | 0.002626 | UP |
| ENSSSCG00000003832 | TACSTD2 | 1.603469 | 0.003006 | UP |
| ENSSSCG00000001867 | PSTPIP1 | 1.435832 | 0.004228 | UP |
| ENSSSCG00000011721 | P2RY1 | 1.015945 | 0.004954 | UP |
| ENSSSCG00000023487 | MSLNL | 1.580108 | 0.005402 | UP |
| ENSSSCG00000009429 | TNFSF11 | 1.696769 | 0.006852 | UP |
| ENSSSCG00000029365 | MSI1 | 1.96927 | 0.009878 | UP |
| ENSSSCG00000001920 | HCN4 | 1.750036 | 0.01162 | UP |
| ENSSSCG00000002835 | TOX3 | 2.953082 | 0.012588 | UP |
| ENSSSCG00000010879 | KIF26B | 1.160783 | 0.012732 | UP |
| ENSSSCG00000038677 | GJB3 | 1.302419 | 0.012749 | UP |
| ENSSSCG00000008090 | IL1A | 3.974737 | 0.012949 | UP |
| ENSSSCG00000014259 | ADAMTS19 | 1.86409 | 0.026785 | UP |
| ENSSSCG00000008344 | ARHGAP25 | 1.403905 | 0.028382 | UP |
| ENSSSCG00000021624 | LAD1 | 1.925821 | 0.028576 | UP |
| ENSSSCG00000027365 | WNT7B | 1.124051 | 0.029506 | UP |
| ENSSSCG00000003737 | NOL4 | 3.545338 | 0.031064 | UP |
| ENSSSCG00000016550 | KLF14 | 1.783307 | 0.034201 | UP |
| ENSSSCG00000039673 | ASB16 | 1.663743 | 0.036668 | UP |
| ENSSSCG00000012780 | PLXNB3 | 1.81124 | 0.037783 | UP |
| ENSSSCG00000039608 | SNORD62 | 1.364448 | 0.038933 | UP |
| ENSSSCG00000021902 | GABRP | 1.190914 | 0.03956 | UP |
| ENSSSCG00000006383 | VANGL2 | 1.295182 | 0.039764 | UP |
| ENSSSCG00000017470 | TNS4 | 2.941658 | 0.041685 | UP |
| ENSSSCG00000015253 | NTM | 1.76429 | 0.041713 | UP |
| ENSSSCG00000008533 | ALK | 1.30706 | 0.042119 | UP |
| ENSSSCG00000009709 | MFAP3L | 1.166697 | 0.047262 | UP |
| ENSSSCG00000013432 | MIDN | -1.22407 | 2.26E-84 | DOWN |
| ENSSSCG00000027426 | BCL3 | -1.96697 | 1.28E-74 | DOWN |
| ENSSSCG00000027480 | KLF10 | -1.58789 | 3.06E-74 | DOWN |
| ENSSSCG00000004948 | SMAD6 | -1.61057 | 5.68E-63 | DOWN |
| ENSSSCG00000002392 | IRF2BPL | -1.38425 | 1.31E-57 | DOWN |
| ENSSSCG00000000411 | NAB2 | -1.01497 | 2.63E-51 | DOWN |
| ENSSSCG00000032613 | SNAI1 | -1.40301 | 1.18E-48 | DOWN |
| ENSSSCG00000025826 | BOC | -1.26772 | 7.71E-46 | DOWN |
| ENSSSCG00000036136 | BHLHE40 | -1.15547 | 5.28E-45 | DOWN |
| ENSSSCG00000037815 | ZC3H12A | -1.14978 | 7.23E-43 | DOWN |
| ENSSSCG00000039473 | NA | -1.03076 | 2.69E-40 | DOWN |
| ENSSSCG00000013735 | JUNB | -1.35209 | 8.02E-39 | DOWN |
| ENSSSCG00000011672 | RASA2 | -1.24041 | 1.90E-25 | DOWN |
| ENSSSCG00000031356 | HES1 | -1.67358 | 5.86E-25 | DOWN |
| ENSSSCG00000039568 | SNAI2 | -1.41544 | 6.41E-24 | DOWN |
| ENSSSCG00000011264 | CSRNP1 | -1.13734 | 3.10E-18 | DOWN |
| ENSSSCG00000020705 | MAP3K8 | -1.30514 | 1.07E-17 | DOWN |
| ENSSSCG00000016841 | SLC1A3 | -1.03638 | 1.82E-17 | DOWN |
| ENSSSCG00000037016 | ID1 | -1.06147 | 3.60E-16 | DOWN |
| ENSSSCG00000036755 | FAM46B | -1.40928 | 1.13E-15 | DOWN |
| ENSSSCG00000015368 | HDAC9 | -1.05441 | 4.13E-15 | DOWN |
| ENSSSCG00000037468 | GNE | -1.06936 | 4.25E-15 | DOWN |
| ENSSSCG00000031321 | NR4A1 | -1.48901 | 7.30E-13 | DOWN |
| ENSSSCG00000010224 | EGR2 | -1.32482 | 5.05E-11 | DOWN |
| ENSSSCG00000009630 | EGR3 | -1.48715 | 5.22E-10 | DOWN |
| ENSSSCG00000035971 | DUSP2 | -1.09133 | 6.85E-07 | DOWN |
| ENSSSCG00000034184 | NA | -1.79103 | 1.08E-06 | DOWN |
| ENSSSCG00000036742 | KLF15 | -2.39243 | 1.43E-06 | DOWN |
| ENSSSCG00000023178 | BATF2 | -1.72637 | 8.73E-06 | DOWN |
| ENSSSCG00000008648 | RSAD2 | -1.74015 | 1.22E-05 | DOWN |
| ENSSSCG00000026592 | TLR6 | -1.40836 | 2.52E-05 | DOWN |
| ENSSSCG00000011874 | PARP14 | -1.07491 | 0.000133 | DOWN |
| ENSSSCG00000032474 | CXCL10 | -2.01551 | 0.000244 | DOWN |
| ENSSSCG00000000791 | PDZRN4 | -1.18904 | 0.001051 | DOWN |
| ENSSSCG00000033363 | NA | -1.1723 | 0.001153 | DOWN |
| ENSSSCG00000008647 | CMPK2 | -1.01183 | 0.00117 | DOWN |
| ENSSSCG00000021997 | ALS2CL | -1.27014 | 0.002852 | DOWN |
| ENSSSCG00000039109 | MTERF2 | -1.02855 | 0.006893 | DOWN |
| ENSSSCG00000017700 | CCL3L1 | -1.32446 | 0.007101 | DOWN |
| ENSSSCG00000022045 | NA | -1.33593 | 0.014264 | DOWN |
| ENSSSCG00000026130 | EPHA3 | -1.38153 | 0.019846 | DOWN |
| ENSSSCG00000035388 | C16orf46 | -1.2979 | 0.024212 | DOWN |
| ENSSSCG00000015618 | LAMB3 | -2.66921 | 0.027999 | DOWN |
| ENSSSCG00000027206 | PARD6B | -1.42611 | 0.034547 | DOWN |
| ENSSSCG00000033768 | LAX1 | -3.5276 | 0.049845 | DOWN |

Gene Name “NA” indicates the gene ID was not matched to a HGNC gene name.
