# Supplementary material for: A Tale of Two Loads: Modulation of IL-1 Induced Inflammatory Responses of Meniscal Cells in Two Models of Dynamic Physiologic Loading
Source: Front Bioeng Biotechnol. 2022 Mar 1;10:837619. doi: 10.3389/fbioe.2022.837619 (PMC8921261; doi:10.3389/fbioe.2022.837619)
Supplement: Supplementary file 11 [file DataSheet10.DOCX]

**Supplemental Table 11**: 10% compression compared to 0% compression for inner zone tissue with exogenous IL-1α stimulation.

| **Gene ID** | **Gene Name** | **Log2Fold Change** | **p-value** | **Up/Down Regulated** |
| --- | --- | --- | --- | --- |
| ENSSSCG00000001471 | BRD2 | 1.078692 | 2.14E-05 | UP |
| ENSSSCG00000001695 | VEGFA | 1.585051 | 2.14E-05 | UP |
| ENSSSCG00000021597 | PHLDA2 | 1.168725 | 6.75E-05 | UP |
| ENSSSCG00000029228 | ZNF276 | 1.209767 | 7.69E-05 | UP |
| ENSSSCG00000012915 | CLCF1 | 1.223574 | 0.000277 | UP |
| ENSSSCG00000030182 | DEDD2 | 1.073921 | 0.000277 | UP |
| ENSSSCG00000008275 | TTC31 | 1.102931 | 0.003068 | UP |
| ENSSSCG00000007432 | ZNF335 | 1.041831 | 0.003318 | UP |
| ENSSSCG00000030424 | USP31 | 1.320472 | 0.009617 | UP |
| ENSSSCG00000008383 | NA | 1.219983 | 0.01268 | UP |
| ENSSSCG00000006518 | HCN3 | 1.188566 | 0.013537 | UP |
| ENSSSCG00000003081 | CEACAM16 | 1.807314 | 0.013898 | UP |
| ENSSSCG00000033692 | B3GALNT2 | 1.099813 | 0.014265 | UP |
| ENSSSCG00000009580 | S1PR3 | 5.362598 | 0.015208 | UP |
| ENSSSCG00000014362 | HBEGF | 1.56947 | 0.016912 | UP |
| ENSSSCG00000009616 | HR | 1.155859 | 0.017219 | UP |
| ENSSSCG00000013119 | STX3 | 1.208069 | 0.021719 | UP |
| ENSSSCG00000015287 | TMCC2 | 1.005518 | 0.021719 | UP |
| ENSSSCG00000032115 | OSGIN1 | 1.403505 | 0.024038 | UP |
| ENSSSCG00000034167 | SLC5A3 | 1.250396 | 0.024065 | UP |
| ENSSSCG00000038051 | ZNFX1-AS1_2 | 1.782442 | 0.024272 | UP |
| ENSSSCG00000030337 | NYAP1 | 1.420154 | 0.024566 | UP |
| ENSSSCG00000031219 | NAPB | 2.001657 | 0.025247 | UP |
| ENSSSCG00000034114 | GPR68 | 1.241582 | 0.025247 | UP |
| ENSSSCG00000034449 | RSRP1 | 1.266145 | 0.03047 | UP |
| ENSSSCG00000037102 | FSTL3 | 1.120071 | 0.030777 | UP |
| ENSSSCG00000006525 | NA | 4.139146 | 0.032909 | UP |
| ENSSSCG00000035987 | EHD3 | 1.431389 | 0.03364 | UP |
| ENSSSCG00000029656 | NDP | 1.53864 | 0.039393 | UP |
| ENSSSCG00000013241 | NR1H3 | 1.128935 | 0.040572 | UP |
| ENSSSCG00000036735 | VCPKMT | 1.10441 | 0.042508 | UP |
| ENSSSCG00000017750 | EVI2B | 1.483237 | 0.045169 | UP |
| ENSSSCG00000002783 | SLC9A5 | 1.082347 | 0.049089 | UP |
| ENSSSCG00000006879 | PTBP2 | 1.597446 | 0.049104 | UP |
| ENSSSCG00000003828 | FGGY | -1.16154 | 2.49E-05 | DOWN |
| ENSSSCG00000032583 | SRSF5 | -1.15406 | 6.06E-05 | DOWN |
| ENSSSCG00000014011 | RASGEF1C | -3.61295 | 9.06E-05 | DOWN |
| ENSSSCG00000013243 | DDB2 | -1.07449 | 0.000187 | DOWN |
| ENSSSCG00000025876 | PBLD | -1.12966 | 0.000273 | DOWN |
| ENSSSCG00000023871 | NA | -1.62055 | 0.000446 | DOWN |
| ENSSSCG00000023618 | FRMD7 | -2.54471 | 0.00062 | DOWN |
| ENSSSCG00000021591 | RBM3 | -1.13362 | 0.000667 | DOWN |
| ENSSSCG00000010627 | PDCD4 | -1.86975 | 0.00079 | DOWN |
| ENSSSCG00000022345 | CHN1 | -1.55779 | 0.00127 | DOWN |
| ENSSSCG00000015175 | VWA5A | -1.01405 | 0.001368 | DOWN |
| ENSSSCG00000004601 | MNS1 | -1.87374 | 0.002039 | DOWN |
| ENSSSCG00000036790 | AKAP7 | -1.17125 | 0.002924 | DOWN |
| ENSSSCG00000025711 | MEIOB | -1.05215 | 0.003476 | DOWN |
| ENSSSCG00000016611 | CADPS2 | -1.37788 | 0.004784 | DOWN |
| ENSSSCG00000008873 | FAM198B | -1.98522 | 0.005158 | DOWN |
| ENSSSCG00000034398 | C8orf37 | -1.20155 | 0.005826 | DOWN |
| ENSSSCG00000001052 | PHACTR1 | -3.6798 | 0.006347 | DOWN |
| ENSSSCG00000004781 | NA | -2.17653 | 0.006473 | DOWN |
| ENSSSCG00000002935 | ZNF568 | -1.09093 | 0.007148 | DOWN |
| ENSSSCG00000011765 | USP13 | -1.64857 | 0.007148 | DOWN |
| ENSSSCG00000039364 | NA | -3.22754 | 0.007317 | DOWN |
| ENSSSCG00000037270 | TCTEX1D4 | -2.01669 | 0.009754 | DOWN |
| ENSSSCG00000005430 | NA | -1.13615 | 0.010008 | DOWN |
| ENSSSCG00000014672 | NA | -2.6039 | 0.010601 | DOWN |
| ENSSSCG00000028623 | RARRES1 | -2.36425 | 0.011434 | DOWN |
| ENSSSCG00000009448 | DIAPH3 | -3.6059 | 0.01268 | DOWN |
| ENSSSCG00000002849 | NA | -3.41007 | 0.013769 | DOWN |
| ENSSSCG00000038475 | NA | -1.24886 | 0.014963 | DOWN |
| ENSSSCG00000016263 | NA | -1.36955 | 0.01544 | DOWN |
| ENSSSCG00000013933 | PBX4 | -1.95519 | 0.015441 | DOWN |
| ENSSSCG00000001476 | HSD17B8 | -1.3624 | 0.015461 | DOWN |
| ENSSSCG00000028092 | GNG2 | -1.41218 | 0.016217 | DOWN |
| ENSSSCG00000016613 | AASS | -1.11944 | 0.021521 | DOWN |
| ENSSSCG00000027860 | ERAP2 | -1.4302 | 0.021601 | DOWN |
| ENSSSCG00000035374 | TRAPPC6A | -1.43973 | 0.021601 | DOWN |
| ENSSSCG00000016831 | SPEF2 | -2.29307 | 0.022191 | DOWN |
| ENSSSCG00000004332 | BACH2 | -1.53379 | 0.022567 | DOWN |
| ENSSSCG00000033993 | PLCXD3 | -2.0515 | 0.022975 | DOWN |
| ENSSSCG00000013455 | IZUMO4 | -1.71107 | 0.022975 | DOWN |
| ENSSSCG00000027466 | PCOLCE | -1.2637 | 0.023818 | DOWN |
| ENSSSCG00000013294 | LDLRAD3 | -1.75718 | 0.02388 | DOWN |
| ENSSSCG00000016690 | CREB5 | -1.51261 | 0.024746 | DOWN |
| ENSSSCG00000026146 | GALNT5 | -1.85012 | 0.026125 | DOWN |
| ENSSSCG00000001427 | C4A | -1.96483 | 0.026418 | DOWN |
| ENSSSCG00000039062 | NA | -2.81397 | 0.028329 | DOWN |
| ENSSSCG00000010214 | NA | -2.23312 | 0.028484 | DOWN |
| ENSSSCG00000031140 | NA | -1.43053 | 0.029476 | DOWN |
| ENSSSCG00000034308 | LRMDA | -1.14508 | 0.029502 | DOWN |
| ENSSSCG00000012627 | NA | -1.70341 | 0.029502 | DOWN |
| ENSSSCG00000023243 | NFIA | -1.0653 | 0.029993 | DOWN |
| ENSSSCG00000014149 | MEF2C | -1.385 | 0.030593 | DOWN |
| ENSSSCG00000015550 | RGS16 | -1.52696 | 0.032909 | DOWN |
| ENSSSCG00000001910 | ISLR | -1.41172 | 0.032909 | DOWN |
| ENSSSCG00000014221 | LVRN | -2.56511 | 0.032909 | DOWN |
| ENSSSCG00000011212 | RARB | -1.41593 | 0.032909 | DOWN |
| ENSSSCG00000000802 | NELL2 | -2.27164 | 0.03298 | DOWN |
| ENSSSCG00000037766 | NR3C2 | -1.28245 | 0.033186 | DOWN |
| ENSSSCG00000010142 | RYR2 | -2.90075 | 0.03364 | DOWN |
| ENSSSCG00000034756 | PLB1 | -4.06773 | 0.034064 | DOWN |
| ENSSSCG00000036772 | CAMTA1 | -1.84193 | 0.034853 | DOWN |
| ENSSSCG00000013043 | MACROD1 | -1.10501 | 0.034933 | DOWN |
| ENSSSCG00000034229 | MIS18A | -1.66117 | 0.035501 | DOWN |
| ENSSSCG00000004782 | BUB1B | -2.43777 | 0.035501 | DOWN |
| ENSSSCG00000004087 | CCDC170 | -1.04964 | 0.035634 | DOWN |
| ENSSSCG00000010330 | PPIF | -1.37032 | 0.036271 | DOWN |
| ENSSSCG00000001977 | STXBP6 | -1.05958 | 0.036911 | DOWN |
| ENSSSCG00000037969 | NA | -1.13041 | 0.037118 | DOWN |
| ENSSSCG00000034802 | NA | -1.10267 | 0.037958 | DOWN |
| ENSSSCG00000004202 | SAMD3 | -1.7252 | 0.038084 | DOWN |
| ENSSSCG00000011090 | NEBL | -3.70206 | 0.03877 | DOWN |
| ENSSSCG00000010816 | TGFB2 | -1.15541 | 0.03993 | DOWN |
| ENSSSCG00000006001 | ENPP2 | -2.49252 | 0.040522 | DOWN |
| ENSSSCG00000009489 | NA | -2.9013 | 0.041016 | DOWN |
| ENSSSCG00000010457 | KIF20B | -1.58442 | 0.041078 | DOWN |
| ENSSSCG00000029227 | LDB2 | -2.52186 | 0.042967 | DOWN |
| ENSSSCG00000004898 | TNFRSF11A | -1.76194 | 0.043434 | DOWN |
| ENSSSCG00000031764 | NA | -1.13152 | 0.043578 | DOWN |
| ENSSSCG00000000207 | NA | -1.24412 | 0.043578 | DOWN |
| ENSSSCG00000010222 | ZNF365 | -1.29506 | 0.044596 | DOWN |
| ENSSSCG00000008101 | FBLN7 | -1.68822 | 0.045544 | DOWN |
| ENSSSCG00000027745 | ABCG1 | -2.0454 | 0.045734 | DOWN |
| ENSSSCG00000031053 | S100A1 | -1.69538 | 0.049104 | DOWN |
| ENSSSCG00000014670 | TRIM5 | -1.23693 | 0.049104 | DOWN |
| ENSSSCG00000031407 | STKLD1 | -1.19466 | 0.049104 | DOWN |

Gene Name “NA” indicates the gene ID was not matched to a HGNC gene name.
