# Supplementary material for: A Tale of Two Loads: Modulation of IL-1 Induced Inflammatory Responses of Meniscal Cells in Two Models of Dynamic Physiologic Loading
Source: Front Bioeng Biotechnol. 2022 Mar 1;10:837619. doi: 10.3389/fbioe.2022.837619 (PMC8921261; doi:10.3389/fbioe.2022.837619)
Supplement: Supplementary file 12 [file DataSheet2.DOCX]

**Supplemental Table 3**: IL-1α stimulation compared to unstimulated samples for unloaded inner zone tissue.

| **Gene ID** | **Gene Name** | **Log2Fold Change** | **p-value** | **Up/Down Regulated** |
| --- | --- | --- | --- | --- |
| ENSSSCG00000008953 | CXCL8 | 8.161269375 | 2.98858E-17 | UP |
| ENSSSCG00000020970 | IL6 | 7.872730219 | 2.47183E-26 | UP |
| ENSSSCG00000008768 | ARAP2 | 7.532095447 | 7.20848E-05 | UP |
| ENSSSCG00000008090 | IL1A | 7.321073766 | 3.80407E-08 | UP |
| ENSSSCG00000008957 | AMCF-II | 7.304568882 | 1.55851E-12 | UP |
| ENSSSCG00000014897 | FAM181B | 7.00931335 | 2.23016E-05 | UP |
| ENSSSCG00000039341 | NA | 6.649815863 | 6.70832E-08 | UP |
| ENSSSCG00000039758 | NA | 6.232611216 | 0.000963462 | UP |
| ENSSSCG00000039761 | MYCL | 6.027238958 | 0.002247325 | UP |
| ENSSSCG00000015715 | EN1 | 5.974708231 | 0.001924558 | UP |
| ENSSSCG00000004195 | ARG1 | 5.949334195 | 1.10323E-23 | UP |
| ENSSSCG00000008959 | CXCL2 | 5.771358833 | 5.77189E-17 | UP |
| ENSSSCG00000009100 | TNIP3 | 5.751407715 | 0.001792195 | UP |
| ENSSSCG00000028460 | S1PR5 | 5.635809522 | 0.001901198 | UP |
| ENSSSCG00000032108 | SH2D1B | 5.590567283 | 0.013708005 | UP |
| ENSSSCG00000017488 | CSF3 | 5.579452667 | 0.000606104 | UP |
| ENSSSCG00000023627 | LYPD6 | 5.42453981 | 0.001959146 | UP |
| ENSSSCG00000039395 | GAS2L2 | 5.402169891 | 0.002947671 | UP |
| ENSSSCG00000029096 | HRH2 | 5.341326741 | 0.014787248 | UP |
| ENSSSCG00000013835 | CASP14 | 5.340600677 | 0.00464404 | UP |
| ENSSSCG00000001841 | RHCG | 5.280138043 | 0.006383709 | UP |
| ENSSSCG00000017991 | PIK3R5 | 5.252031822 | 1.11521E-06 | UP |
| ENSSSCG00000004819 | CERS3 | 5.238326635 | 0.033178896 | UP |
| ENSSSCG00000015299 | STEAP4 | 5.209518375 | 0.000895606 | UP |
| ENSSSCG00000008954 | NA | 5.163310522 | 1.11742E-15 | UP |
| ENSSSCG00000032149 | PLET1 | 5.077037695 | 0.003968402 | UP |
| ENSSSCG00000014987 | MMP12 | 4.98360404 | 4.61017E-17 | UP |
| ENSSSCG00000012479 | PCDH19 | 4.963189013 | 0.00715429 | UP |
| ENSSSCG00000001459 | HLA-DOB | 4.960965568 | 0.010092278 | UP |
| ENSSSCG00000003669 | MFSD2A | 4.935932084 | 9.1859E-12 | UP |
| ENSSSCG00000013614 | CNN1 | 4.825947399 | 0.019150909 | UP |
| ENSSSCG00000008888 | NPY1R | 4.777378517 | 0.037130986 | UP |
| ENSSSCG00000025345 | GUCY2D | 4.72741705 | 0.013352965 | UP |
| ENSSSCG00000008384 | C2orf74 | 4.702322331 | 0.012605838 | UP |
| ENSSSCG00000005941 | KHDRBS3 | 4.687774848 | 0.041420542 | UP |
| ENSSSCG00000024305 | MT3 | 4.586666187 | 0.022100355 | UP |
| ENSSSCG00000038727 | GDNF | 4.577939582 | 0.000251328 | UP |
| ENSSSCG00000008866 | GUCY1A3 | 4.565623193 | 0.004470568 | UP |
| ENSSSCG00000039498 | RBFOX3 | 4.54244829 | 0.01114022 | UP |
| ENSSSCG00000015579 | PTGS2 | 4.514662272 | 2.78173E-19 | UP |
| ENSSSCG00000017723 | CCL2 | 4.445529361 | 6.4508E-05 | UP |
| ENSSSCG00000005203 | IL33 | 4.445092498 | 0.005586224 | UP |
| ENSSSCG00000016254 | CCL20 | 4.365327415 | 6.16051E-18 | UP |
| ENSSSCG00000003744 | MOCOS | 4.256279975 | 2.1161E-05 | UP |
| ENSSSCG00000024022 | TRPC6 | 4.167931948 | 0.011714619 | UP |
| ENSSSCG00000004907 | CCBE1 | 4.114021283 | 5.64723E-05 | UP |
| ENSSSCG00000040961 | LIF | 4.096247295 | 4.82429E-13 | UP |
| ENSSSCG00000013940 | NLRP3 | 4.090497599 | 0.008900228 | UP |
| ENSSSCG00000000291 | GPR84 | 4.024652649 | 1.08523E-06 | UP |
| ENSSSCG00000037735 | NA | 4.006774758 | 0.003649973 | UP |
| ENSSSCG00000010054 | ADORA2A | 3.966015506 | 1.36592E-12 | UP |
| ENSSSCG00000022638 | ATP12A | 3.949733583 | 0.000513818 | UP |
| ENSSSCG00000012027 | ADAMTS5 | 3.88159519 | 4.84398E-14 | UP |
| ENSSSCG00000032857 | S100A12 | 3.847168485 | 6.53489E-05 | UP |
| ENSSSCG00000020580 | SNORA81 | 3.821927932 | 0.045255943 | UP |
| ENSSSCG00000025969 | PTPRR | 3.795683645 | 0.00675753 | UP |
| ENSSSCG00000000385 | SLC39A5 | 3.730235551 | 0.033579042 | UP |
| ENSSSCG00000028112 | CLIC6 | 3.720683303 | 0.012774105 | UP |
| ENSSSCG00000013885 | FCHO1 | 3.676264141 | 3.71695E-06 | UP |
| ENSSSCG00000026587 | BATF3 | 3.640227368 | 4.52271E-06 | UP |
| ENSSSCG00000036980 | NA | 3.639523667 | 0.044692844 | UP |
| ENSSSCG00000006588 | S100A9 | 3.626424673 | 0.000100957 | UP |
| ENSSSCG00000038185 | EREG | 3.567149134 | 8.69558E-08 | UP |
| ENSSSCG00000006590 | S100A8 | 3.561976861 | 0.001178413 | UP |
| ENSSSCG00000002828 | LPCAT2 | 3.522067174 | 0.007281637 | UP |
| ENSSSCG00000034087 | TNFSF15 | 3.462588019 | 3.3002E-10 | UP |
| ENSSSCG00000007488 | DOK5 | 3.451243529 | 0.018832861 | UP |
| ENSSSCG00000014310 | CXCL14 | 3.442100375 | 0.03946346 | UP |
| ENSSSCG00000011727 | PTX3 | 3.436872438 | 3.93426E-09 | UP |
| ENSSSCG00000001404 | TNF | 3.332746914 | 0.01916913 | UP |
| ENSSSCG00000006982 | ZDHHC2 | 3.302621228 | 3.84654E-08 | UP |
| ENSSSCG00000004302 | NA | 3.262574507 | 0.038957292 | UP |
| ENSSSCG00000040707 | RASEF | 3.229016862 | 0.000351503 | UP |
| ENSSSCG00000006391 | ATP1A2 | 3.227200047 | 0.000439494 | UP |
| ENSSSCG00000011747 | CLDN11 | 3.224642129 | 0.038635359 | UP |
| ENSSSCG00000004670 | C15orf48 | 3.221649028 | 2.58464E-06 | UP |
| ENSSSCG00000013551 | C3 | 3.197318104 | 7.28046E-08 | UP |
| ENSSSCG00000024166 | SLC2A6 | 3.19482011 | 4.19152E-06 | UP |
| ENSSSCG00000010960 | SLC28A3 | 3.178416102 | 0.040965696 | UP |
| ENSSSCG00000035541 | NA | 3.164973686 | 0.005520255 | UP |
| ENSSSCG00000016652 | LRRN3 | 3.012187043 | 0.028950295 | UP |
| ENSSSCG00000008870 | GUCY1B3 | 2.993758671 | 0.003078416 | UP |
| ENSSSCG00000026454 | NA | 2.958434573 | 2.52433E-07 | UP |
| ENSSSCG00000007465 | B4GALT5 | 2.953830403 | 2.02784E-13 | UP |
| ENSSSCG00000016519 | AKR1D1 | 2.926492203 | 2.87634E-06 | UP |
| ENSSSCG00000010850 | NA | 2.913561705 | 1.15518E-11 | UP |
| ENSSSCG00000037815 | ZC3H12A | 2.913547905 | 1.03966E-17 | UP |
| ENSSSCG00000012607 | NA | 2.889105211 | 0.035352469 | UP |
| ENSSSCG00000036746 | RASL10B | 2.888774824 | 1.71561E-07 | UP |
| ENSSSCG00000038842 | PCDH9 | 2.879190425 | 4.45637E-13 | UP |
| ENSSSCG00000024439 | PTGER4 | 2.865329876 | 0.000522814 | UP |
| ENSSSCG00000017700 | CCL3L1 | 2.857588025 | 0.001719333 | UP |
| ENSSSCG00000034364 | NA | 2.851940109 | 3.38575E-05 | UP |
| ENSSSCG00000013425 | MISP | 2.850099724 | 1.30334E-07 | UP |
| ENSSSCG00000026602 | PTGIR | 2.801972682 | 2.15128E-10 | UP |
| ENSSSCG00000009630 | EGR3 | 2.801878059 | 0.04749438 | UP |
| ENSSSCG00000016230 | EPHA4 | 2.792020322 | 1.78651E-05 | UP |
| ENSSSCG00000032870 | NA | 2.770504993 | 0.021875531 | UP |
| ENSSSCG00000014985 | MMP3 | 2.757405667 | 1.65427E-09 | UP |
| ENSSSCG00000009002 | TLR2 | 2.729412556 | 4.97279E-13 | UP |
| ENSSSCG00000038646 | NA | 2.729025563 | 4.82429E-13 | UP |
| ENSSSCG00000004125 | STX11 | 2.706639757 | 4.17816E-09 | UP |
| ENSSSCG00000035051 | ADORA2B | 2.696973581 | 2.79071E-05 | UP |
| ENSSSCG00000038562 | RND1 | 2.689996738 | 1.1567E-07 | UP |
| ENSSSCG00000015784 | ACSL1 | 2.670897793 | 4.48679E-18 | UP |
| ENSSSCG00000016997 | FGF18 | 2.669971727 | 1.63401E-07 | UP |
| ENSSSCG00000012026 | ADAMTS1 | 2.663089381 | 0.005638087 | UP |
| ENSSSCG00000033702 | SBSN | 2.659156227 | 1.93515E-05 | UP |
| ENSSSCG00000037642 | ARID3A | 2.656689464 | 1.133E-15 | UP |
| ENSSSCG00000012202 | NA | 2.655411935 | 8.52657E-10 | UP |
| ENSSSCG00000035369 | RD3 | 2.637995254 | 0.007846257 | UP |
| ENSSSCG00000023305 | NA | 2.635370406 | 1.34458E-05 | UP |
| ENSSSCG00000016573 | IRF5 | 2.62160125 | 0.026222612 | UP |
| ENSSSCG00000029675 | MMP8 | 2.602900889 | 0.002319639 | UP |
| ENSSSCG00000036758 | HSPA12A | 2.601590811 | 0.001856342 | UP |
| ENSSSCG00000015487 | TNFSF18 | 2.599203262 | 0.008252373 | UP |
| ENSSSCG00000033952 | CITED4 | 2.572366047 | 0.034744747 | UP |
| ENSSSCG00000035392 | IGFBP2 | 2.571824296 | 0.001434704 | UP |
| ENSSSCG00000034120 | CDKN2B | 2.565024143 | 0.006958338 | UP |
| ENSSSCG00000006987 | SLC7A2 | 2.560199451 | 5.54339E-06 | UP |
| ENSSSCG00000010922 | ELF3 | 2.532569709 | 0.003949348 | UP |
| ENSSSCG00000034721 | NA | 2.506395934 | 0.015099346 | UP |
| ENSSSCG00000033355 | LGI3 | 2.491880479 | 6.34493E-06 | UP |
| ENSSSCG00000016285 | ECEL1 | 2.477380347 | 0.02871291 | UP |
| ENSSSCG00000022961 | CLMP | 2.456402077 | 1.67718E-06 | UP |
| ENSSSCG00000032517 | DMXL2 | 2.454725919 | 1.40542E-09 | UP |
| ENSSSCG00000028531 | SH3BP1 | 2.45458756 | 0.003130286 | UP |
| ENSSSCG00000015595 | ATF3 | 2.437488802 | 5.21698E-10 | UP |
| ENSSSCG00000020953 | NA | 2.434763985 | 0.012674419 | UP |
| ENSSSCG00000012074 | NA | 2.432499581 | 1.08987E-06 | UP |
| ENSSSCG00000036274 | NA | 2.425018566 | 0.009726177 | UP |
| ENSSSCG00000023796 | NA | 2.421215782 | 0.000107319 | UP |
| ENSSSCG00000003451 | NA | 2.415797972 | 2.86359E-05 | UP |
| ENSSSCG00000033606 | HIST1H2AC | 2.407644456 | 0.005196301 | UP |
| ENSSSCG00000023684 | MT1A | 2.402425204 | 2.80628E-05 | UP |
| ENSSSCG00000040207 | P2RY2 | 2.393920541 | 0.000149606 | UP |
| ENSSSCG00000005688 | PTGES | 2.380943375 | 2.98214E-11 | UP |
| ENSSSCG00000010448 | FAS | 2.375737237 | 7.2347E-09 | UP |
| ENSSSCG00000036113 | NA | 2.373609364 | 0.000386472 | UP |
| ENSSSCG00000024914 | NA | 2.373578328 | 1.31117E-05 | UP |
| ENSSSCG00000010219 | ARID5B | 2.363813476 | 8.81067E-10 | UP |
| ENSSSCG00000012138 | ACE2 | 2.340466766 | 0.001070954 | UP |
| ENSSSCG00000008963 | AREG | 2.337203289 | 0.047602336 | UP |
| ENSSSCG00000028359 | BST1 | 2.329166889 | 1.88504E-05 | UP |
| ENSSSCG00000012883 | GAL | 2.326713889 | 0.017253486 | UP |
| ENSSSCG00000037645 | COTL1 | 2.318808631 | 5.19543E-06 | UP |
| ENSSSCG00000020872 | NA | 2.316524609 | 0.001085742 | UP |
| ENSSSCG00000013387 | NA | 2.303690358 | 0.000323292 | UP |
| ENSSSCG00000016497 | DENND2A | 2.301264393 | 2.90723E-05 | UP |
| ENSSSCG00000002653 | JPH3 | 2.290284221 | 0.036708056 | UP |
| ENSSSCG00000026828 | SNORA64 | 2.290174917 | 0.043442835 | UP |
| ENSSSCG00000038500 | TRIB1 | 2.281672397 | 2.43561E-17 | UP |
| ENSSSCG00000001931 | NA | 2.267047665 | 0.027425093 | UP |
| ENSSSCG00000004511 | NA | 2.257242975 | 0.017657454 | UP |
| ENSSSCG00000034114 | GPR68 | 2.25166991 | 2.96874E-08 | UP |
| ENSSSCG00000017755 | NOS2 | 2.250652337 | 0.001790913 | UP |
| ENSSSCG00000004241 | GJA1 | 2.241198988 | 8.42222E-09 | UP |
| ENSSSCG00000001752 | CHRNA3 | 2.235167022 | 0.011410328 | UP |
| ENSSSCG00000024800 | DSC3 | 2.228357306 | 0.040724345 | UP |
| ENSSSCG00000032622 | PPP3CC | 2.220473047 | 3.42086E-21 | UP |
| ENSSSCG00000030300 | MT2A | 2.190365218 | 6.70832E-08 | UP |
| ENSSSCG00000007000 | FAT1 | 2.176476245 | 0.0001098 | UP |
| ENSSSCG00000016900 | ESM1 | 2.170192979 | 0.003654336 | UP |
| ENSSSCG00000031970 | RASSF5 | 2.16896203 | 7.96366E-11 | UP |
| ENSSSCG00000040714 | RSPH6A | 2.142024731 | 0.010647605 | UP |
| ENSSSCG00000034973 | CXCL12 | 2.117046822 | 0.044464893 | UP |
| ENSSSCG00000027607 | IER3 | 2.112201194 | 2.63589E-18 | UP |
| ENSSSCG00000001952 | NFKBIA | 2.095617299 | 1.0527E-14 | UP |
| ENSSSCG00000020591 | 7SK | 2.087742953 | 0.049585631 | UP |
| ENSSSCG00000012583 | ACSL4 | 2.077610189 | 3.29348E-14 | UP |
| ENSSSCG00000037803 | MARCKS | 2.073760766 | 0.002429361 | UP |
| ENSSSCG00000015407 | GNAI1 | 2.072906981 | 0.000358601 | UP |
| ENSSSCG00000007586 | FSCN1 | 2.066891528 | 0.001021503 | UP |
| ENSSSCG00000008168 | RNF149 | 2.058089494 | 1.20729E-14 | UP |
| ENSSSCG00000027529 | BIRC3 | 2.057540633 | 4.74835E-13 | UP |
| ENSSSCG00000036911 | NA | 2.044299296 | 3.7162E-05 | UP |
| ENSSSCG00000006187 | MSC | 2.039887482 | 8.39897E-05 | UP |
| ENSSSCG00000013370 | NA | 2.029674384 | 0.004120134 | UP |
| ENSSSCG00000015390 | NA | 2.028881152 | 0.004143987 | UP |
| ENSSSCG00000006718 | ZNF697 | 2.028325706 | 4.63566E-08 | UP |
| ENSSSCG00000015037 | IL18 | 2.027584448 | 0.001175449 | UP |
| ENSSSCG00000013599 | ANGPTL4 | 2.020806707 | 1.33718E-05 | UP |
| ENSSSCG00000030681 | MYBPH | 2.010205194 | 0.01714286 | UP |
| ENSSSCG00000038073 | NOD2 | 2.007758838 | 1.28627E-06 | UP |
| ENSSSCG00000027991 | NA | 1.986521017 | 0.000366399 | UP |
| ENSSSCG00000011643 | AMOTL2 | 1.986336521 | 5.56194E-15 | UP |
| ENSSSCG00000022447 | F3 | 1.985368835 | 0.004026038 | UP |
| ENSSSCG00000040725 | IL11 | 1.942722722 | 0.035829068 | UP |
| ENSSSCG00000025770 | ST6GAL1 | 1.934604512 | 5.4649E-09 | UP |
| ENSSSCG00000010840 | RGS7 | 1.919354156 | 0.014147364 | UP |
| ENSSSCG00000038285 | KLHL36 | 1.917935967 | 2.53797E-08 | UP |
| ENSSSCG00000005211 | CD274 | 1.911520589 | 0.004504132 | UP |
| ENSSSCG00000007501 | BMP7 | 1.904230227 | 0.00751962 | UP |
| ENSSSCG00000033786 | NA | 1.899079299 | 1.08523E-06 | UP |
| ENSSSCG00000001073 | TPMT | 1.891575467 | 0.000594429 | UP |
| ENSSSCG00000032367 | CEBPD | 1.889253865 | 4.58716E-14 | UP |
| ENSSSCG00000031255 | NA | 1.883238727 | 0.012022598 | UP |
| ENSSSCG00000010312 | PLAU | 1.881651441 | 0.010925159 | UP |
| ENSSSCG00000026962 | A4GALT | 1.86697623 | 1.35407E-06 | UP |
| ENSSSCG00000015476 | CHI3L1 | 1.862949987 | 0.007971147 | UP |
| ENSSSCG00000040317 | SOD2 | 1.854405071 | 6.97398E-07 | UP |
| ENSSSCG00000017448 | KRT14 | 1.846222114 | 0.038845334 | UP |
| ENSSSCG00000002368 | LTBP2 | 1.841036447 | 0.001120608 | UP |
| ENSSSCG00000015444 | LAMB1 | 1.835230127 | 0.000150908 | UP |
| ENSSSCG00000011951 | NFKBIZ | 1.834441545 | 1.42566E-09 | UP |
| ENSSSCG00000001888 | RPP25 | 1.831506081 | 0.002894947 | UP |
| ENSSSCG00000005610 | SLC2A8 | 1.828762082 | 2.14294E-08 | UP |
| ENSSSCG00000009216 | SPP1 | 1.827313063 | 8.80787E-05 | UP |
| ENSSSCG00000002294 | ARG2 | 1.824241829 | 0.02270395 | UP |
| ENSSSCG00000003920 | HPDL | 1.822901781 | 0.039007514 | UP |
| ENSSSCG00000012006 | NA | 1.822863713 | 9.91519E-07 | UP |
| ENSSSCG00000022784 | PLEKHO1 | 1.821465919 | 0.010739047 | UP |
| ENSSSCG00000013766 | IL27RA | 1.807272612 | 2.38279E-07 | UP |
| ENSSSCG00000007140 | SMOX | 1.80686604 | 2.6496E-09 | UP |
| ENSSSCG00000002754 | NQO1 | 1.805371413 | 0.000181229 | UP |
| ENSSSCG00000035077 | INHBA | 1.805332753 | 5.55338E-05 | UP |
| ENSSSCG00000039472 | SLC30A1 | 1.796574772 | 1.39398E-12 | UP |
| ENSSSCG00000015085 | IL10RA | 1.789055189 | 0.006455741 | UP |
| ENSSSCG00000004829 | CACHD1 | 1.783563784 | 0.018634506 | UP |
| ENSSSCG00000022446 | SEL1L3 | 1.777875419 | 0.000219589 | UP |
| ENSSSCG00000022401 | AGTRAP | 1.768630373 | 1.39398E-12 | UP |
| ENSSSCG00000023192 | NA | 1.768420663 | 0.008869327 | UP |
| ENSSSCG00000010330 | PPIF | 1.763357717 | 0.000221603 | UP |
| ENSSSCG00000023520 | PCSK5 | 1.756847654 | 1.81202E-05 | UP |
| ENSSSCG00000015303 | CFAP69 | 1.754810099 | 0.003348762 | UP |
| ENSSSCG00000010473 | EXOC6 | 1.748116976 | 0.034847518 | UP |
| ENSSSCG00000013292 | PRR5L | 1.745610311 | 6.16165E-11 | UP |
| ENSSSCG00000021386 | PTGR1 | 1.745360167 | 8.22499E-05 | UP |
| ENSSSCG00000012967 | FOSL1 | 1.732781096 | 1.03207E-06 | UP |
| ENSSSCG00000000456 | SLC16A7 | 1.732585083 | 2.83786E-05 | UP |
| ENSSSCG00000011218 | SLC4A7 | 1.728876209 | 2.64323E-10 | UP |
| ENSSSCG00000034265 | NA | 1.727978999 | 1.31974E-15 | UP |
| ENSSSCG00000012743 | MTMR1 | 1.727449374 | 4.5573E-21 | UP |
| ENSSSCG00000015136 | UBASH3B | 1.724143362 | 0.003888905 | UP |
| ENSSSCG00000003707 | NPC1 | 1.717168316 | 6.78701E-08 | UP |
| ENSSSCG00000005267 | ANXA1 | 1.716575607 | 1.38618E-05 | UP |
| ENSSSCG00000004149 | NHSL1 | 1.71051055 | 1.63912E-06 | UP |
| ENSSSCG00000017393 | CNTNAP1 | 1.709724022 | 0.033178896 | UP |
| ENSSSCG00000002135 | PNP | 1.704654275 | 8.74371E-06 | UP |
| ENSSSCG00000029813 | TSPAN5 | 1.702004148 | 0.017878027 | UP |
| ENSSSCG00000028322 | BTG2 | 1.697799386 | 5.73012E-08 | UP |
| ENSSSCG00000038671 | TNFRSF6B | 1.693566282 | 0.018680553 | UP |
| ENSSSCG00000007772 | STX1A | 1.690795614 | 0.022857048 | UP |
| ENSSSCG00000008443 | EPAS1 | 1.689668122 | 3.85529E-08 | UP |
| ENSSSCG00000015770 | VEGFC | 1.683273525 | 0.000485601 | UP |
| ENSSSCG00000000475 | IRAK3 | 1.678218727 | 5.58718E-07 | UP |
| ENSSSCG00000008092 | NT5DC4 | 1.663593423 | 0.005054205 | UP |
| ENSSSCG00000032434 | PLAUR | 1.654323609 | 1.6752E-06 | UP |
| ENSSSCG00000020705 | MAP3K8 | 1.652003509 | 1.05596E-07 | UP |
| ENSSSCG00000000843 | TXNRD1 | 1.651868832 | 5.95904E-06 | UP |
| ENSSSCG00000003079 | NA | 1.651828704 | 3.47375E-13 | UP |
| ENSSSCG00000040445 | RND3 | 1.651179487 | 0.000288182 | UP |
| ENSSSCG00000016614 | PTPRZ1 | 1.651154415 | 0.048048318 | UP |
| ENSSSCG00000011147 | NA | 1.640958996 | 0.03821442 | UP |
| ENSSSCG00000023716 | TNFAIP6 | 1.629453656 | 0.000399945 | UP |
| ENSSSCG00000009664 | PTK2B | 1.627121214 | 0.019691476 | UP |
| ENSSSCG00000021597 | PHLDA2 | 1.623610493 | 1.58695E-11 | UP |
| ENSSSCG00000015644 | IKBKE | 1.622135094 | 0.001151289 | UP |
| ENSSSCG00000001518 | ITPR3 | 1.618107017 | 0.011364631 | UP |
| ENSSSCG00000013655 | ICAM1 | 1.608060677 | 0.000884218 | UP |
| ENSSSCG00000012843 | PIDD1 | 1.594579108 | 1.35025E-06 | UP |
| ENSSSCG00000032433 | PTCHD1 | 1.587278619 | 0.015654286 | UP |
| ENSSSCG00000035601 | HAS3 | 1.586727616 | 0.002744324 | UP |
| ENSSSCG00000011828 | FAM43A | 1.584753274 | 8.98773E-06 | UP |
| ENSSSCG00000025021 | NA | 1.58433515 | 0.003033335 | UP |
| ENSSSCG00000017178 | SPHK1 | 1.58098445 | 0.00031538 | UP |
| ENSSSCG00000009468 | KCTD12 | 1.573378702 | 0.0002386 | UP |
| ENSSSCG00000031122 | HIST1H2BB | 1.57125057 | 0.037014226 | UP |
| ENSSSCG00000004154 | TNFAIP3 | 1.5622037 | 1.55847E-05 | UP |
| ENSSSCG00000014336 | EGR1 | 1.559162111 | 0.001686608 | UP |
| ENSSSCG00000011750 | PLD1 | 1.550932586 | 7.5713E-06 | UP |
| ENSSSCG00000009827 | HVCN1 | 1.547023944 | 0.00635078 | UP |
| ENSSSCG00000027130 | TNFRSF12A | 1.543778972 | 1.83718E-08 | UP |
| ENSSSCG00000027477 | TBC1D2B | 1.535739756 | 0.000514391 | UP |
| ENSSSCG00000005269 | TRPM6 | 1.531619294 | 0.040550854 | UP |
| ENSSSCG00000004156 | IFNGR1 | 1.531031258 | 1.89068E-08 | UP |
| ENSSSCG00000028536 | LHFPL2 | 1.529924065 | 4.17384E-09 | UP |
| ENSSSCG00000037670 | TMEM164 | 1.527883838 | 1.40542E-09 | UP |
| ENSSSCG00000002311 | SUSD6 | 1.52673646 | 4.22081E-05 | UP |
| ENSSSCG00000030278 | MLLT11 | 1.524786302 | 1.6895E-10 | UP |
| ENSSSCG00000013735 | JUNB | 1.521664739 | 1.66554E-13 | UP |
| ENSSSCG00000026547 | SLC45A3 | 1.520827178 | 0.000492626 | UP |
| ENSSSCG00000002829 | MMP2 | 1.517748073 | 0.045021059 | UP |
| ENSSSCG00000028964 | PIM2 | 1.517494082 | 1.65305E-06 | UP |
| ENSSSCG00000005486 | KIF12 | 1.516922565 | 0.013998324 | UP |
| ENSSSCG00000017835 | CLUH | 1.513462545 | 2.64323E-10 | UP |
| ENSSSCG00000008535 | CLIP4 | 1.511056212 | 0.008578777 | UP |
| ENSSSCG00000016570 | TSPAN33 | 1.508364337 | 0.006427923 | UP |
| ENSSSCG00000030484 | AHR | 1.506099481 | 0.000467608 | UP |
| ENSSSCG00000027992 | MST1R | 1.504044483 | 0.000417561 | UP |
| ENSSSCG00000009434 | RGCC | 1.500793767 | 1.05704E-05 | UP |
| ENSSSCG00000029304 | STEAP3 | 1.495963341 | 0.000361339 | UP |
| ENSSSCG00000014369 | CD14 | 1.495129731 | 1.65256E-07 | UP |
| ENSSSCG00000032360 | PANX1 | 1.493953588 | 0.002353707 | UP |
| ENSSSCG00000015411 | PTPN12 | 1.489565352 | 2.31054E-11 | UP |
| ENSSSCG00000031118 | PREX1 | 1.48672445 | 1.9578E-07 | UP |
| ENSSSCG00000003805 | PDE4B | 1.484404273 | 6.31508E-09 | UP |
| ENSSSCG00000038594 | SDC4 | 1.483851463 | 8.9726E-07 | UP |
| ENSSSCG00000034758 | PDXK | 1.482907532 | 3.03515E-05 | UP |
| ENSSSCG00000000975 | PANX2 | 1.481619539 | 0.045255943 | UP |
| ENSSSCG00000032242 | NA | 1.481002016 | 8.74371E-06 | UP |
| ENSSSCG00000023972 | DRAM1 | 1.480518093 | 1.08268E-05 | UP |
| ENSSSCG00000032709 | ARL4A | 1.480326273 | 0.019094081 | UP |
| ENSSSCG00000001958 | BAZ1A | 1.478553906 | 0.000190727 | UP |
| ENSSSCG00000001782 | ABHD17C | 1.475401919 | 9.61203E-05 | UP |
| ENSSSCG00000013418 | CFD | 1.474192005 | 0.008948814 | UP |
| ENSSSCG00000010009 | GAL3ST1 | 1.466405432 | 0.040983067 | UP |
| ENSSSCG00000023118 | BZW2 | 1.464495379 | 1.38624E-12 | UP |
| ENSSSCG00000026083 | DNAJC24 | 1.456544094 | 2.11967E-07 | UP |
| ENSSSCG00000024793 | PORCN | 1.446890868 | 1.86767E-05 | UP |
| ENSSSCG00000004192 | CTGF | 1.445923064 | 0.000840111 | UP |
| ENSSSCG00000006277 | SPIDR | 1.441952466 | 4.88768E-08 | UP |
| ENSSSCG00000033037 | NA | 1.441514511 | 1.87807E-06 | UP |
| ENSSSCG00000010006 | NA | 1.436268043 | 0.000218132 | UP |
| ENSSSCG00000032843 | AHRR | 1.426441232 | 0.018306274 | UP |
| ENSSSCG00000000869 | UTP20 | 1.421824747 | 0.000566655 | UP |
| ENSSSCG00000014540 | NA | 1.420488288 | 1.91159E-07 | UP |
| ENSSSCG00000008348 | PLEK | 1.4183327 | 0.009170029 | UP |
| ENSSSCG00000033662 | NA | 1.40517238 | 0.001056859 | UP |
| ENSSSCG00000003914 | NA | 1.405168908 | 0.00016525 | UP |
| ENSSSCG00000015140 | HSPA8 | 1.397393624 | 8.48803E-08 | UP |
| ENSSSCG00000010568 | NPM3 | 1.392424577 | 1.88887E-05 | UP |
| ENSSSCG00000008510 | LTBP1 | 1.391090198 | 3.74644E-06 | UP |
| ENSSSCG00000008374 | B3GNT2 | 1.390256979 | 9.91519E-07 | UP |
| ENSSSCG00000030655 | MAMDC2 | 1.383226532 | 0.002271023 | UP |
| ENSSSCG00000038929 | CEMIP | 1.381696104 | 0.034059459 | UP |
| ENSSSCG00000021815 | NA | 1.381091765 | 0.024137214 | UP |
| ENSSSCG00000011767 | TTC14 | 1.378445052 | 1.31544E-06 | UP |
| ENSSSCG00000038879 | RELB | 1.376230265 | 1.6358E-09 | UP |
| ENSSSCG00000039760 | PRR16 | 1.375372581 | 0.000144586 | UP |
| ENSSSCG00000016233 | SERPINE2 | 1.369932447 | 0.00474003 | UP |
| ENSSSCG00000008115 | NA | 1.363063227 | 0.02600735 | UP |
| ENSSSCG00000040815 | DUSP5 | 1.360138099 | 0.04551471 | UP |
| ENSSSCG00000003169 | PIH1D1 | 1.351877317 | 0.000407008 | UP |
| ENSSSCG00000012084 | PRDM15 | 1.351601907 | 0.008422183 | UP |
| ENSSSCG00000018023 | COX10 | 1.350468577 | 0.000118975 | UP |
| ENSSSCG00000039639 | NA | 1.347480933 | 0.024490849 | UP |
| ENSSSCG00000036675 | NA | 1.342877428 | 0.039973009 | UP |
| ENSSSCG00000039419 | SLCO4A1 | 1.338542741 | 0.003654336 | UP |
| ENSSSCG00000030415 | DPP3 | 1.338115586 | 4.34867E-07 | UP |
| ENSSSCG00000037358 | NA | 1.331795546 | 0.009286429 | UP |
| ENSSSCG00000034339 | NA | 1.331473829 | 7.00022E-05 | UP |
| ENSSSCG00000000951 | NA | 1.331416272 | 3.72437E-05 | UP |
| ENSSSCG00000000521 | PHLDA1 | 1.330930024 | 0.000374685 | UP |
| ENSSSCG00000036956 | NA | 1.330260225 | 3.41003E-10 | UP |
| ENSSSCG00000010340 | FAM213A | 1.32964473 | 0.002744324 | UP |
| ENSSSCG00000003561 | ZDHHC18 | 1.325445503 | 3.32095E-09 | UP |
| ENSSSCG00000016322 | ACKR3 | 1.324701433 | 0.000645671 | UP |
| ENSSSCG00000005524 | DAB2IP | 1.323792438 | 0.011117577 | UP |
| ENSSSCG00000038535 | ARSB | 1.323659613 | 7.13935E-09 | UP |
| ENSSSCG00000015375 | ITGB8 | 1.314729345 | 0.000650478 | UP |
| ENSSSCG00000029507 | RASGEF1B | 1.307929314 | 0.044432059 | UP |
| ENSSSCG00000015302 | STEAP2 | 1.307869333 | 0.044371878 | UP |
| ENSSSCG00000040779 | NA | 1.306727068 | 0.000177031 | UP |
| ENSSSCG00000007874 | NA | 1.304231372 | 0.000783465 | UP |
| ENSSSCG00000013073 | FADS3 | 1.300766735 | 1.04032E-06 | UP |
| ENSSSCG00000036257 | NA | 1.297245939 | 2.43084E-06 | UP |
| ENSSSCG00000015808 | ADAM9 | 1.297214938 | 0.000532332 | UP |
| ENSSSCG00000006290 | SLC19A2 | 1.294431784 | 3.0911E-06 | UP |
| ENSSSCG00000013297 | CD44 | 1.2893886 | 0.003332779 | UP |
| ENSSSCG00000023709 | PTPRJ | 1.287977893 | 1.48851E-05 | UP |
| ENSSSCG00000029002 | PNKD | 1.284492494 | 3.54926E-07 | UP |
| ENSSSCG00000033628 | NA | 1.284393469 | 0.026819397 | UP |
| ENSSSCG00000015545 | GLUL | 1.283070034 | 0.01138995 | UP |
| ENSSSCG00000011518 | SHQ1 | 1.281358446 | 0.000214416 | UP |
| ENSSSCG00000034207 | CEBPB | 1.27929636 | 1.20457E-05 | UP |
| ENSSSCG00000028883 | SETD4 | 1.278463183 | 0.001119222 | UP |
| ENSSSCG00000015293 | NA | 1.277663306 | 0.000152878 | UP |
| ENSSSCG00000030831 | DENND3 | 1.270722454 | 0.018577388 | UP |
| ENSSSCG00000011519 | GXYLT2 | 1.267378096 | 0.000484213 | UP |
| ENSSSCG00000004342 | NDUFAF4 | 1.266370736 | 5.0683E-05 | UP |
| ENSSSCG00000005636 | SLC25A25 | 1.262900073 | 6.34493E-06 | UP |
| ENSSSCG00000011825 | ATP13A3 | 1.258101805 | 3.36802E-10 | UP |
| ENSSSCG00000005503 | TLR4 | 1.256008216 | 0.000836088 | UP |
| ENSSSCG00000039548 | PTGFR | 1.253013735 | 0.017328239 | UP |
| ENSSSCG00000017159 | TBC1D16 | 1.25187905 | 0.021998602 | UP |
| ENSSSCG00000003402 | PGD | 1.251354975 | 1.33152E-07 | UP |
| ENSSSCG00000029230 | ECM1 | 1.247639641 | 0.003434866 | UP |
| ENSSSCG00000011672 | RASA2 | 1.246416052 | 0.000622591 | UP |
| ENSSSCG00000023026 | CLCN5 | 1.245462446 | 0.000417641 | UP |
| ENSSSCG00000033843 | ETFRF1 | 1.244672514 | 0.009333641 | UP |
| ENSSSCG00000012847 | TALDO1 | 1.238776348 | 4.2809E-06 | UP |
| ENSSSCG00000007817 | IL4R | 1.235716703 | 0.020503546 | UP |
| ENSSSCG00000018034 | NA | 1.231270756 | 8.49763E-06 | UP |
| ENSSSCG00000035394 | NA | 1.229725715 | 0.008064184 | UP |
| ENSSSCG00000035101 | KLF5 | 1.227904639 | 1.79349E-06 | UP |
| ENSSSCG00000011022 | SVIL | 1.226343568 | 0.00201104 | UP |
| ENSSSCG00000010580 | NFKB2 | 1.222124466 | 7.69043E-11 | UP |
| ENSSSCG00000003400 | NMNAT1 | 1.221028702 | 0.000178582 | UP |
| ENSSSCG00000006219 | MTFR1 | 1.219060425 | 1.00055E-10 | UP |
| ENSSSCG00000008722 | NA | 1.206616844 | 0.019014493 | UP |
| ENSSSCG00000039575 | PALM | 1.205407088 | 3.04747E-06 | UP |
| ENSSSCG00000000293 | ITGA5 | 1.203994312 | 7.23541E-05 | UP |
| ENSSSCG00000029296 | FOXC2 | 1.203346779 | 0.000390085 | UP |
| ENSSSCG00000034378 | IFNGR2 | 1.20174995 | 3.00733E-08 | UP |
| ENSSSCG00000009396 | PHF11 | 1.201747125 | 1.3921E-09 | UP |
| ENSSSCG00000032741 | TBC1D9 | 1.19765713 | 0.000136085 | UP |
| ENSSSCG00000015616 | HSD11B1 | 1.196572393 | 0.047150799 | UP |
| ENSSSCG00000013753 | IER2 | 1.191836961 | 1.4199E-05 | UP |
| ENSSSCG00000025286 | MCTP1 | 1.186476855 | 6.10498E-05 | UP |
| ENSSSCG00000012769 | ATP2B3 | 1.186393525 | 0.034633804 | UP |
| ENSSSCG00000023078 | WDR4 | 1.18484695 | 1.61091E-08 | UP |
| ENSSSCG00000016851 | OSMR | 1.184156028 | 0.004365603 | UP |
| ENSSSCG00000037591 | AMMECR1 | 1.179959638 | 8.88155E-07 | UP |
| ENSSSCG00000020803 | BRPF3 | 1.177690932 | 0.000351358 | UP |
| ENSSSCG00000004172 | SLC2A12 | 1.176741631 | 0.039631928 | UP |
| ENSSSCG00000015549 | RNASEL | 1.175501367 | 0.00086564 | UP |
| ENSSSCG00000012915 | CLCF1 | 1.170500559 | 7.03086E-05 | UP |
| ENSSSCG00000028080 | ELOVL4 | 1.169983931 | 0.026325523 | UP |
| ENSSSCG00000022742 | PRDX6 | 1.169607833 | 1.11521E-06 | UP |
| ENSSSCG00000022194 | NA | 1.169136441 | 0.006096687 | UP |
| ENSSSCG00000013322 | NA | 1.166709534 | 0.004120463 | UP |
| ENSSSCG00000003464 | DNAJC16 | 1.166273861 | 0.000698651 | UP |
| ENSSSCG00000031789 | ACSL5 | 1.164622395 | 0.007549084 | UP |
| ENSSSCG00000024219 | TIGAR | 1.164210999 | 4.76344E-05 | UP |
| ENSSSCG00000009620 | BMP1 | 1.16323073 | 0.000125369 | UP |
| ENSSSCG00000027722 | BMP2K | 1.162223398 | 0.000670536 | UP |
| ENSSSCG00000026455 | TMEM54 | 1.162197146 | 2.53583E-05 | UP |
| ENSSSCG00000039314 | MCL1 | 1.15994558 | 3.87898E-15 | UP |
| ENSSSCG00000001565 | CDKN1A | 1.15962869 | 0.004374678 | UP |
| ENSSSCG00000023957 | CFAP126 | 1.159492821 | 0.032504714 | UP |
| ENSSSCG00000003080 | NA | 1.156843541 | 0.044417553 | UP |
| ENSSSCG00000040181 | ELL | 1.152819171 | 1.44656E-07 | UP |
| ENSSSCG00000036308 | PGS1 | 1.15238728 | 4.40772E-07 | UP |
| ENSSSCG00000036826 | NA | 1.152331431 | 0.008269578 | UP |
| ENSSSCG00000036466 | METTL21A | 1.14938275 | 2.93429E-09 | UP |
| ENSSSCG00000025598 | COBLL1 | 1.146909543 | 0.004633573 | UP |
| ENSSSCG00000026063 | DPP9 | 1.144881945 | 4.87932E-05 | UP |
| ENSSSCG00000032164 | PEA15 | 1.143919715 | 2.60441E-11 | UP |
| ENSSSCG00000003590 | PTPRU | 1.143459508 | 0.000246253 | UP |
| ENSSSCG00000024771 | TICAM1 | 1.142873812 | 1.78777E-09 | UP |
| ENSSSCG00000026904 | NFKBIB | 1.142217867 | 4.19891E-13 | UP |
| ENSSSCG00000004572 | NA | 1.14106832 | 0.034537346 | UP |
| ENSSSCG00000010540 | ENTPD7 | 1.140545234 | 1.25421E-06 | UP |
| ENSSSCG00000013475 | NCLN | 1.139366252 | 2.63226E-08 | UP |
| ENSSSCG00000000736 | TEAD4 | 1.136333471 | 0.000117658 | UP |
| ENSSSCG00000013298 | PDHX | 1.136146587 | 2.2567E-16 | UP |
| ENSSSCG00000023351 | PLA2G4A | 1.134387431 | 5.01423E-05 | UP |
| ENSSSCG00000008412 | PSME4 | 1.133698277 | 1.56593E-05 | UP |
| ENSSSCG00000009633 | NA | 1.132510824 | 0.000407933 | UP |
| ENSSSCG00000016140 | FZD5 | 1.132458053 | 0.007740364 | UP |
| ENSSSCG00000021654 | ZNF641 | 1.132289465 | 0.007509354 | UP |
| ENSSSCG00000003729 | RNF125 | 1.129948246 | 0.001811633 | UP |
| ENSSSCG00000012880 | CPT1A | 1.12688987 | 0.003468916 | UP |
| ENSSSCG00000038717 | FARS2 | 1.122733745 | 5.45469E-06 | UP |
| ENSSSCG00000040439 | TMED5 | 1.118757365 | 1.92802E-06 | UP |
| ENSSSCG00000040778 | NA | 1.114527107 | 4.10638E-05 | UP |
| ENSSSCG00000023861 | PFAS | 1.113700286 | 1.77493E-05 | UP |
| ENSSSCG00000016646 | IFRD1 | 1.113192347 | 0.003647404 | UP |
| ENSSSCG00000004441 | DSE | 1.112553011 | 7.95342E-06 | UP |
| ENSSSCG00000028249 | SKA3 | 1.112138996 | 5.83118E-05 | UP |
| ENSSSCG00000024752 | ALDH4A1 | 1.110793615 | 0.001087626 | UP |
| ENSSSCG00000021067 | BLVRB | 1.110236938 | 0.001743496 | UP |
| ENSSSCG00000014997 | NA | 1.109302634 | 0.010615323 | UP |
| ENSSSCG00000004888 | SERPINB8 | 1.108273346 | 0.000168027 | UP |
| ENSSSCG00000033860 | ZMAT3 | 1.107718725 | 0.001919077 | UP |
| ENSSSCG00000004569 | LACTB | 1.104488137 | 0.000227257 | UP |
| ENSSSCG00000027723 | NA | 1.104296909 | 4.69108E-09 | UP |
| ENSSSCG00000034379 | MAP2K3 | 1.101054451 | 8.07256E-12 | UP |
| ENSSSCG00000030388 | UPP1 | 1.100407398 | 0.019921306 | UP |
| ENSSSCG00000028202 | RANGAP1 | 1.09978869 | 6.7387E-09 | UP |
| ENSSSCG00000005102 | AEN | 1.098375232 | 7.3497E-05 | UP |
| ENSSSCG00000039731 | NA | 1.098297277 | 0.000417561 | UP |
| ENSSSCG00000003663 | HPCAL1 | 1.096524869 | 0.001696424 | UP |
| ENSSSCG00000022945 | UCHL1 | 1.096161306 | 0.016979378 | UP |
| ENSSSCG00000024696 | NA | 1.095859906 | 0.00027044 | UP |
| ENSSSCG00000009868 | RBM19 | 1.095035218 | 0.000765607 | UP |
| ENSSSCG00000026752 | BYSL | 1.094972395 | 2.02784E-13 | UP |
| ENSSSCG00000023775 | TFB2M | 1.093604028 | 2.43339E-07 | UP |
| ENSSSCG00000027230 | SLC35G1 | 1.09342268 | 0.011853599 | UP |
| ENSSSCG00000014258 | NA | 1.088265029 | 0.001013067 | UP |
| ENSSSCG00000006481 | GPATCH4 | 1.086984225 | 1.40887E-08 | UP |
| ENSSSCG00000009335 | B3GLCT | 1.086692332 | 5.27451E-06 | UP |
| ENSSSCG00000014800 | RNF121 | 1.085245992 | 1.49469E-07 | UP |
| ENSSSCG00000000519 | GLIPR1 | 1.084891318 | 0.044399322 | UP |
| ENSSSCG00000009786 | HIP1R | 1.082278517 | 0.001156186 | UP |
| ENSSSCG00000025067 | ECE2 | 1.079408658 | 0.000457358 | UP |
| ENSSSCG00000025618 | TAP1 | 1.07837796 | 0.000847091 | UP |
| ENSSSCG00000007808 | NFATC2IP | 1.077435391 | 1.01132E-07 | UP |
| ENSSSCG00000037606 | PSMB3 | 1.077005332 | 5.67823E-11 | UP |
| ENSSSCG00000012474 | DIAPH2 | 1.072339571 | 1.4199E-05 | UP |
| ENSSSCG00000006340 | UAP1 | 1.06526117 | 5.72331E-06 | UP |
| ENSSSCG00000008491 | QPCT | 1.064810342 | 0.005468685 | UP |
| ENSSSCG00000004374 | QRSL1 | 1.064735943 | 0.00127265 | UP |
| ENSSSCG00000037209 | TLNRD1 | 1.060137949 | 1.60129E-05 | UP |
| ENSSSCG00000014034 | N4BP3 | 1.059330679 | 0.025188171 | UP |
| ENSSSCG00000010241 | TET1 | 1.05882887 | 0.00706743 | UP |
| ENSSSCG00000037900 | CCND1 | 1.057654433 | 0.007766952 | UP |
| ENSSSCG00000015823 | NA | 1.057114959 | 0.022811031 | UP |
| ENSSSCG00000008383 | NA | 1.054986862 | 0.007812183 | UP |
| ENSSSCG00000030067 | GINM1 | 1.054813607 | 1.83718E-08 | UP |
| ENSSSCG00000036742 | KLF15 | 1.054174283 | 0.008083559 | UP |
| ENSSSCG00000015322 | TFPI2 | 1.053589254 | 0.011602739 | UP |
| ENSSSCG00000001532 | UHRF1BP1 | 1.050131698 | 4.39319E-05 | UP |
| ENSSSCG00000030289 | PTPN11 | 1.048491789 | 7.83375E-12 | UP |
| ENSSSCG00000012890 | TCIRG1 | 1.043060984 | 2.93378E-08 | UP |
| ENSSSCG00000028593 | TEPSIN | 1.042016981 | 1.87402E-06 | UP |
| ENSSSCG00000000765 | IL17RA | 1.040863351 | 1.61102E-05 | UP |
| ENSSSCG00000003458 | EFHD2 | 1.037140891 | 0.001531076 | UP |
| ENSSSCG00000036735 | VCPKMT | 1.036587515 | 0.015298798 | UP |
| ENSSSCG00000000675 | C1R | 1.030385053 | 0.024037375 | UP |
| ENSSSCG00000035454 | B4GALT1 | 1.030243518 | 0.008948814 | UP |
| ENSSSCG00000006865 | RTCA | 1.026885894 | 1.16941E-06 | UP |
| ENSSSCG00000035634 | NA | 1.026748691 | 3.89534E-06 | UP |
| ENSSSCG00000009617 | REEP4 | 1.025190973 | 5.19543E-06 | UP |
| ENSSSCG00000039368 | SLC35D2 | 1.020343398 | 0.00028015 | UP |
| ENSSSCG00000022351 | GSTO1 | 1.018614173 | 0.00030008 | UP |
| ENSSSCG00000017624 | CUEDC1 | 1.018124551 | 0.00914912 | UP |
| ENSSSCG00000022492 | AMPD3 | 1.016958581 | 0.001734817 | UP |
| ENSSSCG00000026922 | DRAM2 | 1.016950182 | 0.018255902 | UP |
| ENSSSCG00000015872 | GPD2 | 1.016491493 | 0.001285386 | UP |
| ENSSSCG00000008318 | VAX2 | 1.016347209 | 0.002521875 | UP |
| ENSSSCG00000003983 | SMAP2 | 1.014914321 | 0.000261448 | UP |
| ENSSSCG00000011470 | ABHD6 | 1.013094167 | 0.000223595 | UP |
| ENSSSCG00000013366 | LDHA | 1.012631067 | 0.002532778 | UP |
| ENSSSCG00000028182 | CDK17 | 1.012273724 | 5.75525E-05 | UP |
| ENSSSCG00000029359 | PHLDA3 | 1.010945699 | 0.006096687 | UP |
| ENSSSCG00000004050 | WTAP | 1.008585762 | 5.18558E-07 | UP |
| ENSSSCG00000013576 | STXBP2 | 1.008376833 | 0.041940732 | UP |
| ENSSSCG00000005596 | ARPC5L | 1.008267586 | 8.94562E-11 | UP |
| ENSSSCG00000037277 | RFK | 1.007790463 | 0.000104602 | UP |
| ENSSSCG00000006354 | TOMM40L | 1.007473113 | 4.55747E-06 | UP |
| ENSSSCG00000014920 | FZD4 | 1.0064512 | 0.034847518 | UP |
| ENSSSCG00000003154 | GYS1 | 1.005787648 | 1.16856E-05 | UP |
| ENSSSCG00000002959 | FAM98C | 1.004453165 | 0.011602739 | UP |
| ENSSSCG00000008043 | TRAF7 | 1.003005326 | 8.47822E-12 | UP |
| ENSSSCG00000017769 | RAB34 | -1.00000938 | 7.39384E-08 | DOWN |
| ENSSSCG00000005322 | NPR2 | -1.000580577 | 0.000422781 | DOWN |
| ENSSSCG00000011683 | PCOLCE2 | -1.000670745 | 0.011581223 | DOWN |
| ENSSSCG00000001061 | JARID2 | -1.001864517 | 0.019357294 | DOWN |
| ENSSSCG00000038507 | RPS29 | -1.002127072 | 0.009039034 | DOWN |
| ENSSSCG00000000681 | P3H3 | -1.002342603 | 0.000165302 | DOWN |
| ENSSSCG00000008835 | RASL11B | -1.003018529 | 0.007429109 | DOWN |
| ENSSSCG00000032964 | STK11IP | -1.004639611 | 0.000783465 | DOWN |
| ENSSSCG00000004054 | NA | -1.005384958 | 0.008353232 | DOWN |
| ENSSSCG00000003042 | RPS19 | -1.006661787 | 0.00172764 | DOWN |
| ENSSSCG00000007864 | GPRC5B | -1.007397479 | 0.001973604 | DOWN |
| ENSSSCG00000000061 | PMM1 | -1.00814907 | 0.000348387 | DOWN |
| ENSSSCG00000031764 | NA | -1.009362423 | 0.013452581 | DOWN |
| ENSSSCG00000035355 | F2R | -1.011419357 | 0.010677481 | DOWN |
| ENSSSCG00000011404 | HYAL1 | -1.012104435 | 0.009079315 | DOWN |
| ENSSSCG00000015866 | FMNL2 | -1.012120954 | 0.016451216 | DOWN |
| ENSSSCG00000009125 | ANK2 | -1.012735368 | 0.01956008 | DOWN |
| ENSSSCG00000040332 | LBH | -1.012754742 | 0.0459159 | DOWN |
| ENSSSCG00000029771 | NA | -1.013482944 | 0.008549268 | DOWN |
| ENSSSCG00000013380 | NA | -1.014723929 | 0.00955422 | DOWN |
| ENSSSCG00000008838 | LNX1 | -1.015750019 | 8.88696E-08 | DOWN |
| ENSSSCG00000024791 | NA | -1.016192978 | 0.020774212 | DOWN |
| ENSSSCG00000009844 | HSPB8 | -1.016686557 | 0.00543511 | DOWN |
| ENSSSCG00000033452 | NA | -1.017968422 | 0.01058741 | DOWN |
| ENSSSCG00000037808 | NA | -1.018305504 | 0.019712603 | DOWN |
| ENSSSCG00000007477 | NFATC2 | -1.018335126 | 0.020267734 | DOWN |
| ENSSSCG00000024163 | NA | -1.019314153 | 2.07489E-06 | DOWN |
| ENSSSCG00000000263 | TNS2 | -1.019540877 | 2.69947E-05 | DOWN |
| ENSSSCG00000005169 | NA | -1.020579389 | 1.17018E-05 | DOWN |
| ENSSSCG00000017566 | ACSF2 | -1.021084974 | 3.4571E-08 | DOWN |
| ENSSSCG00000014569 | NA | -1.022520271 | 2.53747E-05 | DOWN |
| ENSSSCG00000035768 | RPS15A | -1.023358433 | 0.000152604 | DOWN |
| ENSSSCG00000011385 | APEH | -1.024033121 | 2.70611E-09 | DOWN |
| ENSSSCG00000009666 | EPHX2 | -1.024352246 | 0.002495242 | DOWN |
| ENSSSCG00000000848 | GLT8D2 | -1.024376609 | 2.74722E-09 | DOWN |
| ENSSSCG00000032687 | CYP4V2 | -1.025137193 | 0.000670536 | DOWN |
| ENSSSCG00000008899 | ARL9 | -1.025575437 | 0.008605872 | DOWN |
| ENSSSCG00000008081 | BARX1 | -1.025748975 | 2.82546E-08 | DOWN |
| ENSSSCG00000015232 | ST3GAL4 | -1.026073971 | 5.69858E-05 | DOWN |
| ENSSSCG00000005673 | IER5L | -1.026159467 | 1.86958E-05 | DOWN |
| ENSSSCG00000006894 | FNBP1L | -1.026486412 | 0.025679511 | DOWN |
| ENSSSCG00000023408 | SAMD4A | -1.027279544 | 0.000182398 | DOWN |
| ENSSSCG00000034723 | PYCR1 | -1.027929379 | 0.007034877 | DOWN |
| ENSSSCG00000001787 | IL16 | -1.02839751 | 0.003599713 | DOWN |
| ENSSSCG00000012405 | RPS4X | -1.029081044 | 3.29744E-07 | DOWN |
| ENSSSCG00000010027 | PATZ1 | -1.029211988 | 7.17155E-06 | DOWN |
| ENSSSCG00000008698 | RGS12 | -1.03021064 | 0.000811634 | DOWN |
| ENSSSCG00000034227 | RPL35 | -1.030427799 | 6.0591E-05 | DOWN |
| ENSSSCG00000000435 | ARHGEF25 | -1.030442418 | 0.008144726 | DOWN |
| ENSSSCG00000004002 | RPS5 | -1.031312722 | 3.53822E-05 | DOWN |
| ENSSSCG00000038025 | NRBP2 | -1.031867951 | 5.90292E-08 | DOWN |
| ENSSSCG00000004142 | CITED2 | -1.032192594 | 0.004057048 | DOWN |
| ENSSSCG00000015999 | FKBP7 | -1.033365178 | 0.007309607 | DOWN |
| ENSSSCG00000001242 | GABBR1 | -1.034184715 | 1.05704E-05 | DOWN |
| ENSSSCG00000026116 | FHOD1 | -1.035465251 | 3.55528E-05 | DOWN |
| ENSSSCG00000012077 | MX1 | -1.035488401 | 0.00957655 | DOWN |
| ENSSSCG00000026167 | NA | -1.035613663 | 0.015415962 | DOWN |
| ENSSSCG00000002937 | ZNF420 | -1.038067941 | 8.36496E-06 | DOWN |
| ENSSSCG00000030581 | VGLL4 | -1.039957361 | 0.005384043 | DOWN |
| ENSSSCG00000003473 | RSG1 | -1.041796782 | 0.004211871 | DOWN |
| ENSSSCG00000032632 | CMTM4 | -1.042924638 | 0.003159909 | DOWN |
| ENSSSCG00000009019 | RPS3A | -1.044051043 | 1.20739E-05 | DOWN |
| ENSSSCG00000006947 | SYDE2 | -1.045408227 | 0.000397115 | DOWN |
| ENSSSCG00000001867 | PSTPIP1 | -1.047146545 | 0.018561101 | DOWN |
| ENSSSCG00000008557 | EMILIN1 | -1.047286164 | 0.009504951 | DOWN |
| ENSSSCG00000015410 | PHTF2 | -1.047611778 | 0.000604491 | DOWN |
| ENSSSCG00000007736 | TMEM248 | -1.048637336 | 7.34758E-07 | DOWN |
| ENSSSCG00000001620 | MDFI | -1.050027218 | 0.049973563 | DOWN |
| ENSSSCG00000002045 | NA | -1.052196665 | 0.022329939 | DOWN |
| ENSSSCG00000023434 | PPM1L | -1.05285674 | 0.021535257 | DOWN |
| ENSSSCG00000040470 | RPS20 | -1.053446577 | 0.000392559 | DOWN |
| ENSSSCG00000002654 | FBXO31 | -1.053740078 | 7.54252E-09 | DOWN |
| ENSSSCG00000003839 | NA | -1.053924597 | 0.000374272 | DOWN |
| ENSSSCG00000009716 | SH3RF1 | -1.05426548 | 0.000507507 | DOWN |
| ENSSSCG00000039373 | SNX24 | -1.054858108 | 0.000418738 | DOWN |
| ENSSSCG00000006081 | RPL30 | -1.054909434 | 9.27919E-05 | DOWN |
| ENSSSCG00000001078 | MBOAT1 | -1.055129814 | 0.000145358 | DOWN |
| ENSSSCG00000037360 | CST3 | -1.055602671 | 0.017657454 | DOWN |
| ENSSSCG00000022288 | STK38L | -1.055952921 | 2.28146E-05 | DOWN |
| ENSSSCG00000025667 | FBXO2 | -1.056892113 | 0.031127446 | DOWN |
| ENSSSCG00000028661 | ENKD1 | -1.056969135 | 0.02108102 | DOWN |
| ENSSSCG00000001619 | FOXP4 | -1.058511317 | 0.003597357 | DOWN |
| ENSSSCG00000014670 | TRIM5 | -1.061003939 | 0.022100355 | DOWN |
| ENSSSCG00000028192 | DNAJC28 | -1.061166054 | 0.010873064 | DOWN |
| ENSSSCG00000037224 | DEXI | -1.061217077 | 0.003142819 | DOWN |
| ENSSSCG00000009502 | UGGT2 | -1.0616733 | 6.91085E-05 | DOWN |
| ENSSSCG00000009963 | TPST2 | -1.063652068 | 7.31596E-07 | DOWN |
| ENSSSCG00000035446 | CAVIN1 | -1.064851408 | 7.03086E-05 | DOWN |
| ENSSSCG00000034213 | ACER2 | -1.065648388 | 0.001909371 | DOWN |
| ENSSSCG00000028850 | RPL26 | -1.065947977 | 7.5183E-06 | DOWN |
| ENSSSCG00000017526 | COPZ2 | -1.066939998 | 0.002221211 | DOWN |
| ENSSSCG00000022741 | PDGFRB | -1.067147561 | 0.011362168 | DOWN |
| ENSSSCG00000037647 | TFDP2 | -1.067176643 | 0.007127614 | DOWN |
| ENSSSCG00000029715 | OLFM1 | -1.068432407 | 0.002022049 | DOWN |
| ENSSSCG00000002704 | KARS | -1.070230662 | 6.31508E-09 | DOWN |
| ENSSSCG00000008036 | SYNGR3 | -1.07280974 | 0.024582585 | DOWN |
| ENSSSCG00000015324 | GNG11 | -1.075287137 | 0.015580042 | DOWN |
| ENSSSCG00000004055 | RSPH3 | -1.075694471 | 0.003912084 | DOWN |
| ENSSSCG00000027812 | IQSEC1 | -1.078130427 | 4.1869E-05 | DOWN |
| ENSSSCG00000029003 | NA | -1.078736978 | 5.08516E-05 | DOWN |
| ENSSSCG00000011226 | TGFBR2 | -1.078913767 | 6.36916E-05 | DOWN |
| ENSSSCG00000040013 | MTUS1 | -1.079401949 | 0.04374679 | DOWN |
| ENSSSCG00000027198 | PDK2 | -1.079688326 | 3.17517E-05 | DOWN |
| ENSSSCG00000008378 | FAM161A | -1.079857543 | 8.93119E-07 | DOWN |
| ENSSSCG00000003930 | NA | -1.080510548 | 0.000256817 | DOWN |
| ENSSSCG00000034259 | PMEPA1 | -1.08075822 | 0.000102386 | DOWN |
| ENSSSCG00000015071 | SIK3 | -1.081113971 | 0.005443485 | DOWN |
| ENSSSCG00000013074 | RAB3IL1 | -1.081540239 | 5.19799E-05 | DOWN |
| ENSSSCG00000035379 | JCHAIN | -1.08166889 | 0.007642412 | DOWN |
| ENSSSCG00000039740 | NA | -1.082103806 | 0.002013849 | DOWN |
| ENSSSCG00000014133 | RPS23 | -1.083282704 | 0.001366915 | DOWN |
| ENSSSCG00000011272 | NA | -1.08450741 | 1.50186E-06 | DOWN |
| ENSSSCG00000012265 | CHST7 | -1.084664525 | 0.039979768 | DOWN |
| ENSSSCG00000016792 | RETREG1 | -1.085526676 | 0.024284939 | DOWN |
| ENSSSCG00000036356 | ZSCAN2 | -1.086504857 | 0.003918941 | DOWN |
| ENSSSCG00000010385 | VSTM4 | -1.087424519 | 1.22295E-05 | DOWN |
| ENSSSCG00000010734 | NA | -1.089286253 | 0.003053805 | DOWN |
| ENSSSCG00000000204 | PRPF40B | -1.089899701 | 3.88737E-06 | DOWN |
| ENSSSCG00000036569 | PTP4A3 | -1.090500615 | 0.005105408 | DOWN |
| ENSSSCG00000000722 | RAD51AP1 | -1.090637948 | 0.030594087 | DOWN |
| ENSSSCG00000037144 | CDKN1B | -1.090704797 | 4.8427E-08 | DOWN |
| ENSSSCG00000002933 | ZNF382 | -1.090790835 | 0.014903643 | DOWN |
| ENSSSCG00000036206 | C3orf58 | -1.092000414 | 2.20085E-07 | DOWN |
| ENSSSCG00000016435 | SMARCD3 | -1.092932776 | 6.68124E-05 | DOWN |
| ENSSSCG00000037433 | C3orf18 | -1.093010464 | 0.003245107 | DOWN |
| ENSSSCG00000012295 | MAGIX | -1.093564068 | 0.048243103 | DOWN |
| ENSSSCG00000006664 | MTMR11 | -1.094259103 | 0.000954509 | DOWN |
| ENSSSCG00000003165 | RPS11 | -1.09431608 | 5.39303E-05 | DOWN |
| ENSSSCG00000002385 | TGFB3 | -1.094325351 | 0.000664409 | DOWN |
| ENSSSCG00000006218 | PDE7A | -1.095827903 | 0.000347737 | DOWN |
| ENSSSCG00000028282 | SLC1A4 | -1.097171432 | 5.48089E-07 | DOWN |
| ENSSSCG00000016092 | SGO2 | -1.098768748 | 0.002689577 | DOWN |
| ENSSSCG00000020817 | RPS16 | -1.099859466 | 0.000174733 | DOWN |
| ENSSSCG00000028670 | NA | -1.100210367 | 0.000531214 | DOWN |
| ENSSSCG00000033326 | RPL37 | -1.100789814 | 0.000485601 | DOWN |
| ENSSSCG00000003049 | NA | -1.101266453 | 0.030259454 | DOWN |
| ENSSSCG00000002755 | NFAT5 | -1.10251814 | 3.15127E-05 | DOWN |
| ENSSSCG00000017087 | GM2A | -1.102525099 | 4.53284E-06 | DOWN |
| ENSSSCG00000013906 | KXD1 | -1.10365163 | 3.99644E-06 | DOWN |
| ENSSSCG00000015794 | C4orf47 | -1.104374566 | 0.047076509 | DOWN |
| ENSSSCG00000032861 | NUAK1 | -1.104792608 | 0.000364296 | DOWN |
| ENSSSCG00000034191 | SOX6 | -1.104813251 | 3.32076E-05 | DOWN |
| ENSSSCG00000034027 | ZNF544 | -1.10497306 | 0.001907849 | DOWN |
| ENSSSCG00000035389 | NA | -1.105613051 | 0.001714537 | DOWN |
| ENSSSCG00000009048 | GAB1 | -1.106216317 | 0.000834772 | DOWN |
| ENSSSCG00000034630 | ABI2 | -1.107310047 | 2.10547E-12 | DOWN |
| ENSSSCG00000003017 | TGFB1 | -1.107612273 | 0.000112192 | DOWN |
| ENSSSCG00000011495 | PRICKLE2 | -1.108238962 | 0.015101281 | DOWN |
| ENSSSCG00000039194 | KANK2 | -1.109101076 | 0.02056346 | DOWN |
| ENSSSCG00000040273 | NA | -1.109636668 | 1.58692E-05 | DOWN |
| ENSSSCG00000012971 | EFEMP2 | -1.111064596 | 1.53562E-11 | DOWN |
| ENSSSCG00000003108 | NPAS1 | -1.111799194 | 0.035598534 | DOWN |
| ENSSSCG00000025106 | NA | -1.114385468 | 8.76949E-05 | DOWN |
| ENSSSCG00000032157 | NA | -1.116789847 | 0.010069068 | DOWN |
| ENSSSCG00000006889 | ARHGAP29 | -1.117118159 | 4.64586E-06 | DOWN |
| ENSSSCG00000008266 | LOXL3 | -1.118069216 | 0.001475005 | DOWN |
| ENSSSCG00000003989 | NA | -1.118987183 | 0.002458385 | DOWN |
| ENSSSCG00000035007 | RPL17-C18orf32 | -1.119224897 | 2.15711E-06 | DOWN |
| ENSSSCG00000001872 | LINGO1 | -1.119961701 | 0.014599043 | DOWN |
| ENSSSCG00000025335 | ZSCAN31 | -1.124618663 | 0.000965989 | DOWN |
| ENSSSCG00000034229 | MIS18A | -1.124642413 | 0.038537139 | DOWN |
| ENSSSCG00000025822 | SFRP1 | -1.125613446 | 0.020640636 | DOWN |
| ENSSSCG00000016616 | FAM3C | -1.1262413 | 1.47846E-06 | DOWN |
| ENSSSCG00000028169 | NCAPG2 | -1.126399872 | 0.003184665 | DOWN |
| ENSSSCG00000027417 | LDLRAD4 | -1.126969764 | 0.044728403 | DOWN |
| ENSSSCG00000028672 | GCKR | -1.127424424 | 0.01256597 | DOWN |
| ENSSSCG00000024818 | RCAN3 | -1.128424343 | 0.000437652 | DOWN |
| ENSSSCG00000030217 | COLGALT2 | -1.131053819 | 0.015280839 | DOWN |
| ENSSSCG00000032929 | ENKUR | -1.132964944 | 0.000437652 | DOWN |
| ENSSSCG00000032250 | SRRM3 | -1.134738944 | 0.006863979 | DOWN |
| ENSSSCG00000008787 | KLHL5 | -1.134771313 | 0.000118556 | DOWN |
| ENSSSCG00000006333 | NUF2 | -1.135389156 | 0.030281966 | DOWN |
| ENSSSCG00000037459 | TCTA | -1.136490023 | 7.14412E-05 | DOWN |
| ENSSSCG00000024377 | RPS6KA5 | -1.136756956 | 0.017215323 | DOWN |
| ENSSSCG00000030065 | ZBTB47 | -1.137024764 | 7.03374E-06 | DOWN |
| ENSSSCG00000013773 | ADGRL1 | -1.137898981 | 7.63632E-05 | DOWN |
| ENSSSCG00000013282 | ACCS | -1.139309713 | 0.000122964 | DOWN |
| ENSSSCG00000036804 | TCEA2 | -1.140844164 | 4.42617E-06 | DOWN |
| ENSSSCG00000012525 | BEX1 | -1.143588787 | 0.019767987 | DOWN |
| ENSSSCG00000032473 | NYNRIN | -1.144038621 | 0.004769089 | DOWN |
| ENSSSCG00000001755 | HYKK | -1.144552263 | 0.04826962 | DOWN |
| ENSSSCG00000009357 | SMAD9 | -1.147606811 | 2.75879E-06 | DOWN |
| ENSSSCG00000006720 | HAO2 | -1.148846901 | 0.019386806 | DOWN |
| ENSSSCG00000022842 | NA | -1.148945455 | 0.005926501 | DOWN |
| ENSSSCG00000035904 | RPL7A | -1.149224667 | 1.93E-05 | DOWN |
| ENSSSCG00000028771 | MEF2A | -1.150244895 | 5.80412E-12 | DOWN |
| ENSSSCG00000036933 | NR1D1 | -1.150808117 | 0.011224049 | DOWN |
| ENSSSCG00000013895 | MAST3 | -1.152268579 | 0.013390844 | DOWN |
| ENSSSCG00000032111 | RPL7 | -1.15408128 | 7.39384E-08 | DOWN |
| ENSSSCG00000017788 | TP53I13 | -1.154700872 | 0.000128378 | DOWN |
| ENSSSCG00000017389 | RAMP2 | -1.155898207 | 0.018787924 | DOWN |
| ENSSSCG00000033883 | NA | -1.157164141 | 0.016451216 | DOWN |
| ENSSSCG00000038132 | IRF2BP2 | -1.157218124 | 4.20769E-07 | DOWN |
| ENSSSCG00000011775 | KLHL24 | -1.15804605 | 0.000381033 | DOWN |
| ENSSSCG00000012759 | ZNF185 | -1.158320159 | 0.045371404 | DOWN |
| ENSSSCG00000023639 | NRM | -1.160570291 | 0.004474824 | DOWN |
| ENSSSCG00000008020 | IFT140 | -1.161080157 | 8.86561E-06 | DOWN |
| ENSSSCG00000002783 | SLC9A5 | -1.163568141 | 0.00420993 | DOWN |
| ENSSSCG00000024260 | RPL11 | -1.16363917 | 3.03515E-05 | DOWN |
| ENSSSCG00000016432 | PRKAG2 | -1.163686452 | 0.000120896 | DOWN |
| ENSSSCG00000007067 | JAG1 | -1.165187396 | 0.004639245 | DOWN |
| ENSSSCG00000006957 | RHPN1 | -1.166864961 | 0.001370797 | DOWN |
| ENSSSCG00000005510 | PHF19 | -1.167238365 | 0.000195896 | DOWN |
| ENSSSCG00000014908 | CCDC89 | -1.167622977 | 0.006909221 | DOWN |
| ENSSSCG00000016164 | IKZF2 | -1.168473738 | 0.024313131 | DOWN |
| ENSSSCG00000040617 | TNFAIP8 | -1.168822412 | 0.001471054 | DOWN |
| ENSSSCG00000014255 | SLC12A2 | -1.170893347 | 0.000324664 | DOWN |
| ENSSSCG00000014855 | RPS3 | -1.171249423 | 2.04247E-05 | DOWN |
| ENSSSCG00000001672 | NA | -1.172661157 | 0.012821378 | DOWN |
| ENSSSCG00000001727 | TNFRSF21 | -1.173682974 | 0.007005178 | DOWN |
| ENSSSCG00000008170 | NA | -1.174302529 | 1.03338E-05 | DOWN |
| ENSSSCG00000039046 | RPL36A-HNRNPH2 | -1.175780474 | 2.00765E-05 | DOWN |
| ENSSSCG00000000774 | USP18 | -1.176435428 | 0.011668173 | DOWN |
| ENSSSCG00000032028 | EIF4B | -1.182509292 | 1.6895E-10 | DOWN |
| ENSSSCG00000004962 | CORO2B | -1.182527502 | 0.00113518 | DOWN |
| ENSSSCG00000004439 | TSPYL4 | -1.182930552 | 4.80027E-05 | DOWN |
| ENSSSCG00000023814 | FAIM | -1.183245914 | 0.044083416 | DOWN |
| ENSSSCG00000022797 | PPP1R3B | -1.183473437 | 0.008617043 | DOWN |
| ENSSSCG00000039130 | RPL6 | -1.186540102 | 5.22672E-08 | DOWN |
| ENSSSCG00000010416 | ZNF32 | -1.190442669 | 0.000158108 | DOWN |
| ENSSSCG00000003403 | CENPS | -1.193245427 | 0.006769415 | DOWN |
| ENSSSCG00000033019 | NA | -1.19337361 | 1.33708E-07 | DOWN |
| ENSSSCG00000031849 | NA | -1.19403812 | 0.008392691 | DOWN |
| ENSSSCG00000003491 | AKR7A2 | -1.194378881 | 1.4199E-05 | DOWN |
| ENSSSCG00000016571 | NA | -1.195906781 | 0.00196897 | DOWN |
| ENSSSCG00000012426 | ZDHHC15 | -1.19735094 | 0.002037745 | DOWN |
| ENSSSCG00000039756 | FOXC1 | -1.199138296 | 0.000617179 | DOWN |
| ENSSSCG00000006988 | PDGFRL | -1.199275636 | 0.031241904 | DOWN |
| ENSSSCG00000004856 | NFATC1 | -1.202540994 | 8.01522E-05 | DOWN |
| ENSSSCG00000031615 | SERPINB9 | -1.202580031 | 0.013286078 | DOWN |
| ENSSSCG00000038958 | DNM3 | -1.202899884 | 2.86359E-05 | DOWN |
| ENSSSCG00000000402 | RBMS2 | -1.206326055 | 1.23133E-06 | DOWN |
| ENSSSCG00000025928 | NA | -1.207407603 | 2.16713E-05 | DOWN |
| ENSSSCG00000008504 | CRIM1 | -1.207511331 | 0.025803871 | DOWN |
| ENSSSCG00000037406 | TMEM136 | -1.208302003 | 0.002468379 | DOWN |
| ENSSSCG00000014015 | MRNIP | -1.209180214 | 0.011723502 | DOWN |
| ENSSSCG00000003579 | AHDC1 | -1.210899442 | 0.000196047 | DOWN |
| ENSSSCG00000029783 | MKX | -1.210908375 | 2.56848E-08 | DOWN |
| ENSSSCG00000008973 | NAAA | -1.211123821 | 0.026188072 | DOWN |
| ENSSSCG00000039594 | SSBP3 | -1.212621607 | 0.000357959 | DOWN |
| ENSSSCG00000013911 | TMEM59L | -1.213441395 | 0.000650478 | DOWN |
| ENSSSCG00000036411 | ACOT13 | -1.214652021 | 0.000153316 | DOWN |
| ENSSSCG00000001100 | CARMIL1 | -1.215206801 | 1.00927E-05 | DOWN |
| ENSSSCG00000040419 | NA | -1.215467855 | 0.001045965 | DOWN |
| ENSSSCG00000017565 | SPATA20 | -1.21663408 | 0.000925331 | DOWN |
| ENSSSCG00000011731 | SMC4 | -1.216865206 | 0.010959568 | DOWN |
| ENSSSCG00000011811 | LPP | -1.21706215 | 4.56733E-06 | DOWN |
| ENSSSCG00000017143 | CEP131 | -1.217660414 | 0.013651382 | DOWN |
| ENSSSCG00000011765 | USP13 | -1.218421452 | 0.008805681 | DOWN |
| ENSSSCG00000028995 | TLE2 | -1.219226004 | 0.020747211 | DOWN |
| ENSSSCG00000027660 | IFI44L | -1.219454701 | 0.013916651 | DOWN |
| ENSSSCG00000011522 | CNTN3 | -1.219753388 | 0.043475034 | DOWN |
| ENSSSCG00000003854 | ECHDC2 | -1.220593649 | 1.28663E-05 | DOWN |
| ENSSSCG00000038597 | KLHDC9 | -1.22147633 | 0.021200813 | DOWN |
| ENSSSCG00000001977 | STXBP6 | -1.221778138 | 0.00080991 | DOWN |
| ENSSSCG00000017416 | DHX58 | -1.225330974 | 0.00523001 | DOWN |
| ENSSSCG00000032841 | NEAT1_1 | -1.225631617 | 0.001524316 | DOWN |
| ENSSSCG00000008545 | ZNF512 | -1.225964318 | 5.7762E-08 | DOWN |
| ENSSSCG00000036868 | CTNNBIP1 | -1.226146406 | 0.000495612 | DOWN |
| ENSSSCG00000008792 | N4BP2 | -1.228636752 | 4.48375E-05 | DOWN |
| ENSSSCG00000016520 | CREB3L2 | -1.230718645 | 0.000732859 | DOWN |
| ENSSSCG00000008179 | REV1 | -1.230997804 | 8.00636E-07 | DOWN |
| ENSSSCG00000040053 | LSMEM1 | -1.23102262 | 0.012918071 | DOWN |
| ENSSSCG00000017759 | ALDOC | -1.232688884 | 0.002207427 | DOWN |
| ENSSSCG00000031838 | RPS27 | -1.233121616 | 0.000150177 | DOWN |
| ENSSSCG00000017387 | WNK4 | -1.23386812 | 0.04943725 | DOWN |
| ENSSSCG00000002025 | SLC22A17 | -1.233918832 | 2.43317E-12 | DOWN |
| ENSSSCG00000038084 | NA | -1.234043073 | 0.000351358 | DOWN |
| ENSSSCG00000002906 | ETV2 | -1.234216821 | 0.044951085 | DOWN |
| ENSSSCG00000022296 | CDK15 | -1.234599903 | 0.002501824 | DOWN |
| ENSSSCG00000028740 | CTDSPL | -1.235025212 | 0.032747164 | DOWN |
| ENSSSCG00000032969 | NA | -1.237338101 | 9.57903E-06 | DOWN |
| ENSSSCG00000013064 | EEF1G | -1.237453004 | 9.08391E-06 | DOWN |
| ENSSSCG00000015982 | HOXD9 | -1.237469042 | 1.92815E-09 | DOWN |
| ENSSSCG00000037614 | NEAT1_3 | -1.24251951 | 0.00134651 | DOWN |
| ENSSSCG00000027890 | NA | -1.243864388 | 0.002021203 | DOWN |
| ENSSSCG00000005004 | LRR1 | -1.244320992 | 0.000366091 | DOWN |
| ENSSSCG00000009268 | CRYL1 | -1.244372321 | 8.46855E-08 | DOWN |
| ENSSSCG00000012952 | TMEM151A | -1.245861695 | 0.041105439 | DOWN |
| ENSSSCG00000040719 | KIAA0040 | -1.246256064 | 5.09364E-05 | DOWN |
| ENSSSCG00000031954 | SLC39A10 | -1.248583562 | 1.64844E-06 | DOWN |
| ENSSSCG00000034757 | FGF11 | -1.24919107 | 0.004794705 | DOWN |
| ENSSSCG00000021414 | NA | -1.249745541 | 0.021789291 | DOWN |
| ENSSSCG00000037376 | MXRA7 | -1.249850187 | 4.23781E-05 | DOWN |
| ENSSSCG00000013020 | MAP4K2 | -1.25237509 | 0.00775585 | DOWN |
| ENSSSCG00000032566 | ZHX3 | -1.252746737 | 1.44656E-07 | DOWN |
| ENSSSCG00000026425 | ADAMTSL2 | -1.253732182 | 0.003819707 | DOWN |
| ENSSSCG00000022774 | NOP53 | -1.257419668 | 4.07487E-07 | DOWN |
| ENSSSCG00000000859 | PARPBP | -1.260058291 | 0.009086865 | DOWN |
| ENSSSCG00000023142 | GAMT | -1.260268486 | 9.9229E-09 | DOWN |
| ENSSSCG00000021646 | KLF9 | -1.260920244 | 0.00017991 | DOWN |
| ENSSSCG00000012546 | NRK | -1.261653343 | 0.002037415 | DOWN |
| ENSSSCG00000039793 | NA | -1.261915725 | 0.00017818 | DOWN |
| ENSSSCG00000028108 | ASAP3 | -1.263806317 | 1.36999E-06 | DOWN |
| ENSSSCG00000014303 | JADE2 | -1.263919164 | 0.00051935 | DOWN |
| ENSSSCG00000039546 | KNTC1 | -1.265509107 | 0.045320006 | DOWN |
| ENSSSCG00000025675 | EEF2 | -1.267993427 | 5.18458E-09 | DOWN |
| ENSSSCG00000029666 | HOXA13 | -1.268987825 | 2.78623E-07 | DOWN |
| ENSSSCG00000015462 | TPK1 | -1.276845437 | 4.68348E-05 | DOWN |
| ENSSSCG00000025141 | PCDHGB1 | -1.278824488 | 0.013519677 | DOWN |
| ENSSSCG00000012532 | TCEAL1 | -1.27941819 | 1.07271E-05 | DOWN |
| ENSSSCG00000006954 | EEF1D | -1.279821952 | 3.02338E-06 | DOWN |
| ENSSSCG00000005232 | SMARCA2 | -1.281705286 | 6.03378E-05 | DOWN |
| ENSSSCG00000004193 | ENPP1 | -1.283994631 | 2.77534E-05 | DOWN |
| ENSSSCG00000012181 | PDK3 | -1.284346507 | 4.67307E-10 | DOWN |
| ENSSSCG00000015885 | PLA2R1 | -1.286024147 | 0.016483524 | DOWN |
| ENSSSCG00000030309 | NDRG2 | -1.286058028 | 0.000545933 | DOWN |
| ENSSSCG00000004290 | TBX18 | -1.286438783 | 0.000409115 | DOWN |
| ENSSSCG00000015022 | LAYN | -1.286808664 | 0.00054023 | DOWN |
| ENSSSCG00000000483 | NA | -1.288441172 | 0.000416167 | DOWN |
| ENSSSCG00000038635 | ZNF554 | -1.288659694 | 0.003462982 | DOWN |
| ENSSSCG00000007567 | CHST12 | -1.288674514 | 5.0683E-05 | DOWN |
| ENSSSCG00000029305 | FNDC4 | -1.288847431 | 0.004323964 | DOWN |
| ENSSSCG00000003740 | ZSCAN30 | -1.288883766 | 0.028442221 | DOWN |
| ENSSSCG00000022609 | WWP2 | -1.288995082 | 0.038738159 | DOWN |
| ENSSSCG00000021757 | ZNF700 | -1.289126595 | 0.002671458 | DOWN |
| ENSSSCG00000012784 | SSR4 | -1.290963975 | 0.000352917 | DOWN |
| ENSSSCG00000023653 | GLIS2 | -1.291583688 | 0.003523378 | DOWN |
| ENSSSCG00000014173 | LNPEP | -1.292100358 | 8.86683E-07 | DOWN |
| ENSSSCG00000038452 | ADAMTS17 | -1.292769908 | 0.000885435 | DOWN |
| ENSSSCG00000026780 | NA | -1.295227552 | 0.00157259 | DOWN |
| ENSSSCG00000028981 | ZNF367 | -1.29581134 | 0.011420483 | DOWN |
| ENSSSCG00000024669 | NA | -1.297189287 | 6.1016E-05 | DOWN |
| ENSSSCG00000031088 | NA | -1.298385103 | 1.36287E-06 | DOWN |
| ENSSSCG00000024132 | TMEM47 | -1.301740004 | 4.25492E-05 | DOWN |
| ENSSSCG00000032031 | QARS | -1.302217409 | 1.86725E-08 | DOWN |
| ENSSSCG00000008677 | NA | -1.302710119 | 0.034854241 | DOWN |
| ENSSSCG00000006899 | RPL5 | -1.306339027 | 8.99775E-07 | DOWN |
| ENSSSCG00000038660 | HAAO | -1.30694959 | 0.028348845 | DOWN |
| ENSSSCG00000021825 | NA | -1.307542457 | 0.000112465 | DOWN |
| ENSSSCG00000036083 | NA | -1.308211353 | 1.96961E-07 | DOWN |
| ENSSSCG00000021834 | ERCC6L | -1.308443926 | 0.010731918 | DOWN |
| ENSSSCG00000034972 | CNIH3 | -1.309208201 | 0.009495037 | DOWN |
| ENSSSCG00000037508 | GSN | -1.309210741 | 7.67453E-05 | DOWN |
| ENSSSCG00000021440 | GPSM2 | -1.310108582 | 0.000435444 | DOWN |
| ENSSSCG00000037318 | TRABD2B | -1.310380582 | 0.015857789 | DOWN |
| ENSSSCG00000010903 | LHX9 | -1.311607036 | 0.000327851 | DOWN |
| ENSSSCG00000003038 | GRIK5 | -1.31184166 | 1.86149E-05 | DOWN |
| ENSSSCG00000004087 | CCDC170 | -1.311947793 | 0.00030058 | DOWN |
| ENSSSCG00000034189 | LDOC1 | -1.312427101 | 2.08907E-07 | DOWN |
| ENSSSCG00000008767 | NA | -1.312738022 | 0.000283091 | DOWN |
| ENSSSCG00000007235 | TPX2 | -1.313335758 | 0.025856366 | DOWN |
| ENSSSCG00000036213 | FGF2 | -1.315535277 | 0.000247936 | DOWN |
| ENSSSCG00000035890 | NA | -1.31686701 | 0.043977621 | DOWN |
| ENSSSCG00000007268 | E2F1 | -1.317923574 | 0.017795278 | DOWN |
| ENSSSCG00000028725 | TMEM102 | -1.318816403 | 6.10498E-05 | DOWN |
| ENSSSCG00000023133 | OSBPL6 | -1.319009226 | 2.2598E-06 | DOWN |
| ENSSSCG00000017254 | MAP2K6 | -1.319299254 | 0.010391918 | DOWN |
| ENSSSCG00000031821 | HASPIN | -1.319693066 | 0.013527381 | DOWN |
| ENSSSCG00000002902 | ATP4A | -1.320502157 | 0.024341976 | DOWN |
| ENSSSCG00000009071 | JADE1 | -1.320822145 | 8.11031E-07 | DOWN |
| ENSSSCG00000034863 | PARD6A | -1.321316508 | 0.021192484 | DOWN |
| ENSSSCG00000000600 | EPS8 | -1.322085402 | 1.14899E-06 | DOWN |
| ENSSSCG00000035364 | VKORC1 | -1.322145311 | 2.90723E-05 | DOWN |
| ENSSSCG00000002930 | ZNF260 | -1.322295987 | 0.000930761 | DOWN |
| ENSSSCG00000016128 | EEF1B2 | -1.322477444 | 4.90811E-07 | DOWN |
| ENSSSCG00000002850 | NA | -1.323487341 | 0.005308152 | DOWN |
| ENSSSCG00000000217 | NA | -1.326508736 | 0.007186223 | DOWN |
| ENSSSCG00000037854 | L3HYPDH | -1.32881337 | 2.76498E-08 | DOWN |
| ENSSSCG00000002274 | HSPA2 | -1.328947598 | 6.73769E-06 | DOWN |
| ENSSSCG00000031741 | NA | -1.331246898 | 0.018127239 | DOWN |
| ENSSSCG00000000370 | DGKA | -1.332620432 | 7.34139E-06 | DOWN |
| ENSSSCG00000000915 | DCN | -1.33406552 | 7.00022E-05 | DOWN |
| ENSSSCG00000013008 | CDCA5 | -1.334261852 | 0.0419464 | DOWN |
| ENSSSCG00000026689 | CCDC114 | -1.334328186 | 0.046539521 | DOWN |
| ENSSSCG00000036396 | NEAT1_2 | -1.336297298 | 0.000407123 | DOWN |
| ENSSSCG00000017338 | PLCD3 | -1.337264956 | 7.925E-06 | DOWN |
| ENSSSCG00000009881 | OAS2 | -1.337613165 | 0.03566677 | DOWN |
| ENSSSCG00000012520 | BHLHB9 | -1.33913089 | 6.31508E-09 | DOWN |
| ENSSSCG00000037483 | RFTN2 | -1.339522608 | 0.00025499 | DOWN |
| ENSSSCG00000009146 | RPL34 | -1.340146245 | 1.31544E-06 | DOWN |
| ENSSSCG00000017255 | ABCA5 | -1.340856869 | 0.004941455 | DOWN |
| ENSSSCG00000015958 | NA | -1.342035929 | 7.77846E-06 | DOWN |
| ENSSSCG00000006911 | TGFBR3 | -1.342242264 | 5.08516E-05 | DOWN |
| ENSSSCG00000039797 | APCDD1 | -1.34321958 | 0.005637782 | DOWN |
| ENSSSCG00000021843 | SYT5 | -1.347482586 | 0.00918286 | DOWN |
| ENSSSCG00000036363 | PDE4C | -1.347569638 | 0.01983979 | DOWN |
| ENSSSCG00000039966 | NXPH4 | -1.347708407 | 0.000382109 | DOWN |
| ENSSSCG00000030113 | SHISA2 | -1.349088167 | 0.026407783 | DOWN |
| ENSSSCG00000026994 | ADAMTS10 | -1.349457213 | 1.48723E-11 | DOWN |
| ENSSSCG00000013010 | NAALADL1 | -1.350762918 | 0.030406118 | DOWN |
| ENSSSCG00000016836 | NADK2 | -1.351581278 | 1.35006E-05 | DOWN |
| ENSSSCG00000035757 | SSBP2 | -1.351834755 | 1.37142E-05 | DOWN |
| ENSSSCG00000029626 | NECAB3 | -1.355630234 | 0.049886069 | DOWN |
| ENSSSCG00000006296 | ATP1B1 | -1.356567132 | 2.76112E-05 | DOWN |
| ENSSSCG00000003344 | VWA1 | -1.356793374 | 0.00017742 | DOWN |
| ENSSSCG00000015036 | DIXDC1 | -1.358986889 | 0.000127856 | DOWN |
| ENSSSCG00000040837 | NA | -1.360440278 | 0.000473344 | DOWN |
| ENSSSCG00000011689 | PLOD2 | -1.36099422 | 0.000797525 | DOWN |
| ENSSSCG00000036679 | SORBS2 | -1.361204322 | 0.000429878 | DOWN |
| ENSSSCG00000016857 | DAB2 | -1.36372398 | 1.00053E-07 | DOWN |
| ENSSSCG00000010702 | PLEKHA1 | -1.365990314 | 6.70646E-08 | DOWN |
| ENSSSCG00000034879 | MAML2 | -1.369950996 | 0.000231525 | DOWN |
| ENSSSCG00000004898 | TNFRSF11A | -1.370840349 | 0.037472178 | DOWN |
| ENSSSCG00000026113 | ZBTB20 | -1.372163772 | 1.75344E-06 | DOWN |
| ENSSSCG00000007727 | AUTS2 | -1.37249623 | 2.00765E-05 | DOWN |
| ENSSSCG00000024604 | ATAT1 | -1.37284161 | 0.02866718 | DOWN |
| ENSSSCG00000034617 | RPS27A | -1.372899999 | 3.93182E-07 | DOWN |
| ENSSSCG00000040735 | DDAH1 | -1.37688598 | 3.85234E-06 | DOWN |
| ENSSSCG00000033783 | NA | -1.377456641 | 0.02809466 | DOWN |
| ENSSSCG00000005277 | GCNT1 | -1.385091284 | 0.000170418 | DOWN |
| ENSSSCG00000031370 | NA | -1.385259276 | 8.81218E-06 | DOWN |
| ENSSSCG00000021384 | BRIP1 | -1.386740344 | 0.010092278 | DOWN |
| ENSSSCG00000038598 | ADRB2 | -1.387006539 | 0.048790376 | DOWN |
| ENSSSCG00000015825 | ADGRA2 | -1.388470332 | 2.16713E-05 | DOWN |
| ENSSSCG00000023522 | TGM2 | -1.38888145 | 0.03221762 | DOWN |
| ENSSSCG00000000133 | TST | -1.388947443 | 0.009283392 | DOWN |
| ENSSSCG00000037241 | RGS2 | -1.389470706 | 0.047189948 | DOWN |
| ENSSSCG00000008590 | MFSD2B | -1.392169122 | 0.002771395 | DOWN |
| ENSSSCG00000036091 | MORC4 | -1.393200033 | 1.67398E-08 | DOWN |
| ENSSSCG00000018029 | NA | -1.394059653 | 5.53946E-08 | DOWN |
| ENSSSCG00000037970 | SOX8 | -1.394619064 | 1.94308E-05 | DOWN |
| ENSSSCG00000006213 | ADHFE1 | -1.396819217 | 0.001460164 | DOWN |
| ENSSSCG00000022306 | ROPN1L | -1.397332521 | 0.038845334 | DOWN |
| ENSSSCG00000011704 | WWTR1 | -1.398696366 | 6.18663E-06 | DOWN |
| ENSSSCG00000026142 | TNK1 | -1.400250161 | 0.034847518 | DOWN |
| ENSSSCG00000021232 | SYNC | -1.401719067 | 0.02056346 | DOWN |
| ENSSSCG00000009123 | CAMK2D | -1.402619841 | 8.46846E-20 | DOWN |
| ENSSSCG00000009729 | ZNF84 | -1.404371838 | 2.77677E-07 | DOWN |
| ENSSSCG00000011556 | NA | -1.405474367 | 0.016374299 | DOWN |
| ENSSSCG00000004971 | TLE3 | -1.406855867 | 0.000369256 | DOWN |
| ENSSSCG00000000090 | CBX7 | -1.407133021 | 0.000134582 | DOWN |
| ENSSSCG00000013869 | CPAMD8 | -1.409167711 | 0.004280719 | DOWN |
| ENSSSCG00000035997 | RPL10 | -1.410652715 | 1.89185E-06 | DOWN |
| ENSSSCG00000022280 | DACT3 | -1.412841137 | 0.000273926 | DOWN |
| ENSSSCG00000005030 | NID2 | -1.412992844 | 0.020250224 | DOWN |
| ENSSSCG00000014011 | RASGEF1C | -1.41413635 | 0.033845464 | DOWN |
| ENSSSCG00000010128 | SEPT5 | -1.414718084 | 3.26846E-05 | DOWN |
| ENSSSCG00000037733 | TRIQK | -1.414800213 | 2.73187E-06 | DOWN |
| ENSSSCG00000015328 | SGCE | -1.415115316 | 1.96579E-08 | DOWN |
| ENSSSCG00000008646 | RNF144A | -1.418529608 | 5.68073E-05 | DOWN |
| ENSSSCG00000006169 | ZFHX4 | -1.418581239 | 7.74646E-05 | DOWN |
| ENSSSCG00000021702 | XRRA1 | -1.42014988 | 0.008948814 | DOWN |
| ENSSSCG00000027928 | TMEM9 | -1.421801563 | 0.019392624 | DOWN |
| ENSSSCG00000012842 | RPLP2 | -1.423471034 | 0.000112318 | DOWN |
| ENSSSCG00000010101 | P2RX6 | -1.42350028 | 0.018339406 | DOWN |
| ENSSSCG00000005371 | HEMGN | -1.423706236 | 2.22181E-05 | DOWN |
| ENSSSCG00000012266 | SLC9A7 | -1.42498364 | 0.024693675 | DOWN |
| ENSSSCG00000008162 | IL1R1 | -1.429078011 | 4.67384E-08 | DOWN |
| ENSSSCG00000021204 | HOXA10 | -1.430263475 | 0.000143086 | DOWN |
| ENSSSCG00000012318 | MAGED1 | -1.430395664 | 2.10655E-05 | DOWN |
| ENSSSCG00000023028 | JAM2 | -1.431747318 | 0.000656417 | DOWN |
| ENSSSCG00000012700 | NA | -1.432103956 | 0.00134651 | DOWN |
| ENSSSCG00000015780 | STOX2 | -1.433291943 | 0.001946477 | DOWN |
| ENSSSCG00000005166 | MLLT3 | -1.433415676 | 0.002253221 | DOWN |
| ENSSSCG00000001818 | FES | -1.433796645 | 1.84708E-05 | DOWN |
| ENSSSCG00000006105 | GEM | -1.434782976 | 0.008353232 | DOWN |
| ENSSSCG00000018044 | ALDH3A1 | -1.43684868 | 0.005395351 | DOWN |
| ENSSSCG00000037898 | CXXC5 | -1.438164393 | 1.60129E-05 | DOWN |
| ENSSSCG00000008647 | CMPK2 | -1.43877104 | 0.001524316 | DOWN |
| ENSSSCG00000005110 | SYNE2 | -1.438821132 | 0.0115922 | DOWN |
| ENSSSCG00000008422 | NA | -1.44140881 | 1.97967E-07 | DOWN |
| ENSSSCG00000000010 | FBLN1 | -1.4417883 | 0.021767492 | DOWN |
| ENSSSCG00000010483 | PLCE1 | -1.445239198 | 0.005261458 | DOWN |
| ENSSSCG00000008117 | ZNF2 | -1.447149222 | 1.46731E-07 | DOWN |
| ENSSSCG00000025910 | ZNF277 | -1.451421001 | 9.51658E-08 | DOWN |
| ENSSSCG00000031461 | ZKSCAN1 | -1.451667756 | 0.000818455 | DOWN |
| ENSSSCG00000015119 | MFRP | -1.452873934 | 1.33262E-05 | DOWN |
| ENSSSCG00000010214 | NA | -1.453219167 | 0.046314641 | DOWN |
| ENSSSCG00000006533 | ADAM15 | -1.459027388 | 2.7791E-08 | DOWN |
| ENSSSCG00000030395 | ASB5 | -1.459084059 | 0.022437069 | DOWN |
| ENSSSCG00000002436 | TTC7B | -1.459229253 | 5.9593E-06 | DOWN |
| ENSSSCG00000024103 | ADPRHL1 | -1.460713788 | 0.030692503 | DOWN |
| ENSSSCG00000034288 | AP4M1 | -1.461027609 | 1.09115E-12 | DOWN |
| ENSSSCG00000013858 | CALR3 | -1.461886079 | 0.000404585 | DOWN |
| ENSSSCG00000029756 | ADGRG2 | -1.462054 | 0.000118613 | DOWN |
| ENSSSCG00000014137 | HAPLN1 | -1.463220843 | 0.035418604 | DOWN |
| ENSSSCG00000005785 | PCSK6 | -1.46407987 | 0.009756049 | DOWN |
| ENSSSCG00000007998 | RHBDL1 | -1.464547282 | 0.010650262 | DOWN |
| ENSSSCG00000040730 | NA | -1.466150835 | 0.016785056 | DOWN |
| ENSSSCG00000036512 | FGFRL1 | -1.466669049 | 0.000513532 | DOWN |
| ENSSSCG00000037967 | NA | -1.467234625 | 0.010696138 | DOWN |
| ENSSSCG00000008601 | SDC1 | -1.467455003 | 0.007474832 | DOWN |
| ENSSSCG00000000203 | KCNH3 | -1.469127862 | 0.02769251 | DOWN |
| ENSSSCG00000006082 | MATN2 | -1.471504925 | 6.53573E-05 | DOWN |
| ENSSSCG00000040525 | NA | -1.472023523 | 0.045841965 | DOWN |
| ENSSSCG00000040400 | GPR19 | -1.472678362 | 0.038661041 | DOWN |
| ENSSSCG00000012323 | TSPYL2 | -1.476005032 | 5.75231E-06 | DOWN |
| ENSSSCG00000014910 | NA | -1.477148655 | 0.007098651 | DOWN |
| ENSSSCG00000011030 | CUBN | -1.477884616 | 0.007963755 | DOWN |
| ENSSSCG00000004165 | PDE7B | -1.478373756 | 0.000639133 | DOWN |
| ENSSSCG00000014235 | SNCAIP | -1.481234523 | 0.001321078 | DOWN |
| ENSSSCG00000033410 | FAM212A | -1.485781427 | 0.011581223 | DOWN |
| ENSSSCG00000031072 | ZNF514 | -1.486458765 | 2.64002E-10 | DOWN |
| ENSSSCG00000031866 | TIMP3 | -1.486463382 | 0.00164865 | DOWN |
| ENSSSCG00000008834 | SPATA18 | -1.486964838 | 0.00466367 | DOWN |
| ENSSSCG00000006578 | S100A4 | -1.487925138 | 0.033153487 | DOWN |
| ENSSSCG00000016674 | MINDY4 | -1.489071496 | 0.001071317 | DOWN |
| ENSSSCG00000029503 | F7 | -1.490313591 | 0.003348762 | DOWN |
| ENSSSCG00000015871 | NR4A2 | -1.500315264 | 0.00737312 | DOWN |
| ENSSSCG00000022504 | CDON | -1.500898651 | 7.82767E-06 | DOWN |
| ENSSSCG00000034656 | RTN4R | -1.503157059 | 0.008294459 | DOWN |
| ENSSSCG00000009281 | SGCG | -1.504212406 | 2.36439E-07 | DOWN |
| ENSSSCG00000001517 | NA | -1.508350785 | 4.49316E-05 | DOWN |
| ENSSSCG00000011110 | CCDC3 | -1.509352594 | 0.006216496 | DOWN |
| ENSSSCG00000040267 | CYS1 | -1.510079907 | 4.17636E-05 | DOWN |
| ENSSSCG00000004835 | MAGEL2 | -1.510330217 | 0.007305467 | DOWN |
| ENSSSCG00000009050 | INPP4B | -1.510602711 | 0.001749501 | DOWN |
| ENSSSCG00000034821 | ARMCX4 | -1.511641343 | 6.70799E-07 | DOWN |
| ENSSSCG00000023243 | NFIA | -1.517220581 | 1.42518E-05 | DOWN |
| ENSSSCG00000012508 | NA | -1.52168107 | 0.000226803 | DOWN |
| ENSSSCG00000017904 | ENO3 | -1.522720046 | 0.000100001 | DOWN |
| ENSSSCG00000022846 | SLC4A3 | -1.524624583 | 0.017485391 | DOWN |
| ENSSSCG00000014803 | LRRC51 | -1.530568192 | 0.013396139 | DOWN |
| ENSSSCG00000035388 | C16orf46 | -1.531349066 | 0.011251401 | DOWN |
| ENSSSCG00000008687 | MXD4 | -1.532046997 | 3.20121E-05 | DOWN |
| ENSSSCG00000016453 | TCAF1 | -1.53340794 | 4.75851E-08 | DOWN |
| ENSSSCG00000005608 | ANGPTL2 | -1.535977157 | 1.67039E-05 | DOWN |
| ENSSSCG00000015345 | GLCCI1 | -1.536826575 | 3.17517E-05 | DOWN |
| ENSSSCG00000036383 | LGALS3BP | -1.54081308 | 1.73413E-06 | DOWN |
| ENSSSCG00000006624 | SELENBP1 | -1.542018082 | 0.007865181 | DOWN |
| ENSSSCG00000014909 | NA | -1.54365699 | 0.013311517 | DOWN |
| ENSSSCG00000010816 | TGFB2 | -1.546165196 | 0.000142821 | DOWN |
| ENSSSCG00000029264 | PKMYT1 | -1.546833589 | 0.022324496 | DOWN |
| ENSSSCG00000028052 | OBSL1 | -1.548395724 | 4.52049E-07 | DOWN |
| ENSSSCG00000010076 | ZNF70 | -1.549503773 | 6.16165E-11 | DOWN |
| ENSSSCG00000014978 | ANGPTL5 | -1.549916096 | 0.013258554 | DOWN |
| ENSSSCG00000032674 | TCEA3 | -1.551158089 | 1.299E-11 | DOWN |
| ENSSSCG00000009011 | FHDC1 | -1.551558272 | 0.033641636 | DOWN |
| ENSSSCG00000002283 | FUT8 | -1.55206409 | 1.32861E-07 | DOWN |
| ENSSSCG00000040334 | CBX6 | -1.553767864 | 1.33152E-07 | DOWN |
| ENSSSCG00000031141 | ABCA13 | -1.553971012 | 0.000571175 | DOWN |
| ENSSSCG00000038969 | DMPK | -1.554067915 | 9.30424E-05 | DOWN |
| ENSSSCG00000029509 | KIF22 | -1.554641949 | 0.01022438 | DOWN |
| ENSSSCG00000027428 | ENHO | -1.55465301 | 0.009364633 | DOWN |
| ENSSSCG00000016691 | JAZF1 | -1.55633621 | 1.87273E-05 | DOWN |
| ENSSSCG00000026387 | REEP6 | -1.55708258 | 0.018561679 | DOWN |
| ENSSSCG00000000073 | NA | -1.557156412 | 0.022032957 | DOWN |
| ENSSSCG00000001064 | GMPR | -1.558256139 | 0.026483154 | DOWN |
| ENSSSCG00000030241 | TSC22D3 | -1.563829879 | 3.4571E-08 | DOWN |
| ENSSSCG00000039703 | EEPD1 | -1.564605804 | 0.004467293 | DOWN |
| ENSSSCG00000024312 | ID4 | -1.564924236 | 0.000870464 | DOWN |
| ENSSSCG00000033338 | NA | -1.568084604 | 0.004055773 | DOWN |
| ENSSSCG00000030362 | NA | -1.568519368 | 0.001102803 | DOWN |
| ENSSSCG00000028777 | MYLK4 | -1.570661786 | 0.012146855 | DOWN |
| ENSSSCG00000016863 | OXCT1 | -1.570873049 | 3.33073E-08 | DOWN |
| ENSSSCG00000035267 | NA | -1.57179505 | 2.00399E-05 | DOWN |
| ENSSSCG00000034868 | NA | -1.577426921 | 3.4337E-09 | DOWN |
| ENSSSCG00000031594 | NA | -1.577812292 | 4.02168E-11 | DOWN |
| ENSSSCG00000008468 | PKDCC | -1.578735764 | 0.00200402 | DOWN |
| ENSSSCG00000032715 | CERS6 | -1.583414959 | 0.011939322 | DOWN |
| ENSSSCG00000039062 | NA | -1.584257346 | 0.012325822 | DOWN |
| ENSSSCG00000009232 | NKX6-1 | -1.588377992 | 0.047125711 | DOWN |
| ENSSSCG00000035217 | NA | -1.589554905 | 0.012579411 | DOWN |
| ENSSSCG00000001832 | ACAN | -1.59321149 | 0.019959782 | DOWN |
| ENSSSCG00000021208 | SELENOP | -1.595401087 | 1.22466E-05 | DOWN |
| ENSSSCG00000002032 | SLC7A8 | -1.601294015 | 0.000967075 | DOWN |
| ENSSSCG00000039875 | NKD1 | -1.605836492 | 0.000393743 | DOWN |
| ENSSSCG00000031666 | C11orf95 | -1.607391857 | 0.001478367 | DOWN |
| ENSSSCG00000038902 | KCNK6 | -1.607837318 | 3.39452E-06 | DOWN |
| ENSSSCG00000009330 | ALOX5AP | -1.609281802 | 4.30299E-06 | DOWN |
| ENSSSCG00000006338 | DDR2 | -1.611812021 | 7.82412E-09 | DOWN |
| ENSSSCG00000030359 | ARHGEF3 | -1.612252574 | 3.94508E-05 | DOWN |
| ENSSSCG00000029855 | LHFPL6 | -1.612718566 | 0.000166197 | DOWN |
| ENSSSCG00000024233 | RAB39B | -1.613603315 | 0.023456144 | DOWN |
| ENSSSCG00000017583 | SGCA | -1.614321858 | 0.000398242 | DOWN |
| ENSSSCG00000024071 | SCARF1 | -1.619988844 | 1.61654E-05 | DOWN |
| ENSSSCG00000040162 | NUPR1 | -1.626754866 | 0.00045245 | DOWN |
| ENSSSCG00000036307 | OAF | -1.627223111 | 8.40876E-06 | DOWN |
| ENSSSCG00000013318 | RCN1 | -1.627741216 | 0.000612812 | DOWN |
| ENSSSCG00000039854 | NA | -1.627929225 | 2.51514E-05 | DOWN |
| ENSSSCG00000012531 | NA | -1.632425989 | 0.04435041 | DOWN |
| ENSSSCG00000029029 | ZNF713 | -1.63315517 | 5.63254E-07 | DOWN |
| ENSSSCG00000002469 | OTUB2 | -1.638159946 | 0.009387301 | DOWN |
| ENSSSCG00000013256 | ARHGAP1 | -1.63888255 | 1.4348E-16 | DOWN |
| ENSSSCG00000028612 | PTPRS | -1.643486594 | 0.000892413 | DOWN |
| ENSSSCG00000033564 | NA | -1.643618843 | 0.005207415 | DOWN |
| ENSSSCG00000033854 | LGALS1 | -1.644138903 | 0.024693675 | DOWN |
| ENSSSCG00000003876 | CDKN2C | -1.64625655 | 2.06977E-07 | DOWN |
| ENSSSCG00000033546 | UBXN11 | -1.646971815 | 0.000214416 | DOWN |
| ENSSSCG00000001657 | CUL7 | -1.648256135 | 3.19558E-06 | DOWN |
| ENSSSCG00000035169 | HOTAIRM1_3 | -1.64879214 | 0.00091572 | DOWN |
| ENSSSCG00000038492 | FAM109B | -1.64926433 | 9.30695E-05 | DOWN |
| ENSSSCG00000007356 | PLCG1 | -1.649507408 | 7.23087E-05 | DOWN |
| ENSSSCG00000003326 | NA | -1.650894052 | 3.18728E-05 | DOWN |
| ENSSSCG00000012504 | NAP1L3 | -1.651285489 | 1.44491E-06 | DOWN |
| ENSSSCG00000005087 | SIX1 | -1.653932835 | 7.59356E-09 | DOWN |
| ENSSSCG00000015607 | HHAT | -1.657651854 | 0.00013931 | DOWN |
| ENSSSCG00000004931 | IGDCC4 | -1.662903181 | 0.001468062 | DOWN |
| ENSSSCG00000010768 | CFAP46 | -1.664376643 | 0.047678603 | DOWN |
| ENSSSCG00000012561 | FRMPD3 | -1.665394588 | 0.003809007 | DOWN |
| ENSSSCG00000023105 | NET1 | -1.667132325 | 0.001589253 | DOWN |
| ENSSSCG00000024973 | NA | -1.66827619 | 0.039370802 | DOWN |
| ENSSSCG00000014960 | AMOTL1 | -1.669513855 | 8.93119E-07 | DOWN |
| ENSSSCG00000035755 | SHISA8 | -1.673850447 | 0.026325523 | DOWN |
| ENSSSCG00000032536 | B3GNT8 | -1.677240037 | 0.002081108 | DOWN |
| ENSSSCG00000032942 | NA | -1.679119632 | 2.45016E-07 | DOWN |
| ENSSSCG00000036080 | NA | -1.686868533 | 0.020549259 | DOWN |
| ENSSSCG00000037087 | PRRT4 | -1.688375949 | 0.041570059 | DOWN |
| ENSSSCG00000038643 | KLF11 | -1.688435677 | 1.89608E-05 | DOWN |
| ENSSSCG00000008555 | CGREF1 | -1.688767747 | 0.002578382 | DOWN |
| ENSSSCG00000015617 | G0S2 | -1.696209041 | 0.028063864 | DOWN |
| ENSSSCG00000010122 | CDC45 | -1.696947327 | 7.22591E-05 | DOWN |
| ENSSSCG00000024481 | NA | -1.700059647 | 1.31816E-06 | DOWN |
| ENSSSCG00000037416 | CLIC5 | -1.702006352 | 0.033931758 | DOWN |
| ENSSSCG00000017131 | FN3K | -1.705129732 | 0.000657862 | DOWN |
| ENSSSCG00000039802 | FBXL2 | -1.707339183 | 8.95425E-05 | DOWN |
| ENSSSCG00000036977 | NA | -1.709973954 | 0.001156186 | DOWN |
| ENSSSCG00000027528 | TMEM107 | -1.710143698 | 7.60244E-10 | DOWN |
| ENSSSCG00000015368 | HDAC9 | -1.712035747 | 1.5438E-05 | DOWN |
| ENSSSCG00000010627 | PDCD4 | -1.712697973 | 0.00016525 | DOWN |
| ENSSSCG00000040603 | SGTB | -1.713335344 | 0.00024368 | DOWN |
| ENSSSCG00000006725 | TBX15 | -1.719852401 | 4.79099E-10 | DOWN |
| ENSSSCG00000017101 | ADCY2 | -1.722612724 | 0.006075006 | DOWN |
| ENSSSCG00000022429 | KAZALD1 | -1.723817138 | 0.002903103 | DOWN |
| ENSSSCG00000013332 | KIF18A | -1.725188517 | 0.001778945 | DOWN |
| ENSSSCG00000017428 | JUP | -1.725855736 | 5.05424E-06 | DOWN |
| ENSSSCG00000027144 | LMNTD1 | -1.727325473 | 5.45633E-07 | DOWN |
| ENSSSCG00000037450 | SSC5D | -1.731014101 | 1.69077E-05 | DOWN |
| ENSSSCG00000022247 | PROSER2 | -1.731120093 | 8.08423E-05 | DOWN |
| ENSSSCG00000016085 | NA | -1.732413332 | 3.2234E-05 | DOWN |
| ENSSSCG00000025244 | HTR6 | -1.733143462 | 0.01672271 | DOWN |
| ENSSSCG00000027434 | OLFM2 | -1.738831179 | 0.013120255 | DOWN |
| ENSSSCG00000013382 | PLEKHA7 | -1.739625621 | 0.008735136 | DOWN |
| ENSSSCG00000014581 | TUB | -1.741666541 | 2.97557E-07 | DOWN |
| ENSSSCG00000025856 | TMEM106A | -1.741672295 | 1.20457E-05 | DOWN |
| ENSSSCG00000031378 | KBTBD6 | -1.744764505 | 8.52657E-10 | DOWN |
| ENSSSCG00000000699 | LPAR5 | -1.745247768 | 0.044182541 | DOWN |
| ENSSSCG00000012730 | AFF2 | -1.748594368 | 0.013214268 | DOWN |
| ENSSSCG00000002786 | ELMO3 | -1.750987793 | 0.027729119 | DOWN |
| ENSSSCG00000005385 | NR4A3 | -1.751602961 | 0.014942375 | DOWN |
| ENSSSCG00000040461 | NA | -1.759913202 | 0.000171522 | DOWN |
| ENSSSCG00000015396 | SEMA3D | -1.760116558 | 0.002708936 | DOWN |
| ENSSSCG00000031798 | DNAH3 | -1.761514413 | 0.002708438 | DOWN |
| ENSSSCG00000035738 | RPL36AL | -1.76244665 | 0.000374272 | DOWN |
| ENSSSCG00000036454 | TMEM220 | -1.76748818 | 5.16444E-09 | DOWN |
| ENSSSCG00000035243 | RAB27B | -1.773428551 | 9.45088E-05 | DOWN |
| ENSSSCG00000012557 | NA | -1.773894571 | 0.010025795 | DOWN |
| ENSSSCG00000002768 | CENPT | -1.773962607 | 0.043746853 | DOWN |
| ENSSSCG00000029656 | NDP | -1.775768064 | 0.00127265 | DOWN |
| ENSSSCG00000002510 | CYP46A1 | -1.777894444 | 2.88152E-05 | DOWN |
| ENSSSCG00000032909 | CDCA8 | -1.778255901 | 0.002714271 | DOWN |
| ENSSSCG00000001910 | ISLR | -1.779379184 | 0.000219589 | DOWN |
| ENSSSCG00000017082 | SPARC | -1.779771327 | 0.000313367 | DOWN |
| ENSSSCG00000011065 | MASTL | -1.781428599 | 0.01719285 | DOWN |
| ENSSSCG00000001434 | EGFL8 | -1.785144005 | 0.001110825 | DOWN |
| ENSSSCG00000021880 | MXRA8 | -1.787179134 | 7.54871E-07 | DOWN |
| ENSSSCG00000006331 | PBX1 | -1.787960591 | 2.78704E-11 | DOWN |
| ENSSSCG00000003949 | CDC20 | -1.78850826 | 0.001137699 | DOWN |
| ENSSSCG00000022029 | RAP1GAP | -1.789999922 | 0.004658423 | DOWN |
| ENSSSCG00000012448 | ITM2A | -1.792339889 | 0.011008835 | DOWN |
| ENSSSCG00000014892 | USP35 | -1.793414504 | 0.000851822 | DOWN |
| ENSSSCG00000015774 | NA | -1.802644855 | 0.002355447 | DOWN |
| ENSSSCG00000000371 | PMEL | -1.803703827 | 0.047741706 | DOWN |
| ENSSSCG00000001469 | SLA-DMB | -1.803772002 | 0.027775515 | DOWN |
| ENSSSCG00000020720 | MRVI1 | -1.804486097 | 0.014006651 | DOWN |
| ENSSSCG00000006344 | NOS1AP | -1.806632243 | 0.000732859 | DOWN |
| ENSSSCG00000030005 | LGALSL | -1.80708849 | 4.73661E-16 | DOWN |
| ENSSSCG00000015175 | VWA5A | -1.809547218 | 2.30512E-17 | DOWN |
| ENSSSCG00000030469 | NA | -1.81117437 | 0.017291081 | DOWN |
| ENSSSCG00000012371 | AR | -1.817916342 | 0.025212795 | DOWN |
| ENSSSCG00000014041 | MXD3 | -1.819214101 | 0.008190604 | DOWN |
| ENSSSCG00000016658 | ANLN | -1.820181281 | 0.000794704 | DOWN |
| ENSSSCG00000003715 | NA | -1.820272648 | 1.13877E-08 | DOWN |
| ENSSSCG00000004013 | SMOC2 | -1.828275637 | 0.002753336 | DOWN |
| ENSSSCG00000025423 | KCNK5 | -1.832368011 | 0.003891436 | DOWN |
| ENSSSCG00000031731 | NA | -1.835422475 | 0.004382315 | DOWN |
| ENSSSCG00000010701 | BTBD16 | -1.838936218 | 0.004498675 | DOWN |
| ENSSSCG00000010322 | ZNF503 | -1.842030102 | 6.71919E-08 | DOWN |
| ENSSSCG00000012699 | NA | -1.843800234 | 8.30208E-13 | DOWN |
| ENSSSCG00000039587 | NA | -1.846005611 | 5.23096E-06 | DOWN |
| ENSSSCG00000037674 | NA | -1.846922206 | 3.95575E-06 | DOWN |
| ENSSSCG00000037098 | C19orf81 | -1.847021122 | 0.011362011 | DOWN |
| ENSSSCG00000037835 | TRIM7 | -1.847250088 | 0.000129259 | DOWN |
| ENSSSCG00000016794 | MYO10 | -1.851914237 | 2.94069E-09 | DOWN |
| ENSSSCG00000010064 | NA | -1.852470979 | 3.86389E-06 | DOWN |
| ENSSSCG00000017046 | EBF1 | -1.852865595 | 3.35153E-06 | DOWN |
| ENSSSCG00000010278 | NA | -1.85376642 | 0.01527753 | DOWN |
| ENSSSCG00000010698 | FGFR2 | -1.855456151 | 0.005209425 | DOWN |
| ENSSSCG00000029151 | RASIP1 | -1.864160308 | 0.017287133 | DOWN |
| ENSSSCG00000023526 | RAPGEF3 | -1.867329179 | 5.46014E-06 | DOWN |
| ENSSSCG00000006690 | NUDT17 | -1.867529597 | 0.002139071 | DOWN |
| ENSSSCG00000001914 | LOXL1 | -1.867997696 | 0.004234123 | DOWN |
| ENSSSCG00000038514 | DDAH2 | -1.870453841 | 9.44908E-08 | DOWN |
| ENSSSCG00000026326 | CCNF | -1.872911044 | 0.009876769 | DOWN |
| ENSSSCG00000037120 | TK1 | -1.875634745 | 0.005189964 | DOWN |
| ENSSSCG00000023273 | SH3YL1 | -1.875758028 | 9.66115E-05 | DOWN |
| ENSSSCG00000006582 | S100A14 | -1.877139274 | 2.89118E-06 | DOWN |
| ENSSSCG00000005992 | SHAS2 | -1.879413302 | 0.002754113 | DOWN |
| ENSSSCG00000007991 | WDR90 | -1.883386208 | 1.29194E-05 | DOWN |
| ENSSSCG00000031053 | S100A1 | -1.886331959 | 0.002908665 | DOWN |
| ENSSSCG00000026427 | RORC | -1.886591454 | 0.001073865 | DOWN |
| ENSSSCG00000006153 | FABP5 | -1.887939222 | 0.049502348 | DOWN |
| ENSSSCG00000036305 | NA | -1.888917781 | 7.68184E-05 | DOWN |
| ENSSSCG00000009122 | ARSJ | -1.89008008 | 1.79561E-09 | DOWN |
| ENSSSCG00000017068 | FAXDC2 | -1.892403151 | 0.001139065 | DOWN |
| ENSSSCG00000033444 | SPC24 | -1.893942416 | 0.00819665 | DOWN |
| ENSSSCG00000016983 | STC2 | -1.894605305 | 5.4191E-06 | DOWN |
| ENSSSCG00000012510 | ARMCX2 | -1.899564472 | 0.000687475 | DOWN |
| ENSSSCG00000016784 | ANKH | -1.901499679 | 4.73661E-16 | DOWN |
| ENSSSCG00000003572 | SYTL1 | -1.905182329 | 0.001614694 | DOWN |
| ENSSSCG00000037766 | NR3C2 | -1.907784607 | 1.82788E-06 | DOWN |
| ENSSSCG00000008747 | NCAPG | -1.911330788 | 0.005279058 | DOWN |
| ENSSSCG00000009720 | DDX60 | -1.914996561 | 0.000891133 | DOWN |
| ENSSSCG00000033337 | ARHGDIB | -1.915042119 | 0.005834758 | DOWN |
| ENSSSCG00000017367 | MPP2 | -1.91840282 | 0.000912334 | DOWN |
| ENSSSCG00000015700 | TMEM163 | -1.920548891 | 0.013449756 | DOWN |
| ENSSSCG00000004675 | DUOX1 | -1.921721796 | 0.000469571 | DOWN |
| ENSSSCG00000033993 | PLCXD3 | -1.927814098 | 0.004633573 | DOWN |
| ENSSSCG00000008913 | IGFBP7 | -1.932476235 | 1.20306E-06 | DOWN |
| ENSSSCG00000004554 | PCLAF | -1.934818421 | 0.001814489 | DOWN |
| ENSSSCG00000013909 | CRLF1 | -1.935998304 | 1.23461E-06 | DOWN |
| ENSSSCG00000005657 | PKN3 | -1.93620455 | 0.000558669 | DOWN |
| ENSSSCG00000006688 | ANKRD35 | -1.938496413 | 1.41593E-07 | DOWN |
| ENSSSCG00000004489 | EEF1A1 | -1.938788198 | 2.0176E-11 | DOWN |
| ENSSSCG00000018538 | SNORA52 | -1.940564466 | 0.005133117 | DOWN |
| ENSSSCG00000039182 | C11orf96 | -1.944586173 | 0.001037592 | DOWN |
| ENSSSCG00000015399 | SEMA3E | -1.945203899 | 0.00421257 | DOWN |
| ENSSSCG00000014957 | C11orf97 | -1.94586032 | 0.02809466 | DOWN |
| ENSSSCG00000033001 | FZD8 | -1.946956829 | 4.19891E-13 | DOWN |
| ENSSSCG00000030827 | FGFR3 | -1.950376559 | 0.000146057 | DOWN |
| ENSSSCG00000002916 | APLP1 | -1.951680374 | 3.79735E-05 | DOWN |
| ENSSSCG00000037413 | NA | -1.956383322 | 8.9351E-08 | DOWN |
| ENSSSCG00000000893 | AMDHD1 | -1.958008463 | 0.031302217 | DOWN |
| ENSSSCG00000017607 | TMEM100 | -1.958785516 | 0.010371452 | DOWN |
| ENSSSCG00000016976 | ZNF366 | -1.959664278 | 0.013178327 | DOWN |
| ENSSSCG00000013440 | ATP8B3 | -1.960024479 | 0.000348387 | DOWN |
| ENSSSCG00000038038 | NA | -1.960167741 | 0.01529324 | DOWN |
| ENSSSCG00000004422 | WISP3 | -1.960990002 | 0.044131258 | DOWN |
| ENSSSCG00000039045 | SLC26A2 | -1.964823802 | 1.20457E-05 | DOWN |
| ENSSSCG00000015196 | SIAE | -1.964912743 | 3.89247E-14 | DOWN |
| ENSSSCG00000028492 | C4orf46 | -1.965186251 | 0.007509569 | DOWN |
| ENSSSCG00000002306 | GALNT16 | -1.965418206 | 5.73452E-06 | DOWN |
| ENSSSCG00000005278 | NA | -1.966724005 | 0.021001614 | DOWN |
| ENSSSCG00000021479 | RGS9BP | -1.970545419 | 0.041461425 | DOWN |
| ENSSSCG00000031787 | NA | -1.976157674 | 0.000537961 | DOWN |
| ENSSSCG00000002490 | GSC | -1.976475761 | 0.016097595 | DOWN |
| ENSSSCG00000000148 | NA | -1.979906581 | 4.00508E-06 | DOWN |
| ENSSSCG00000022980 | TBX4 | -1.988039485 | 0.006325535 | DOWN |
| ENSSSCG00000022128 | MXI1 | -1.988383059 | 9.54211E-11 | DOWN |
| ENSSSCG00000016618 | CPED1 | -1.991975076 | 6.31508E-09 | DOWN |
| ENSSSCG00000028135 | NA | -1.994039397 | 0.000639133 | DOWN |
| ENSSSCG00000008125 | NCAPH | -1.995266288 | 0.005047259 | DOWN |
| ENSSSCG00000010600 | CALHM2 | -1.995849257 | 6.39001E-05 | DOWN |
| ENSSSCG00000028924 | AURKB | -1.999558951 | 0.011853599 | DOWN |
| ENSSSCG00000040486 | BIRC5 | -2.002863785 | 7.03086E-05 | DOWN |
| ENSSSCG00000012832 | MXRA5 | -2.008080406 | 0.008226148 | DOWN |
| ENSSSCG00000004209 | PTPRK | -2.010365565 | 1.2503E-07 | DOWN |
| ENSSSCG00000033780 | B4GALNT4 | -2.013797995 | 0.019220974 | DOWN |
| ENSSSCG00000006923 | GBP2 | -2.01500402 | 0.001344715 | DOWN |
| ENSSSCG00000014943 | DEUP1 | -2.019986989 | 0.000561766 | DOWN |
| ENSSSCG00000013639 | SLC44A2 | -2.020475583 | 6.02664E-17 | DOWN |
| ENSSSCG00000010017 | SMTN | -2.021513892 | 1.30052E-05 | DOWN |
| ENSSSCG00000028144 | EPHX3 | -2.023031148 | 0.001917062 | DOWN |
| ENSSSCG00000017511 | PLXDC1 | -2.024614773 | 0.000650644 | DOWN |
| ENSSSCG00000016313 | HJURP | -2.031679783 | 0.019014493 | DOWN |
| ENSSSCG00000014219 | CDO1 | -2.033327259 | 1.31957E-05 | DOWN |
| ENSSSCG00000000455 | LRIG3 | -2.03365374 | 1.30229E-06 | DOWN |
| ENSSSCG00000011928 | CCDC80 | -2.034615594 | 0.000425711 | DOWN |
| ENSSSCG00000000739 | FOXM1 | -2.035859167 | 0.002039161 | DOWN |
| ENSSSCG00000036060 | RRAD | -2.040042466 | 0.001190458 | DOWN |
| ENSSSCG00000039838 | RGS10 | -2.044501731 | 0.000702446 | DOWN |
| ENSSSCG00000036096 | NA | -2.047008355 | 0.003990575 | DOWN |
| ENSSSCG00000015353 | SCIN | -2.048333148 | 0.024648515 | DOWN |
| ENSSSCG00000024043 | ADAMTS2 | -2.049914032 | 7.45096E-07 | DOWN |
| ENSSSCG00000006101 | NA | -2.052294725 | 0.02522331 | DOWN |
| ENSSSCG00000004961 | ITGA11 | -2.052329702 | 0.004626099 | DOWN |
| ENSSSCG00000034266 | NA | -2.053271574 | 0.022329939 | DOWN |
| ENSSSCG00000017364 | C17orf53 | -2.054206567 | 0.002147377 | DOWN |
| ENSSSCG00000012375 | DLG3 | -2.057620548 | 1.78827E-08 | DOWN |
| ENSSSCG00000026868 | LRRC15 | -2.059170717 | 0.011364631 | DOWN |
| ENSSSCG00000007072 | SPTLC3 | -2.060335588 | 5.9649E-05 | DOWN |
| ENSSSCG00000006524 | THBS3 | -2.063559626 | 1.07585E-06 | DOWN |
| ENSSSCG00000015862 | LIMS2 | -2.065551366 | 0.014760959 | DOWN |
| ENSSSCG00000003081 | CEACAM16 | -2.070380814 | 0.000153316 | DOWN |
| ENSSSCG00000015120 | USP2 | -2.073286676 | 7.15787E-14 | DOWN |
| ENSSSCG00000022322 | BCL2L11 | -2.074274409 | 6.71706E-08 | DOWN |
| ENSSSCG00000008613 | GEN1 | -2.075955255 | 0.038845334 | DOWN |
| ENSSSCG00000033648 | NA | -2.081051438 | 8.77886E-06 | DOWN |
| ENSSSCG00000025590 | NA | -2.084586769 | 0.019599808 | DOWN |
| ENSSSCG00000040706 | DBN1 | -2.085155492 | 4.63133E-06 | DOWN |
| ENSSSCG00000039332 | SEC16B | -2.095144241 | 1.99843E-08 | DOWN |
| ENSSSCG00000014436 | ARHGEF37 | -2.096914731 | 0.001917576 | DOWN |
| ENSSSCG00000003687 | EPB41L3 | -2.098231099 | 0.00158581 | DOWN |
| ENSSSCG00000004891 | SERPINB7 | -2.09939261 | 0.000290699 | DOWN |
| ENSSSCG00000000002 | GTSE1 | -2.101438873 | 0.008275857 | DOWN |
| ENSSSCG00000015271 | PRELP | -2.101974935 | 9.44694E-05 | DOWN |
| ENSSSCG00000012519 | GPRASP1 | -2.104561133 | 2.03468E-10 | DOWN |
| ENSSSCG00000011208 | ZNF385D | -2.108318369 | 1.342E-07 | DOWN |
| ENSSSCG00000001793 | ADAMTSL3 | -2.120929332 | 4.65043E-12 | DOWN |
| ENSSSCG00000016617 | WNT16 | -2.123041769 | 0.036884709 | DOWN |
| ENSSSCG00000030337 | NYAP1 | -2.12359575 | 2.12934E-06 | DOWN |
| ENSSSCG00000004466 | TTK | -2.124918903 | 0.022018834 | DOWN |
| ENSSSCG00000014959 | PIWIL4 | -2.128513298 | 0.003707162 | DOWN |
| ENSSSCG00000000265 | ESPL1 | -2.129233399 | 0.003626941 | DOWN |
| ENSSSCG00000010109 | SCARF2 | -2.130800092 | 5.14712E-05 | DOWN |
| ENSSSCG00000036114 | NA | -2.130997222 | 2.13736E-05 | DOWN |
| ENSSSCG00000029811 | PLCL2 | -2.132980223 | 1.20729E-14 | DOWN |
| ENSSSCG00000012032 | NA | -2.134546001 | 0.026325523 | DOWN |
| ENSSSCG00000005056 | DLGAP5 | -2.139898696 | 0.007244089 | DOWN |
| ENSSSCG00000014047 | FGFR4 | -2.147356865 | 0.011594075 | DOWN |
| ENSSSCG00000011014 | BAMBI | -2.14893485 | 1.32827E-05 | DOWN |
| ENSSSCG00000037054 | NA | -2.150063936 | 0.0450136 | DOWN |
| ENSSSCG00000024290 | NA | -2.150370203 | 0.035275075 | DOWN |
| ENSSSCG00000004136 | AIG1 | -2.154166778 | 3.07161E-18 | DOWN |
| ENSSSCG00000036748 | NA | -2.159259629 | 0.000730548 | DOWN |
| ENSSSCG00000006174 | JPH1 | -2.160980732 | 0.027445785 | DOWN |
| ENSSSCG00000026516 | EPHB3 | -2.162500641 | 3.93708E-09 | DOWN |
| ENSSSCG00000009378 | CKAP2 | -2.1659567 | 5.63646E-05 | DOWN |
| ENSSSCG00000003909 | NA | -2.166182558 | 2.69947E-05 | DOWN |
| ENSSSCG00000022953 | PTER | -2.174271909 | 4.06338E-05 | DOWN |
| ENSSSCG00000011239 | NA | -2.174959161 | 3.19558E-06 | DOWN |
| ENSSSCG00000031244 | GAP43 | -2.176504978 | 7.39282E-06 | DOWN |
| ENSSSCG00000039107 | NA | -2.181122832 | 0.001211481 | DOWN |
| ENSSSCG00000037015 | SESN3 | -2.181597942 | 1.44656E-07 | DOWN |
| ENSSSCG00000016589 | LRRC4 | -2.182588399 | 0.000214416 | DOWN |
| ENSSSCG00000003479 | MFAP2 | -2.187806943 | 1.07966E-17 | DOWN |
| ENSSSCG00000030361 | PRKCZ | -2.19257664 | 0.033158594 | DOWN |
| ENSSSCG00000010959 | NTRK2 | -2.192911562 | 0.001222608 | DOWN |
| ENSSSCG00000012512 | ZMAT1 | -2.193168721 | 0.036481952 | DOWN |
| ENSSSCG00000010603 | NEURL1 | -2.199780714 | 0.035992707 | DOWN |
| ENSSSCG00000000734 | NA | -2.202220157 | 0.001481025 | DOWN |
| ENSSSCG00000038811 | NA | -2.203906541 | 1.51131E-05 | DOWN |
| ENSSSCG00000012528 | BEX3 | -2.209147318 | 1.11965E-07 | DOWN |
| ENSSSCG00000036007 | MFAP4 | -2.214933888 | 6.38247E-07 | DOWN |
| ENSSSCG00000032383 | NA | -2.220808585 | 0.032113034 | DOWN |
| ENSSSCG00000006933 | CLCA1 | -2.221931374 | 0.003954329 | DOWN |
| ENSSSCG00000003697 | NDC80 | -2.225621027 | 0.007762757 | DOWN |
| ENSSSCG00000015828 | ZNF703 | -2.229068573 | 7.80754E-13 | DOWN |
| ENSSSCG00000033443 | DEPDC1 | -2.229494397 | 0.041527061 | DOWN |
| ENSSSCG00000033805 | NA | -2.238580605 | 0.009665768 | DOWN |
| ENSSSCG00000012377 | NA | -2.239806704 | 0.00103486 | DOWN |
| ENSSSCG00000013401 | DKK3 | -2.249316109 | 0.0115922 | DOWN |
| ENSSSCG00000000707 | SCNN1A | -2.254141813 | 0.002322369 | DOWN |
| ENSSSCG00000009448 | DIAPH3 | -2.254164994 | 0.001299838 | DOWN |
| ENSSSCG00000039009 | NA | -2.257337929 | 0.004653095 | DOWN |
| ENSSSCG00000005314 | ARHGEF39 | -2.285033851 | 0.010437601 | DOWN |
| ENSSSCG00000003931 | KIF2C | -2.285143792 | 0.000373669 | DOWN |
| ENSSSCG00000008948 | ALB | -2.28669467 | 0.036845467 | DOWN |
| ENSSSCG00000004588 | CCNB2 | -2.293753138 | 0.000544093 | DOWN |
| ENSSSCG00000015006 | NA | -2.294191996 | 0.030878762 | DOWN |
| ENSSSCG00000005494 | TNC | -2.296947872 | 0.000104583 | DOWN |
| ENSSSCG00000003600 | TINAGL1 | -2.29961014 | 2.43348E-10 | DOWN |
| ENSSSCG00000038724 | C3orf70 | -2.304767367 | 2.55119E-08 | DOWN |
| ENSSSCG00000012257 | MAOA | -2.304915731 | 1.99715E-18 | DOWN |
| ENSSSCG00000004782 | BUB1B | -2.30563401 | 0.001737155 | DOWN |
| ENSSSCG00000017342 | KIF18B | -2.306930529 | 0.015580042 | DOWN |
| ENSSSCG00000016199 | CYP27A1 | -2.311474785 | 0.000267566 | DOWN |
| ENSSSCG00000005094 | TMEM30B | -2.315452168 | 1.0535E-10 | DOWN |
| ENSSSCG00000013934 | CILP2 | -2.317009735 | 1.08248E-06 | DOWN |
| ENSSSCG00000003633 | TEKT2 | -2.318805227 | 1.00695E-07 | DOWN |
| ENSSSCG00000015522 | ANGPTL1 | -2.321433347 | 0.044715778 | DOWN |
| ENSSSCG00000014232 | LOX | -2.329227002 | 1.83491E-08 | DOWN |
| ENSSSCG00000038491 | MEX3B | -2.329911162 | 4.59769E-06 | DOWN |
| ENSSSCG00000020846 | SCARA3 | -2.333959087 | 2.96185E-05 | DOWN |
| ENSSSCG00000003351 | MMP23B | -2.335982301 | 0.013390844 | DOWN |
| ENSSSCG00000013772 | ASF1B | -2.336550693 | 0.000788353 | DOWN |
| ENSSSCG00000016006 | NA | -2.338000589 | 0.00288764 | DOWN |
| ENSSSCG00000010429 | PRKG1 | -2.338123987 | 0.006412488 | DOWN |
| ENSSSCG00000003148 | DBP | -2.33921356 | 2.29432E-11 | DOWN |
| ENSSSCG00000017789 | ABHD15 | -2.345717254 | 1.65433E-05 | DOWN |
| ENSSSCG00000016717 | MPP6 | -2.346533387 | 1.69611E-08 | DOWN |
| ENSSSCG00000022490 | GPR83 | -2.349472874 | 0.045722492 | DOWN |
| ENSSSCG00000009351 | MAB21L1 | -2.349768553 | 0.009529002 | DOWN |
| ENSSSCG00000031188 | C1orf185 | -2.349994799 | 0.023657081 | DOWN |
| ENSSSCG00000035284 | BMF | -2.355568783 | 2.61223E-08 | DOWN |
| ENSSSCG00000039568 | SNAI2 | -2.357306263 | 7.77675E-07 | DOWN |
| ENSSSCG00000008230 | ATOH8 | -2.357943348 | 6.10726E-07 | DOWN |
| ENSSSCG00000009867 | TBX5 | -2.359779691 | 1.13671E-07 | DOWN |
| ENSSSCG00000007365 | L3MBTL1 | -2.360913345 | 0.038419173 | DOWN |
| ENSSSCG00000036814 | CLEC11A | -2.363300745 | 0.000141634 | DOWN |
| ENSSSCG00000035223 | SYNM | -2.364802602 | 1.47802E-16 | DOWN |
| ENSSSCG00000040638 | DIO2 | -2.365359987 | 0.001059203 | DOWN |
| ENSSSCG00000017022 | HMMR | -2.369527076 | 0.001714537 | DOWN |
| ENSSSCG00000024621 | KAT2B | -2.370341014 | 6.9402E-19 | DOWN |
| ENSSSCG00000021997 | ALS2CL | -2.37305475 | 0.000105771 | DOWN |
| ENSSSCG00000023261 | GDF5 | -2.37667697 | 0.006662369 | DOWN |
| ENSSSCG00000006281 | METTL11B | -2.380949357 | 0.017267976 | DOWN |
| ENSSSCG00000027466 | PCOLCE | -2.381433372 | 1.44305E-09 | DOWN |
| ENSSSCG00000035908 | NA | -2.383664265 | 0.034213219 | DOWN |
| ENSSSCG00000016521 | DGKI | -2.387370428 | 8.07544E-06 | DOWN |
| ENSSSCG00000012286 | ZNF630 | -2.389724453 | 0.000208086 | DOWN |
| ENSSSCG00000006857 | COL11A1 | -2.391261456 | 3.77465E-05 | DOWN |
| ENSSSCG00000001510 | KIFC1 | -2.393667751 | 8.03983E-06 | DOWN |
| ENSSSCG00000014575 | NA | -2.39658918 | 0.00466367 | DOWN |
| ENSSSCG00000014314 | NA | -2.398432782 | 1.37828E-05 | DOWN |
| ENSSSCG00000039370 | NA | -2.403180178 | 0.036214802 | DOWN |
| ENSSSCG00000009283 | TNFRSF19 | -2.411617706 | 4.1993E-05 | DOWN |
| ENSSSCG00000017754 | NA | -2.41707336 | 0.00926032 | DOWN |
| ENSSSCG00000017032 | NA | -2.417505161 | 0.001295898 | DOWN |
| ENSSSCG00000039558 | NA | -2.418085095 | 0.024592232 | DOWN |
| ENSSSCG00000023915 | SLC2A4 | -2.429606411 | 0.016512624 | DOWN |
| ENSSSCG00000038013 | DIRAS1 | -2.441302306 | 0.034037739 | DOWN |
| ENSSSCG00000000910 | CRADD | -2.442732646 | 2.73784E-24 | DOWN |
| ENSSSCG00000007073 | ISM1 | -2.445121698 | 6.1885E-06 | DOWN |
| ENSSSCG00000012774 | DUSP9 | -2.446616329 | 0.037942118 | DOWN |
| ENSSSCG00000013625 | CCDC151 | -2.448094742 | 0.029569425 | DOWN |
| ENSSSCG00000004939 | MEGF11 | -2.450897281 | 0.001975328 | DOWN |
| ENSSSCG00000001081 | SOX4 | -2.452848305 | 2.69947E-05 | DOWN |
| ENSSSCG00000000683 | CDCA3 | -2.456737601 | 0.000126883 | DOWN |
| ENSSSCG00000038149 | KCNE4 | -2.458990801 | 5.56194E-15 | DOWN |
| ENSSSCG00000026404 | SERTAD4 | -2.461672441 | 1.31816E-06 | DOWN |
| ENSSSCG00000032660 | TMEM35B | -2.463910608 | 0.000198477 | DOWN |
| ENSSSCG00000011831 | APOD | -2.46695313 | 1.96815E-05 | DOWN |
| ENSSSCG00000008029 | BAIAP3 | -2.467773242 | 0.043475034 | DOWN |
| ENSSSCG00000010087 | YDJC | -2.472067154 | 0.001172914 | DOWN |
| ENSSSCG00000003875 | TTC39A | -2.475888859 | 0.008012961 | DOWN |
| ENSSSCG00000036796 | VWA7 | -2.476235303 | 0.033134952 | DOWN |
| ENSSSCG00000011511 | FRMD4B | -2.477321271 | 3.4571E-08 | DOWN |
| ENSSSCG00000009181 | C4orf17 | -2.480057863 | 0.045111966 | DOWN |
| ENSSSCG00000029838 | FZD2 | -2.481412901 | 0.014392242 | DOWN |
| ENSSSCG00000038545 | C10orf105 | -2.481516169 | 0.029610282 | DOWN |
| ENSSSCG00000011959 | ABI3BP | -2.482213752 | 4.00129E-08 | DOWN |
| ENSSSCG00000021683 | NA | -2.485728905 | 9.47809E-11 | DOWN |
| ENSSSCG00000037918 | ADIRF | -2.487927754 | 0.003055464 | DOWN |
| ENSSSCG00000031300 | NA | -2.489405165 | 0.019376843 | DOWN |
| ENSSSCG00000004573 | NA | -2.490764214 | 5.86196E-05 | DOWN |
| ENSSSCG00000013263 | CREB3L1 | -2.495153169 | 1.93236E-11 | DOWN |
| ENSSSCG00000023498 | HSPB6 | -2.49589991 | 6.87933E-28 | DOWN |
| ENSSSCG00000032330 | THY1 | -2.49744415 | 6.3492E-09 | DOWN |
| ENSSSCG00000006572 | NPR1 | -2.500444373 | 0.007415369 | DOWN |
| ENSSSCG00000038579 | DKKL1 | -2.501607083 | 1.08967E-10 | DOWN |
| ENSSSCG00000023090 | BEST1 | -2.503037941 | 0.001304175 | DOWN |
| ENSSSCG00000006132 | MMP16 | -2.512369072 | 8.81067E-10 | DOWN |
| ENSSSCG00000012034 | TIAM1 | -2.520965379 | 0.048541492 | DOWN |
| ENSSSCG00000025826 | BOC | -2.521434816 | 3.12547E-09 | DOWN |
| ENSSSCG00000032525 | NA | -2.529484933 | 0.009220737 | DOWN |
| ENSSSCG00000037892 | YPEL4 | -2.531817447 | 0.00370591 | DOWN |
| ENSSSCG00000003022 | TMEM145 | -2.536971569 | 9.46715E-13 | DOWN |
| ENSSSCG00000000478 | GRIP1 | -2.538209765 | 0.017445431 | DOWN |
| ENSSSCG00000027970 | NA | -2.551071323 | 2.5278E-06 | DOWN |
| ENSSSCG00000027854 | HSD17B6 | -2.552261559 | 0.005852367 | DOWN |
| ENSSSCG00000015604 | NEK2 | -2.55262974 | 0.000278335 | DOWN |
| ENSSSCG00000037206 | NA | -2.555995622 | 0.011659088 | DOWN |
| ENSSSCG00000008314 | NA | -2.56607435 | 0.005510722 | DOWN |
| ENSSSCG00000014088 | IQGAP2 | -2.566435195 | 0.00209878 | DOWN |
| ENSSSCG00000000591 | PIK3C2G | -2.569010639 | 0.033171793 | DOWN |
| ENSSSCG00000040228 | NA | -2.570243772 | 3.30171E-06 | DOWN |
| ENSSSCG00000002315 | SLC8A3 | -2.575344056 | 4.7342E-07 | DOWN |
| ENSSSCG00000033314 | DLX6 | -2.575767057 | 4.25492E-05 | DOWN |
| ENSSSCG00000003371 | GPR153 | -2.583931214 | 2.12233E-10 | DOWN |
| ENSSSCG00000011471 | FLNB | -2.584487789 | 6.588E-05 | DOWN |
| ENSSSCG00000037106 | C2orf40 | -2.586884711 | 0.006455741 | DOWN |
| ENSSSCG00000040397 | COLEC12 | -2.587386606 | 0.000166691 | DOWN |
| ENSSSCG00000011664 | RBP1 | -2.594598661 | 0.0115922 | DOWN |
| ENSSSCG00000038290 | RNF182 | -2.602847229 | 6.52654E-05 | DOWN |
| ENSSSCG00000004663 | SEMA6D | -2.609461392 | 1.56332E-08 | DOWN |
| ENSSSCG00000033235 | NA | -2.609919631 | 0.007197887 | DOWN |
| ENSSSCG00000004902 | RNF152 | -2.609964461 | 8.07138E-15 | DOWN |
| ENSSSCG00000015937 | KLHL23 | -2.612060362 | 0.020011037 | DOWN |
| ENSSSCG00000017296 | ACE | -2.61555603 | 0.003468916 | DOWN |
| ENSSSCG00000009966 | MN1 | -2.617880488 | 6.77866E-08 | DOWN |
| ENSSSCG00000001975 | PRKD1 | -2.621356216 | 1.32702E-15 | DOWN |
| ENSSSCG00000016294 | C2orf82 | -2.627620551 | 0.042199913 | DOWN |
| ENSSSCG00000017938 | YBX2 | -2.631772779 | 1.64171E-06 | DOWN |
| ENSSSCG00000040581 | CISH | -2.634114292 | 2.424E-13 | DOWN |
| ENSSSCG00000006497 | MEX3A | -2.651203588 | 0.032912184 | DOWN |
| ENSSSCG00000016873 | NIM1K | -2.657576762 | 1.15711E-06 | DOWN |
| ENSSSCG00000002637 | NA | -2.659488049 | 0.000135767 | DOWN |
| ENSSSCG00000003592 | SDC3 | -2.659836989 | 4.10362E-07 | DOWN |
| ENSSSCG00000012570 | NA | -2.665137843 | 0.007277157 | DOWN |
| ENSSSCG00000017257 | ABCA9 | -2.666421391 | 5.68073E-05 | DOWN |
| ENSSSCG00000035294 | NA | -2.674297383 | 0.003051783 | DOWN |
| ENSSSCG00000011309 | KIF15 | -2.679648423 | 0.000181556 | DOWN |
| ENSSSCG00000016958 | PIK3R1 | -2.68741267 | 1.63319E-08 | DOWN |
| ENSSSCG00000003088 | APOE | -2.689184801 | 0.012827506 | DOWN |
| ENSSSCG00000009839 | NA | -2.694573716 | 0.004990116 | DOWN |
| ENSSSCG00000037307 | NA | -2.695831779 | 1.0656E-05 | DOWN |
| ENSSSCG00000007572 | LFNG | -2.695936614 | 1.07465E-07 | DOWN |
| ENSSSCG00000006475 | IQGAP3 | -2.706001786 | 1.61091E-08 | DOWN |
| ENSSSCG00000035299 | SFRP4 | -2.711952106 | 0.044666381 | DOWN |
| ENSSSCG00000007423 | UBE2C | -2.714785973 | 3.1966E-05 | DOWN |
| ENSSSCG00000002849 | NA | -2.731628641 | 0.000177274 | DOWN |
| ENSSSCG00000000744 | NA | -2.732722565 | 0.036880566 | DOWN |
| ENSSSCG00000007358 | EMILIN3 | -2.73744874 | 0.001791858 | DOWN |
| ENSSSCG00000037288 | YPEL1 | -2.74326603 | 0.000114386 | DOWN |
| ENSSSCG00000036157 | BARX2 | -2.743429524 | 0.01529324 | DOWN |
| ENSSSCG00000007463 | PTGIS | -2.755259684 | 4.88213E-28 | DOWN |
| ENSSSCG00000024954 | FGF1 | -2.755876507 | 0.000259885 | DOWN |
| ENSSSCG00000005627 | AK1 | -2.756055194 | 7.03112E-10 | DOWN |
| ENSSSCG00000017473 | TOP2A | -2.758648806 | 6.10498E-05 | DOWN |
| ENSSSCG00000023004 | FZD9 | -2.762812724 | 0.00051935 | DOWN |
| ENSSSCG00000001490 | KHDRBS2 | -2.766824174 | 0.0243521 | DOWN |
| ENSSSCG00000014994 | PDGFD | -2.770654014 | 4.82429E-13 | DOWN |
| ENSSSCG00000024403 | PRRT1 | -2.783342783 | 0.000658265 | DOWN |
| ENSSSCG00000039358 | SUSD5 | -2.78726945 | 0.001826302 | DOWN |
| ENSSSCG00000000602 | RERG | -2.798843693 | 3.94575E-06 | DOWN |
| ENSSSCG00000010543 | ABCC2 | -2.800515753 | 0.006970447 | DOWN |
| ENSSSCG00000011294 | FAM198A | -2.802479245 | 4.17816E-09 | DOWN |
| ENSSSCG00000011207 | SGO1 | -2.805573647 | 0.000268779 | DOWN |
| ENSSSCG00000007949 | SRL | -2.807180422 | 0.001230699 | DOWN |
| ENSSSCG00000014149 | MEF2C | -2.817417749 | 1.85845E-11 | DOWN |
| ENSSSCG00000012076 | MX2 | -2.820198713 | 0.004633573 | DOWN |
| ENSSSCG00000035055 | NA | -2.834017286 | 0.004016297 | DOWN |
| ENSSSCG00000021941 | NA | -2.834173908 | 0.000811153 | DOWN |
| ENSSSCG00000008832 | LRRC66 | -2.849997124 | 0.029273599 | DOWN |
| ENSSSCG00000033259 | C1orf228 | -2.851860102 | 0.000662351 | DOWN |
| ENSSSCG00000016018 | FRZB | -2.8529672 | 0.002461722 | DOWN |
| ENSSSCG00000038838 | DLX5 | -2.853528667 | 2.6496E-09 | DOWN |
| ENSSSCG00000040951 | SCT | -2.854627086 | 0.033957086 | DOWN |
| ENSSSCG00000008446 | SIX2 | -2.856343309 | 8.36794E-10 | DOWN |
| ENSSSCG00000016866 | GHR | -2.868026505 | 2.75912E-21 | DOWN |
| ENSSSCG00000004291 | NT5E | -2.879597146 | 1.42876E-09 | DOWN |
| ENSSSCG00000006748 | TSPAN2 | -2.904910496 | 1.97844E-08 | DOWN |
| ENSSSCG00000031819 | TP53I11 | -2.90754359 | 0.003654336 | DOWN |
| ENSSSCG00000012637 | KLHL13 | -2.91277285 | 0.033373628 | DOWN |
| ENSSSCG00000035798 | PRTG | -2.917744689 | 0.002916275 | DOWN |
| ENSSSCG00000026146 | GALNT5 | -2.918004611 | 5.27631E-07 | DOWN |
| ENSSSCG00000017643 | SEPT4 | -2.919616337 | 2.94069E-09 | DOWN |
| ENSSSCG00000015073 | TAGLN | -2.924648868 | 0.000372754 | DOWN |
| ENSSSCG00000010471 | KIF11 | -2.927088272 | 8.20375E-06 | DOWN |
| ENSSSCG00000004928 | CILP | -2.934854162 | 1.33498E-06 | DOWN |
| ENSSSCG00000010272 | ADAMTS14 | -2.936367202 | 0.003496626 | DOWN |
| ENSSSCG00000004969 | NA | -2.946762828 | 8.1322E-05 | DOWN |
| ENSSSCG00000015281 | PLEKHA6 | -2.946997054 | 0.003891436 | DOWN |
| ENSSSCG00000028284 | KCND1 | -2.954117275 | 0.027997569 | DOWN |
| ENSSSCG00000029260 | NDNF | -2.956948363 | 8.93467E-05 | DOWN |
| ENSSSCG00000037105 | EVL | -2.960998472 | 1.49009E-06 | DOWN |
| ENSSSCG00000009671 | PBK | -2.963909407 | 6.10726E-07 | DOWN |
| ENSSSCG00000016328 | RAB17 | -2.964716169 | 0.034326507 | DOWN |
| ENSSSCG00000037195 | FOXF2 | -2.981590718 | 0.018950231 | DOWN |
| ENSSSCG00000029326 | CCNB1 | -2.985773011 | 9.7125E-05 | DOWN |
| ENSSSCG00000015913 | SCN9A | -2.986112819 | 1.91242E-07 | DOWN |
| ENSSSCG00000038693 | RAB19 | -3.011303599 | 0.006245245 | DOWN |
| ENSSSCG00000013916 | COMP | -3.014192033 | 1.49181E-09 | DOWN |
| ENSSSCG00000007528 | PHACTR3 | -3.033631882 | 0.000666639 | DOWN |
| ENSSSCG00000026943 | MRAP2 | -3.038345224 | 0.014556185 | DOWN |
| ENSSSCG00000001710 | RUNX2 | -3.049618264 | 0.025577717 | DOWN |
| ENSSSCG00000017569 | CHAD | -3.054694062 | 3.58765E-07 | DOWN |
| ENSSSCG00000033657 | GREM1 | -3.069529471 | 0.000108737 | DOWN |
| ENSSSCG00000000464 | C12orf56 | -3.071629482 | 0.021588395 | DOWN |
| ENSSSCG00000013598 | KANK3 | -3.087428884 | 6.8866E-06 | DOWN |
| ENSSSCG00000013294 | LDLRAD3 | -3.101309382 | 1.12592E-08 | DOWN |
| ENSSSCG00000014326 | KIF20A | -3.116553714 | 2.75879E-06 | DOWN |
| ENSSSCG00000026748 | PLK1 | -3.126225006 | 0.000373664 | DOWN |
| ENSSSCG00000038077 | PPP1R14A | -3.134963892 | 0.001395028 | DOWN |
| ENSSSCG00000011582 | CAND2 | -3.137218527 | 6.02664E-17 | DOWN |
| ENSSSCG00000017129 | NA | -3.139411897 | 0.009726177 | DOWN |
| ENSSSCG00000038143 | NA | -3.142460194 | 0.002178465 | DOWN |
| ENSSSCG00000024145 | PRR11 | -3.145167974 | 2.03563E-05 | DOWN |
| ENSSSCG00000038384 | COX4I2 | -3.153443238 | 5.44326E-09 | DOWN |
| ENSSSCG00000023585 | SERINC2 | -3.16941233 | 0.000327002 | DOWN |
| ENSSSCG00000017563 | MYCBPAP | -3.176450089 | 0.015599599 | DOWN |
| ENSSSCG00000014095 | ZBED3 | -3.195759274 | 1.35159E-05 | DOWN |
| ENSSSCG00000006958 | TOP1MT | -3.203125417 | 0.027123669 | DOWN |
| ENSSSCG00000028203 | NA | -3.205849268 | 0.010558564 | DOWN |
| ENSSSCG00000040631 | LPL | -3.22147265 | 0.001332751 | DOWN |
| ENSSSCG00000034570 | IFI6 | -3.222967288 | 4.64586E-06 | DOWN |
| ENSSSCG00000016838 | RANBP3L | -3.246895615 | 5.2058E-09 | DOWN |
| ENSSSCG00000012975 | SNX32 | -3.249222917 | 3.86744E-05 | DOWN |
| ENSSSCG00000011401 | LSMEM2 | -3.251341855 | 0.01367531 | DOWN |
| ENSSSCG00000011326 | PTH1R | -3.253611851 | 1.20098E-09 | DOWN |
| ENSSSCG00000012882 | NA | -3.25432137 | 0.001470147 | DOWN |
| ENSSSCG00000011455 | CACNA1D | -3.265954973 | 5.30102E-05 | DOWN |
| ENSSSCG00000026257 | STMN1 | -3.277275906 | 3.82359E-08 | DOWN |
| ENSSSCG00000013403 | GALNT18 | -3.279352836 | 5.80412E-12 | DOWN |
| ENSSSCG00000015581 | CENPF | -3.285286678 | 0.000236296 | DOWN |
| ENSSSCG00000004201 | TMEM200A | -3.292992633 | 0.005593083 | DOWN |
| ENSSSCG00000000857 | IGF1 | -3.31465934 | 4.81405E-10 | DOWN |
| ENSSSCG00000013049 | RCOR2 | -3.317880663 | 2.96616E-21 | DOWN |
| ENSSSCG00000036437 | NOG | -3.320908886 | 2.90632E-05 | DOWN |
| ENSSSCG00000025523 | COL2A1 | -3.326163291 | 0.000646858 | DOWN |
| ENSSSCG00000038688 | PGLYRP1 | -3.346905197 | 0.001507795 | DOWN |
| ENSSSCG00000002712 | LDHD | -3.348249132 | 2.49581E-10 | DOWN |
| ENSSSCG00000003147 | CA11 | -3.349450954 | 1.92739E-11 | DOWN |
| ENSSSCG00000030325 | C1QTNF6 | -3.351135272 | 8.70527E-14 | DOWN |
| ENSSSCG00000031487 | LSP1 | -3.357368311 | 2.18166E-13 | DOWN |
| ENSSSCG00000000206 | FAIM2 | -3.371426421 | 0.041029391 | DOWN |
| ENSSSCG00000038522 | FAM162B | -3.373937087 | 0.010767751 | DOWN |
| ENSSSCG00000013385 | INSC | -3.383270346 | 7.00326E-05 | DOWN |
| ENSSSCG00000034681 | NA | -3.393359621 | 0.025798203 | DOWN |
| ENSSSCG00000028148 | DMD | -3.408533677 | 7.74088E-06 | DOWN |
| ENSSSCG00000031903 | TNNT3 | -3.409007701 | 0.02368261 | DOWN |
| ENSSSCG00000008397 | EFEMP1 | -3.417318528 | 3.02367E-05 | DOWN |
| ENSSSCG00000035419 | RARRES2 | -3.424099143 | 1.24606E-13 | DOWN |
| ENSSSCG00000029949 | CD248 | -3.427718473 | 0.000482518 | DOWN |
| ENSSSCG00000024223 | ARHGEF16 | -3.433268245 | 1.04422E-06 | DOWN |
| ENSSSCG00000007344 | KIAA1755 | -3.451738684 | 5.57222E-09 | DOWN |
| ENSSSCG00000003333 | C1QTNF12 | -3.458064607 | 0.003760294 | DOWN |
| ENSSSCG00000021656 | NA | -3.468948121 | 0.000182398 | DOWN |
| ENSSSCG00000011911 | DRD3 | -3.475012515 | 0.013480168 | DOWN |
| ENSSSCG00000033509 | SAMD11 | -3.476634251 | 1.11458E-13 | DOWN |
| ENSSSCG00000011246 | VILL | -3.480718798 | 0.022871158 | DOWN |
| ENSSSCG00000026130 | EPHA3 | -3.50537973 | 0.026397113 | DOWN |
| ENSSSCG00000010529 | SFRP5 | -3.516992363 | 1.50186E-06 | DOWN |
| ENSSSCG00000008725 | CYTL1 | -3.517551306 | 0.012886519 | DOWN |
| ENSSSCG00000000874 | GAS2L3 | -3.526365962 | 3.81145E-08 | DOWN |
| ENSSSCG00000000963 | SYCE3 | -3.528754144 | 0.044991091 | DOWN |
| ENSSSCG00000010896 | ASPM | -3.542807034 | 0.000503044 | DOWN |
| ENSSSCG00000014066 | TMEM171 | -3.555184715 | 0.000689674 | DOWN |
| ENSSSCG00000024892 | NSG1 | -3.562596528 | 1.62169E-05 | DOWN |
| ENSSSCG00000031074 | FAM110D | -3.569889233 | 9.18765E-07 | DOWN |
| ENSSSCG00000017041 | ADRA1B | -3.583626131 | 0.044774981 | DOWN |
| ENSSSCG00000038905 | MSMP | -3.592902769 | 0.013029965 | DOWN |
| ENSSSCG00000008103 | MERTK | -3.605460296 | 0.024143327 | DOWN |
| ENSSSCG00000004534 | CCDC68 | -3.614587425 | 0.015145733 | DOWN |
| ENSSSCG00000022206 | UOX | -3.616226459 | 0.000963462 | DOWN |
| ENSSSCG00000016841 | SLC1A3 | -3.634421919 | 4.517E-05 | DOWN |
| ENSSSCG00000029395 | TMEM25 | -3.636584879 | 0.0214046 | DOWN |
| ENSSSCG00000012307 | CCNB3 | -3.654631756 | 0.000150457 | DOWN |
| ENSSSCG00000021540 | C2CD4D | -3.670046416 | 0.005350251 | DOWN |
| ENSSSCG00000033759 | TBXA2R | -3.689093872 | 0.000419757 | DOWN |
| ENSSSCG00000021374 | FXYD1 | -3.695564948 | 2.74259E-05 | DOWN |
| ENSSSCG00000040811 | NA | -3.699470013 | 0.035891669 | DOWN |
| ENSSSCG00000002476 | SERPINA1 | -3.701611726 | 0.044951085 | DOWN |
| ENSSSCG00000008501 | VIT | -3.709711276 | 2.16713E-05 | DOWN |
| ENSSSCG00000032048 | SCRG1 | -3.710905103 | 0.000184981 | DOWN |
| ENSSSCG00000004807 | SCG5 | -3.750981991 | 0.006508721 | DOWN |
| ENSSSCG00000028529 | REM1 | -3.757441633 | 1.37203E-13 | DOWN |
| ENSSSCG00000016290 | EFHD1 | -3.768065863 | 0.000122573 | DOWN |
| ENSSSCG00000015250 | ADAMTS15 | -3.772306104 | 0.004641688 | DOWN |
| ENSSSCG00000025777 | ESR1 | -3.7810973 | 9.39988E-13 | DOWN |
| ENSSSCG00000007385 | KCNS1 | -3.79007846 | 3.69947E-06 | DOWN |
| ENSSSCG00000026407 | NCCRP1 | -3.791444968 | 2.07262E-10 | DOWN |
| ENSSSCG00000000688 | LAG3 | -3.794384029 | 0.000135913 | DOWN |
| ENSSSCG00000037846 | ACOXL | -3.803994835 | 0.002037745 | DOWN |
| ENSSSCG00000040513 | AQP3 | -3.817316467 | 5.53402E-05 | DOWN |
| ENSSSCG00000009631 | PEBP4 | -3.819123907 | 0.048403549 | DOWN |
| ENSSSCG00000035729 | MYOZ3 | -3.824946899 | 5.50769E-06 | DOWN |
| ENSSSCG00000034993 | NA | -3.843500117 | 4.46078E-10 | DOWN |
| ENSSSCG00000009222 | SPARCL1 | -3.84771977 | 0.001501267 | DOWN |
| ENSSSCG00000002464 | PRIMA1 | -3.850265639 | 0.044728403 | DOWN |
| ENSSSCG00000036474 | GJB6 | -3.870450591 | 0.033020692 | DOWN |
| ENSSSCG00000008101 | FBLN7 | -3.879902131 | 1.22178E-11 | DOWN |
| ENSSSCG00000003508 | KIF17 | -3.887469275 | 0.045867106 | DOWN |
| ENSSSCG00000000029 | SCUBE1 | -3.906721642 | 0.000182546 | DOWN |
| ENSSSCG00000029227 | LDB2 | -3.914132293 | 1.31861E-08 | DOWN |
| ENSSSCG00000017181 | CYGB | -3.919823455 | 1.6752E-06 | DOWN |
| ENSSSCG00000022026 | AIPL1 | -3.932763863 | 0.033272274 | DOWN |
| ENSSSCG00000002831 | IRX3 | -3.964321174 | 0.035958102 | DOWN |
| ENSSSCG00000040607 | MAF | -3.965057796 | 4.1306E-11 | DOWN |
| ENSSSCG00000000234 | GRASP | -3.967873094 | 0.019231529 | DOWN |
| ENSSSCG00000029545 | GPRC5D | -3.970481904 | 0.005532259 | DOWN |
| ENSSSCG00000029558 | EXTL1 | -3.982728673 | 0.004842826 | DOWN |
| ENSSSCG00000003521 | NA | -3.982927179 | 0.036500352 | DOWN |
| ENSSSCG00000037821 | RGS5 | -3.985948576 | 0.00387165 | DOWN |
| ENSSSCG00000017893 | PIMREG | -4.000946437 | 2.01096E-05 | DOWN |
| ENSSSCG00000013337 | BBOX1 | -4.044921161 | 0.047471698 | DOWN |
| ENSSSCG00000009580 | S1PR3 | -4.056783051 | 0.022940541 | DOWN |
| ENSSSCG00000009429 | TNFSF11 | -4.079456383 | 0.047107714 | DOWN |
| ENSSSCG00000000791 | PDZRN4 | -4.079859791 | 0.000237783 | DOWN |
| ENSSSCG00000027157 | SLC40A1 | -4.080929657 | 2.51018E-16 | DOWN |
| ENSSSCG00000033344 | H19_2 | -4.092276808 | 0.009209981 | DOWN |
| ENSSSCG00000007391 | MATN4 | -4.095824985 | 4.02648E-07 | DOWN |
| ENSSSCG00000025034 | LAPTM5 | -4.097463142 | 0.001156186 | DOWN |
| ENSSSCG00000001989 | CIDEB | -4.117684662 | 0.047346807 | DOWN |
| ENSSSCG00000011641 | SLCO2A1 | -4.140739454 | 0.002746557 | DOWN |
| ENSSSCG00000011538 | LMCD1 | -4.143789819 | 5.16444E-09 | DOWN |
| ENSSSCG00000024492 | EPHB1 | -4.162369375 | 3.77465E-05 | DOWN |
| ENSSSCG00000022390 | RGN | -4.165187782 | 0.0033937 | DOWN |
| ENSSSCG00000016810 | PDZD2 | -4.170745072 | 0.001298043 | DOWN |
| ENSSSCG00000012408 | NHSL2 | -4.177234565 | 0.000348387 | DOWN |
| ENSSSCG00000037697 | MGP | -4.221953752 | 7.9264E-06 | DOWN |
| ENSSSCG00000014272 | NA | -4.224016048 | 0.004838379 | DOWN |
| ENSSSCG00000010056 | NA | -4.239340827 | 7.14412E-05 | DOWN |
| ENSSSCG00000035224 | NA | -4.25297708 | 0.004529783 | DOWN |
| ENSSSCG00000008263 | M1AP | -4.256633874 | 0.043510748 | DOWN |
| ENSSSCG00000035020 | STK32B | -4.257878386 | 0.040786594 | DOWN |
| ENSSSCG00000004218 | RSPO3 | -4.281302444 | 0.002336131 | DOWN |
| ENSSSCG00000023760 | CLEC14A | -4.285039209 | 0.00125345 | DOWN |
| ENSSSCG00000010545 | CPN1 | -4.324314277 | 0.003675902 | DOWN |
| ENSSSCG00000011324 | NA | -4.334980388 | 0.015287939 | DOWN |
| ENSSSCG00000038801 | NPNT | -4.373471454 | 0.018536948 | DOWN |
| ENSSSCG00000010402 | ZFAND4 | -4.380425428 | 0.014581055 | DOWN |
| ENSSSCG00000001723 | PLA2G7 | -4.385652754 | 0.04393223 | DOWN |
| ENSSSCG00000006472 | CRABP2 | -4.427351055 | 2.20427E-08 | DOWN |
| ENSSSCG00000002764 | SMPD3 | -4.45030224 | 0.000518085 | DOWN |
| ENSSSCG00000034604 | CLEC3A | -4.46682855 | 3.57741E-06 | DOWN |
| ENSSSCG00000013273 | CHST1 | -4.47306461 | 8.3165E-06 | DOWN |
| ENSSSCG00000032400 | C1QTNF8 | -4.485793093 | 0.000137707 | DOWN |
| ENSSSCG00000017770 | PROCA1 | -4.496570246 | 0.00186314 | DOWN |
| ENSSSCG00000039392 | SNPH | -4.498181147 | 0.009200575 | DOWN |
| ENSSSCG00000013892 | KCNN1 | -4.500344184 | 0.000381429 | DOWN |
| ENSSSCG00000038543 | CENPA | -4.546102275 | 3.87593E-07 | DOWN |
| ENSSSCG00000010800 | B3GALT2 | -4.554045965 | 0.03248164 | DOWN |
| ENSSSCG00000030732 | GP1BB | -4.576168079 | 0.025804615 | DOWN |
| ENSSSCG00000003374 | ESPN | -4.579490602 | 2.15711E-06 | DOWN |
| ENSSSCG00000025858 | ELN | -4.584265092 | 6.78299E-12 | DOWN |
| ENSSSCG00000012121 | EGFL6 | -4.590052112 | 0.022100355 | DOWN |
| ENSSSCG00000015334 | PDK4 | -4.601830002 | 0.009461728 | DOWN |
| ENSSSCG00000026254 | PRRG3 | -4.611337103 | 0.036046554 | DOWN |
| ENSSSCG00000006919 | NA | -4.669658923 | 0.026564041 | DOWN |
| ENSSSCG00000026894 | NFE2L3 | -4.67532377 | 0.029610282 | DOWN |
| ENSSSCG00000010728 | CPXM2 | -4.679327858 | 1.23577E-07 | DOWN |
| ENSSSCG00000006034 | RSPO2 | -4.693060874 | 1.42876E-09 | DOWN |
| ENSSSCG00000034181 | NKX3-2 | -4.699451195 | 6.11791E-06 | DOWN |
| ENSSSCG00000006031 | TMEM74 | -4.71067777 | 0.000802922 | DOWN |
| ENSSSCG00000022592 | FIBIN | -4.747908844 | 3.42086E-21 | DOWN |
| ENSSSCG00000000781 | ABCD2 | -4.803955564 | 0.000167245 | DOWN |
| ENSSSCG00000015474 | PPFIA4 | -4.850730671 | 0.005233153 | DOWN |
| ENSSSCG00000038685 | MPPED1 | -4.855186004 | 6.10726E-07 | DOWN |
| ENSSSCG00000011217 | NEK10 | -4.883030577 | 0.011969542 | DOWN |
| ENSSSCG00000011241 | DCLK3 | -4.927055142 | 0.010739047 | DOWN |
| ENSSSCG00000027768 | SLC29A4 | -4.957049375 | 0.028155876 | DOWN |
| ENSSSCG00000011527 | CNTN4 | -4.967624742 | 0.034237042 | DOWN |
| ENSSSCG00000004309 | CNR1 | -4.970020337 | 0.021469259 | DOWN |
| ENSSSCG00000006474 | NES | -4.977513751 | 0.029290015 | DOWN |
| ENSSSCG00000010428 | DKK1 | -4.984197344 | 0.002052314 | DOWN |
| ENSSSCG00000003410 | MASP2 | -4.993513344 | 1.39398E-12 | DOWN |
| ENSSSCG00000004678 | DUOX2 | -5.021688398 | 0.015525543 | DOWN |
| ENSSSCG00000030485 | ELFN1 | -5.023441288 | 0.008550334 | DOWN |
| ENSSSCG00000022543 | VWDE | -5.028093742 | 0.018137073 | DOWN |
| ENSSSCG00000012584 | CAPN6 | -5.030701487 | 3.14869E-09 | DOWN |
| ENSSSCG00000026517 | CALML4 | -5.040737965 | 7.05335E-10 | DOWN |
| ENSSSCG00000032398 | VGLL3 | -5.122219599 | 0.010536668 | DOWN |
| ENSSSCG00000028996 | ALDH1A1 | -5.15756957 | 0.003520089 | DOWN |
| ENSSSCG00000010086 | NA | -5.260552314 | 0.004242849 | DOWN |
| ENSSSCG00000017498 | PPP1R1B | -5.271579285 | 0.000819511 | DOWN |
| ENSSSCG00000000584 | SLCO1A2 | -5.318394204 | 0.008876625 | DOWN |
| ENSSSCG00000037067 | NA | -5.361069371 | 0.001662129 | DOWN |
| ENSSSCG00000011299 | CLEC3B | -5.39394871 | 1.24295E-09 | DOWN |
| ENSSSCG00000005352 | FRMPD1 | -5.424369722 | 0.023900131 | DOWN |
| ENSSSCG00000031648 | WISP2 | -5.446577494 | 0.001775406 | DOWN |
| ENSSSCG00000029334 | MYT1L | -5.450852038 | 0.009401331 | DOWN |
| ENSSSCG00000030921 | APOA1 | -5.478285265 | 0.009902774 | DOWN |
| ENSSSCG00000009498 | NA | -5.502315641 | 0.040425679 | DOWN |
| ENSSSCG00000040031 | SMIM32 | -5.622407839 | 0.029490744 | DOWN |
| ENSSSCG00000031154 | ACKR2 | -5.642683667 | 3.49211E-07 | DOWN |
| ENSSSCG00000015753 | ANGPT2 | -5.668401697 | 3.97268E-07 | DOWN |
| ENSSSCG00000012490 | TMEM35A | -5.677461208 | 0.000148898 | DOWN |
| ENSSSCG00000001479 | HMGCLL1 | -5.680645491 | 0.016453356 | DOWN |
| ENSSSCG00000018758 | ssc-mir-214 | -5.700690536 | 0.000119029 | DOWN |
| ENSSSCG00000006171 | CRISPLD1 | -5.798206201 | 6.9919E-26 | DOWN |
| ENSSSCG00000012558 | PIH1D3 | -5.819357396 | 0.002681924 | DOWN |
| ENSSSCG00000006852 | NTNG1 | -5.872191391 | 0.005658848 | DOWN |
| ENSSSCG00000027684 | TRIM63 | -5.881087553 | 0.006017208 | DOWN |
| ENSSSCG00000004402 | METTL24 | -5.881772673 | 0.024903137 | DOWN |
| ENSSSCG00000008306 | NOTO | -5.910711403 | 0.00133391 | DOWN |
| ENSSSCG00000014827 | PLEKHB1 | -5.936279247 | 0.00032841 | DOWN |
| ENSSSCG00000010912 | KIF14 | -6.090601523 | 0.000130735 | DOWN |
| ENSSSCG00000009219 | IBSP | -6.103242812 | 0.000293535 | DOWN |
| ENSSSCG00000005740 | SARDH | -6.202699572 | 0.010292418 | DOWN |
| ENSSSCG00000007507 | PCK1 | -6.277814783 | 0.003763531 | DOWN |
| ENSSSCG00000000623 | BCL2L14 | -6.286809018 | 0.000377646 | DOWN |
| ENSSSCG00000006307 | RCSD1 | -6.441199761 | 6.48804E-06 | DOWN |
| ENSSSCG00000022554 | MATN1 | -6.454465334 | 0.00024624 | DOWN |
| ENSSSCG00000033234 | SSTR5 | -6.55823906 | 0.00040747 | DOWN |
| ENSSSCG00000026932 | RS1 | -6.609552208 | 8.36496E-06 | DOWN |
| ENSSSCG00000009517 | GPR183 | -6.650885172 | 0.004498675 | DOWN |
| ENSSSCG00000030998 | WIF1 | -6.783444124 | 1.93687E-07 | DOWN |
| ENSSSCG00000025005 | B4GALT6 | -6.87246458 | 0.00025794 | DOWN |
| ENSSSCG00000006051 | CTHRC1 | -6.937789266 | 0.000726748 | DOWN |
| ENSSSCG00000021573 | KCNJ5 | -6.992214313 | 3.67585E-06 | DOWN |
| ENSSSCG00000011397 | SLC38A3 | -7.530187568 | 4.83917E-10 | DOWN |
| ENSSSCG00000028896 | DIO1 | -7.842884574 | 0.001907695 | DOWN |
| ENSSSCG00000008842 | KIT | -7.96894114 | 1.67718E-06 | DOWN |
| ENSSSCG00000010376 | GDF10 | -8.148831192 | 1.31586E-11 | DOWN |
| ENSSSCG00000025483 | GREB1 | -8.243271936 | 2.44756E-07 | DOWN |
| ENSSSCG00000014843 | CHRDL2 | -8.425329254 | 3.59398E-07 | DOWN |
| ENSSSCG00000011646 | KY | -8.762651493 | 0.00010585 | DOWN |

Gene Name “NA” indicates the gene ID was not matched to a HGNC gene name.
