# Supplementary material for: A Tale of Two Loads: Modulation of IL-1 Induced Inflammatory Responses of Meniscal Cells in Two Models of Dynamic Physiologic Loading
Source: Front Bioeng Biotechnol. 2022 Mar 1;10:837619. doi: 10.3389/fbioe.2022.837619 (PMC8921261; doi:10.3389/fbioe.2022.837619)
Supplement: Supplementary file 13 [file DataSheet8.DOCX]

**Supplemental Table 9**: 5% stretch compared to 0% stretch for outer zone cells with exogenous IL-1α stimulation.

| **Gene ID** | **Gene Name** | **Log2Fold Change** | **p-value** | **Up/Down Regulated** |
| --- | --- | --- | --- | --- |
| ENSSSCG00000007477 | NFATC2 | 1.636833 | 1.22E-123 | UP |
| ENSSSCG00000034114 | GPR68 | 1.101139 | 1.40E-79 | UP |
| ENSSSCG00000036060 | RRAD | 2.453191 | 6.85E-69 | UP |
| ENSSSCG00000039780 | RTN4RL1 | 1.269437 | 1.43E-44 | UP |
| ENSSSCG00000014235 | SNCAIP | 1.146818 | 1.42E-27 | UP |
| ENSSSCG00000038965 | ARC | 1.782916 | 2.38E-21 | UP |
| ENSSSCG00000014909 | NA | 1.053571 | 3.98E-19 | UP |
| ENSSSCG00000001061 | JARID2 | 1.021236 | 7.47E-17 | UP |
| ENSSSCG00000021576 | CD83 | 1.079934 | 1.08E-16 | UP |
| ENSSSCG00000001620 | MDFI | 1.096936 | 1.28E-16 | UP |
| ENSSSCG00000029656 | NDP | 1.56369 | 1.25E-14 | UP |
| ENSSSCG00000007682 | SH2B2 | 1.035571 | 1.73E-12 | UP |
| ENSSSCG00000009580 | S1PR3 | 1.003554 | 3.77E-12 | UP |
| ENSSSCG00000015487 | TNFSF18 | 1.013286 | 4.81E-12 | UP |
| ENSSSCG00000033327 | PDGFB | 1.536443 | 8.58E-12 | UP |
| ENSSSCG00000005992 | SHAS2 | 1.179195 | 1.41E-11 | UP |
| ENSSSCG00000035077 | INHBA | 1.267864 | 2.05E-11 | UP |
| ENSSSCG00000018047 | FAM83G | 1.720327 | 2.51E-11 | UP |
| ENSSSCG00000016573 | IRF5 | 1.282503 | 5.30E-10 | UP |
| ENSSSCG00000040388 | ZBTB46 | 1.423817 | 1.64E-08 | UP |
| ENSSSCG00000033397 | KCNK9 | 1.778593 | 6.11E-08 | UP |
| ENSSSCG00000014448 | ARSI | 1.273333 | 1.10E-07 | UP |
| ENSSSCG00000006235 | TOX | 1.211487 | 1.20E-07 | UP |
| ENSSSCG00000007572 | LFNG | 1.046783 | 2.37E-07 | UP |
| ENSSSCG00000008689 | ZFYVE28 | 1.560419 | 9.43E-07 | UP |
| ENSSSCG00000009676 | ZNF395 | 1.247384 | 3.44E-06 | UP |
| ENSSSCG00000011538 | LMCD1 | 1.596134 | 5.70E-06 | UP |
| ENSSSCG00000012104 | NA | 1.14547 | 8.19E-06 | UP |
| ENSSSCG00000036364 | EGR4 | 1.626817 | 1.19E-05 | UP |
| ENSSSCG00000001473 | COL11A2 | 1.555728 | 4.05E-05 | UP |
| ENSSSCG00000012034 | TIAM1 | 1.132697 | 7.82E-05 | UP |
| ENSSSCG00000006902 | GFI1 | 1.607526 | 0.000171 | UP |
| ENSSSCG00000040636 | KRT80 | 1.170496 | 0.000235 | UP |
| ENSSSCG00000006497 | MEX3A | 1.017299 | 0.000256 | UP |
| ENSSSCG00000036768 | PRAG1 | 1.074023 | 0.000287 | UP |
| ENSSSCG00000033759 | TBXA2R | 1.245369 | 0.00037 | UP |
| ENSSSCG00000001597 | LRFN2 | 1.483167 | 0.000404 | UP |
| ENSSSCG00000023487 | MSLNL | 2.382585 | 0.000471 | UP |
| ENSSSCG00000003069 | KCNN4 | 1.208014 | 0.000487 | UP |
| ENSSSCG00000006758 | SYT6 | 1.56221 | 0.000798 | UP |
| ENSSSCG00000017041 | ADRA1B | 1.313095 | 0.001024 | UP |
| ENSSSCG00000038643 | KLF11 | 1.517949 | 0.001757 | UP |
| ENSSSCG00000037856 | EDN2 | 4.446907 | 0.002456 | UP |
| ENSSSCG00000009278 | FGF9 | 1.464958 | 0.002639 | UP |
| ENSSSCG00000010031 | NA | 1.089076 | 0.002671 | UP |
| ENSSSCG00000010222 | ZNF365 | 1.350542 | 0.003277 | UP |
| ENSSSCG00000011721 | P2RY1 | 1.445269 | 0.003475 | UP |
| ENSSSCG00000038886 | CAGE1 | 1.429289 | 0.003504 | UP |
| ENSSSCG00000029796 | KBTBD11 | 1.814899 | 0.004579 | UP |
| ENSSSCG00000016887 | ITGA2 | 1.744744 | 0.005303 | UP |
| ENSSSCG00000022380 | SH2D3A | 1.163128 | 0.006977 | UP |
| ENSSSCG00000038296 | NA | 1.361766 | 0.007108 | UP |
| ENSSSCG00000010974 | CNTFR | 1.154356 | 0.007347 | UP |
| ENSSSCG00000027405 | U2 | 1.395459 | 0.007822 | UP |
| ENSSSCG00000006286 | NA | 1.076855 | 0.01386 | UP |
| ENSSSCG00000000791 | PDZRN4 | 1.078461 | 0.01527 | UP |
| ENSSSCG00000002507 | BCL11B | 1.643059 | 0.016088 | UP |
| ENSSSCG00000017223 | USH1G | 3.94533 | 0.017532 | UP |
| ENSSSCG00000006336 | CCDC190 | 1.458932 | 0.018623 | UP |
| ENSSSCG00000030675 | MIXL1 | 1.08563 | 0.018635 | UP |
| ENSSSCG00000017349 | ADAM11 | 1.101838 | 0.020512 | UP |
| ENSSSCG00000016992 | NEURL1B | 1.598136 | 0.023816 | UP |
| ENSSSCG00000025992 | NA | 1.298247 | 0.023994 | UP |
| ENSSSCG00000031272 | FGF5 | 1.294135 | 0.024344 | UP |
| ENSSSCG00000035711 | NA | 2.177382 | 0.025102 | UP |
| ENSSSCG00000000531 | BICD1 | 1.023894 | 0.03037 | UP |
| ENSSSCG00000018229 | SNORD74 | 1.010924 | 0.034036 | UP |
| ENSSSCG00000007642 | NA | 1.058324 | 0.036141 | UP |
| ENSSSCG00000000584 | SLCO1A2 | 1.851079 | 0.038138 | UP |
| ENSSSCG00000005216 | NA | 1.684481 | 0.038277 | UP |
| ENSSSCG00000003239 | HAS1 | 1.373485 | 0.038311 | UP |
| ENSSSCG00000009000 | NA | 1.078281 | 0.03876 | UP |
| ENSSSCG00000034609 | RNF112 | 2.053752 | 0.040645 | UP |
| ENSSSCG00000036566 | LY6G6C | 3.506852 | 0.04238 | UP |
| ENSSSCG00000017730 | CDK5R1 | 1.239934 | 0.045172 | UP |
| ENSSSCG00000007139 | ADRA1D | 1.444028 | 0.047137 | UP |
| ENSSSCG00000006829 | SYPL2 | 1.237126 | 0.047274 | UP |
| ENSSSCG00000039821 | GPRIN3 | 2.811393 | 0.048065 | UP |
| ENSSSCG00000032698 | TPPP | 3.257334 | 0.048231 | UP |
| ENSSSCG00000036136 | BHLHE40 | -1.39968 | 9.17E-75 | DOWN |
| ENSSSCG00000016438 | NUB1 | -1.05416 | 4.38E-70 | DOWN |
| ENSSSCG00000040887 | PAPD5 | -1.39811 | 1.34E-63 | DOWN |
| ENSSSCG00000004657 | CEP152 | -1.45092 | 1.53E-62 | DOWN |
| ENSSSCG00000012890 | TCIRG1 | -1.24957 | 7.44E-58 | DOWN |
| ENSSSCG00000004464 | FAM46A | -1.29531 | 1.11E-57 | DOWN |
| ENSSSCG00000031380 | NA | -1.13067 | 5.59E-55 | DOWN |
| ENSSSCG00000035400 | YPEL2 | -1.39254 | 3.51E-53 | DOWN |
| ENSSSCG00000021383 | CGAS | -2.02654 | 2.40E-52 | DOWN |
| ENSSSCG00000032715 | CERS6 | -1.38534 | 4.59E-52 | DOWN |
| ENSSSCG00000004053 | TAGAP | -2.22269 | 2.78E-51 | DOWN |
| ENSSSCG00000001463 | PSMB9 | -1.90639 | 1.71E-50 | DOWN |
| ENSSSCG00000016502 | PARP12 | -1.50414 | 3.31E-49 | DOWN |
| ENSSSCG00000015563 | RGL1 | -1.15164 | 4.55E-49 | DOWN |
| ENSSSCG00000024973 | NA | -3.26004 | 5.74E-49 | DOWN |
| ENSSSCG00000027894 | FAM76A | -1.48452 | 9.84E-49 | DOWN |
| ENSSSCG00000015801 | TLR3 | -1.46113 | 1.73E-48 | DOWN |
| ENSSSCG00000025618 | TAP1 | -1.74051 | 1.87E-48 | DOWN |
| ENSSSCG00000001233 | TRIM26 | -1.21283 | 2.13E-48 | DOWN |
| ENSSSCG00000037572 | EPSTI1 | -2.38264 | 9.37E-48 | DOWN |
| ENSSSCG00000006127 | NBN | -1.23925 | 1.08E-47 | DOWN |
| ENSSSCG00000016512 | ZC3HAV1 | -1.02529 | 3.21E-47 | DOWN |
| ENSSSCG00000001667 | ZNF318 | -1.07493 | 4.02E-47 | DOWN |
| ENSSSCG00000024219 | TIGAR | -1.52961 | 1.95E-46 | DOWN |
| ENSSSCG00000000396 | STAT2 | -1.08043 | 1.95E-46 | DOWN |
| ENSSSCG00000014303 | JADE2 | -1.15061 | 2.08E-46 | DOWN |
| ENSSSCG00000007007 | IDO1 | -1.78875 | 2.57E-46 | DOWN |
| ENSSSCG00000008123 | ARID5A | -1.92629 | 2.31E-42 | DOWN |
| ENSSSCG00000005364 | TDRD7 | -1.09979 | 2.56E-42 | DOWN |
| ENSSSCG00000004897 | ZCCHC2 | -1.15743 | 1.04E-41 | DOWN |
| ENSSSCG00000006066 | RNF19A | -1.04897 | 1.31E-41 | DOWN |
| ENSSSCG00000035634 | NA | -1.19763 | 1.55E-41 | DOWN |
| ENSSSCG00000006625 | RFX5 | -1.32875 | 2.04E-41 | DOWN |
| ENSSSCG00000005724 | SETX | -1.45027 | 2.04E-41 | DOWN |
| ENSSSCG00000015550 | RGS16 | -1.5051 | 2.71E-41 | DOWN |
| ENSSSCG00000029030 | SMCHD1 | -1.14432 | 2.71E-41 | DOWN |
| ENSSSCG00000014277 | IRF1 | -1.70902 | 3.52E-41 | DOWN |
| ENSSSCG00000017087 | GM2A | -1.26166 | 7.25E-41 | DOWN |
| ENSSSCG00000002841 | N4BP1 | -1.23516 | 7.61E-41 | DOWN |
| ENSSSCG00000009469 | ACOD1 | -2.46365 | 8.40E-41 | DOWN |
| ENSSSCG00000006923 | GBP2 | -2.57068 | 1.74E-40 | DOWN |
| ENSSSCG00000015782 | IRF2 | -1.95399 | 2.56E-40 | DOWN |
| ENSSSCG00000017420 | CNP | -1.63162 | 7.08E-40 | DOWN |
| ENSSSCG00000035153 | TRIM38 | -1.53805 | 3.57E-39 | DOWN |
| ENSSSCG00000040648 | CCL11 | -2.63252 | 4.18E-39 | DOWN |
| ENSSSCG00000032360 | PANX1 | -1.34346 | 7.64E-39 | DOWN |
| ENSSSCG00000029438 | SESN2 | -1.01286 | 1.83E-38 | DOWN |
| ENSSSCG00000014780 | TRIM21 | -1.11086 | 3.82E-38 | DOWN |
| ENSSSCG00000031610 | NA | -3.57309 | 5.02E-38 | DOWN |
| ENSSSCG00000004830 | ATP10A | -1.13507 | 7.78E-38 | DOWN |
| ENSSSCG00000001509 | DAXX | -1.02666 | 5.26E-37 | DOWN |
| ENSSSCG00000013551 | C3 | -2.1866 | 1.24E-36 | DOWN |
| ENSSSCG00000033613 | FOXS1 | -2.81392 | 2.08E-36 | DOWN |
| ENSSSCG00000027806 | SAMHD1 | -1.04682 | 4.00E-36 | DOWN |
| ENSSSCG00000033089 | NA | -3.61274 | 4.78E-36 | DOWN |
| ENSSSCG00000022101 | BRCA1 | -1.00786 | 6.53E-36 | DOWN |
| ENSSSCG00000011874 | PARP14 | -2.82542 | 9.42E-36 | DOWN |
| ENSSSCG00000015299 | STEAP4 | -2.62331 | 9.49E-35 | DOWN |
| ENSSSCG00000016261 | SP110 | -1.0784 | 2.16E-34 | DOWN |
| ENSSSCG00000034980 | IRF8 | -3.33069 | 3.22E-34 | DOWN |
| ENSSSCG00000025206 | RNF19B | -1.02364 | 2.13E-33 | DOWN |
| ENSSSCG00000002392 | IRF2BPL | -1.04208 | 2.27E-33 | DOWN |
| ENSSSCG00000015549 | RNASEL | -1.868 | 9.11E-33 | DOWN |
| ENSSSCG00000040719 | KIAA0040 | -1.26155 | 1.76E-31 | DOWN |
| ENSSSCG00000039751 | NLRC5 | -3.00919 | 1.62E-30 | DOWN |
| ENSSSCG00000026951 | PSMB8 | -1.00229 | 4.39E-30 | DOWN |
| ENSSSCG00000011239 | NA | -2.75712 | 8.81E-30 | DOWN |
| ENSSSCG00000008648 | RSAD2 | -3.94842 | 4.36E-29 | DOWN |
| ENSSSCG00000017298 | TANC2 | -1.11561 | 5.04E-29 | DOWN |
| ENSSSCG00000013147 | FAM111B | -1.00736 | 1.03E-28 | DOWN |
| ENSSSCG00000009240 | PLAC8 | -2.27403 | 2.82E-28 | DOWN |
| ENSSSCG00000016057 | STAT1 | -1.1882 | 3.18E-28 | DOWN |
| ENSSSCG00000039568 | SNAI2 | -1.53697 | 8.30E-28 | DOWN |
| ENSSSCG00000004971 | TLE3 | -1.01175 | 1.06E-27 | DOWN |
| ENSSSCG00000025788 | ENPP4 | -1.80126 | 1.34E-27 | DOWN |
| ENSSSCG00000030801 | NA | -2.51505 | 1.58E-27 | DOWN |
| ENSSSCG00000017488 | CSF3 | -1.25097 | 1.68E-27 | DOWN |
| ENSSSCG00000003137 | PLEKHA4 | -1.52632 | 1.13E-26 | DOWN |
| ENSSSCG00000000148 | NA | -2.28114 | 1.45E-26 | DOWN |
| ENSSSCG00000023379 | UBE2L6 | -1.54677 | 1.63E-26 | DOWN |
| ENSSSCG00000010454 | IFIT5 | -1.42371 | 2.12E-26 | DOWN |
| ENSSSCG00000032652 | NA | -1.91281 | 3.20E-26 | DOWN |
| ENSSSCG00000013382 | PLEKHA7 | -2.93951 | 3.90E-26 | DOWN |
| ENSSSCG00000012853 | IRF7 | -1.73832 | 4.33E-26 | DOWN |
| ENSSSCG00000027709 | PARP9 | -1.65401 | 5.17E-26 | DOWN |
| ENSSSCG00000006919 | NA | -3.17314 | 1.30E-25 | DOWN |
| ENSSSCG00000017614 | TRIM25 | -1.17947 | 4.04E-25 | DOWN |
| ENSSSCG00000032474 | CXCL10 | -3.35368 | 4.37E-25 | DOWN |
| ENSSSCG00000012076 | MX2 | -3.0424 | 5.53E-25 | DOWN |
| ENSSSCG00000040617 | TNFAIP8 | -1.34152 | 8.75E-25 | DOWN |
| ENSSSCG00000035940 | SPSB1 | -1.33395 | 1.00E-24 | DOWN |
| ENSSSCG00000020705 | MAP3K8 | -1.02445 | 1.48E-24 | DOWN |
| ENSSSCG00000010452 | IFIT1 | -2.3699 | 2.40E-24 | DOWN |
| ENSSSCG00000030408 | DDX58 | -2.22419 | 4.09E-24 | DOWN |
| ENSSSCG00000023178 | BATF2 | -2.55499 | 7.33E-24 | DOWN |
| ENSSSCG00000022312 | RHPN2 | -2.47627 | 2.48E-23 | DOWN |
| ENSSSCG00000020906 | TNFSF10 | -3.12395 | 2.66E-23 | DOWN |
| ENSSSCG00000030548 | HERC5 | -2.17368 | 3.77E-23 | DOWN |
| ENSSSCG00000038471 | NUAK2 | -1.41564 | 3.97E-23 | DOWN |
| ENSSSCG00000014618 | NA | -3.39533 | 3.07E-22 | DOWN |
| ENSSSCG00000015897 | IFIH1 | -1.43066 | 3.43E-22 | DOWN |
| ENSSSCG00000000728 | PARP11 | -1.10408 | 3.67E-22 | DOWN |
| ENSSSCG00000024914 | NA | -1.30134 | 4.92E-22 | DOWN |
| ENSSSCG00000015324 | GNG11 | -1.0141 | 1.09E-21 | DOWN |
| ENSSSCG00000031321 | NR4A1 | -1.87611 | 1.21E-21 | DOWN |
| ENSSSCG00000033222 | TRIM14 | -1.19744 | 1.43E-21 | DOWN |
| ENSSSCG00000028056 | ZFP36 | -1.11301 | 1.75E-21 | DOWN |
| ENSSSCG00000023630 | CPM | -1.14871 | 2.85E-21 | DOWN |
| ENSSSCG00000005211 | CD274 | -3.05063 | 3.82E-21 | DOWN |
| ENSSSCG00000035420 | HES4 | -2.70416 | 4.57E-21 | DOWN |
| ENSSSCG00000032436 | NA | -1.14174 | 4.67E-21 | DOWN |
| ENSSSCG00000017754 | NA | -2.36162 | 1.02E-20 | DOWN |
| ENSSSCG00000010224 | EGR2 | -1.90403 | 1.12E-20 | DOWN |
| ENSSSCG00000011495 | PRICKLE2 | -1.8693 | 2.17E-20 | DOWN |
| ENSSSCG00000017146 | RNF213 | -1.43193 | 2.94E-20 | DOWN |
| ENSSSCG00000037241 | RGS2 | -1.08916 | 2.95E-20 | DOWN |
| ENSSSCG00000012077 | MX1 | -1.62094 | 3.33E-20 | DOWN |
| ENSSSCG00000034802 | NA | -1.24417 | 4.83E-20 | DOWN |
| ENSSSCG00000038867 | PPM1K | -1.19674 | 7.31E-20 | DOWN |
| ENSSSCG00000009542 | TNFSF13B | -3.25428 | 8.24E-20 | DOWN |
| ENSSSCG00000001561 | ETV7 | -2.51477 | 1.05E-19 | DOWN |
| ENSSSCG00000010451 | IFIT2 | -2.27593 | 1.17E-19 | DOWN |
| ENSSSCG00000008496 | EIF2AK2 | -1.12764 | 1.48E-19 | DOWN |
| ENSSSCG00000008334 | MXD1 | -1.48868 | 4.27E-19 | DOWN |
| ENSSSCG00000025856 | TMEM106A | -1.0087 | 4.86E-19 | DOWN |
| ENSSSCG00000029507 | RASGEF1B | -1.82921 | 5.26E-19 | DOWN |
| ENSSSCG00000040010 | BCL2A1 | -1.161 | 1.20E-18 | DOWN |
| ENSSSCG00000016554 | MEST | -1.07895 | 1.29E-18 | DOWN |
| ENSSSCG00000000774 | USP18 | -1.89281 | 1.29E-18 | DOWN |
| ENSSSCG00000032475 | CEP44 | -1.17653 | 1.42E-18 | DOWN |
| ENSSSCG00000011876 | DTX3L | -2.15807 | 1.99E-18 | DOWN |
| ENSSSCG00000012828 | STARD8 | -1.00059 | 4.13E-18 | DOWN |
| ENSSSCG00000013753 | IER2 | -1.10411 | 5.53E-18 | DOWN |
| ENSSSCG00000036340 | ZBTB5 | -1.69915 | 5.61E-18 | DOWN |
| ENSSSCG00000001912 | PML | -1.04711 | 8.75E-18 | DOWN |
| ENSSSCG00000007508 | ZBP1 | -2.03172 | 1.38E-17 | DOWN |
| ENSSSCG00000017416 | DHX58 | -1.53807 | 1.69E-17 | DOWN |
| ENSSSCG00000027855 | SOCS1 | -1.95517 | 4.13E-17 | DOWN |
| ENSSSCG00000009720 | DDX60 | -1.9827 | 1.35E-16 | DOWN |
| ENSSSCG00000012375 | DLG3 | -2.17026 | 3.00E-16 | DOWN |
| ENSSSCG00000021712 | HERC6 | -1.59248 | 3.13E-16 | DOWN |
| ENSSSCG00000006940 | CYR61 | -1.14625 | 6.59E-16 | DOWN |
| ENSSSCG00000036383 | LGALS3BP | -1.40967 | 1.83E-15 | DOWN |
| ENSSSCG00000009881 | OAS2 | -1.61646 | 7.07E-15 | DOWN |
| ENSSSCG00000033453 | BST2 | -1.64772 | 9.79E-15 | DOWN |
| ENSSSCG00000040575 | ISG15 | -1.84399 | 2.11E-14 | DOWN |
| ENSSSCG00000017886 | FBXO39 | -1.66174 | 2.69E-14 | DOWN |
| ENSSSCG00000016053 | NA | -1.21296 | 3.52E-14 | DOWN |
| ENSSSCG00000013307 | LMO2 | -1.29195 | 6.31E-14 | DOWN |
| ENSSSCG00000035240 | GPR63 | -2.77268 | 6.44E-14 | DOWN |
| ENSSSCG00000033909 | NA | -1.06161 | 1.48E-13 | DOWN |
| ENSSSCG00000025836 | SULT1C4 | -1.60487 | 3.46E-13 | DOWN |
| ENSSSCG00000040673 | TMEM140 | -2.19463 | 4.71E-13 | DOWN |
| ENSSSCG00000008647 | CMPK2 | -1.94231 | 6.54E-13 | DOWN |
| ENSSSCG00000027660 | IFI44L | -1.09726 | 1.03E-12 | DOWN |
| ENSSSCG00000014336 | EGR1 | -1.31641 | 1.08E-12 | DOWN |
| ENSSSCG00000001787 | IL16 | -1.17548 | 3.50E-12 | DOWN |
| ENSSSCG00000006987 | SLC7A2 | -1.18584 | 5.14E-12 | DOWN |
| ENSSSCG00000039950 | RMI1 | -1.05457 | 5.23E-12 | DOWN |
| ENSSSCG00000006247 | PLAG1 | -1.43412 | 1.50E-11 | DOWN |
| ENSSSCG00000013664 | C19orf66 | -1.02236 | 4.87E-11 | DOWN |
| ENSSSCG00000016018 | FRZB | -1.48881 | 1.19E-10 | DOWN |
| ENSSSCG00000033787 | NA | -2.13525 | 1.81E-10 | DOWN |
| ENSSSCG00000005269 | TRPM6 | -1.14907 | 2.49E-10 | DOWN |
| ENSSSCG00000003763 | IFI44 | -1.04983 | 2.66E-10 | DOWN |
| ENSSSCG00000034989 | LRRTM2 | -1.42719 | 2.93E-10 | DOWN |
| ENSSSCG00000001064 | GMPR | -1.38297 | 5.12E-10 | DOWN |
| ENSSSCG00000034570 | IFI6 | -1.25379 | 1.96E-09 | DOWN |
| ENSSSCG00000036742 | KLF15 | -1.61121 | 2.98E-09 | DOWN |
| ENSSSCG00000025560 | PGLYRP2 | -1.00139 | 5.12E-09 | DOWN |
| ENSSSCG00000036064 | CALHM6 | -1.98925 | 2.88E-08 | DOWN |
| ENSSSCG00000028711 | CASP1 | -2.82388 | 3.57E-08 | DOWN |
| ENSSSCG00000026592 | TLR6 | -1.80726 | 4.45E-08 | DOWN |
| ENSSSCG00000032710 | E2F2 | -1.5519 | 5.99E-08 | DOWN |
| ENSSSCG00000014672 | NA | -1.56682 | 8.13E-08 | DOWN |
| ENSSSCG00000038149 | KCNE4 | -1.06108 | 1.82E-07 | DOWN |
| ENSSSCG00000026729 | TMEM150C | -1.60536 | 2.33E-07 | DOWN |
| ENSSSCG00000005268 | RORB | -1.1001 | 2.68E-07 | DOWN |
| ENSSSCG00000011391 | CDHR4 | -2.13324 | 4.99E-07 | DOWN |
| ENSSSCG00000038141 | TICAM2 | -1.14853 | 5.32E-07 | DOWN |
| ENSSSCG00000009921 | OASL | -3.02975 | 7.87E-07 | DOWN |
| ENSSSCG00000000773 | TUBA8 | -1.40493 | 1.38E-06 | DOWN |
| ENSSSCG00000022773 | NA | -1.14016 | 1.39E-06 | DOWN |
| ENSSSCG00000013311 | KIAA1549L | -1.04075 | 1.61E-06 | DOWN |
| ENSSSCG00000000623 | BCL2L14 | -2.57417 | 1.63E-06 | DOWN |
| ENSSSCG00000039300 | IL27 | -3.29876 | 2.64E-06 | DOWN |
| ENSSSCG00000032149 | PLET1 | -1.03582 | 2.65E-06 | DOWN |
| ENSSSCG00000036693 | NA | -1.29026 | 2.85E-06 | DOWN |
| ENSSSCG00000001252 | NA | -3.01568 | 6.46E-06 | DOWN |
| ENSSSCG00000031924 | NKX3-1 | -1.38592 | 7.02E-06 | DOWN |
| ENSSSCG00000008978 | CXCL11 | -4.0687 | 7.58E-06 | DOWN |
| ENSSSCG00000032561 | PDCD1LG2 | -6.2906 | 9.15E-06 | DOWN |
| ENSSSCG00000026943 | MRAP2 | -2.08958 | 1.11E-05 | DOWN |
| ENSSSCG00000005994 | SNTB1 | -1.23548 | 1.16E-05 | DOWN |
| ENSSSCG00000018015 | DNAH9 | -2.36012 | 1.36E-05 | DOWN |
| ENSSSCG00000035598 | EDN1 | -1.59706 | 1.49E-05 | DOWN |
| ENSSSCG00000016868 | NA | -1.57764 | 1.93E-05 | DOWN |
| ENSSSCG00000005511 | TRAF1 | -1.02486 | 1.95E-05 | DOWN |
| ENSSSCG00000035297 | ISG12(A) | -1.20193 | 3.04E-05 | DOWN |
| ENSSSCG00000010504 | BLNK | -3.08614 | 3.35E-05 | DOWN |
| ENSSSCG00000034741 | HOXD11 | -1.22514 | 3.84E-05 | DOWN |
| ENSSSCG00000009410 | RUBCNL | -1.87838 | 3.98E-05 | DOWN |
| ENSSSCG00000009169 | SLC39A8 | -1.02954 | 4.71E-05 | DOWN |
| ENSSSCG00000017466 | CCR7 | -2.04293 | 5.53E-05 | DOWN |
| ENSSSCG00000040017 | NKX2-2 | -1.37839 | 0.00011 | DOWN |
| ENSSSCG00000001231 | NA | -1.11133 | 0.000115 | DOWN |
| ENSSSCG00000003702 | GATA6 | -1.01802 | 0.000156 | DOWN |
| ENSSSCG00000024867 | ISG20 | -5.48478 | 0.000162 | DOWN |
| ENSSSCG00000001042 | MAK | -1.16921 | 0.000168 | DOWN |
| ENSSSCG00000029675 | MMP8 | -1.00712 | 0.000217 | DOWN |
| ENSSSCG00000037420 | TMEM170B | -1.05562 | 0.000228 | DOWN |
| ENSSSCG00000015979 | HOXD13 | -1.99983 | 0.000233 | DOWN |
| ENSSSCG00000022256 | C10orf10 | -1.29604 | 0.000253 | DOWN |
| ENSSSCG00000005163 | IFNB1 | -1.17984 | 0.000357 | DOWN |
| ENSSSCG00000005967 | FAM84B | -1.46037 | 0.000447 | DOWN |
| ENSSSCG00000008124 | NEURL3 | -4.54009 | 0.000591 | DOWN |
| ENSSSCG00000015584 | PROX1 | -1.14364 | 0.000741 | DOWN |
| ENSSSCG00000009132 | ENPEP | -1.43513 | 0.001027 | DOWN |
| ENSSSCG00000011630 | ACKR4 | -1.06624 | 0.001212 | DOWN |
| ENSSSCG00000002988 | NA | -1.12078 | 0.001468 | DOWN |
| ENSSSCG00000012132 | ASB9 | -1.36747 | 0.001547 | DOWN |
| ENSSSCG00000024344 | CCR5 | -1.95246 | 0.001689 | DOWN |
| ENSSSCG00000034708 | PITX2 | -3.45617 | 0.001809 | DOWN |
| ENSSSCG00000009859 | NA | -2.45406 | 0.002261 | DOWN |
| ENSSSCG00000032686 | RUNX3 | -1.59662 | 0.002622 | DOWN |
| ENSSSCG00000035521 | KLHL38 | -4.8912 | 0.003011 | DOWN |
| ENSSSCG00000014321 | KLHL3 | -1.59487 | 0.004391 | DOWN |
| ENSSSCG00000040830 | NA | -1.32295 | 0.006465 | DOWN |
| ENSSSCG00000006418 | NA | -4.22294 | 0.009167 | DOWN |
| ENSSSCG00000016164 | IKZF2 | -1.20682 | 0.011189 | DOWN |
| ENSSSCG00000023791 | TMEM229B | -2.47538 | 0.012731 | DOWN |
| ENSSSCG00000033392 | SCML4 | -1.72287 | 0.017694 | DOWN |
| ENSSSCG00000015798 | ANKRD37 | -1.27033 | 0.018003 | DOWN |
| ENSSSCG00000009051 | IL15 | -3.6852 | 0.018258 | DOWN |
| ENSSSCG00000026583 | TLR1 | -3.26897 | 0.019995 | DOWN |
| ENSSSCG00000023785 | TMEM156 | -3.0313 | 0.020862 | DOWN |
| ENSSSCG00000031897 | GIMAP7 | -1.63227 | 0.02167 | DOWN |
| ENSSSCG00000016832 | IL7R | -2.36998 | 0.025356 | DOWN |
| ENSSSCG00000031787 | NA | -1.34325 | 0.026013 | DOWN |
| ENSSSCG00000034184 | NA | -1.12359 | 0.026849 | DOWN |
| ENSSSCG00000007499 | TFAP2C | -1.18403 | 0.044443 | DOWN |
| ENSSSCG00000016256 | SPHKAP | -1.20037 | 0.048236 | DOWN |

Gene Name “NA” indicates the gene ID was not matched to a HGNC gene name.
