# Supplementary material for: A Tale of Two Loads: Modulation of IL-1 Induced Inflammatory Responses of Meniscal Cells in Two Models of Dynamic Physiologic Loading
Source: Front Bioeng Biotechnol. 2022 Mar 1;10:837619. doi: 10.3389/fbioe.2022.837619 (PMC8921261; doi:10.3389/fbioe.2022.837619)
Supplement: Supplementary file 14 [file DataSheet15.DOCX]

**Supplemental Table 16**: Genes with a significant (p<0.05) interaction effect between load (10% dynamic compression) and IL-1α treatment, inner zone tissue.

| **Gene ID** | **Gene Name** | **p-value** |
| --- | --- | --- |
| ENSSSCG00000034802 | RTP4 | 0.001516 |
| ENSSSCG00000023618 | FRMD7 | 0.001516 |
| ENSSSCG00000004050 | WTAP | 0.005877 |
| ENSSSCG00000039986 | RGS8 | 0.005877 |
| ENSSSCG00000020970 | IL6 | 0.005877 |
| ENSSSCG00000004192 | CTGF | 0.023102 |
| ENSSSCG00000014011 | RASGEF1C | 0.023102 |
| ENSSSCG00000006689 | PIAS3 | 0.02588 |
| ENSSSCG00000025858 | ELN | 0.026712 |
| ENSSSCG00000034288 | AP4M1 | 0.030463 |
| ENSSSCG00000009129 | TIFA | 0.036978 |
| ENSSSCG00000015550 | RGS16 | 0.037078 |
| ENSSSCG00000010457 | KIF20B | 0.037078 |
| ENSSSCG00000010055 | GGT5 | 0.037078 |
| ENSSSCG00000011643 | AMOTL2 | 0.039418 |
