# Supplementary material for: A Tale of Two Loads: Modulation of IL-1 Induced Inflammatory Responses of Meniscal Cells in Two Models of Dynamic Physiologic Loading
Source: Front Bioeng Biotechnol. 2022 Mar 1;10:837619. doi: 10.3389/fbioe.2022.837619 (PMC8921261; doi:10.3389/fbioe.2022.837619)
Supplement: Supplementary file 15 [file DataSheet12.DOCX]

**Supplemental Table 13**: 10% compression compared to 0% compression for outer zone tissue with exogenous IL-1α stimulation.

| **Gene ID** | **Gene Name** | **Log2Fold Change** | **p-value** | **Up/Down Regulated** |
| --- | --- | --- | --- | --- |
| ENSSSCG00000034114 | GPR68 | 1.519752 | 0.012796 | UP |
| ENSSSCG00000034167 | SLC5A3 | 1.511736 | 0.012796 | UP |
| ENSSSCG00000000749 | SLC6A12 | 1.87065 | 0.018547 | UP |
| ENSSSCG00000001695 | VEGFA | 1.124665 | 0.026533 | UP |
| ENSSSCG00000013427 | CIRBP | -1.41156 | 8.29E-07 | DOWN |
| ENSSSCG00000023871 | NA | -2.16978 | 1.87E-06 | DOWN |
| ENSSSCG00000025876 | PBLD | -1.20658 | 0.000941 | DOWN |
| ENSSSCG00000035581 | SUGCT | -1.1371 | 0.004528 | DOWN |
| ENSSSCG00000034308 | LRMDA | -1.40368 | 0.016341 | DOWN |
| ENSSSCG00000025106 | NA | -1.11929 | 0.018547 | DOWN |
| ENSSSCG00000034351 | B3GALT1 | -1.53795 | 0.021126 | DOWN |
| ENSSSCG00000035887 | C8orf34 | -1.72783 | 0.026533 | DOWN |
| ENSSSCG00000038542 | NCBP2-AS2 | -1.00945 | 0.043641 | DOWN |

Gene Name “NA” indicates the gene ID was not matched to a HGNC gene name.
