# Supplementary material for: A Tale of Two Loads: Modulation of IL-1 Induced Inflammatory Responses of Meniscal Cells in Two Models of Dynamic Physiologic Loading
Source: Front Bioeng Biotechnol. 2022 Mar 1;10:837619. doi: 10.3389/fbioe.2022.837619 (PMC8921261; doi:10.3389/fbioe.2022.837619)
Supplement: Supplementary file 16 [file DataSheet11.DOCX]

**Supplemental Table 12**: 10% compression compared to 0% compression for outer zone tissue without exogenous IL-1α stimulation.

| **Gene ID** | **Gene Name** | **Log2Fold Change** | **p-value** | **Up/Down Regulated** |
| --- | --- | --- | --- | --- |
| ENSSSCG00000011000 | DNAJA1 | 1.077782 | 6.69E-05 | UP |
| ENSSSCG00000015579 | PTGS2 | 2.243237 | 0.001095 | UP |
| ENSSSCG00000040608 | AKR1B1 | 1.423546 | 0.001931 | UP |
| ENSSSCG00000015965 | GPR155 | 1.749735 | 0.014006 | UP |
| ENSSSCG00000004241 | GJA1 | 1.304596 | 0.014419 | UP |
| ENSSSCG00000000774 | USP18 | 1.343951 | 0.022033 | UP |
| ENSSSCG00000039434 | NA | 1.092731 | 0.025877 | UP |
| ENSSSCG00000003763 | IFI44 | 1.020683 | 0.032611 | UP |
| ENSSSCG00000015590 | FLVCR1 | 1.065791 | 0.032737 | UP |
| ENSSSCG00000002135 | PNP | 1.111649 | 0.036043 | UP |
| ENSSSCG00000037572 | EPSTI1 | 1.214905 | 0.036143 | UP |
| ENSSSCG00000034167 | SLC5A3 | 1.156462 | 0.037414 | UP |
| ENSSSCG00000013313 | PRRG4 | 1.436377 | 0.038395 | UP |
| ENSSSCG00000020970 | IL6 | 2.21698 | 0.04173 | UP |
| ENSSSCG00000035421 | ATP8A2 | -3.9473 | 0.000152 | DOWN |
| ENSSSCG00000023871 | NA | -1.60115 | 0.000305 | DOWN |
| ENSSSCG00000010325 | KCNMA1 | -1.36995 | 0.000576 | DOWN |
| ENSSSCG00000024088 | TLN2 | -1.51864 | 0.001057 | DOWN |
| ENSSSCG00000027505 | ESYT3 | -1.76696 | 0.001454 | DOWN |
| ENSSSCG00000037416 | CLIC5 | -3.18753 | 0.001454 | DOWN |
| ENSSSCG00000031141 | ABCA13 | -1.88625 | 0.00172 | DOWN |
| ENSSSCG00000004281 | KCNQ5 | -1.91421 | 0.001931 | DOWN |
| ENSSSCG00000015399 | SEMA3E | -2.69648 | 0.001931 | DOWN |
| ENSSSCG00000016943 | ADAMTS6 | -1.12247 | 0.002396 | DOWN |
| ENSSSCG00000034191 | SOX6 | -1.0624 | 0.002457 | DOWN |
| ENSSSCG00000027415 | WWOX | -1.43522 | 0.002547 | DOWN |
| ENSSSCG00000016703 | HOXA5 | -1.18206 | 0.002694 | DOWN |
| ENSSSCG00000015618 | LAMB3 | -3.29734 | 0.00271 | DOWN |
| ENSSSCG00000016690 | CREB5 | -1.89499 | 0.00294 | DOWN |
| ENSSSCG00000009283 | TNFRSF19 | -2.23277 | 0.00294 | DOWN |
| ENSSSCG00000016093 | NA | -2.24421 | 0.003018 | DOWN |
| ENSSSCG00000024954 | FGF1 | -2.86954 | 0.003018 | DOWN |
| ENSSSCG00000025588 | FJX1 | -1.48185 | 0.00308 | DOWN |
| ENSSSCG00000025106 | NA | -1.08523 | 0.003512 | DOWN |
| ENSSSCG00000029438 | SESN2 | -1.07096 | 0.003598 | DOWN |
| ENSSSCG00000026818 | CTC1 | -1.25094 | 0.003633 | DOWN |
| ENSSSCG00000035371 | NA | -1.55195 | 0.003633 | DOWN |
| ENSSSCG00000031940 | GAS2 | -1.59484 | 0.003633 | DOWN |
| ENSSSCG00000017383 | NA | -4.82132 | 0.003633 | DOWN |
| ENSSSCG00000001807 | AP3B2 | -2.8251 | 0.003692 | DOWN |
| ENSSSCG00000026259 | LINGO4 | -6.0423 | 0.00397 | DOWN |
| ENSSSCG00000035867 | GFOD1 | -1.7175 | 0.00415 | DOWN |
| ENSSSCG00000028076 | ZBTB7C | -1.30497 | 0.004454 | DOWN |
| ENSSSCG00000036772 | CAMTA1 | -2.5624 | 0.004454 | DOWN |
| ENSSSCG00000039364 | NA | -3.45458 | 0.004629 | DOWN |
| ENSSSCG00000007456 | SULF2 | -1.63715 | 0.004943 | DOWN |
| ENSSSCG00000015700 | TMEM163 | -3.20171 | 0.004943 | DOWN |
| ENSSSCG00000010142 | RYR2 | -3.6134 | 0.004943 | DOWN |
| ENSSSCG00000017054 | CYFIP2 | -1.61777 | 0.00613 | DOWN |
| ENSSSCG00000009489 | NA | -3.6483 | 0.006305 | DOWN |
| ENSSSCG00000008312 | DYSF | -2.55637 | 0.00639 | DOWN |
| ENSSSCG00000003592 | SDC3 | -1.89925 | 0.006432 | DOWN |
| ENSSSCG00000030681 | MYBPH | -3.25376 | 0.006815 | DOWN |
| ENSSSCG00000006344 | NOS1AP | -1.85213 | 0.007283 | DOWN |
| ENSSSCG00000031780 | MAGI2 | -1.63697 | 0.007285 | DOWN |
| ENSSSCG00000035887 | C8orf34 | -1.64538 | 0.007308 | DOWN |
| ENSSSCG00000022988 | TSPEAR | -5.779 | 0.00757 | DOWN |
| ENSSSCG00000014909 | NA | -2.18138 | 0.007686 | DOWN |
| ENSSSCG00000012362 | ARHGEF9 | -1.16191 | 0.007748 | DOWN |
| ENSSSCG00000020717 | FAM160A1 | -1.4895 | 0.007973 | DOWN |
| ENSSSCG00000010276 | UNC5B | -1.85986 | 0.008127 | DOWN |
| ENSSSCG00000004896 | PHLPP1 | -1.22248 | 0.008378 | DOWN |
| ENSSSCG00000040875 | ZFPM1 | -1.48278 | 0.008658 | DOWN |
| ENSSSCG00000011384 | BSN | -1.63491 | 0.008658 | DOWN |
| ENSSSCG00000035223 | SYNM | -1.04121 | 0.008752 | DOWN |
| ENSSSCG00000016338 | PER2 | -1.01866 | 0.008938 | DOWN |
| ENSSSCG00000004856 | NFATC1 | -1.04047 | 0.009156 | DOWN |
| ENSSSCG00000023403 | NA | -3.49529 | 0.009259 | DOWN |
| ENSSSCG00000030998 | WIF1 | -4.54026 | 0.00989 | DOWN |
| ENSSSCG00000034308 | LRMDA | -1.30717 | 0.010216 | DOWN |
| ENSSSCG00000017296 | ACE | -3.06716 | 0.010526 | DOWN |
| ENSSSCG00000033341 | NA | -2.11872 | 0.010531 | DOWN |
| ENSSSCG00000029744 | PLCH2 | -3.86007 | 0.010732 | DOWN |
| ENSSSCG00000024517 | AKAP6 | -1.32692 | 0.011091 | DOWN |
| ENSSSCG00000007485 | BCAS1 | -2.60766 | 0.011091 | DOWN |
| ENSSSCG00000026943 | MRAP2 | -4.07688 | 0.011091 | DOWN |
| ENSSSCG00000022554 | MATN1 | -5.83567 | 0.011091 | DOWN |
| ENSSSCG00000033234 | SSTR5 | -6.49527 | 0.012208 | DOWN |
| ENSSSCG00000007872 | XYLT1 | -1.69081 | 0.012568 | DOWN |
| ENSSSCG00000039890 | RASL11A | -2.81594 | 0.012575 | DOWN |
| ENSSSCG00000008984 | SHROOM3 | -3.77625 | 0.012575 | DOWN |
| ENSSSCG00000024873 | PCDHB6 | -1.0087 | 0.012807 | DOWN |
| ENSSSCG00000013294 | LDLRAD3 | -1.84469 | 0.012807 | DOWN |
| ENSSSCG00000013403 | GALNT18 | -1.63431 | 0.013266 | DOWN |
| ENSSSCG00000031244 | GAP43 | -1.7564 | 0.013266 | DOWN |
| ENSSSCG00000014581 | TUB | -1.13939 | 0.013293 | DOWN |
| ENSSSCG00000004332 | BACH2 | -1.57788 | 0.013602 | DOWN |
| ENSSSCG00000017561 | ABCC3 | -1.52989 | 0.013858 | DOWN |
| ENSSSCG00000033412 | B4GALNT3 | -1.58047 | 0.014006 | DOWN |
| ENSSSCG00000014011 | RASGEF1C | -2.06748 | 0.014541 | DOWN |
| ENSSSCG00000036933 | NR1D1 | -1.39367 | 0.015112 | DOWN |
| ENSSSCG00000004980 | THSD4 | -2.29252 | 0.015415 | DOWN |
| ENSSSCG00000034181 | NKX3-2 | -3.45404 | 0.015415 | DOWN |
| ENSSSCG00000017498 | PPP1R1B | -5.13539 | 0.015974 | DOWN |
| ENSSSCG00000032053 | ST8SIA5 | -5.05845 | 0.01614 | DOWN |
| ENSSSCG00000017254 | MAP2K6 | -1.62016 | 0.016653 | DOWN |
| ENSSSCG00000008881 | RAPGEF2 | -1.13488 | 0.017792 | DOWN |
| ENSSSCG00000015955 | ITGA6 | -1.35161 | 0.017822 | DOWN |
| ENSSSCG00000022492 | AMPD3 | -1.04143 | 0.018401 | DOWN |
| ENSSSCG00000000029 | SCUBE1 | -3.26257 | 0.018985 | DOWN |
| ENSSSCG00000004191 | MOXD1 | -2.90657 | 0.019028 | DOWN |
| ENSSSCG00000021562 | PLXNA4 | -2.70359 | 0.019956 | DOWN |
| ENSSSCG00000038693 | RAB19 | -3.59025 | 0.020047 | DOWN |
| ENSSSCG00000011557 | CIDEC | -1.91427 | 0.020327 | DOWN |
| ENSSSCG00000012848 | EPS8L2 | -2.29401 | 0.020762 | DOWN |
| ENSSSCG00000022609 | WWP2 | -1.77863 | 0.021507 | DOWN |
| ENSSSCG00000001004 | SLC22A23 | -2.05692 | 0.021637 | DOWN |
| ENSSSCG00000031764 | NA | -1.21253 | 0.021915 | DOWN |
| ENSSSCG00000035971 | DUSP2 | -2.14192 | 0.021915 | DOWN |
| ENSSSCG00000023915 | SLC2A4 | -3.1098 | 0.022033 | DOWN |
| ENSSSCG00000017676 | BCAS3 | -1.03457 | 0.022232 | DOWN |
| ENSSSCG00000011959 | ABI3BP | -1.41778 | 0.02283 | DOWN |
| ENSSSCG00000017747 | RAB11FIP4 | -1.98762 | 0.023443 | DOWN |
| ENSSSCG00000010280 | VSIR | -1.18014 | 0.02351 | DOWN |
| ENSSSCG00000011397 | SLC38A3 | -3.2668 | 0.023829 | DOWN |
| ENSSSCG00000012852 | CDHR5 | -5.45475 | 0.023986 | DOWN |
| ENSSSCG00000016983 | STC2 | -1.28303 | 0.024268 | DOWN |
| ENSSSCG00000015211 | PKNOX2 | -1.49739 | 0.024616 | DOWN |
| ENSSSCG00000009807 | RHOF | -1.15257 | 0.024657 | DOWN |
| ENSSSCG00000005449 | PTPN3 | -1.04494 | 0.024661 | DOWN |
| ENSSSCG00000012323 | TSPYL2 | -1.00322 | 0.024721 | DOWN |
| ENSSSCG00000004422 | WISP3 | -2.70604 | 0.024885 | DOWN |
| ENSSSCG00000032532 | CHRM2 | -2.99965 | 0.024961 | DOWN |
| ENSSSCG00000025523 | COL2A1 | -2.88326 | 0.025394 | DOWN |
| ENSSSCG00000031692 | NA | -2.10102 | 0.025616 | DOWN |
| ENSSSCG00000005380 | COL15A1 | -1.36224 | 0.025811 | DOWN |
| ENSSSCG00000016611 | CADPS2 | -1.07727 | 0.025829 | DOWN |
| ENSSSCG00000005371 | HEMGN | -1.02322 | 0.025877 | DOWN |
| ENSSSCG00000022525 | ARHGEF5 | -6.05487 | 0.025877 | DOWN |
| ENSSSCG00000016381 | SNED1 | -1.61416 | 0.026287 | DOWN |
| ENSSSCG00000009338 | FRY | -1.53686 | 0.027407 | DOWN |
| ENSSSCG00000007800 | 1-Sep | -2.72933 | 0.027911 | DOWN |
| ENSSSCG00000028185 | FGD3 | -2.7606 | 0.028297 | DOWN |
| ENSSSCG00000001473 | COL11A2 | -2.64291 | 0.028406 | DOWN |
| ENSSSCG00000004387 | FOXO3 | -1.00308 | 0.028544 | DOWN |
| ENSSSCG00000027745 | ABCG1 | -2.16317 | 0.028949 | DOWN |
| ENSSSCG00000026318 | NETO2 | -2.61099 | 0.029048 | DOWN |
| ENSSSCG00000010267 | NA | -1.20978 | 0.029099 | DOWN |
| ENSSSCG00000009929 | TRPV4 | -1.5343 | 0.029934 | DOWN |
| ENSSSCG00000027002 | KLHL32 | -2.51129 | 0.030107 | DOWN |
| ENSSSCG00000033314 | DLX6 | -1.88179 | 0.03064 | DOWN |
| ENSSSCG00000023749 | MIOX | -1.90522 | 0.030715 | DOWN |
| ENSSSCG00000001964 | NPAS3 | -2.53066 | 0.03108 | DOWN |
| ENSSSCG00000034943 | GDF6 | -1.70056 | 0.031582 | DOWN |
| ENSSSCG00000000162 | BTBD11 | -1.53273 | 0.031775 | DOWN |
| ENSSSCG00000016657 | AOAH | -3.4219 | 0.032204 | DOWN |
| ENSSSCG00000005785 | PCSK6 | -1.5638 | 0.03303 | DOWN |
| ENSSSCG00000038545 | C10orf105 | -3.11549 | 0.033323 | DOWN |
| ENSSSCG00000021874 | UNC5C | -1.30923 | 0.033478 | DOWN |
| ENSSSCG00000015350 | THSD7A | -3.07618 | 0.033722 | DOWN |
| ENSSSCG00000003440 | AADACL4 | -2.95902 | 0.03374 | DOWN |
| ENSSSCG00000026427 | RORC | -1.63905 | 0.033852 | DOWN |
| ENSSSCG00000039182 | C11orf96 | -1.67108 | 0.034646 | DOWN |
| ENSSSCG00000034178 | AIF1L | -2.67854 | 0.035366 | DOWN |
| ENSSSCG00000014959 | PIWIL4 | -2.08805 | 0.036123 | DOWN |
| ENSSSCG00000031866 | TIMP3 | -1.31568 | 0.036319 | DOWN |
| ENSSSCG00000003374 | ESPN | -2.80552 | 0.03722 | DOWN |
| ENSSSCG00000013382 | PLEKHA7 | -1.85354 | 0.037414 | DOWN |
| ENSSSCG00000039245 | RIMS3 | -5.81658 | 0.037414 | DOWN |
| ENSSSCG00000002684 | CDH13 | -1.07386 | 0.037995 | DOWN |
| ENSSSCG00000014567 | TRIM66 | -1.03089 | 0.039028 | DOWN |
| ENSSSCG00000012585 | DCX | -1.2164 | 0.039028 | DOWN |
| ENSSSCG00000036446 | PALD1 | -1.38 | 0.039028 | DOWN |
| ENSSSCG00000006810 | KCNC4 | -1.59384 | 0.039058 | DOWN |
| ENSSSCG00000039317 | SLC25A21 | -1.34203 | 0.039379 | DOWN |
| ENSSSCG00000040989 | GPRC5C | -2.39405 | 0.039379 | DOWN |
| ENSSSCG00000006542 | KCNN3 | -4.06395 | 0.039379 | DOWN |
| ENSSSCG00000034681 | NA | -2.92847 | 0.039491 | DOWN |
| ENSSSCG00000017046 | EBF1 | -1.10819 | 0.039587 | DOWN |
| ENSSSCG00000005512 | C5 | -1.34245 | 0.039675 | DOWN |
| ENSSSCG00000014570 | NRIP3 | -1.89997 | 0.040396 | DOWN |
| ENSSSCG00000036261 | CROCC2 | -2.6434 | 0.040396 | DOWN |
| ENSSSCG00000027215 | CTRC | -5.18148 | 0.040396 | DOWN |
| ENSSSCG00000011582 | CAND2 | -1.08733 | 0.04173 | DOWN |
| ENSSSCG00000010522 | ANKRD2 | -1.34369 | 0.04173 | DOWN |
| ENSSSCG00000017244 | SDK2 | -1.52393 | 0.04173 | DOWN |
| ENSSSCG00000014436 | ARHGEF37 | -1.93285 | 0.04173 | DOWN |
| ENSSSCG00000014221 | LVRN | -2.47029 | 0.041843 | DOWN |
| ENSSSCG00000000716 | KCNA1 | -2.95899 | 0.041956 | DOWN |
| ENSSSCG00000023746 | NA | -5.23594 | 0.042598 | DOWN |
| ENSSSCG00000011471 | FLNB | -1.80699 | 0.042754 | DOWN |
| ENSSSCG00000010201 | RASGEF1A | -2.87636 | 0.042754 | DOWN |
| ENSSSCG00000002688 | PLCG2 | -1.45281 | 0.042968 | DOWN |
| ENSSSCG00000018712 | ssc-mir-339-1 | -2.76515 | 0.042968 | DOWN |
| ENSSSCG00000003201 | ATF5 | -1.14328 | 0.043529 | DOWN |
| ENSSSCG00000006933 | CLCA1 | -2.05475 | 0.043529 | DOWN |
| ENSSSCG00000038190 | MPST | -1.66707 | 0.045282 | DOWN |
| ENSSSCG00000033750 | NA | -1.08536 | 0.045282 | DOWN |
| ENSSSCG00000015556 | LAMC2 | -1.93794 | 0.045282 | DOWN |
| ENSSSCG00000015701 | MGAT5 | -1.19846 | 0.045282 | DOWN |
| ENSSSCG00000007052 | FERMT1 | -2.00024 | 0.045462 | DOWN |
| ENSSSCG00000027928 | TMEM9 | -1.58775 | 0.045462 | DOWN |
| ENSSSCG00000006001 | ENPP2 | -2.40421 | 0.046227 | DOWN |
| ENSSSCG00000012669 | ARHGAP36 | -1.46345 | 0.046238 | DOWN |
| ENSSSCG00000038296 | NA | -1.33631 | 0.046238 | DOWN |
| ENSSSCG00000021053 | GIPR | -4.739 | 0.046238 | DOWN |
| ENSSSCG00000037846 | ACOXL | -3.53425 | 0.046685 | DOWN |
| ENSSSCG00000003988 | NA | -2.08411 | 0.046729 | DOWN |
| ENSSSCG00000017101 | ADCY2 | -1.64755 | 0.047896 | DOWN |
| ENSSSCG00000007067 | JAG1 | -1.07468 | 0.049091 | DOWN |
| ENSSSCG00000033537 | NA | -1.62824 | 0.049379 | DOWN |

Gene Name “NA” indicates the gene ID was not matched to a HGNC gene name.
