# Supplementary material for: A Tale of Two Loads: Modulation of IL-1 Induced Inflammatory Responses of Meniscal Cells in Two Models of Dynamic Physiologic Loading
Source: Front Bioeng Biotechnol. 2022 Mar 1;10:837619. doi: 10.3389/fbioe.2022.837619 (PMC8921261; doi:10.3389/fbioe.2022.837619)
Supplement: Supplementary file 17 [file DataSheet1.DOCX]

**Supplemental Table 2**: IL-1α stimulation compared to unstimulated samples for unloaded inner zone cells.

| **Gene ID** | **Gene Name** | **Log2Fold Change** | **p-value** | **Up/Down Regulated** |
| --- | --- | --- | --- | --- |
| ENSSSCG00000008090 | IL1A | 11.61867272 | 5.2E-22 | UP |
| ENSSSCG00000009469 | ACOD1 | 10.92229471 | 2.59E-19 | UP |
| ENSSSCG00000023737 | CSF2 | 10.80913916 | 1.06E-18 | UP |
| ENSSSCG00000007007 | IDO1 | 10.68248245 | 6.93E-36 | UP |
| ENSSSCG00000016254 | CCL20 | 10.57053175 | 2.89E-81 | UP |
| ENSSSCG00000001404 | TNF | 10.51359978 | 3.63E-18 | UP |
| ENSSSCG00000017755 | NOS2 | 9.850934415 | 0 | UP |
| ENSSSCG00000034980 | IRF8 | 9.620633366 | 1.42E-19 | UP |
| ENSSSCG00000037735 | NA | 9.415597894 | 1.02E-14 | UP |
| ENSSSCG00000022405 | P2RX1 | 9.320724957 | 1.69E-14 | UP |
| ENSSSCG00000032474 | CXCL10 | 9.293013624 | 1.9E-154 | UP |
| ENSSSCG00000014987 | MMP12 | 8.927408546 | 7.43E-50 | UP |
| ENSSSCG00000032149 | PLET1 | 8.799628104 | 6.98E-13 | UP |
| ENSSSCG00000034015 | NA | 8.782693001 | 6E-13 | UP |
| ENSSSCG00000022089 | ADGRF4 | 8.679889732 | 2.49E-12 | UP |
| ENSSSCG00000033909 | NA | 8.648330514 | 1.46E-12 | UP |
| ENSSSCG00000005163 | IFNB1 | 8.574119534 | 4.48E-12 | UP |
| ENSSSCG00000020970 | IL6 | 8.382313263 | 0 | UP |
| ENSSSCG00000008953 | CXCL8 | 8.166503309 | 0 | UP |
| ENSSSCG00000013551 | C3 | 8.072980593 | 9.26E-58 | UP |
| ENSSSCG00000001252 | NA | 8.055471882 | 2.07E-10 | UP |
| ENSSSCG00000040036 | NA | 7.9245084 | 4.41E-33 | UP |
| ENSSSCG00000039214 | NA | 7.726131777 | 1.86E-09 | UP |
| ENSSSCG00000038562 | RND1 | 7.721733443 | 0 | UP |
| ENSSSCG00000004890 | SERPINB2 | 7.671210615 | 1.33E-09 | UP |
| ENSSSCG00000008954 | NA | 7.655625077 | 1.1E-244 | UP |
| ENSSSCG00000008978 | CXCL11 | 7.541013783 | 3.84E-09 | UP |
| ENSSSCG00000005504 | BRINP1 | 7.485808335 | 1.47E-28 | UP |
| ENSSSCG00000002821 | CCL22 | 7.461353099 | 2.37E-09 | UP |
| ENSSSCG00000000291 | GPR84 | 7.435213569 | 2.27E-23 | UP |
| ENSSSCG00000006987 | SLC7A2 | 7.397351308 | 0 | UP |
| ENSSSCG00000040648 | CCL11 | 7.275384688 | 5.8E-99 | UP |
| ENSSSCG00000028711 | CASP1 | 7.185641795 | 1.12E-08 | UP |
| ENSSSCG00000005211 | CD274 | 7.180051788 | 1.9E-99 | UP |
| ENSSSCG00000004195 | ARG1 | 7.054412954 | 2.55E-44 | UP |
| ENSSSCG00000024867 | ISG20 | 6.952901993 | 1.77E-07 | UP |
| ENSSSCG00000008959 | CXCL2 | 6.798832368 | 0 | UP |
| ENSSSCG00000024914 | NA | 6.757391626 | 2.42E-88 | UP |
| ENSSSCG00000008124 | NEURL3 | 6.645334528 | 8.14E-07 | UP |
| ENSSSCG00000006256 | SOX17 | 6.570999535 | 2.11E-06 | UP |
| ENSSSCG00000006919 | NA | 6.558866944 | 2.57E-83 | UP |
| ENSSSCG00000033952 | CITED4 | 6.539031467 | 1.83E-34 | UP |
| ENSSSCG00000008648 | RSAD2 | 6.437737707 | 1.09E-77 | UP |
| ENSSSCG00000009542 | TNFSF13B | 6.411248264 | 1.63E-47 | UP |
| ENSSSCG00000006418 | NA | 6.234733346 | 8.2E-06 | UP |
| ENSSSCG00000009429 | TNFSF11 | 6.227423317 | 7E-42 | UP |
| ENSSSCG00000004572 | NA | 6.194205081 | 0 | UP |
| ENSSSCG00000017488 | CSF3 | 6.112626079 | 2.79E-54 | UP |
| ENSSSCG00000007964 | NA | 6.092058544 | 3.83E-06 | UP |
| ENSSSCG00000013885 | FCHO1 | 6.014539712 | 6.71E-80 | UP |
| ENSSSCG00000012839 | NA | 6.00486409 | 1.96E-06 | UP |
| ENSSSCG00000031610 | NA | 5.963895421 | 1.91E-47 | UP |
| ENSSSCG00000031788 | PTAFR | 5.947806676 | 2.16E-05 | UP |
| ENSSSCG00000033089 | NA | 5.931167795 | 1.11E-81 | UP |
| ENSSSCG00000040961 | LIF | 5.91534656 | 0 | UP |
| ENSSSCG00000035016 | NA | 5.90632827 | 4.1E-06 | UP |
| ENSSSCG00000039300 | IL27 | 5.890351267 | 8.54E-06 | UP |
| ENSSSCG00000022083 | NA | 5.883111023 | 4.87E-06 | UP |
| ENSSSCG00000014897 | FAM181B | 5.865997253 | 7.24E-06 | UP |
| ENSSSCG00000011727 | PTX3 | 5.840634517 | 0 | UP |
| ENSSSCG00000013236 | MYBPC3 | 5.838683942 | 2.7E-146 | UP |
| ENSSSCG00000022849 | IL2RA | 5.707572502 | 1.28E-27 | UP |
| ENSSSCG00000017517 | TBX21 | 5.686052051 | 0.00014 | UP |
| ENSSSCG00000004755 | DLL4 | 5.685845549 | 5.22E-41 | UP |
| ENSSSCG00000031646 | APOC4 | 5.684880355 | 2.93E-05 | UP |
| ENSSSCG00000033507 | ASCL2 | 5.557245054 | 0.000201 | UP |
| ENSSSCG00000012960 | CST6 | 5.550804579 | 1.52E-12 | UP |
| ENSSSCG00000014985 | MMP3 | 5.51570533 | 5.2E-262 | UP |
| ENSSSCG00000009477 | EDNRB | 5.502195047 | 9.9E-16 | UP |
| ENSSSCG00000035732 | NA | 5.500881481 | 0.000121 | UP |
| ENSSSCG00000010054 | ADORA2A | 5.496949937 | 1.1E-145 | UP |
| ENSSSCG00000008957 | AMCF-II | 5.485979061 | 0 | UP |
| ENSSSCG00000027762 | TNFRSF11B | 5.468243601 | 1.6E-132 | UP |
| ENSSSCG00000011874 | PARP14 | 5.452370082 | 2E-127 | UP |
| ENSSSCG00000014618 | NA | 5.438497352 | 1.33E-26 | UP |
| ENSSSCG00000033327 | PDGFB | 5.431941841 | 9.72E-10 | UP |
| ENSSSCG00000012076 | MX2 | 5.400413406 | 9.17E-78 | UP |
| ENSSSCG00000007607 | KPNA7 | 5.389020034 | 1.28E-06 | UP |
| ENSSSCG00000016903 | GZMA | 5.336792055 | 5.5E-05 | UP |
| ENSSSCG00000016832 | IL7R | 5.333968621 | 0.000228 | UP |
| ENSSSCG00000001561 | ETV7 | 5.322155664 | 7.19E-75 | UP |
| ENSSSCG00000000749 | SLC6A12 | 5.298836092 | 6.11E-09 | UP |
| ENSSSCG00000024344 | CCR5 | 5.297795588 | 6.42E-05 | UP |
| ENSSSCG00000004332 | BACH2 | 5.265546171 | 1.38E-37 | UP |
| ENSSSCG00000017723 | CCL2 | 5.259486213 | 0 | UP |
| ENSSSCG00000010451 | IFIT2 | 5.253363609 | 4.6E-103 | UP |
| ENSSSCG00000017720 | CCL1 | 5.246277191 | 0.000201 | UP |
| ENSSSCG00000000148 | NA | 5.211380458 | 4.8E-104 | UP |
| ENSSSCG00000014198 | NA | 5.189812317 | 2.75E-06 | UP |
| ENSSSCG00000040317 | SOD2 | 5.167889075 | 0 | UP |
| ENSSSCG00000013425 | MISP | 5.16135198 | 4.51E-11 | UP |
| ENSSSCG00000017474 | GJD3 | 5.146436509 | 0.000134 | UP |
| ENSSSCG00000016959 | MARVELD2 | 5.097833049 | 1.3E-52 | UP |
| ENSSSCG00000027855 | SOCS1 | 5.07919744 | 4.1E-100 | UP |
| ENSSSCG00000017705 | CCL5 | 5.078075161 | 0 | UP |
| ENSSSCG00000030681 | MYBPH | 5.029870155 | 5.94E-08 | UP |
| ENSSSCG00000040725 | IL11 | 5.020767748 | 3.7E-254 | UP |
| ENSSSCG00000037358 | NA | 5.010680114 | 4.18E-92 | UP |
| ENSSSCG00000010212 | NA | 5.006239853 | 1E-209 | UP |
| ENSSSCG00000006359 | ADAMTS4 | 4.980962877 | 0 | UP |
| ENSSSCG00000004125 | STX11 | 4.976300633 | 0 | UP |
| ENSSSCG00000040720 | SLC26A9 | 4.964048809 | 0.000292 | UP |
| ENSSSCG00000000718 | GALNT8 | 4.955964049 | 0.000378 | UP |
| ENSSSCG00000013382 | PLEKHA7 | 4.911730164 | 2.32E-57 | UP |
| ENSSSCG00000030801 | NA | 4.892378075 | 8.83E-50 | UP |
| ENSSSCG00000003801 | IL23R | 4.885916743 | 0.000346 | UP |
| ENSSSCG00000026958 | WNT10A | 4.883948267 | 2.88E-05 | UP |
| ENSSSCG00000037132 | POU2F2 | 4.88224388 | 1.05E-11 | UP |
| ENSSSCG00000008796 | RBM47 | 4.861487259 | 5.77E-10 | UP |
| ENSSSCG00000037949 | NA | 4.858962641 | 0.000356 | UP |
| ENSSSCG00000006862 | VCAM1 | 4.85688235 | 7.3E-293 | UP |
| ENSSSCG00000004670 | C15orf48 | 4.841574159 | 7.8E-225 | UP |
| ENSSSCG00000037541 | NA | 4.836787849 | 0.000564 | UP |
| ENSSSCG00000000623 | BCL2L14 | 4.831763865 | 3.72E-09 | UP |
| ENSSSCG00000011570 | IRAK2 | 4.813873431 | 0 | UP |
| ENSSSCG00000026701 | NA | 4.813867363 | 8.47E-51 | UP |
| ENSSSCG00000033750 | NA | 4.809377601 | 9.7E-279 | UP |
| ENSSSCG00000036064 | CALHM6 | 4.795021098 | 1.82E-18 | UP |
| ENSSSCG00000023630 | CPM | 4.791373504 | 1.2E-159 | UP |
| ENSSSCG00000015299 | STEAP4 | 4.742815003 | 1.13E-34 | UP |
| ENSSSCG00000004154 | TNFAIP3 | 4.737317655 | 0 | UP |
| ENSSSCG00000005472 | SLC46A2 | 4.68301546 | 1.06E-19 | UP |
| ENSSSCG00000017921 | ZMYND15 | 4.664710973 | 1.5E-16 | UP |
| ENSSSCG00000004369 | PRDM1 | 4.6574503 | 3.71E-72 | UP |
| ENSSSCG00000005439 | ACTL7A | 4.651481338 | 0.000639 | UP |
| ENSSSCG00000028816 | NA | 4.63651341 | 0.001216 | UP |
| ENSSSCG00000003737 | NOL4 | 4.635478627 | 0.000434 | UP |
| ENSSSCG00000000647 | OLR1 | 4.633293905 | 0.001985 | UP |
| ENSSSCG00000015476 | CHI3L1 | 4.631125816 | 1.69E-21 | UP |
| ENSSSCG00000006288 | SELP | 4.623078168 | 0.000369 | UP |
| ENSSSCG00000024973 | NA | 4.610070657 | 6.82E-98 | UP |
| ENSSSCG00000031649 | RNASE1 | 4.589878778 | 0.001302 | UP |
| ENSSSCG00000023178 | BATF2 | 4.587852498 | 3.2E-69 | UP |
| ENSSSCG00000002501 | NA | 4.570319633 | 0 | UP |
| ENSSSCG00000009002 | TLR2 | 4.534103927 | 1.3E-178 | UP |
| ENSSSCG00000013369 | NA | 4.487592451 | 4.1E-115 | UP |
| ENSSSCG00000027405 | U2 | 4.484329465 | 0.000811 | UP |
| ENSSSCG00000010452 | IFIT1 | 4.472294502 | 1.82E-86 | UP |
| ENSSSCG00000008647 | CMPK2 | 4.451085065 | 3.66E-66 | UP |
| ENSSSCG00000024759 | CX3CL1 | 4.446492297 | 6.1E-281 | UP |
| ENSSSCG00000005688 | PTGES | 4.444457651 | 0 | UP |
| ENSSSCG00000024022 | TRPC6 | 4.440809594 | 2.1E-44 | UP |
| ENSSSCG00000023796 | NA | 4.440064597 | 1.33E-37 | UP |
| ENSSSCG00000035037 | NA | 4.433113232 | 1.8E-210 | UP |
| ENSSSCG00000029284 | NPHS1 | 4.424065773 | 0.000198 | UP |
| ENSSSCG00000008742 | CD38 | 4.399378615 | 2.12E-11 | UP |
| ENSSSCG00000016519 | AKR1D1 | 4.383017957 | 7.5E-110 | UP |
| ENSSSCG00000008664 | FAM84A | 4.376558016 | 2.36E-43 | UP |
| ENSSSCG00000006398 | SLAMF8 | 4.364608559 | 2.14E-09 | UP |
| ENSSSCG00000006187 | MSC | 4.355893294 | 2.3E-156 | UP |
| ENSSSCG00000030408 | DDX58 | 4.352358467 | 1.95E-92 | UP |
| ENSSSCG00000014277 | IRF1 | 4.332270163 | 1.2E-259 | UP |
| ENSSSCG00000016255 | DAW1 | 4.296093702 | 5.92E-14 | UP |
| ENSSSCG00000039751 | NLRC5 | 4.290298914 | 1.23E-60 | UP |
| ENSSSCG00000016030 | ZSWIM2 | 4.282342478 | 0.002085 | UP |
| ENSSSCG00000038185 | EREG | 4.272993646 | 1.2E-234 | UP |
| ENSSSCG00000017645 | TEX14 | 4.23986387 | 2.54E-41 | UP |
| ENSSSCG00000003451 | NA | 4.211662655 | 0 | UP |
| ENSSSCG00000008213 | CD8B | 4.208294505 | 0.004158 | UP |
| ENSSSCG00000006286 | NA | 4.20501931 | 1.94E-07 | UP |
| ENSSSCG00000032532 | CHRM2 | 4.202072556 | 7.4E-195 | UP |
| ENSSSCG00000024166 | SLC2A6 | 4.196731897 | 0 | UP |
| ENSSSCG00000012132 | ASB9 | 4.181319968 | 8.23E-12 | UP |
| ENSSSCG00000031380 | NA | 4.134081588 | 0 | UP |
| ENSSSCG00000033520 | IL23A | 4.12299724 | 3.36E-07 | UP |
| ENSSSCG00000033286 | NA | 4.105512983 | 3.57E-08 | UP |
| ENSSSCG00000023522 | TGM2 | 4.100712111 | 0 | UP |
| ENSSSCG00000017700 | CCL3L1 | 4.098784833 | 5.56E-43 | UP |
| ENSSSCG00000007899 | NA | 4.068411693 | 0.002974 | UP |
| ENSSSCG00000016022 | ZNF804A | 4.068378611 | 1.61E-05 | UP |
| ENSSSCG00000015715 | EN1 | 4.067750675 | 1.31E-18 | UP |
| ENSSSCG00000040575 | ISG15 | 4.053789422 | 1.06E-68 | UP |
| ENSSSCG00000023374 | SRGN | 4.048030505 | 1.61E-05 | UP |
| ENSSSCG00000015579 | PTGS2 | 4.043743359 | 0 | UP |
| ENSSSCG00000023716 | TNFAIP6 | 4.037336066 | 0 | UP |
| ENSSSCG00000007501 | BMP7 | 4.029419912 | 0.001023 | UP |
| ENSSSCG00000001463 | PSMB9 | 4.014539733 | 3.2E-163 | UP |
| ENSSSCG00000033015 | B3GNT7 | 4.00174825 | 1.5E-298 | UP |
| ENSSSCG00000009125 | ANK2 | 3.99599545 | 0 | UP |
| ENSSSCG00000003006 | CYP2B6 | 3.978678722 | 0.004266 | UP |
| ENSSSCG00000040904 | CLDN1 | 3.959854256 | 1.7E-153 | UP |
| ENSSSCG00000020906 | TNFSF10 | 3.955056348 | 3.29E-36 | UP |
| ENSSSCG00000031924 | NKX3-1 | 3.942599755 | 4.8E-31 | UP |
| ENSSSCG00000015784 | ACSL1 | 3.929504039 | 0 | UP |
| ENSSSCG00000012066 | KCNJ15 | 3.926609051 | 1.1E-21 | UP |
| ENSSSCG00000013296 | SLC1A2 | 3.922953356 | 3.88E-43 | UP |
| ENSSSCG00000031970 | RASSF5 | 3.921819816 | 2.7E-280 | UP |
| ENSSSCG00000031579 | NA | 3.91367058 | 0 | UP |
| ENSSSCG00000037815 | ZC3H12A | 3.908722751 | 0 | UP |
| ENSSSCG00000039442 | BMP2 | 3.907360706 | 5.9E-154 | UP |
| ENSSSCG00000014924 | CTSC | 3.905323898 | 0 | UP |
| ENSSSCG00000015959 | RAPGEF4 | 3.903553394 | 2.93E-38 | UP |
| ENSSSCG00000001764 | SH2D7 | 3.901779353 | 6.35E-15 | UP |
| ENSSSCG00000000657 | NA | 3.88845066 | 0.000119 | UP |
| ENSSSCG00000006455 | NA | 3.884523848 | 0.01421 | UP |
| ENSSSCG00000030228 | PIWIL2 | 3.882989101 | 3.5E-08 | UP |
| ENSSSCG00000007508 | ZBP1 | 3.867019562 | 8.47E-62 | UP |
| ENSSSCG00000013890 | SLC5A5 | 3.86521786 | 1.99E-09 | UP |
| ENSSSCG00000023591 | ADGRF2 | 3.823945102 | 1.42E-05 | UP |
| ENSSSCG00000030548 | HERC5 | 3.808074963 | 1.55E-69 | UP |
| ENSSSCG00000016900 | ESM1 | 3.804468927 | 1.9E-148 | UP |
| ENSSSCG00000011876 | DTX3L | 3.794288646 | 1.84E-55 | UP |
| ENSSSCG00000021515 | HS3ST1 | 3.793747331 | 0.000285 | UP |
| ENSSSCG00000039862 | TRIB3 | 3.792719785 | 9.9E-262 | UP |
| ENSSSCG00000035078 | CD40 | 3.788573764 | 0 | UP |
| ENSSSCG00000011951 | NFKBIZ | 3.765628944 | 3.2E-247 | UP |
| ENSSSCG00000005385 | NR4A3 | 3.756739776 | 1.2E-112 | UP |
| ENSSSCG00000022833 | FGF16 | 3.737011301 | 2.82E-11 | UP |
| ENSSSCG00000023785 | TMEM156 | 3.731737722 | 0.000902 | UP |
| ENSSSCG00000039395 | GAS2L2 | 3.665061495 | 0.015147 | UP |
| ENSSSCG00000010445 | ANKRD22 | 3.655684229 | 0.010155 | UP |
| ENSSSCG00000008123 | ARID5A | 3.652263209 | 4.6E-140 | UP |
| ENSSSCG00000001483 | FAM83B | 3.652140509 | 0.000944 | UP |
| ENSSSCG00000001952 | NFKBIA | 3.627998803 | 0 | UP |
| ENSSSCG00000016696 | EVX1 | 3.6051533 | 4.44E-07 | UP |
| ENSSSCG00000009434 | RGCC | 3.605138886 | 0 | UP |
| ENSSSCG00000015549 | RNASEL | 3.604959888 | 1.3E-113 | UP |
| ENSSSCG00000006932 | NA | 3.604939775 | 0.0192 | UP |
| ENSSSCG00000026454 | NA | 3.595772146 | 0 | UP |
| ENSSSCG00000009997 | OSM | 3.582277264 | 5.06E-05 | UP |
| ENSSSCG00000035420 | HES4 | 3.579985905 | 3.15E-37 | UP |
| ENSSSCG00000032417 | NGF | 3.576559978 | 2.4E-255 | UP |
| ENSSSCG00000003805 | PDE4B | 3.559454522 | 0 | UP |
| ENSSSCG00000037087 | PRRT4 | 3.540505401 | 1.46E-42 | UP |
| ENSSSCG00000036011 | ISLR2 | 3.53318328 | 3.65E-06 | UP |
| ENSSSCG00000009320 | FLT1 | 3.529730465 | 1.61E-84 | UP |
| ENSSSCG00000009720 | DDX60 | 3.517313685 | 9.94E-50 | UP |
| ENSSSCG00000017991 | PIK3R5 | 3.510668704 | 1.5E-140 | UP |
| ENSSSCG00000017861 | ASPA | 3.510054649 | 0.016698 | UP |
| ENSSSCG00000010330 | PPIF | 3.486282933 | 0 | UP |
| ENSSSCG00000025560 | PGLYRP2 | 3.475672831 | 8.11E-47 | UP |
| ENSSSCG00000039341 | NA | 3.449170688 | 0.031717 | UP |
| ENSSSCG00000034973 | CXCL12 | 3.447633487 | 3.5E-122 | UP |
| ENSSSCG00000017886 | FBXO39 | 3.437357796 | 4.99E-55 | UP |
| ENSSSCG00000025206 | RNF19B | 3.436018957 | 0 | UP |
| ENSSSCG00000022512 | TRDC | 3.424721832 | 0.03358 | UP |
| ENSSSCG00000017920 | NA | 3.415941145 | 0 | UP |
| ENSSSCG00000027607 | IER3 | 3.413020569 | 0 | UP |
| ENSSSCG00000035859 | WNT5A | 3.405711645 | 1.3E-187 | UP |
| ENSSSCG00000031356 | HES1 | 3.395036298 | 6.2E-235 | UP |
| ENSSSCG00000033613 | FOXS1 | 3.384303274 | 6.88E-62 | UP |
| ENSSSCG00000013940 | NLRP3 | 3.374284859 | 0 | UP |
| ENSSSCG00000012027 | ADAMTS5 | 3.373643081 | 6.7E-185 | UP |
| ENSSSCG00000010504 | BLNK | 3.364929211 | 1.14E-05 | UP |
| ENSSSCG00000020705 | MAP3K8 | 3.348294399 | 6.6E-199 | UP |
| ENSSSCG00000040673 | TMEM140 | 3.346766555 | 1.91E-31 | UP |
| ENSSSCG00000017754 | NA | 3.345685797 | 8.5E-27 | UP |
| ENSSSCG00000017091 | TNIP1 | 3.342887796 | 0 | UP |
| ENSSSCG00000015897 | IFIH1 | 3.335258376 | 1.5E-115 | UP |
| ENSSSCG00000010449 | CH25H | 3.330914442 | 1.19E-22 | UP |
| ENSSSCG00000015595 | ATF3 | 3.329515125 | 0 | UP |
| ENSSSCG00000007486 | CYP24A1 | 3.320788472 | 1.21E-17 | UP |
| ENSSSCG00000000774 | USP18 | 3.320366983 | 5.05E-57 | UP |
| ENSSSCG00000012853 | IRF7 | 3.320056308 | 3.78E-94 | UP |
| ENSSSCG00000032320 | TCIM | 3.313077405 | 6.18E-47 | UP |
| ENSSSCG00000011286 | KLHL40 | 3.312319291 | 8.25E-10 | UP |
| ENSSSCG00000035940 | SPSB1 | 3.307936602 | 1.9E-139 | UP |
| ENSSSCG00000006023 | SYBU | 3.299464237 | 9.1E-121 | UP |
| ENSSSCG00000009786 | HIP1R | 3.281448494 | 1.76E-53 | UP |
| ENSSSCG00000017590 | NA | 3.271216172 | 9.94E-23 | UP |
| ENSSSCG00000002376 | PGF | 3.259596668 | 2.4E-196 | UP |
| ENSSSCG00000013654 | NA | 3.259322127 | 7.76E-23 | UP |
| ENSSSCG00000029507 | RASGEF1B | 3.25182412 | 3.87E-37 | UP |
| ENSSSCG00000038594 | SDC4 | 3.249668696 | 0 | UP |
| ENSSSCG00000029449 | NA | 3.244521804 | 1.49E-10 | UP |
| ENSSSCG00000006923 | GBP2 | 3.237788188 | 1.71E-64 | UP |
| ENSSSCG00000003744 | MOCOS | 3.236891072 | 1.14E-68 | UP |
| ENSSSCG00000009881 | OAS2 | 3.220799105 | 2.19E-58 | UP |
| ENSSSCG00000039042 | NA | 3.21148657 | 3.71E-51 | UP |
| ENSSSCG00000015953 | DLX1 | 3.210933106 | 1.61E-07 | UP |
| ENSSSCG00000014249 | MARCH3 | 3.210579352 | 0 | UP |
| ENSSSCG00000017262 | SLC16A6 | 3.201370681 | 9.34E-06 | UP |
| ENSSSCG00000005593 | OLFML2A | 3.195707547 | 3.4E-173 | UP |
| ENSSSCG00000004218 | RSPO3 | 3.183942262 | 8.48E-27 | UP |
| ENSSSCG00000011730 | IL12A | 3.17964461 | 4.95E-05 | UP |
| ENSSSCG00000012077 | MX1 | 3.176264623 | 1.28E-77 | UP |
| ENSSSCG00000038783 | IGFBP3 | 3.174339152 | 8.55E-07 | UP |
| ENSSSCG00000006802 | NA | 3.167514294 | 6.51E-21 | UP |
| ENSSSCG00000025500 | HTR1D | 3.166751279 | 0.002543 | UP |
| ENSSSCG00000019154 | ssc-mir-155 | 3.163920425 | 1.26E-54 | UP |
| ENSSSCG00000030300 | MT2A | 3.153222185 | 0 | UP |
| ENSSSCG00000007499 | TFAP2C | 3.150414021 | 0.000568 | UP |
| ENSSSCG00000031023 | NA | 3.146694091 | 1.44E-96 | UP |
| ENSSSCG00000009240 | PLAC8 | 3.146661222 | 2.5E-40 | UP |
| ENSSSCG00000040557 | SERTM1 | 3.146038594 | 7.37E-07 | UP |
| ENSSSCG00000027967 | IGSF6 | 3.145264216 | 0.048744 | UP |
| ENSSSCG00000004919 | NEDD4L | 3.144857901 | 0 | UP |
| ENSSSCG00000032652 | NA | 3.142983595 | 6.97E-71 | UP |
| ENSSSCG00000011298 | CDCP1 | 3.141676267 | 3.41E-66 | UP |
| ENSSSCG00000027030 | BDKRB2 | 3.135333808 | 5.9E-278 | UP |
| ENSSSCG00000030108 | ZNFX1 | 3.130977094 | 1.3E-250 | UP |
| ENSSSCG00000025836 | SULT1C4 | 3.125766994 | 2.06E-34 | UP |
| ENSSSCG00000009921 | OASL | 3.123288519 | 4.52E-08 | UP |
| ENSSSCG00000015085 | IL10RA | 3.1228453 | 8.1E-128 | UP |
| ENSSSCG00000000504 | PTPRB | 3.119040716 | 2.07E-17 | UP |
| ENSSSCG00000009410 | RUBCNL | 3.10869949 | 1.98E-11 | UP |
| ENSSSCG00000007465 | B4GALT5 | 3.108137899 | 0 | UP |
| ENSSSCG00000038180 | ADAMTS3 | 3.101862283 | 1.4E-120 | UP |
| ENSSSCG00000015550 | RGS16 | 3.10097633 | 1.1E-149 | UP |
| ENSSSCG00000009979 | NEFH | 3.094345125 | 2.17E-28 | UP |
| ENSSSCG00000015433 | NA | 3.092697676 | 0.000574 | UP |
| ENSSSCG00000021906 | SYT7 | 3.086866823 | 1.56E-22 | UP |
| ENSSSCG00000023710 | REEP1 | 3.086710407 | 6.1E-154 | UP |
| ENSSSCG00000010184 | AGT | 3.086172132 | 1.63E-75 | UP |
| ENSSSCG00000027477 | TBC1D2B | 3.085720411 | 0 | UP |
| ENSSSCG00000006059 | NCALD | 3.085534922 | 7.51E-30 | UP |
| ENSSSCG00000010339 | DYDC2 | 3.085173703 | 1.27E-08 | UP |
| ENSSSCG00000039731 | NA | 3.082460935 | 0 | UP |
| ENSSSCG00000032622 | PPP3CC | 3.078064969 | 0 | UP |
| ENSSSCG00000016031 | CALCRL | 3.064337478 | 1.99E-13 | UP |
| ENSSSCG00000029675 | MMP8 | 3.054756815 | 2.26E-06 | UP |
| ENSSSCG00000033787 | NA | 3.049613996 | 1.38E-16 | UP |
| ENSSSCG00000021383 | CGAS | 3.038527015 | 2.3E-118 | UP |
| ENSSSCG00000009655 | EBF2 | 3.032062691 | 5.2E-134 | UP |
| ENSSSCG00000012996 | CDC42EP2 | 3.028082946 | 4.67E-45 | UP |
| ENSSSCG00000017416 | DHX58 | 3.021872494 | 2.87E-66 | UP |
| ENSSSCG00000034989 | LRRTM2 | 3.013616586 | 1.32E-39 | UP |
| ENSSSCG00000013303 | ABTB2 | 2.99999668 | 3.75E-50 | UP |
| ENSSSCG00000006542 | KCNN3 | 2.996196929 | 4.51E-05 | UP |
| ENSSSCG00000001931 | NA | 2.994537817 | 6.3E-192 | UP |
| ENSSSCG00000030655 | MAMDC2 | 2.993531617 | 0 | UP |
| ENSSSCG00000025410 | PRSS16 | 2.973388642 | 0.000343 | UP |
| ENSSSCG00000037572 | EPSTI1 | 2.97102337 | 3.06E-76 | UP |
| ENSSSCG00000035774 | ERRFI1 | 2.968256985 | 8.7E-116 | UP |
| ENSSSCG00000011391 | CDHR4 | 2.967868049 | 2.14E-09 | UP |
| ENSSSCG00000014221 | LVRN | 2.967322808 | 7.3E-10 | UP |
| ENSSSCG00000003669 | MFSD2A | 2.963237921 | 1.8E-295 | UP |
| ENSSSCG00000021815 | NA | 2.962964935 | 4.1E-106 | UP |
| ENSSSCG00000036340 | ZBTB5 | 2.948865133 | 8.3E-52 | UP |
| ENSSSCG00000010338 | DYDC1 | 2.94131603 | 1.57E-19 | UP |
| ENSSSCG00000023379 | UBE2L6 | 2.938884289 | 1.92E-95 | UP |
| ENSSSCG00000021712 | HERC6 | 2.937110703 | 6.33E-55 | UP |
| ENSSSCG00000008384 | C2orf74 | 2.927531043 | 0.004892 | UP |
| ENSSSCG00000014988 | MMP13 | 2.911446964 | 4.06E-08 | UP |
| ENSSSCG00000036893 | PTHLH | 2.900198663 | 6.6E-185 | UP |
| ENSSSCG00000011218 | SLC4A7 | 2.898948722 | 0 | UP |
| ENSSSCG00000014780 | TRIM21 | 2.897608832 | 9.7E-240 | UP |
| ENSSSCG00000039953 | PNRC1 | 2.893785642 | 1.3E-294 | UP |
| ENSSSCG00000031856 | DACT1 | 2.87920654 | 3.02E-98 | UP |
| ENSSSCG00000011239 | NA | 2.872540232 | 8.96E-32 | UP |
| ENSSSCG00000017466 | CCR7 | 2.864670456 | 0.022931 | UP |
| ENSSSCG00000022312 | RHPN2 | 2.862698418 | 1.49E-34 | UP |
| ENSSSCG00000037247 | NA | 2.858370483 | 4.61E-05 | UP |
| ENSSSCG00000017402 | STAT5A | 2.857200777 | 0 | UP |
| ENSSSCG00000025618 | TAP1 | 2.836692863 | 3E-130 | UP |
| ENSSSCG00000034708 | PITX2 | 2.832689468 | 1.56E-05 | UP |
| ENSSSCG00000016438 | NUB1 | 2.825787285 | 0 | UP |
| ENSSSCG00000023165 | SEMA7A | 2.822115186 | 7.23E-48 | UP |
| ENSSSCG00000006353 | NR1I3 | 2.815753562 | 0.005105 | UP |
| ENSSSCG00000013655 | ICAM1 | 2.792876121 | 0 | UP |
| ENSSSCG00000032097 | DUSP16 | 2.78892841 | 0 | UP |
| ENSSSCG00000006742 | MAB21L3 | 2.786507648 | 9.72E-53 | UP |
| ENSSSCG00000009051 | IL15 | 2.784801945 | 3.61E-07 | UP |
| ENSSSCG00000015512 | PAPPA2 | 2.767276198 | 0.01062 | UP |
| ENSSSCG00000010337 | MAT1A | 2.764008188 | 0.000943 | UP |
| ENSSSCG00000010241 | TET1 | 2.757747303 | 1.9E-230 | UP |
| ENSSSCG00000001695 | VEGFA | 2.749098768 | 2.2E-145 | UP |
| ENSSSCG00000003729 | RNF125 | 2.748703721 | 7.2E-200 | UP |
| ENSSSCG00000016573 | IRF5 | 2.745028778 | 1.81E-30 | UP |
| ENSSSCG00000017995 | USP43 | 2.732160443 | 0.000365 | UP |
| ENSSSCG00000028552 | BHLHE41 | 2.729499489 | 9.1E-248 | UP |
| ENSSSCG00000031868 | XK | 2.720687321 | 8.28E-05 | UP |
| ENSSSCG00000037177 | C2CD4C | 2.713007128 | 0.005735 | UP |
| ENSSSCG00000034207 | CEBPB | 2.698820654 | 0 | UP |
| ENSSSCG00000008617 | FAM49A | 2.675863456 | 8.1E-47 | UP |
| ENSSSCG00000013758 | ZSWIM4 | 2.670951622 | 0 | UP |
| ENSSSCG00000010650 | AFAP1L2 | 2.665408662 | 9.03E-07 | UP |
| ENSSSCG00000026729 | TMEM150C | 2.657189215 | 2.04E-13 | UP |
| ENSSSCG00000032082 | NA | 2.652407195 | 0.022048 | UP |
| ENSSSCG00000000146 | NA | 2.648423059 | 2.84E-85 | UP |
| ENSSSCG00000034633 | CFAP99 | 2.647155256 | 9.43E-05 | UP |
| ENSSSCG00000004493 | SIGLEC15 | 2.64039918 | 2.14E-06 | UP |
| ENSSSCG00000013599 | ANGPTL4 | 2.638161746 | 4E-271 | UP |
| ENSSSCG00000016247 | TM4SF20 | 2.637183283 | 0.000635 | UP |
| ENSSSCG00000009129 | TIFA | 2.632997558 | 6.6E-109 | UP |
| ENSSSCG00000015435 | NAMPT | 2.632774891 | 5.1E-273 | UP |
| ENSSSCG00000010580 | NFKB2 | 2.627710104 | 0 | UP |
| ENSSSCG00000039761 | MYCL | 2.625721007 | 9.4E-16 | UP |
| ENSSSCG00000020872 | NA | 2.625659364 | 6.46E-24 | UP |
| ENSSSCG00000038351 | NA | 2.623345347 | 7.36E-05 | UP |
| ENSSSCG00000022029 | RAP1GAP | 2.610624437 | 2.41E-27 | UP |
| ENSSSCG00000009100 | TNIP3 | 2.604051193 | 4.82E-29 | UP |
| ENSSSCG00000037116 | ZNRF2 | 2.599510077 | 9.09E-36 | UP |
| ENSSSCG00000033453 | BST2 | 2.593668687 | 1.55E-36 | UP |
| ENSSSCG00000032561 | PDCD1LG2 | 2.593144817 | 0.000238 | UP |
| ENSSSCG00000021598 | EVA1C | 2.592695557 | 2.26E-61 | UP |
| ENSSSCG00000037536 | SLC25A28 | 2.591437691 | 2.8E-249 | UP |
| ENSSSCG00000006383 | VANGL2 | 2.58662276 | 3.9E-08 | UP |
| ENSSSCG00000003839 | NA | 2.585487875 | 0 | UP |
| ENSSSCG00000009865 | TBX3 | 2.582813756 | 3.2E-200 | UP |
| ENSSSCG00000005097 | SNAPC1 | 2.580061936 | 2.1E-162 | UP |
| ENSSSCG00000036274 | NA | 2.579038329 | 6.7E-251 | UP |
| ENSSSCG00000015782 | IRF2 | 2.577512321 | 1.31E-70 | UP |
| ENSSSCG00000010302 | USP54 | 2.565095541 | 0 | UP |
| ENSSSCG00000016285 | ECEL1 | 2.560597448 | 1.85E-18 | UP |
| ENSSSCG00000009621 | PHYHIP | 2.554942047 | 1.82E-09 | UP |
| ENSSSCG00000006737 | IGSF3 | 2.545667156 | 1.82E-12 | UP |
| ENSSSCG00000036556 | IL10RB | 2.542701395 | 0 | UP |
| ENSSSCG00000016101 | CFLAR | 2.539627507 | 0 | UP |
| ENSSSCG00000022618 | CMTM8 | 2.52824385 | 2.64E-07 | UP |
| ENSSSCG00000014420 | JAKMIP2 | 2.525015081 | 2.4E-164 | UP |
| ENSSSCG00000028063 | TACC2 | 2.515205472 | 1.9E-303 | UP |
| ENSSSCG00000011133 | PFKFB3 | 2.513766828 | 8.8E-175 | UP |
| ENSSSCG00000034369 | SPSB4 | 2.503249872 | 2.26E-40 | UP |
| ENSSSCG00000013418 | CFD | 2.497734294 | 2E-07 | UP |
| ENSSSCG00000026943 | MRAP2 | 2.495142791 | 9.7E-10 | UP |
| ENSSSCG00000001073 | TPMT | 2.493759127 | 0 | UP |
| ENSSSCG00000035598 | EDN1 | 2.492259825 | 5.86E-14 | UP |
| ENSSSCG00000001912 | PML | 2.491732491 | 2.3E-99 | UP |
| ENSSSCG00000008311 | CYP26B1 | 2.479359422 | 4.02E-44 | UP |
| ENSSSCG00000031794 | TNFAIP8L3 | 2.472402358 | 1.03E-20 | UP |
| ENSSSCG00000030957 | NFKB1 | 2.470524582 | 0 | UP |
| ENSSSCG00000031456 | ARL5B | 2.470399606 | 2.33E-76 | UP |
| ENSSSCG00000010261 | PPA1 | 2.464870327 | 0 | UP |
| ENSSSCG00000002252 | ARRDC4 | 2.463643029 | 4.13E-57 | UP |
| ENSSSCG00000006502 | ARHGEF2 | 2.46278659 | 0 | UP |
| ENSSSCG00000013307 | LMO2 | 2.461473741 | 1.76E-36 | UP |
| ENSSSCG00000023085 | STAC | 2.452068552 | 1.82E-13 | UP |
| ENSSSCG00000008727 | MSX1 | 2.449979182 | 4.86E-60 | UP |
| ENSSSCG00000002135 | PNP | 2.448828961 | 4.2E-289 | UP |
| ENSSSCG00000035612 | COX6B2 | 2.44798176 | 1.74E-10 | UP |
| ENSSSCG00000006196 | NA | 2.442972224 | 2.85E-07 | UP |
| ENSSSCG00000013298 | PDHX | 2.440397916 | 0 | UP |
| ENSSSCG00000024439 | PTGER4 | 2.439963456 | 6E-111 | UP |
| ENSSSCG00000035240 | GPR63 | 2.432179868 | 2.64E-17 | UP |
| ENSSSCG00000033878 | RAB38 | 2.427810202 | 0.005641 | UP |
| ENSSSCG00000012173 | SAT1 | 2.419602613 | 2.7E-174 | UP |
| ENSSSCG00000027709 | PARP9 | 2.416572838 | 9.61E-56 | UP |
| ENSSSCG00000026041 | MAP3K5 | 2.415308941 | 8.4E-235 | UP |
| ENSSSCG00000036932 | WNT6 | 2.414942326 | 1.33E-09 | UP |
| ENSSSCG00000040061 | NINJ1 | 2.408040539 | 1.1E-228 | UP |
| ENSSSCG00000032709 | ARL4A | 2.404063629 | 4.88E-15 | UP |
| ENSSSCG00000026602 | PTGIR | 2.401542751 | 5.79E-84 | UP |
| ENSSSCG00000010454 | IFIT5 | 2.400945348 | 1.12E-75 | UP |
| ENSSSCG00000032015 | SH3BGRL2 | 2.398680824 | 1.3E-124 | UP |
| ENSSSCG00000025770 | ST6GAL1 | 2.395807078 | 0 | UP |
| ENSSSCG00000008496 | EIF2AK2 | 2.394944597 | 1.55E-87 | UP |
| ENSSSCG00000027426 | BCL3 | 2.390820325 | 2.1E-198 | UP |
| ENSSSCG00000001920 | HCN4 | 2.390560605 | 2.92E-05 | UP |
| ENSSSCG00000002716 | MLKL | 2.385390589 | 0 | UP |
| ENSSSCG00000040608 | AKR1B1 | 2.384777502 | 2.7E-250 | UP |
| ENSSSCG00000038500 | TRIB1 | 2.38306616 | 9.9E-211 | UP |
| ENSSSCG00000015375 | ITGB8 | 2.377763546 | 1.4E-261 | UP |
| ENSSSCG00000004479 | FILIP1 | 2.370762582 | 2.96E-07 | UP |
| ENSSSCG00000004053 | TAGAP | 2.369418525 | 3.88E-67 | UP |
| ENSSSCG00000025969 | PTPRR | 2.367872903 | 5.59E-05 | UP |
| ENSSSCG00000007482 | NA | 2.366541696 | 0.001677 | UP |
| ENSSSCG00000016502 | PARP12 | 2.356954138 | 3.1E-120 | UP |
| ENSSSCG00000029002 | PNKD | 2.354962158 | 2.9E-297 | UP |
| ENSSSCG00000008334 | MXD1 | 2.352340619 | 1.14E-46 | UP |
| ENSSSCG00000034378 | IFNGR2 | 2.35201864 | 0 | UP |
| ENSSSCG00000035403 | RFX2 | 2.347987364 | 1.95E-76 | UP |
| ENSSSCG00000023004 | FZD9 | 2.341987164 | 2.11E-14 | UP |
| ENSSSCG00000025535 | TFAP2B | 2.333015966 | 0.00444 | UP |
| ENSSSCG00000015255 | IGSF9B | 2.328847362 | 1.81E-32 | UP |
| ENSSSCG00000021596 | KCNA5 | 2.327779516 | 0.000411 | UP |
| ENSSSCG00000002841 | N4BP1 | 2.324316312 | 1.6E-142 | UP |
| ENSSSCG00000035634 | NA | 2.322439657 | 7.4E-151 | UP |
| ENSSSCG00000028182 | CDK17 | 2.321742687 | 7.4E-295 | UP |
| ENSSSCG00000035297 | ISG12(A) | 2.321142824 | 2.35E-18 | UP |
| ENSSSCG00000040815 | DUSP5 | 2.308604683 | 2.93E-72 | UP |
| ENSSSCG00000014275 | SLC22A4 | 2.305369552 | 0.03091 | UP |
| ENSSSCG00000026951 | PSMB8 | 2.30371287 | 2.6E-148 | UP |
| ENSSSCG00000001233 | TRIM26 | 2.294866393 | 3.7E-172 | UP |
| ENSSSCG00000015823 | NA | 2.293022196 | 2.21E-64 | UP |
| ENSSSCG00000006199 | PREX2 | 2.289365095 | 2.97E-05 | UP |
| ENSSSCG00000002835 | TOX3 | 2.28848761 | 0.03326 | UP |
| ENSSSCG00000022402 | GRAMD2B | 2.287510871 | 0 | UP |
| ENSSSCG00000031109 | HILPDA | 2.283869447 | 0 | UP |
| ENSSSCG00000024096 | RIPK2 | 2.28358809 | 6.1E-213 | UP |
| ENSSSCG00000025593 | NA | 2.281172497 | 0 | UP |
| ENSSSCG00000032686 | RUNX3 | 2.279662775 | 1.63E-06 | UP |
| ENSSSCG00000002294 | ARG2 | 2.273900906 | 3.99E-92 | UP |
| ENSSSCG00000007874 | NA | 2.267459625 | 5.2E-291 | UP |
| ENSSSCG00000038879 | RELB | 2.263689818 | 0 | UP |
| ENSSSCG00000012583 | ACSL4 | 2.263115767 | 0 | UP |
| ENSSSCG00000001841 | RHCG | 2.25718507 | 5.02E-31 | UP |
| ENSSSCG00000023351 | PLA2G4A | 2.253793312 | 0 | UP |
| ENSSSCG00000023957 | CFAP126 | 2.249205228 | 3.71E-21 | UP |
| ENSSSCG00000037063 | PROKR2 | 2.240036919 | 7.97E-07 | UP |
| ENSSSCG00000034610 | NA | 2.239191833 | 1.2E-112 | UP |
| ENSSSCG00000036911 | NA | 2.237963539 | 7.24E-87 | UP |
| ENSSSCG00000036157 | BARX2 | 2.237909864 | 4.11E-09 | UP |
| ENSSSCG00000039998 | CLIC2 | 2.23670538 | 1.6E-115 | UP |
| ENSSSCG00000022011 | NMI | 2.224083445 | 4.3E-179 | UP |
| ENSSSCG00000008881 | RAPGEF2 | 2.216343488 | 0 | UP |
| ENSSSCG00000006161 | IL7 | 2.215149702 | 7.45E-06 | UP |
| ENSSSCG00000001516 | BAK1 | 2.214335661 | 0 | UP |
| ENSSSCG00000004050 | WTAP | 2.213049161 | 0 | UP |
| ENSSSCG00000014224 | SEMA6A | 2.212150758 | 8.09E-98 | UP |
| ENSSSCG00000017797 | SLC6A4 | 2.211127511 | 3.61E-54 | UP |
| ENSSSCG00000009042 | OTUD4 | 2.210101192 | 9.9E-280 | UP |
| ENSSSCG00000017146 | RNF213 | 2.207759483 | 9.27E-49 | UP |
| ENSSSCG00000005967 | FAM84B | 2.206540138 | 3.77E-07 | UP |
| ENSSSCG00000007140 | SMOX | 2.204500026 | 0 | UP |
| ENSSSCG00000011825 | ATP13A3 | 2.199015337 | 1.5E-238 | UP |
| ENSSSCG00000027053 | PACSIN1 | 2.195980763 | 0.017528 | UP |
| ENSSSCG00000036113 | NA | 2.193042213 | 7.04E-58 | UP |
| ENSSSCG00000014996 | NA | 2.190397781 | 0.027278 | UP |
| ENSSSCG00000008348 | PLEK | 2.183485071 | 4.04E-30 | UP |
| ENSSSCG00000032715 | CERS6 | 2.182959135 | 4.1E-112 | UP |
| ENSSSCG00000012265 | CHST7 | 2.182125522 | 7.4E-47 | UP |
| ENSSSCG00000017306 | ITGB3 | 2.181692857 | 3E-236 | UP |
| ENSSSCG00000025598 | COBLL1 | 2.167609798 | 1E-179 | UP |
| ENSSSCG00000014997 | NA | 2.167007208 | 6.1E-222 | UP |
| ENSSSCG00000028460 | S1PR5 | 2.155776814 | 5.19E-16 | UP |
| ENSSSCG00000031781 | PSMB10 | 2.153216832 | 4.1E-270 | UP |
| ENSSSCG00000010169 | SIPA1L2 | 2.150921328 | 5.43E-77 | UP |
| ENSSSCG00000035400 | YPEL2 | 2.148964702 | 7.7E-120 | UP |
| ENSSSCG00000002311 | SUSD6 | 2.130693718 | 4.1E-221 | UP |
| ENSSSCG00000011465 | NA | 2.125847094 | 7.8E-208 | UP |
| ENSSSCG00000015886 | ITGB6 | 2.125648835 | 0.010723 | UP |
| ENSSSCG00000014156 | ARRDC3 | 2.124892358 | 2.46E-90 | UP |
| ENSSSCG00000034802 | NA | 2.11900062 | 4.64E-57 | UP |
| ENSSSCG00000014672 | NA | 2.117106297 | 2.93E-19 | UP |
| ENSSSCG00000003707 | NPC1 | 2.115458374 | 2.5E-271 | UP |
| ENSSSCG00000004509 | LIPG | 2.112572567 | 2.2E-212 | UP |
| ENSSSCG00000030165 | MAFF | 2.110757271 | 3.9E-185 | UP |
| ENSSSCG00000016851 | OSMR | 2.110486927 | 2.3E-232 | UP |
| ENSSSCG00000037530 | NA | 2.109535701 | 2.5E-38 | UP |
| ENSSSCG00000016497 | DENND2A | 2.104928295 | 1.13E-17 | UP |
| ENSSSCG00000033258 | TCF21 | 2.102255238 | 3.9E-137 | UP |
| ENSSSCG00000017614 | TRIM25 | 2.101469379 | 4.81E-80 | UP |
| ENSSSCG00000005533 | PTGS1 | 2.098393342 | 4.2E-102 | UP |
| ENSSSCG00000021586 | ZHX2 | 2.097247115 | 1.5E-128 | UP |
| ENSSSCG00000001231 | NA | 2.09124762 | 3.75E-15 | UP |
| ENSSSCG00000003584 | THEMIS2 | 2.091110947 | 0.001339 | UP |
| ENSSSCG00000017420 | CNP | 2.089929531 | 7.43E-67 | UP |
| ENSSSCG00000008721 | ABLIM2 | 2.081367051 | 0.036139 | UP |
| ENSSSCG00000015650 | MAPKAPK2 | 2.080683678 | 0 | UP |
| ENSSSCG00000003513 | ECE1 | 2.077488307 | 4.9E-102 | UP |
| ENSSSCG00000004918 | ALPK2 | 2.075491434 | 1.99E-05 | UP |
| ENSSSCG00000002004 | PSME2 | 2.072986725 | 1.2E-232 | UP |
| ENSSSCG00000004902 | RNF152 | 2.072173056 | 1.09E-37 | UP |
| ENSSSCG00000014362 | HBEGF | 2.071688769 | 1.38E-34 | UP |
| ENSSSCG00000032221 | FAM110C | 2.069974182 | 2.76E-17 | UP |
| ENSSSCG00000033786 | NA | 2.068688789 | 0 | UP |
| ENSSSCG00000029296 | FOXC2 | 2.057276851 | 4.49E-45 | UP |
| ENSSSCG00000003079 | NA | 2.050476747 | 9.7E-227 | UP |
| ENSSSCG00000004497 | PSTPIP2 | 2.046373054 | 1.84E-11 | UP |
| ENSSSCG00000036008 | TACR1 | 2.04293504 | 0.0299 | UP |
| ENSSSCG00000011670 | PXYLP1 | 2.042626822 | 6.2E-208 | UP |
| ENSSSCG00000035077 | INHBA | 2.0412919 | 1.93E-29 | UP |
| ENSSSCG00000039314 | MCL1 | 2.037783256 | 0 | UP |
| ENSSSCG00000040887 | PAPD5 | 2.037770881 | 5E-134 | UP |
| ENSSSCG00000004367 | POPDC3 | 2.036361602 | 1.57E-06 | UP |
| ENSSSCG00000037670 | TMEM164 | 2.032296546 | 4.6E-125 | UP |
| ENSSSCG00000014034 | N4BP3 | 2.031147633 | 1.29E-19 | UP |
| ENSSSCG00000032436 | NA | 2.029516866 | 3.14E-61 | UP |
| ENSSSCG00000034691 | ZEB2_AS1_1 | 2.027870119 | 1.66E-08 | UP |
| ENSSSCG00000022780 | UGCG | 2.024283537 | 5.3E-132 | UP |
| ENSSSCG00000027340 | NA | 2.023942414 | 2.9E-221 | UP |
| ENSSSCG00000001076 | RNF144B | 2.020397976 | 2.4E-135 | UP |
| ENSSSCG00000015014 | ZC3H12C | 2.018897865 | 2.5E-173 | UP |
| ENSSSCG00000035715 | GCH1 | 2.01154156 | 4.8E-142 | UP |
| ENSSSCG00000032367 | CEBPD | 2.007154002 | 2.1E-183 | UP |
| ENSSSCG00000031817 | NA | 2.004232283 | 0.012797 | UP |
| ENSSSCG00000036746 | RASL10B | 2.003656037 | 2.4E-170 | UP |
| ENSSSCG00000014136 | VCAN | 2.001477485 | 1.1E-147 | UP |
| ENSSSCG00000023972 | DRAM1 | 2.000295343 | 5.1E-192 | UP |
| ENSSSCG00000017672 | MED13 | 1.9973203 | 1.6E-297 | UP |
| ENSSSCG00000016261 | SP110 | 1.99443008 | 3.6E-111 | UP |
| ENSSSCG00000011859 | HEG1 | 1.990066607 | 6.6E-205 | UP |
| ENSSSCG00000033012 | NA | 1.988555863 | 0.024599 | UP |
| ENSSSCG00000002002 | IRF9 | 1.988555328 | 3.8E-05 | UP |
| ENSSSCG00000027372 | SAMD9 | 1.983221302 | 1.36E-67 | UP |
| ENSSSCG00000025588 | FJX1 | 1.98143302 | 4.3E-171 | UP |
| ENSSSCG00000033647 | NA | 1.979845605 | 0.015882 | UP |
| ENSSSCG00000038622 | NA | 1.977105664 | 0.003966 | UP |
| ENSSSCG00000008820 | TEC | 1.975736485 | 3.43E-29 | UP |
| ENSSSCG00000032433 | PTCHD1 | 1.97517241 | 1.18E-48 | UP |
| ENSSSCG00000008768 | ARAP2 | 1.97388606 | 2.22E-06 | UP |
| ENSSSCG00000003137 | PLEKHA4 | 1.973365104 | 1.01E-45 | UP |
| ENSSSCG00000027806 | SAMHD1 | 1.972285833 | 2.9E-126 | UP |
| ENSSSCG00000005994 | SNTB1 | 1.970145328 | 3.21E-07 | UP |
| ENSSSCG00000024161 | NA | 1.967784019 | 7.19E-18 | UP |
| ENSSSCG00000036063 | LPAR6 | 1.96449615 | 5.06E-50 | UP |
| ENSSSCG00000038535 | ARSB | 1.952949766 | 2.8E-249 | UP |
| ENSSSCG00000015559 | NCF2 | 1.951565511 | 0.005814 | UP |
| ENSSSCG00000036520 | EFNA5 | 1.949863047 | 3.5E-100 | UP |
| ENSSSCG00000025097 | TMEM61 | 1.949613718 | 9.64E-32 | UP |
| ENSSSCG00000005724 | SETX | 1.949029784 | 6.1E-76 | UP |
| ENSSSCG00000028536 | LHFPL2 | 1.944549045 | 0 | UP |
| ENSSSCG00000011499 | LRIG1 | 1.943275693 | 3.4E-104 | UP |
| ENSSSCG00000040830 | NA | 1.941691387 | 3.15E-05 | UP |
| ENSSSCG00000011198 | RFTN1 | 1.940592728 | 4.7E-161 | UP |
| ENSSSCG00000008092 | NT5DC4 | 1.939756609 | 0.001362 | UP |
| ENSSSCG00000038471 | NUAK2 | 1.928578264 | 1.31E-41 | UP |
| ENSSSCG00000007067 | JAG1 | 1.928373862 | 3.4E-209 | UP |
| ENSSSCG00000000717 | KCNA6 | 1.928119051 | 1.59E-48 | UP |
| ENSSSCG00000004421 | FYN | 1.925306356 | 3.7E-282 | UP |
| ENSSSCG00000003730 | RNF138 | 1.919285818 | 2.1E-135 | UP |
| ENSSSCG00000006354 | TOMM40L | 1.914835378 | 6.2E-187 | UP |
| ENSSSCG00000016512 | ZC3HAV1 | 1.914732788 | 3.9E-166 | UP |
| ENSSSCG00000006186 | TRPA1 | 1.912314237 | 2.68E-07 | UP |
| ENSSSCG00000036383 | LGALS3BP | 1.911047481 | 6.51E-29 | UP |
| ENSSSCG00000037832 | PMP22 | 1.910695778 | 0 | UP |
| ENSSSCG00000013114 | SLC15A3 | 1.908615654 | 3.3E-110 | UP |
| ENSSSCG00000013297 | CD44 | 1.905979564 | 0 | UP |
| ENSSSCG00000016892 | FST | 1.902831981 | 1.12E-39 | UP |
| ENSSSCG00000038677 | GJB3 | 1.901797154 | 8.29E-06 | UP |
| ENSSSCG00000024596 | NOCT | 1.90097964 | 2.52E-72 | UP |
| ENSSSCG00000004971 | TLE3 | 1.900393183 | 3.48E-86 | UP |
| ENSSSCG00000015379 | NA | 1.899247135 | 2.24E-05 | UP |
| ENSSSCG00000031262 | TXNIP | 1.896676846 | 2.33E-83 | UP |
| ENSSSCG00000017199 | TRIM47 | 1.894235254 | 1.7E-122 | UP |
| ENSSSCG00000008261 | HK2 | 1.893094568 | 2.2E-150 | UP |
| ENSSSCG00000012548 | MUM1L1 | 1.886176577 | 2.97E-80 | UP |
| ENSSSCG00000022649 | SLC7A11 | 1.883879691 | 7.5E-127 | UP |
| ENSSSCG00000027882 | C1orf115 | 1.881852638 | 0.000229 | UP |
| ENSSSCG00000016057 | STAT1 | 1.877185861 | 2.58E-71 | UP |
| ENSSSCG00000035101 | KLF5 | 1.861946753 | 2.1E-140 | UP |
| ENSSSCG00000033222 | TRIM14 | 1.85846687 | 4.77E-56 | UP |
| ENSSSCG00000028331 | IL1R2 | 1.852543216 | 2.89E-10 | UP |
| ENSSSCG00000010540 | ENTPD7 | 1.848973479 | 0 | UP |
| ENSSSCG00000040162 | NUPR1 | 1.84794673 | 9E-117 | UP |
| ENSSSCG00000026893 | NA | 1.847405718 | 1.9E-149 | UP |
| ENSSSCG00000028304 | ZFP36L1 | 1.845316818 | 1.2E-122 | UP |
| ENSSSCG00000017962 | KDM6B | 1.844083777 | 3.11E-40 | UP |
| ENSSSCG00000013360 | TMEM86A | 1.840860743 | 1.31E-22 | UP |
| ENSSSCG00000014012 | GFPT2 | 1.840845641 | 2.8E-226 | UP |
| ENSSSCG00000038521 | CHAC1 | 1.838530075 | 1.3E-80 | UP |
| ENSSSCG00000016018 | FRZB | 1.833287609 | 5.64E-16 | UP |
| ENSSSCG00000001422 | C2 | 1.831957112 | 5.39E-43 | UP |
| ENSSSCG00000040181 | ELL | 1.831288238 | 0 | UP |
| ENSSSCG00000011899 | CD80 | 1.829860454 | 1.45E-10 | UP |
| ENSSSCG00000033307 | GNG4 | 1.828891211 | 1.73E-06 | UP |
| ENSSSCG00000017258 | FAM20A | 1.824535447 | 9.89E-60 | UP |
| ENSSSCG00000026940 | CASP10 | 1.824140798 | 1.5E-184 | UP |
| ENSSSCG00000033660 | FIGNL2 | 1.822138044 | 6.21E-22 | UP |
| ENSSSCG00000038727 | GDNF | 1.820617111 | 9.21E-96 | UP |
| ENSSSCG00000016550 | KLF14 | 1.819248989 | 0.010347 | UP |
| ENSSSCG00000007005 | CSGALNACT1 | 1.814465778 | 9.31E-07 | UP |
| ENSSSCG00000036125 | NA | 1.81367594 | 3.39E-05 | UP |
| ENSSSCG00000002504 | AK7 | 1.813375602 | 0.003525 | UP |
| ENSSSCG00000023592 | TAC1 | 1.812918875 | 2.76E-28 | UP |
| ENSSSCG00000025286 | MCTP1 | 1.808033422 | 4.15E-49 | UP |
| ENSSSCG00000006127 | NBN | 1.807946643 | 7.3E-99 | UP |
| ENSSSCG00000007586 | FSCN1 | 1.799786867 | 2.1E-267 | UP |
| ENSSSCG00000025788 | ENPP4 | 1.798700235 | 3.72E-28 | UP |
| ENSSSCG00000001229 | NA | 1.798494107 | 9.2E-130 | UP |
| ENSSSCG00000017548 | NGFR | 1.795512456 | 3.48E-13 | UP |
| ENSSSCG00000006066 | RNF19A | 1.793069419 | 8.1E-120 | UP |
| ENSSSCG00000026904 | NFKBIB | 1.786786633 | 2.5E-179 | UP |
| ENSSSCG00000009132 | ENPEP | 1.785410005 | 8.6E-06 | UP |
| ENSSSCG00000015871 | NR4A2 | 1.784570881 | 9.29E-52 | UP |
| ENSSSCG00000015584 | PROX1 | 1.779892077 | 3.05E-06 | UP |
| ENSSSCG00000005706 | ABL1 | 1.778234072 | 2.4E-256 | UP |
| ENSSSCG00000034364 | NA | 1.76475741 | 1.9E-148 | UP |
| ENSSSCG00000024136 | AMPH | 1.762355228 | 7.39E-26 | UP |
| ENSSSCG00000017835 | CLUH | 1.759075198 | 1.4E-294 | UP |
| ENSSSCG00000035153 | TRIM38 | 1.755748938 | 1.37E-52 | UP |
| ENSSSCG00000032702 | NA | 1.755382737 | 0.006255 | UP |
| ENSSSCG00000006209 | VCPIP1 | 1.754400543 | 4.5E-140 | UP |
| ENSSSCG00000027060 | TBX2 | 1.753754148 | 8.9E-119 | UP |
| ENSSSCG00000033120 | PALM2 | 1.752761091 | 0 | UP |
| ENSSSCG00000009446 | PCDH17 | 1.748649677 | 2.3E-198 | UP |
| ENSSSCG00000015770 | VEGFC | 1.74799445 | 3.7E-205 | UP |
| ENSSSCG00000001509 | DAXX | 1.746837083 | 2E-107 | UP |
| ENSSSCG00000006776 | MOV10 | 1.740832114 | 2.2E-189 | UP |
| ENSSSCG00000026587 | BATF3 | 1.739492198 | 2.28E-18 | UP |
| ENSSSCG00000029096 | HRH2 | 1.738942456 | 0.004363 | UP |
| ENSSSCG00000008141 | ST6GAL2 | 1.738635685 | 0.008362 | UP |
| ENSSSCG00000040466 | FMN1 | 1.737313981 | 7.1E-154 | UP |
| ENSSSCG00000004952 | SMAD3 | 1.727651638 | 1.7E-252 | UP |
| ENSSSCG00000027660 | IFI44L | 1.726110569 | 6.59E-31 | UP |
| ENSSSCG00000030767 | NA | 1.725882331 | 5.86E-13 | UP |
| ENSSSCG00000021571 | KIF27 | 1.725189312 | 1.72E-79 | UP |
| ENSSSCG00000032434 | PLAUR | 1.723166662 | 2.3E-210 | UP |
| ENSSSCG00000017274 | PITPNC1 | 1.721203645 | 9.8E-49 | UP |
| ENSSSCG00000016997 | FGF18 | 1.720441806 | 1.03E-05 | UP |
| ENSSSCG00000018057 | SOCS7 | 1.719222951 | 5.6E-231 | UP |
| ENSSSCG00000024771 | TICAM1 | 1.71665556 | 3.3E-125 | UP |
| ENSSSCG00000021576 | CD83 | 1.715188322 | 5.72E-51 | UP |
| ENSSSCG00000008866 | GUCY1A3 | 1.714481575 | 1.48E-38 | UP |
| ENSSSCG00000036446 | PALD1 | 1.712451142 | 3.86E-91 | UP |
| ENSSSCG00000024219 | TIGAR | 1.71003142 | 2.67E-61 | UP |
| ENSSSCG00000030153 | SMURF1 | 1.709161166 | 4.9E-134 | UP |
| ENSSSCG00000037241 | RGS2 | 1.706774197 | 1.45E-45 | UP |
| ENSSSCG00000000475 | IRAK3 | 1.705024647 | 1.3E-53 | UP |
| ENSSSCG00000009633 | NA | 1.704713682 | 1.4E-193 | UP |
| ENSSSCG00000029037 | DRD1 | 1.704406783 | 0.022042 | UP |
| ENSSSCG00000006625 | RFX5 | 1.704280935 | 1.82E-67 | UP |
| ENSSSCG00000012112 | ARHGAP6 | 1.703844394 | 2.04E-46 | UP |
| ENSSSCG00000002828 | LPCAT2 | 1.702933866 | 1.28E-05 | UP |
| ENSSSCG00000022961 | CLMP | 1.701238477 | 3.1E-242 | UP |
| ENSSSCG00000026583 | TLR1 | 1.700892571 | 0.008567 | UP |
| ENSSSCG00000005269 | TRPM6 | 1.699739619 | 4.8E-17 | UP |
| ENSSSCG00000016986 | CREBRF | 1.694198293 | 3.68E-45 | UP |
| ENSSSCG00000023684 | MT1A | 1.688520865 | 0.001357 | UP |
| ENSSSCG00000006651 | ADAMTSL4 | 1.688003402 | 5.33E-83 | UP |
| ENSSSCG00000035907 | NA | 1.686850829 | 0.019341 | UP |
| ENSSSCG00000004657 | CEP152 | 1.686669808 | 3.81E-92 | UP |
| ENSSSCG00000015413 | FGL2 | 1.685467361 | 0.035848 | UP |
| ENSSSCG00000032408 | CASP7 | 1.679106136 | 1.3E-175 | UP |
| ENSSSCG00000013366 | LDHA | 1.675600612 | 0 | UP |
| ENSSSCG00000016062 | NABP1 | 1.672347941 | 5.19E-85 | UP |
| ENSSSCG00000007485 | BCAS1 | 1.66663305 | 9.68E-37 | UP |
| ENSSSCG00000004830 | ATP10A | 1.662887372 | 5.9E-87 | UP |
| ENSSSCG00000004825 | CHSY1 | 1.661919753 | 3.3E-234 | UP |
| ENSSSCG00000032517 | DMXL2 | 1.661770492 | 5.02E-68 | UP |
| ENSSSCG00000018450 | SNORD100 | 1.661563151 | 0.010627 | UP |
| ENSSSCG00000009859 | NA | 1.661211701 | 0.013113 | UP |
| ENSSSCG00000018685 | SNORA68 | 1.656322674 | 0.043365 | UP |
| ENSSSCG00000015037 | IL18 | 1.655990761 | 5.18E-66 | UP |
| ENSSSCG00000010502 | CCNJ | 1.649612113 | 6.13E-95 | UP |
| ENSSSCG00000013408 | ADM | 1.643100285 | 2.3E-145 | UP |
| ENSSSCG00000007682 | SH2B2 | 1.64273834 | 3.22E-24 | UP |
| ENSSSCG00000027860 | ERAP2 | 1.6415219 | 9.52E-52 | UP |
| ENSSSCG00000039760 | PRR16 | 1.641174217 | 4.83E-05 | UP |
| ENSSSCG00000005423 | ABCA1 | 1.639855369 | 6.1E-171 | UP |
| ENSSSCG00000013261 | CHRM4 | 1.639505144 | 0.022124 | UP |
| ENSSSCG00000017993 | NTN1 | 1.638254144 | 4.8E-100 | UP |
| ENSSSCG00000020666 | EHD4 | 1.638156213 | 2.8E-197 | UP |
| ENSSSCG00000011437 | ALAS1 | 1.636478301 | 2.5E-232 | UP |
| ENSSSCG00000017607 | TMEM100 | 1.635776133 | 7.62E-09 | UP |
| ENSSSCG00000015545 | GLUL | 1.635577018 | 6.6E-153 | UP |
| ENSSSCG00000038460 | FOXF1 | 1.635141109 | 5.71E-20 | UP |
| ENSSSCG00000007355 | NA | 1.632790182 | 2.2E-298 | UP |
| ENSSSCG00000022679 | COCH | 1.628614489 | 0.02344 | UP |
| ENSSSCG00000021573 | KCNJ5 | 1.626443635 | 2.64E-07 | UP |
| ENSSSCG00000034087 | TNFSF15 | 1.624958326 | 2.12E-15 | UP |
| ENSSSCG00000004149 | NHSL1 | 1.62353048 | 1.71E-44 | UP |
| ENSSSCG00000024108 | SLC43A2 | 1.620946704 | 3.2E-122 | UP |
| ENSSSCG00000004897 | ZCCHC2 | 1.619817056 | 2.86E-83 | UP |
| ENSSSCG00000016230 | EPHA4 | 1.613862192 | 2.8E-113 | UP |
| ENSSSCG00000004540 | ONECUT2 | 1.613618451 | 4.62E-07 | UP |
| ENSSSCG00000007559 | MAFK | 1.612672743 | 1.06E-31 | UP |
| ENSSSCG00000000433 | B4GALNT1 | 1.610846858 | 7.8E-22 | UP |
| ENSSSCG00000001049 | HIVEP1 | 1.608977116 | 1.25E-65 | UP |
| ENSSSCG00000012277 | TIMP1 | 1.60830388 | 4.6E-144 | UP |
| ENSSSCG00000016991 | DUSP1 | 1.606209088 | 1.5E-100 | UP |
| ENSSSCG00000035958 | EVA1A | 1.603754911 | 1.7E-117 | UP |
| ENSSSCG00000012055 | MORC3 | 1.599957346 | 8.55E-69 | UP |
| ENSSSCG00000035371 | NA | 1.598861986 | 3.93E-83 | UP |
| ENSSSCG00000018056 | SRCIN1 | 1.597310752 | 2.08E-05 | UP |
| ENSSSCG00000017915 | VMO1 | 1.594815053 | 0.001279 | UP |
| ENSSSCG00000003718 | TAF4B | 1.594362381 | 4.55E-79 | UP |
| ENSSSCG00000039947 | KCNJ2 | 1.592812279 | 7.52E-26 | UP |
| ENSSSCG00000034570 | IFI6 | 1.590818775 | 1.25E-15 | UP |
| ENSSSCG00000039828 | NA | 1.58702743 | 4.2E-09 | UP |
| ENSSSCG00000036338 | NA | 1.585257001 | 0.024295 | UP |
| ENSSSCG00000015987 | NFE2L2 | 1.582328927 | 2.3E-252 | UP |
| ENSSSCG00000005738 | RALGDS | 1.581908896 | 2.39E-67 | UP |
| ENSSSCG00000028679 | NA | 1.579705217 | 9.1E-276 | UP |
| ENSSSCG00000001703 | NFKBIE | 1.575096013 | 1E-133 | UP |
| ENSSSCG00000012258 | FUNDC1 | 1.574832851 | 4.18E-96 | UP |
| ENSSSCG00000040412 | NA | 1.573377538 | 0.001779 | UP |
| ENSSSCG00000015801 | TLR3 | 1.572997638 | 6.55E-58 | UP |
| ENSSSCG00000004791 | RASGRP1 | 1.57160938 | 0.033022 | UP |
| ENSSSCG00000003670 | RLF | 1.569134898 | 1.68E-93 | UP |
| ENSSSCG00000000843 | TXNRD1 | 1.566597194 | 0 | UP |
| ENSSSCG00000005083 | DHRS7 | 1.564424833 | 4.7E-115 | UP |
| ENSSSCG00000006718 | ZNF697 | 1.562981303 | 1.2E-145 | UP |
| ENSSSCG00000022490 | GPR83 | 1.562056734 | 0.02889 | UP |
| ENSSSCG00000032360 | PANX1 | 1.559033427 | 2.55E-53 | UP |
| ENSSSCG00000011047 | FAM171A1 | 1.55734657 | 2.2E-100 | UP |
| ENSSSCG00000004687 | B2M | 1.553451268 | 1.47E-78 | UP |
| ENSSSCG00000007469 | PTPN1 | 1.551742312 | 1.1E-139 | UP |
| ENSSSCG00000014171 | ERAP1 | 1.551083779 | 1.3E-134 | UP |
| ENSSSCG00000004384 | NR2E1 | 1.548627843 | 0.025433 | UP |
| ENSSSCG00000005944 | NDRG1 | 1.540482066 | 2.7E-149 | UP |
| ENSSSCG00000035650 | NA | 1.539881015 | 2.6E-233 | UP |
| ENSSSCG00000032773 | ZEB2_AS1_4 | 1.537078829 | 0.025187 | UP |
| ENSSSCG00000003763 | IFI44 | 1.536704146 | 4.44E-22 | UP |
| ENSSSCG00000028637 | CYP27B1 | 1.531612144 | 5.06E-09 | UP |
| ENSSSCG00000032778 | PLEKHG1 | 1.53133526 | 7.34E-23 | UP |
| ENSSSCG00000033844 | CYP1B1 | 1.531060506 | 1.7E-244 | UP |
| ENSSSCG00000026466 | SLC23A2 | 1.529681373 | 4.7E-288 | UP |
| ENSSSCG00000029230 | ECM1 | 1.528001011 | 1.9E-130 | UP |
| ENSSSCG00000011630 | ACKR4 | 1.52286274 | 0.028983 | UP |
| ENSSSCG00000040847 | AP1S3 | 1.52147899 | 9.36E-13 | UP |
| ENSSSCG00000005511 | TRAF1 | 1.520549009 | 7.22E-12 | UP |
| ENSSSCG00000035051 | ADORA2B | 1.519242319 | 1.3E-136 | UP |
| ENSSSCG00000025114 | FMNL3 | 1.514985347 | 4.5E-179 | UP |
| ENSSSCG00000034993 | NA | 1.514549147 | 1.42E-80 | UP |
| ENSSSCG00000006543 | ADAR | 1.514114704 | 1.3E-111 | UP |
| ENSSSCG00000014800 | RNF121 | 1.513510612 | 2.1E-121 | UP |
| ENSSSCG00000005224 | GLIS3 | 1.513312615 | 5.69E-95 | UP |
| ENSSSCG00000021646 | KLF9 | 1.508872992 | 1.35E-87 | UP |
| ENSSSCG00000034753 | FAM177A1 | 1.508336451 | 3.5E-112 | UP |
| ENSSSCG00000017756 | NLK | 1.507289732 | 1.39E-88 | UP |
| ENSSSCG00000005943 | ST3GAL1 | 1.505443051 | 1.2E-191 | UP |
| ENSSSCG00000013766 | IL27RA | 1.50275765 | 1.4E-179 | UP |
| ENSSSCG00000010581 | PSD | 1.494716544 | 3.93E-07 | UP |
| ENSSSCG00000016262 | NA | 1.494279555 | 9.11E-86 | UP |
| ENSSSCG00000022060 | RASSF10 | 1.492745432 | 0.042746 | UP |
| ENSSSCG00000008374 | B3GNT2 | 1.491183877 | 3E-131 | UP |
| ENSSSCG00000015569 | SWT1 | 1.48003035 | 5.24E-46 | UP |
| ENSSSCG00000025423 | KCNK5 | 1.478797919 | 5.28E-13 | UP |
| ENSSSCG00000016922 | GPBP1 | 1.475361401 | 5.1E-140 | UP |
| ENSSSCG00000039608 | SNORD62 | 1.473641734 | 0.007571 | UP |
| ENSSSCG00000022925 | NA | 1.473253306 | 4E-112 | UP |
| ENSSSCG00000023784 | SEMA3C | 1.471637175 | 1.2E-206 | UP |
| ENSSSCG00000001394 | NA | 1.471002583 | 3.2E-111 | UP |
| ENSSSCG00000015667 | MBD5 | 1.470489593 | 1.28E-68 | UP |
| ENSSSCG00000021557 | SULT1A3 | 1.46906273 | 9.8E-07 | UP |
| ENSSSCG00000015235 | ETS1 | 1.468868849 | 8.6E-268 | UP |
| ENSSSCG00000016322 | ACKR3 | 1.465562918 | 4.6E-144 | UP |
| ENSSSCG00000008388 | REL | 1.464558325 | 3.09E-68 | UP |
| ENSSSCG00000035500 | ZEB2_AS1_3 | 1.462179278 | 0.001046 | UP |
| ENSSSCG00000040839 | NA | 1.458177966 | 1.21E-06 | UP |
| ENSSSCG00000015403 | HGF | 1.453922744 | 5.45E-05 | UP |
| ENSSSCG00000004420 | TRAF3IP2 | 1.453644922 | 8.4E-201 | UP |
| ENSSSCG00000008767 | NA | 1.453628747 | 0.000356 | UP |
| ENSSSCG00000010448 | FAS | 1.452382623 | 4.1E-113 | UP |
| ENSSSCG00000010071 | MMP11 | 1.450639531 | 1.8E-43 | UP |
| ENSSSCG00000005186 | TTC39B | 1.45050037 | 4.78E-79 | UP |
| ENSSSCG00000000728 | PARP11 | 1.449350888 | 6.8E-39 | UP |
| ENSSSCG00000040047 | NA | 1.447631711 | 5.49E-06 | UP |
| ENSSSCG00000033879 | ZNF280B | 1.445751366 | 1.25E-84 | UP |
| ENSSSCG00000011212 | RARB | 1.444425363 | 7.24E-74 | UP |
| ENSSSCG00000015301 | STEAP1 | 1.443231972 | 7.69E-95 | UP |
| ENSSSCG00000011828 | FAM43A | 1.443188837 | 3.2E-107 | UP |
| ENSSSCG00000023247 | OPTN | 1.442720742 | 4E-203 | UP |
| ENSSSCG00000010772 | ADAM8 | 1.440654664 | 5.26E-18 | UP |
| ENSSSCG00000030042 | SBNO2 | 1.440370111 | 1.4E-157 | UP |
| ENSSSCG00000002375 | RPS6KL1 | 1.439623446 | 6.65E-64 | UP |
| ENSSSCG00000006309 | CD247 | 1.437045371 | 7.87E-12 | UP |
| ENSSSCG00000014670 | TRIM5 | 1.436330836 | 1.74E-60 | UP |
| ENSSSCG00000001849 | ANPEP | 1.435519651 | 4.4E-134 | UP |
| ENSSSCG00000034347 | TRIM56 | 1.430242396 | 2.1E-222 | UP |
| ENSSSCG00000003983 | SMAP2 | 1.429610551 | 4.4E-163 | UP |
| ENSSSCG00000009216 | SPP1 | 1.428864592 | 4.7E-114 | UP |
| ENSSSCG00000011576 | HRH1 | 1.428468412 | 3.99E-09 | UP |
| ENSSSCG00000012276 | SYN1 | 1.424883738 | 5.11E-08 | UP |
| ENSSSCG00000015411 | PTPN12 | 1.424026282 | 5.1E-134 | UP |
| ENSSSCG00000017087 | GM2A | 1.423877146 | 1.79E-54 | UP |
| ENSSSCG00000016186 | TMBIM1 | 1.422571622 | 1.2E-171 | UP |
| ENSSSCG00000009445 | PCDH8 | 1.421912542 | 0.002492 | UP |
| ENSSSCG00000015960 | MAP3K20 | 1.419268513 | 1.9E-150 | UP |
| ENSSSCG00000001064 | GMPR | 1.415293558 | 1.81E-10 | UP |
| ENSSSCG00000005935 | AGO2 | 1.415145323 | 1.9E-107 | UP |
| ENSSSCG00000007484 | ZNF217 | 1.415102864 | 1.9E-127 | UP |
| ENSSSCG00000002669 | CRISPLD2 | 1.41384824 | 3.8E-124 | UP |
| ENSSSCG00000006875 | PLPPR4 | 1.410979331 | 5.79E-40 | UP |
| ENSSSCG00000022446 | SEL1L3 | 1.410384966 | 4.56E-26 | UP |
| ENSSSCG00000030278 | MLLT11 | 1.40993424 | 2.5E-51 | UP |
| ENSSSCG00000010948 | NA | 1.409910272 | 2.41E-32 | UP |
| ENSSSCG00000009664 | PTK2B | 1.407154322 | 1.73E-37 | UP |
| ENSSSCG00000028964 | PIM2 | 1.40684838 | 4.77E-34 | UP |
| ENSSSCG00000003167 | FLT3LG | 1.404779058 | 9.95E-71 | UP |
| ENSSSCG00000031789 | ACSL5 | 1.40191434 | 3.6E-231 | UP |
| ENSSSCG00000006864 | CDC14A | 1.401853098 | 2.96E-41 | UP |
| ENSSSCG00000001916 | C15orf59 | 1.401565087 | 5.89E-08 | UP |
| ENSSSCG00000009293 | NA | 1.398991675 | 1.93E-17 | UP |
| ENSSSCG00000010987 | UBAP2 | 1.398766505 | 8.3E-257 | UP |
| ENSSSCG00000010755 | PTPRE | 1.398736769 | 4.1E-06 | UP |
| ENSSSCG00000016715 | OSBPL3 | 1.395470031 | 4.5E-111 | UP |
| ENSSSCG00000023526 | RAPGEF3 | 1.394190221 | 1.56E-07 | UP |
| ENSSSCG00000037642 | ARID3A | 1.390938015 | 8.46E-16 | UP |
| ENSSSCG00000016568 | AHCYL2 | 1.39081317 | 1.22E-35 | UP |
| ENSSSCG00000015839 | NA | 1.387938018 | 1.79E-07 | UP |
| ENSSSCG00000011264 | CSRNP1 | 1.38757197 | 1.34E-33 | UP |
| ENSSSCG00000034858 | RAP1GAP2 | 1.385022765 | 4.24E-23 | UP |
| ENSSSCG00000006917 | NA | 1.383062869 | 2.11E-63 | UP |
| ENSSSCG00000024671 | WNT9A | 1.377058079 | 1.82E-23 | UP |
| ENSSSCG00000029438 | SESN2 | 1.375840468 | 3.87E-74 | UP |
| ENSSSCG00000006009 | EXT1 | 1.368603859 | 1.4E-159 | UP |
| ENSSSCG00000017403 | STAT3 | 1.365540851 | 3E-197 | UP |
| ENSSSCG00000007659 | ZCWPW1 | 1.364335848 | 3.61E-12 | UP |
| ENSSSCG00000016656 | ELMO1 | 1.362427457 | 9.1E-64 | UP |
| ENSSSCG00000024174 | TGIF1 | 1.361078633 | 1.41E-86 | UP |
| ENSSSCG00000013664 | C19orf66 | 1.358033546 | 1.55E-20 | UP |
| ENSSSCG00000009396 | PHF11 | 1.354652086 | 3.5E-178 | UP |
| ENSSSCG00000014303 | JADE2 | 1.346602936 | 6.69E-75 | UP |
| ENSSSCG00000009927 | FAM222A | 1.342443983 | 0.007875 | UP |
| ENSSSCG00000007000 | FAT1 | 1.337879078 | 9.41E-73 | UP |
| ENSSSCG00000015525 | TOR3A | 1.337858241 | 5.25E-75 | UP |
| ENSSSCG00000001909 | STRA6 | 1.336687671 | 0.000447 | UP |
| ENSSSCG00000034692 | SH3BP4 | 1.336520317 | 5.7E-116 | UP |
| ENSSSCG00000012890 | TCIRG1 | 1.334002687 | 2.19E-69 | UP |
| ENSSSCG00000010256 | COL13A1 | 1.332071182 | 0.002467 | UP |
| ENSSSCG00000036418 | SLC8A1 | 1.331794613 | 0.000266 | UP |
| ENSSSCG00000015302 | STEAP2 | 1.329964965 | 2.27E-91 | UP |
| ENSSSCG00000040617 | TNFAIP8 | 1.329248026 | 2.65E-25 | UP |
| ENSSSCG00000023408 | SAMD4A | 1.327435025 | 0 | UP |
| ENSSSCG00000010148 | ERO1B | 1.322245438 | 1.8E-20 | UP |
| ENSSSCG00000007817 | IL4R | 1.318477877 | 4E-101 | UP |
| ENSSSCG00000004629 | LYSMD2 | 1.316294887 | 5.04E-22 | UP |
| ENSSSCG00000015979 | HOXD13 | 1.315849377 | 6.86E-05 | UP |
| ENSSSCG00000003080 | NA | 1.313993451 | 4.24E-07 | UP |
| ENSSSCG00000022361 | NA | 1.313040808 | 3.4E-07 | UP |
| ENSSSCG00000016758 | NA | 1.312820852 | 8.6E-113 | UP |
| ENSSSCG00000016237 | DOCK10 | 1.310332613 | 1.56E-05 | UP |
| ENSSSCG00000033037 | NA | 1.308352113 | 1.68E-31 | UP |
| ENSSSCG00000010341 | TSPAN14 | 1.308094007 | 3.17E-85 | UP |
| ENSSSCG00000021343 | ZEB2 | 1.306430481 | 2E-196 | UP |
| ENSSSCG00000006173 | GDAP1 | 1.305462179 | 7.43E-28 | UP |
| ENSSSCG00000004921 | NA | 1.304759325 | 1.88E-23 | UP |
| ENSSSCG00000015522 | ANGPTL1 | 1.30253626 | 1.46E-09 | UP |
| ENSSSCG00000009348 | STARD13 | 1.301161158 | 1.4E-117 | UP |
| ENSSSCG00000017551 | FAM117A | 1.298430264 | 8.92E-24 | UP |
| ENSSSCG00000002340 | PSEN1 | 1.297240956 | 1.6E-177 | UP |
| ENSSSCG00000024071 | SCARF1 | 1.295940868 | 6.52E-26 | UP |
| ENSSSCG00000014565 | NA | 1.293027386 | 4.12E-52 | UP |
| ENSSSCG00000031882 | PNPT1 | 1.290949479 | 3.56E-59 | UP |
| ENSSSCG00000005364 | TDRD7 | 1.286364037 | 3.07E-59 | UP |
| ENSSSCG00000033581 | RGS7BP | 1.285021253 | 0.000489 | UP |
| ENSSSCG00000029754 | SLC39A2 | 1.279610158 | 0.005163 | UP |
| ENSSSCG00000007864 | GPRC5B | 1.279320992 | 3.5E-132 | UP |
| ENSSSCG00000021902 | GABRP | 1.278467748 | 0.008379 | UP |
| ENSSSCG00000026318 | NETO2 | 1.277610058 | 3.33E-19 | UP |
| ENSSSCG00000005288 | TLE4 | 1.277365237 | 6.54E-72 | UP |
| ENSSSCG00000040445 | RND3 | 1.276597286 | 1.2E-100 | UP |
| ENSSSCG00000011518 | SHQ1 | 1.276414358 | 2.64E-41 | UP |
| ENSSSCG00000012743 | MTMR1 | 1.275513851 | 1.52E-88 | UP |
| ENSSSCG00000014214 | TRIM36 | 1.273724965 | 6.3E-06 | UP |
| ENSSSCG00000016233 | SERPINE2 | 1.271269061 | 8.1E-117 | UP |
| ENSSSCG00000021206 | IL1RAP | 1.269429713 | 2.52E-60 | UP |
| ENSSSCG00000008641 | ADAM17 | 1.268525265 | 6.2E-190 | UP |
| ENSSSCG00000022447 | F3 | 1.267927752 | 5.03E-15 | UP |
| ENSSSCG00000009440 | ELF1 | 1.266569493 | 3.4E-136 | UP |
| ENSSSCG00000013457 | DOT1L | 1.266515667 | 1.57E-70 | UP |
| ENSSSCG00000009616 | HR | 1.263681146 | 1.68E-56 | UP |
| ENSSSCG00000032644 | HLX | 1.263615284 | 3.95E-37 | UP |
| ENSSSCG00000004464 | FAM46A | 1.26345504 | 3.93E-57 | UP |
| ENSSSCG00000012014 | NA | 1.263287012 | 4E-150 | UP |
| ENSSSCG00000035791 | SIX5 | 1.261738108 | 1.01E-47 | UP |
| ENSSSCG00000004114 | RAB32 | 1.259949826 | 2.5E-175 | UP |
| ENSSSCG00000022064 | MLNR | 1.256967957 | 1.48E-06 | UP |
| ENSSSCG00000008043 | TRAF7 | 1.256861674 | 3.5E-171 | UP |
| ENSSSCG00000038671 | TNFRSF6B | 1.256818763 | 2.01E-05 | UP |
| ENSSSCG00000016501 | KDM7A | 1.256010112 | 1.64E-25 | UP |
| ENSSSCG00000000396 | STAT2 | 1.255903068 | 2.83E-66 | UP |
| ENSSSCG00000025856 | TMEM106A | 1.255456221 | 1.45E-34 | UP |
| ENSSSCG00000003705 | CABLES1 | 1.253139339 | 1.18E-88 | UP |
| ENSSSCG00000016690 | CREB5 | 1.253093896 | 1.28E-48 | UP |
| ENSSSCG00000021738 | NA | 1.250185181 | 3.09E-86 | UP |
| ENSSSCG00000008624 | LPIN1 | 1.250024536 | 3.1E-102 | UP |
| ENSSSCG00000025194 | ZSWIM6 | 1.248989444 | 6.1E-54 | UP |
| ENSSSCG00000009169 | SLC39A8 | 1.247849655 | 1.03E-07 | UP |
| ENSSSCG00000031462 | ZNRF1 | 1.247636052 | 2E-109 | UP |
| ENSSSCG00000007733 | NA | 1.247543747 | 1.12E-61 | UP |
| ENSSSCG00000024084 | SNORD12 | 1.247255297 | 0.011109 | UP |
| ENSSSCG00000012026 | ADAMTS1 | 1.247230654 | 9.28E-43 | UP |
| ENSSSCG00000037910 | DUSP6 | 1.246932627 | 5.32E-65 | UP |
| ENSSSCG00000037452 | HTR2A | 1.246258612 | 3.85E-15 | UP |
| ENSSSCG00000011454 | SFMBT1 | 1.245962621 | 6.86E-55 | UP |
| ENSSSCG00000005992 | SHAS2 | 1.245535525 | 2.22E-13 | UP |
| ENSSSCG00000026082 | DNAJC3 | 1.239374072 | 5.4E-144 | UP |
| ENSSSCG00000001398 | NA | 1.238160032 | 3.08E-84 | UP |
| ENSSSCG00000038080 | EMCN | 1.237150027 | 0.004563 | UP |
| ENSSSCG00000011251 | MYD88 | 1.23540276 | 2.64E-83 | UP |
| ENSSSCG00000012074 | NA | 1.235188732 | 4E-142 | UP |
| ENSSSCG00000011641 | SLCO2A1 | 1.234461929 | 1.81E-12 | UP |
| ENSSSCG00000023192 | NA | 1.231798152 | 0.04944 | UP |
| ENSSSCG00000012029 | BACH1 | 1.231199927 | 2.27E-55 | UP |
| ENSSSCG00000011699 | HPS3 | 1.230235163 | 7.17E-83 | UP |
| ENSSSCG00000010509 | PIK3AP1 | 1.22836315 | 1.91E-05 | UP |
| ENSSSCG00000023400 | ZNF598 | 1.227166301 | 5.43E-69 | UP |
| ENSSSCG00000004156 | IFNGR1 | 1.226812711 | 2.6E-98 | UP |
| ENSSSCG00000016554 | MEST | 1.222051006 | 7.44E-25 | UP |
| ENSSSCG00000033115 | IFNAR1 | 1.221378826 | 1.2E-123 | UP |
| ENSSSCG00000007470 | RIPOR3 | 1.219687358 | 1.6E-13 | UP |
| ENSSSCG00000005096 | HIF1A | 1.218430649 | 7.7E-230 | UP |
| ENSSSCG00000010996 | BAG1 | 1.217456369 | 7.2E-106 | UP |
| ENSSSCG00000005098 | SYT16 | 1.216545922 | 0.000891 | UP |
| ENSSSCG00000032171 | TCF20 | 1.215771914 | 3.4E-125 | UP |
| ENSSSCG00000001042 | MAK | 1.213931871 | 1.52E-05 | UP |
| ENSSSCG00000025053 | RYBP | 1.211233682 | 1.69E-39 | UP |
| ENSSSCG00000011596 | TRH | 1.210687811 | 2.13E-07 | UP |
| ENSSSCG00000031118 | PREX1 | 1.208728381 | 3.2E-107 | UP |
| ENSSSCG00000032728 | EFNB1 | 1.208236758 | 7.76E-65 | UP |
| ENSSSCG00000018015 | DNAH9 | 1.207702862 | 0.000257 | UP |
| ENSSSCG00000034012 | CASP3 | 1.206086453 | 1.88E-82 | UP |
| ENSSSCG00000001554 | SRPK1 | 1.2053681 | 9.31E-91 | UP |
| ENSSSCG00000021457 | ZBTB8A | 1.204464955 | 6.91E-37 | UP |
| ENSSSCG00000025298 | TWISTNB | 1.203763966 | 1.07E-74 | UP |
| ENSSSCG00000027777 | NA | 1.203031507 | 6.62E-48 | UP |
| ENSSSCG00000004075 | RGS17 | 1.202067854 | 1.61E-36 | UP |
| ENSSSCG00000023662 | CHST3 | 1.200451386 | 5.8E-117 | UP |
| ENSSSCG00000011361 | SLC26A6 | 1.19940292 | 1.02E-19 | UP |
| ENSSSCG00000016053 | NA | 1.196709642 | 1.85E-15 | UP |
| ENSSSCG00000038505 | MSI2 | 1.196630862 | 1.99E-22 | UP |
| ENSSSCG00000016140 | FZD5 | 1.196198604 | 4.04E-19 | UP |
| ENSSSCG00000017865 | CTNS | 1.193867418 | 8.4E-99 | UP |
| ENSSSCG00000034702 | NA | 1.193420782 | 2.57E-07 | UP |
| ENSSSCG00000015303 | CFAP69 | 1.192209905 | 1.12E-09 | UP |
| ENSSSCG00000040663 | HERPUD1 | 1.190876195 | 1.7E-159 | UP |
| ENSSSCG00000016027 | ITGAV | 1.188989433 | 4E-93 | UP |
| ENSSSCG00000002516 | WARS | 1.188183999 | 3.03E-67 | UP |
| ENSSSCG00000010438 | ATAD1 | 1.185529827 | 6.36E-55 | UP |
| ENSSSCG00000039779 | CHGB | 1.184034859 | 0.000902 | UP |
| ENSSSCG00000037598 | SNX10 | 1.18396545 | 1.55E-06 | UP |
| ENSSSCG00000000704 | TAPBPL | 1.18367669 | 7.56E-68 | UP |
| ENSSSCG00000011493 | ATXN7 | 1.183381224 | 9.32E-91 | UP |
| ENSSSCG00000007554 | ZFAND2A | 1.179776118 | 2.46E-83 | UP |
| ENSSSCG00000024674 | ABL2 | 1.175911061 | 5.5E-77 | UP |
| ENSSSCG00000015563 | RGL1 | 1.17535858 | 5.46E-54 | UP |
| ENSSSCG00000029763 | IFI35 | 1.175045514 | 2.94E-49 | UP |
| ENSSSCG00000005589 | NR6A1 | 1.174818221 | 1.99E-10 | UP |
| ENSSSCG00000030048 | PLEKHG2 | 1.174567613 | 6.5E-117 | UP |
| ENSSSCG00000031327 | NA | 1.172129459 | 0.046787 | UP |
| ENSSSCG00000009850 | TAOK3 | 1.171731309 | 1.9E-112 | UP |
| ENSSSCG00000040834 | NA | 1.165410193 | 0.012414 | UP |
| ENSSSCG00000037579 | PPBP | 1.163609822 | 0.000501 | UP |
| ENSSSCG00000009029 | ARHGAP10 | 1.161210909 | 1.43E-68 | UP |
| ENSSSCG00000026499 | NMT2 | 1.160730728 | 3.8E-20 | UP |
| ENSSSCG00000012967 | FOSL1 | 1.160384117 | 5.33E-83 | UP |
| ENSSSCG00000004781 | NA | 1.158860965 | 0.03915 | UP |
| ENSSSCG00000032877 | NA | 1.158829723 | 6.03E-10 | UP |
| ENSSSCG00000028097 | NA | 1.15872702 | 2.2E-135 | UP |
| ENSSSCG00000009806 | SETD1B | 1.155211274 | 5.44E-71 | UP |
| ENSSSCG00000035181 | RNF24 | 1.154889077 | 4.92E-49 | UP |
| ENSSSCG00000012375 | DLG3 | 1.154180843 | 5.23E-06 | UP |
| ENSSSCG00000008275 | TTC31 | 1.153330663 | 2.33E-87 | UP |
| ENSSSCG00000016981 | CPEB4 | 1.152926161 | 4.64E-77 | UP |
| ENSSSCG00000002267 | RGMA | 1.150306473 | 2.06E-40 | UP |
| ENSSSCG00000027529 | BIRC3 | 1.150263867 | 3.24E-60 | UP |
| ENSSSCG00000004989 | FBXO33 | 1.148525092 | 3.81E-80 | UP |
| ENSSSCG00000001507 | TAPBP | 1.147559952 | 1.59E-86 | UP |
| ENSSSCG00000003580 | EYA3 | 1.147158055 | 6.4E-142 | UP |
| ENSSSCG00000016243 | RHBDD1 | 1.147136957 | 3.92E-85 | UP |
| ENSSSCG00000016518 | TRIM24 | 1.14543864 | 1.64E-72 | UP |
| ENSSSCG00000023738 | COG3 | 1.144428817 | 1.6E-169 | UP |
| ENSSSCG00000023803 | ELK3 | 1.141577022 | 1.8E-133 | UP |
| ENSSSCG00000015649 | DYRK3 | 1.141241968 | 3.38E-19 | UP |
| ENSSSCG00000039330 | ANO8 | 1.139672077 | 2.7E-115 | UP |
| ENSSSCG00000039758 | NA | 1.139251678 | 0.003499 | UP |
| ENSSSCG00000001036 | TFAP2A | 1.138350364 | 0.000168 | UP |
| ENSSSCG00000014852 | ARRB1 | 1.137194938 | 1.82E-11 | UP |
| ENSSSCG00000006159 | HEY1 | 1.135992982 | 0.001047 | UP |
| ENSSSCG00000034942 | STAR | 1.134642811 | 0.010119 | UP |
| ENSSSCG00000001951 | PSMA6 | 1.130469617 | 1.4E-155 | UP |
| ENSSSCG00000001848 | MESP2 | 1.129821053 | 3.37E-31 | UP |
| ENSSSCG00000037619 | EEF1AKMT3 | 1.124793439 | 5.63E-23 | UP |
| ENSSSCG00000024793 | PORCN | 1.124292813 | 7.94E-64 | UP |
| ENSSSCG00000000766 | CECR2 | 1.123052198 | 1.4E-10 | UP |
| ENSSSCG00000007079 | FLRT3 | 1.121570293 | 3.19E-38 | UP |
| ENSSSCG00000010235 | SIRT1 | 1.120641218 | 2.34E-53 | UP |
| ENSSSCG00000010638 | TCF7L2 | 1.119617934 | 1.49E-29 | UP |
| ENSSSCG00000006360 | B4GALT3 | 1.118152944 | 2.36E-37 | UP |
| ENSSSCG00000010219 | ARID5B | 1.118116369 | 3.88E-91 | UP |
| ENSSSCG00000036693 | NA | 1.11707316 | 7.03E-07 | UP |
| ENSSSCG00000036865 | NA | 1.115273534 | 0.038435 | UP |
| ENSSSCG00000020783 | SLC41A1 | 1.114539129 | 8.5E-113 | UP |
| ENSSSCG00000040550 | CASC1 | 1.111363493 | 1.36E-17 | UP |
| ENSSSCG00000011903 | NA | 1.11063678 | 2.97E-90 | UP |
| ENSSSCG00000029174 | CCDC126 | 1.108254461 | 2.52E-25 | UP |
| ENSSSCG00000005215 | JAK2 | 1.108182224 | 3.88E-50 | UP |
| ENSSSCG00000000951 | NA | 1.10595573 | 1.01E-86 | UP |
| ENSSSCG00000037267 | MAX | 1.105168707 | 2.04E-70 | UP |
| ENSSSCG00000033367 | PLEKHA2 | 1.105153803 | 7.02E-47 | UP |
| ENSSSCG00000012156 | CDKL5 | 1.099298052 | 8E-21 | UP |
| ENSSSCG00000016824 | RAI14 | 1.098104637 | 4E-138 | UP |
| ENSSSCG00000016653 | DNAJB9 | 1.096562575 | 2.65E-60 | UP |
| ENSSSCG00000005965 | MYC | 1.093393822 | 8.72E-48 | UP |
| ENSSSCG00000016263 | NA | 1.092743636 | 5.32E-22 | UP |
| ENSSSCG00000003702 | GATA6 | 1.092741386 | 2E-08 | UP |
| ENSSSCG00000030507 | SMNDC1 | 1.09245977 | 9.27E-52 | UP |
| ENSSSCG00000004859 | ZNF516 | 1.08508051 | 6.79E-32 | UP |
| ENSSSCG00000027991 | NA | 1.084898765 | 1.75E-35 | UP |
| ENSSSCG00000003155 | PPP1R15A | 1.084621607 | 6.09E-72 | UP |
| ENSSSCG00000034484 | SPEN | 1.084432314 | 1.57E-63 | UP |
| ENSSSCG00000037202 | CACNG4 | 1.084095735 | 0.030332 | UP |
| ENSSSCG00000009827 | HVCN1 | 1.083546164 | 4.09E-25 | UP |
| ENSSSCG00000016286 | PRSS56 | 1.082480149 | 0.029197 | UP |
| ENSSSCG00000011004 | TOPORS | 1.079687655 | 9.29E-74 | UP |
| ENSSSCG00000005375 | CORO2A | 1.079523991 | 8.48E-11 | UP |
| ENSSSCG00000012408 | NHSL2 | 1.077695682 | 3.09E-05 | UP |
| ENSSSCG00000015362 | TSPAN13 | 1.077149092 | 0.001032 | UP |
| ENSSSCG00000009431 | DGKH | 1.076542813 | 1.38E-34 | UP |
| ENSSSCG00000040773 | TOB1 | 1.076171367 | 9.2E-125 | UP |
| ENSSSCG00000008553 | PREB | 1.075895017 | 3.83E-55 | UP |
| ENSSSCG00000038872 | GMEB1 | 1.075752642 | 5.54E-46 | UP |
| ENSSSCG00000015603 | LPGAT1 | 1.075115751 | 1.8E-116 | UP |
| ENSSSCG00000003590 | PTPRU | 1.074710895 | 1.93E-89 | UP |
| ENSSSCG00000005268 | RORB | 1.073289681 | 0.008643 | UP |
| ENSSSCG00000006324 | ALDH9A1 | 1.069855767 | 7.1E-127 | UP |
| ENSSSCG00000001473 | COL11A2 | 1.068102735 | 0.001156 | UP |
| ENSSSCG00000014976 | ARHGAP42 | 1.067546421 | 3.05E-43 | UP |
| ENSSSCG00000000293 | ITGA5 | 1.067347395 | 3.02E-66 | UP |
| ENSSSCG00000037278 | NA | 1.067296969 | 5.29E-05 | UP |
| ENSSSCG00000004425 | LAMA4 | 1.067004995 | 7.1E-163 | UP |
| ENSSSCG00000011056 | FRMD4A | 1.065938767 | 1E-133 | UP |
| ENSSSCG00000023870 | SNORD12 | 1.065176549 | 0.001734 | UP |
| ENSSSCG00000027646 | TIPARP | 1.062040392 | 2.84E-41 | UP |
| ENSSSCG00000022413 | TIRAP | 1.059647868 | 1.32E-35 | UP |
| ENSSSCG00000006371 | USF1 | 1.055431967 | 3.24E-87 | UP |
| ENSSSCG00000009049 | USP38 | 1.054554694 | 1.11E-82 | UP |
| ENSSSCG00000015136 | UBASH3B | 1.054394485 | 1.71E-38 | UP |
| ENSSSCG00000010329 | ZMIZ1 | 1.053945447 | 3.47E-40 | UP |
| ENSSSCG00000007043 | GPCPD1 | 1.052492146 | 6.53E-40 | UP |
| ENSSSCG00000012631 | LONRF3 | 1.052437395 | 5.49E-52 | UP |
| ENSSSCG00000010629 | ADRA2A | 1.05196395 | 0.000808 | UP |
| ENSSSCG00000022584 | PPP1R3F | 1.051963617 | 4.91E-05 | UP |
| ENSSSCG00000026931 | SERTAD1 | 1.051806287 | 1.33E-54 | UP |
| ENSSSCG00000005166 | MLLT3 | 1.048970159 | 7.25E-09 | UP |
| ENSSSCG00000032705 | USP42 | 1.047733455 | 2.52E-42 | UP |
| ENSSSCG00000004371 | CRYBG1 | 1.047696384 | 5.8E-128 | UP |
| ENSSSCG00000017601 | TOM1L1 | 1.047390563 | 4.69E-07 | UP |
| ENSSSCG00000009833 | SH2B3 | 1.04694452 | 1.39E-76 | UP |
| ENSSSCG00000038055 | CORO1A | 1.046632175 | 0.003702 | UP |
| ENSSSCG00000013722 | WDR83 | 1.046239965 | 8.5E-36 | UP |
| ENSSSCG00000004138 | HIVEP2 | 1.046030525 | 1.89E-63 | UP |
| ENSSSCG00000040010 | BCL2A1 | 1.044775991 | 3.53E-19 | UP |
| ENSSSCG00000023028 | JAM2 | 1.044423943 | 3.15E-25 | UP |
| ENSSSCG00000015872 | GPD2 | 1.043917385 | 1.94E-52 | UP |
| ENSSSCG00000008347 | FBXO48 | 1.043135513 | 0.000106 | UP |
| ENSSSCG00000000567 | SOX5 | 1.042457919 | 2.42E-34 | UP |
| ENSSSCG00000001805 | WHAMM | 1.041974749 | 3.04E-44 | UP |
| ENSSSCG00000036307 | OAF | 1.041913091 | 1.71E-28 | UP |
| ENSSSCG00000014251 | NA | 1.041312278 | 0.000162 | UP |
| ENSSSCG00000011810 | BCL6 | 1.041040518 | 1E-105 | UP |
| ENSSSCG00000016853 | RICTOR | 1.040348661 | 2.54E-27 | UP |
| ENSSSCG00000032240 | RSPO1 | 1.038538964 | 5.5E-05 | UP |
| ENSSSCG00000016567 | STRIP2 | 1.037208587 | 0.018909 | UP |
| ENSSSCG00000035790 | BTG1 | 1.037141839 | 1.5E-115 | UP |
| ENSSSCG00000001347 | PPP1R10 | 1.034392186 | 1.26E-36 | UP |
| ENSSSCG00000034379 | MAP2K3 | 1.030328517 | 2.5E-108 | UP |
| ENSSSCG00000010340 | FAM213A | 1.02774938 | 1.54E-16 | UP |
| ENSSSCG00000016916 | IL6ST | 1.026857094 | 6.05E-54 | UP |
| ENSSSCG00000011628 | DNAJC13 | 1.026436097 | 9.03E-66 | UP |
| ENSSSCG00000035057 | RUNDC1 | 1.02283523 | 2.68E-39 | UP |
| ENSSSCG00000008368 | UGP2 | 1.018555727 | 1.07E-64 | UP |
| ENSSSCG00000040448 | NA | 1.01789367 | 3.86E-10 | UP |
| ENSSSCG00000009408 | LRCH1 | 1.017723463 | 5.49E-20 | UP |
| ENSSSCG00000017167 | CYTH1 | 1.017108412 | 9.34E-65 | UP |
| ENSSSCG00000002917 | NFKBID | 1.015877259 | 1.38E-15 | UP |
| ENSSSCG00000022895 | CRYBG3 | 1.014132882 | 2.1E-30 | UP |
| ENSSSCG00000035059 | MCM10 | 1.013609982 | 1.2E-54 | UP |
| ENSSSCG00000034887 | HAPLN4 | 1.013061704 | 9.34E-29 | UP |
| ENSSSCG00000025060 | NA | 1.012889005 | 6.76E-66 | UP |
| ENSSSCG00000015846 | RBPMS | 1.010883736 | 1.64E-50 | UP |
| ENSSSCG00000010925 | PPP1R12B | 1.008932061 | 8.85E-21 | UP |
| ENSSSCG00000029592 | GPRC5A | 1.007644228 | 2.36E-12 | UP |
| ENSSSCG00000003897 | MOB3C | 1.006369536 | 2.6E-32 | UP |
| ENSSSCG00000006648 | CTSS | 1.000003863 | 4.76E-26 | UP |
| ENSSSCG00000032692 | SPIN3 | -1.000655299 | 1.45E-15 | DOWN |
| ENSSSCG00000016074 | ANKRD44 | -1.001441862 | 4.91E-19 | DOWN |
| ENSSSCG00000037950 | NIPAL1 | -1.001477701 | 1.21E-24 | DOWN |
| ENSSSCG00000032063 | THEM6 | -1.001691552 | 0.010948 | DOWN |
| ENSSSCG00000016701 | HOXA7 | -1.002042078 | 1.09E-24 | DOWN |
| ENSSSCG00000026746 | NA | -1.003102549 | 7.66E-12 | DOWN |
| ENSSSCG00000027997 | NME5 | -1.004373306 | 0.035948 | DOWN |
| ENSSSCG00000035664 | ZBTB45 | -1.005501873 | 6.25E-17 | DOWN |
| ENSSSCG00000004898 | TNFRSF11A | -1.005657793 | 0.041832 | DOWN |
| ENSSSCG00000020924 | MC1R | -1.006108189 | 0.002701 | DOWN |
| ENSSSCG00000038126 | MGAT3 | -1.008069162 | 0.001866 | DOWN |
| ENSSSCG00000040219 | NA | -1.008904132 | 0.029567 | DOWN |
| ENSSSCG00000032841 | NEAT1_1 | -1.009849184 | 1.84E-06 | DOWN |
| ENSSSCG00000025980 | CHMP4C | -1.009881468 | 8.77E-06 | DOWN |
| ENSSSCG00000009627 | NA | -1.010901016 | 8.77E-21 | DOWN |
| ENSSSCG00000028293 | RCOR3 | -1.011448519 | 1.69E-22 | DOWN |
| ENSSSCG00000025616 | SENP8 | -1.012339386 | 5.97E-07 | DOWN |
| ENSSSCG00000029656 | NDP | -1.012428117 | 6.74E-06 | DOWN |
| ENSSSCG00000028981 | ZNF367 | -1.012835883 | 9.2E-39 | DOWN |
| ENSSSCG00000036482 | ZSWIM7 | -1.012982291 | 3.14E-23 | DOWN |
| ENSSSCG00000010224 | EGR2 | -1.013289217 | 1.57E-07 | DOWN |
| ENSSSCG00000036057 | POLG2 | -1.013373554 | 2.59E-05 | DOWN |
| ENSSSCG00000038569 | ARMCX1 | -1.014417073 | 1.25E-15 | DOWN |
| ENSSSCG00000035369 | RD3 | -1.014492483 | 0.002013 | DOWN |
| ENSSSCG00000039802 | FBXL2 | -1.015473966 | 0.001969 | DOWN |
| ENSSSCG00000017510 | CACNB1 | -1.015669165 | 1.29E-07 | DOWN |
| ENSSSCG00000016448 | KCNH2 | -1.017538615 | 0.000393 | DOWN |
| ENSSSCG00000003989 | NA | -1.017818433 | 0.000677 | DOWN |
| ENSSSCG00000010573 | NA | -1.021177598 | 2.13E-43 | DOWN |
| ENSSSCG00000021082 | NRG2 | -1.021581311 | 0.007307 | DOWN |
| ENSSSCG00000007801 | ZNF48 | -1.022096681 | 3.05E-19 | DOWN |
| ENSSSCG00000015593 | NSL1 | -1.022336777 | 8.37E-13 | DOWN |
| ENSSSCG00000037647 | TFDP2 | -1.022713187 | 2.77E-27 | DOWN |
| ENSSSCG00000015336 | SLC25A13 | -1.023190915 | 1.45E-40 | DOWN |
| ENSSSCG00000029420 | NA | -1.024197104 | 4.07E-07 | DOWN |
| ENSSSCG00000001503 | B3GALT4 | -1.024906126 | 7.34E-08 | DOWN |
| ENSSSCG00000008120 | NA | -1.025363108 | 0.019874 | DOWN |
| ENSSSCG00000021628 | TMEM70 | -1.026944572 | 1.37E-23 | DOWN |
| ENSSSCG00000037023 | NA | -1.027260198 | 0.000702 | DOWN |
| ENSSSCG00000006982 | ZDHHC2 | -1.027481827 | 3.64E-16 | DOWN |
| ENSSSCG00000010559 | NA | -1.030413466 | 0.000251 | DOWN |
| ENSSSCG00000037309 | TIGD7 | -1.031001074 | 1.09E-15 | DOWN |
| ENSSSCG00000017380 | ARL4D | -1.03147568 | 0.000456 | DOWN |
| ENSSSCG00000009079 | INTU | -1.031525656 | 1.18E-11 | DOWN |
| ENSSSCG00000015427 | ORC5 | -1.031806663 | 1.33E-26 | DOWN |
| ENSSSCG00000016206 | CNPPD1 | -1.033481749 | 2.97E-38 | DOWN |
| ENSSSCG00000008360 | CEP68 | -1.033628997 | 4.29E-20 | DOWN |
| ENSSSCG00000036751 | PPM1H | -1.035276693 | 3.71E-19 | DOWN |
| ENSSSCG00000017890 | KIAA0753 | -1.036337564 | 1.15E-50 | DOWN |
| ENSSSCG00000006321 | FAM78B | -1.037509119 | 5.54E-09 | DOWN |
| ENSSSCG00000005128 | TUSC1 | -1.037914566 | 0.003905 | DOWN |
| ENSSSCG00000039056 | GAS7 | -1.038446275 | 9.42E-37 | DOWN |
| ENSSSCG00000036675 | NA | -1.038517445 | 6.76E-18 | DOWN |
| ENSSSCG00000035595 | HMCN1 | -1.040695122 | 4.59E-16 | DOWN |
| ENSSSCG00000012266 | SLC9A7 | -1.04153761 | 0.000527 | DOWN |
| ENSSSCG00000009048 | GAB1 | -1.041552493 | 3.95E-30 | DOWN |
| ENSSSCG00000010031 | NA | -1.041894282 | 9.25E-07 | DOWN |
| ENSSSCG00000006572 | NPR1 | -1.041958419 | 2.76E-08 | DOWN |
| ENSSSCG00000023850 | SDHAF3 | -1.04232371 | 1.94E-09 | DOWN |
| ENSSSCG00000033506 | KCTD21 | -1.042614001 | 3.37E-06 | DOWN |
| ENSSSCG00000026248 | PAQR4 | -1.042839606 | 9.14E-34 | DOWN |
| ENSSSCG00000029392 | HUNK | -1.043897603 | 0.013676 | DOWN |
| ENSSSCG00000023596 | NA | -1.044132974 | 0.000237 | DOWN |
| ENSSSCG00000031102 | C1orf174 | -1.044598153 | 2.92E-20 | DOWN |
| ENSSSCG00000014581 | TUB | -1.0456186 | 3.46E-19 | DOWN |
| ENSSSCG00000040419 | NA | -1.046036725 | 0.009456 | DOWN |
| ENSSSCG00000034563 | NA | -1.046164724 | 0.02299 | DOWN |
| ENSSSCG00000005020 | ATL1 | -1.046312997 | 4.86E-15 | DOWN |
| ENSSSCG00000014904 | DLG2 | -1.047700219 | 4.83E-08 | DOWN |
| ENSSSCG00000017518 | OSBPL7 | -1.048035632 | 1.96E-15 | DOWN |
| ENSSSCG00000035663 | NA | -1.051161824 | 1.61E-05 | DOWN |
| ENSSSCG00000030395 | ASB5 | -1.051912772 | 0.005669 | DOWN |
| ENSSSCG00000020954 | PCGF6 | -1.052742261 | 1.27E-11 | DOWN |
| ENSSSCG00000016086 | C2orf69 | -1.053093764 | 1.42E-21 | DOWN |
| ENSSSCG00000004300 | SMIM8 | -1.055830235 | 4.33E-09 | DOWN |
| ENSSSCG00000034697 | NA | -1.056365346 | 5.17E-39 | DOWN |
| ENSSSCG00000023975 | C15orf52 | -1.056675439 | 5.27E-14 | DOWN |
| ENSSSCG00000037781 | NA | -1.057039893 | 0.004305 | DOWN |
| ENSSSCG00000036396 | NEAT1_2 | -1.057351809 | 1.29E-06 | DOWN |
| ENSSSCG00000016932 | DEPDC1B | -1.057407293 | 9.9E-09 | DOWN |
| ENSSSCG00000027251 | CDC7 | -1.058480453 | 4.47E-22 | DOWN |
| ENSSSCG00000033616 | ZNF10 | -1.05870869 | 3.75E-11 | DOWN |
| ENSSSCG00000006850 | FAM102B | -1.058781165 | 2.49E-58 | DOWN |
| ENSSSCG00000010457 | KIF20B | -1.059345516 | 1.1E-28 | DOWN |
| ENSSSCG00000000079 | FAM83F | -1.059473527 | 9.88E-06 | DOWN |
| ENSSSCG00000031793 | ZNF331 | -1.059581662 | 2.83E-05 | DOWN |
| ENSSSCG00000008574 | KIF3C | -1.059799946 | 8.22E-33 | DOWN |
| ENSSSCG00000031661 | C12orf75 | -1.059940918 | 1.89E-12 | DOWN |
| ENSSSCG00000038044 | GYG2 | -1.06048307 | 2.02E-64 | DOWN |
| ENSSSCG00000033892 | EVA1B | -1.060712256 | 1.33E-41 | DOWN |
| ENSSSCG00000010608 | SFR1 | -1.062160383 | 2.18E-10 | DOWN |
| ENSSSCG00000033305 | PGBD2 | -1.062186463 | 0.003595 | DOWN |
| ENSSSCG00000002490 | GSC | -1.062976882 | 1.45E-06 | DOWN |
| ENSSSCG00000021359 | CDC42EP3 | -1.063768828 | 1.56E-55 | DOWN |
| ENSSSCG00000013426 | MUM1 | -1.063875077 | 9.11E-40 | DOWN |
| ENSSSCG00000011074 | ARHGAP21 | -1.065075322 | 7.94E-67 | DOWN |
| ENSSSCG00000024780 | ZFAND1 | -1.065847784 | 3.22E-25 | DOWN |
| ENSSSCG00000011207 | SGO1 | -1.067275048 | 8.25E-28 | DOWN |
| ENSSSCG00000002792 | HSF4 | -1.068017854 | 0.000129 | DOWN |
| ENSSSCG00000000874 | GAS2L3 | -1.068549088 | 2.79E-39 | DOWN |
| ENSSSCG00000031361 | CELSR1 | -1.068982428 | 7.84E-09 | DOWN |
| ENSSSCG00000010698 | FGFR2 | -1.070221091 | 3.2E-44 | DOWN |
| ENSSSCG00000021027 | PGBD5 | -1.070786648 | 2.9E-08 | DOWN |
| ENSSSCG00000012392 | NA | -1.071923908 | 0.011587 | DOWN |
| ENSSSCG00000009356 | RFXAP | -1.073407979 | 1.63E-29 | DOWN |
| ENSSSCG00000009943 | SSH1 | -1.07411722 | 4.96E-66 | DOWN |
| ENSSSCG00000015947 | DCAF17 | -1.074647022 | 2.95E-11 | DOWN |
| ENSSSCG00000015345 | GLCCI1 | -1.076401992 | 1.77E-17 | DOWN |
| ENSSSCG00000003949 | CDC20 | -1.076487373 | 1.57E-53 | DOWN |
| ENSSSCG00000015010 | EXPH5 | -1.076739622 | 2.45E-05 | DOWN |
| ENSSSCG00000023258 | CLEC4F | -1.078728917 | 0.007307 | DOWN |
| ENSSSCG00000031701 | GXYLT1 | -1.078872319 | 5.94E-40 | DOWN |
| ENSSSCG00000010440 | NA | -1.080657171 | 6.59E-59 | DOWN |
| ENSSSCG00000025120 | BORA | -1.081232258 | 5.86E-23 | DOWN |
| ENSSSCG00000029600 | NA | -1.081275832 | 1.1E-06 | DOWN |
| ENSSSCG00000004247 | ASF1A | -1.081764981 | 2.99E-15 | DOWN |
| ENSSSCG00000013244 | PACSIN3 | -1.081843438 | 4.38E-34 | DOWN |
| ENSSSCG00000028117 | NA | -1.082395727 | 9.87E-28 | DOWN |
| ENSSSCG00000009953 | NA | -1.082490732 | 0.000357 | DOWN |
| ENSSSCG00000006793 | NA | -1.082910268 | 9.45E-05 | DOWN |
| ENSSSCG00000015732 | TFCP2L1 | -1.083024782 | 0.001062 | DOWN |
| ENSSSCG00000007954 | SLX4 | -1.083887138 | 3.64E-29 | DOWN |
| ENSSSCG00000006273 | MCM4 | -1.084889953 | 3.59E-92 | DOWN |
| ENSSSCG00000037580 | ITPRIPL1 | -1.085466977 | 9.66E-29 | DOWN |
| ENSSSCG00000016067 | STK17B | -1.085541658 | 5.17E-31 | DOWN |
| ENSSSCG00000014959 | PIWIL4 | -1.086245143 | 0.002828 | DOWN |
| ENSSSCG00000002651 | CDT1 | -1.086955428 | 1.4E-38 | DOWN |
| ENSSSCG00000011561 | PRRT3 | -1.087379403 | 1.35E-08 | DOWN |
| ENSSSCG00000038933 | PURG | -1.08850089 | 2.48E-15 | DOWN |
| ENSSSCG00000010651 | ABLIM1 | -1.088912116 | 0.000511 | DOWN |
| ENSSSCG00000013012 | SNX15 | -1.08933365 | 1.1E-08 | DOWN |
| ENSSSCG00000010027 | PATZ1 | -1.089595662 | 3.44E-27 | DOWN |
| ENSSSCG00000000376 | SUOX | -1.089814037 | 1.39E-26 | DOWN |
| ENSSSCG00000004245 | FAM184A | -1.089983213 | 6.45E-11 | DOWN |
| ENSSSCG00000000443 | GLI1 | -1.091210706 | 0.020391 | DOWN |
| ENSSSCG00000005286 | CEP78 | -1.092774694 | 3.65E-22 | DOWN |
| ENSSSCG00000032473 | NYNRIN | -1.093195535 | 3.7E-36 | DOWN |
| ENSSSCG00000038852 | NA | -1.093816614 | 4.69E-37 | DOWN |
| ENSSSCG00000000665 | RIMKLB | -1.094058142 | 0.013159 | DOWN |
| ENSSSCG00000003114 | DHX34 | -1.095058139 | 7.73E-43 | DOWN |
| ENSSSCG00000001977 | STXBP6 | -1.096584131 | 4.16E-20 | DOWN |
| ENSSSCG00000025741 | SNX20 | -1.097767178 | 9.29E-16 | DOWN |
| ENSSSCG00000039568 | SNAI2 | -1.097973936 | 1.12E-15 | DOWN |
| ENSSSCG00000005376 | TBC1D2 | -1.098229183 | 5.9E-18 | DOWN |
| ENSSSCG00000015113 | NLRX1 | -1.098269872 | 1.26E-39 | DOWN |
| ENSSSCG00000014356 | DNAJC18 | -1.100524989 | 6.88E-60 | DOWN |
| ENSSSCG00000037159 | MNT | -1.103281063 | 5.92E-26 | DOWN |
| ENSSSCG00000009713 | CLCN3 | -1.105462716 | 1.22E-23 | DOWN |
| ENSSSCG00000005297 | DNAJB5 | -1.10565614 | 1.22E-14 | DOWN |
| ENSSSCG00000013893 | ARRDC2 | -1.105949442 | 3.17E-48 | DOWN |
| ENSSSCG00000001486 | LRRC1 | -1.107465944 | 6.55E-05 | DOWN |
| ENSSSCG00000007807 | CD19 | -1.108822683 | 0.032078 | DOWN |
| ENSSSCG00000004795 | MEIS2 | -1.109027446 | 1.48E-18 | DOWN |
| ENSSSCG00000003324 | NA | -1.110651897 | 2.67E-05 | DOWN |
| ENSSSCG00000000738 | RHNO1 | -1.111057535 | 5.66E-37 | DOWN |
| ENSSSCG00000033788 | OXLD1 | -1.111643387 | 1.44E-11 | DOWN |
| ENSSSCG00000031683 | NA | -1.11282987 | 0.001202 | DOWN |
| ENSSSCG00000007252 | DNMT3B | -1.114542759 | 7.18E-13 | DOWN |
| ENSSSCG00000031875 | ZNF469 | -1.114806227 | 1.72E-27 | DOWN |
| ENSSSCG00000027105 | TUBGCP5 | -1.11496958 | 1.09E-19 | DOWN |
| ENSSSCG00000025417 | BBS2 | -1.116375985 | 4.14E-21 | DOWN |
| ENSSSCG00000024517 | AKAP6 | -1.116638328 | 1.53E-09 | DOWN |
| ENSSSCG00000000275 | MAP3K12 | -1.116653644 | 1.05E-20 | DOWN |
| ENSSSCG00000035284 | BMF | -1.116817689 | 5.81E-08 | DOWN |
| ENSSSCG00000002932 | ZNF567 | -1.118020075 | 5.93E-06 | DOWN |
| ENSSSCG00000023176 | TROAP | -1.119066763 | 5.78E-25 | DOWN |
| ENSSSCG00000016453 | TCAF1 | -1.119100654 | 1.51E-96 | DOWN |
| ENSSSCG00000010912 | KIF14 | -1.119543368 | 3.98E-34 | DOWN |
| ENSSSCG00000038886 | CAGE1 | -1.119569711 | 0.003455 | DOWN |
| ENSSSCG00000005673 | IER5L | -1.119569738 | 2.36E-57 | DOWN |
| ENSSSCG00000023976 | DCP1B | -1.120516318 | 0.000227 | DOWN |
| ENSSSCG00000027916 | PEX10 | -1.120972665 | 4.04E-27 | DOWN |
| ENSSSCG00000001621 | TFEB | -1.123030482 | 6.57E-10 | DOWN |
| ENSSSCG00000001885 | C15orf39 | -1.123378082 | 3.69E-15 | DOWN |
| ENSSSCG00000007680 | MYL10 | -1.12348465 | 0.013754 | DOWN |
| ENSSSCG00000003376 | PLEKHG5 | -1.123504035 | 1.23E-60 | DOWN |
| ENSSSCG00000037669 | PTPN7 | -1.124086846 | 0.001128 | DOWN |
| ENSSSCG00000032541 | TIGD2 | -1.124613585 | 2.52E-25 | DOWN |
| ENSSSCG00000035387 | ZNF550 | -1.125265112 | 0.001353 | DOWN |
| ENSSSCG00000015052 | USP28 | -1.126111292 | 1.26E-41 | DOWN |
| ENSSSCG00000037614 | NEAT1_3 | -1.126118809 | 5.99E-08 | DOWN |
| ENSSSCG00000021203 | PIK3CB | -1.126242601 | 1.21E-14 | DOWN |
| ENSSSCG00000006493 | PMF1 | -1.127444649 | 0.041318 | DOWN |
| ENSSSCG00000032834 | ZNF169 | -1.127574417 | 1.24E-14 | DOWN |
| ENSSSCG00000016589 | LRRC4 | -1.128558396 | 1.5E-07 | DOWN |
| ENSSSCG00000027016 | C19orf47 | -1.129842011 | 8.87E-20 | DOWN |
| ENSSSCG00000013079 | DAGLA | -1.12995561 | 2.02E-27 | DOWN |
| ENSSSCG00000006893 | BCAR3 | -1.131393716 | 4.05E-31 | DOWN |
| ENSSSCG00000001888 | RPP25 | -1.132671256 | 0.000534 | DOWN |
| ENSSSCG00000021741 | ZNF527 | -1.132779162 | 9.32E-06 | DOWN |
| ENSSSCG00000001808 | CPEB1 | -1.133711252 | 3.06E-05 | DOWN |
| ENSSSCG00000004439 | TSPYL4 | -1.133861535 | 1.74E-13 | DOWN |
| ENSSSCG00000017546 | ZNF652 | -1.134261493 | 8.93E-22 | DOWN |
| ENSSSCG00000010456 | PANK1 | -1.135110021 | 2.6E-13 | DOWN |
| ENSSSCG00000006331 | PBX1 | -1.13680087 | 1.22E-35 | DOWN |
| ENSSSCG00000028699 | NEIL2 | -1.13719698 | 6.88E-06 | DOWN |
| ENSSSCG00000023478 | SLC46A1 | -1.138679429 | 2.4E-09 | DOWN |
| ENSSSCG00000015206 | CCDC15 | -1.138910594 | 2.05E-06 | DOWN |
| ENSSSCG00000017471 | CDC6 | -1.139538508 | 2.14E-18 | DOWN |
| ENSSSCG00000038941 | SNAPC5 | -1.141615908 | 3.41E-05 | DOWN |
| ENSSSCG00000027467 | DZIP1L | -1.141670832 | 1.08E-73 | DOWN |
| ENSSSCG00000017508 | STAC2 | -1.142549216 | 5.21E-18 | DOWN |
| ENSSSCG00000039348 | H1F0 | -1.144350666 | 1.77E-35 | DOWN |
| ENSSSCG00000034630 | ABI2 | -1.14484007 | 8.26E-30 | DOWN |
| ENSSSCG00000011787 | MAGEF1 | -1.145872012 | 2.62E-29 | DOWN |
| ENSSSCG00000034027 | ZNF544 | -1.146320661 | 0.000181 | DOWN |
| ENSSSCG00000002346 | PNMA1 | -1.147286148 | 2.27E-11 | DOWN |
| ENSSSCG00000040731 | TMEM65 | -1.149702685 | 1E-41 | DOWN |
| ENSSSCG00000006972 | C8orf48 | -1.149753218 | 4.54E-19 | DOWN |
| ENSSSCG00000039373 | SNX24 | -1.149981371 | 1.58E-19 | DOWN |
| ENSSSCG00000021258 | NA | -1.150060808 | 0.001323 | DOWN |
| ENSSSCG00000025349 | CCDC14 | -1.150135674 | 5.43E-15 | DOWN |
| ENSSSCG00000029408 | ZFP30 | -1.153079895 | 2.95E-21 | DOWN |
| ENSSSCG00000032250 | SRRM3 | -1.153409928 | 0.000398 | DOWN |
| ENSSSCG00000002353 | FAM161B | -1.153767186 | 4.07E-17 | DOWN |
| ENSSSCG00000009774 | C12orf65 | -1.154545152 | 1.59E-13 | DOWN |
| ENSSSCG00000014999 | KBTBD3 | -1.154675048 | 9.94E-12 | DOWN |
| ENSSSCG00000022073 | ZBTB38 | -1.155306956 | 1.68E-92 | DOWN |
| ENSSSCG00000031378 | KBTBD6 | -1.156193255 | 1.34E-25 | DOWN |
| ENSSSCG00000023267 | SCN1B | -1.15661278 | 2.12E-11 | DOWN |
| ENSSSCG00000011768 | CCDC39 | -1.158148434 | 0.015707 | DOWN |
| ENSSSCG00000001975 | PRKD1 | -1.162983877 | 1.78E-21 | DOWN |
| ENSSSCG00000008593 | KLHL29 | -1.163350324 | 1.06E-41 | DOWN |
| ENSSSCG00000010026 | PIK3IP1 | -1.163862105 | 1.54E-12 | DOWN |
| ENSSSCG00000031576 | TMEM42 | -1.164184519 | 1.43E-26 | DOWN |
| ENSSSCG00000012490 | TMEM35A | -1.164471839 | 3.14E-06 | DOWN |
| ENSSSCG00000005204 | RANBP6 | -1.167060266 | 6.25E-47 | DOWN |
| ENSSSCG00000031527 | NA | -1.167217898 | 0.023945 | DOWN |
| ENSSSCG00000032483 | NA | -1.168738574 | 5.05E-11 | DOWN |
| ENSSSCG00000021495 | C3orf33 | -1.168901487 | 5.21E-11 | DOWN |
| ENSSSCG00000015816 | LETM2 | -1.169050315 | 3.65E-07 | DOWN |
| ENSSSCG00000005598 | SCAI | -1.171289246 | 2.03E-07 | DOWN |
| ENSSSCG00000032078 | ZNF362 | -1.172237329 | 1.71E-46 | DOWN |
| ENSSSCG00000022987 | TMEM132E | -1.17295427 | 1.75E-14 | DOWN |
| ENSSSCG00000028372 | FAM131A | -1.173915625 | 2.2E-07 | DOWN |
| ENSSSCG00000007603 | NPTX2 | -1.177702717 | 4.16E-31 | DOWN |
| ENSSSCG00000000399 | TIMELESS | -1.178016839 | 7.46E-29 | DOWN |
| ENSSSCG00000010589 | SFXN2 | -1.178901953 | 1.56E-34 | DOWN |
| ENSSSCG00000040905 | FAAP24 | -1.179222613 | 8.21E-17 | DOWN |
| ENSSSCG00000014187 | NUDT12 | -1.18075867 | 5.1E-12 | DOWN |
| ENSSSCG00000015923 | NOSTRIN | -1.181758242 | 0.002807 | DOWN |
| ENSSSCG00000010561 | TLX1 | -1.182655063 | 5.45E-05 | DOWN |
| ENSSSCG00000040018 | NA | -1.182736246 | 0.00927 | DOWN |
| ENSSSCG00000022280 | DACT3 | -1.183458924 | 3.85E-37 | DOWN |
| ENSSSCG00000005000 | FANCM | -1.184473532 | 8.61E-17 | DOWN |
| ENSSSCG00000029866 | CCDC112 | -1.184726134 | 0.000497 | DOWN |
| ENSSSCG00000034259 | PMEPA1 | -1.184747171 | 3.9E-67 | DOWN |
| ENSSSCG00000009457 | DACH1 | -1.186025136 | 0.02726 | DOWN |
| ENSSSCG00000009867 | TBX5 | -1.18605781 | 3.56E-22 | DOWN |
| ENSSSCG00000035055 | NA | -1.186972549 | 3.26E-13 | DOWN |
| ENSSSCG00000002350 | ELMSAN1 | -1.187383739 | 1.18E-60 | DOWN |
| ENSSSCG00000005657 | PKN3 | -1.18756848 | 1.24E-17 | DOWN |
| ENSSSCG00000024109 | BDH1 | -1.188246071 | 0.000927 | DOWN |
| ENSSSCG00000010761 | STK32C | -1.189611762 | 0.019353 | DOWN |
| ENSSSCG00000023155 | NA | -1.191580965 | 4E-33 | DOWN |
| ENSSSCG00000009468 | KCTD12 | -1.19190964 | 3.01E-30 | DOWN |
| ENSSSCG00000001072 | NA | -1.193299132 | 3.29E-06 | DOWN |
| ENSSSCG00000036839 | NA | -1.193427421 | 1.33E-16 | DOWN |
| ENSSSCG00000011911 | DRD3 | -1.193525285 | 0.010048 | DOWN |
| ENSSSCG00000027325 | TRNP1 | -1.194147713 | 0.040197 | DOWN |
| ENSSSCG00000003876 | CDKN2C | -1.194819214 | 2.33E-95 | DOWN |
| ENSSSCG00000032808 | NA | -1.196212009 | 0.024669 | DOWN |
| ENSSSCG00000002268 | AKAP5 | -1.198046266 | 0.003938 | DOWN |
| ENSSSCG00000020931 | MSH6 | -1.198451077 | 5.14E-63 | DOWN |
| ENSSSCG00000034924 | JRKL | -1.199610195 | 3.33E-22 | DOWN |
| ENSSSCG00000006940 | CYR61 | -1.200277584 | 2.27E-18 | DOWN |
| ENSSSCG00000002838 | ZNF423 | -1.200943317 | 6.12E-27 | DOWN |
| ENSSSCG00000006466 | SH2D2A | -1.202238471 | 1.08E-06 | DOWN |
| ENSSSCG00000024134 | MGLL | -1.202760943 | 1.84E-24 | DOWN |
| ENSSSCG00000003610 | IQCC | -1.203734959 | 9.44E-30 | DOWN |
| ENSSSCG00000010896 | ASPM | -1.203937366 | 1.41E-53 | DOWN |
| ENSSSCG00000035971 | DUSP2 | -1.204885129 | 2.65E-08 | DOWN |
| ENSSSCG00000007675 | EPHB4 | -1.205226763 | 1.69E-40 | DOWN |
| ENSSSCG00000034191 | SOX6 | -1.206692939 | 1.77E-32 | DOWN |
| ENSSSCG00000003326 | NA | -1.206794306 | 3.76E-09 | DOWN |
| ENSSSCG00000040339 | ZFHX2 | -1.206798379 | 0.00528 | DOWN |
| ENSSSCG00000037413 | NA | -1.206940381 | 2.11E-14 | DOWN |
| ENSSSCG00000003988 | NA | -1.20807317 | 0.0002 | DOWN |
| ENSSSCG00000011732 | TRIM59 | -1.209094703 | 7.99E-42 | DOWN |
| ENSSSCG00000010980 | C9orf24 | -1.209254669 | 0.049738 | DOWN |
| ENSSSCG00000016157 | NA | -1.209503998 | 9.63E-14 | DOWN |
| ENSSSCG00000016941 | RNF180 | -1.209758349 | 0.019115 | DOWN |
| ENSSSCG00000003341 | TAS1R3 | -1.211907552 | 1.73E-16 | DOWN |
| ENSSSCG00000002937 | ZNF420 | -1.212430107 | 1.83E-13 | DOWN |
| ENSSSCG00000004475 | NA | -1.212522901 | 2.93E-46 | DOWN |
| ENSSSCG00000035755 | SHISA8 | -1.213124364 | 0.011947 | DOWN |
| ENSSSCG00000000739 | FOXM1 | -1.213209438 | 2.72E-49 | DOWN |
| ENSSSCG00000022347 | WNT11 | -1.213402668 | 2.98E-07 | DOWN |
| ENSSSCG00000009968 | TTC28 | -1.213903617 | 4.01E-59 | DOWN |
| ENSSSCG00000021749 | MCF2L2 | -1.21455391 | 1.4E-14 | DOWN |
| ENSSSCG00000035527 | NA | -1.214682202 | 9.91E-10 | DOWN |
| ENSSSCG00000032443 | KIAA0895L | -1.216182238 | 3.2E-19 | DOWN |
| ENSSSCG00000040400 | GPR19 | -1.217528929 | 1.67E-18 | DOWN |
| ENSSSCG00000012283 | ZNF81 | -1.220232343 | 2.3E-17 | DOWN |
| ENSSSCG00000014157 | NR2F1 | -1.222138994 | 4.59E-07 | DOWN |
| ENSSSCG00000040334 | CBX6 | -1.222900746 | 7.63E-87 | DOWN |
| ENSSSCG00000040418 | FSIP1 | -1.223397026 | 5.71E-06 | DOWN |
| ENSSSCG00000005971 | ZNF572 | -1.225686836 | 1.19E-05 | DOWN |
| ENSSSCG00000012652 | SASH3 | -1.228377169 | 0.006987 | DOWN |
| ENSSSCG00000011075 | KIAA1217 | -1.229033244 | 5.78E-07 | DOWN |
| ENSSSCG00000014998 | AASDHPPT | -1.229341429 | 3.59E-44 | DOWN |
| ENSSSCG00000012150 | REPS2 | -1.231654545 | 0.01123 | DOWN |
| ENSSSCG00000036428 | NA | -1.232053987 | 1.3E-41 | DOWN |
| ENSSSCG00000001710 | RUNX2 | -1.233031343 | 5.61E-05 | DOWN |
| ENSSSCG00000000203 | KCNH3 | -1.234913715 | 0.01477 | DOWN |
| ENSSSCG00000006889 | ARHGAP29 | -1.235285534 | 3.8E-66 | DOWN |
| ENSSSCG00000034987 | IQANK1 | -1.235513383 | 0.011814 | DOWN |
| ENSSSCG00000004390 | SESN1 | -1.236886296 | 2.62E-22 | DOWN |
| ENSSSCG00000037499 | NEDD4 | -1.240434933 | 6.1E-49 | DOWN |
| ENSSSCG00000010108 | KLHL22 | -1.240664924 | 2.08E-42 | DOWN |
| ENSSSCG00000026108 | CDC42EP1 | -1.241092153 | 5.98E-47 | DOWN |
| ENSSSCG00000000523 | BBS10 | -1.242097339 | 3E-07 | DOWN |
| ENSSSCG00000009963 | TPST2 | -1.242580187 | 2.41E-19 | DOWN |
| ENSSSCG00000023956 | NA | -1.24472644 | 5.54E-83 | DOWN |
| ENSSSCG00000005052 | WDHD1 | -1.246250629 | 1.4E-31 | DOWN |
| ENSSSCG00000022692 | RINL | -1.248062609 | 0.021626 | DOWN |
| ENSSSCG00000007033 | AP3M2 | -1.248249894 | 0.000489 | DOWN |
| ENSSSCG00000017392 | CCR10 | -1.248258145 | 3.8E-09 | DOWN |
| ENSSSCG00000010644 | DCLRE1A | -1.248278132 | 1.88E-21 | DOWN |
| ENSSSCG00000025542 | NEMP1 | -1.248390836 | 6.29E-20 | DOWN |
| ENSSSCG00000017226 | FADS6 | -1.250236343 | 0.043836 | DOWN |
| ENSSSCG00000004782 | BUB1B | -1.25044288 | 4.68E-43 | DOWN |
| ENSSSCG00000022664 | ZSCAN16 | -1.25056173 | 8.76E-07 | DOWN |
| ENSSSCG00000021436 | DFFB | -1.252533114 | 2.11E-14 | DOWN |
| ENSSSCG00000002777 | HSD11B2 | -1.253122932 | 0.015834 | DOWN |
| ENSSSCG00000011496 | ADAMTS9 | -1.253457124 | 1.27E-69 | DOWN |
| ENSSSCG00000009037 | ZNF827 | -1.254873395 | 6.53E-56 | DOWN |
| ENSSSCG00000004598 | GCOM1 | -1.254908688 | 0.008148 | DOWN |
| ENSSSCG00000033189 | FAM107A | -1.255261066 | 0.035093 | DOWN |
| ENSSSCG00000000730 | PRMT8 | -1.259646991 | 0.028038 | DOWN |
| ENSSSCG00000015944 | TLK1 | -1.261847453 | 1.32E-74 | DOWN |
| ENSSSCG00000017382 | EZH1 | -1.262134065 | 3.24E-14 | DOWN |
| ENSSSCG00000013351 | NAV2 | -1.263400415 | 5.74E-57 | DOWN |
| ENSSSCG00000010076 | ZNF70 | -1.264467089 | 3.44E-07 | DOWN |
| ENSSSCG00000007350 | PPP1R16B | -1.267671662 | 0.000166 | DOWN |
| ENSSSCG00000036373 | NA | -1.268475512 | 0.006363 | DOWN |
| ENSSSCG00000015645 | NA | -1.268531012 | 2.68E-61 | DOWN |
| ENSSSCG00000035388 | C16orf46 | -1.270290383 | 0.012178 | DOWN |
| ENSSSCG00000003628 | CLSPN | -1.270402842 | 1.08E-39 | DOWN |
| ENSSSCG00000036356 | ZSCAN2 | -1.271603267 | 2.17E-09 | DOWN |
| ENSSSCG00000004309 | CNR1 | -1.271832148 | 0.042231 | DOWN |
| ENSSSCG00000017195 | TRIM65 | -1.271842643 | 1.46E-06 | DOWN |
| ENSSSCG00000014163 | SLF1 | -1.272308619 | 2.21E-18 | DOWN |
| ENSSSCG00000013320 | PAX6 | -1.273447492 | 7.19E-07 | DOWN |
| ENSSSCG00000033456 | GPR157 | -1.275405099 | 5.42E-06 | DOWN |
| ENSSSCG00000025335 | ZSCAN31 | -1.275966773 | 2.17E-14 | DOWN |
| ENSSSCG00000011215 | OXSM | -1.276372955 | 1.16E-24 | DOWN |
| ENSSSCG00000014900 | RAB30 | -1.27807235 | 7.49E-11 | DOWN |
| ENSSSCG00000029778 | SPATA17 | -1.279172543 | 0.035546 | DOWN |
| ENSSSCG00000022026 | AIPL1 | -1.280333316 | 2.87E-06 | DOWN |
| ENSSSCG00000021638 | NEU3 | -1.280679659 | 6.45E-20 | DOWN |
| ENSSSCG00000012824 | GAB3 | -1.282025225 | 6.73E-05 | DOWN |
| ENSSSCG00000034551 | SCX | -1.282797887 | 4.5E-66 | DOWN |
| ENSSSCG00000027767 | FAM35A | -1.283512047 | 0.020446 | DOWN |
| ENSSSCG00000003076 | NA | -1.285759809 | 2.26E-13 | DOWN |
| ENSSSCG00000010012 | SLC35E4 | -1.287455437 | 1.81E-15 | DOWN |
| ENSSSCG00000030680 | TCF7 | -1.288770019 | 1.25E-95 | DOWN |
| ENSSSCG00000000682 | GNB3 | -1.290311196 | 1.44E-05 | DOWN |
| ENSSSCG00000012882 | NA | -1.290574866 | 3.17E-06 | DOWN |
| ENSSSCG00000038902 | KCNK6 | -1.291127324 | 5.6E-19 | DOWN |
| ENSSSCG00000031849 | NA | -1.291629557 | 1.22E-15 | DOWN |
| ENSSSCG00000035739 | SLC26A1 | -1.292195084 | 0.032443 | DOWN |
| ENSSSCG00000021138 | CEP250 | -1.292964202 | 1.37E-39 | DOWN |
| ENSSSCG00000010316 | KAT6B | -1.294768601 | 1.83E-60 | DOWN |
| ENSSSCG00000040652 | CBLN1 | -1.298131017 | 4.25E-06 | DOWN |
| ENSSSCG00000023776 | CCSAP | -1.298440622 | 3.01E-28 | DOWN |
| ENSSSCG00000002424 | SPATA7 | -1.298874648 | 4.37E-10 | DOWN |
| ENSSSCG00000033792 | NA | -1.299069525 | 5.35E-28 | DOWN |
| ENSSSCG00000017112 | IRX4 | -1.302215121 | 9.13E-06 | DOWN |
| ENSSSCG00000016707 | HOXA1 | -1.303193779 | 2.38E-05 | DOWN |
| ENSSSCG00000017053 | NIPAL4 | -1.303670469 | 3.82E-09 | DOWN |
| ENSSSCG00000033213 | LYRM7 | -1.303805936 | 1.24E-07 | DOWN |
| ENSSSCG00000006810 | KCNC4 | -1.304420692 | 4.42E-06 | DOWN |
| ENSSSCG00000008601 | SDC1 | -1.305531645 | 5.89E-59 | DOWN |
| ENSSSCG00000011439 | PHF7 | -1.307614792 | 8.92E-05 | DOWN |
| ENSSSCG00000009357 | SMAD9 | -1.307858496 | 2.56E-62 | DOWN |
| ENSSSCG00000027505 | ESYT3 | -1.30824022 | 0.000223 | DOWN |
| ENSSSCG00000015982 | HOXD9 | -1.30888402 | 1.86E-30 | DOWN |
| ENSSSCG00000039425 | BPGM | -1.308916883 | 1.39E-59 | DOWN |
| ENSSSCG00000001573 | PIM1 | -1.310721875 | 1.23E-40 | DOWN |
| ENSSSCG00000002930 | ZNF260 | -1.310938734 | 6.22E-33 | DOWN |
| ENSSSCG00000028274 | FAM131B | -1.31157325 | 0.00136 | DOWN |
| ENSSSCG00000010416 | ZNF32 | -1.311633066 | 6.06E-14 | DOWN |
| ENSSSCG00000000160 | PRDM4 | -1.312095613 | 1.38E-69 | DOWN |
| ENSSSCG00000031976 | ANKRD53 | -1.312788643 | 0.002128 | DOWN |
| ENSSSCG00000008892 | NA | -1.315614456 | 7.17E-24 | DOWN |
| ENSSSCG00000040875 | ZFPM1 | -1.31608443 | 7.09E-08 | DOWN |
| ENSSSCG00000027790 | NA | -1.316342312 | 0.000437 | DOWN |
| ENSSSCG00000014822 | ARHGEF17 | -1.316395312 | 7.35E-78 | DOWN |
| ENSSSCG00000032632 | CMTM4 | -1.317244168 | 3.87E-46 | DOWN |
| ENSSSCG00000015880 | TANC1 | -1.318360641 | 8.12E-71 | DOWN |
| ENSSSCG00000026116 | FHOD1 | -1.31840547 | 5.94E-65 | DOWN |
| ENSSSCG00000021640 | ZNF354C | -1.319357414 | 6.39E-10 | DOWN |
| ENSSSCG00000003473 | RSG1 | -1.320944683 | 3.03E-09 | DOWN |
| ENSSSCG00000038186 | TIGD4 | -1.320970539 | 0.018081 | DOWN |
| ENSSSCG00000038095 | NA | -1.323312264 | 3.15E-18 | DOWN |
| ENSSSCG00000026710 | CARHSP1 | -1.323911202 | 2.6E-146 | DOWN |
| ENSSSCG00000009243 | THAP9 | -1.326225158 | 1.86E-29 | DOWN |
| ENSSSCG00000022462 | ZNF852 | -1.326730192 | 0.001029 | DOWN |
| ENSSSCG00000035495 | KITLG | -1.328318597 | 9.26E-29 | DOWN |
| ENSSSCG00000008613 | GEN1 | -1.331122448 | 6.07E-12 | DOWN |
| ENSSSCG00000012944 | PELI3 | -1.332266593 | 3.11E-16 | DOWN |
| ENSSSCG00000000040 | SEPT3 | -1.333520312 | 2.36E-07 | DOWN |
| ENSSSCG00000036452 | LRRC17 | -1.334183621 | 4.83E-15 | DOWN |
| ENSSSCG00000000075 | MKL1 | -1.335489199 | 3E-147 | DOWN |
| ENSSSCG00000026326 | CCNF | -1.337123872 | 4.1E-78 | DOWN |
| ENSSSCG00000029257 | NA | -1.337693319 | 0.00015 | DOWN |
| ENSSSCG00000000936 | SLC6A15 | -1.338717479 | 3.69E-25 | DOWN |
| ENSSSCG00000020987 | WDR31 | -1.340803284 | 3.22E-07 | DOWN |
| ENSSSCG00000008874 | TMEM144 | -1.341380782 | 1.16E-05 | DOWN |
| ENSSSCG00000029949 | CD248 | -1.342647951 | 2.7E-45 | DOWN |
| ENSSSCG00000037900 | CCND1 | -1.34386075 | 6.5E-149 | DOWN |
| ENSSSCG00000011326 | PTH1R | -1.344304559 | 3.36E-20 | DOWN |
| ENSSSCG00000009351 | MAB21L1 | -1.345575019 | 4.86E-15 | DOWN |
| ENSSSCG00000002348 | NA | -1.348462599 | 1.67E-23 | DOWN |
| ENSSSCG00000035456 | WWC1 | -1.349214911 | 0.00013 | DOWN |
| ENSSSCG00000030005 | LGALSL | -1.35072981 | 5.33E-66 | DOWN |
| ENSSSCG00000006101 | NA | -1.351158969 | 6.34E-09 | DOWN |
| ENSSSCG00000024158 | ANO1 | -1.351910433 | 1.69E-35 | DOWN |
| ENSSSCG00000006664 | MTMR11 | -1.352286484 | 6.54E-17 | DOWN |
| ENSSSCG00000007949 | SRL | -1.353436953 | 0.00014 | DOWN |
| ENSSSCG00000038541 | TMEM60 | -1.357213026 | 7.83E-46 | DOWN |
| ENSSSCG00000014406 | PRELID2 | -1.357369238 | 1.67E-15 | DOWN |
| ENSSSCG00000036787 | APOLD1 | -1.357484849 | 0.00056 | DOWN |
| ENSSSCG00000030294 | ZNF398 | -1.360231916 | 1.95E-36 | DOWN |
| ENSSSCG00000016625 | CTTNBP2 | -1.360825357 | 2.45E-16 | DOWN |
| ENSSSCG00000005630 | NAIF1 | -1.361392357 | 4.68E-05 | DOWN |
| ENSSSCG00000013276 | PRDM11 | -1.363272038 | 1.95E-06 | DOWN |
| ENSSSCG00000038469 | NA | -1.365936375 | 6.95E-55 | DOWN |
| ENSSSCG00000014385 | PCDHGC5 | -1.366886681 | 9.62E-13 | DOWN |
| ENSSSCG00000004082 | NA | -1.367657528 | 1.5E-31 | DOWN |
| ENSSSCG00000029165 | DOK4 | -1.368596074 | 9.79E-37 | DOWN |
| ENSSSCG00000009122 | ARSJ | -1.369775138 | 1.46E-82 | DOWN |
| ENSSSCG00000035788 | NA | -1.371390991 | 1.37E-05 | DOWN |
| ENSSSCG00000029796 | KBTBD11 | -1.372165083 | 0.003218 | DOWN |
| ENSSSCG00000015492 | DARS2 | -1.372185579 | 1.51E-27 | DOWN |
| ENSSSCG00000035968 | LMX1B | -1.373227071 | 7.64E-07 | DOWN |
| ENSSSCG00000028606 | ZBTB7B | -1.375057056 | 1.8E-108 | DOWN |
| ENSSSCG00000021440 | GPSM2 | -1.377091411 | 1.05E-55 | DOWN |
| ENSSSCG00000022333 | C16orf87 | -1.379823379 | 2.01E-21 | DOWN |
| ENSSSCG00000040037 | MTSS1L | -1.3880092 | 8.44E-69 | DOWN |
| ENSSSCG00000040215 | TFAP4 | -1.389754043 | 1.75E-13 | DOWN |
| ENSSSCG00000036052 | NA | -1.390482382 | 3.36E-05 | DOWN |
| ENSSSCG00000039609 | ANKRD33B | -1.390485023 | 4.1E-65 | DOWN |
| ENSSSCG00000036098 | NKX6-2 | -1.390792293 | 0.014751 | DOWN |
| ENSSSCG00000026346 | UBE3D | -1.391226627 | 4E-10 | DOWN |
| ENSSSCG00000022636 | DENND5B | -1.392483965 | 9.87E-32 | DOWN |
| ENSSSCG00000016506 | KLRG2 | -1.392953351 | 0.002444 | DOWN |
| ENSSSCG00000034471 | PSORS1C2 | -1.393991074 | 0.017047 | DOWN |
| ENSSSCG00000037995 | NA | -1.394310989 | 0.021437 | DOWN |
| ENSSSCG00000035798 | PRTG | -1.394646631 | 4.38E-05 | DOWN |
| ENSSSCG00000018758 | ssc-mir-214 | -1.394928054 | 4.73E-09 | DOWN |
| ENSSSCG00000018029 | NA | -1.395996903 | 0.000178 | DOWN |
| ENSSSCG00000022988 | TSPEAR | -1.398105146 | 0.005119 | DOWN |
| ENSSSCG00000021204 | HOXA10 | -1.401762759 | 4.26E-30 | DOWN |
| ENSSSCG00000035895 | JDP2 | -1.40379646 | 1.92E-55 | DOWN |
| ENSSSCG00000033000 | TIMP4 | -1.407044801 | 0.011845 | DOWN |
| ENSSSCG00000027970 | NA | -1.407923589 | 2.08E-08 | DOWN |
| ENSSSCG00000004490 | SETBP1 | -1.40864407 | 1.73E-34 | DOWN |
| ENSSSCG00000040067 | ZNF835 | -1.411034184 | 0.019863 | DOWN |
| ENSSSCG00000005008 | POLE2 | -1.413497939 | 9.12E-14 | DOWN |
| ENSSSCG00000007304 | NA | -1.414321248 | 0.01393 | DOWN |
| ENSSSCG00000005475 | ZFP37 | -1.414451527 | 0.001002 | DOWN |
| ENSSSCG00000011888 | GPR156 | -1.418008932 | 9.31E-29 | DOWN |
| ENSSSCG00000030585 | HOXC6 | -1.419260885 | 0.001353 | DOWN |
| ENSSSCG00000015742 | TMEM177 | -1.421665208 | 5.09E-09 | DOWN |
| ENSSSCG00000005308 | RUSC2 | -1.422040532 | 2.9E-161 | DOWN |
| ENSSSCG00000012912 | TBC1D10C | -1.422134434 | 0.011597 | DOWN |
| ENSSSCG00000007940 | NA | -1.4228009 | 2.91E-05 | DOWN |
| ENSSSCG00000001646 | BICRAL | -1.424755736 | 5E-71 | DOWN |
| ENSSSCG00000016703 | HOXA5 | -1.424883547 | 5.3E-78 | DOWN |
| ENSSSCG00000022728 | HOXC10 | -1.425415607 | 0.000868 | DOWN |
| ENSSSCG00000016531 | C7orf49 | -1.426827623 | 3.12E-38 | DOWN |
| ENSSSCG00000016841 | SLC1A3 | -1.430021745 | 8.38E-30 | DOWN |
| ENSSSCG00000034181 | NKX3-2 | -1.430047594 | 2.35E-05 | DOWN |
| ENSSSCG00000036030 | FAM212B | -1.431010206 | 5.25E-44 | DOWN |
| ENSSSCG00000031744 | NA | -1.432358474 | 1.25E-19 | DOWN |
| ENSSSCG00000039627 | TMEM200C | -1.43819826 | 9.45E-05 | DOWN |
| ENSSSCG00000016983 | STC2 | -1.438352054 | 5.93E-25 | DOWN |
| ENSSSCG00000000111 | BAIAP2L2 | -1.443247077 | 5.44E-15 | DOWN |
| ENSSSCG00000038296 | NA | -1.44430754 | 1.02E-05 | DOWN |
| ENSSSCG00000003471 | EPHA2 | -1.446795326 | 1.46E-63 | DOWN |
| ENSSSCG00000027869 | PHF13 | -1.447206099 | 1.02E-57 | DOWN |
| ENSSSCG00000027206 | PARD6B | -1.448887667 | 0.014282 | DOWN |
| ENSSSCG00000015523 | RALGPS2 | -1.452732059 | 2.76E-05 | DOWN |
| ENSSSCG00000021704 | PTPDC1 | -1.452763811 | 1.19E-13 | DOWN |
| ENSSSCG00000012637 | KLHL13 | -1.453150915 | 0.004375 | DOWN |
| ENSSSCG00000035104 | TERB1 | -1.453171144 | 0.015384 | DOWN |
| ENSSSCG00000031764 | NA | -1.453363283 | 3.07E-10 | DOWN |
| ENSSSCG00000024205 | ARHGEF26 | -1.454496074 | 4.99E-06 | DOWN |
| ENSSSCG00000000530 | FGD4 | -1.455036364 | 4.31E-25 | DOWN |
| ENSSSCG00000001872 | LINGO1 | -1.455322202 | 1.91E-09 | DOWN |
| ENSSSCG00000007454 | NA | -1.457135239 | 2.9E-99 | DOWN |
| ENSSSCG00000017041 | ADRA1B | -1.457204642 | 1.73E-05 | DOWN |
| ENSSSCG00000011618 | GATA2 | -1.459515515 | 3.13E-18 | DOWN |
| ENSSSCG00000012591 | AMOT | -1.45959265 | 1.5E-113 | DOWN |
| ENSSSCG00000025527 | NA | -1.460481981 | 0.008919 | DOWN |
| ENSSSCG00000029039 | BRCA2 | -1.462060375 | 1.05E-39 | DOWN |
| ENSSSCG00000033032 | ZBTB14 | -1.463542437 | 1.42E-27 | DOWN |
| ENSSSCG00000039161 | MEIS1 | -1.465966839 | 2.99E-17 | DOWN |
| ENSSSCG00000036933 | NR1D1 | -1.468886627 | 6.35E-22 | DOWN |
| ENSSSCG00000009605 | GFRA2 | -1.470089556 | 2.86E-25 | DOWN |
| ENSSSCG00000004388 | NA | -1.470348418 | 0.019023 | DOWN |
| ENSSSCG00000007531 | FAM217B | -1.470832118 | 1.06E-15 | DOWN |
| ENSSSCG00000034283 | NA | -1.471884597 | 0.028363 | DOWN |
| ENSSSCG00000001247 | ZFP57 | -1.472611693 | 0.002492 | DOWN |
| ENSSSCG00000016451 | NA | -1.473044678 | 1.39E-08 | DOWN |
| ENSSSCG00000035074 | FOXO6 | -1.473859299 | 0.026395 | DOWN |
| ENSSSCG00000013344 | ANO5 | -1.474753494 | 0.010505 | DOWN |
| ENSSSCG00000000934 | LRRIQ1 | -1.476329646 | 0.018496 | DOWN |
| ENSSSCG00000020934 | NA | -1.476356062 | 0.000222 | DOWN |
| ENSSSCG00000009232 | NKX6-1 | -1.476428319 | 6.05E-05 | DOWN |
| ENSSSCG00000015961 | CDCA7 | -1.477328378 | 2.31E-73 | DOWN |
| ENSSSCG00000007073 | ISM1 | -1.48169202 | 9.7E-192 | DOWN |
| ENSSSCG00000016873 | NIM1K | -1.481880351 | 3.47E-09 | DOWN |
| ENSSSCG00000008197 | SEMA4C | -1.482756872 | 6.56E-36 | DOWN |
| ENSSSCG00000019359 | NA | -1.484778871 | 0.000148 | DOWN |
| ENSSSCG00000038492 | FAM109B | -1.484941878 | 8.9E-100 | DOWN |
| ENSSSCG00000037406 | TMEM136 | -1.485364294 | 1.54E-08 | DOWN |
| ENSSSCG00000039875 | NKD1 | -1.486589965 | 3.02E-26 | DOWN |
| ENSSSCG00000003238 | SPACA6 | -1.492530201 | 0.00683 | DOWN |
| ENSSSCG00000010170 | DISC1 | -1.494003865 | 0.001537 | DOWN |
| ENSSSCG00000040730 | NA | -1.494556688 | 0.000824 | DOWN |
| ENSSSCG00000005506 | MEGF9 | -1.497183858 | 4.8E-65 | DOWN |
| ENSSSCG00000030337 | NYAP1 | -1.497622232 | 0.000145 | DOWN |
| ENSSSCG00000015985 | HOXD3 | -1.498499051 | 4.74E-09 | DOWN |
| ENSSSCG00000031713 | H2AFY2 | -1.498963915 | 2.09E-08 | DOWN |
| ENSSSCG00000016659 | KIAA0895 | -1.50057386 | 3.9E-12 | DOWN |
| ENSSSCG00000011430 | DUSP7 | -1.503732276 | 1.6E-120 | DOWN |
| ENSSSCG00000040798 | NA | -1.504032839 | 1.39E-05 | DOWN |
| ENSSSCG00000024431 | ATG9B | -1.506612306 | 0.023033 | DOWN |
| ENSSSCG00000033718 | XIST_intron | -1.50713502 | 6.45E-05 | DOWN |
| ENSSSCG00000015144 | GRAMD1B | -1.507708457 | 8.66E-08 | DOWN |
| ENSSSCG00000007198 | ANGPT4 | -1.508823559 | 0.041978 | DOWN |
| ENSSSCG00000038732 | AGAP2 | -1.509726013 | 0.001458 | DOWN |
| ENSSSCG00000033800 | PELI2 | -1.511228434 | 3.04E-09 | DOWN |
| ENSSSCG00000013718 | NA | -1.512809932 | 0.002784 | DOWN |
| ENSSSCG00000039101 | C1QL1 | -1.514320339 | 0.019117 | DOWN |
| ENSSSCG00000007530 | PPP1R3D | -1.518013653 | 1.52E-25 | DOWN |
| ENSSSCG00000017892 | PITPNM3 | -1.519018569 | 4.81E-09 | DOWN |
| ENSSSCG00000003112 | NA | -1.521071334 | 9.96E-12 | DOWN |
| ENSSSCG00000031819 | TP53I11 | -1.521719509 | 4.64E-40 | DOWN |
| ENSSSCG00000003644 | FHL3 | -1.522018477 | 4.5E-166 | DOWN |
| ENSSSCG00000024396 | NOVA1 | -1.526604075 | 1.52E-17 | DOWN |
| ENSSSCG00000031589 | NA | -1.52677176 | 4.84E-16 | DOWN |
| ENSSSCG00000021273 | NA | -1.526821447 | 2.14E-10 | DOWN |
| ENSSSCG00000002900 | NA | -1.5271676 | 8.94E-20 | DOWN |
| ENSSSCG00000008422 | NA | -1.527452003 | 3.7E-115 | DOWN |
| ENSSSCG00000009067 | PABPC4L | -1.527483158 | 4.16E-06 | DOWN |
| ENSSSCG00000036261 | CROCC2 | -1.527542145 | 2.91E-07 | DOWN |
| ENSSSCG00000000791 | PDZRN4 | -1.528563697 | 1.46E-05 | DOWN |
| ENSSSCG00000035267 | NA | -1.529820878 | 1.29E-07 | DOWN |
| ENSSSCG00000035222 | CHST8 | -1.531237341 | 3.64E-10 | DOWN |
| ENSSSCG00000006890 | ABCA4 | -1.537218818 | 0.020051 | DOWN |
| ENSSSCG00000037016 | ID1 | -1.537244794 | 6.17E-35 | DOWN |
| ENSSSCG00000000789 | CNTN1 | -1.538422954 | 0.012155 | DOWN |
| ENSSSCG00000022611 | CAPRIN2 | -1.541937323 | 1.91E-12 | DOWN |
| ENSSSCG00000034008 | NA | -1.5424476 | 1.93E-05 | DOWN |
| ENSSSCG00000026961 | NA | -1.54246556 | 4.13E-05 | DOWN |
| ENSSSCG00000033759 | TBXA2R | -1.549503106 | 5.24E-07 | DOWN |
| ENSSSCG00000039838 | RGS10 | -1.550967665 | 5.28E-24 | DOWN |
| ENSSSCG00000013897 | RAB3A | -1.555663321 | 5.14E-11 | DOWN |
| ENSSSCG00000024663 | SPICE1 | -1.557905143 | 1.01E-12 | DOWN |
| ENSSSCG00000038600 | PRSS8 | -1.562286572 | 0.017929 | DOWN |
| ENSSSCG00000000411 | NAB2 | -1.563024501 | 1E-112 | DOWN |
| ENSSSCG00000032831 | BRI3BP | -1.563485274 | 8.53E-44 | DOWN |
| ENSSSCG00000037391 | NA | -1.566381352 | 2.09E-18 | DOWN |
| ENSSSCG00000015120 | USP2 | -1.567708094 | 1.28E-20 | DOWN |
| ENSSSCG00000026130 | EPHA3 | -1.56977909 | 0.004678 | DOWN |
| ENSSSCG00000038220 | RXRA | -1.570647501 | 2.59E-96 | DOWN |
| ENSSSCG00000016838 | RANBP3L | -1.574419262 | 0.005378 | DOWN |
| ENSSSCG00000014448 | ARSI | -1.575182447 | 4.77E-50 | DOWN |
| ENSSSCG00000028192 | DNAJC28 | -1.575460047 | 1E-08 | DOWN |
| ENSSSCG00000025260 | CARD10 | -1.576224004 | 1.14E-37 | DOWN |
| ENSSSCG00000032156 | GPR146 | -1.576658514 | 0.004816 | DOWN |
| ENSSSCG00000035810 | NA | -1.57783823 | 0.000132 | DOWN |
| ENSSSCG00000005975 | MTSS1 | -1.577978885 | 1.48E-08 | DOWN |
| ENSSSCG00000040698 | PRR7 | -1.581249167 | 1.06E-05 | DOWN |
| ENSSSCG00000031072 | ZNF514 | -1.586884275 | 2.14E-19 | DOWN |
| ENSSSCG00000001867 | PSTPIP1 | -1.589232473 | 0.016273 | DOWN |
| ENSSSCG00000016217 | DNAJB2 | -1.589238929 | 1.49E-48 | DOWN |
| ENSSSCG00000033351 | NA | -1.590077313 | 4.92E-09 | DOWN |
| ENSSSCG00000007356 | PLCG1 | -1.595322364 | 1.3E-149 | DOWN |
| ENSSSCG00000033809 | IFITM10 | -1.59755866 | 0.000193 | DOWN |
| ENSSSCG00000002847 | GPT2 | -1.60024194 | 7.14E-30 | DOWN |
| ENSSSCG00000022797 | PPP1R3B | -1.602328165 | 1.19E-37 | DOWN |
| ENSSSCG00000015346 | ICA1 | -1.605853045 | 9.11E-15 | DOWN |
| ENSSSCG00000035448 | SS18L1 | -1.610806338 | 3.94E-27 | DOWN |
| ENSSSCG00000010653 | TRUB1 | -1.611627519 | 1.21E-05 | DOWN |
| ENSSSCG00000037480 | NA | -1.611975812 | 5.72E-05 | DOWN |
| ENSSSCG00000011914 | ZDHHC23 | -1.613411158 | 1.12E-28 | DOWN |
| ENSSSCG00000011217 | NEK10 | -1.613887122 | 1.1E-12 | DOWN |
| ENSSSCG00000011404 | HYAL1 | -1.614622848 | 6.49E-37 | DOWN |
| ENSSSCG00000008720 | HMX1 | -1.617941162 | 2.42E-05 | DOWN |
| ENSSSCG00000030018 | ZNF396 | -1.617964304 | 4.73E-29 | DOWN |
| ENSSSCG00000012152 | NA | -1.618461915 | 0.000203 | DOWN |
| ENSSSCG00000009283 | TNFRSF19 | -1.625423395 | 1.62E-19 | DOWN |
| ENSSSCG00000003740 | ZSCAN30 | -1.626287041 | 0.000453 | DOWN |
| ENSSSCG00000039089 | NA | -1.629648984 | 0.00048 | DOWN |
| ENSSSCG00000005481 | NA | -1.631093016 | 5.3E-109 | DOWN |
| ENSSSCG00000005378 | ANKS6 | -1.631875303 | 5.22E-62 | DOWN |
| ENSSSCG00000021490 | PRDM16 | -1.633085479 | 1.22E-18 | DOWN |
| ENSSSCG00000005315 | CA9 | -1.634423588 | 3.71E-10 | DOWN |
| ENSSSCG00000017376 | MEOX1 | -1.635230382 | 2.63E-12 | DOWN |
| ENSSSCG00000033941 | C2orf88 | -1.636942043 | 0.020613 | DOWN |
| ENSSSCG00000036206 | C3orf58 | -1.637123734 | 4.57E-67 | DOWN |
| ENSSSCG00000003719 | NA | -1.637940193 | 2.78E-63 | DOWN |
| ENSSSCG00000023376 | PARD6G | -1.638085536 | 1E-12 | DOWN |
| ENSSSCG00000012332 | WNK3 | -1.641370155 | 5.58E-05 | DOWN |
| ENSSSCG00000029998 | KLF7 | -1.647245765 | 1.1E-158 | DOWN |
| ENSSSCG00000007231 | MYLK2 | -1.647943653 | 0.009433 | DOWN |
| ENSSSCG00000016487 | MGAM2 | -1.648733197 | 0.017999 | DOWN |
| ENSSSCG00000016705 | HOXA3 | -1.649481211 | 0.000419 | DOWN |
| ENSSSCG00000036975 | PSRC1 | -1.650472807 | 7.16E-52 | DOWN |
| ENSSSCG00000017391 | PLEKHH3 | -1.650487546 | 3.57E-30 | DOWN |
| ENSSSCG00000000910 | CRADD | -1.656218659 | 1.6E-102 | DOWN |
| ENSSSCG00000023084 | ATP2B2 | -1.663676641 | 0.00897 | DOWN |
| ENSSSCG00000002681 | HSDL1 | -1.664096959 | 1.22E-60 | DOWN |
| ENSSSCG00000034883 | AMIGO1 | -1.665597631 | 3.95E-09 | DOWN |
| ENSSSCG00000016672 | ADCYAP1R1 | -1.665855642 | 0.04233 | DOWN |
| ENSSSCG00000035029 | NOVA2 | -1.66588051 | 0.045993 | DOWN |
| ENSSSCG00000033993 | PLCXD3 | -1.667061764 | 1.07E-38 | DOWN |
| ENSSSCG00000009278 | FGF9 | -1.667955965 | 8.39E-05 | DOWN |
| ENSSSCG00000031977 | PTCH2 | -1.670487074 | 0.01814 | DOWN |
| ENSSSCG00000029323 | KCNE3 | -1.675247948 | 5.88E-06 | DOWN |
| ENSSSCG00000036363 | PDE4C | -1.675472934 | 0.000235 | DOWN |
| ENSSSCG00000015368 | HDAC9 | -1.67657339 | 1.74E-32 | DOWN |
| ENSSSCG00000009089 | ADAD1 | -1.678156208 | 0.023522 | DOWN |
| ENSSSCG00000009389 | TRIM13 | -1.678425768 | 1.16E-41 | DOWN |
| ENSSSCG00000009966 | MN1 | -1.680750277 | 2.07E-13 | DOWN |
| ENSSSCG00000009101 | PRDM5 | -1.681739655 | 2.98E-61 | DOWN |
| ENSSSCG00000031192 | NA | -1.686622694 | 0.026758 | DOWN |
| ENSSSCG00000007022 | ANK1 | -1.687416683 | 0.000325 | DOWN |
| ENSSSCG00000039412 | DOC2B | -1.688720061 | 1.87E-17 | DOWN |
| ENSSSCG00000013049 | RCOR2 | -1.691682374 | 7.99E-80 | DOWN |
| ENSSSCG00000016002 | NA | -1.695227131 | 3.99E-59 | DOWN |
| ENSSSCG00000031915 | ZFP82 | -1.696744004 | 3.1E-06 | DOWN |
| ENSSSCG00000034632 | PDXP | -1.696997442 | 5.57E-10 | DOWN |
| ENSSSCG00000035169 | HOTAIRM1_3 | -1.697938069 | 0.020034 | DOWN |
| ENSSSCG00000031173 | KCNMB4 | -1.699155608 | 0.019211 | DOWN |
| ENSSSCG00000038491 | MEX3B | -1.700805308 | 9.41E-60 | DOWN |
| ENSSSCG00000004548 | PIF1 | -1.701077689 | 1.58E-14 | DOWN |
| ENSSSCG00000010376 | GDF10 | -1.703783578 | 5.64E-11 | DOWN |
| ENSSSCG00000034821 | ARMCX4 | -1.703951578 | 1.78E-16 | DOWN |
| ENSSSCG00000025208 | RNF39 | -1.704658229 | 1.17E-20 | DOWN |
| ENSSSCG00000004974 | LARP6 | -1.706665044 | 1.67E-83 | DOWN |
| ENSSSCG00000000811 | PCED1B | -1.710335796 | 0.009126 | DOWN |
| ENSSSCG00000016857 | DAB2 | -1.711636367 | 1E-218 | DOWN |
| ENSSSCG00000026232 | FAM184B | -1.711653867 | 0.024562 | DOWN |
| ENSSSCG00000023848 | C8orf46 | -1.712635528 | 1.76E-06 | DOWN |
| ENSSSCG00000018513 | ssc-mir-145 | -1.715942305 | 0.043973 | DOWN |
| ENSSSCG00000005045 | BMP4 | -1.716991955 | 1.83E-77 | DOWN |
| ENSSSCG00000002648 | CBFA2T3 | -1.725832048 | 1.63E-13 | DOWN |
| ENSSSCG00000009488 | SLITRK5 | -1.729436456 | 0.018533 | DOWN |
| ENSSSCG00000016684 | SCRN1 | -1.730519051 | 5.11E-89 | DOWN |
| ENSSSCG00000030511 | LGR5 | -1.731376366 | 1.09E-10 | DOWN |
| ENSSSCG00000040392 | NA | -1.734587504 | 9.88E-22 | DOWN |
| ENSSSCG00000040160 | MMP24 | -1.734602114 | 0.013829 | DOWN |
| ENSSSCG00000033065 | NA | -1.735305891 | 0.046552 | DOWN |
| ENSSSCG00000003783 | FPGT | -1.736210515 | 7.73E-18 | DOWN |
| ENSSSCG00000035539 | ST8SIA4 | -1.739575663 | 1.62E-20 | DOWN |
| ENSSSCG00000025992 | NA | -1.739620506 | 0.000473 | DOWN |
| ENSSSCG00000010721 | PSTK | -1.743691321 | 2.75E-09 | DOWN |
| ENSSSCG00000012504 | NAP1L3 | -1.744246877 | 3.09E-30 | DOWN |
| ENSSSCG00000017538 | HOXB7 | -1.746019641 | 0.017208 | DOWN |
| ENSSSCG00000009148 | LEF1 | -1.746730557 | 5.87E-06 | DOWN |
| ENSSSCG00000037099 | ANKLE1 | -1.754825773 | 3.35E-09 | DOWN |
| ENSSSCG00000002258 | KLHL25 | -1.754831716 | 4.09E-14 | DOWN |
| ENSSSCG00000010259 | TYSND1 | -1.757520333 | 1.21E-56 | DOWN |
| ENSSSCG00000017313 | NA | -1.763014637 | 2.71E-17 | DOWN |
| ENSSSCG00000034948 | RASSF9 | -1.765292986 | 3.08E-27 | DOWN |
| ENSSSCG00000039774 | NA | -1.769265836 | 0.002851 | DOWN |
| ENSSSCG00000032094 | DKK2 | -1.769528816 | 1.17E-08 | DOWN |
| ENSSSCG00000001081 | SOX4 | -1.77152613 | 1.3E-126 | DOWN |
| ENSSSCG00000039283 | SIX3 | -1.777052933 | 0.00302 | DOWN |
| ENSSSCG00000021702 | XRRA1 | -1.777179752 | 0.000238 | DOWN |
| ENSSSCG00000031201 | LMOD1 | -1.780465163 | 2.7E-119 | DOWN |
| ENSSSCG00000035497 | NA | -1.786801468 | 2.22E-05 | DOWN |
| ENSSSCG00000026042 | NA | -1.788078266 | 0.029187 | DOWN |
| ENSSSCG00000011743 | MECOM | -1.788146845 | 3.27E-11 | DOWN |
| ENSSSCG00000011125 | GATA3 | -1.79101782 | 5.34E-24 | DOWN |
| ENSSSCG00000005361 | ALDH1B1 | -1.791161431 | 1.39E-65 | DOWN |
| ENSSSCG00000026991 | LURAP1 | -1.792145605 | 2.55E-21 | DOWN |
| ENSSSCG00000032684 | BOK | -1.801182944 | 1.88E-38 | DOWN |
| ENSSSCG00000006121 | PIP4P2 | -1.80572452 | 3.68E-13 | DOWN |
| ENSSSCG00000028529 | REM1 | -1.812768309 | 1.3E-65 | DOWN |
| ENSSSCG00000010556 | PAX2 | -1.81682949 | 0.001166 | DOWN |
| ENSSSCG00000014242 | ZNF608 | -1.831933481 | 7E-189 | DOWN |
| ENSSSCG00000008842 | KIT | -1.833528422 | 0.020978 | DOWN |
| ENSSSCG00000036542 | PPP1R13L | -1.837553562 | 0.001945 | DOWN |
| ENSSSCG00000004990 | LRFN5 | -1.838868689 | 4.84E-06 | DOWN |
| ENSSSCG00000005182 | CCDC171 | -1.840840952 | 0.00129 | DOWN |
| ENSSSCG00000004948 | SMAD6 | -1.841832811 | 7.08E-76 | DOWN |
| ENSSSCG00000031329 | ST8SIA1 | -1.842315181 | 7.6E-104 | DOWN |
| ENSSSCG00000015841 | TEX15 | -1.842554165 | 0.018876 | DOWN |
| ENSSSCG00000038701 | EFNA2 | -1.847375121 | 3.4E-30 | DOWN |
| ENSSSCG00000012768 | ZFP92 | -1.854252596 | 0.000425 | DOWN |
| ENSSSCG00000030485 | ELFN1 | -1.856018034 | 3.06E-29 | DOWN |
| ENSSSCG00000016111 | FZD7 | -1.858972888 | 8.55E-98 | DOWN |
| ENSSSCG00000012950 | RIN1 | -1.861082528 | 8.51E-92 | DOWN |
| ENSSSCG00000023525 | TMEM26 | -1.863681422 | 2.03E-94 | DOWN |
| ENSSSCG00000025590 | NA | -1.863702004 | 0.003091 | DOWN |
| ENSSSCG00000023303 | FIGNL1 | -1.86478046 | 5.39E-25 | DOWN |
| ENSSSCG00000016160 | ERBB4 | -1.865926002 | 0.04563 | DOWN |
| ENSSSCG00000010446 | STAMBPL1 | -1.86640364 | 1.36E-19 | DOWN |
| ENSSSCG00000034013 | TMEM88B | -1.868531317 | 0.043604 | DOWN |
| ENSSSCG00000036437 | NOG | -1.869805681 | 4.44E-20 | DOWN |
| ENSSSCG00000013859 | C19orf44 | -1.875749473 | 2.64E-15 | DOWN |
| ENSSSCG00000025160 | DPF1 | -1.87607826 | 4.04E-11 | DOWN |
| ENSSSCG00000040607 | MAF | -1.876300484 | 1.93E-34 | DOWN |
| ENSSSCG00000028327 | RHOBTB1 | -1.878199168 | 3.37E-25 | DOWN |
| ENSSSCG00000010600 | CALHM2 | -1.883525933 | 7.07E-72 | DOWN |
| ENSSSCG00000033541 | NAT8L | -1.883964917 | 0.004436 | DOWN |
| ENSSSCG00000036201 | NPR3 | -1.885740623 | 3.2E-19 | DOWN |
| ENSSSCG00000008396 | CCDC85A | -1.887972741 | 0.009267 | DOWN |
| ENSSSCG00000004632 | GLDN | -1.890870103 | 5.19E-89 | DOWN |
| ENSSSCG00000031998 | NA | -1.894684998 | 0.008034 | DOWN |
| ENSSSCG00000015986 | HOXD1 | -1.896328705 | 3.59E-27 | DOWN |
| ENSSSCG00000033766 | ETFBKMT | -1.897700661 | 1.25E-09 | DOWN |
| ENSSSCG00000031379 | HOXA11-AS1_1 | -1.899150272 | 0.020994 | DOWN |
| ENSSSCG00000039514 | ID3 | -1.899957306 | 4.72E-88 | DOWN |
| ENSSSCG00000039392 | SNPH | -1.901330545 | 0.000801 | DOWN |
| ENSSSCG00000009111 | SYNPO2 | -1.902730947 | 1.5E-211 | DOWN |
| ENSSSCG00000014909 | NA | -1.905969329 | 5.24E-85 | DOWN |
| ENSSSCG00000017750 | EVI2B | -1.908328734 | 7.06E-06 | DOWN |
| ENSSSCG00000017144 | NPTX1 | -1.911328058 | 7.73E-88 | DOWN |
| ENSSSCG00000024403 | PRRT1 | -1.914146786 | 1.26E-06 | DOWN |
| ENSSSCG00000012371 | AR | -1.921730658 | 5.08E-10 | DOWN |
| ENSSSCG00000011936 | ZBED2 | -1.925549325 | 4.63E-70 | DOWN |
| ENSSSCG00000016548 | NA | -1.926228924 | 0.0123 | DOWN |
| ENSSSCG00000000781 | ABCD2 | -1.927850119 | 0.004601 | DOWN |
| ENSSSCG00000033397 | KCNK9 | -1.930187964 | 0.000518 | DOWN |
| ENSSSCG00000008606 | OSR1 | -1.933187251 | 2.27E-97 | DOWN |
| ENSSSCG00000015281 | PLEKHA6 | -1.935888611 | 1.67E-06 | DOWN |
| ENSSSCG00000032698 | TPPP | -1.941544752 | 0.005556 | DOWN |
| ENSSSCG00000009638 | RHOBTB2 | -1.942440929 | 3.5E-230 | DOWN |
| ENSSSCG00000031799 | TMEM139 | -1.943173541 | 2.19E-06 | DOWN |
| ENSSSCG00000015984 | HOXD4 | -1.945619861 | 3.68E-05 | DOWN |
| ENSSSCG00000038048 | NECAB2 | -1.947788194 | 9.48E-11 | DOWN |
| ENSSSCG00000000260 | SOAT2 | -1.94861375 | 0.002216 | DOWN |
| ENSSSCG00000032327 | TMEM169 | -1.948735917 | 1.11E-09 | DOWN |
| ENSSSCG00000006162 | ZC2HC1A | -1.950508529 | 4.09E-27 | DOWN |
| ENSSSCG00000031565 | NA | -1.951607646 | 9E-12 | DOWN |
| ENSSSCG00000014395 | PCDH12 | -1.952410034 | 1.11E-11 | DOWN |
| ENSSSCG00000034776 | FAM19A5 | -1.956264466 | 0.01578 | DOWN |
| ENSSSCG00000028927 | LGI2 | -1.956385178 | 8.25E-06 | DOWN |
| ENSSSCG00000005773 | RBFA | -1.958589382 | 1.93E-83 | DOWN |
| ENSSSCG00000014076 | FAM169A | -1.959376506 | 0.013445 | DOWN |
| ENSSSCG00000040228 | NA | -1.971526881 | 3.93E-05 | DOWN |
| ENSSSCG00000005480 | NA | -1.971794845 | 0.002835 | DOWN |
| ENSSSCG00000016170 | BARD1 | -1.972420601 | 1.31E-58 | DOWN |
| ENSSSCG00000010086 | NA | -1.972958969 | 0.020699 | DOWN |
| ENSSSCG00000012284 | ZNF182 | -1.973343305 | 2.27E-26 | DOWN |
| ENSSSCG00000004170 | NA | -1.973671743 | 5.66E-15 | DOWN |
| ENSSSCG00000033178 | NA | -1.977741988 | 5.17E-45 | DOWN |
| ENSSSCG00000004201 | TMEM200A | -1.980988997 | 1E-117 | DOWN |
| ENSSSCG00000014892 | USP35 | -1.988615887 | 3.73E-29 | DOWN |
| ENSSSCG00000037354 | MMACHC | -1.994083523 | 3.11E-12 | DOWN |
| ENSSSCG00000017254 | MAP2K6 | -1.994869659 | 0.002299 | DOWN |
| ENSSSCG00000009083 | SPRY1 | -1.998585058 | 7.5E-24 | DOWN |
| ENSSSCG00000007072 | SPTLC3 | -1.999746876 | 2.01E-20 | DOWN |
| ENSSSCG00000004167 | MYB | -2.002012614 | 1.2E-05 | DOWN |
| ENSSSCG00000012164 | CNKSR2 | -2.006698509 | 6.07E-11 | DOWN |
| ENSSSCG00000006729 | FAM46C | -2.010385261 | 1.13E-22 | DOWN |
| ENSSSCG00000011765 | USP13 | -2.014008448 | 9.65E-60 | DOWN |
| ENSSSCG00000009229 | ARHGAP24 | -2.015272519 | 2.44E-35 | DOWN |
| ENSSSCG00000029752 | C16orf54 | -2.017828896 | 2.98E-06 | DOWN |
| ENSSSCG00000023156 | NA | -2.018073253 | 5.41E-08 | DOWN |
| ENSSSCG00000031979 | NA | -2.023361396 | 3.67E-06 | DOWN |
| ENSSSCG00000021941 | NA | -2.023429245 | 0.005296 | DOWN |
| ENSSSCG00000038932 | PPM1E | -2.023900675 | 0.00035 | DOWN |
| ENSSSCG00000016725 | NA | -2.028199806 | 6.53E-83 | DOWN |
| ENSSSCG00000001637 | GUCA1B | -2.029552222 | 1.21E-18 | DOWN |
| ENSSSCG00000030209 | MFNG | -2.029582517 | 0.022092 | DOWN |
| ENSSSCG00000015035 | C11orf52 | -2.033903881 | 0.002384 | DOWN |
| ENSSSCG00000000531 | BICD1 | -2.037809882 | 7.97E-16 | DOWN |
| ENSSSCG00000035355 | F2R | -2.039087987 | 6.86E-27 | DOWN |
| ENSSSCG00000040208 | PAG1 | -2.040424489 | 7.29E-42 | DOWN |
| ENSSSCG00000035682 | ANKRD63 | -2.046016229 | 0.017404 | DOWN |
| ENSSSCG00000037195 | FOXF2 | -2.047776552 | 1.26E-08 | DOWN |
| ENSSSCG00000009676 | ZNF395 | -2.048299041 | 4.88E-22 | DOWN |
| ENSSSCG00000026618 | CAVIN2 | -2.048855072 | 1.24E-07 | DOWN |
| ENSSSCG00000040746 | LRP2 | -2.05109495 | 0.033701 | DOWN |
| ENSSSCG00000037835 | TRIM7 | -2.052885441 | 0.000104 | DOWN |
| ENSSSCG00000021483 | NPBWR1 | -2.052930772 | 2.48E-13 | DOWN |
| ENSSSCG00000011071 | THNSL1 | -2.055599239 | 9.2E-13 | DOWN |
| ENSSSCG00000038580 | NA | -2.056770827 | 3.31E-07 | DOWN |
| ENSSSCG00000002383 | FOS | -2.057362858 | 9.1E-25 | DOWN |
| ENSSSCG00000008187 | KIAA1211L | -2.059433734 | 0.00653 | DOWN |
| ENSSSCG00000027952 | ADCY5 | -2.063208828 | 1.38E-08 | DOWN |
| ENSSSCG00000012952 | TMEM151A | -2.067622138 | 8.34E-17 | DOWN |
| ENSSSCG00000008702 | DOK7 | -2.069921709 | 4.97E-08 | DOWN |
| ENSSSCG00000022247 | PROSER2 | -2.069999307 | 1.49E-21 | DOWN |
| ENSSSCG00000013333 | BDNF | -2.074654394 | 2.1E-05 | DOWN |
| ENSSSCG00000021683 | NA | -2.074800822 | 8.7E-17 | DOWN |
| ENSSSCG00000037015 | SESN3 | -2.075310567 | 4.71E-88 | DOWN |
| ENSSSCG00000034921 | CLDN19 | -2.077815349 | 1.99E-05 | DOWN |
| ENSSSCG00000035891 | ADGRB1 | -2.077944586 | 1.03E-10 | DOWN |
| ENSSSCG00000031706 | MICALCL | -2.078319338 | 9.47E-18 | DOWN |
| ENSSSCG00000009630 | EGR3 | -2.085574892 | 1.56E-17 | DOWN |
| ENSSSCG00000007476 | KCNG1 | -2.08579519 | 9.62E-69 | DOWN |
| ENSSSCG00000016342 | HES6 | -2.087734085 | 4.38E-42 | DOWN |
| ENSSSCG00000038290 | RNF182 | -2.104046166 | 5.52E-09 | DOWN |
| ENSSSCG00000008545 | ZNF512 | -2.107852016 | 1.06E-34 | DOWN |
| ENSSSCG00000016608 | IQUB | -2.108582283 | 0.000138 | DOWN |
| ENSSSCG00000014908 | CCDC89 | -2.11308067 | 3.5E-12 | DOWN |
| ENSSSCG00000005314 | ARHGEF39 | -2.113644891 | 5.29E-38 | DOWN |
| ENSSSCG00000030016 | PDE9A | -2.113824303 | 2.35E-10 | DOWN |
| ENSSSCG00000031272 | FGF5 | -2.123366003 | 0.000977 | DOWN |
| ENSSSCG00000011556 | NA | -2.127903956 | 0.026892 | DOWN |
| ENSSSCG00000035294 | NA | -2.130600469 | 0.000438 | DOWN |
| ENSSSCG00000003715 | NA | -2.138732938 | 3.7E-248 | DOWN |
| ENSSSCG00000002988 | NA | -2.140085155 | 1.18E-16 | DOWN |
| ENSSSCG00000040013 | MTUS1 | -2.142803801 | 2.13E-50 | DOWN |
| ENSSSCG00000004469 | LCA5 | -2.160078414 | 0.000176 | DOWN |
| ENSSSCG00000011514 | MITF | -2.163484691 | 3.32E-50 | DOWN |
| ENSSSCG00000006758 | SYT6 | -2.167775766 | 0.007357 | DOWN |
| ENSSSCG00000033009 | NA | -2.173624953 | 0.000485 | DOWN |
| ENSSSCG00000026417 | EPS8L1 | -2.176426877 | 0.003265 | DOWN |
| ENSSSCG00000038643 | KLF11 | -2.177605471 | 2.98E-07 | DOWN |
| ENSSSCG00000001216 | NA | -2.185836094 | 1.94E-06 | DOWN |
| ENSSSCG00000034184 | NA | -2.189647804 | 8.97E-09 | DOWN |
| ENSSSCG00000001203 | ZSCAN9 | -2.18967705 | 3.08E-05 | DOWN |
| ENSSSCG00000006526 | TRIM46 | -2.191432964 | 2.92E-08 | DOWN |
| ENSSSCG00000017640 | RNF43 | -2.196724303 | 1.58E-11 | DOWN |
| ENSSSCG00000007572 | LFNG | -2.196926275 | 3.14E-30 | DOWN |
| ENSSSCG00000027684 | TRIM63 | -2.199995849 | 3.76E-05 | DOWN |
| ENSSSCG00000032620 | PLCXD2 | -2.200504945 | 8.6E-22 | DOWN |
| ENSSSCG00000021218 | TDRD6 | -2.209003293 | 0.008087 | DOWN |
| ENSSSCG00000014598 | PPFIBP2 | -2.213671805 | 9.01E-31 | DOWN |
| ENSSSCG00000015954 | DLX2 | -2.215272213 | 1.75E-09 | DOWN |
| ENSSSCG00000038598 | ADRB2 | -2.223662558 | 1.09E-09 | DOWN |
| ENSSSCG00000009709 | MFAP3L | -2.228542883 | 0.003754 | DOWN |
| ENSSSCG00000026516 | EPHB3 | -2.233898504 | 4.7E-99 | DOWN |
| ENSSSCG00000032527 | FOSL2 | -2.240551259 | 0 | DOWN |
| ENSSSCG00000017036 | CCNJL | -2.242966075 | 0.036945 | DOWN |
| ENSSSCG00000000991 | FOXQ1 | -2.25066931 | 0.017594 | DOWN |
| ENSSSCG00000005400 | GRIN3A | -2.251439163 | 1.86E-21 | DOWN |
| ENSSSCG00000034474 | HOXC9 | -2.258300804 | 2.18E-07 | DOWN |
| ENSSSCG00000008446 | SIX2 | -2.258783394 | 2.08E-54 | DOWN |
| ENSSSCG00000017223 | USH1G | -2.266185072 | 0.025019 | DOWN |
| ENSSSCG00000032145 | IRX5 | -2.267060649 | 0.000904 | DOWN |
| ENSSSCG00000035720 | HRCT1 | -2.27188218 | 0.017587 | DOWN |
| ENSSSCG00000014095 | ZBED3 | -2.273943639 | 1.69E-06 | DOWN |
| ENSSSCG00000021434 | KRBA2 | -2.274918393 | 0.015936 | DOWN |
| ENSSSCG00000014149 | MEF2C | -2.276932457 | 3.5E-158 | DOWN |
| ENSSSCG00000001538 | DEF6 | -2.288644753 | 0.010074 | DOWN |
| ENSSSCG00000012151 | NHS | -2.292789035 | 2.33E-19 | DOWN |
| ENSSSCG00000036360 | LURAP1L | -2.296324615 | 2.58E-34 | DOWN |
| ENSSSCG00000004109 | ZC3H12D | -2.308884123 | 2.58E-12 | DOWN |
| ENSSSCG00000007986 | NME4 | -2.324734123 | 0.01517 | DOWN |
| ENSSSCG00000004192 | CTGF | -2.329097977 | 2.3E-301 | DOWN |
| ENSSSCG00000037433 | C3orf18 | -2.329301324 | 1.45E-24 | DOWN |
| ENSSSCG00000015780 | STOX2 | -2.332132111 | 1.88E-53 | DOWN |
| ENSSSCG00000036679 | SORBS2 | -2.335068662 | 2.2E-209 | DOWN |
| ENSSSCG00000012508 | NA | -2.34203711 | 4.97E-07 | DOWN |
| ENSSSCG00000032383 | NA | -2.34320507 | 1.68E-18 | DOWN |
| ENSSSCG00000008259 | LRRTM4 | -2.344478921 | 2.88E-37 | DOWN |
| ENSSSCG00000002831 | IRX3 | -2.347015164 | 1.04E-24 | DOWN |
| ENSSSCG00000040638 | DIO2 | -2.349893868 | 4.31E-65 | DOWN |
| ENSSSCG00000028562 | NA | -2.354778092 | 0.003626 | DOWN |
| ENSSSCG00000006034 | RSPO2 | -2.362833312 | 0.001014 | DOWN |
| ENSSSCG00000000688 | LAG3 | -2.367843147 | 0.001209 | DOWN |
| ENSSSCG00000000162 | BTBD11 | -2.368535306 | 3.18E-26 | DOWN |
| ENSSSCG00000024290 | NA | -2.374225532 | 0.000632 | DOWN |
| ENSSSCG00000033979 | KLHL34 | -2.37491861 | 4.87E-21 | DOWN |
| ENSSSCG00000007191 | RAD21L1 | -2.381820556 | 0.002235 | DOWN |
| ENSSSCG00000035987 | EHD3 | -2.385724265 | 5.4E-171 | DOWN |
| ENSSSCG00000002464 | PRIMA1 | -2.387001671 | 3.48E-13 | DOWN |
| ENSSSCG00000033363 | NA | -2.389726468 | 5.41E-09 | DOWN |
| ENSSSCG00000009896 | BICDL1 | -2.390566141 | 8E-06 | DOWN |
| ENSSSCG00000008919 | EPHA5 | -2.393426638 | 1.85E-05 | DOWN |
| ENSSSCG00000010747 | C10orf90 | -2.401748459 | 0.019187 | DOWN |
| ENSSSCG00000033103 | NA | -2.407155943 | 2.12E-06 | DOWN |
| ENSSSCG00000006495 | SEMA4A | -2.411185073 | 3.18E-07 | DOWN |
| ENSSSCG00000010839 | NA | -2.416705345 | 0.013516 | DOWN |
| ENSSSCG00000009729 | ZNF84 | -2.423767784 | 1.64E-25 | DOWN |
| ENSSSCG00000031936 | GJB2 | -2.424044783 | 2.34E-08 | DOWN |
| ENSSSCG00000003921 | ZSWIM5 | -2.432185254 | 5.74E-05 | DOWN |
| ENSSSCG00000030256 | ZNF790 | -2.439494606 | 0.003113 | DOWN |
| ENSSSCG00000000106 | KCNJ4 | -2.441468524 | 0.015006 | DOWN |
| ENSSSCG00000040985 | KCTD7 | -2.444501243 | 3.14E-19 | DOWN |
| ENSSSCG00000024261 | CBX2 | -2.458570081 | 3.92E-33 | DOWN |
| ENSSSCG00000012658 | RAB33A | -2.464764052 | 2.88E-06 | DOWN |
| ENSSSCG00000006530 | EFNA1 | -2.46930668 | 1.54E-18 | DOWN |
| ENSSSCG00000014015 | MRNIP | -2.47252518 | 2.41E-10 | DOWN |
| ENSSSCG00000007305 | SPAG4 | -2.503202982 | 0.003012 | DOWN |
| ENSSSCG00000006874 | PALMD | -2.505002005 | 4.8E-178 | DOWN |
| ENSSSCG00000022705 | SALL2 | -2.506223212 | 1.22E-37 | DOWN |
| ENSSSCG00000000398 | APOF | -2.51659066 | 0.000212 | DOWN |
| ENSSSCG00000001639 | TRERF1 | -2.54948953 | 1.76E-53 | DOWN |
| ENSSSCG00000024685 | GRIA2 | -2.551823588 | 0.040197 | DOWN |
| ENSSSCG00000026718 | PLCH1 | -2.556610426 | 0.015169 | DOWN |
| ENSSSCG00000006068 | FBXO43 | -2.56193157 | 0.005903 | DOWN |
| ENSSSCG00000029838 | FZD2 | -2.577042911 | 9.12E-37 | DOWN |
| ENSSSCG00000011463 | IL17RD | -2.617516724 | 1.3E-144 | DOWN |
| ENSSSCG00000039678 | TMEM269 | -2.619320334 | 0.005375 | DOWN |
| ENSSSCG00000038644 | HOXC8 | -2.622581555 | 7.29E-09 | DOWN |
| ENSSSCG00000030368 | HSPA1L | -2.630295195 | 3.56E-05 | DOWN |
| ENSSSCG00000039821 | GPRIN3 | -2.63384093 | 0.032356 | DOWN |
| ENSSSCG00000037404 | DRGX | -2.636255602 | 1.48E-05 | DOWN |
| ENSSSCG00000039780 | RTN4RL1 | -2.640071948 | 5E-188 | DOWN |
| ENSSSCG00000011695 | AGTR1 | -2.641824766 | 0.034841 | DOWN |
| ENSSSCG00000010209 | FAM13C | -2.644631297 | 9.36E-34 | DOWN |
| ENSSSCG00000022550 | DGKG | -2.655014021 | 2.03E-19 | DOWN |
| ENSSSCG00000013461 | PEAK3 | -2.661370268 | 0.002973 | DOWN |
| ENSSSCG00000039890 | RASL11A | -2.681205529 | 1.36E-15 | DOWN |
| ENSSSCG00000021920 | SOX12 | -2.681943722 | 9.82E-28 | DOWN |
| ENSSSCG00000006780 | WNT2B | -2.683627167 | 2.62E-18 | DOWN |
| ENSSSCG00000006336 | CCDC190 | -2.685032284 | 7.61E-09 | DOWN |
| ENSSSCG00000021601 | ZNF711 | -2.689345472 | 0.011344 | DOWN |
| ENSSSCG00000007552 | NA | -2.701647312 | 6.34E-05 | DOWN |
| ENSSSCG00000008468 | PKDCC | -2.706200789 | 2.02E-77 | DOWN |
| ENSSSCG00000036831 | EFNA4 | -2.745047904 | 0.002847 | DOWN |
| ENSSSCG00000005452 | C9orf152 | -2.751184395 | 0.031812 | DOWN |
| ENSSSCG00000035876 | ZNF599 | -2.813939877 | 2.75E-06 | DOWN |
| ENSSSCG00000009071 | JADE1 | -2.856943875 | 7.4E-116 | DOWN |
| ENSSSCG00000039109 | MTERF2 | -2.877256041 | 1.37E-09 | DOWN |
| ENSSSCG00000005369 | FOXE1 | -2.890854758 | 1.19E-05 | DOWN |
| ENSSSCG00000040031 | SMIM32 | -2.894313139 | 0.01558 | DOWN |
| ENSSSCG00000006235 | TOX | -2.899702935 | 7.66E-56 | DOWN |
| ENSSSCG00000013106 | PTGDR2 | -2.900771666 | 6.61E-05 | DOWN |
| ENSSSCG00000029201 | AJUBA | -2.905994368 | 4.98E-75 | DOWN |
| ENSSSCG00000015873 | ACVR1C | -2.918703147 | 1.35E-06 | DOWN |
| ENSSSCG00000016992 | NEURL1B | -2.933174092 | 7.67E-21 | DOWN |
| ENSSSCG00000023618 | FRMD7 | -2.936399827 | 8.21E-51 | DOWN |
| ENSSSCG00000033509 | SAMD11 | -2.9401966 | 9.75E-87 | DOWN |
| ENSSSCG00000001620 | MDFI | -2.952833714 | 5.7E-172 | DOWN |
| ENSSSCG00000013469 | ZNF555 | -2.95399883 | 0.002784 | DOWN |
| ENSSSCG00000017789 | ABHD15 | -2.963191411 | 3.14E-12 | DOWN |
| ENSSSCG00000037823 | ANKRD34A | -2.968228986 | 0.00019 | DOWN |
| ENSSSCG00000038149 | KCNE4 | -2.996982267 | 1E-109 | DOWN |
| ENSSSCG00000000455 | LRIG3 | -3.014389982 | 1.7E-193 | DOWN |
| ENSSSCG00000033997 | NA | -3.031714086 | 5.01E-06 | DOWN |
| ENSSSCG00000029260 | NDNF | -3.052531114 | 0 | DOWN |
| ENSSSCG00000034262 | FIGN | -3.070820754 | 1.21E-27 | DOWN |
| ENSSSCG00000029753 | CYTIP | -3.080265268 | 5.28E-27 | DOWN |
| ENSSSCG00000032606 | NA | -3.081288272 | 1.31E-09 | DOWN |
| ENSSSCG00000031537 | HAND2 | -3.094016126 | 0.000268 | DOWN |
| ENSSSCG00000032749 | PCDH18 | -3.095760776 | 0 | DOWN |
| ENSSSCG00000038838 | DLX5 | -3.127514213 | 5.3E-121 | DOWN |
| ENSSSCG00000040446 | NA | -3.14577543 | 8.49E-06 | DOWN |
| ENSSSCG00000010461 | ANKRD1 | -3.155374843 | 0.015156 | DOWN |
| ENSSSCG00000012415 | NAP1L2 | -3.247933821 | 4.09E-05 | DOWN |
| ENSSSCG00000029849 | S1PR1 | -3.249318524 | 5.48E-32 | DOWN |
| ENSSSCG00000029324 | COLEC10 | -3.312591493 | 0.010515 | DOWN |
| ENSSSCG00000037066 | GADD45A | -3.318956857 | 0 | DOWN |
| ENSSSCG00000027157 | SLC40A1 | -3.362473223 | 0 | DOWN |
| ENSSSCG00000012785 | PDZD4 | -3.366526321 | 1.14E-06 | DOWN |
| ENSSSCG00000032157 | NA | -3.385486993 | 1.22E-12 | DOWN |
| ENSSSCG00000032446 | C1orf116 | -3.39508981 | 6.12E-10 | DOWN |
| ENSSSCG00000001523 | GRM4 | -3.426540785 | 0.00509 | DOWN |
| ENSSSCG00000011538 | LMCD1 | -3.539589281 | 5.45E-26 | DOWN |
| ENSSSCG00000033314 | DLX6 | -3.547343732 | 1.53E-06 | DOWN |
| ENSSSCG00000006531 | NA | -3.550744546 | 0.000378 | DOWN |
| ENSSSCG00000038527 | NA | -3.619815361 | 0.012903 | DOWN |
| ENSSSCG00000030843 | APLN | -3.652298594 | 5.16E-05 | DOWN |
| ENSSSCG00000016225 | MOGAT1 | -3.699148611 | 0.007767 | DOWN |
| ENSSSCG00000019141 | ssc-mir-365-1 | -3.699914778 | 0.010146 | DOWN |
| ENSSSCG00000036755 | FAM46B | -3.806877161 | 2.25E-59 | DOWN |
| ENSSSCG00000010475 | CYP26A1 | -3.831587725 | 1.28E-22 | DOWN |
| ENSSSCG00000037512 | ZSCAN23 | -3.879084792 | 2.2E-06 | DOWN |
| ENSSSCG00000022289 | PCDH1 | -3.88158101 | 2.13E-91 | DOWN |
| ENSSSCG00000004215 | KIAA0408 | -3.916505316 | 1.11E-18 | DOWN |
| ENSSSCG00000023921 | VSTM2B | -3.94628364 | 4.68E-05 | DOWN |
| ENSSSCG00000008230 | ATOH8 | -3.996846481 | 2.32E-64 | DOWN |
| ENSSSCG00000017879 | SPNS2 | -4.103865809 | 1.26E-14 | DOWN |
| ENSSSCG00000036647 | SPIN4 | -4.138616346 | 0.002889 | DOWN |
| ENSSSCG00000010222 | ZNF365 | -4.15974327 | 5.14E-24 | DOWN |
| ENSSSCG00000033001 | FZD8 | -4.229085751 | 0 | DOWN |
| ENSSSCG00000021624 | LAD1 | -4.274339679 | 0.002901 | DOWN |
| ENSSSCG00000038578 | HRH3 | -4.608264023 | 5.41E-05 | DOWN |
| ENSSSCG00000028341 | NA | -4.664365313 | 0.001081 | DOWN |
| ENSSSCG00000012153 | RAI2 | -4.666235681 | 0.000548 | DOWN |
| ENSSSCG00000036367 | PABPN1L | -4.828171352 | 0.000613 | DOWN |
| ENSSSCG00000034328 | SP7 | -5.156372488 | 1.21E-08 | DOWN |

Gene Name “NA” indicates the gene ID was not matched to a HGNC gene name.
